# Number of People Blind or Visually Impaired by Glaucoma Worldwide and in World Regions 1990 – 2010: A Meta-Analysis

**DOI:** 10.1371/journal.pone.0162229

**Published:** 2016-10-20

**Authors:** Rupert R. A. Bourne, Hugh R. Taylor, Seth R. Flaxman, Jill Keeffe, Janet Leasher, Kovin Naidoo, Konrad Pesudovs, Richard A. White, Tien Y. Wong, Serge Resnikoff, Jost B. Jonas, Vision Loss Expert Group of the Global Burden of Disease Study

**Affiliations:** 1 Vision & Eye Research Unit, Postgraduate Medical Institute, Anglia Ruskin University, Cambridge, United Kingdom; 2 Melbourne School of Population Health, University of Melbourne, Australia; 3 School of Computer Science & Heinz College, Carnegie Mellon University, Pittsburgh, Pennsylvania, United States of America; 4 L V Prasad Eye Institute, Hyderabad, India; 5 Nova Southeastern University, Fort Lauderdale, Florida, United States of America; 6 African Vision Research Institute, University of Kwazulu-Natal, South Africa & Brien Holden Vision Institute, Sydney, Australia; 7 NHMRC Centre for Clinical Eye Research, Flinders University, Adelaide, Australia; 8 Department of Genes and Environment, Division of Epidemiology, Norwegian Institute of Public Health, Oslo, Norway; 9 Singapore Eye Research Institute, Singapore, Singapore; 10 Brien Holden Vision Institute, Sydney, Australia; 11 Department of Ophthalmology, Medical Faculty Mannheim, Heidelberg University, Mannheim, Germany; Purdue University, UNITED STATES

## Abstract

**Objective:**

To assess the number of individuals visually impaired or blind due to glaucoma and to examine regional differences and temporal changes in this parameter for the period from 1990 to 2012.

**Methods:**

As part of the Global Burden of Diseases (GBD) Study 2010, we performed a systematic literature review for the period from 1980 to 2012. We primarily identified 14,908 relevant manuscripts, out of which 243 high-quality, population-based studies remained after review by an expert panel that involved application of selection criteria that dwelt on population representativeness and clarity of visual acuity methods used. Sixty-six specified the proportion attributable to glaucoma. The software tool DisMod-MR (Disease Modeling–Metaregression) of the GBD was used to calculate fraction of vision impairment due to glaucoma.

**Results:**

In 2010, 2.1 million (95% Uncertainty Interval (UI):1.9,2.6) people were blind, and 4.2 (95% UI:3.7,5.8) million were visually impaired due to glaucoma. Glaucoma caused worldwide 6.6% (95% UI:5.9,7.9) of all blindness in 2010 and 2.2% (95% UI:2.0,2.8) of all moderate and severe visual impairment (MSVI). These figures were lower in regions with younger populations (<5% in South Asia) than in high-income regions with relatively old populations (>10%). From 1990 to 2010, the number of blind or visually impaired due to glaucoma increased by 0.8 million (95%UI:0.7, 1.1) or 62% and by 2.3 million (95%UI:2.1,3.5) or 83%, respectively. Percentage of global blindness caused by glaucoma increased between 1990 and 2010 from 4.4% (4.0,5.1) to 6.6%. Age-standardized prevalence of glaucoma related blindness and MSVI did not differ markedly between world regions nor between women.

**Significance:**

By 2010, one out of 15 blind people was blind due to glaucoma, and one of 45 visually impaired people was visually impaired, highlighting the increasing global burden of glaucoma.

## Introduction

Previous population-based investigations have shown that glaucoma is one of the most common, and thus most important, causes for vision loss worldwide [1–34]. Previous estimations of global burden of glaucoma were based on meta-analyses which did not include studies, as far as available, from all regions of the world, which did not include all available population-based studies, which did not assess a change during the last 2 decades, or which mostly reported on the prevalence of the disease [1–3]. Many of the population-based glaucoma studies did not report on the number of people blind or visually impaired due glaucoma. For public health purposes, however, the number of patients functionally affected is more important than the number of patients with any stage of the disease. For the individual patient and thus for the society, the burden of a disease is more important than just the presence of a disease including its early stages. We therefore conducted this meta-analysis of all available population-based studies performed worldwide within the last two decades to estimate the number of people affected by blindness (defined as presenting visual acuity <3/60) and moderately to severe visual impairment (MSVI; presenting visual acuity <6/18, ≥3/60) due to glaucoma, to assess changes in that figures during the period from 1990 to 2010, to examine regional differences in the prevalence of glaucoma related blindness and MSVI, and finally to compare the number of blind and visually impaired people with glaucoma with the number of people blind and visually impaired due to other diseases.

## Methods

**Fig 1:**
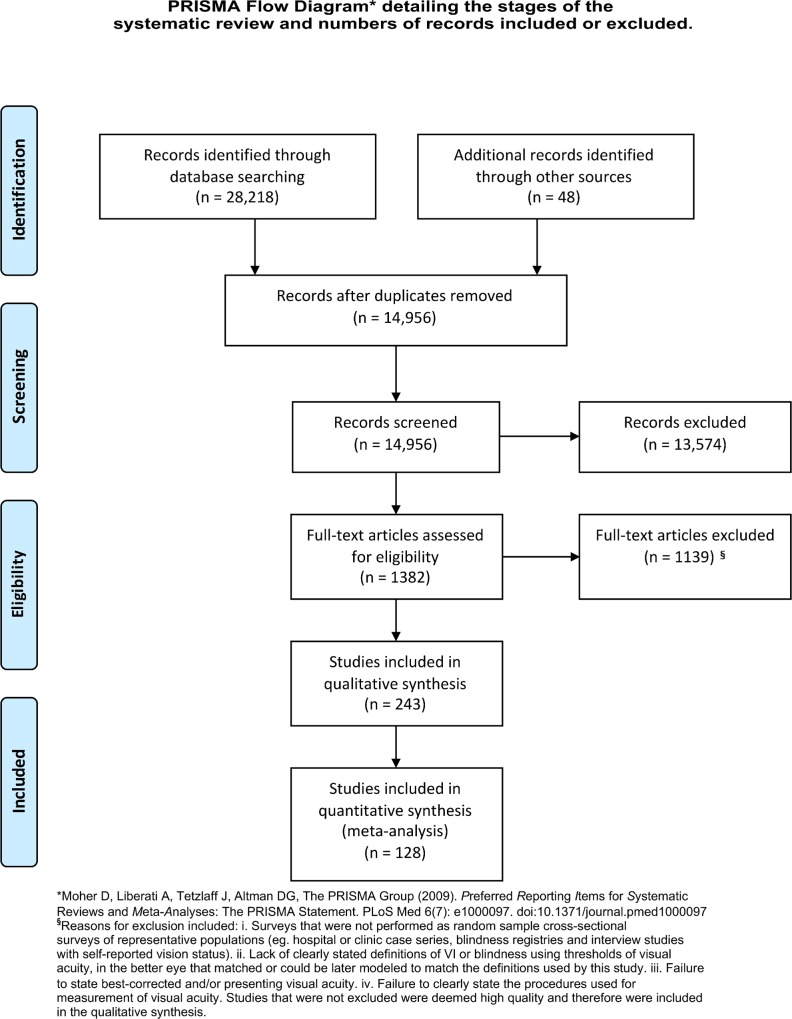
PRISMA (Preferred Reporting Items for Systematic Reviews and Meta-Analyses) flowchart.

In a systemic literature research we used the systems of Medline, Embase and the WHO (World Health Organization) library information system to search for articles on vision loss and published in the period between 1980 and 2012. The methodology for this systematic review is described in Fig 1 as a PRISMA (Preferred Reporting Items for Systematic Reviews and Meta-Analyses) flowchart with a PRISMA checklist in S1 Appendix. The search strategy is presented in S2 Appendix. Out of primarily identified 14,908 relevant manuscripts, we selected 243 high-quality, population-based studies after review by an expert panel. The latter involved application of selection criteria that were based on population representativeness and clarity of visual acuity methods used. As described in detail recently, search terms included concepts to describe “blindness”, “visual impairment”, “population”, “eye”, “survey”, and a list of ocular disorders [35–37]. Additional unpublished data sources were found by personal communication with researchers identified in the literature search. Population-based studies that reported prevalence of visual impairment and blindness disaggregated by cause (128 studies) provided the basic data to calculate the proportion of blindness and MSVI that were due to glaucoma, besides other causes such as cataract, macular degeneration, diabetic retinopathy, trachoma, or undercorrection of refractive error. A full list of data sources used for each cause has been presented recently (Table B in S3 Appendix) [37]. Two studies per region were available for 18 of the 21 GBD (Global Burden of Disease) Study regions, while only one study was identified for Central Europe. Eastern Europe and Central Africa did not have any study with cause-specific data. No study was identified for 126 of 191 countries. Data were extracted from published and unpublished reports into an electronic database (Microsoft Excel) by two investigators working independently with consistency checks in order to minimize data inputting errors. Extracted data included prevalence of predefined severities of vision loss by age, gender, country, region, and cause.

Stratifying by age, sex, and geographical region, we estimated trends in causes of vision impairment and included an analysis of uncertainties. For geographical stratification, we used the 21 regions defined in the GBD Study [38]. As part of the statistical analysis, we first identified and accessed the data and then estimated fractions for each cause. We stratified the results by the severity of vision impairment, sex, age, and region. We finally applied the cause fractions to the prevalence of all-cause presenting vision impairment [37]. The method has been described in detail previously [37].

For the statistical analysis, the Disease Modeling–Metaregression (DisMod-MR) model from the GBD Study was used to determine the fraction of vision impairment caused by glaucoma or due to other causes mentioned above (more detailed information is available in S3 Appendix with an explanation of country and regional groupings, Table A, and a full list of citations of the studies, Table B) [37]. Briefly, the DisMod-MR model is a negative binomial regression model which included the following elements: covariates that predicted the variation in the true proportion of vision impairment from each disease; fixed effects that adjusted for definitional differences (e.g. whether the causes of presenting vs. best-corrected vision impairment were reported); a hierarchical model structure which fitted random intercepts in individual countries derived from the data observed in the country, in its region, and in other regions based on the availability and consistency of country- and region-specific data; age-specific fixed-effects which allowed for a non-linear age pattern; and a fixed effect for data on males. For the assessment of the fractions of blindness and visual impairment which were caused by glaucoma, we fitted one DisMod-MR model and used three covariates: an indicator variable which described whether the data were for blindness or for MSVI, an indicator variable describing whether the data were based on presenting visual acuity or best-corrected visual acuity measurements, and a country-level covariate which reflected the health systems access. We made two sets of the prediction for glaucoma, one for best-corrected blindness and one for best-corrected MSVI. Using the WHO reference population, we age-standardized the prevalences [39]. We also calculated the numbers of people with vision impairment and blindness caused by glaucoma. It reflected each region’s population size and age structure.

## Results

Blindness caused by glaucoma was found to be present in 2.1 million (95% uncertainty interval (UI): 1.9, 2.6) people, and MSVI caused by glaucoma was detected for 4.2 million (95%UI: 3.7–5.8) million individuals (Table 1). Taking into account that overall 32.4 million people were blind and 191 million people were vision impaired in 2010, glaucoma caused worldwide 6.6% (95%UI: 5.9, 7.9) of all causes for blindness in 2010 and 2.2% (95%UI: 2.0, 2.8) of all causes for MSVI (Table 1) [35]. The percentage of blindness caused by glaucoma varied from <5% in South Asia, East and West Sub-Saharan Africa, and Oceania, to 15.5% (9.5–21.9%) in Tropical Latin America.

**Table 1:** Number of people (mean, 95% uncertainty interval) blind (presenting visual acuity <3/60) or visually impaired (MSVI) (presenting visual acuity <6/18, ≥3/60) due to glaucoma and the age-standardized prevalences (mean, 95% uncertainty interval) in different world regions in 2010.

World Region	Blindness / Moderate to Severe Visual Impairment (MSVI) by Glaucoma	Total Population 2010	Number of People Affected in 2010			Age-Standardized Prevalence in People Aged 50+ Years in 2010			Percent of Blindness / Visual Impairment by Glaucoma in 2010
			Mean Value	Lower Value	Upper Value	Mean Value	Lower Value	Upper Value	
World	BLIND	6,890,000,000	2,129,010	1,867,190	2,631,980	0.1%	0.1%	0.2%	6.6 (5.9, 7.9)
Asia Pacific, High Income	BLIND	169,000,000	41,236	21,680	92,060	0.0%	0.0%	0.1%	11.7 (7.1, 18.8)
Asia, Central	BLIND	68,800,000	16,146	10,742	26,920	0.1%	0.1%	0.2%	12.0 (8.7, 17.2)
Asia, East	BLIND	1,190,000,000	280,664	179,792	445,252	0.1%	0.1%	0.1%	5.4 (3.5, 8.5)
Asia, South	BLIND	1,120,000,000	493,126	328,286	787,091	0.2%	0.2%	0.4%	4.7 (3.3, 7.5)
Asia, Southeast	BLIND	460,000,000	195,036	128,088	280,470	0.2%	0.1%	0.3%	5.6 (4.3, 8.2)
Australasia	BLIND	20,500,000	4,359	2,279	12,153	0.0%	0.0%	0.1%	11.3 (6.8, 18.8)
Caribbean	BLIND	34,300,000	21,996	13,683	33,690	0.2%	0.1%	0.3%	11.2 (8.0, 15.1)
Europe, Central	BLIND	122,000,000	40,894	28,224	93,954	0.1%	0.1%	0.2%	12.5 (9.1, 17.0)
Europe, Eastern	BLIND	222,000,000	79,285	39,916	154,466	0.1%	0.0%	0.2%	13.5 (8.6, 20.6)
Europe, Western	BLIND	381,000,000	101,391	65,985	159,726	0.0%	0.0%	0.1%	10.6 (8.2, 14.0)
Latin America, Andean	BLIND	38,600,000	22,996	13,920	35,935	0.3%	0.2%	0.4%	11.7 (7.9, 17.1)
Latin America, Central	BLIND	166,000,000	118,569	80,675	177,954	0.3%	0.2%	0.4%	13.0 (9.6, 18.2)
Latin America, Southern	BLIND	48,900,000	28,401	18,116	50,815	0.2%	0.1%	0.3%	12.6 (7.9, 19.3)
Latin America, Tropical	BLIND	154,000,000	123,409	65,267	262,580	0.3%	0.2%	0.6%	15.5 (9.6, 21.9)
North Africa/Middle East	BLIND	301,000,000	300,578	216,888	434,025	0.5%	0.4%	0.7%	9.6 (7.5, 13.2)
North America, High Income	BLIND	281,000,000	50,464	28,478	90,572	0.0%	0.0%	0.1%	10.7 (7.0, 15.7)
Oceania	BLIND	5,814,186	1,375	704	2,694	0.2%	0.1%	0.4%	4.2 (2.5, 7.2)
Sub-Saharan Africa, Central	BLIND	53,400,000	14,663	7,574	34,857	0.2%	0.1%	0.5%	5.2 (3.4, 8.8)
Sub-Saharan Africa, East	BLIND	208,000,000	83,252	57,759	121,613	0.3%	0.2%	0.4%	4.0 (3.1, 5.4)
Sub-Saharan Africa, South	BLIND	52,600,000	21,870	10,551	34,971	0.3%	0.1%	0.4%	7.3 (5.2, 10.4)
Sub-Saharan Africa, West	BLIND	201,000,000	91,532	64,503	129,601	0.3%	0.2%	0.5%	4.4 (3.4, 5.9)
World	MSVI	6,890,000,000	4,209,790	3,693,040	5,808,270	0.3%	0.2%	0.4%	2.2 (2.0, 2.8)
Asia Pacific, High Income	MSVI	169,000,000	72,451	41,009	284,553	0.1%	0.0%	0.3%	3.7 (2.3, 6.9)
Asia, Central	MSVI	68,800,000	42,894	26,157	91,467	0.3%	0.2%	0.6%	3.6 (2.6, 5.4)
Asia, East	MSVI	1,190,000,000	519,648	296,002	911,581	0.2%	0.1%	0.3%	1.6 (0.94, 2.5)
Asia, South	MSVI	1,120,000,000	1,111,183	707,164	1939,554	0.5%	0.3%	0.8%	1.6 (1.0, 2.6)
Asia, Southeast	MSVI	460,000,000	330,064	227,354	687,535	0.3%	0.2%	0.7%	1.8 (1.3, 3.0)
Australasia	MSVI	20,500,000	14,724	6,098	41,810	0.1%	0.1%	0.4%	3.2 (1.9, 5.9)
Caribbean	MSVI	34,300,000	52,416	29,454	85,792	0.5%	0.3%	0.9%	4.3 (3.1, 6.4)
Europe, Central	MSVI	122,000,000	128,461	68,054	265,300	0.3%	0.1%	0.5%	3.9 (2.8, 6.0)
Europe, Eastern	MSVI	222,000,000	263,377	104,141	592,790	0.3%	0.1%	0.7%	4.5 (2.6, 7.7)
Europe, Western	MSVI	381,000,000	252,546	171,693	484,709	0.1%	0.1%	0.2%	3.4 (2.5, 4.9)
Latin America, Andean	MSVI	38,600,000	62,579	36,452	107,134	0.7%	0.4%	1.2%	4.5 (2.9, 7.5)
Latin America, Central	MSVI	166,000,000	234,065	157,738	362,576	0.6%	0.4%	0.9%	4.6 (3.2, 7.1)
Latin America, Southern	MSVI	48,900,000	63,187	36,186	139,233	0.3%	0.2%	0.8%	4.0 (2.5, 6.3)
Latin America, Tropical	MSVI	154,000,000	250,589	132,867	420,236	0.6%	0.3%	1.0%	5.2 (3.2, 8.4)
North Africa/Middle East	MSVI	301,000,000	414,896	262,711	701,481	0.6%	0.4%	1.1%	3.0 (2.1, 4.7)
North America, High Income	MSVI	281,000,000	104,104	68,661	236,663	0.1%	0.0%	0.2%	3.4 (2.3, 5.4)
Oceania	MSVI	5,814,186	3,389	1,669	6,666	0.4%	0.2%	0.7%	1.4 (0.85, 2.5)
Sub-Saharan Africa, Central	MSVI	53,400,000	26,745	14,950	62,450	0.3%	0.2%	0.8%	1.9 (1.2, 3.3)
Sub-Saharan Africa, East	MSVI	208,000,000	105,933	76,706	160,640	0.3%	0.2%	0.5%	1.5 (1.1, 2.2)
Sub-Saharan Africa, Southern	MSVI	52,600,000	24,600	15,208	49,832	0.3%	0.2%	0.5%	2.6 (1.8, 4.0)
Sub-Saharan Africa, West	MSVI	201,000,000	129,427	92,406	222,330	0.4%	0.3%	0.7%	1.8 (1.3, 2.7)

From the baseline in 1990 to 2010 the number of individuals blind due to glaucoma increased by 0.8 million (95%UI: 0.7, 1.1) and the number of individuals with MSVI due to glaucoma increased by 2.3 million (95%UI: 2.1, 3.5) (Tables 1 and 2). If only individuals with an age of 50+ years were included, the number of people blind due to glaucoma increased from 1.3 million (95%UI: 1.2, 1.6) in 1990 to 2.0 million (95%UI: 1.8, 2.5) in 2010, and the number of individuals with glaucoma related visual impairment increased from 1.9 million (95%UI: 1.5, 2.3) in 1990 to 3.8 million (95%UI: 3.3, 5.3) in 2010. Compared with 1990, the percentage of global blindness caused by glaucoma increased from 4.4% (4.0, 5.1) to 6.6% (Tables 1 and 2). World regions with older populations such as the high-income regions, Southern Latin America, and Central and Eastern Europe, as compared to regions with younger populations showed a higher percentage of blindness caused by glaucoma in 1990 and in 2010 (Table 1). The increase in the percentage of global blindness caused by glaucoma from 1990 to 2010 had taken place in all world regions without major difference between them (Tables 1 and 2).

**Table 2:** Number of people (mean, 95% uncertainty interval) blind (presenting visual acuity <3/60) or visually impaired (MSVI) (presenting visual acuity <6/18, ≥3/60) due to glaucoma and the age-standardized prevalences (mean, 95% uncertainty interval) in different world regions in 1990.

Region		Number of People Affected in 1990			Mean Difference in the Number of People Affected 2010–1990			Age-Standardized Prevalence in People Aged 50+ Years in 1990			Percent of Blindness / Visual Impairment by Glaucoma in 1990
		Mean	Lower Value	Upper Value	Mean	Lower Value	Upper Value	Mean	Lower Value	Upper Value	
World	BLIND	1316596	1155344	1573536	812414	711846	1058444	0,2%	0,1%	0,2%	4.4 (4.0, 5.1)
Asia Pacific, High Income	BLIND	24203	13897	48421	17033	7783	43639	0,1%	0,0%	0,1%	9.0 (6.0, 12.8)
Asia, Central	BLIND	18340	12302	28142	-2194	-1560	-1222	0,2%	0,1%	0,3%	9.5 (7.3, 12.6)
Asia, East	BLIND	220.492	145.928	353.111	60172	33864	92141	0,1%	0,1%	0,2%	3.9 (2.6, 5.8)
Asia, South	BLIND	206493	146182	284448	286633	182104	502643	0,2%	0,1%	0,3%	2.4 (1.7, 3.3)
Asia, Southeast	BLIND	102470	68890	141352	92566	59198	139118	0,2%	0,2%	0,3%	3.3 (2.6, 4.4)
Australasia	BLIND	3240	2152	8014	1119	127	4139	0,1%	0,0%	0,1%	9.6 (7.4, 13.1)
Caribbean	BLIND	17989	12161	25590	4007	1522	8100	0,3%	0,2%	0,4%	9.1 (7.3, 11.8)
Europe, Central	BLIND	40973	28957	80175	-79	-733	13779	0,1%	0,1%	0,2%	10.2 (7.9, 13.3)
Europe, Eastern	BLIND	100223	49413	171479	-20938	-9497	-17013	0,2%	0,1%	0,3%	10.8 (7.7, 15.4)
Europe, Western	BLIND	104486	76973	166514	-3095	-10988	-6788	0,1%	0,0%	0,1%	9.0 (7.4, 11.3)
Latin America, Andean	BLIND	12274	7599	18478	10722	6321	17457	0,3%	0,2%	0,5%	6.8 (5.0, 9.6)
Latin America, Central	BLIND	68707	49116	97253	49862	31559	80701	0,4%	0,3%	0,5%	8.6 (6.7, 11.7)
Latin America, Southern	BLIND	20923	13656	33269	7478	4460	17546	0,2%	0,1%	0,3%	9.3 (6.6, 12.6)
Latin America, Tropical	BLIND	64043	33539	128981	59366	31728	133599	0,4%	0,2%	0,8%	9.2 (5.9, 14.0)
North Africa / Middle East	BLIND	156025	100468	225966	144553	116420	208059	0,6%	0,4%	0,8%	5.6 (4.4, 7.6)
North America, High Income	BLIND	40330	25890	65139	10134	2588	25433	0,0%	0,0%	0,1%	9.2 (6.7, 11.9)
Oceania	BLIND	701	368	1267	674	336	1427	0,2%	0,1%	0,3%	2.8 (2.0, 4.1)
Sub-Saharan Africa, Central	BLIND	8359	4813	17587	6304	2761	17270	0,2%	0,1%	0,5%	3.3 (2.4, 4.6)
Sub-Saharan Africa, East	BLIND	42392	30040	56506	40860	27719	65107	0,3%	0,2%	0,4%	2.9 (2.4, 3.6)
Sub-Saharan Africa, Southern	BLIND	15121	7703	22286	6749	2848	12685	0,4%	0,2%	0,5%	5.4 (4.2, 7.3)
Sub-Saharan Africa, West	BLIND	49454	34740	67855	42078	29763	61746	0,3%	0,2%	0,5%	2.9 (2.4, 3.8)
World	MSVI	1880978	1544298	2335496	2328812	2148742	3472774	0,2%	0,2%	0,3%	1.2 (1.1, 1.5)
Asia Pacific, High Income	MSVI	33651	19846	95198	38800	21163	189355	0,1%	0,0%	0,2%	2.3 (1.5, 3.5)
Asia, Central	MSVI	31447	18045	56188	11447	8112	35279	0,3%	0,2%	0,6%	2.3 (1.8, 3.3)
Asia, East	MSVI	267765	139163	435633	251883	156839	475948	0,1%	0,1%	0,2%	0.92 (0.57, 1.5)
Asia, South	MSVI	336127	211531	503752	775056	495633	1435802	0,3%	0,2%	0,4%	0.66 (0.47, 0.90)
Asia, Southeast	MSVI	116945	78579	175589	213119	148775	511946	0,2%	0,2%	0,4%	0.83 (0.65, 1.1)
Australasia	MSVI	9028	4311	20390	5696	1787	21420	0,2%	0,1%	0,3%	2.4 (1.7, 3.4)
Caribbean	MSVI	31364	16368	43633	21052	13086	42159	0,5%	0,3%	0,8%	3.0 (2.2, 4.1)
Europe, Central	MSVI	88984	43131	155615	39477	24923	109685	0,3%	0,1%	0,5%	2.5 (1.9, 3.3)
Europe, Eastern	MSVI	206804	96231	351954	56573	7910	240836	0,3%	0,1%	0,5%	2.7 (1.8, 4.2)
Europe, Western	MSVI	184746	121141	311166	67800	50552	173543	0,1%	0,1%	0,2%	2.3 (1.8, 3.0)
Latin America, Andean	MSVI	22239	12032	34711	40340	24420	72423	0,5%	0,3%	0,8%	2.1 (1.4, 3.0)
Latin America, Central	MSVI	97887	58316	148486	136178	99422	214090	0,5%	0,3%	0,8%	2.5 (1.9, 3.7)
Latin America, Southern	MSVI	34980	20728	64235	28207	15458	74998	0,3%	0,2%	0,6%	2.4 (1.7, 3.7)
Latin America, Tropical	MSVI	102190	51076	171029	148399	81791	249207	0,6%	0,3%	0,9%	2.7 (1.6, 4.5)
North Africa/Middle East	MSVI	139327	87028	195413	275569	175683	506068	0,5%	0,3%	0,7%	1.4 (1.1, 1.9)
North America, High Income	MSVI	67720	46918	122215	36384	21743	114448	0,1%	0,1%	0,1%	2.4 (1.7, 3.4)
Oceania	MSVI	1130	572	1785	2259	1097	4881	0,3%	0,1%	0,4%	0.73 (0.51, 1.1)
Sub-Saharan Africa, Central	MSVI	10799	5902	20538	15946	9048	41912	0,3%	0,1%	0,5%	1.0 (0.71, 1.5)
Sub-Saharan Africa, East	MSVI	41593	30430	57934	64340	46276	102706	0,3%	0,2%	0,4%	0.95 (0.76, 1.2)
Sub-Saharan Africa, Southern	MSVI	10997	7531	17821	13603	7677	32011	0,3%	0,2%	0,4%	1.5 (1.1, 2.3)
Sub-Saharan Africa, West	MSVI	45875	30759	65282	83552	61647	157048	0,3%	0,2%	0,4%	0.93 (0.71, 1.3)

Age-standardized prevalence of glaucoma related blindness was worldwide 0.1% (95%UI: 0.1, 02) in adults aged 50+ years in 2010, and the age-standardized prevalence of MSVI caused by glaucoma was worldwide 0.3% (95%UI: 0.2, 0.4) (Table 1). Compared with 1990, the age-standardized prevalence of glaucoma-related blindness was reduced from 0.2% (95%UI: 0.1, 0.2) to 0.1% and the prevalence of glaucoma-related MSVI increased from 0.2% (95%UI: 0.2, 0.3) to 0.3% (Tables 1 and 2).

With respect to sex, the age-standardized prevalence of glaucoma related blindness among women (0.1%; 95%UI: 0.1, 0.2) and men (0.1%; 95%UI: 0.1, 0.2) did not differ. The same held true for the age-standardized prevalence of MSVI due to glaucoma (0.3% (95%UI: 0.2, 0.4) in women versus 0.3% (95%UI: 0.3, 0.4) in men).

## Discussion

Glaucoma was the cause for blindness in 2.1 million people or 6.6% of overall 32.4 million blind people globally in 2010, and glaucoma was the cause for MSVI in 4.2 million people or 2.2% of overall 191 million people visually impaired in 2010 [36]. These figures are lower than those reported by Quigley and Broman who forecasted in 2005 that in 2010, bilateral glaucoma related blindness would affect 8.4 million people [2]. Quigley and Broman discussed the difference between their estimate and an estimate of 4.4 million that was the most recent estimate at the time by the WHO Vision Group published by Resnikoff et al. [40]. Quigley and Broman argued that the difference was due to methodological issues given that blindness prevalence surveys often assigned the most ‘‘treatable” disease as the primary cause of blindness. It is often assumed that cataract is more treatable than glaucoma, which leads to an underestimation of glaucoma blindness. In the recent analysis of global blindness, glaucoma ranked third together with macular degeneration (both: 6.6% of all blind people globally) after cataract (33.4% of all blind people globally) and undercorrection of refractive error (20.9%) in the list of the most common causes of global blindness [36]. Glaucoma and macular degeneration ranked first in the list of most common irreversible causes of blindness. With respect to MSVI, glaucoma ranked fourth (2.2%) after undercorrection of refractive error (52.9% of all people with MSVI globally), cataract (18.4%) and macular degeneration (2.2%) in the list of the most common causes of MSVI worldwide. These data confirm previous studies and meta-analyses which showed that glaucoma had a prominent ranking in the frequency list of causes for blindness and visual impairment. In contrast to the previous landmark study by Quigley and Broman, glaucoma was ranked third and fourth in our study as compared to being ranked second by Quigley and Broman and more recently Pascolini et al. as cause for blindness worldwide [2,41]. The numbers however also show that on a global perspective, cataract and undercorrection of refractive error are by far more prevalent as causes for blindness and MSVI. Only one out of 15 blind people was blind due to glaucoma, and only one out of 45 visually impaired people was visually impaired due to glaucoma. These figures may suggest that from a public health of view, providing adequate glasses for correction of refractive error and supplying cataract surgery to the blind and visually impaired may be at least as important as glaucoma care. In the recent meta-analysis of population-based studies by Tham and colleagues, the global prevalence of glaucoma in the population aged 40 to 80 years was 3.54% (95% Credible Intervals, 2.09, 5.82), and the number of individuals aged 40 to 80 years and affected by glaucoma worldwide was 64.3 million in the year 2013 [3]. These figures cannot directly be compared with the figures found in our investigation since Tham´s study addressed the number of individuals affected by glaucoma, independently of the stage of the disease, while our study assessed the number of individuals visually impaired or blind due to glaucoma.

The percentage of blindness caused by glaucoma showed regional variations, with relatively low figures in regions with relatively young populations such as South Asia and Sub-Saharan Africa, and with relatively high figures in regions with relatively old populations such as the high-income regions (Tables 1 and 2). It was due to the dependence of the prevalence of glaucoma on age, while other causes for blindness and MSVI, namely undercorrection of refractive error and, to a lesser degree, cataract occurred also in younger groups of the population. These regional differences in the percentage of glaucoma as cause for blindness and MSVI remained mostly unchanged in the period from 1990 to 2010, since the differences in the age structure between the various world regions did not markedly change.

The global number of glaucoma blind increased by 0.8 million in the period from 1990 to 2010, although the age-standardized global prevalence of glaucoma related blindness in adults aged 50+ years decreased from 0.2% to 0.1%. The worldwide demographic transition with increasing population size, substantial increase in the average age in most regions and falling death rates more than outweighed the decrease in the prevalence of glaucoma related blindness so that the absolute numbers increased by 0.8 million or 62% from 1990 to 2010. The global prevalence of glaucoma related MSVI increased from 0.2% to 0.3% from 1990 to 2010, leading to marked increase in the absolute number of people visually impaired by glaucoma by 2.3 million or 83% in the same period. These figures show that, despite the relatively low percentage of glaucoma related blindness and MSVI on all causes of blindness and MSVI, an intensification of measures to address the growing number of people blind or visually impaired by glaucoma appears mandatory.

Expressed in percentage points, the age-standardized global prevalence of cataract, undercorrected refractive error and trachoma showed marked declines between 1990 and 2010 as reported previously [35]. The age-standardized prevalence of glaucoma declined less (for blindness) or even increased slightly (for MSVI). Similar findings were observed for the age-standardized prevalence of macular degeneration and diabetic retinopathy [35]. These developments may indicate a shift in the relative importance of the various diseases as causes for blindness and visual impairment, with a decrease for the major causes of cataract and undercorrection refractive error, which are relatively easily, safely and cost efficiently treatable, unlike diseases such as glaucoma, macular degeneration and diabetic retinopathy for which the therapy takes considerably more time and effort with a markedly lower rate of success.

Globally and in all regions, a larger percentage of blindness and MSVI caused by cataract and macular degeneration occurs in women than in men [36]. Globally, 36% of blindness among women was caused by cataract versus 30% of blindness among men; for MSVI, the figures were 20% versus 16%, respectively. In a similar manner, macular degeneration caused 7.3% of blindness among women versus 5.5% of blindness among men [36]. The glaucoma related blindness and MSVI did not show such marked disparities by sex in our study. This differs from the predictions of Quigley and Broman who estimated that 59% of all people with glaucoma of any stage would be women in 2010.

Literature reviews published by the WHO and the WHO Prevention of Blindness and Deafness program have previously been used to make worldwide estimates of numbers of people blind or with vision impairment. The latest of these studies included literature published in the period from 2000 to 2010 [41]. That analysis was limited to three age groups, with no breakdown by sex, provision of a point estimate for 2010, or estimates for the six WHO epidemiological subregions within a more limited timeframe. Interestingly, the figures for the percentage of blindness and MSVI caused by glaucoma did not markedly differ between Pascolini and Mariotti´s study and our study (glaucoma related blindness: 2% versus 2.2%; glaucoma related MSVI: 8% versus 6.0%) [41].

The present study forms part of a series of investigations on the prevalence and causes of vision loss in different world regions and on the number of individuals affected by MSVI and blindness caused by the major disorders of under-correction of refractive error, cataract, macular degeneration, diabetic retinopathy and glaucomatous optic neuropathy [42–51]. Applying the same statistical method (DisMod-MR) as the previous investigation, the present study addressed the number of individuals affected by glaucoma as cause for their MSVI or blindness. These figures may be of interest and help for politicians to direct financial means for the improvement of public health with respect to MSVI and blindness and to direct financial support for research in specific fields of medicine and in particular of ophthalmology. The previous studies of the series either assessed the worldwide prevalence of MSVI and blindness or examined the number of people affected by the main ocular disorders except for glaucomatous optic neuropathy.

The figure of 2.1 million individuals blind due to glaucoma and of 4.2 million individuals visually impaired due to glaucoma (representing 6.6% of all blindness and 2.2% of all MSVI worldwide) were lower than the figures of 10.8 million people blind and 35.1 million visually impaired due to cataract (representing 33.4% of all blindness and 18.4% of all MSVI worldwide), and also lower than the figures of 6.8 million people blind and 101.2 million people vision impaired due to undercorrected refractive errors (representing 20.9% of all blindness and 52.9% of all MSVI worldwide) [49,51]. The figures of glaucoma associated blindness and MSVI were similar to the numbers of 2.1 million individuals blind and 6.0 million individuals visually impaired due to macular diseases (representing 6.6% of all blindness and 3.1% of all vision impairment) [48]. They were higher than the figures of 0.8 million people were blind due to diabetic retinopathy and 3.7 million visually impaired due to diabetic retinopathy (2.6% of all blindness and 1.9% of all MSVI worldwide) [50].

The design of our study had potential limitations. First, as we discussed in our report of global prevalence of vision loss, a significant limitation was that many country-years lacked data, or there was only sub-national data available [36]. Relatively few national studies reported vision impairment for all ages and for all causes. Second, some data sources did not present the prevalence by age. By imputing age-specific cause fractions we were able to utilize this data with the assumption that the age pattern of the vision impaired in the particular study matched the modeled age pattern in the country where the study was conducted [36]. Third, the majority of population-based studies within the database that reported on vision loss due to glaucoma did not disaggregate their reported findings into glaucoma diagnostic subtypes such as open-angle glaucoma and angle-closure glaucoma, therefore it was not possible to differentiate between glaucoma subtypes in our analysis. Fourth, protocol dictated that population-based studies will report one cause as the principal cause for an individual examined in that individual study, so that causal prevalence can be calculated. In situations where multiple disorders contribute equally to visual loss, only the ‘‘most easily preventable” or the ‘‘most readily curable” cause is usually recorded [52]. This approach can underestimate the impact of diseases such as diabetic retinopathy and glaucoma when a study participant presents with cataract, while underestimating cataract burden when study participants also have an uncorrected refractive error [53]. Finally, the relatively small sample size of some studies meant that the confidence intervals of cause-specific prevalence estimates were relatively large. Our methods did however take sample size into account, so small sample size studies had less influence on the estimates than larger studies. Strengths of this study included the large amount of population-based data accessed and utilized and the trend analysis of causes of vision impairment and blindness, usage of non-linear age trends and modeling of data that were not reported by age, systematic quantitative analysis and reporting of uncertainty intervals. The large size of network of ophthalmologic researchers involved in first identification and then evaluation of data sources allowed access to unpublished materials and permitted us to obtain additional unpublished data from study investigators who had only published summary data, to evaluate all the major vision impairment studies, and to include only studies that met specific inclusion criteria regarding population representativeness and clear description and definition of visual acuity procedures.

## Conclusion

In conclusion, in 2010, 2.1 million people were blind and 4.2 million people were visually impaired due to glaucoma. The number of people blind and visually impaired due to glaucoma increased by 0.8 million people or 62% and by 2.3 million people or 83%, respectively, in the period from 1990 to 2010. The contribution of glaucoma to total blindness and MSVI was higher in high-income regions with relatively older populations. One out of 15 blind people was blind due to glaucoma, and one out of 45 visually impaired people was visually impaired due to glaucoma.

## Supporting Information

S1 Appendix PRISMA (Preferred Reporting Items for Systematic Reviews and Meta-Analyses) checklist. (DOC)

S2 Appendix The Search Strategy for the Systematic Review. (DOCX)

S3 Appendix Data Sources and Data Analysis. (PDF)
