# Supplementary material for: Number of People Blind or Visually Impaired by Glaucoma Worldwide and in World Regions 1990 – 2010: A Meta-Analysis
Source: PLoS One. 2016 Oct 20;11(10):e0162229. doi: 10.1371/journal.pone.0162229 (PMC5072735; doi:10.1371/journal.pone.0162229)
Supplement: S1 Appendix — (DOC) [file pone.0162229.s001.doc]

| **Section/topic** | **#** | **Checklist item** | **Reported on page #** |
| --- | --- | --- | --- |
| **TITLE** | | |  |
| Title | 1 | Identify the report as a systematic review, meta-analysis, or both.  *The report is identified as a systematic review in Abstract and Methods section.* | 3, 4-6 |
| **ABSTRACT** | | |  |
| Structured summary | 2 | Provide a structured summary including, as applicable: background; objectives; data sources; study eligibility criteria, participants, and interventions; study appraisal and synthesis methods; results; limitations; conclusions and implications of key findings; systematic review registration number.  *This summary is given in the abstract.* | 3 |
| **INTRODUCTION** | | |  |
| Rationale | 3 | Describe the rationale for the review in the context of what is already known.  *The rationale is clearly stated in the Introduction section.* | 4 |
| Objectives | 4 | Provide an explicit statement of questions being addressed with reference to participants, interventions, comparisons, outcomes, and study design (PICOS).  *The rationale is clearly stated in the Introduction section.* | 4 |
| **METHODS** | | |  |
| Protocol and registration | 5 | Indicate if a review protocol exists, if and where it can be accessed (e.g., Web address), and, if available, provide registration information including registration number.  *The review protocol is given in Appendix S2* Supporting Information *- see section A ‘Developing the Search Strategy’* | Appendix S2 Supporting Information |
| Eligibility criteria | 6 | Specify study characteristics (e.g., PICOS, length of follow-up) and report characteristics (e.g., years considered, language, publication status) used as criteria for eligibility, giving rationale.  *The eligibility criteria are given in Appendix S2* Supporting Information *- see section A ‘Developing the Search Strategy’* | Appendix S2 Supporting Information |
| Information sources | 7 | Describe all information sources (e.g., databases with dates of coverage, contact with study authors to identify additional studies) in the search and date last searched.  *The information sources are given in Appendix S2* Supporting Information *- see Section A ‘Developing the Search Strategy’ and Section B ‘Final Search Strategies’.* | Appendix S2 Supporting Information |
| Search | 8 | Present full electronic search strategy for at least one database, including any limits used, such that it could be repeated.  *This is given in Appendix S2* Supporting Information *- Section B ‘Final Search Strategies’.* | Appendix S2 Supporting Information |
| Study selection | 9 | State the process for selecting studies (i.e., screening, eligibility, included in systematic review, and, if applicable, included in the meta-analysis).  *The study selection for eligibility and screening is given in the main manuscript under Methods.* | 4-6 |
| Data collection process | 10 | Describe method of data extraction from reports (e.g., piloted forms, independently, in duplicate) and any processes for obtaining and confirming data from investigators.  *This is stated in the Methods section, Paragraph 1.* | 5 |
| Data items | 11 | List and define all variables for which data were sought (e.g., PICOS, funding sources) and any assumptions and simplifications made.  *This is stated in the Methods section, Paragraph 1.* | 5 |
| Risk of bias in individual studies | 12 | Describe methods used for assessing risk of bias of individual studies (including specification of whether this was done at the study or outcome level), and how this information is to be used in any data synthesis.  *The principal risk of bias with data sources resided with data sources that were not population-based, the visual acuity measurement methods were unclear or where the visual acuity cut-offs for vision loss were not compatible with those of this study. These were minimised by exclusion of full-text articles as shown in the PRISMA flowchart (Figure 1) and in the first paragraph of the Methods section that explains the review by the expert panel.* | 4 |
| Summary measures | 13 | State the principal summary measures (e.g., risk ratio, difference in means).  *We estimated trends in causes of vision impairment, including analysis of uncertainties, by age, sex, and geographical region and fractions of blindness and visual impairment due to glaucoma. This is detailed in the Methods section, second paragraph.* | 5 |
| Synthesis of results | 14 | Describe the methods of handling data and combining results of studies, if done, including measures of consistency (e.g., I^2^) for each meta-analysis.  *These are explained in the final paragraph of the Methods section.* | 6 |

Page 1 of 2

| **Section/topic** | **#** | **Checklist item** | **Reported on page #** |
| --- | --- | --- | --- |
| Risk of bias across studies | 15 | Specify any assessment of risk of bias that may affect the cumulative evidence (e.g., publication bias, selective reporting within studies).  *These are described under ‘limitations of the study’ in the final paragraph of the Discussion section.* | 10 |
| Additional analyses | 16 | Describe methods of additional analyses (e.g., sensitivity or subgroup analyses, meta-regression), if done, indicating which were pre-specified.  *These were not performed.* | N/A |
| **RESULTS** | | |  |
| Study selection | 17 | Give numbers of studies screened, assessed for eligibility, and included in the review, with reasons for exclusions at each stage, ideally with a flow diagram.  *This is given as Figure 1 using a PRISMA flowchart.* | Figure 1 |
| Study characteristics | 18 | For each study, present characteristics for which data were extracted (e.g., study size, PICOS, follow-up period) and provide the citations.  *The citations are available in Appendix S3 Table B of* Supporting Information | Appendix S3 Table B of Supporting Information |
| Risk of bias within studies | 19 | Present data on risk of bias of each study and, if available, any outcome level assessment (see item 12).  *We were unable to comment on this beyond the decision to exclude studies that carried a significant risk of bias as detailed in Point 12 of the checklist.* | 4 |
| Results of individual studies | 20 | For all outcomes considered (benefits or harms), present, for each study: (a) simple summary data for each intervention group (b) effect estimates and confidence intervals, ideally with a forest plot.  *In terms of prevalence of vision impairment and blindness, the graphs in Figure H of Appendix S3 of Supporting Information demonstrate the confidence intervals around the prevalence for studies included in the analysis.* | Figure H of Appendix S3 of Supporting Information |
| Synthesis of results | 21 | Present results of each meta-analysis done, including confidence intervals and measures of consistency.  *These results are reported with 95% Uncertainty Intervals in the results section and Tables.* | 6-7 |
| Risk of bias across studies | 22 | Present results of any assessment of risk of bias across studies (see Item 15).  *The statistical model investigated the risk of bias across studies and an account of this can be found in Appendix S3 of Supporting Information* | Appendix S3 of Supporting Information. |
| Additional analysis | 23 | Give results of additional analyses, if done (e.g., sensitivity or subgroup analyses, meta-regression [see Item 16]).  *Not applicable.* | N/A |
| **DISCUSSION** | | |  |
| Summary of evidence | 24 | Summarize the main findings including the strength of evidence for each main outcome; consider their relevance to key groups (e.g., healthcare providers, users, and policy makers).  *This is summarised in the discussions section.* | 7-9 |
| Limitations | 25 | Discuss limitations at study and outcome level (e.g., risk of bias), and at review-level (e.g., incomplete retrieval of identified research, reporting bias).  *The limitations are discussed in the discussion section.* | 10 |
| Conclusions | 26 | Provide a general interpretation of the results in the context of other evidence, and implications for future research.  *Please see the discussion and conclusions section.* | 7-11 |
| **FUNDING** | | |  |
| Funding | 27 | Describe sources of funding for the systematic review and other support (e.g., supply of data); role of funders for the systematic review.  *These are given in the title page of the manuscript* | 2 |

*From:*  Moher D, Liberati A, Tetzlaff J, Altman DG, The PRISMA Group (2009). Preferred Reporting Items for Systematic Reviews and Meta-Analyses: The PRISMA Statement. PLoS Med 6(7): e1000097. doi:10.1371/journal.pmed1000097

For more information, visit: **www.prisma-statement.org**.

Page 2 of 2
