# Supplementary material for: Number of People Blind or Visually Impaired by Glaucoma Worldwide and in World Regions 1990 – 2010: A Meta-Analysis
Source: PLoS One. 2016 Oct 20;11(10):e0162229. doi: 10.1371/journal.pone.0162229 (PMC5072735; doi:10.1371/journal.pone.0162229)
Supplement: S2 Appendix — (DOCX) [file pone.0162229.s002.docx]

**Webappendix: The Search Strategy for the Systematic Review**

**Table of Contents**

## Developing the Search Strategy

1. **FINAL SEARCH STRATEGIES**
2. **DISCUSSION**

## A. Developing the Search Strategy

The search strategy was required to capture epidemiological studies of vision loss and blindness. The date limit for the search was records published in the period 1980 to 2012 inclusive. The search excluded animal studies.

Several approaches to capturing the search concepts were developed and tested out in Ovid MEDLINE. The objective was to achieve a focused strategy that would identify epidemiological studies of blindness. The strategy needed to balance adequate sensitivity (not missing too many relevant records) against reasonable precision (not producing high proportions of irrelevant records) bearing in mind the resource available to process the records produced by the searches. To achieve this balance required the use, in a master strategy for MEDLINE, of the various indexing options that were available. The strategy also had to take account of variability in the way authors describe their research and indexers index research with Medical Subject Headings (MeSH). In an ideal world we would expect all records about the epidemiology of blindness to be indexed with the MeSH and appropriate subheading, for example BLINDNESS/ep. In practice, however, there are many records about that topic that are not indexed in that way. The strategy takes account of such variability in indexing approaches. The strategy also had to account for the fact that the search would be undertaken in both indexed and unindexed records (e.g. MEDLINE In Process records) so had to search for text words (in the title and abstract) as well as MeSH.

As well as the concept of the epidemiology of blindness, the strategy had to retrieve studies about the epidemiology of selected specific eye diseases which might result in blindness or visual impairment.

After several iterations, checking the impact of changes at each stage, a strategy with three elements was agreed as follows for Ovid MEDLINE (see also Box 1).

### Element 1

A search on the concepts of blindness and visual impairment linked to epidemiological terms through three approaches:

1. Precoordination of a MeSH blindness/visual impairment term with its epidemiology subheading, e.g.

*exp blindness/ep*

ii. Searching for the occurrence of a MeSH blindness/visual impairment term along with the occurrence of an epidemiological MeSH term in the same record, e.g.

*exp blindness/ and (incidence/ or prevalence/ or mortality/ or morbidity/)*

iii. Searching for the occurrence of a MeSH blindness/visual impairment term and an epidemiological concept expressed as a text word in the title or abstract of a record, e.g.

*exp blindness/ and (epidemiology or incidence or prevalence or mortality).ti,ab.*

### Element 2

A search for specific eye diseases linked to epidemiological terms and also to terms indicating blindness using the following approaches:

1. Precoordination of a MeSH eye disease heading with the epidemiology subheading and then looking for the occurrence of that combination in the same record as one where blindness/visual impairment is a term in the title or abstract, e.g.

- *conjunctival diseases/ep*
- *exp blindness/ or (blindness or (visual adj3 impair$) or (vision adj3 impair$)).ti,ab.*
- *1 and 2*

1. Searching for the occurrence of a MeSH eye disease heading in the same record as an epidemiological MeSH heading and as blindness/visual impairment as a term in the title or abstract, e.g.

- *conjunctival diseases/ and (incidence/ or prevalence/ or age distribution/ or sex distribution/ or morbidity/)*
- *exp blindness/ or (blindness or (visual adj3 impair$) or (vision adj3 impair$)).ti,ab.*
- *1 and 2*

**iii.** Searching for the occurrence of eye disease text words in close proximity to epidemiology text words in the title and abstract of records, in the same records as blindness MeSH or blindness/visual impairment text words, e.g.

- *(retinitis adj3 (incidence or prevalence or epidemiology or mortality)).ti,ab.*
- *exp blindness/ or (blindness or (visual adj3 impair$) or (vision adj3 impair$)).ti,ab.*
- *1 and 2*

### Element 3

Searching for records that specifically mention population-based eye surveys using a range of synonyms for that concept in the title and abstract, e.g.

*(population adj3 eye adj3 survey$).ti,ab.*

Box 1. Key to Ovid MEDLINE search syntax

Exp Explodes a MeSH to capture more specific MeSH

.ti,ab. Searches for a word in the title and abstract of a record

/ Indicates that the search term is a Medical Subject Heading (MeSH)

/ep Searches for a subheading linked to a MeSH, in this case ‘epidemiology’

And Achieves a Boolean AND combination

Or Achieves a Boolean OR combination

adj3 Adjacency operator, searches for words up to 3 words apart

$ Truncation operator, searches for words beginning with the stem, e.g. impair$ retrieves impair, impairment, impairments, impaired, impairing and impairs

To improve the precision of the search a range of limits and their effects on the numbers of records retrieved were explored:

- Removing studies about animals by using a safe exclusion approach:

*{Result set} NOT (animals/ NOT humans/)*

This approach excludes records which have are coded solely as Animal studies but retains those that are also indexed as Human studies (i.e. have Animals/ as well as Humans/ in the subject indexing). This is an improvement on searching specifically for Human studies because not all records involving human subjects are coded with the MeSH Humans/

- Excluding letters coded in the MEDLINE Publication Type field;
- Excluding comments coded in the MEDLINE Publication Type field;
- Excluding editorials coded in the MEDLINE Publication Type field;
- Excluding clinical trials coded in the MEDLINE Publication Type field;
- Excluding reviews coded in the MEDLINE Publication Type field or as a text word in the title.

The search strategy was also combined with search terms to retrieve records from specific countries. A country listing was provided. Following queries some revisions to the list were made. Israel did not receive a country grouping at that stage.

Once agreed the MEDLINE strategy was translated appropriately for EMBASE.

The search strategy was adapted to run in WHOLIS. WHOLIS uses MeSH for indexing, but, following careful reading of the Help file, does not seem to offer explosions. The Help file seems to indicate that MeSH terms are included in a “words and phrases” search and that parentheses and Boolean operators can be used within a search string. Based on this assessment selected terms were searched for as “words and phrases”. The strategy was adapted to be sensitive.

The following databases were searched in August 2012:

- Ovid MEDLINE(R) In-Process & Other Non-Indexed Citations and Ovid MEDLINE(R) <1950 to Present>;
- Ovid EMBASE <1980 to 2012>;
- WHOLIS library catalogue (http://dosei.who.int/uhtbin/webcat).

The search results from MEDLINE and EMBASE were downloaded in country groupings. Records about Israel were stored in an Israel database for future inclusion in a wider group. Other records, which were not possible to identify easily during the search as belonging to a specific country, were downloaded as a group which was labelled ‘miscellaneous’.

Records were loaded into Endnote bibliographic software as a series of country group specific databases. The WHOLIS records were loaded into a single separate Endnote database.

Records were deduplicated. Animal studies were deleted as they were identified. Records which appeared in the incorrect country group were moved into their relevant country database. Records in the ‘miscellaneous’ database whose country of focus could be easily identified were moved into their relevant country database.

**B. FINAL SEARCH STRATEGIES**

The final search strategies and results are shown below.

## MEDLINE Search

1 exp blindness/ep or exp blindness/mo

2 exp blindness/ and (incidence/ or prevalence/ or mortality/ or morbidity/)

3 exp blindness/ and (age distribution/ or sex distribution/)

4 exp blindness/ and exp eye diseases/ep

5 exp blindness/ and (epidemiology or incidence or prevalence or mortality).ti,ab.

6 (blindness and (epidemiology or incidence or prevalence or mortality)).ti,ab.

7 vision/ep or vision/mo

8 vision/ and (age distribution/ or sex distribution/)

9 (vision and (epidemiology or incidence or prevalence or mortality)).ti,ab.

10 vision/ and (incidence/ or prevalence/ or mortality/ or morbidity/)

11 exp visual acuity/ and (incidence/ or prevalence/ or mortality/ or morbidity/)

12 exp visual acuity/ and (age distribution/ or sex distribution/)

13 (visual acuity and (epidemiology or incidence or prevalence or mortality)).ti,ab.

14 vision, binocular/ and (incidence/ or prevalence/ or morbidity/ or mortality/)

15 vision, binocular/ and (age distribution/ or sex distribution/)

16 ((vision adj3 binocular) and (epidemiology or incidence or prevalence or mortality)).ti,ab.

17 Vision, Low/ep or vision, low/mo

18 vision, low/ and (incidence/ or prevalence/ or mortality/ or morbidity/)

19 vision, low/ and (age distribution/ or sex distribution/)

20 (low vision and (epidemiology or incidence or prevalence or mortality)).ti,ab.

21 Night Blindness/ep or night blindness/mo

22 night blindness/ and (incidence/ or prevalence/ or morbidity/ or mortality/)

23 night blindness/ and (age distribution/ or sex distribution/)

24 Presbyopia/ep or presbyopia/mo

25 presbyopia/ and (incidence/ or prevalence/ or mortality/ or morbidity/)

26 presbyopia/ and (age distribution/ or sex distribution/)

27 (presbyopi$ and (epidemiology or incidence or prevalence or morbidity)).ti,ab.

28 Visually Impaired Persons/ and (incidence/ or prevalence/ or morbidity/ or mortality/)

29 visually impaired persons/ and (age distribution/ or sex distribution/)

30 (visual$ adj3 impair$ adj3 (incidence or prevalence or epidemiolog$ or morbidity)).ti,ab.

31 (vision adj3 impair$ adj3 (incidence or prevalence or epidemiolog$ or morbidity)).ti,ab.

32 ((amaurosis or deaf-blind) adj3 (incidence or prevalence or epidemiolog$ or morbidity)).ti,ab.

33 conjunctival diseases/ep or conjunctival diseases/mo

34 conjunctival diseases/ and (incidence/ or prevalence/ or age distribution/ or sex distribution/ or morbidity/ or mortality/)

35 conjunctivitis/ep, mo

36 conjunctivitis/ and (incidence/ or prevalence/ or age distribution/ or sex distribution/ or morbidity/ or mortality/)

37 ((conjunctivitis or conjunctival) adj3 (incidence or prevalence or epidemiolog$ or mortality)).ti,ab.

38 ophthalmia neonatorum/ep, mo

39 ophthalmia neonatorum/ and (incidence/ or prevalence/ or age distribution/ or sex distribution/ or morbidity/ or mortality/)

40 (ophthalmia neonatorum adj3 (incidence or prevalence or epidemiology or mortality)).ti,ab.

41 trachoma/ep, mo

42 trachoma/ and (incidence/ or prevalence/ or age distribution/ or sex distribution/ or morbidity/ or mortality/)

43 (trachoma adj3 (incidence or prevalence or epidemiology or mortality)).ti,ab.

44 pterygium/ep, mo

45 pterygium/ and (incidence/ or prevalence/ or age distribution/ or sex distribution/ or morbidity/ or mortality/)

46 (pterygium adj3 (incidence or prevalence or epidemiology or mortality)).ti,ab.

47 xerophthalmia/ep, mo

48 xerophthalmia/ and (incidence/ or prevalence/ or age distribution/ or sex distribution/ or morbidity/ or mortality/)

49 (xerophthalmia adj3 (incidence or prevalence or epidemiology or mortality)).ti,ab.

50 corneal diseases/ep, mo or (corneal diseases/ and (incidence/ or prevalence/ or age distribution/ or sex distribution/ or morbidity/ or mortality/))

51 (corneal adj3 disease$ adj3 (incidence or prevalence or epidemiology or mortality)).ti,ab.

52 corneal opacity/ep, mo or (corneal opacity/ and (incidence/ or prevalence/ or age distribution/ or sex distribution/ or morbidity/ or mortality/))

53 (cornea$1 adj3 (opaque$ or opac$) adj3 (incidence or prevalence or epidemiology or mortality)).ti,ab.

54 keratitis/ep, mo or (keratitis/ and (incidence/ or prevalence/ or age distribution/ or sex distribution/ or morbidity/ or mortality/))

55 (keratitis adj3 (incidence or prevalence or epidemiology or mortality)).ti,ab.

56 corneal ulcer/ep, mo or (corneal ulcer/ and (incidence/ or prevalence/ or age distribution/ or sex distribution/ or morbidity/ or mortality/))

57 (cornea$ adj3 ulcer$ adj3 (incidence or prevalence or epidemiology or mortality)).ti,ab.

58 keratoconus/ep, mo or (keratoconus/ and (incidence/ or prevalence/ or age distribution/ or sex distribution/ or morbidity/ or mortality/))

59 (keratoconus adj3 (incidence or prevalence or epidemiology or mortality)).ti,ab.

60 eye diseases, hereditary/ep, mo or (eye diseases, hereditary/ and (incidence/ or prevalence/ or age distribution/ or sex distribution/ or morbidity/ or mortality/)) (40)

61 (hereditary adj3 eye adj3 (incidence or prevalence or epidemiology or mortality)).ti,ab.

62 retinitis pigmentosa/ep, mo or (retinitis pigmentosa/ and (incidence/ or prevalence/ or age distribution/ or sex distribution/ or morbidity/ or mortality/))

63 (retinitis pigmentosa adj3 (incidence or prevalence or epidemiology or mortality)).ti,ab.

64 exp eye infections/ep, mo or (exp eye infections/ and (incidence/ or prevalence/ or age distribution/ or sex distribution/ or morbidity/ or mortality/))

65 (infection$ adj3 eye$1 adj3 (incidence or prevalence or epidemiology or mortality)).ti,ab.

66 lens diseases/ep, mo or (lens diseases/ and (incidence/ or prevalence/ or age distribution/ or sex distribution/ or morbidity/ or mortality/))

67 (lens adj3 disease$ adj3 (incidence or prevalence or epidemiology or mortality)).ti,ab.

68 exp aphakia/ep, mo or (exp aphakia/ and (incidence/ or prevalence/ or age distribution/ or sex distribution/ or morbidity/ or mortality/))

69 (aphakia adj3 (incidence or prevalence or epidemiology or mortality)).ti,ab.

70 cataract/ep, mo or (cataract/ and (incidence/ or prevalence/ or age distribution/ or sex distribution/ or morbidity/ or mortality/))

71 (cataract$ adj3 (incidence or prevalence or epidemiology or mortality)).ti,ab.

72 ocular hypertension/ep, mo or (ocular hypertension/ and (incidence/ or prevalence/ or age distribution/ or sex distribution/ or morbidity/ or mortality/))

73 ((ocular or intraocular) adj hypertens$ adj3 (incidence or prevalence or epidemiology or mortality)).ti,ab.

74 glaucoma/ep, mo or (glaucoma/ and (incidence/ or prevalence/ or age distribution/ or sex distribution/ or morbidity/ or mortality/))

75 glaucoma, angle-closure/ep, mo or (glaucoma, angle-closure/ and (incidence/ or prevalence/ or age distribution/ or sex distribution/ or morbidity/ or mortality/))

76 glaucoma, open-angle/ep, mo or (glaucoma, open-angle/ and (incidence/ or prevalence/ or age distribution/ or sex distribution/ or morbidity/ or mortality/))

77 (glaucoma adj3 (incidence or prevalence or epidemiology or mortality)).ti,ab.

78 optic nerve diseases/ep, mo or (optic nerve diseases/ and (incidence/ or prevalence/ or age distribution/ or sex distribution/ or morbidity/ or mortality/))

79 (optic nerve adj3 (incidence or prevalence or epidemiology or mortality)).ti,ab.

80 exp optic atrophy/ep, mo or (exp optic atrophy/ and (incidence/ or prevalence/ or age distribution/ or sex distribution/ or morbidity/ or mortality/))

81 (optic atrophy adj3 (incidence or prevalence or epidemiology or mortality)).ti,ab.

82 refractive errors/ep, mo or (refractive errors/ and (incidence/ or prevalence/ or age distribution/ or sex distribution/ or morbidity/ or mortality/))

83 (refractive error$ adj3 (incidence or prevalence or epidemiology or mortality)).ti,ab.

84 astigmatism/ep, mo or (astigmatism/ and (incidence/ or prevalence/ or age distribution/ or sex distribution/ or morbidity/ or mortality/))

85 (astigmatism adj3 (incidence or prevalence or epidemiology or mortality)).ti,ab.

86 hyperopia/ep, mo or (hyperopia/ and (incidence/ or prevalence/ or age distribution/ or sex distribution/ or morbidity/ or mortality/))

87 (hyperopia adj3 (incidence or prevalence or epidemiology or mortality)).ti,ab.

88 exp myopia/ep, mo or (exp myopia/ and (incidence/ or prevalence/ or age distribution/ or sex distribution/ or morbidity/ or mortality/))

89 ((myopia or myopic) adj3 (incidence or prevalence or epidemiology or mortality)).ti,ab.

90 retinal diseases/ep, mo or (retinal diseases/ and (incidence/ or prevalence/ or age distribution/ or sex distribution/ or morbidity/ or mortality/))

91 (retina$1 adj3 disease$ adj3 (incidence or prevalence or epidemiology or mortality)).ti,ab.

92 diabetic retinopathy/ep, mo or (diabetic retinopathy/ and (incidence/ or prevalence/ or age distribution/ or sex distribution/ or morbidity/ or mortality/))

93 (diabetic retinopath$ adj3 (incidence or prevalence or epidemiology or mortality)).ti,ab.

94 retinal degeneration/ep, mo or (retinal degeneration/ and (incidence/ or prevalence/ or age distribution/ or sex distribution/ or morbidity/ or mortality/))

95 (retina$1 adj3 degenerat$ adj3 (incidence or prevalence or epidemiology or mortality)).ti,ab.

96 exp macular degeneration/ep, mo or (exp macular degeneration/ and (incidence/ or prevalence/ or age distribution/ or sex distribution/ or morbidity/ or mortality/))

97 (macular adj3 degenerat$ adj3 (incidence or prevalence or epidemiology or mortality)).ti,ab.

98 retinal detachment/ep, mo or (retinal detachment/ and (incidence/ or prevalence/ or age distribution/ or sex distribution/ or morbidity/ or mortality/))

99 (retina$1 adj3 detach$ adj3 (incidence or prevalence or epidemiology or mortality)).ti,ab.

100 retinal vein occlusion/ep, mo or (retinal vein occlusion/ and (incidence/ or prevalence/ or age distribution/ or sex distribution/ or morbidity/ or mortality/))

101 (retina$1 adj3 vein adj3 occlu$ adj3 (incidence or prevalence or epidemiology or mortality)).ti,ab.

102 retinitis/ep, mo or (retinitis/ and (incidence/ or prevalence/ or age distribution/ or sex distribution/ or morbidity/ or mortality/))

103 (retinitis adj3 (incidence or prevalence or epidemiology or mortality)).ti,ab.

104 chorioretinitis/ep, mo or (chorioretinitis/ and (incidence/ or prevalence/ or age distribution/ or sex distribution/ or morbidity/ or mortality/))

105 (chorioretinitis adj3 (incidence or prevalence or epidemiology or mortality)).ti,ab.

106 cytomegalovirus retinitis/ep, mo or (cytomegalovirus retinitis/ and (incidence/ or prevalence/ or age distribution/ or sex distribution/ or morbidity/ or mortality/))

107 retinopathy of prematurity/ep, mo or (retinopathy of prematurity/ and (incidence/ or prevalence/ or age distribution/ or sex distribution/ or morbidity/ or mortality/))

108 (retinopathy adj3 prematurity adj3 (incidence or prevalence or epidemiology or mortality)).ti,ab.

109 exp choroid diseases/ep, mo or (exp choroid diseases/ and (incidence/ or prevalence/ or age distribution/ or sex distribution/ or morbidity/ or mortality/))

110 (choroid adj3 disease$ adj3 (incidence or prevalence or epidemiology or mortality)).ti,ab.

111 exp uveitis/ep, mo or (exp uveitis/ and (incidence/ or prevalence/ or age distribution/ or sex distribution/ or morbidity/ or mortality/))

112 (uveitis adj3 (incidence or prevalence or epidemiology or mortality)).ti,ab.

113 vision disorders/ep, mo or (vision disorders/ and (incidence/ or prevalence/ or age distribution/ or sex distribution/ or morbidity/ or mortality/))

114 amblyopia/ep, mo or (amblyopia/ and (incidence/ or prevalence/ or age distribution/ or sex distribution/ or morbidity/ or mortality/))

115 (amblyopia adj3 (incidence or prevalence or epidemiology or mortality)).ti,ab.

116 (population adj3 eye adj3 survey$).ti,ab.

117 (population adj3 vision adj3 survey$).ti,ab.

118 (population adj3 blindness adj3 survey$).ti,ab.

119 (population adj3 visual adj3 survey$).ti,ab.

120 animals/ not humans/

121 (letter or comment or editorial).pt.

122 (clinical trial or controlled clinical trial).pt.

123 randomized controlled trial.pt.

124 review.pt.

125 exp blindness/ or (blindness or (visual adj3 impair$) or (vision adj3 impair$)).ti,ab.

126 or/1-32

127 or/33-115

128 or/116-119

129 126 or (127 and 125) or 128

130 129 not (120 or 121 or 122 or 123 or 124)

131 limit 130 to yr="1980 - 2012"

132 Brunei/ or Japan/ or Korea/ or Singapore/

133 (Brunei or Japan$ or Korea$ or Singapore$).ti,ab.

134 131 and (132 or 133)

135 Armenia/ or Azerbaijan/ or Kazakhstan/ or "Asia, Central"/ or Kyrgyzstan/ or Mongolia/ or Tajikistan/ or Turkmenistan/ or Uzbekistan/ or USSR/

136 "georgia (republic)"/

137 (Armenia$ or Azerbaijan$ or Kazakhstan$ or Central Asia or Kyrgyzstan$ or Mongolia$ or Tajikistan$ or Turkmenistan$ or Uzbekistan$ or USSR).ti,ab.

138 (Georgia or Georgian).ti,ab. not United States/

139 or/135-138

140 131 and 139

141 exp China/ or Korea/ or Hong Kong/ or Taiwan/ or Macao/ or Far East/

142 (china or Chinese or korea$ or hong kong or Taiwan$ or macao or far east$).ti,ab.

143 131 and (141 or 142)

144 Afghanistan/ or Bangladesh/ or Bhutan/ or exp India/ or Nepal/ or Pakistan/ or "Asia, Western"/

145 (Afghanistan$ or Bangladesh$ or Bhutan$ or India or Indian or Sikkim$ or Nepal$ or Pakistan$ or West Asia$ or western asia).ti,ab.

146 131 and (144 or 145)

147 Cambodia/ or Indonesia/ or Laos/ or Malaysia/ or Myanmar/ or Philippines/ or "Sri Lanka"/ or Thailand/ or East Timor/ or Vietnam/ or "Asia, Southeastern"/ or exp Indian Ocean Islands/ or Micronesia/

148 (Cambodia$ or Indonesia$ or Laos or laotian or Malaysia$ or Mauritius or mauritain or Myanmar or Philippine$ or Seychelles or Sri Lanka$ or Thailand or thai or East Timor$ or Vietnam$ or Southeast$ Asia$1 or south east$ asia$1 or Indian Ocean Islands or Micronesia$ or comoros).ti,ab.

149 131 and (147 or 148)

150 exp Australia/ or New Zealand/

151 (Australia$ or new Zealand$).ti,ab.

152 131 and (150 or 151)

153 Caribbean Region/ or exp West Indies/ or Aruba/ or Belize/ or Bermuda/ or British Virgin Islands/ or Cayman Islands/ or French Guiana/ or Guyana/ or Montserrat/ or Suriname/ or "Turks and Caicos Islands"/

154 (Antigua$ or Caribbean or West Indies or west Indian or Aruba or Bahama$ or Barbados or Beliz$ or Bermuda$ or Virgin Islands or virgin islanders or Cayman Islands or Cayman Islanders or Cuba or cuban$ or Dominica$ or French Guiana$ or Grenada$ or Guadeloupe$ or Guyana$ or Haiti$ or Jamaica$ or Martinique or Montserrat or Antilles or (Saint Kitts and Nevis) or saint kitts or Saint Lucia$ or Saint Vincent or Grenadines or Suriname or Trinidad$ or Tobago$ or Puerto Rico or Puerto Rican$ or (Turks adj3 Caicos)).ti,ab.

155 131 and (153 or 154)

156 Albania/ or Yugoslavia/ or Bulgaria/ or Czechoslovakia/ or Hungary/ or Poland/ or Romania/ or Montenegro/ or Czech Republic/ or Slovakia/ or "Macedonia (republic)"/ or bosnia-herzegovina/ or slovenia/ or croatia/

157 (Albania$ or Yugoslav$ or Bulgaria$ or Czechoslovakia$ or Hungary or hungarian or Poland or polish or Romania$ or Montenegro or montenegran or Czech$1 or Slovakia$ or bosnia$ or slovenia or slovene or croatia$).ti,ab.

158 131 and (156 or 157)

159 exp Baltic States/ or Moldova/ or exp Russia/ or Ukraine/ or Byelarus/ or USSR/

160 (Lithuania$ or estonia$ or Latvia$ or Baltic States or Moldova$ or Russia$ or Ukraine or ukrainian or Byelarus$ or USSR).ti,ab.

161 131 and (159 or 160)

162 Andorra/ or Austria/ or Belgium/ or Finland/ or exp France/ or exp Germany/ or Gibraltar/ or exp Great Britain/ or Greece/ or Iceland/ or Ireland/ or Italy/ or Rome/ or Liechtenstein/ or Luxembourg/ or Mediterranean Region/ or Monaco/ or Netherlands/ or Portugal/ or Azores/ or San Marino/ or exp Scandinavia/ or Spain/ or Switzerland/ or Vatican City/ or Greenland/

163 (Andorra$ or Austria$ or Belgium or belgian or Finland or finnish or France or french or Germany or german or Gibraltar$ or Great Britain or Scotland or Ireland or Wales or Scottish or irish or welsh or Greece or greek or Iceland$ or Eire or Italy or italian or Liechtenstein$ or Luxembourg$ or Mediterranean or Monaco or Monegasque or dutch or Netherlands or Norway or norwegian or Portugal or Portuguese or Azores or San Marino$ or Scandinavia$ or Sweden or Swedish or Spain or Spanish or Switzerland or swiss or Vatican City or Greenland).ti,ab.

164 131 and (162 or 163)

165 Bolivia/ or Ecuador/ or Peru/

166 (Bolivia$ or Ecuador$ or peru or Peruvian).ti,ab.

167 131 and (165 or 166)

168 Colombia/ or (exp Central America/ not Belize/) or Mexico/ or Venezuela/

169 (Colombia$ or Central America$ or costa rica$ or el Salvador$ or guatemal$ or hondura$ or Nicaragua$ or panama$ or Mexico or Mexican or Venezuela$).ti,ab.

170 131 and (168 or 169)

171 Argentina/ or Chile/ or Falkland Islands/ or Uruguay/

172 (Argentina or argentinian or Chile or Chilean or Falkland Island$ or Uruguay$).ti,ab.

173 131 and (171 or 172)

174 Brazil/ or Paraguay/

175 (brazil$ or Paraguay$).ti,ab.

176 131 and (174 or 175) (58)

177 (exp Africa, Northern/ or exp Middle East/) not (Afghanistan/ or Israel/)

178 (Algeria$ or Egypt$ or Libya$ or Moroc$ or Tunisia$ or Northern Africa$ or north Africa$ or Middle East or Bahrain$ or Iran$ or Iraq$ or Jordan$ or Kuwait$ or Lebanon$ or Oman$ or Qatar$ or Saudi Arabia$ or Syria$ or Turkey or Turkish or United Arab Emirates or Yemen$).ti,ab.

179 131 and (177 or 178)

180 exp Canada/ or exp United States/

181 (Canada or Canadian or united states or usa or north american).ti,ab.

182 131 and (180 or 181)

183 exp Pacific Islands/ not Hawaii/

184 (Pacific Island$ or Melanesia$ or Fiji$ or New Caledonia$ or Papua New Guinea$ or Vanuatu$ or Micronesia$ or Guam or Palau or Pitcairn Island$ or Samoa$ or Tonga$).ti,ab.

185 131 and (183 or 184)

186 Angola/ or (exp Africa, Central/ not (Cameroon/ or Chad/))

187 (Angola$ or central Africa$ or Congo or congolese or Equatorial Guinea or Gabon$).ti,ab.

188 131 and (186 or 187)

189 exp Africa, Eastern/ or Madagascar/ or Malawi/ or Mozambique/ or Zambia/

190 (Eastern africa or Burundi$ or Djibouti$ or Eritrea$ or Ethiopia$ or Kenya$ or Rwanda$ or Somalia$ or Sudan$ or Tanzania$ or Uganda$ or madagasc$ or Malawi$ or mozambiqu$ or zambia$).ti,ab.

191 131 and (189 or 190)

192 exp Africa, Southern/ not (Angola/ or Malawi/ or Mozambique/ or Zambia/)

193 (Southern africa$ or Botswana$ or Lesotho or lesothan or Namibia$ or South Africa$ or swazi$ or Zimbabwe$).ti,ab.

194 131 and (192 or 193)

195 (exp Africa, Western/ or Cameroon/ or Chad/ or Atlantic Islands/) not (Azores/ or Bermuda/ or Falkland Islands/)

196 (west Africa$ or western africa$ or Benin$ or Burkina Faso or Cote d'Ivoire or Gambia$ or Ghana$ or Guinea$ or Guinea-Bissau or Liberia$ or Mali$ or Mauritania$ or Niger or Nigeria$ or Senegal$ or Sierra Leone$ or Togo$ or Cameroon$ or chad$).ti,ab.

197 131 and (195 or 196)

198 israel/ or israel.ti,ab.

199 131 and 198 (57)

200 or/134,140,143,146,149,152,155,158,161,164,167,170,173,176,179,182,185,188,191,194,197,199

201 131 not 200

202 201 and chinese.jw.

203 201 and japanese.jw.

204 201 not (202 or 203)

205 204 and indian.jw.

206 204 not 205

207 206 and india.in.

208 206 not 207

209 208 and nepal.in,jw.

210 208 not 209

211 210 and australia$.in,jw.

212 210 not 211

213 212 and new zealand.in,jw.

214 212 not 213

215 214 and (japanese or korean).la.

216 214 not 215

217 216 and chinese.la.

218 216 not 217

219 218 and (polish.la. or poland.in,jw.)

220 218 not 219

221 220 and (german.la. or germany.in. or german.jw. or austria.in. or austrian.jw.)

222 220 not 221

223 222 and (italian.la. or italy.in. or italian.jw. or french.la. or france.in. or french.jw.)

224 222 not 223

225 224 and (swedish.la. or sweden.in. or swedish.jw. or scandinavian.jw.)

226 224 not 225

227 226 and (bolivia or ecuador or peru).in.

228 226 and (argentina or chile).in.

229 226 not 228

230 229 and (brazil or paraguay).in.

231 229 not 230

232 231 and (Algeria$ or Egypt$ or Libya$ or Moroc$ or Tunisia$ or Northern Africa$ or north Africa$ or Middle East or Bahrain$ or Iran$ or Iraq$ or Jordan$ or Kuwait$ or Lebanon$ or Oman$ or Qatar$ or Saudi Arabia$ or Syria$ or Turkey or Turkish or United Arab Emirates or Yemen$).in.

233 231 not 232

234 233 and (canada.in. or canadian.jw.)

235 233 not 234

236 235 and (Eastern africa or Burundi$ or Djibouti$ or Eritrea$ or Ethiopia$ or Kenya$ or Rwanda$ or Somalia$ or Sudan$ or Tanzania$ or Uganda$ or madagasc$ or Malawi$ or mozambiqu$ or zambia$).in.

237 235 not 236

238 237 and (Lithuania$ or estonia$ or Latvia$ or Baltic States or Moldova$ or Russia$ or Ukraine or ukrainian or Byelarus$ or USSR).in.

239 237 not 238

240 239 and (Albania$ or Yugoslav$ or Bulgaria$ or Czechoslovakia$ or Hungary or hungarian or Poland or polish or Romania$ or Montenegro or montenegran or Czech$1 or Slovakia$ or bosnia$ or slovenia or slovene or croatia$).in.

241 239 not 240

242 241 and (Antigua$ or Caribbean or West Indies or west Indian or Aruba or Bahama$ or Barbados or Beliz$ or Bermuda$ or Virgin Islands or virgin islanders or Cayman Islands or Cayman Islanders or Cuba or cuban$ or Dominica$ or French Guiana$ or Grenada$ or Guadeloupe$ or Guyana$ or Haiti$ or Jamaica$ or Martinique or Montserrat or Antilles or (Saint Kitts and Nevis) or saint kitts or Saint Lucia$ or Saint Vincent or Grenadines or Suriname or Trinidad$ or Tobago$ or Puerto Rico or Puerto Rican$ or (Turks adj3 Caicos)).in.

243 241 not 242

244 243 and (Cambodia$ or Indonesia$ or Laos or laotian or Malaysia$ or Mauritius or mauritain or Myanmar or Philippine$ or Seychelles or Sri Lanka$ or Thailand or thai or East Timor$ or Vietnam$ or Southeast$ Asia$1 or south east$ asia$1 or Indian Ocean Islands or Micronesia$ or comoros).in.

245 243 not 244

246 245 and (Afghanistan$ or Bangladesh$ or Bhutan$ or India or Indian or Sikkim$ or Nepal$ or Pakistan$ or West Asia$ or western asia).in.

247 245 not 246

248 247 and (portugal or spain).in.

249 247 not 248

250 249 and usa.in.

251 249 not 250

252 251 and (uk or untied kingdom).ti,in.

253 251 not 252

254 253 and united kingdom.ti,in.

255 253 not 254

256 255 and korea$.in,jw.

257 255 not 256

258 257 and israel$.in,jw.

259 257 not 258

260 259 and serbia.ti,ab.

261 259 not 260

262 261 and japan.in.

263 261 not 262

264 263 and (brasil$ or brazil$).in,ti,ab,jw.

265 263 not 264

266 265 and (Andorra$ or Austria$ or Belgium or belgian or Finland or finnish or France or french or Germany or german or Gibraltar$ or Great Britain or Scotland or Ireland or Wales or Scottish or irish or welsh or Greece or greek or Iceland$ or Eire or Italy or italian or Liechtenstein$ or Luxembourg$ or Mediterranean or Monaco or Monegasque or dutch or Netherlands or Norway or norwegian or Portugal or Portuguese or Azores or San Marino$ or Scandinavia$ or Sweden or Swedish or Spain or Spanish or Switzerland or swiss or Vatican City or Greenland or denmark).in.

267 265 not 266

268 267 and singapore.in,jw.

269 267 not 268

270 269 and espana.in.

271 269 not 270

272 271 and (Colombia$ or Central America$ or costa rica$ or el Salvador$ or guatemal$ or hondura$ or Nicaragua$ or panama$ or Mexico or Mexican or Venezuela$).in.

273 271 not 272

274 273 and taiwan.in.

275 273 not 274

276 275 and espanola.jw.

277 275 not 276

278 277 and (serbian or czech or polish).lg.

279 277 not 278

280 279 and (norwegian or swedish or finnish or icelandic or danish or dutch or flemish or french).lg.

281 279 not 280

282 281 and (harefuah or hebrew).jw,lg.

283 281 not 282

284 283 and slovak.lg.

285 283 not 284

286 285 and pakistan$.jw,in,lg.

287 285 not 286

288 287 and united states.in.

289 287 not 288

290 289 and russian.lg.

291 289 not 290

292 291 and (west Africa$ or western africa$ or Benin$ or Burkina Faso or Cote d'Ivoire or Gambia$ or Ghana$ or Guinea$ or Guinea-Bissau or Liberia$ or Mali$ or Mauritania$ or Niger or Nigeria$ or Senegal$ or Sierra Leone$ or Togo$ or Cameroon$ or chad$).in.

293 291 not 292

294 293 and china.in.

295 293 not 294

296 from 295 keep 1

## EMBASE Search

1 exp blindness/ep

2 exp blindness/ and (incidence/ or prevalence/ or mortality/ or morbidity/)

3 exp blindness/ and (age distribution/ or sex ratio/)

4 exp blindness/ and exp eye disease/ep

5 exp blindness/ and (epidemiology or incidence or prevalence or mortality).ti,ab.

6 (blindness and (epidemiology or incidence or prevalence or mortality)).ti,ab.

7 vision/ and (age distribution/ or sex ratio/)

8 (vision and (epidemiology or incidence or prevalence or mortality)).ti,ab.

9 vision/ and (incidence/ or prevalence/ or mortality/ or morbidity/)

10 exp visual acuity/ and (incidence/ or prevalence/ or mortality/ or morbidity/)

11 exp visual acuity/ and (age distribution/ or sex ratio/)

12 (visual acuity and (epidemiology or incidence or prevalence or mortality)).ti,ab.

13 binocular vision/ and (incidence/ or prevalence/ or morbidity/ or mortality/)

14 binocular vision/ and (age distribution/ or sex ratio/)

15 ((vision adj3 binocular) and (epidemiology or incidence or prevalence or mortality)).ti,ab.

16 visual disorder/ep

17 visual disorder/ and (incidence/ or prevalence/ or mortality/ or morbidity/)

18 visual disorder/ and (age distribution/ or sex ratio/)

19 (low vision and (epidemiology or incidence or prevalence or mortality)).ti,ab.

20 night blindness/ep

21 night blindness/ and (incidence/ or prevalence/ or morbidity/ or mortality/)

22 night blindness/ and (age distribution/ or sex ratio/)

23 presbyopia/ep

24 presbyopia/ and (incidence/ or prevalence/ or mortality/ or morbidity/)

25 presbyopia/ and (age distribution/ or sex ratio/)

26 (presbyopi$ and (epidemiology or incidence or prevalence or morbidity)).ti,ab.

27 (visual$ adj3 impair$).ti,ab. and (incidence/ or prevalence/ or morbidity/ or mortality/)

28 (visual$ adj3 impair$).ti,ab. and (age distribution/ or sex ratio/)

29 (visual$ adj3 impair$ adj3 (incidence or prevalence or epidemiolog$ or morbidity)).ti,ab.

30 (vision adj3 impair$ adj3 (incidence or prevalence or epidemiolog$ or morbidity)).ti,ab.

31 ((amaurosis or deaf-blind) adj3 (incidence or prevalence or epidemiolog$ or morbidity)).ti,ab.

32 conjunctiva disease/ep

33 conjunctiva disease/ and (incidence/ or prevalence/ or age distribution/ or sex ratio/ or morbidity/ or mortality/)

34 conjunctivitis/ep

35 conjunctivitis/ and (incidence/ or prevalence/ or age distribution/ or sex ratio/ or morbidity/ or mortality/)

36 ((conjunctivitis or conjunctival) adj3 (incidence or prevalence or epidemiolog$ or mortality)).ti,ab.

37 newborn ophthalmia/ep

38 newborn ophthalmia/ and (incidence/ or prevalence/ or age distribution/ or sex ratio/ or morbidity/ or mortality/)

39 (ophthalmia adj3 (neonat$ or newborn) adj3 (incidence or prevalence or epidemiology or mortality)).ti,ab.

40 trachoma/ep

41 trachoma/ and (incidence/ or prevalence/ or age distribution/ or sex ratio/ or morbidity/ or mortality/)

42 (trachoma adj3 (incidence or prevalence or epidemiology or mortality)).ti,ab.

43 pterygium/ep

44 pterygium/ and (incidence/ or prevalence/ or age distribution/ or sex ratio/ or morbidity/ or mortality/)

45 (pterygium adj3 (incidence or prevalence or epidemiology or mortality)).ti,ab.

46 xerophthalmia/ep

47 xerophthalmia/ and (incidence/ or prevalence/ or age distribution/ or sex ratio/ or morbidity/ or mortality/)

48 (xerophthalmia adj3 (incidence or prevalence or epidemiology or mortality)).ti,ab.

49 cornea disease/ep

50 cornea disease/ and (incidence/ or prevalence/ or age distribution/ or sex ratio/ or morbidity/ or mortality/)

51 (cornea$ adj3 disease$ adj3 (incidence or prevalence or epidemiology or mortality)).ti,ab.

52 cornea opacity/ep

53 cornea opacity/ and (incidence/ or prevalence/ or age distribution/ or sex ratio/ or morbidity/ or mortality/)

54 (cornea$ adj3 (opaque$ or opac$) adj3 (incidence or prevalence or epidemiology or mortality)).ti,ab.

55 keratitis/ep

56 keratitis/ and (incidence/ or prevalence/ or age distribution/ or sex ratio/ or morbidity/ or mortality/)

57 (keratitis adj3 (incidence or prevalence or epidemiology or mortality)).ti,ab.

58 cornea ulcer/ep

59 cornea ulcer/ and (incidence/ or prevalence/ or age distribution/ or sex ratio/ or morbidity/ or mortality/)

60 (cornea$ adj3 ulcer$ adj3 (incidence or prevalence or epidemiology or mortality)).ti,ab.

61 keratoconus/ep

62 keratoconus/ and (incidence/ or prevalence/ or age distribution/ or sex ratio/ or morbidity/ or mortality/)

63 (keratoconus adj3 (incidence or prevalence or epidemiology or mortality)).ti,ab.

64 (hereditary adj3 eye).ti,ab. and (incidence/ or prevalence/ or age distribution/ or sex distribution/ or morbidity/ or mortality/)

65 (hereditary adj3 eye adj3 (incidence or prevalence or epidemiology or mortality)).ti,ab.

66 retinitis pigmentosa/ep

67 retinitis pigmentosa/ and (incidence/ or prevalence/ or age distribution/ or sex ratio/ or morbidity/ or mortality/)

68 (retinitis pigmentosa adj3 (incidence or prevalence or epidemiology or mortality)).ti,ab.

69 exp eye infection/ep

70 exp eye infection/ and (incidence/ or prevalence/ or age distribution/ or sex ratio/ or morbidity/ or mortality/)

71 (infection$ adj3 eye$1 adj3 (incidence or prevalence or epidemiology or mortality)).ti,ab.

72 lens disease/ep

73 lens disease/ and (incidence/ or prevalence/ or age distribution/ or sex ratio/ or morbidity/ or mortality/)

74 (lens adj3 disease$ adj3 (incidence or prevalence or epidemiology or mortality)).ti,ab.

75 exp aphakia/ep

76 exp aphakia/ and (incidence/ or prevalence/ or age distribution/ or sex ratio/ or morbidity/ or mortality/)

77 (aphakia adj3 (incidence or prevalence or epidemiology or mortality)).ti,ab.

78 exp cataract/ep

79 exp cataract/ and (incidence/ or prevalence/ or age distribution/ or sex ratio/ or morbidity/ or mortality/)

80 (cataract$ adj3 (incidence or prevalence or epidemiology or mortality)).ti,ab.

81 exp glaucoma/ep

82 exp glaucoma/ and (incidence/ or prevalence/ or age distribution/ or sex ratio/ or morbidity/ or mortality/)

83 (glaucoma adj3 (incidence or prevalence or epidemiology or mortality)).ti,ab.

84 ((ocular hypertens$ or intraocular hypertens$) adj3 (incidence or prevalence or epidemiology or mortality)).ti,ab.

85 optic nerve disease/ep

86 optic nerve disease/ and (incidence/ or prevalence/ or age distribution/ or sex ratio/ or morbidity/ or mortality/)

87 (optic nerve adj3 (incidence or prevalence or epidemiology or mortality)).ti,ab.

88 exp optic nerve atrophy/ep

89 exp optic nerve atrophy/ and (incidence/ or prevalence/ or age distribution/ or sex ratio/ or morbidity/ or mortality/)

90 (optic adj3 atroph$ adj3 (incidence or prevalence or epidemiology or mortality)).ti,ab.

91 refraction error/ep

92 refraction error/ and (incidence/ or prevalence/ or age distribution/ or sex ratio/ or morbidity/ or mortality/)

93 (refract$ adj3 error$ adj3 (incidence or prevalence or epidemiology or mortality)).ti,ab.

94 astigmatism/ep

95 astigmatism/ and (incidence/ or prevalence/ or age distribution/ or sex ratio/ or morbidity/ or mortality/)

96 (astigmatism adj3 (incidence or prevalence or epidemiology or mortality)).ti,ab.

97 hypermetropia/ep

98 hypermetropia/ and (incidence/ or prevalence/ or age distribution/ or sex ratio/ or morbidity/ or mortality/)

99 ((hyperopia or hypermetropia) adj3 (incidence or prevalence or epidemiology or mortality)).ti,ab.

100 exp myopia/ep

101 exp myopia/ and (incidence/ or prevalence/ or age distribution/ or sex ratio/ or morbidity/ or mortality/)

102 ((myopia or myopic) adj3 (incidence or prevalence or epidemiology or mortality)).ti,ab.

103 retina disease/ep

104 retina disease/ and (incidence/ or prevalence/ or age distribution/ or sex ratio/ or morbidity/ or mortality/)

105 (retina$1 adj3 disease$ adj3 (incidence or prevalence or epidemiology or mortality)).ti,ab

106 diabetic retinopathy/ep

107 diabetic retinopathy/ and (incidence/ or prevalence/ or age distribution/ or sex distribution/ or morbidity/ or mortality/)

108 (diabetic retinopath$ adj3 (incidence or prevalence or epidemiology or mortality)).ti,ab.

109 retina degeneration/ep

110 retina degeneration/ and (incidence/ or prevalence/ or age distribution/ or sex ratio/ or morbidity/ or mortality/)

111 (retina$1 adj3 degenerat$ adj3 (incidence or prevalence or epidemiology or mortality)).ti,ab.

112 exp retina maculopathy/ep

113 exp retina maculopathy/ and (incidence/ or prevalence/ or age distribution/ or sex ratio/ or morbidity/ or mortality/)

114 (macular adj3 degenerat$ adj3 (incidence or prevalence or epidemiology or mortality)).ti,ab

115 retina detachment/ep

116 retina detachment/ and (incidence/ or prevalence/ or age distribution/ or sex ratio/ or morbidity/ or mortality/)

117 (retina$1 adj3 detach$ adj3 (incidence or prevalence or epidemiology or mortality)).ti,ab.

118 exp retina vein occlusion/ep

119 exp retina vein occlusion/ and (incidence/ or prevalence/ or age distribution/ or sex ratio/ or morbidity/ or mortality/)

120 (retina$1 adj3 vein adj3 occlu$ adj3 (incidence or prevalence or epidemiology or mortality)).ti,ab.

121 exp retinitis/ep

122 exp retinitis/ and (incidence/ or prevalence/ or age distribution/ or sex ratio/ or morbidity/ or mortality/)

123 (retinitis adj3 (incidence or prevalence or epidemiology or mortality)).ti,ab.

124 (chorioretinitis adj3 (incidence or prevalence or epidemiology or mortality)).ti,ab.

125 retrolental fibroplasia/ep

126 retrolental fibroplasia/ and (incidence/ or prevalence/ or age distribution/ or sex ratio/ or morbidity/ or mortality/)

127 (((retinopathy adj3 prematurity) or retrolental fibroplasia) adj3 (incidence or prevalence or epidemiology or mortality)).ti,ab.

128 exp choroid disease/ep

129 exp choroid disease/ and (incidence/ or prevalence/ or age distribution/ or sex ratio/ or morbidity/ or mortality/)

130 (choroid adj3 disease$ adj3 (incidence or prevalence or epidemiology or mortality)).ti,ab.

131 exp uveitis/ep

132 exp uveitis/ and (incidence/ or prevalence/ or age distribution/ or sex ratio/ or morbidity/ or mortality/)

133 (uveitis adj3 (incidence or prevalence or epidemiology or mortality)).ti,ab.

134 visual disorder/ep

135 visual disorder/ and (incidence/ or prevalence/ or age distribution/ or sex ratio/ or morbidity/ or mortality/)

136 amblyopia/ep

137 amblyopia/ and (incidence/ or prevalence/ or age distribution/ or sex distribution/ or morbidity/ or mortality/)

138 (amblyopia adj3 (incidence or prevalence or epidemiology or mortality)).ti,ab.

139 (population adj3 eye adj3 survey$).ti,ab.

140 (population adj3 vision adj3 survey$).ti,ab.

141 (population adj3 blindness adj3 survey$).ti,ab.

142 (population adj3 visual adj3 survey$).ti,ab.

143 or/1-31

144 or/32-138

145 exp blindness/ or (blindness or (visual adj3 impair$) or (vision adj3 impair$)).ti,ab.

146 144 and 145

147 143 or 146 or (or/139-142)

148 limit 147 to (amphibia or ape or bird or cat or cattle or chicken or dog or "ducks and geese" or fish or "frogs and toads" or goat or guinea pig or "hamsters and gerbils" or horse or monkey or mouse or "pigeons and doves" or "rabbits and hares" or rat or reptile or sheep or swine)

149 limit 147 to animal studies

150 limit 147 to animals

151 limit 147 to human

152 (148 or 149 or 150) not 151

153 147 not 152

154 (letter or editorial or review).pt.

155 153 not 154

156 limit 155 to yr="1980 - 2012"

157 japan/ or brunei darussalam/ or singapore/ or korea/ or south korea/

158 (Brunei or Japan$ or Korea$ or Singapore$).ti,ab.

159 157 or 158

160 asia/ or kazakhstan/ or kyrgyzstan/ or tajikistan/ or turkmenistan/ or uzbekistan/ or mongolia/ or armenia/ or azerbaijan/ or "georgia (republic)"/

161 ussr/

162 (Armenia$ or Azerbaijan$ or Kazakhstan$ or Central Asia or Kyrgyzstan$ or Mongolia$ or Tajikistan$ or Turkmenistan$ or Uzbekistan$ or USSR).ti,ab

163 Georgia.ti,ab. not United States/

164 or/160-163

165 far east/ or china/ or macao/ or taiwan/ or hong kong/ or exp korea/

166 (china or Chinese or korea$ or hong kong or Taiwan$ or macao or far east$).ti,ab.

167 165 or 166

168 exp south asia/

169 (Afghanistan$ or Bangladesh$ or Bhutan$ or India or Indian or Sikkim$ or Nepal$ or Pakistan$ or West Asia$ or western asia).ti,ab.

170 168 or 169

171 philippines/ or southeast asia/ or cambodia/ or indonesia/ or laos/ or malaysia/ or myanmar/ or thailand/ or timor-leste/ or viet nam/ or sri lanka/

172 exp indian ocean/ or "federated states of micronesia"/

173 (Cambodia$ or Indonesia$ or Laos or laotian or Malaysia$ or Mauritius or mauritain or Myanmar or Philippine$ or Seychelles or Sri Lanka$ or Thailand or thai or East Timor$ or Vietnam$ or Southeast$ Asia$1 or south east$ asia$1 or Indian Ocean Islands or Micronesia$ or comoros).ti,ab.

174 171 or 172 or 173

175 exp "australia and new zealand"/

176 (Australia$ or new Zealand$).ti,ab.

177 175 or 176

178 bermuda/ or exp caribbean islands/ or belize/ or french guiana/ or guyana/ or suriname/

179 (Antigua$ or Caribbean or West Indies or west Indian or Aruba or Bahama$ or Barbados or Beliz$ or Bermuda$ or Virgin Islands or virgin islanders or Cayman Islands or Cayman Islanders or Cuba or cuban$ or Dominica$ or French Guiana$ or Grenada$ or Guadeloupe$ or Guyana$ or Haiti$ or Jamaica$ or Martinique or Montserrat or Antilles or (Saint Kitts and Nevis) or saint kitts or Saint Lucia$ or Saint Vincent or Grenadines or Suriname or Trinidad$ or Tobago$ or Puerto Rico or Puerto Rican$ or (Turks adj3 Caicos)).ti,ab.

180 178 or 179

181 albania/ or "bosnia and herzegovina"/ or bulgaria/ or croatia/ or czech republic/ or hungary/ or "macedonia (republic)"/ or poland/ or romania/ or slovakia/ or slovenia/ or yugoslavia/ or czechoslovakia/ or exp "yugoslavia (pre-1992)"/

182 (Albania$ or Yugoslav$ or Bulgaria$ or croatia$ or Czechoslovakia$ or Hungary or hungarian or Poland or polish or Romania$ or Montenegro or montenegran or Czech$1 or Slovakia$ or bosnia$ or slovenia or slovene).ti,ab.

183 181 or 182

184 belarus/ or moldova/ or russian federation/ or ukraine/ or exp baltic states/ or ussr/

185 (Lithuania$ or estonia$ or Latvia$ or Baltic States or Moldova$ or Russia$ or Ukraine or ukrainian or Byelarus$ or USSR).ti,ab.

186 184 or 185

187 exp western Europe/

188 atlantic islands/

189 (Andorra$ or Austria$ or Belgium or belgian or Finland or finnish or France or french or Germany or german or Gibraltar$ or Great Britain or Scotland or Ireland or Wales or Scottish or irish or welsh or Greece or greek or Iceland$ or Eire or Italy or italian or Liechtenstein$ or Luxembourg$ or Mediterranean or Monaco or Monegasque or dutch or Netherlands or Norway or norwegian or Portugal or Portuguese or Azores or San Marino$ or Scandinavia$ or Sweden or Swedish or Spain or Spanish or Switzerland or swiss or Vatican City or Greenland).ti,ab.

190 187 or 188 or 189

191 bolivia/ or ecuador/ or peru/

192 (Bolivia$ or Ecuador$ or peru or Peruvian).ti,ab.

193 191 or 192

194 Colombia/ or (exp Central America/ not Belize/) or Mexico/ or Venezuela/

195 (Colombia$ or Central America$ or costa rica$ or el Salvador$ or guatemal$ or hondura$ or Nicaragua$ or panama$ or Mexico or Mexican or Venezuela$).ti,ab.

196 194 or 195

197 Argentina/ or Chile/ or Falkland Islands/ or Uruguay/

198 (Argentina or argentinian or Chile or Chilean or Falkland Island$ or Uruguay$).ti,ab.

199 197 or 198

200 Brazil/ or Paraguay/ or (brazil$ or Paraguay$).ti,ab.

201 (exp north africa/ or exp middle east/) not israel/

202 (Algeria$ or Egypt$ or Libya$ or Moroc$ or Tunisia$ or Northern Africa$ or north Africa$ or Middle East or Bahrain$ or Iran$ or Iraq$ or Jordan$ or Kuwait$ or Lebanon$ or Oman$ or Qatar$ or Saudi Arabia$ or Syria$ or Turkey or Turkish or United Arab Emirates or Yemen$).ti,ab.

203 201 or 202

204 canada/ or united states/

205 (Canada or Canadian or united states or usa).ti,ab.

206 204 or 205

207 exp pacific islands/

208 (Pacific Island$ or Melanesia$ or Fiji$ or New Caledonia$ or Papua New Guinea$ or Vanuatu$ or Micronesia$ or Guam or Palau or Pitcairn Island$ or Samoa$ or Tonga$).ti,ab.

209 207 or 208

210 angola/ or central africa/ or central african republic/ or congo/ or equatorial guinea/ or gabon/

211 (Angola$ or central Africa$ or Congo or congolese or Equatorial Guinea or Gabon$).ti,ab.

212 210 or 211

213 burundi/ or djibouti/ or eritrea/ or ethiopia/ or kenya/ or madagascar/ or malawi/ or mozambique/ or rwanda/ or somalia/ or sudan/ or tanzania/ or uganda/ or zambia/

214 (East$ africa or Burundi$ or Djibouti$ or Eritrea$ or Ethiopia$ or Kenya$ or Rwanda$ or Somalia$ or Sudan$ or Tanzania$ or Uganda$ or madagasc$ or Malawi$ or mozambiqu$ or zambia$).ti,ab.

215 213 or 214

216 botswana/ or lesotho/ or namibia/ or south africa/ or swaziland/ or zimbabwe/

217 (Southern africa$ or Botswana$ or Lesotho or lesothan or Namibia$ or South Africa$ or swazi$ or Zimbabwe$).ti,ab.

218 216 or 217

219 africa/ or benin/ or burkina faso/ or cameroon/ or chad/ or cote d'ivoire/ or gambia/ or ghana/ or guinea/ or guinea-bissau/ or liberia/ or mali/ or niger/ or nigeria/ or senegal/ or sierra leone/ or togo/ or mauritania/ or saint helena/ or "sao tome and principe"/

220 (west Africa$ or western africa$ or Benin$ or Burkina Faso or Cote d'Ivoire or Gambia$ or Ghana$ or Guinea$ or Guinea-Bissau or Liberia$ or Mali$ or Mauritania$ or Niger or Nigeria$ or Senegal$ or Sierra Leone$ or Togo$ or Cameroon$ or chad$).ti,ab.

221 219 or 220

222 israel/ or israel$.ti,ab.

223 156 and 159

224 156 and 164

225 156 and 167

226 156 and 170

227 156 and 174

228 156 and 177

229 156 and 180

230 156 and 183

231 156 and 186

232 156 and 190

233 156 and 193

234 156 and 196

235 156 and 199

236 156 and 200

237 156 and 203

238 156 and 206

239 156 and 209

240 156 and 212

241 156 and 215

242 156 and 218

243 156 and 221

244 156 and 222

245 or/223-244

246 156 not 245

## WHOLIS Search

The WHOLIS strategy was adapted because of the interface and the search facilities available. The adaptation should have been very sensitive.

1. blindness or visual or vision or acuity or amaurosis
2. conjunctiv$ or ophthalmia or trachoma or pterygium or xerophthalmia or cornea$ or keratitis or keratoconus or (eye disease$) or (eye infect$) or lens or aphakia or cataract$ or (ocular hypertens$) or (intraocular hypertens$)
3. glaucoma or (optic nerve$) or (optic atrophy) or (refract$ error$) or astigmatism or hyperopia or hypermetropia or myopia or retinitis or retina$ or retinopathy or (macular degeneration) or chorioretinitis or choroid or uveitis or amblyopia
4. epidemiology or incidence or prevalence or mortality or (age distibution) or (sex distribution)

All limited to 1980–2012.

**C. DISCUSSION**

All searches are compromises. Strategies trade off sensitivity against precision, and decisions are made by research teams about the numbers of records that they are able to process in the time available. The strategy developed is reasonably sensitive and precise. It could have been made more sensitive, for example by increasing the value of the adj operator (so that words could occur further apart but still be retrieved), by using AND rather than adj or by using ‘ep’ as a floating subheading that is not linked to specific MeSH. Sensitivity might also have been increased by identifying more synonyms and spelling variants for the eye diseases which may result in blindness. These approaches would have generated many more studies to assess for relevance, but might have retrieved additional relevant studies.

The strategy could also have been made more precise perhaps by omitting some of the combinations of eye diseases and epidemiology terms as text words in the title and abstract. However, increasing search precision usually risks losing relevant studies.
