# Supplementary material for: Number of People Blind or Visually Impaired by Glaucoma Worldwide and in World Regions 1990 – 2010: A Meta-Analysis
Source: PLoS One. 2016 Oct 20;11(10):e0162229. doi: 10.1371/journal.pone.0162229 (PMC5072735; doi:10.1371/journal.pone.0162229)
Supplement: S3 Appendix — (PDF) [file pone.0162229.s003.pdf]

Text A.Definitions and analytic framework

We aim to calculate the prevalence of mild distance vision loss, severe distance vision loss, and distance blindness, by age, sex and year for the period 1990-2010 in 190 countries (Table A). We define the following *extended* vision loss categories:

- Blind: $< 3/60$ and/or a visual field of no greater than 10 in radius around central fixation
- Severe vision loss: $< 6/60$ and $\geq 3/60$
- Moderate vision loss: $< 6/18$ and $\geq 6/60$
- Mild vision loss: $< 6/60$ and $\geq 6/18$

Studies that report severe, moderate, and mild vision loss were sparse compared to studies that report the prevalence of blindness and/or severe and moderate vision loss combined (MSVI). At the same time, the prevalence of severe, moderate and mild vision loss are highly correlated with MSVI. We therefore chose to create models for two *core* levels of visual impairment:

- Blind: $< 3/60$ and/or a visual field of no greater than 10 in radius around central fixation
- MSVI: $< 6/18$ and $\geq 3/60$

Our study was carried out in five steps:

- Data identification and access (Text B)
- Conversion of vision impairment data to two core severity levels for modeling, blindness and MSVI (Text C)
- Application of an age pattern to data which are not presented by age (Text D)
- Development of a hierarchical model to estimate prevalence of two core levels of vision impairment (Text E)
- Conversion of vision impairment data from two core levels to four extended severity levels (Text F)

Text B. Data identification and access

We used data from epidemiologic studies and surveys identified in a previously published systematic review[1]. Bourne *et al.* identified studies or surveys fulfilling the following inclusion criteria:

- The studies reported prevalence of visual impairment from cross-sectional surveys of representative populations of a country or area of a country. Studies that targetted high-prevalence areas, such as trachoma or onchocerciasis-endemic villages, were excluded.
- The definitions of visual impairment were clearly stated, using thresholds of visual acuity in the better eye that matched the extended definitions of visual impairment.
- Best corrected and/or presenting visual acuity was given.
- The procedures used for measurement of visual acuity were clearly stated.

The authors carried out a systematic review of the literature. They also obtained unpublished data sources. In total, they identified 243 studies or surveys fulfilling their inclusion criteria. We excluded 1 study because it measured only near vision impairment; 3 studies because they only measured incidence of distance vision impairment; 7 studies because they did not report all-cause vision impairment; and 1 study from Cook Islands because covariate data were not available. Finally, we excluded 4 studies because they only reported visual acuity levels which we did not map to our core visual acuity levels (see section 3 for further details). Thus, we used data from 227 studies; 216 reported or could be used to estimate the prevalence of blindness and 192 reported or could be used to estimate the prevalence of MSVI. Data used in this analysis are listed in Table B.

Text C. Methods for comparability of vision impairment definition

Not all prevalence data reported in the literature use the definitions of visual impairment selected for this analysis. To include data calculated using other definitions of visual impairment, we developed statistical models to convert the prevalence of visual impairment using other severity thresholds to the thresholds used in this analysis.

Our aim is thus to convert all prevalence data reported in studies to our core levels of visual impairment, blindness and MSVI. We first pooled all study data reporting visual impairment using multiple definitions. We then fit four logistic regressions to convert data using alternative definitions of visual impairment to our core definitions, as follows:

Definitions of blindness (P_X) converted to our core definition of blindness (P_Y; $< 3/60$):

- $< 6/60$ (see Figure A)
- $\leq 6/60$ (see Figure B)

Definitions of visual impairment (P_X) converted to our core definition of visual impairment (P_Y; $< 6/18$ & $\geq 3/60$):

- $< 6/18$ (see Figure C)
- $< 6/12$ (see Figure D)

Each equation had the following form:

$$\text{logit}(P_Y) = \beta \cdot \text{logit}(P_X) + \alpha$$

Fitted regression coefficients are reported in Table C. The converted data (blind and MSVI) are then used in the models to predict the prevalence of vision impairment by country, age, sex, and year.

Text D. Methods for comparability of data aggregated by age

Vision loss has a strong age pattern; because of this, using the midpoint on wide-age data may be inappropriate. To allow data with wide age-ranges to be included in the model we imputed narrow-age prevalences and then used those to model vision impairment by country, year, age and sex. We first found a universal age pattern and then applied it to the wide age-range data, which allowed us to split the wide age-range data into five-year age groups. We began by modeling all of the narrow age-range data (five-year-or-less age range). We use the following notation:

- observations are denoted o and indexed by h.
- studies are denoted st and indexed by i.

We fit a logistic regression using the *lmer* function in *R*. We fit a universal age pattern using an age spline and study-specific random effects a as follows, with p_h equal to the prevalence of visual impairment underlying age-specific observation h:

$$\begin{aligned} y_h &\sim \text{Binom}(n_h, p_h) \\ p_h &= \text{logit}^{-1}\left(a^{st}_{i[h]} + X_h\beta\right). \end{aligned}$$

The β covariates represent a linear spline for age with knots at 40 and 70 years old. Then, for each wide age-range datapoint we created appropriate sets of 5-year age-ranges. With each corresponding set of 5-year age-range data we performed a grid-search to find an appropriate study-offet that allowed the population weighted 5-year age-range data estimate to be the same as its corresponding wide age-range datapoint. Once this had been performed for all wide age-range datapoints, the 5-year age-range estimates were substituted for their wide age-range counterparts.

Text E. Methods for calculating prevalence of visual impairment by country, year, age, and sex

We fit two hierarchical mixed-model logistic regressions using the *R* function *lmer* to separately estimate the prevalence of blindness and MSVI in each country-age group[2,3]. For observation o indexed by h, we define y_h to be the number of subjects with visual impairment out of a total sample size n_h. Our model includes a 5-level hierarchy, denoted a:

- 216 (blind model) or 192 (MSVI model) studies are denoted st and indexed by i.
- 184 countries are denoted c and indexed by j.
- 21 subregions are denoted sb and indexed by k.
- 4 regions are denoted r and indexed by l.
- Global-level effects are denoted g.

Our regression is as follows, with p_h equal to the prevalence of visual impairment underlying observation h:

$$\begin{aligned} y_h &\sim \text{Binom}(n_h, p_h) \\ p_h &= \text{logit}^{-1}\left(a^g + a^r_{l[h]} + a^{sb}_{k[h]} + a^c_{j[h]} + a^{st}_{i[h]} + b^r_{l[h]} sex_h + c^r_{l[h]} year_{i[h]} + X_h\beta\right). \end{aligned}$$

Where b^r_l and c^r_l are region-specific sex and linear year random slopes, respectively.

Study-specific random effects allow for additional variability in studies not explained by sampling uncertainty. The variance of the study-specific random effects depends on whether the study is national or subnational, so:

$$\sigma_{st,i} = \begin{cases} \sigma^{\text{nat'l}} & \text{if study } i \text{ is nationally representative} \\ \sigma^{\text{subnat'l}} & \text{otherwise.} \end{cases}$$

This allows the model estimates to track more closely to national studies than subnational/community studies.

Covariates represent characteristics of the data at the observation, study, and country levels. The observation-specific characteristics are age category, denoted by a three-piece linear spline z^{0+}, z^{1+}, z^{2+} (with spline knots at age 40 and age 70), and an indicator variable for when presenting (vs. best-corrected) vision was measured (*pres*). Because the ratio of presenting and best-corrected vision impairment was significantly larger in South Asia, we fit a separate presenting offset for South Asia (*presSA*). The blind model study-level covariates are an offset for studies that are rural-only, and an offset for studies that are urban-only. There were no study-level covariates for the MSVI model: rural-only and urban-only offsets were not significant and had little effect on the model outcome, and were therefore excluded to maintain a parsimonious model. Country-level covariates were selected using cross-validation, as described in the main text. The country-level covariates for both models are mean years of education and measure of health access.

All together, the covariates for the blind model are:

$$X_h\beta = \beta_0 z_h^{0+} + \beta_1 z_h^{1+} + \beta_2 z_h^{2+} + \beta_3 \text{presSA}_h + \beta_4 \text{pres}_h + \beta_5 \text{rural}_{i[h]} + \beta_6 \text{urban}_{i[h]} + \beta_7 \text{health\_access}_{j[h]} + \beta_8 \text{educ}_{j[h]}$$

The covariates for the MSVI model are:

$$X_h\beta = \beta_0 z_h^{0+} + \beta_1 z_h^{1+} + \beta_2 z_h^{2+} + \beta_3 \text{presSA}_h + \beta_4 \text{pres}_h + \beta_7 \text{health\_access}_{j[h]} + \beta_8 \text{educ}_{j[h]}$$

To predict the prevalence of blindness and MSVI for each country-year-age unit, we use global, regional, subregional and country random effects, and regional sex and year random slopes. Study effects (a_i^{st}) are excluded when making predictions, and the urban and rural fixed effects (rural_i and urban_i) are set to zero. To predict presenting vision impairment, we set the *pres* indicator variable to one and the *presSA* indicator variable to one for the South Asian region and zero for all other regions. We also predict best-corrected vision impairment, setting both presenting indicator variables to zero (results not shown). Thus, we use the following equation to make predictions for each country-year-age unit:

$$\begin{aligned} p_h =& \text{logit}^{-1}(a^g + a^r_{l[j]} + a^{sb}_{k[j]} + a^c_j + b^r_{l[j]} sex + c^r_{l[j]} year \\ & + \beta_0 z^{0+} + \beta_1 z^{1+} + \beta_2 z^{2+} + \beta_3 \text{presSA} + \beta_4 \text{pres} + \beta_7 \text{health\_access}_j + \beta_8 \text{educ}_j) \end{aligned}$$

We predict the central estimates of blindness and MSVI prevalence by fitting the model to the full study dataset. We generated uncertainty using a bootstrap procedure. For each model, we drew *st* studies with replacement, creating 500 bootstrap samples comprising *st* studies. We then fitted each model 500 times, once for each bootstrap dataset, and extracted the model coefficients. For countries with no data in the bootstrap sample, and thus without an estimate of the country-specific random effect, we randomly drew a country-specific effect by sampling from a Normal distribution with mean zero and standard deviation calculated from the fitted country random effects for that model. We then generated 500 draws comprising predictions for each country/year/sex combination as described above; these draws represent the vision impairment envelope's uncertainty distribution. In a few bootstrap interations among age groups >80 years, our estimates of the prevalence of blindness and MSVI sum to more than 100%. For each draw we normalize blind and MSVI estimates to sum to 100%. For each bootstrap sample, another draw is saved with the presenting variables set to zero; the difference between these two draws consititutes vision impairment attributable to refractive error (not presented in this publication).

Finally, we predict the prevalence of severe, moderate, and mild visual impairment using the predicted prevalences of blindness and MSVI, reversing the procedure described in Text C. Using the all survey data which reported multiple definitions of visual impairment described in Text C, we fit logistic regressions to predict the proportion of MSVI that is moderate visual impairment, and the proportion of mild and no visual impairment that is mild visual impairment. We fit the following equations (see Figures E, F, and G):

$$\text{logit}\left(\frac{\text{Prev(moderate)}}{\text{Prev(MSVI)}}\right) = \beta_0 + \beta_1 \text{logit(Prev(MSVI))} + \beta_2 \text{logit(Prev(blind))}$$

$$\text{logit}\left(\frac{\text{Prev(mild)}}{1 - \text{Prev(blind)} - \text{Prev(MSVI)}}\right) = \beta_0 + \beta_1 \text{logit(Prev(MSVI))} + \beta_2 \text{logit(Prev(blind))}$$

For the central estimate and for each bootstrap draw generated as described above, we predict the prevalence of severe, moderate, and mild visual impairment (*e.g.*, 501 values per age-sex-country-year unit). In order to propagate uncertainty, for the bootstrap draws, we predict each of these levels of vision impairment by sampling from the regression coefficients and error terms.

We present uncertainty intervals in summary estimates as the 2.5-97.5 percentiles of the distribution of draws, and the central estimate as the predictions from the model fitted with all study data. We also calculate trends in visual impairment and its uncertainty by creating (for each draw and for the central estimate) age-standardized total vision impairment estimates for all-ages and for ages 50 years and greater, for 1990 and 2010 and for all countries, regions, and the world. We then calculate the difference between the 1990 and 2010 prevalences, by draw and for the central estimate. We present the 2.5th percentile and 97.5th percentile of the difference from the draws as the upper and lower confidence intervals, respectively, for the time trend.

References

1. R. Bourne, H. Price, and H. Taylor. The global burden of disease project: Rationale and methodology of the systematic review by the vision loss group. *Ophthalmic Epidemiology*, X(X):XX, 2012.

2. R Core Team. *R: A Language and Environment for Statistical Computing*. R Foundation for Statistical Computing, Vienna, Austria, 2012. URL `http://www.R-project.org`. ISBN 3-900051-07-0.

3. Douglas Bates, Martin Maechler, and Ben Bolker. *lme4: Linear mixed-effects models using S4 classes*, 2011. URL `http://CRAN.R-project.org/package=lme4`. R package version 0.999375-42.

Table A. **Countries and territories in analysis regions.**

Subregion	Countries
**High-income region**	
Asia-Pacific, high income	Brunei Darussalam, Japan, Republic of Korea, Singapore
Australasia	Australia, New Zealand
North America, high income	Canada, United States of America
Western Europe	Andorra, Austria, Belgium, Cyprus, Denmark, Finland, France, Germany, Greece, Iceland, Ireland, Israel, Italy, Luxembourg, Malta, Netherlands, Norway, Portugal, Spain, Sweden, Switzerland, United Kingdom
**South Asia region**	
South Asia	Afghanistan, Bangladesh, Bhutan, India, Nepal, Pakistan
**Sub-Saharan Africa region**	
Central Africa	Angola, Central African Republic, Congo, Democratic Republic of the Congo, Equatorial Guinea, Gabon
East Africa	Burundi, Comoros, Djibouti, Eritrea, Ethiopia, Kenya, Madagascar, Malawi, Mauritius, Mozambique, Rwanda, Seychelles, Somalia, Sudan, Uganda, United Republic of Tanzania, Zambia
Southern Africa	Botswana, Lesotho, Namibia, South Africa, Swaziland, Zimbabwe
West Africa	Benin, Burkina Faso, Cameroon, Cape Verde, Chad, Côte d'Ivoire, Gambia, Ghana, Guinea, Guinea-Bissau, Liberia, Mali, Mauritania, Niger, Nigeria, Senegal, Sierra Leone, São Tomé and Príncipe, Togo
**Other regions**	
Central Asia	Armenia, Azerbaijan, Georgia, Kazakhstan, Kyrgyzstan, Mongolia, Tajikistan, Turkmenistan, Uzbekistan
East Asia	China, Hong Kong SAR (China), Macau SAR (China), Democratic People's Republic of Korea, Taiwan
Southeast Asia	Cambodia, Indonesia, Lao People's Democratic Republic, Malaysia, Maldives, Myanmar, Philippines, Sri Lanka, Thailand, Timor-Leste, Viet Nam
Oceania	Fiji, Kiribati, Marshall Islands, Micronesia (Federated States of), Papua New Guinea, Samoa, Solomon Islands, Tonga, Vanuatu
Central Europe	Albania, Bosnia and Herzegovina, Bulgaria, Croatia, Czech Republic, Hungary, Montenegro, Poland, Romania, Serbia, Slovakia, Slovenia, Macedonia (Former Yugoslav Republic of)
Eastern Europe	Belarus, Estonia, Latvia, Lithuania, Moldova, Russian Federation, Ukraine
Andean Latin America	Bolivia, Ecuador, Peru
Central Latin America	Colombia, Costa Rica, El Salvador, Guatemala, Honduras, Mexico, Nicaragua, Panama, Venezuela (Bolivarian Republic of)
Southern Latin America	Argentina, Chile, Uruguay
Tropical Latin America	Brazil, Paraguay
Caribbean	Antigua and Barbuda, Bahamas, Barbados, Belize, Cuba, Dominica, Dominican Republic, Grenada, Guyana, Haiti, Jamaica, Puerto Rico, Saint Lucia, Saint Vincent and the Grenadines, Suriname, Trinidad and Tobago
North Africa and Middle East	Algeria, Bahrain, Egypt, Iran (Islamic Republic of), Iraq, Jordan, Kuwait, Lebanon, Libyan Arab Jamahiriya, Morocco, Occupied Palestinian Territory, Oman, Qatar, Saudi Arabia, Syrian Arab Republic, Tunisia, Turkey, United Arab Emirates, Yemen

Table B. Characteristics of data sources used in the analysis.

GBD Region	Country	Coverage	Years (NS = not specified)	Age range	Total examined	Urban / rural	Rapid	Presenting or best-corrected	Core visual acuity levels used	Reference
Andean Latin America	Ecuador	national	2009	50-99	4012	both	Yes	Both	blind, low vision	Felipe Chiriboga, RAAB in Ecuador (2009) unpublished
Andean Latin America	Peru	local	2002	50-99	4782	rural	Yes	Both	blind, low vision	Pongo Aguila, L., R. Carrion, et al. (2005). Cataract blindness in people 50 years old or older in a semirural area of northern Peru. [Spanish]. Revista Panamericana de Salud Publica/Pan American Journal of Public Health 17(5-6): 387-393.
Andean Latin America	Peru	national	2011	50-99	4852	both	Yes	Both	blind, low vision	PERU RAPID DATA
Asia-Pacific, high-income	Japan	local	1997-2000	40-79	2263	urban	No	Best-corrected	blind, low vision	Iwano, M., H. Nomura, et al. (2004). Visual Acuity in a Community-dwelling Japanese Population and Factors Associated with Visual Impairment. Japanese Journal of Ophthalmology 48(1): 37-43.
Asia-Pacific, high-income	Japan	local	2000-2001	40-99	2977	urban	No	Best-corrected	blind, low vision	Iwase, A., M. Araie, et al. (2006). Prevalence and causes of low vision and blindness in a Japanese adult population: the Tajimi Study. Ophthalmology 113(8): 1354-62.
Asia-Pacific, high-income	Japan	local	2005-2006	40-99	3762	rural	No	Presenting	blind, low vision	Nakamura Y, Tomidokoro A, Sawaguchi S, Sakai H, Iwase A, Araie M. Prevalence and causes of low vision and blindness in a rural Southwest Island of Japan: the Kumejima study. Ophthalmology. 2010 Dec;117(12):2315-21
Asia-Pacific, high-income	Singapore	local	1997-1998	40-79	1152	urban	No	Best-corrected	blind, low vision	Saw, S. M., P. J. Foster, et al. (2004). Causes of blindness, low vision, and questionnaire-assessed poor visual function in Singaporean Chinese adults: The Tanjong Pagar Survey. Ophthalmology 111(6): 1161-8.
Asia-Pacific, high-income	Singapore	subnational	NS	3-6	3009	urban	No	Presenting	low vision	Dirani M, Zhou B, Hornbeak D, Chang BC, Gazzard G, Chia A, Ling Y, Selvaraj P, Young TL, Varma R, Wong TY, Saw SM. Prevalence and causes of decreased visual acuity in Singaporean Chinese preschoolers. Br J Ophthalmol. 2010 Dec;94(12):1561-5.
Australasia	Australia	local	1992	70-99	1446	urban	No	Best-corrected	blind, low vision	Casson R, Giles L, Newland HS. Prevalence of blindness and visual impairment in an elderly urban population. Aust N Z J Ophthalmol. 1996 Aug;24(3):239-43.
Australasia	Australia	local	1992-1994	49-99	3650	urban	No	Both	blind, low vision	Blue Mountains Sydney Microdata
Australasia	Australia	local	1992-1996	40-99	4386	urban	No	Both	blind, low vision	Weih LM, VanNewkirk MR, McCarty CA, Taylor HR. Age-specific causes of bilateral visual impairment. Archives of Ophthalmology 2000; 118(2): 264-9.
Australasia	Australia	local	1997-1999	50-99	2614	urban	No	Both	low vision	Foran S, Rose K, Wang JJ, Mitchell P. Correctable visual impairment in an older population: The Blue Mountains Eye Study. American Journal of Ophthalmology 2002; 134(5): 712-9.
Australasia	Australia	local	2003-2005	5-8	1738	urban	No	Both	blind	Robaei D, Rose K, Ojaimi E, et al. Visual acuity and the causes of visual loss in a population-based sample of 6-year-old Australian children. 2005 JOphthalmology; 112(7): 1275-82
Australasia	Australia	local	2004-2005	12	2353	urban	No	Both	blind, low vision	Robaei D, Huynh SC, Kifley A, Mitchell P. Correctable and Non-Correctable Visual Impairment in a Population-Based Sample of 12-Year-Old Australian Children. 2006; 142(1): 112-8.e1.
Australasia	Australia	subnational	2007-2009	4-6	1188	urban	No	Presenting	low vision	Pai AS, Wang JJ, Samarawickrama C, Burlutsky G, Rose KA, Varma R, Wong TY, Mitchell P. Prevalence and risk factors for visual impairment in preschool children the sydney paediatric eye disease study. Ophthalmology. 2011 Aug;118(8):1495-500.
Caribbean	Barbados	national	1988-1992	40-99	4068	both	No	Best-corrected	blind, low vision	Hyman, L., S. Y. Wu, et al. (2001). Prevalence and causes of visual impairment in The Barbados Eye Study.[see comment]. Ophthalmology 108(10): 1751-6
Caribbean	Cuba	local	2005	50-99	2716	urban	Yes	Both	blind, low vision	Juan R. Hernández Silva, Marcelino Río Torres y Carmen Ma. Padilla González Resultados del RACSS en Ciudad de La Habana, Cuba, 2005 Rev Cubana Oftalmol 2006;19(1)
Caribbean	Dominican Republic	national	2008	50-99	3873	both	Yes	Both	blind, low vision	Diaz, Evelyn Garcia (2008) RAAB Dominican Republic, Unpublished
Central Africa	Congo	national	1988	0-99	6185	both	No	Best-corrected	blind, low vision	Annon Weekly Epidemiological Record, 1990:65:249-256 (N°33)
Central Asia	Mongolia	subnational	1991-1992	40-99	4345	both	No	Both	blind	Baasanhu J, Johnson G J, Burendei G, Minassian D C : Prevalence and causes of blindness and visual impairment in Mongolia: a survey of populations aged 40 years and older. 1994. Bulletin of the World Health Organization. 72(5), 771-776
Central Asia	Mongolia	subnational	1995	40-87	942	both	No	Presenting	blind	Foster P J, Baasanhu J, Alsbirk P H, Munkhbayar D, Uranchimeg D, Johnson G J, : Glaucoma in Mongolia. A Population-Based Survey in Hovsgol Provence, Northern Mongolia. 1996. Arch Opthalmol. 114, 1235-1241
Central Asia	Turkmenistan	national	2000-2001	50-99	6011	both	Yes	Presenting	blind, low vision	Amansakhatov S, Volokhovskaya Z P, Afanasyeva A N, Limburg H: Cataract blindness in Turkmenistan: results of a national survey. 2002. British Journal of Ophthalmology. 86, 1207-1210

GBD Region	Country	Coverage	Years (NS = not specified)	Age range	Total examined	Urban / rural	Rapid	Presenting or best-corrected	Core visual acuity levels used	Reference
Central Europe	Bulgaria	local	1993	50-99	6173	both	No	Presenting	blind, low vision	Vassileva, P., S. C. Gieser, et al. (1996). Blindness and visual impairment in western Bulgaria. Ophthalmic Epidemiology 3, 143-9.
Central Latin	El Salvador	national	2011	50-99	3399	both	Yes	Both	blind, low vision	EL SALVADOR RAPID DATA
Central Latin America	Guatemala	subnational	2004	50-99	4806	both	Yes	Both	blind, low vision	Beltranena F, Casasola K, Silva JC, Limburg H. Cataract blindness in 4 regions in Guatemala:results of a population-based survey.Ophthalmology. 2007;114(8):1558–63.
Central Latin America	Mexico	subnational	2005-2006	50-99	3764	both	Yes	Both	blind, low vision	Pedro Gomez cited in Limburg et al. Cataract in Latin America Rev Panam Salud Publica/Pan Am J Public Health 25(5), 2009
Central Latin America	Mexico	subnational	2010	50-99	2864	both	Yes	Both	blind, low vision	Sarah Polack RAAB in Chiapas, Mexico (2010) unpublished
Central Latin America	Venezuela (Bolivarian Republic of)	national	2005	50-99		both	Yes	Presenting	blind, low vision	Sisi F. Results of rapid assessment of cataract surgical services, national survey. 2004. Fundacion Venezolana para la prevencion de la ceguera.unpublished report
East Africa	Eritrea	national	2008	50-99	3163	both	Yes	Both	blind, low vision	Andreas Mueller, RAAB in Eritrea (2008) unpublished
East Africa	Ethiopia	subnational	NS	7-99	11441	both	No	Presenting	blind, low vision	Cerulli, L., C. Cedrone, et al. (1984). Assessment of visual status of the population in seven regions of Ethiopia. Revue Internationale du Trachome et de Pathologie Oculaire Tropicale et Subtropicale et de Sante Publique(2-4): 127-42.
East Africa	Ethiopia	local	1994-1995	0-99	7423	both	No	Presenting	blind, low vision	Zerihun 1997 Blindness and low vision in Jimma zone, Ethiopia: results of a population based survey Ophthalmic Epidemiology p19-25
East Africa	Ethiopia	local	NS	40-99	2639	rural	No	Presenting	blind, low vision	Melese 2003 Low vision and blindness in adults in Gurage zone, Central Ethiopia Br J Ophth p677-680
East Africa	Ethiopia	national	2005-2006	0-99		both	No	Presenting	blind, low vision	National survey on blindness, low vision and trachoma in Ethiopia.Federal MOH of Ethiopia, The Carter Center, CBM, ITI, ORBIS Int.Ethiopia and LfW, Opthalmol Society of Ethiopia, Ethiopian Public Health Association. Addis Ababa, Ethiopia 2006.
East Africa	Ethiopia	subnational	2009	0-16	58480	both	No	Presenting	blind	Demissie BS, Solomon AW. Magnitude and causes of childhood blindness and severe visual impairment in Sekoru District, Southwest Ethiopia: a survey using the key informant method. Trans R Soc Trop Med Hyg. 2011 Sep;105(9):507-11.
East Africa	Kenya	local	NS	0-99		rural	No	Presenting	blind, low vision	Whitfield 1990 Blindness and eye disease in Kenya;ocular status survey results from the Kenya Rural Blindness Prevention Project Br J Ophth p333-344
East Africa	Kenya	local	2002-2003	0-89		urban	No	Presenting	blind, low vision	Ndegwa 2006 Prevalence of visual impairment and blindness in a Nairobi urban population Ophthalmology p69-72
East Africa	Kenya	local	2005	50-99	3503	both	Yes	Presenting	blind, low vision	Mathenge 2007 Rapid assessment of avoidable blindness in Nakuru district Kenya (Kenya -Nukuru RAAB) Ophthalmology p 599-605
East Africa	Kenya	local	2007	50-99	3376	rural	Yes	Presenting	blind, low vision	Karimurio, Sheila, Gichangi, Adala and Huguet 2008 Rapid Assessment of Cataract Surgical Services in Embu District Kenya, East African Journal of Ophthalmology 13 p 19-25
East Africa	Malawi	local	1983	6-99	1664	rural	No	Best-corrected	blind, low vision	Chirambo 1986 Blindess and visual impairment in southern Malawi Bulletin of the WHO p567-577
East Africa	Malawi	local	2009-2010	50-99	3583	rural	Yes	Presenting	blind, low vision	Kalua K, Lindfield R, Mtupanyama M, Mtumodzi D, Msiska V. Findings from a rapid assessment of avoidable blindness (RAAB) in Southern Malawi. PLoS One. 2011 Apr 25;6(4):e19226.
East Africa	Rwanda	subnational	2006	50-99	2206	both	Yes	Presenting	blind, low vision	Mathenge 2007 Rapid assessment of avoidable blindness in Western Rwanda; Blindness in a postconflict setting PLoS Medicine p1187-1193
East Africa	Sudan	local	2005	5-99	2499	rural	No	Presenting	blind, low vision	Ngondi 2006 Prevalence and causes of blindness and low vision in Southern Sudan PLoS Medicine p2416-2426
East Africa	Uganda	local	NS	13-99	4076	rural	No	Presenting	blind, low vision	Mbulaiteye 2002 Evaluation of E optotypes as a screening test and the prevalence and causes of visual loss in a rural population of SW Uganda. Ophthalmic Epidemiology p251-262
East Africa	Uganda	local	2007	50-99		both	Yes	Presenting	blind, low vision	Uganda 2007 RACCS unpublished report, no authors, Masaka
East Africa	United Republic of Tanzania	local	1986	7-99	1827	rural	No	Best-corrected	blind, low vision	Rapoza 1991 Prevalence and causes of vision loss in central Tanzania International Ophthalmology p 123-9
East Africa	United Republic of Tanzania	subnational	2007	50-99		both	Yes	Presenting	blind, low vision	name(?) Rapid Assessment of avoidable blindness in Zanzibar. Submitted for MSc Community Eye Health at the London School of Hygiene and Tropical Medicine. 2007

GBD Region	Country	Coverage	Years (NS = not specified)	Age range	Total examined	Urban / rural	Rapid	Presenting or best-corrected	Core visual acuity levels used	Reference
East Africa	United Republic of Tanzania	local	2007	50-99		both	Yes	Presenting	blind, low vision	Tanzania 2007 - RACSS unpublished report, Kyela District
East Africa	United Republic of Tanzania	local	2007	50-99	3436	rural	Yes	Best-corrected	blind, low vision	Habiyakire, Kabona, Courtright, Lewallen (2010) Rapid Assessment of Avoidable Blindness and Cataract Surgical Services in Kilamanjaro Region, TanzaniaOphth Epi. 17(2)90-94
East Asia	China	national	1987	0-99	1579316	both	No	Best-corrected	blind, low vision	Zhang, et al.national epidemiological survey of blindness and low vision in ChinaChinese medical journal 1992;7:603-608
East Asia	China	local	1996	50-99	5052	rural	No	Presenting	blind, low vision	Zhao J et al. prevalence of blindness and cataract surgery in Shunyi Country, ChinaAJO 1998
East Asia	China	local	1997	50-99	5342	rural	No	Both	blind, low vision	S Li, J Xu, M He, et al.A Survey of Blindness and Cataract Surgery in Doumen County, China. Ophthalmology 1999;106:1602–1615
East Asia	China	local	1998	5-15	5882	urban	No	Both	blind, low vision	Gilbert CE et al. Prevalence and causes of functional low vision in school-age children: Results from standardized population surverys in Asia, Africa, and Latin America Investigative Ophthalmology & Visual Science, March 2008, Vol. 49, No. 3
East Asia	China	subnational	1999-2000	0-99	12644	rural	No	Presenting	blind	S Dunzhu, FS Wang, P Courtright et al. (2003)Blindness and eye diseases in Tibet: findings from a randomised, population based survey Br J Ophthalmol 2003;87:1443–1459
East Asia	China	subnational	2001	35-99	4438	urban	No	Best-corrected	blind, low vision	Bejing Eye Study microdata
East Asia	China	local	2003	60-99	3040	both	No	Best-corrected	blind, low vision	Li L, Guan H, kun P, Zhou J, Gu H. Prevalence and causes of visual impairment among the elderly in Nantong, China. Eye, 2008; 22:1069-75.
East Asia	China	local	2002-2003	5-15	4359	urban	No	Both	blind, low vision	Gilbert CE et al. Prevalence and causes of functional low vision in school-age children: Results from standardized population surverys in Asia, Africa, and Latin America Investigative Ophthalmology & Visual Science, March 2008, Vol. 49, No. 3
East Asia	China	local	2004	3-6	28738	both	No	Presenting	blind, low vision	Lu Q, Zheng Y, Sun B, Cui T, Congdon N, Hu A, Chen J, Shi J. A population-based study of visual impairment among pre-school children in Beijing: the Beijing study of visual impairment in children. Am J Ophthalmol. 2009 Jun;147(6):1075-81
East Asia	China	local	2005	12-18	2454	rural	No	Both	blind	He, M., W. Huang, et al. (2007). Refractive Error and Visual Impairment in School Children in Rural Southern China. Ophthalmology 114(2): 374-382.
East Asia	China	subnational	2007	11-17	1892	rural	No	Both	blind	N Congdon, Y Wang, Y Song, et al. (2008)Visual Disability, Visual Function, and Myopia among Rural Chinese Secondary School Children: The Xichang Pediatric Refractive Error Study (X-PRES)—Report 1.Invest Ophthalmol Vis Sci. 2008;49:2888–2894
East Asia	China	subnational	2006-2007	50-99	45747	rural	No	Both	blind, low vision	Zhao, J., L. B. Ellwein, et al. (2010). Prevalence of Vision Impairment in Older Adults in Rural China:: The China Nine-Province Survey. Ophthalmology 117(3): 409-416.
East Asia	China	local	2007	40-99	4956	rural	No	Both	blind	Song W, Sun X, Shao Z, Zhou X, Kang Y, Sui H, Yuan H. 2010. Prevalence and causes of visual impairment in a rural North-east China adult population: a population-based survey in Bin County, Harbin. Acta Ophthalmol. 2010 Sep;88(6):669-86
East Asia	China	local	2007	50-99	3839	urban	Yes	Both	blind, low vision	Xiao B, Kuper H, Guan C, Bailey K, Limburg H. Rapid assessment of avoidable blindness in three counties, Jiangxi Province, China. Br J Ophthalmol 2010;94 1437-1504
East Asia	China	local	2007	50-99	2861	urban	Yes	Both	blind, low vision	Xiao B, Kuper H, Guan C, Bailey K, Limburg H. Rapid assessment of avoidable blindness in three counties, Jiangxi Province, China. Br J Ophthalmol 2010;94 1437-1450
East Asia	China	local	2007	50-99		urban	Yes	Both	blind, low vision	Xiao B, Kuper H, Guan C, Bailey K, Limburg H. Rapid assessment of avoidable blindness in three counties, Jiangxi Province, China. Br J Ophthalmol 2010;94 1437-1442
East Asia	China	local	2006-2007	50-96	5057	rural	No	Presenting	blind, low vision	Li Z, Cui H, Liu P, Zhang L, Yang H, Zhang L. Prevalence and causes of blindness and visual impairment among the elderly in rural southern Harbin, China. Ophthalmic Epidemiol, 2008; 15:334-8.
East Asia	China	local	2006-2007	30-97	6830	rural	No	Both	blind, low vision	Liang YB, Friedman DS, Wong TY, et al.(2008). Prevalence and Causes of Low Vision and Blindness in a Rural Chinese Adult Population The Handan Eye Study. Ophthalmology 115(11):1965-72.
East Asia	China	subnational	2008	50-99	2842	both	Yes	Both	blind, low vision	Min Wu, RAAB in Luliang County, China (2008) unpublished

GBD Region	Country	Coverage	Years (NS = not specified)	Age range	Total examined	Urban / rural	Rapid	Presenting or best-corrected	Core visual acuity levels used	Reference
East Asia	China	local	2009	0-15	23675	both	No	Presenting	blind	Xiao B, Fan J, Deng Y, Ding Y, Muhit M, Kuper H.Using key informant method to assess the prevalence and causes of childhood blindness in Xiu'shui County, Jiangxi Province, Southeast China. Ophthalmic Epidemiol. 2011 Feb;18(1):30-5.
East Asia	China	local	2010	50-99	939	both	Yes	Both	blind, low vision	CHINA INNER MONGOLIA TUOKETUO RAPID DATA
East Asia	China	local	2010	50-99	980	both	Yes	Both	blind, low vision	CHINA INNER MONGOLIA SHANGDU RAPID DATA
East Asia	China	local	2011	50-99	1723	both	Yes	Both	blind, low vision	CHINA SICHUAN DECHANG RAPID DATA
East Asia	China	local	2011	50-99	2865	both	Yes	Both	blind, low vision	CHINA SICHUAN MIANNING RAPID DATA
East Asia	China, Hong Kong SAR	local	1998	60-99	3441	urban	No	Both	blind, low vision	Michon, Lau, Chan, Ellwein.prevalence of visual impairment,blindness, and cataract surgery in the Hong Kong elderly BJO 2002;86:133-139
East Asia	Taiwan	local	1993-1995	50-99	2038	urban	No	Best-corrected	low vision	J Liu, C Cheng, S Chen, F Lee. Visual impairment in a Taiwanese population: Prevalence, causes, and socioeconomic factors. Ophthalmic epidemiology.2001;8:339-355
East Asia	Taiwan	national	2002	65-99	3160	both	No	Best-corrected	blind, low vision	Tsai, C. Y., L. C. Woung, et al. (2005). The current status of visual disability in the elderly population of Taiwan. Japanese Journal of Ophthalmology 49(2): 166-172.
Eastern Europe	Estonia	local	NS	65-99	910	urban	No	Best-corrected	blind, low vision	Seland, Vingerling,Augood, Bentham, Chakravarthy et al. (2009) Visual Impairment and quality of life in the Older European Population, the EUREYE study Act Ophth 2009
Eastern Europe	Russian Federation	local	2008	50-99		urban	Yes	Presenting	blind, low vision	Branchevskiy S. 2008, RAAB Samara district Russia - unpublished report
North Africa and Middle East	Egypt	subnational	1994	7-15	5839	urban	No	Both	low vision	El-Bayoumy, B. M., A. Saad, et al. (2007). Prevalence of refractive error and low vision among schoolchildren in Cairo. Eastern Mediterranean Health Journal 13(3): 575-9.
North Africa and Middle East	Egypt	local	2009	50-99	2905	rural	Yes	Both	blind, low vision	EGYPT FAYOUM KASR BASEAL RAPID ASSESSMENT
North Africa and Middle East	Egypt	local	2009	50-99	2918	rural	Yes	Both	blind, low vision	EGYPT KAFARELSHEAKHSHABAS RAPID ASSESSMENT
North Africa and Middle East	Egypt	local	2010	50-99	2811	rural	Yes	Both	blind, low vision	EGYPT BANISWEILF MAZORA RAPID ASSESSMENT
North Africa and Middle East	Egypt	local	2010	50-99	2706	rural	Yes	Both	blind, low vision	EGYPT MENYA QALTA RAPID ASSESSMENT
North Africa and Middle East	Egypt	local	2010	50-99	2953	rural	Yes	Both	blind, low vision	EGYPT SOHAG BAGA RAPID ASSESSMENT
North Africa and Middle East	Iran (Islamic Republic of)	local	2002	1-99	4565	urban	No	Both	blind, low vision	Fotouhi, A., H. Hashemi, et al. (2004). The prevalence and causes of visual impairment in Tehran: the Tehran Eye Study. British Journal of Ophthalmology 88(6): 740-5.
North Africa and Middle East	Iran (Islamic Republic of)	local	2005	7-15	5544	both	No	Both	blind	Fotouhi, A., H. Hashemi, et al. (2007). The prevalence of refractive errors among schoolchildren in Dezrul, Iran. British Journal of Ophthalmology 91(3): 287-292.
North Africa and Middle East	Iran (Islamic Republic of)	local	NS	0-15	136000	both	No	Presenting	blind	Razavi H, Kuper H, Rezvan F, Amelie K, Mahboobi-Pur H, Oladi MR, Muhit M, Hashemi H. 2010. Prevalence and causes of severe visual impairment and blindness among children in the lorestan province of iran, using the key informant method. Ophthalm
North Africa and Middle East	Iran (Islamic Republic of)	local	2009	50-99	2819	both	Yes	Presenting	blind, low vision	Rajavi Z, Katibeh M, Ziaei H, Fardesmaeilpour N, Sehat M, Ahmadieh H, Javadi MA. Rapid assessment of avoidable blindness in Iran. Ophthalmology. 2011 Sep;118(9):1812-8
North Africa and Middle East	Iran (Islamic Republic of)	local	2008-2009	40-64	5190	urban	No	Presenting	low vision	Emamian MH, Zeraati H, Majdzadeh R, Shariati M, Hashemi H, Fotouhi A. The gap of visual impairment between economic groups in Shahroud, Iran: a Blinder-Oaxaca decomposition. Am J Epidemiol. 2011 Jun 15;173(12):1463-7.
North Africa and Middle East	Lebanon	national	1995	3-98	10148	both	No	Presenting	blind, low vision	Mansour, A. M., K. Kassak, et al. (1997). National survey of blindness and low vision in Lebanon. British Journal of Ophthalmology 81(10): 905-6.
North Africa and Middle East	Morocco	national	1992	0-99	8878	both	No	Best-corrected	blind, low vision	Annon (1994). Prevention of blindness (PBL) : prevalence and causes of blindness and low vision. Weekly Epidemiological Record 69(18): 129-161.
North Africa and Middle East	Occupied Palestinian Territory	national	2008	50-99	3579	both	Yes	Presenting	blind, low vision	Chiang F, Kuper H, Lindfield R, Keenan T, Seyam N, Magauran D, Khalilia N, Batta H, Abdeen Z, Sargent N. Rapid assessment of avoidable blindness in the Occupied Palestinian Territories. PLoS One. 2010 Jul 29;5(7):e11854.

GBD Region	Country	Coverage	Years (NS = not specified)	Age range	Total examined	Urban / rural	Rapid	Presenting or best-corrected	Core visual acuity levels used	Reference
North Africa and Middle East	Oman	national	1996-1997	0-99	11417	both	No	Presenting	blind	Khandekar, R., A. J. Mohammed, et al. (2004). Coverage of cataract surgery per person and per eye: review of a community-based blindness survey in Oman. Ophthalmic Epidemiology 11(4): 291-9.
North Africa and Middle East	Qatar	national	2009	50-99	2433	both	Yes	Best-corrected	blind, low vision	Al Gamra H, Al Mansouri F, Khandekar R, Elshafei M, Al Qahtani O, Singh R, Hashim SP, Mujahed A, Makled A, Pai A. Prevalence and causes of blindness, low vision and status of cataract in 50 years and older citizen of Qatar-a community based sur
North Africa and Middle East	Tunisia	national	1993	0-99	3547	both	No	Presenting	blind, low vision	Ayed S, Négrel A-D, Nabli M, Jebri A M, Siddhom J. Prevalence et causes de la cecité en Republique Tunisienne. Cahiers Sante 1998;8:275-282.
North Africa and Middle East	Turkey	subnational	1989	0-99	7497	both	No	Presenting	blind, low vision	Negrel, A. D., D. C. Minassian, et al. (1996). Blindness and low vision in southeast Turkey. Ophthalmic Epidemiology 3(3): 127-34.
North Africa and Middle East	Yemen	local	2009	50-99	1789	rural	Yes	Both	blind, low vision	YEMEN AMRAN RAPID ASSESSMENT
North Africa and Middle East	Yemen	local	2009	50-99	1836	rural	Yes	Both	blind, low vision	YEMEN LAHJ RAPID ASSESSMENT
North America, high-income	United States of America	local	1985-1988	40-99	5308	urban	No	Best-corrected	blind, low vision	BALTIMORE EYE STUDY
North America, high-income	United States of America	local	1988-1990	40-89	4916	rural	No	Best-corrected	blind, low vision	Klein R, Klein BEK, Lee KE. Changes in visual acuity in a population. The Beaver Dam Eye Study. Ophthalmology 1996;103:1169–78.
North America, high-income	United States of America	local	1993-1995	65-84	2519	urban	No	Best-corrected	blind, low vision	Salisbury ES
North America, high-income	United States of America	local	1999-2000	40-99	4766	urban	No	Best-corrected	blind, low vision	Proyecto VER
Oceania	Fiji	national	2006-2007	0-15		both	No	Best-corrected	low vision	Cama AT, Sikivou BT, Keeffe JE. Childhood visual impairment in Fiji. Arch Ophthalmol. 2010 May;128(5):608-12.
Oceania	Fiji	subnational	2009	40-99	1381	both	No	Presenting	blind, low vision	Brian G, Pearce MG, Ramke J. Refractive error and presbyopia among adults in Fiji. Ophthalmic Epidemiol. 2011 Apr;18(2):75-82.
Oceania	Papua New Guinea	local	2004-2005	50-99	1174	both	Yes	Presenting	blind, low vision	Garap, J. N., S. Sheeladevi, et al. (2006). Blindness and vision impairment in the elderly of Papua New Guinea. Clinical and Experimental Ophthalmology 34(4): 335-341.
Oceania	Tonga	subnational	1991	20-99	4056	both	No	Best-corrected	blind, low vision	Newland, Woodward, et al. (1994). Epidemiology of blindness and visual impairment in the kingdom of Tonga. British Journal of Ophthalmology 78(5): 344-8.
Oceania	Vanuatu	national	1989	6-99	3520	both	No	Best-corrected	blind	Newland, H. S., M. F. Harris, et al. (1992). Epidemiology of blindness and visual impairment in Vanuatu. Bulletin of the World Health Organization 70(3): 369-72.
South Asia	Bangladesh	national	1999-2000	30-99	11624	both	No	Both	blind, low vision	Bourne Bangladesh National Eye Survey Microdata (1999)
South Asia	Bangladesh	local	2005	50-99	4868	rural	Yes	Presenting	blind, low vision	Wadud, Z., H. Kuper, et al. (2006). Rapid assessment of avoidable blindness and needs assessment of cataract surgical services in Satkhira District, Bangladesh. British Journal of Ophthalmology 90(10): 1225-9.
South Asia	India	national	1986-1989	0-99		both	No	Presenting	blind	Mohan, Survey of blindness in India 1986-1989, National Programme for control of blindness Ministry of Health of Govt of India
South Asia	India	local	1993-1995	3-18	3669	urban	No	Both	blind, low vision	Kalikivayi, V., T. J. Naduvilath, et al. (1997). Visual impairment in school children in southern India.[erratum appears in Indian J Ophthalmol 1997 Sep;45(3):168]. Indian Journal of Ophthalmology 45(2): 129-34.
South Asia	India	local	1995-1997	6-99	9012	rural	No	Both	blind	Thulasiraj, R. D., P. K. Nirmalan, et al. (2003). Blindness and vision impairment in a rural South Indian population: The Aravind Comprehensive Eye Survey. Ophthalmology 110(8): 1491-1498.
South Asia	India	local	1996	0-99	10293	both	No	Presenting	blind, low vision	Dandona, R., L. Dandona, et al. (2002). Moderate visual impairment in India: the Andhra Pradesh Eye Disease Study. British Journal of Ophthalmology 86(4): 373-7.
South Asia	India	local	NS	50-99	4284	rural	No	Both	blind, low vision	Murthy, G. V. S., L. B. Ellwein, et al. (2001). A population-based eye survey of older adults in a rural district of Rajasthan: I. Central vision impairment, blindness, and cataract surgery. Ophthalmology 108(4): 679-685.
South Asia	India	local	1999	50-99	4642	both	No	Both	blind, low vision	Thulasiraj, R. D., R. Rahamathulla, et al. (2002). The Sivaganga eye survey: I. Blindness and cataract surgery. Ophthalmic Epidemiology 9(5): 299-353

GBD Region	Country	Coverage	Years (NS = not specified)	Age range	Total examined	Urban / rural	Rapid	Presenting or best-corrected	Core visual acuity levels used	Reference
South Asia	India	local	2000	50-99	5411	rural	No	Presenting	blind	Nirmalan PK, Thulasiraj RD, Maneksha V, Rahmathullah R, Ramakrishnan R, Padmavathi A and others. 2002. A population based eye survey of older adults in Tirunelveli district of south India: blindness, cataract surgery, and visual outcomes. Briti
South Asia	India	subnational	1999-2001	50-99	63337	both	No	Both	blind, low vision	Murthy, G. V., S. K. Gupta, et al. (2005). Current estimates of blindness in India. British Journal of Ophthalmology 89(3): 257-60.
South Asia	India	local	2000-2001	5-15	4973	both	No	Both	blind, low vision	Gilbert CE et al. Prevalence and causes of functional low vision in school-age children: Results from standardized population surverys in Asia, Africa, and Latin America Investigative Ophthalmology & Visual Science, March 2008, Vol. 49, No. 3
South Asia	India	local	2002	50-99	1505	rural	No	Presenting	blind	Chandrashekhar TS, Bhat HV, Pai RP, Nair SK.: Prevalence of blindness and its causes among those aged 50 years and above in rural Karnataka, South India. 2007. Tropical Doctor. 37, 18-21.
South Asia	India	local	2002	0-15	10605	rural	No	Both	blind, low vision	Nirmalan, P. K., P. Vijayalakshmi, et al. (2003). The Kariapatti pediatric eye evaluation project: Baseline ophthalmic data of children aged 15 years or younger in Southern India. American Journal of Ophthalmology 136(4): 703-709.
South Asia	India	local	2001-2003	40-99	3924	rural	No	Both	blind, low vision	Vijaya, L., R. George, et al. (2006). Prevalence and causes of blindness in the rural population of the Chennai Glaucoma Study. British Journal of Ophthalmology 90(4): 407-10.
South Asia	India	local	NS	0-15	8684	rural	No	Best-corrected	blind	Dorairaj SK, Bandrakalli P, Shetty C, Vathsala R, Misquith D, Ritch R.: Childhood blindness in a rural population of Southern India: Prevalence and etiology. 2008. Ophthalmic Epidemiology. 15(3), 176-182.
South Asia	India	subnational	2007	50-99	40447	both	Yes	Presenting	blind, low vision	Neena, J., J. Rachel, et al. (2008). Rapid Assessment of Avoidable Blindness in India. PLoS ONE [Electronic Resource] 3(8): e2867.
South Asia	India	local	2009	50-99	2004	both	Yes	Best-corrected	blind, low vision	Dhake PV, Dole K, Khandekar R, Deshpande M. Prevalence and causes of avoidable blindness and severe visual impairment in a tribal district of Maharashtra, India. Oman J Ophthalmol. 2011 Sep;4(3):129-34.
South Asia	India	local	2011	50-99	2907	both	Yes	Both	blind, low vision	KOLAR KARNATAKA RAPID ASSESSMENT
South Asia	Nepal	national	1980-1981	0-99	39887	both	No	Best-corrected	blind	Brilliant LB, Pokhrel RP, Grasset NC, Lepkowski JM, Kolstad A, Hawks W, Pararajasegaram R, Brilliant GE, Gilbert S, Shrestha SR, Juo J.: Epidemiology of blindness in Nepal. 1985. Bulletin of the World Health Organisation. 63(2), 375-386.
South Asia	Nepal	subnational	1997	5-15	4803	both	No	Both	low vision	Pokharel GP, Negrel AD, Munoz SR, Ellwein LB. 2000. Refractive error study in children: Results from Mechi Zone, Nepal. American Journal of Ophthalmology 129(4):436-444.
South Asia	Nepal	local	1998	5-15	4802	rural	No	Both	blind, low vision	Gilbert CE et al. Prevalence and causes of functional low vision in school-age children: Results from standardized population surverys in Asia, Africa, and Latin America Investigative Ophthalmology & Visual Science, March 2008, Vol. 49, No. 3
South Asia	Nepal	local	2002	45-99	5002	rural	No	Both	blind	Sapkota, Y. D., G. P. Pokharel, et al. (2006). Prevalence of blindness and cataract surgery in Gandaki Zone, Nepal. British Journal of Ophthalmology 90(4): 411-6.
South Asia	Nepal	local	2006	50-99	4717	rural	Yes	Both	blind, low vision	Sapkota YD, Sunuwar M, Naito T, Akura J, Adhikari HK. The prevalence of blindness and cataract surgery in rautahat district, Nepal. Ophthalmic Epidemiol. 2010 Mar;17(2):82-9.
South Asia	Nepal	local	2006	50-99	5138	rural	Yes	Both	blind, low vision	Sherchan A, Kandel RP, Sharma MK, Sapkota YD, Aghajanian J, Bassett KL. Blindness prevalence and cataract surgical coverage in Lumbini Zone and Chetwan District of Nepal. Br J Ophthalmol. 2010 Feb;94(2):161-6.
South Asia	Nepal	local	NS	40-99	4003	both	No	Both	blind, low vision	Thapa SS, Berg RV, Khanal S, Paudyal I, Pandey P, Maharjan N, Twyana SN, Paudyal G, Gurung R, Ruit S, Rens GH. 2011. Prevalence of visual impairment, cataract surgery and awareness of cataract and glaucoma in Bhaktapur district of Nepal: the Bh
South Asia	Nepal	local	2008	50-99	2914	rural	Yes	Both	blind, low vision	NEPAL SAGARMATA ZONE RAPID DATA
South Asia	Nepal	local	2008	50-99	2895	both	Yes	Both	blind, low vision	NEPAL KOSHI ZONE RAPID DATA
South Asia	Nepal	subnational	2008	50-99	3613	both	Yes	Both	blind, low vision	Reeta Gurung (2008), RAAB in Bagmati and Janakpur zone, Nepal. Unpublished
South Asia	Nepal	local	2008	50-99	2513	rural	Yes	Both	blind, low vision	NEPAL SETI & MAHAKALI ZONES RAPID DATA
South Asia	Nepal	local	2008	50-99	1171	rural	Yes	Both	blind, low vision	NEPAL KARNALI ZONE RAPID DATA

GBD Region	Country	Coverage	Years (NS = not specified)	Age range	Total examined	Urban / rural	Rapid	Presenting or best-corrected	Core visual acuity levels used	Reference
South Asia	Nepal	local	2009	50-99	3041	rural	Yes	Both	blind, low vision	NEPAL MECHI ZONE RAPID DATA
South Asia	Nepal	local	2009	50-99	2993	rural	Yes	Both	blind, low vision	NEPAL BHERI ZONE RAPID DATA
South Asia	Nepal	local	2010	50-99	2990	rural	Yes	Both	blind, low vision	NEPAL DHAULAGIRI ZONE RAPID DATA
South Asia	Nepal	local	2010	50-99	2921	rural	Yes	Both	blind, low vision	NEPAL RAPTI ZONE RAPID DATA
South Asia	Pakistan	local	1998	40-99	1106	rural	No	Presenting	blind, low vision	Ahmad K, Khan MD, Qureshi MB, Munami S, Shah RA, Rasheed H, Jamali B, Baluch A, Khan MA.; Prevalence and causes of blindness and low vision in a rural setting in Pakistan. 2005. Ophthalmic Epidemiology. 12(1), 19-23.
South Asia	Pakistan	local	NS	50-99	1505	both	Yes	Both	blind, low vision	Haider S, Hussain A, Limburg H, 2003. Cataract blindness in Chakwal District, Pakistan: results of a survey. Ophthalmic Epidemiology 10(4):249-58.
South Asia	Pakistan	national	2001-2003	30-99	16507	both	No	Presenting	blind, low vision	Bourne Pakistan national Eye Survey Microdata (2001)
South Asia	Pakistan	local	NS	50-99	1549	rural	Yes	Best-corrected	blind	Anjum KM, Qureshi MB, Khan MA, Jan N, Ali A, Ahmad K, Khan MD.: Cataract blindness and visual outcome of cataract surgery in a tribal area in Pakistan. 2006. British Journal of Ophthalmology. 90, 135-138.
South Asia	Pakistan	local	2003	5-15	5110	urban	No	Presenting	blind, low vision	Shaikh, S. P. and T. M. Aziz (2005). Pattern of eye diseases in children of 5-15 years at Bazzertaline area (South Karachi) Pakistan. Journal of the College of Physicians and Surgeons Pakistan 15(5): 291-294.
Southeast Asia	Cambodia	subnational	1996	0-99	5803	both	No	Presenting	blind, low vision	Rutzen, A. R., N. J. Ellish, et al. (2007). Blindness and eye disease in Cambodia. Ophthalmic Epidemiology 14(6): 360-6.
Southeast Asia	Cambodia	subnational	2002	50-99		both	Yes	Presenting	blind, low vision	Sub-Committee for the Prevention of Blindness, Cambodia. Results of rapid assessment of cataract surgical services. Cambodia: Battabang. 2002.
Southeast Asia	Cambodia	national	2007	50-99		both	Yes	Presenting	blind, low vision	Cambodia 2007 - contact: Dr. Do Seiha RAAB unpublished report without authors
Southeast Asia	Indonesia	local	2001-2002	21-99	989	both	No	Presenting	blind, low vision	Saw, S. M., R. Husain, et al. (2003). Causes of low vision and blindness in rural Indonesia. British Journal of Ophthalmology 87(9): 1075-8.
Southeast Asia	Indonesia	local	2004	50-99		both	Yes	Presenting	blind, low vision	Indonesia 2004 - RACSS unpublished report, not sure of principal investigator, Lombok
Southeast Asia	Malaysia	local	1993-1994	18-99	282	rural	No	Best-corrected	blind, low vision	Zainal, M., L. Masran, et al. (1998). Blindness and visual impairment amongst rural Malays in Kuala Selangor, Selangor. Medical Journal of Malaysia 53(1): 46-50.
Southeast Asia	Malaysia	national	1996-1997	0-99	18027	both	No	Presenting	blind, low vision	Zainal, M., S. M. Ismail, et al. (2002). Prevalence of blindness and low vision in Malaysian population: results from the National Eye Survey 1996. British Journal of Ophthalmology 86(9): 951-6.
Southeast Asia	Malaysia	local	2000	40-99	311	rural	No	Presenting	blind, low vision	Reddy, S. C., L. Rampal, et al. (2004). Prevalence and causes of visual impairment and blindness in a rural population in Sepang district, Selangor. Medical Journal of Malaysia 59(2): 212-7.
Southeast Asia	Malaysia	local	2003	7-15	4629	urban	No	Both	blind, low vision	Goh PP, Abqariyah Y, Pokharel GP, Ellwein LB.: Refractive error and visual impairment in school-age children in Gombak district, Malaysia. 2005. American Academy of Ophthalmology. 112, 678-685.
Southeast Asia	Malaysia	local	2004-2006	40-80	3280	urban	No	Both	blind, low vision	Wong, T. Y., E. W. Chong, et al. (2008). Prevalence and causes of low vision and blindness in an urban malay population: the Singapore Malay Eye Study. Archives of Ophthalmology 126(8): 1091-9.
Southeast Asia	Myanmar	local	2001	50-99		both	Yes	Presenting	blind, low vision	Limburg H, Maung N, Khin Aye Soe, Mynt Than Win. Study report on rapid assessment of cataract surgical services in Hinthada Township, Union of Myanmar. Ministry of Health, Department of Health, Trachoma Control and Prevention of Blindness Progr
Southeast Asia	Myanmar	local	2005	40-99	2076	rural	No	Both	blind, low vision	Casson, R. J., H. S. Newland, et al. (2007). Prevalence and Causes of Visual Impairment in Rural Myanmar. The Meiktila Eye Study. Ophthalmology 114(12): 2302-2308.
Southeast Asia	Philippines	local	2005	50-99	2774	rural	Yes	Presenting	blind, low vision	Eusebio, C., H. Kuper, et al. (2007). Rapid assessment of avoidable blindness in Negros Island and Antique District, Philippines. British Journal of Ophthalmology 91(12): 1588-1592.
Southeast Asia	Philippines	local	2006	50-99	3177	rural	Yes	Presenting	blind, low vision	Eusebio, C., H. Kuper, et al. (2007). Rapid assessment of avoidable blindness in Negros Island and Antique District, Philippines. British Journal of Ophthalmology 91(12): 1588-1592.

GBD Region	Country	Coverage	Years (NS = not specified)	Age range	Total examined	Urban / rural	Rapid	Presenting or best-corrected	Core visual acuity levels used	Reference
Southeast Asia	Sri Lanka	local	2006-2007	40-99	1375	rural	No	Best-corrected	blind, low vision	Edussuriya K, Sennanayake S, Senaratne T, Marshall D, Sullivan T, Selva D, Casson RJ. The prevalence and causes of visual impairment in central Sri Lanka the Kandy Eye study. Ophthalmology. 2009 Jan;116(1):52-6.
Southeast Asia	Thailand	national	1994-1995	0-99		both	No	Presenting	blind, low vision	Prompubesara and Wongwetsawat Third National Survey on blindness and low vision in Thailand 1994 UNPUBLISHED
Southeast Asia	Thailand	local	1997-1998	40-99	2092	urban	No	Best-corrected	blind, low vision	Singalavanija, A., A. Metheetrairut, et al. (2001). Ocular diseases and blindness in elderly Thais. Journal of the Medical Association of Thailand 84(10): 1383-8.
Southeast Asia	Thailand	local	1997-1999	50-99	701	urban	No	Both	blind	Bourne, R. R., P. Sukudom, et al. (2003). Prevalence of glaucoma in Thailand: a population based survey in Rom Klao District, Bangkok. British Journal of Ophthalmology 87(9): 1069-74.
Southeast Asia	Timor-Leste	subnational	2005	40-99	1414	both	Yes	Presenting	blind, low vision	Ramke, J., R. du Toit, et al. (2007). Correction of refractive error and presbyopia in Timor-Leste.[see comment]. British Journal of Ophthalmology 91(7): 860-6.
Southeast Asia	Viet Nam	subnational	2000-2002	50-99	14138	both	Yes	Both	blind, low vision	Limburg (2008): Results of Rapid Assessment for Avoidable blindness (RAAB) in 16 provices of Vietnam
Southeast Asia	Viet Nam	subnational	2007	50-99	28033	both	Yes	Both	blind, low vision	Limburg (2008): Results of Rapid Assessment for Avoidable blindness (RAAB) in 16 provices of Vietnam
Southern Africa	Botswana	national	1998	60-99	372	both	No	Presenting	blind	Clausen T, Romoren TI, Ferriera M, Kristensen P, Ingstad B, Holmboe-Ottensen G. Chronic diseases and health inequalities in older persons in Botswana (southern Africa): A national survey. 2005. The Journal of Nutrition, Health and Aging. 9 (6
Southern Africa	South Africa	local	NS	0-99	6090	rural	No	Presenting	blind, low vision	Cook CD, Knight SE, Crofton-Briggs I.: Prevalence and causes of low vision and blindness in northern Kwazulu. 1993. SAMJ. 83 590-593.
Southern Africa	South Africa	local	1992	40-99	987	rural	No	Presenting	blind	Salmon JF, Mermoud A, Ivey A, Swanevelder SA, Hoffman M.: The prevalenceof primary angle closure glaucoma, and open angle glaucoma in Mamre, Western Cape, South Africa. 1993. Arch Ophthalmol. 111 1263-1269.
Southern Africa	South Africa	local	1998-1999	40-99	1005	rural	No	Presenting	blind	Rotchford AP, Johnson GJ.: Glaucoma in Zulus. A Population-Based Cross-sectional Survey in a Rural District in South Africa. 2002. Arch Ophthalmol. 120. 471-478.
Southern Africa	South Africa	local	2002	5-15	4679	both	No	Both	blind, low vision	Gilbert CE et al.(2008) Prevalence and causes of functional low vision in school-age children: Results from standardized population surverys in Asia, Africa, and Latin America Investigative Ophthalmology & Visual Science, Vol. 49, No. 3
Southern Africa	Zimbabwe	local	NS	60-99	278	both	No	Presenting	blind	Allain TJ, Wilson AO, Gomo ZAR, Mushangi E, Senzanje B, Adamchak DJ, Matenga JA.: Morbidity and disability in elderly Zimbabweans. 1997. Age and Ageing. 26 115-121.
Southern Latin America	Argentina	local	2005	50-99	4302	urban	Yes	Both	blind, low vision	Nano, M. E., H. D. Nano, et al. (2006). Rapid assessment of visual impairment due to cataract and cataract surgical services in urban Argentina. Ophthalmic Epidemiology 13(3): 191-197.
Southern Latin America	Chile	local	1998	5-15	5284	urban	No	Both	blind, low vision	Gilbert CE et al. Prevalence and causes of functional low vision in school-age children: Results from standardized population surverys in Asia, Africa, and Latin America Investigative Ophthalmology & Visual Science, March 2008, Vol. 49, No. 3
Southern Latin America	Chile	local	2006-2007	50-99	2915	both	Yes	Both	blind, low vision	Fernando BarrÃ-, Arcg, Chil. Oftal 2007 Análisis de las barreras,cobertura y resultados postoperatorios de cirugía de catarata determinados mediante encuesta rápida de ceguera evitable en la VIII región, Chile
Southern Latin America	Uruguay	local	2011	50-99	3729	both	Yes	Both	blind, low vision	URUGUAY RAPID DATA
Tropical Latin America	Brazil	local	2002	0-99	801	urban	No	Both	blind, low vision	Araujo Filho, A., S. R. Salomao, et al. (2008). Prevalence of visual impairment, blindness, ocular disorders and cataract surgery outcomes in low-income elderly from a metropolitan region of Sao Paulo--Brazil. Arquivos Brasileiros de Oftalmolog
Tropical Latin America	Brazil	local	2003	50-99	2224	urban	Yes	Both	blind, low vision	Arieta, C. E. L., D. F. de Oliveira, et al. (2009). Cataract remains an important cause of blindness in Campinas, Brazil. Ophthalmic Epidemiology 16(1): 58-63.
Tropical Latin America	Brazil	local	2004	0-99	3678	urban	No	Both	blind, low vision	Salomao, S. R., R. W. Cinoto, et al. (2008). Prevalence and causes of vision impairment and blindness in older adults in Brazil: the sao paulo eye study. Ophthalmic Epidemiology 15(3): 167-75.

GBD Region	Country	Coverage	Years (NS = not specified)	Age range	Total examined	Urban / rural	Rapid	Presenting or best-corrected	Core visual acuity levels used	Reference
Tropical Latin America	Brazil	local	NS	0-99	2485	urban	No	Both	blind, low vision	Schellini SA, Durkin SR, Hoyama E, Hirai F, Cordeiro R, Casson RJ, Selva D, Padovani CR. Prevalence and causes of visual impairment in a Brazilian population: the Botucatu Eye Study. BMC Ophthalmol. 2009 Aug 19;9:8.
Tropical Latin America	Paraguay	national	1999	50-99	2136	both	Yes	Both	blind, low vision	Duerksen R, Limburg H, Carron JE, Foster A. Cataract blindness in Paraguay—results of a national survey. Ophthalmic Epidemiol. 2003; 10(5):349–57.
Tropical Latin America	Paraguay	national	2011	50-99	2862	both	Yes	Both	blind, low vision	PARAGUAY 2011 RAPID DATA
West Africa	Benin	national	1990	0-99	7047	both	No	Best-corrected	blind	Negrel AD, Avognon Z, Minassian DC, Babagbeto M, Oussa G, Bassabi S.: Blindness in Benin. 1995. Med Trop (Mars) 55 (4 Pt 2) 409-414.
West Africa	Cameroon	subnational	1992	6-99	10647	both	No	Presenting	blind, low vision	Wilson, M. R., M. Mansour, et al. (1996). Prevalence and causes of low vision and blindness in the Extreme North Province of Cameroon, West Africa. Ophthalmic Epidemiology 3(1): 23-33.
West Africa	Cameroon	local	NS	40-99	1787	rural	Yes	Presenting	blind, low vision	Oye JE, Kuper H, Dineen B, Befidi-Mengue R, Foster A.: Prevalence ad causes of blindess and visual imapirment in Muyuka: a rural health distric in South West Province, Cameroon. 2006. British Journal of Ophthalmology. 90 (5) 538-542.
West Africa	Cameroon	local	NS	40-99	2215	urban	Yes	Presenting	blind, low vision	Oye JE, Kuper H.: Prevalence and causes of blindness and visual impairment in Limbe urban area, South West Province, Cameroon. 2007. British Journal of Ophthalmology. 91 1435-1439.
West Africa	Cape Verde	subnational	1998	0-99	3374	both	No	Presenting	blind, low vision	Schemann JF, Inocencio F, Monteiro ML, Andrade J, Auzemery A, Guelfi Y. :Blindness and Low Vision in cape Verde Islands: Results of a National Eye Survey. 2006. Ophthalmic Epidemiology. 13(4), 219-226.
West Africa	Gambia	national	1986	0-99	8174	both	No	Presenting	blind, low vision	Faal H, Minassian D, Sowa S, Foster A.: National survey of blindness and low vision in The Gambia. 1989. British Journal of Ophthalmology 73 82-87.
West Africa	Gambia	national	1996	5-99	13046	both	No	Presenting	blind, low vision	Faal H, Minassian DC, dolin PJ, Mohamed AA, Ajewole J, Johnson GJ.: Evaluation of a national eye care programme: re-survey after 10 years. 2000. British Journal of Ophthalmology. 84 948-951.
West Africa	Gambia	subnational	2007	50-99		both	Yes	Presenting	blind, low vision	Gambia 2007
West Africa	Ghana	local	2001	40-99	2298	both	No	Both	blind, low vision	Guzek JP, Anyomi FK, Fiadoyor S, Nyonator F.: Prevalence of blindness in people over 40 years in the Volta region of Ghana. 2005. Ghana Medical Journal. 39 (2) 55-62.
West Africa	Mali	local	1990	0-99	5871	rural	No	Best-corrected	blind	Kortlang C, Koster JCA, Coulibaly S, Dubbeldam RP.: Prevalence of blindness and visual impairment in the region of Segou, Mali. A baseline survey for a primary eye care programme. 1996. Tropical medicine and International Health. 1 (3) 314-319
West Africa	Mali	subnational	2002	50-99		both	Yes	Presenting	blind, low vision	Ceccon JF: Results of rapid assessment of cataract surgical services in Koulikoro, Mali. 2002. Institute of African Tropical Ophthalmology, Bamako Mali.
West Africa	Mauritania	local	2002	50-99		urban	Yes	Presenting	blind, low vision	Ahmedou SE. Results of rapid assessment of cataract surgical services in urban Nouackchott, Mauritania. 2001. Service Ophthalmologie, MOH, Nouackchott, Mauritania.
West Africa	Nigeria	local	1991	0-99	2921	rural	No	Both	blind	Adeoye A.: Survey of blindness in rural communities of south-western Nigeria. 1996. Tropical Medicine and International Health. 1 (5) 672-676.
West Africa	Nigeria	subnational	1992	0-99	1752	both	No	Best-corrected	blind, low vision	Ezepue UF.: Magnitude and causes of blindness and low vision in Anambra State of Nigeria (results of 1992 point prevalence survey). 1997. Public Health. 111 305-309.
West Africa	Nigeria	local	1995	0-99	3332	both	No	Presenting	blind, low vision	Abdu L.: Prevalence and causes of blindness and low vision in Dambatta local government area, Kano State Nigeria. Remainder of reference details not available on abstract.
West Africa	Nigeria	subnational	1999	40-99	1461	rural	Yes	Presenting	blind, low vision	Rabiu, M. (2001). Cataract blindness and barriers to uptake of cataract surgery in a rural community of northern Nigeria. British Medical Journal 85(7): 776.
West Africa	Nigeria	local	NS	50-99	477	rural	Yes	Presenting	blind	Oluleye TS.: Cataract Blindness and Barriers to Cataract Surgical Intervention in Three Rural Communities of Oyo State, Nigeria. 2004 13 (2) 156-160.
West Africa	Nigeria	local	NS	0-99	3204	rural	No	Presenting	blind	Adeoti CO.: Prevalence and causes of blindness in a tropical African population. 2004. WAJM. 23 (3). 249-252.
West Africa	Nigeria	local	2001	50-99	684	both	Yes	Presenting	blind	Patrick-Ferife G, Ashaye AO, Osuntokun OO. : Rapid assessment of cataract blindness among Ughelli clan in an urban/rural district of Delta State, Nigeria. 2005. Annals of African Medicine. 4 (2) 52-57.
West Africa	Nigeria	local	2002	8-92	2201	rural	No	Presenting	blind	Adegbehingbe BO, Majengbasan TO.: Ocular health status of rural dwellers in south-western Nigeria. 2007. Aust J Rural Health. 15 269-272.

GBD Region	Country	Coverage	Years (NS = not specified)	Age range	Total examined	Urban / rural	Rapid	Presenting or best-corrected	Core visual acuity levels used	Reference
West Africa	Nigeria	local	2002	4-24	1144	both	No	Presenting	blind, low vision	Ajaiyeoba AI, Isawumi MA, Adeoye AO, Oluleye TS.: Pattern of eye disease and visual impairment among students in southwestern Nigeria. 2007. Int Ophthalmol. 27 287-292.
West Africa	Nigeria	local	NS	0-99	1248	rural	No	Presenting	blind, low vision	Onakpoya OH, Adeoye AO, Akinsola FB, Adegbehingbe.: Prevalence of blindness and visual impairment in Atakunmosa West Local Government Area of southwestern Nigeria. 2007. Tanzania Health Research Bulletin. 9 (2) 126-131.
West Africa	Nigeria	local	2005	0-99	4848	rural	No	Presenting	blind, low vision	Muhammad N, Mansur RM, Dantani AM, Elhassan E, Isiyaku S. Prevalence and causes of blindness and visual impairment in sokoto state, Nigeria: baseline data for vision 2020: the right to sight eye care programme.. Middle East Afr J Ophthalmol. 20
West Africa	Nigeria	subnational	2006	1-97	3357	rural	No	Presenting	blind	Mpyet C, Ogoshi C, Goyol M. : Prevalence of Trachoma in Yobe State, North-Eastern Nigeria. 2008. Ophthalmic Epidemiology. 15, 303-307.
West Africa	Nigeria	national	2007	40-99	13599	both	No	Both	blind, low vision	Kyari, F., M. V. S. Gudlavalleti, et al. (2009). Prevalence of Blindness and Visual Impairment in Nigeria: The national blindness and visual impairment survey. Investigative Ophthalmology & Visual Science 50(5): 2033
West Africa	Sierra Leone	national	2010-2011	50-99	2976	both	Yes	Both	blind, low vision	SIERRA LEONE RAPID DATA
Western Europe	Denmark	local	1986-1988	60-80	946	urban	No	Both	blind, low vision	Buch, H., T. Vinding, et al. (2001). Prevalence and causes of visual impairment according to World Health Organization and United States criteria in an aged, urban Scandinavian population: the Copenhagen City Eye Study. Ophthalmology 108(12): 2
Western Europe	Finland	national	2000-2001	30-99	6663	both	No	Presenting	blind	Laitinen, A., S. Koskinen, et al. (2005). A nationwide population-based survey on visual acuity, near vision, and self-reported visual function in the adult population in Finland. Ophthalmology 112, 2227-2237.
Western Europe	France	local	NS	65-99	702	urban	No	Best-corrected	blind, low vision	Seland, Vingerling,Augood, Bentham, Chakravarthy et al. (2009) Visual Impairment and quality of life in the Older European Population, the EUREYE study Act Ophth 2009
Western Europe	Greece	local	NS	65-99	586	urban	No	Best-corrected	blind, low vision	Seland, Vingerling,Augood, Bentham, Chakravarthy et al. (2009) Visual Impairment and quality of life in the Older European Population, the EUREYE study Act Ophth 2009
Western Europe	Iceland	local	1996	50-99	1635	urban	No	Best-corrected	blind, low vision	Gunnlaugsdottir E, Arnarsson A, Jonasson F. Prevalence and causes of visual impairment and blindness in Icelanders aged 50 years and older: the Reykjavik Eye Study. Acta Ophthalmol. 2008 Nov;86(7):778-85.
North Africa and Middle East	Occupied Palestinian Territory	local	1982-1983	0-99	9054	both	No	Presenting	blind, low vision	Thomson I M, Chumbley L C. Eye disease in the West Bank and Gaza strip. Br J Ophthalmol 1984;68:598-602
Western Europe	Italy	local	1988	40-87	538	rural	No	Best-corrected	blind, low vision	Cedrone, C., F. Ricci, et al. (2007). Age-specific changes in the prevalence of best-corrected visual impairment in an Italian population. Ophthalmic Epidemiology 14(5): 320-326.
Western Europe	Italy	local	2000	40-99	845	urban	No	Both	blind, low vision	Cedrone, C., C. Nucci, et al. (2005). Prevalence of blindness and low vision in an Italian population: a comparison with other European studies. Eye 20(6): 661-667.
Western Europe	Italy	local	NS	65-99	601	urban	No	Best-corrected	blind, low vision	Seland, Vingerling,Augood, Bentham, Chakravarthy et al. (2009) Visual Impairment and quality of life in the Older European Population, the EUREYE study Act Ophth 2009
Western Europe	Netherlands	local	1990-1993	55-99		urban	No	Best-corrected	blind, low vision	Klaver, C. C., R. C. Wolfs, et al. (1998). Age-specific prevalence and causes of blindness and visual impairment in an older population: the Rotterdam Study. Archives of Ophthalmology 116(5): 653-8.
Western Europe	Netherlands	local	1997-1999	85-99	459	both	No	Presenting	low vision	Gussekloo, J., A. J. de Craen, et al. (2005). Sensory impairment and cognitive functioning in oldest-old subjects: the Leiden 85+ Study. American Journal of Geriatric Psychiatry 13, 781-6.
Western Europe	Norway	local	NS	65-99	737	urban	No	Best-corrected	blind, low vision	Seland, Vingerling,Augood, Bentham, Chakravarthy et al. (2009) Visual Impairment and quality of life in the Older European Population, the EUREYE study Act Ophth 2009
Western Europe	Spain	local	NS	64-97	1144	both	No	Best-corrected	blind, low vision	Esteban, J. J., M. S. Martinez, et al. (2008). Visual impairment and quality of life: gender differences in the elderly in Cuenca, Spain. Quality of Life Research 17, 37-45.
Western Europe	Spain	local	2007	40-79	510	urban	No	Best-corrected	blind, low vision	Anton A et al. Epidemiology of Refractive Errors in an Adult European Population: The Segovia Study (Spain) 2009 Ophth Epi, Vol. 16, No. 4 , Pages 231-237
Western Europe	United Kingdom	national	1980	10-12	12853	both	No	Presenting	low vision	Stewart-Brown, S. and N. Butler (1985). Visual acuity in a national sample of 10 year old children. Journal of Epidemiology & Community Health 39(2): 107-12.

GBD Region	Country	Coverage	Years (NS = not specified)	Age range	Total examined	Urban / rural	Rapid	Presenting or best-corrected	Core visual acuity levels used	Reference
Western Europe	United Kingdom	local	1982-1984	75-99	529	both	No	Best-corrected	blind, low vision	Gibson, J. M., J. R. Lavery, et al. (1986). Blindness and partial sight in an elderly population. British Journal of Ophthalmology 70, 700-5.
Western Europe	United Kingdom	local	NS	65-99	207	urban	No	Presenting	blind, low vision	Wormald, R. P., L. A. Wright, et al. (1992). Visual problems in the elderly population and implications for services.[see comment]. BMJ 304, 1226-9.
Western Europe	United Kingdom	national	1994-1995	65-99	1362	both	No	Presenting	low vision	van der Pols, J. C., C. J. Bates, et al. (2000). Visual acuity measurements in a national sample of British elderly people. British Journal of Ophthalmology 84, 165-70.
Western Europe	United Kingdom	local	NS	65-99	629	urban	No	Best-corrected	blind, low vision	Seland, Vingerling,Augood, Bentham, Chakravarthy et al. (2009) Visual Impairment and quality of life in the Older European Population, the EUREYE study Act Ophth 2009

Table C. Coefficients **for conversion to core levels of vision impairment.**

	Intercept		Coefficient		N	Adjusted R-squared
	Value	Standard error	Value	Standard error		
Conversion to blind (<3/60)						
from <6/60	-1.024	0.032	0.911	0.010	944	0.895
from ≤6/60	-1.213	0.139	0.813	0.030	76	0.905
Conversion to low vision (<6/18 and ≥3/60)						
from <6/18	-0.524	0.014	0.898	0.005	1270	0.965
from <6/12	-1.280	0.057	0.837	0.021	85	0.951

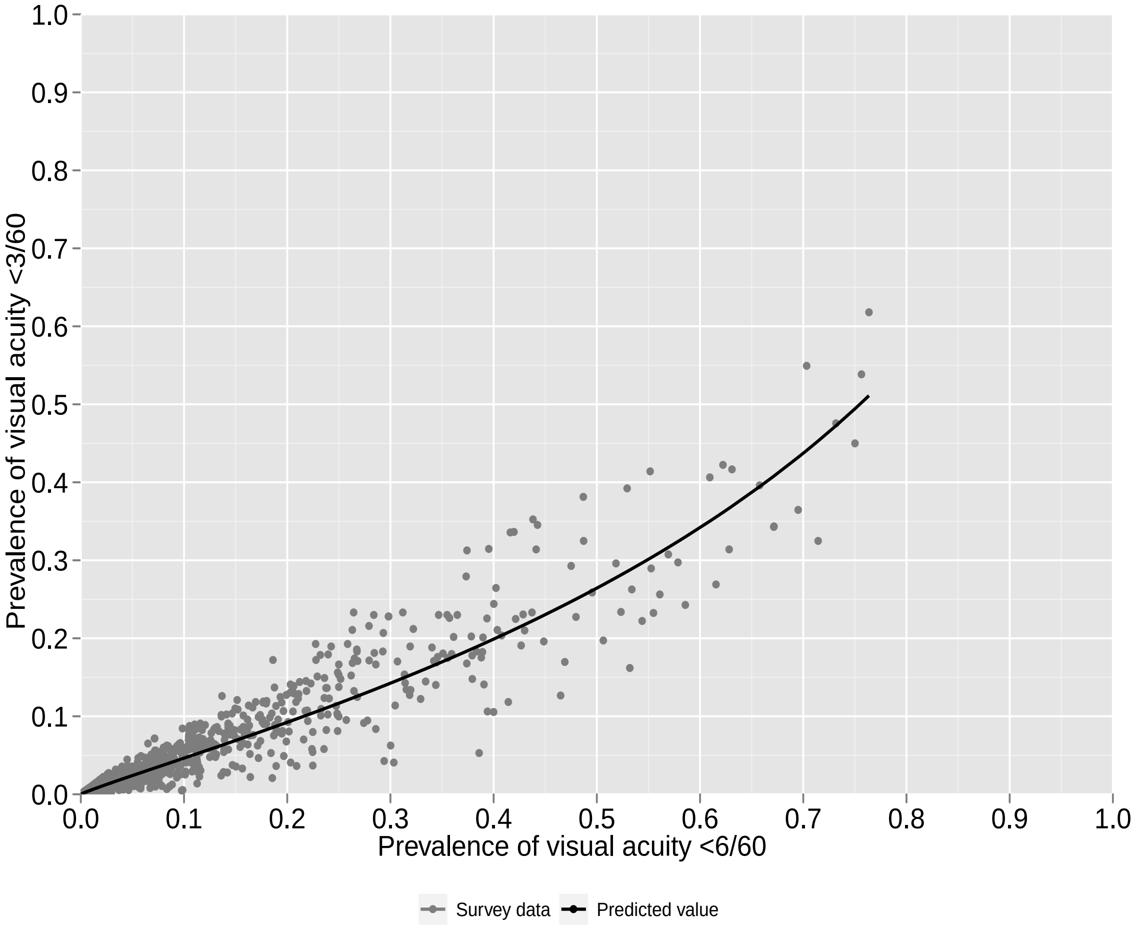

Figure A: Conversion from visual acuity $< 6/60$ to blind ($< 3/60$)

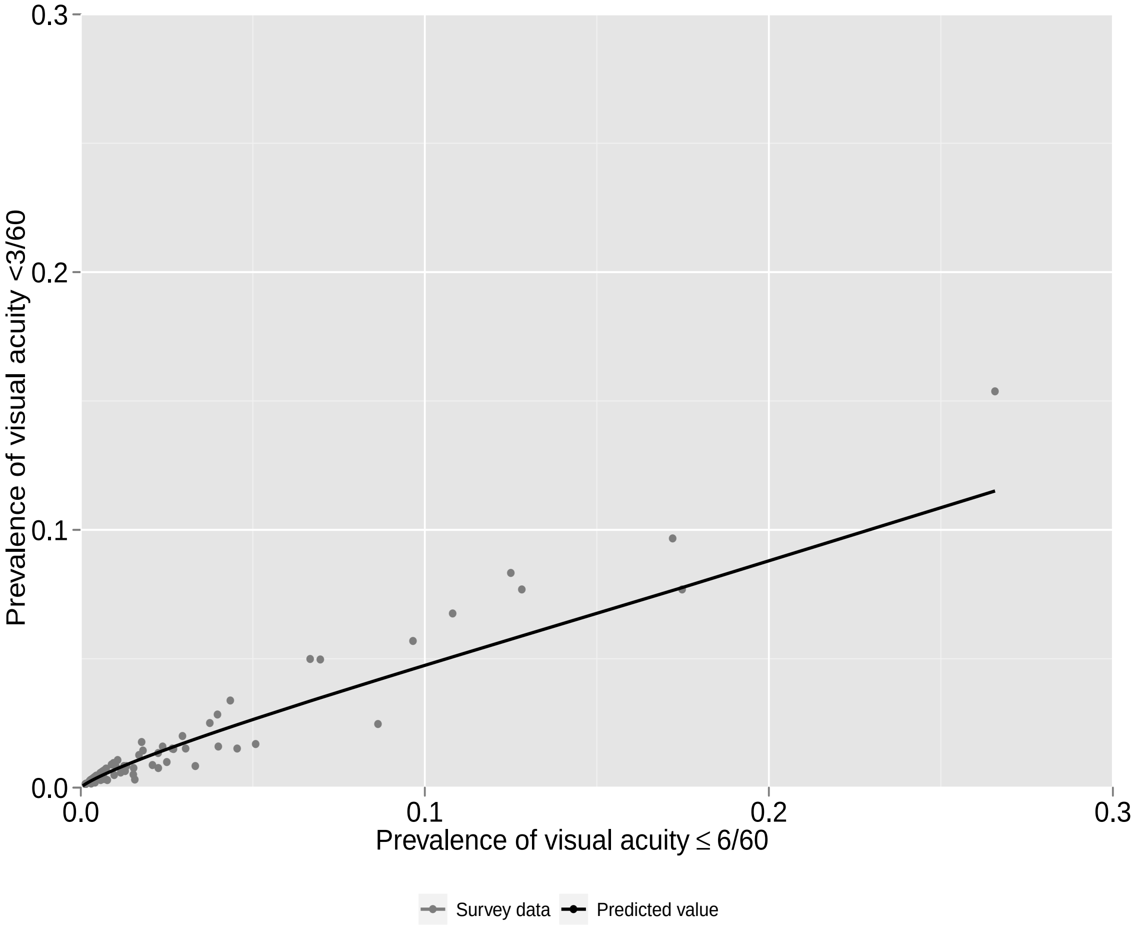

Figure B: Conversion from visual acuity ≤6/60 to blind (< 3/60)

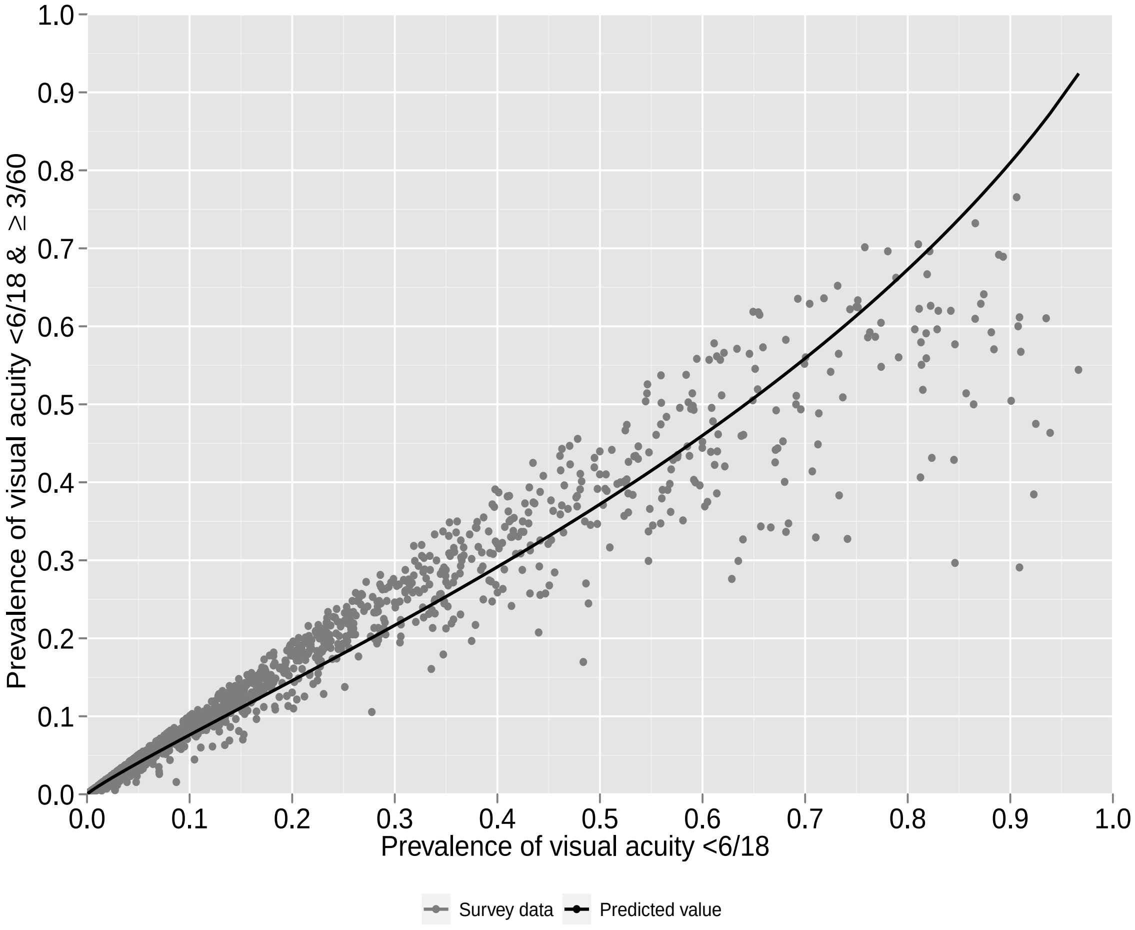

Figure C: Conversion from visual acuity $<6/18$ to MSVI ($< 6/18$ and $\geq 3/60$)

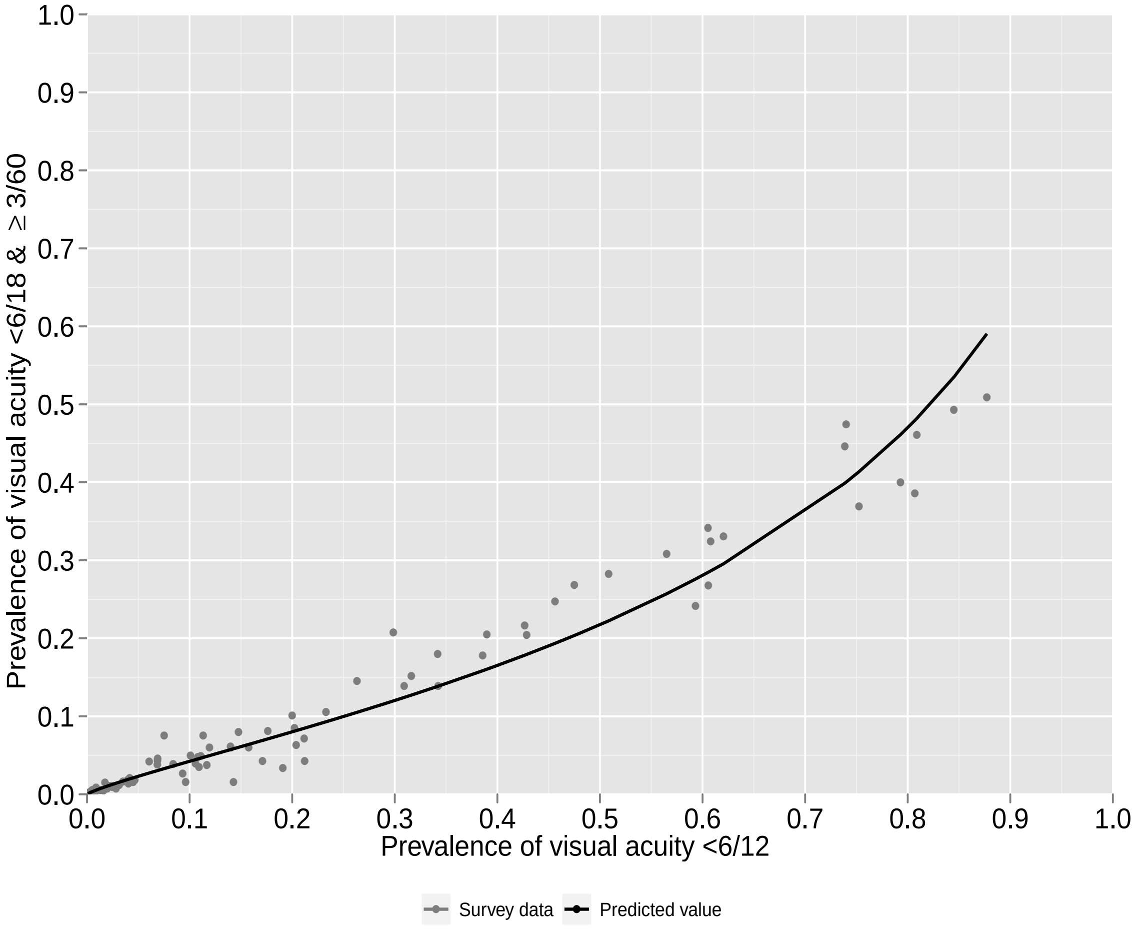

Figure D: Conversion from visual acuity $<6/12$ to MSVI ($< 6/18$ and $\geq 3/60$)

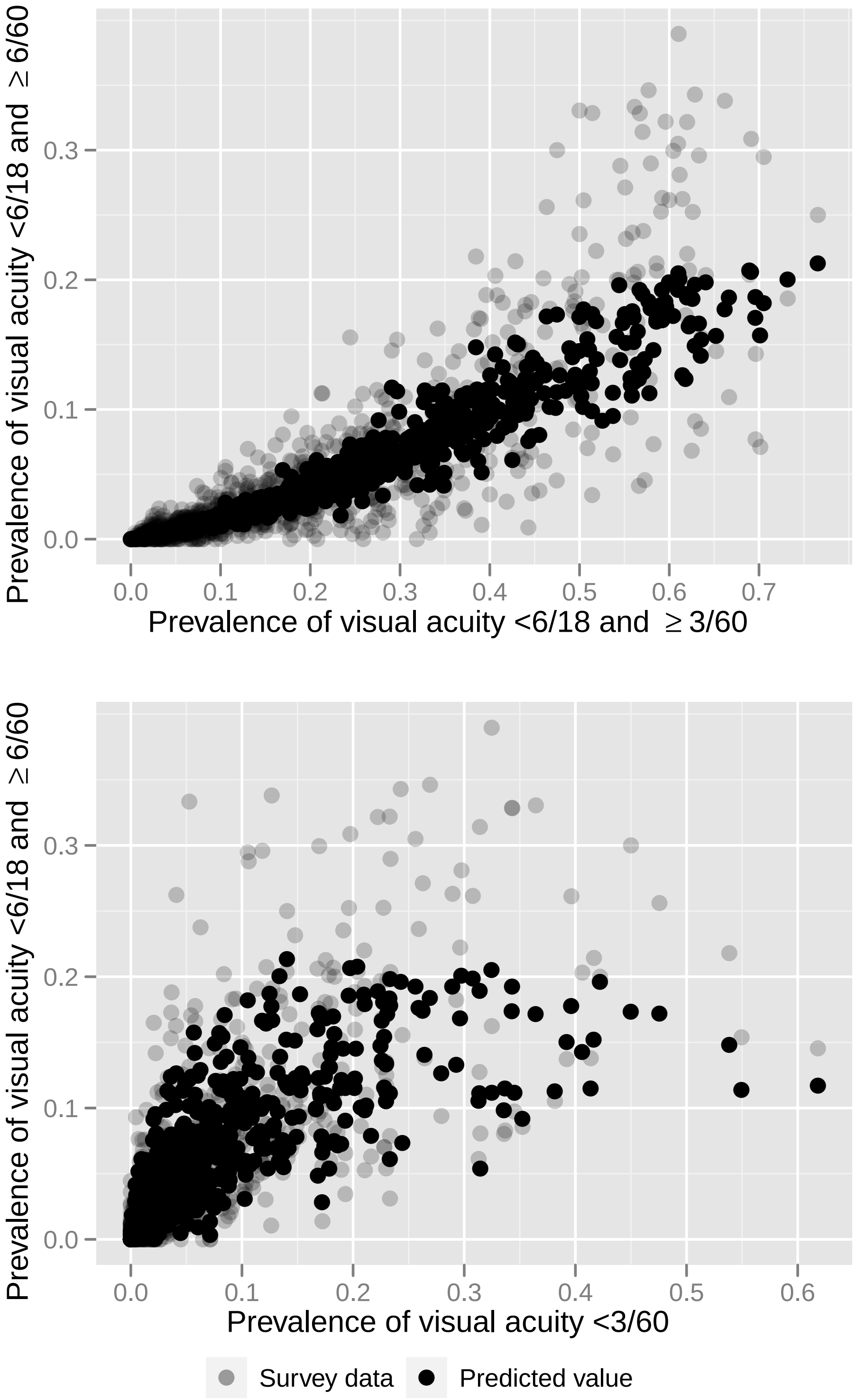

Figure E: Prediction of severe vision impairment prevalence from MSVI and blind prevalence

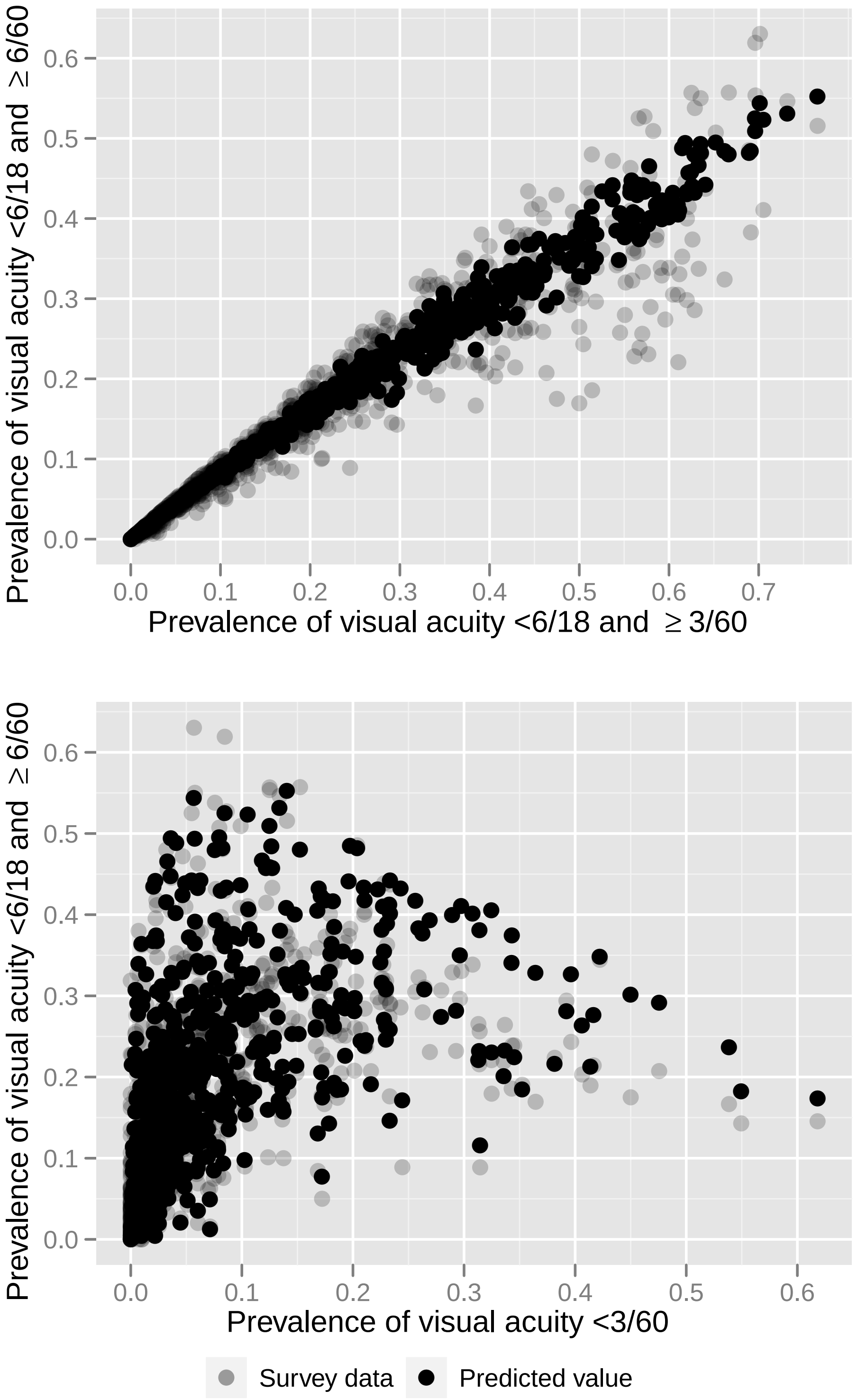

Figure F: Prediction of moderate vision impairment prevalence from MSVI and blind prevalence

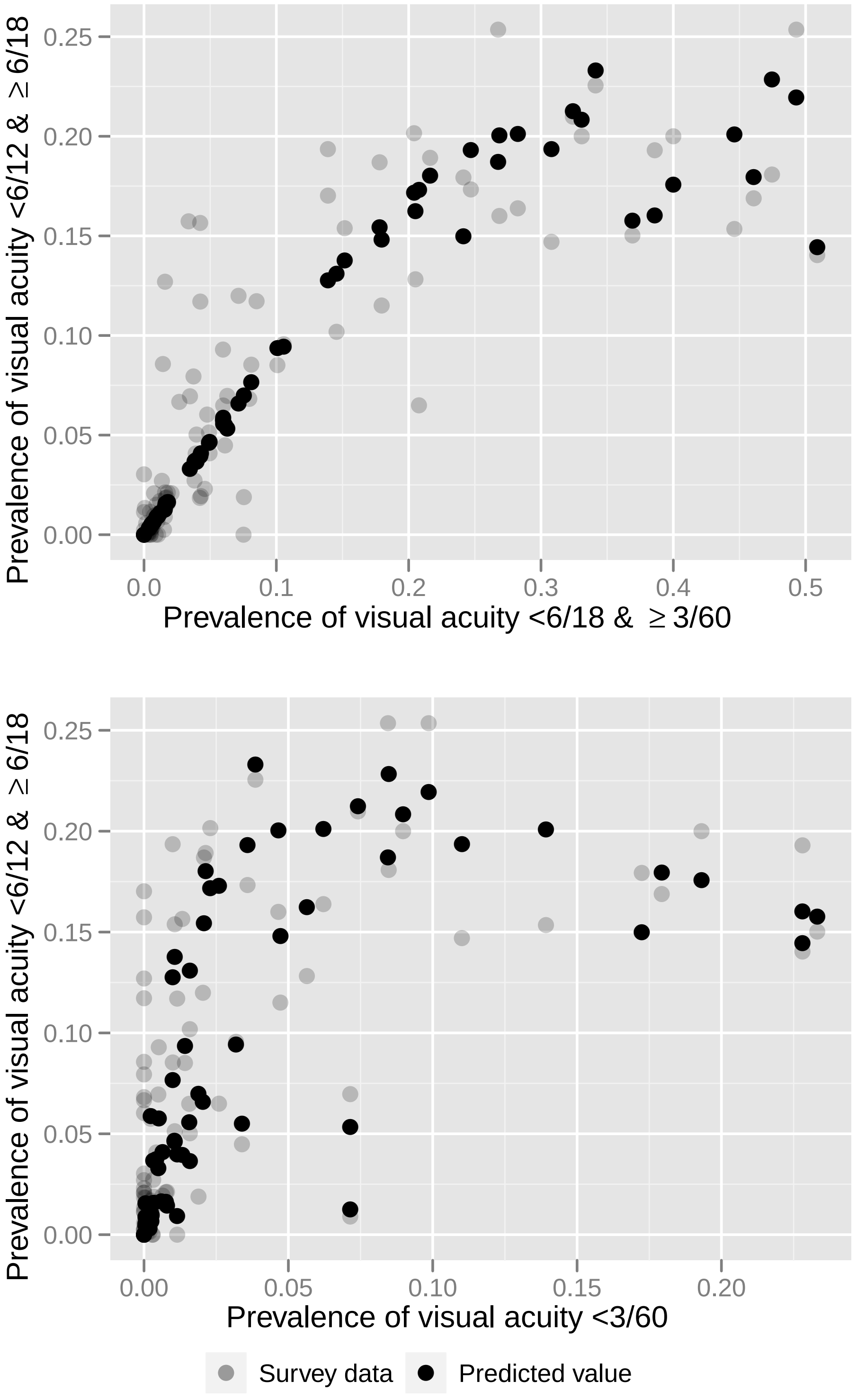

Figure G: Prediction of mild vision impairment prevalence from MSVI and blind prevalence

Figure H Survey data and model estimates. The model's central estimate is shown in red, and the shaded grey area shows the 95% uncertainty interval.

+ Presenting
+ Best corrected

National
Sub−national

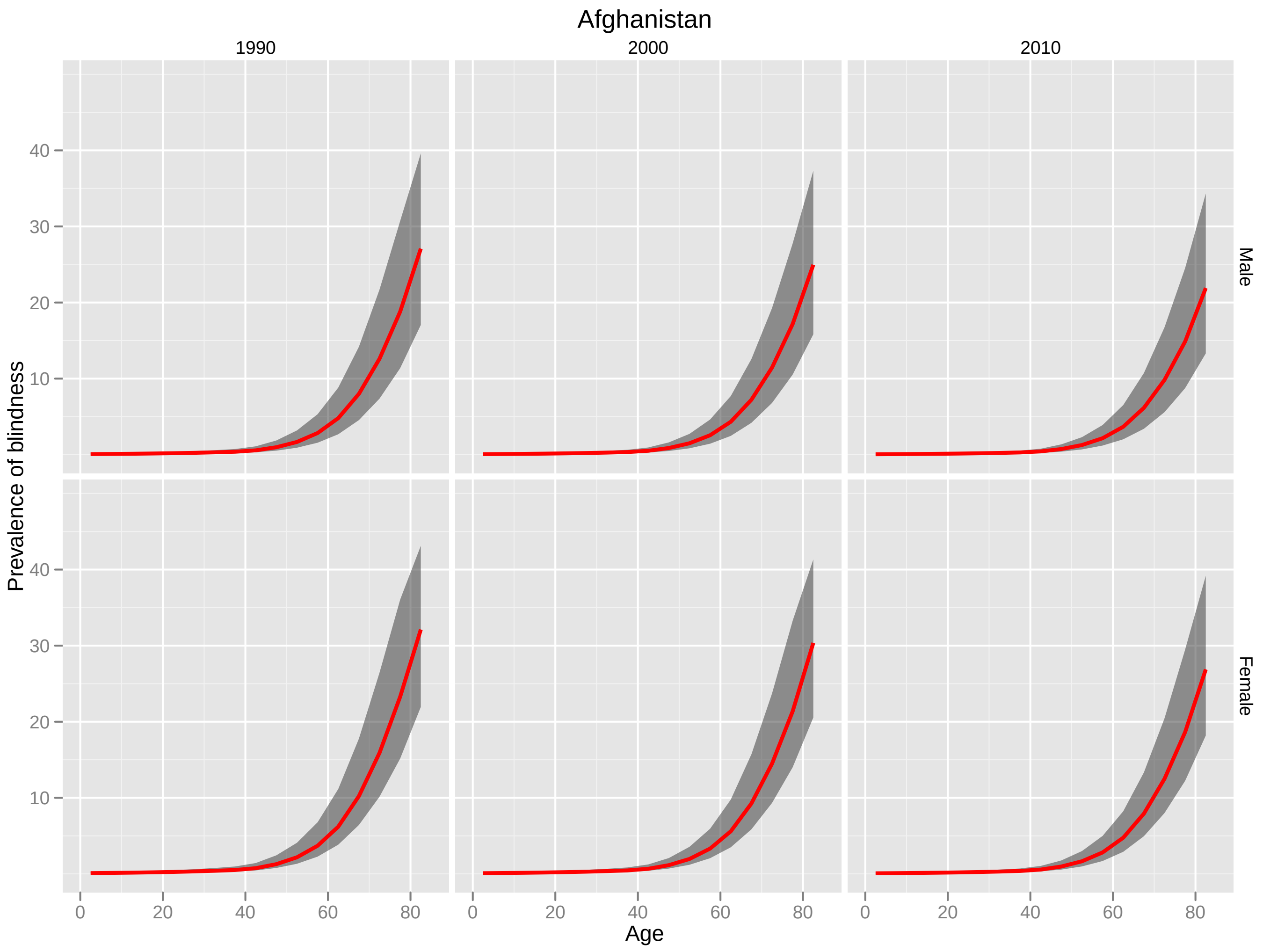
Afghanistan
1990
2000
2010
Male
Female
Prevalence of blindness
10
20
30
40
0
20
40
60
80
Age

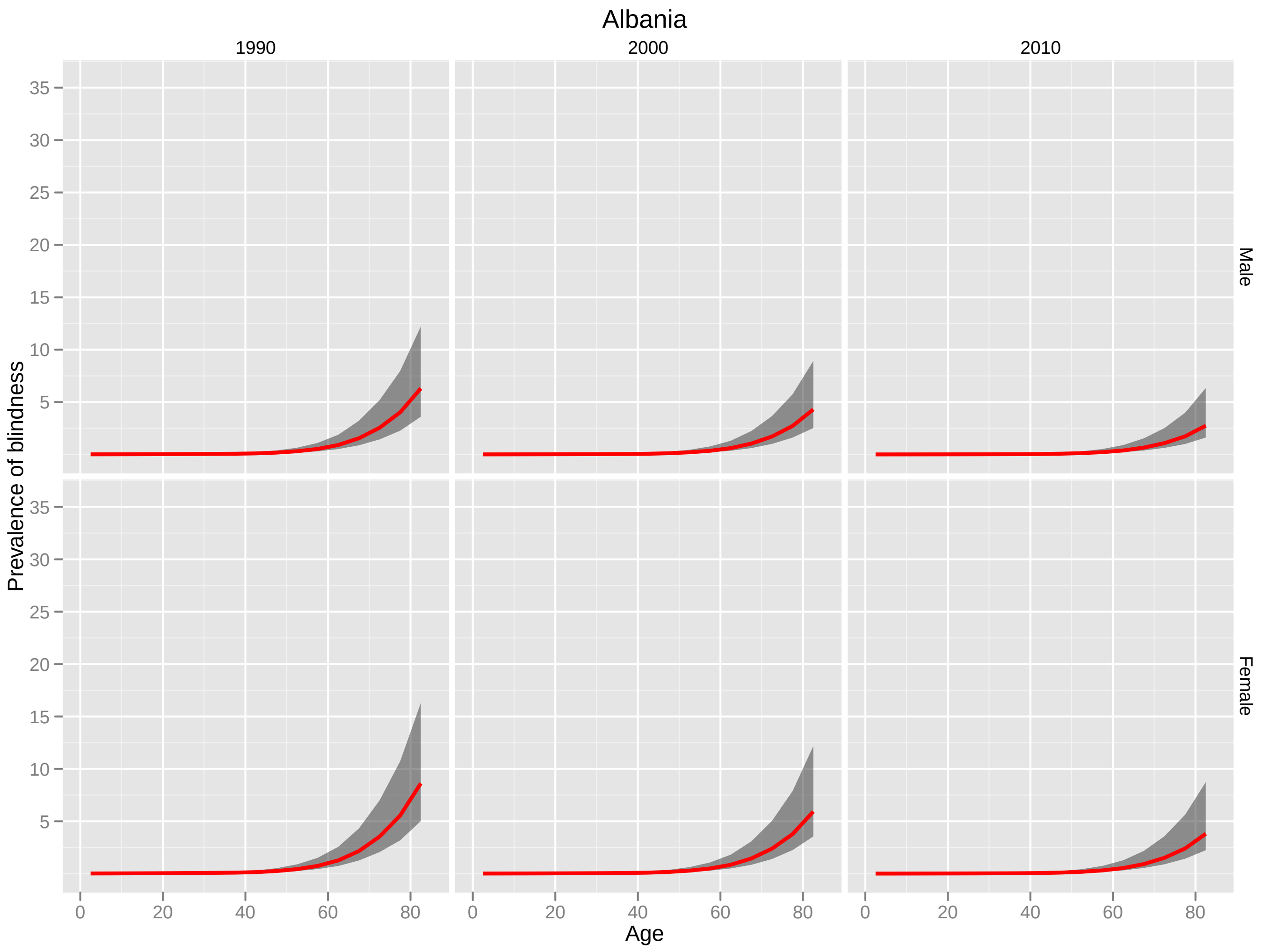
Albania
1990
2000
2010
Male
Female
Prevalence of blindness
Age
0
20
40
60
80
5
10
15
20
25
30
35

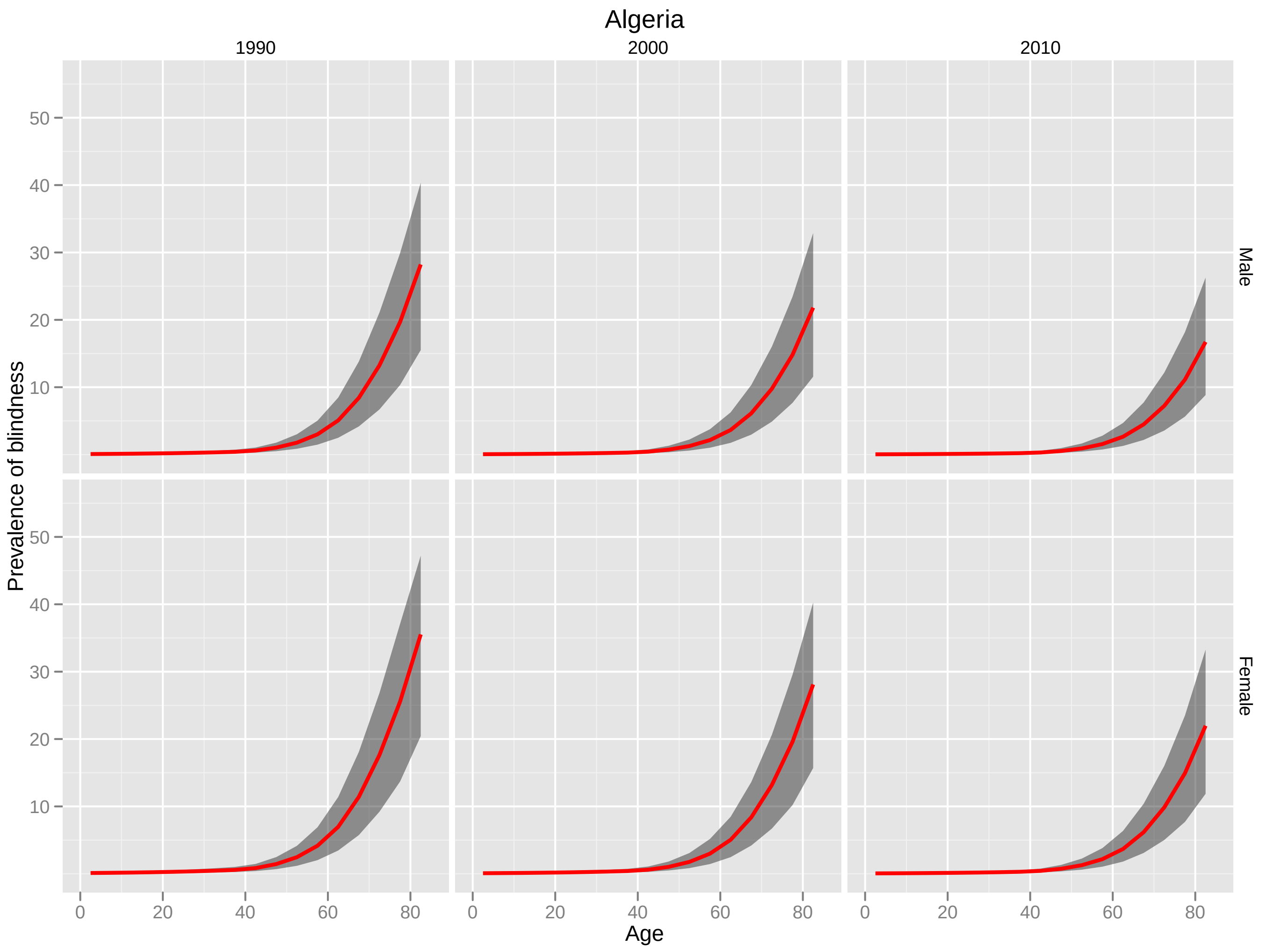
Algeria
1990
2000
2010
Male
Female
Prevalence of blindness
Age
10
20
30
40
50
0
20
40
60
80

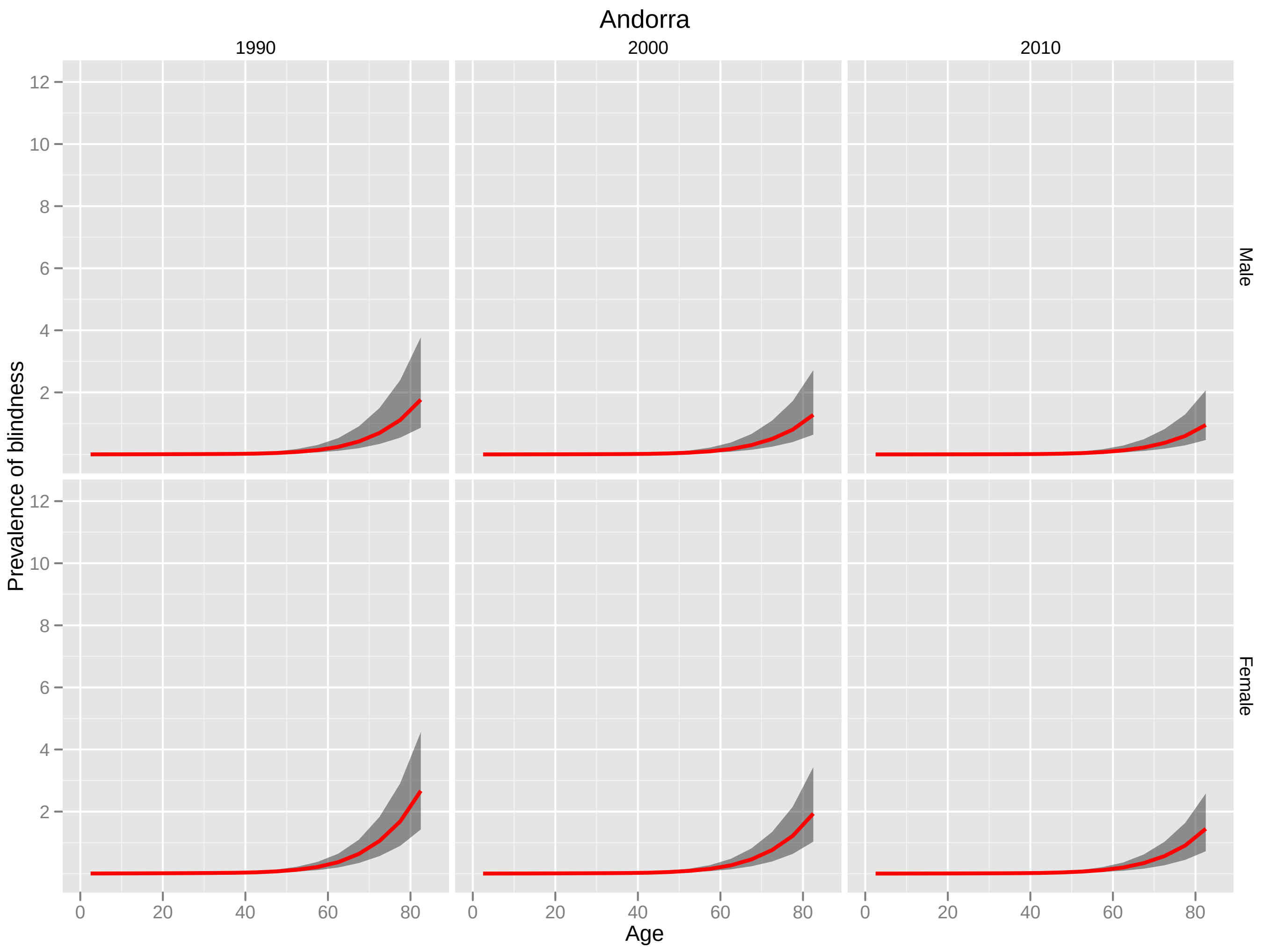
Andorra
1990
2000
2010
Male
Female
Prevalence of blindness
Age
0
20
40
60
80
2
4
6
8
10
12

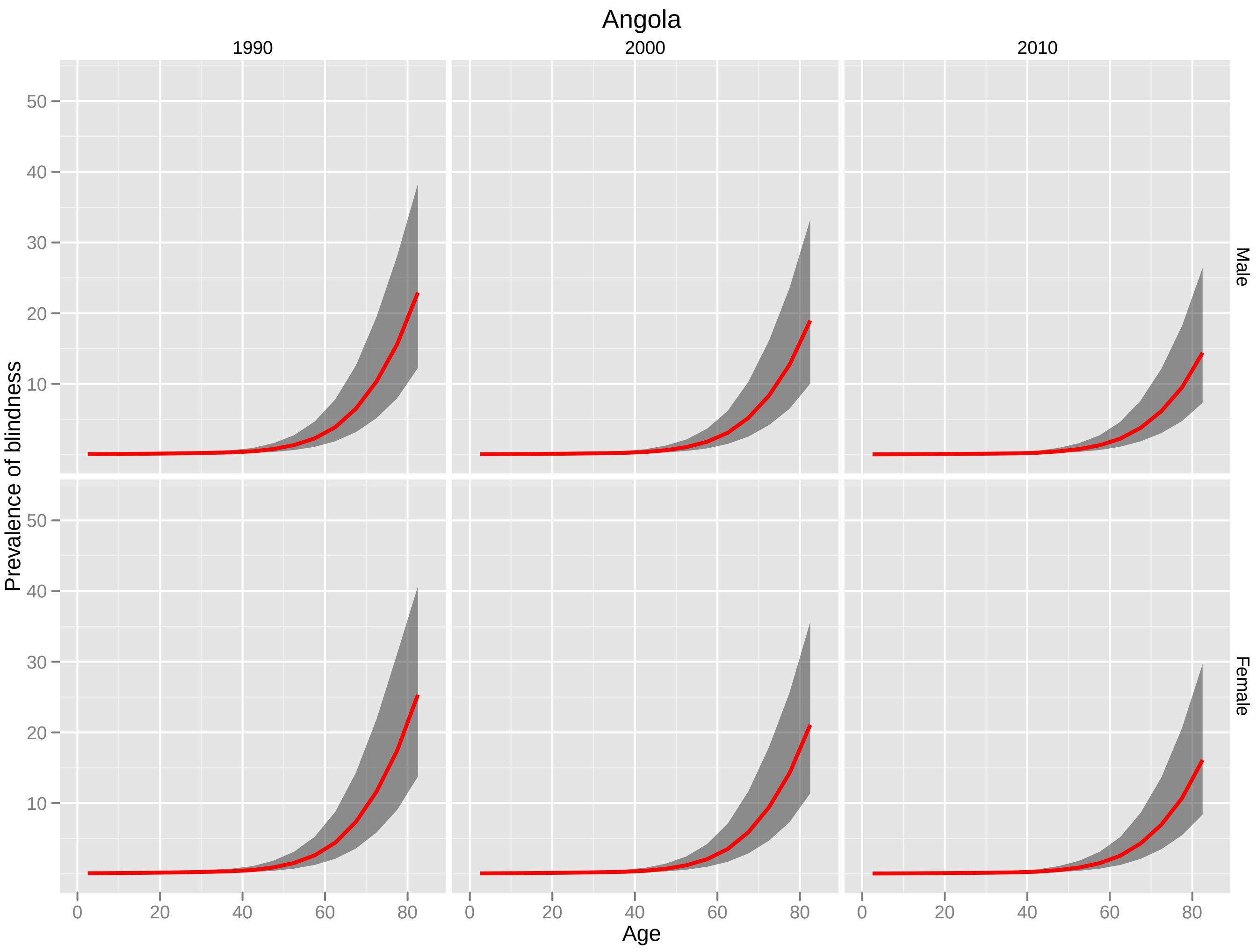
Angola
1990
2000
2010
Male
Female
Prevalence of blindness
Age
10
20
30
40
50
0
20
40
60
80

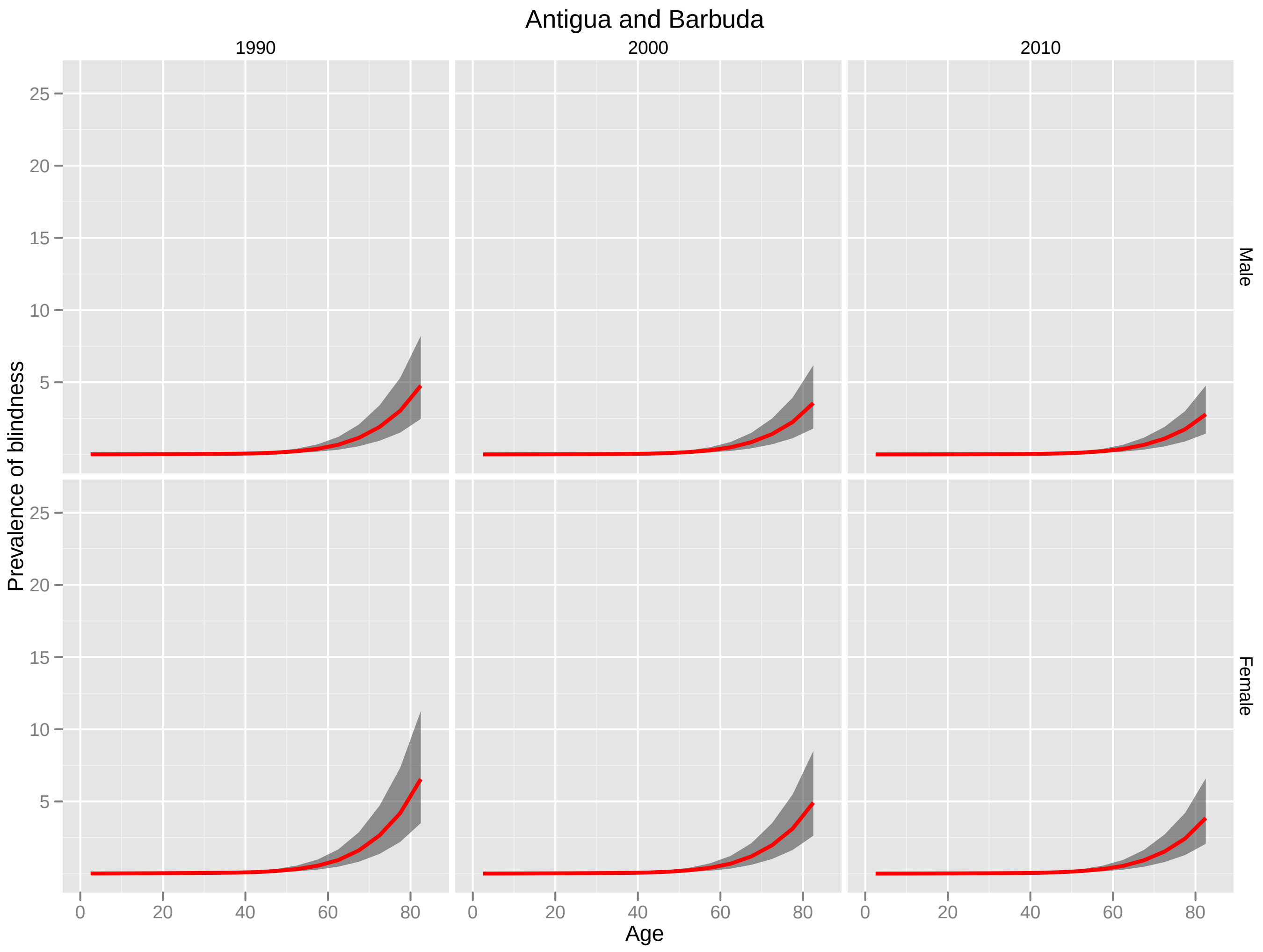
Antigua and Barbuda
1990
2000
2010
Male
Female
Prevalence of blindness
Age
0
20
40
60
80
5
10
15
20
25

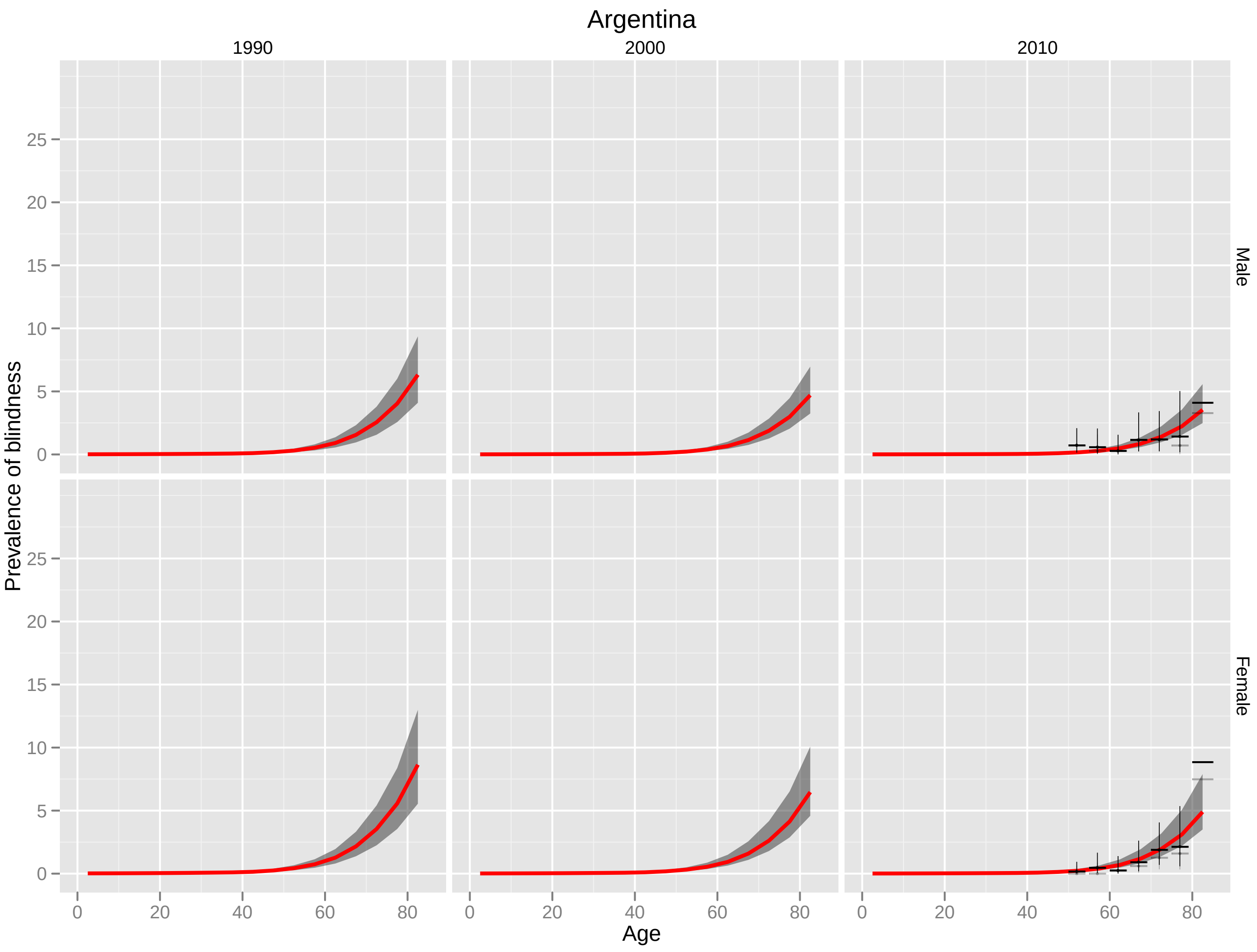
Argentina
1990
2000
2010
Male
Female
Prevalence of blindness
Age
0
5
10
15
20
25
0
20
40
60
80

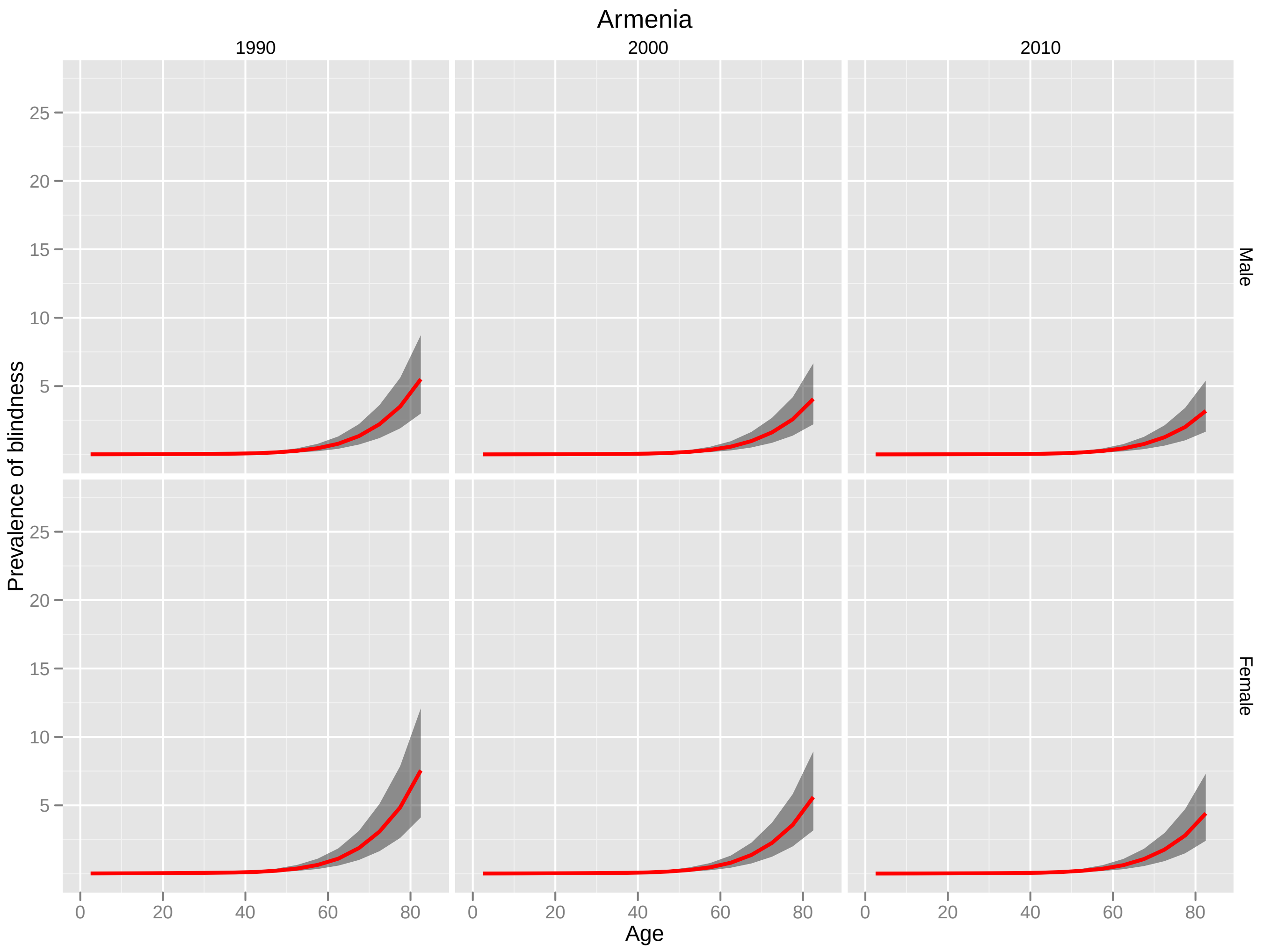
Armenia
1990
2000
2010
Male
Female
Prevalence of blindness
Age
0
20
40
60
80
5
10
15
20
25

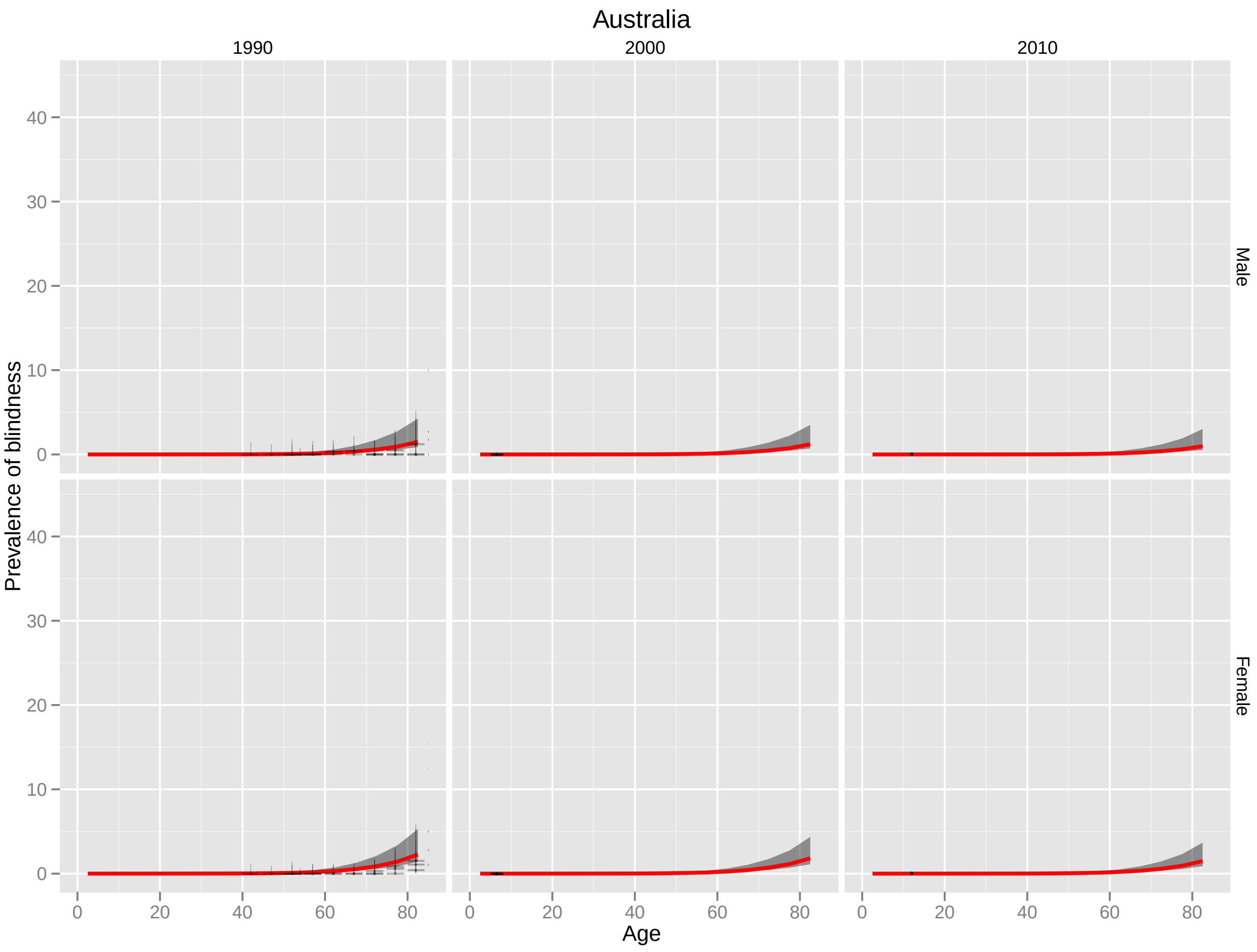
Australia
1990
2000
2010
Male
Female
Prevalence of blindness
0
10
20
30
40
Age
0
20
40
60
80

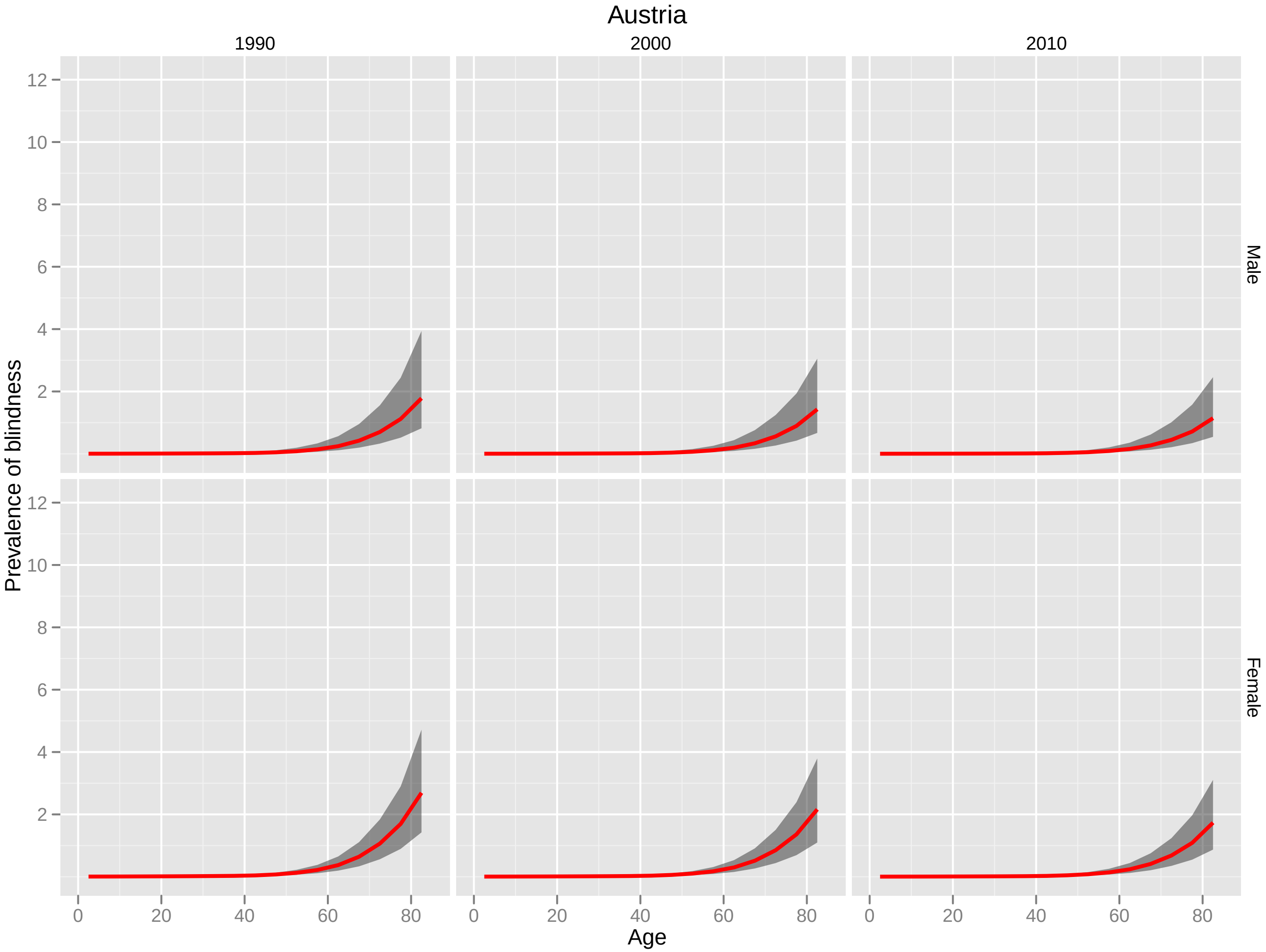
Austria
1990
2000
2010
Male
Female
Prevalence of blindness
Age
0
20
40
60
80
2
4
6
8
10
12

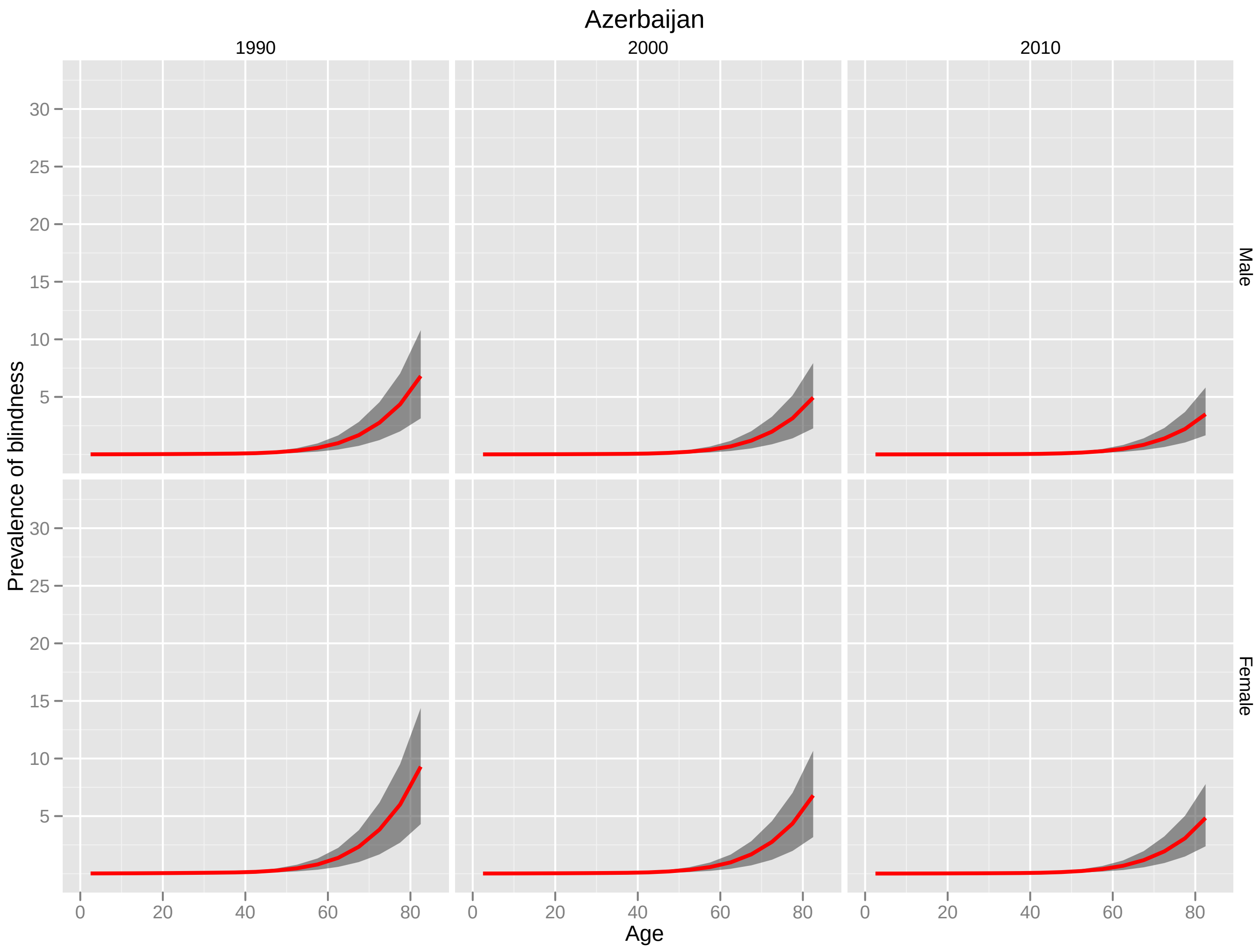
Azerbaijan
1990
2000
2010
Male
Female
Prevalence of blindness
Age
30
25
20
15
10
5
0
20
40
60
80

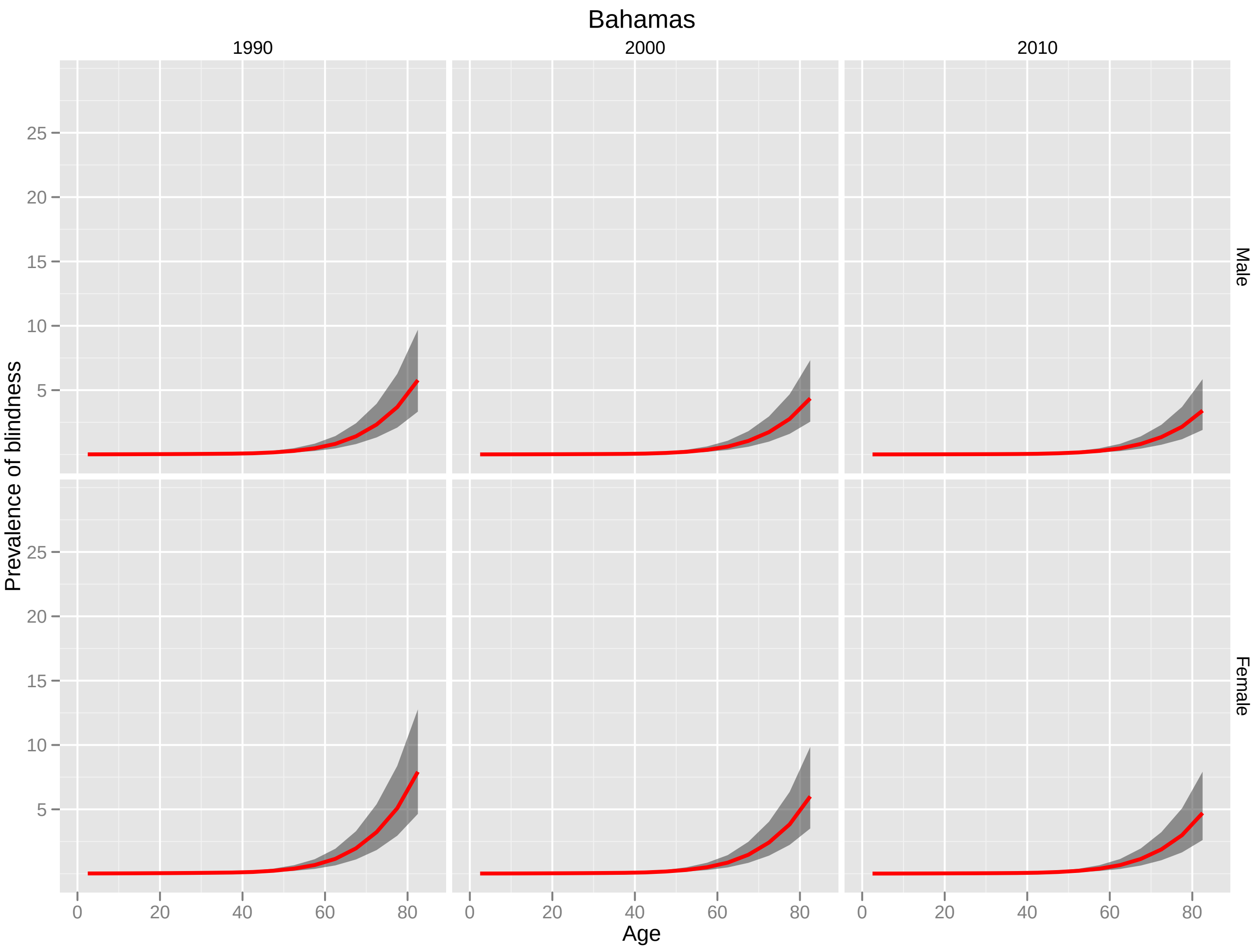
Bahamas
1990
2000
2010
Male
Female
Prevalence of blindness
Age
5
10
15
20
25
0
20
40
60
80

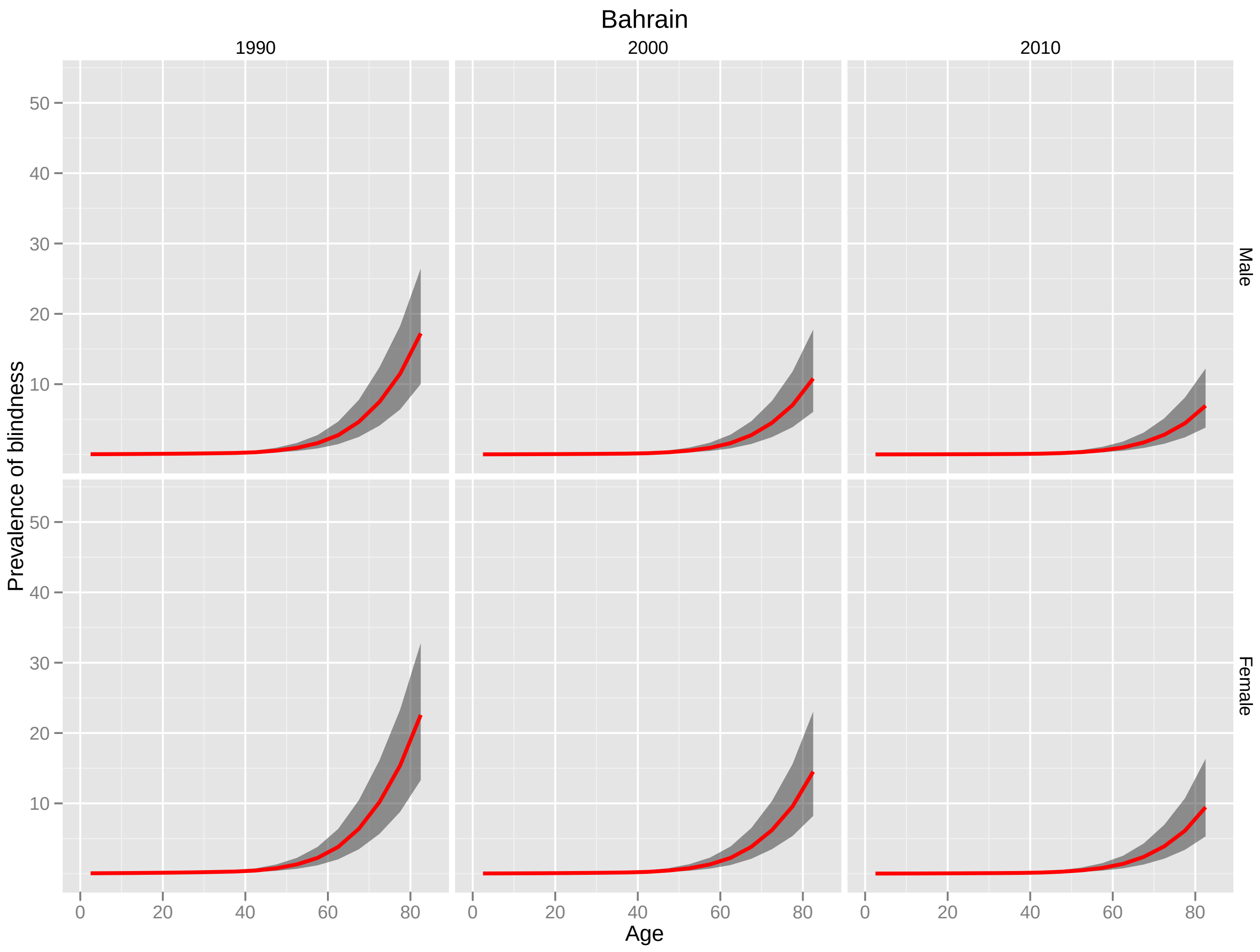
Bahrain
1990
2000
2010
Male
Female
Prevalence of blindness
Age
10
20
30
40
50
0
20
40
60
80

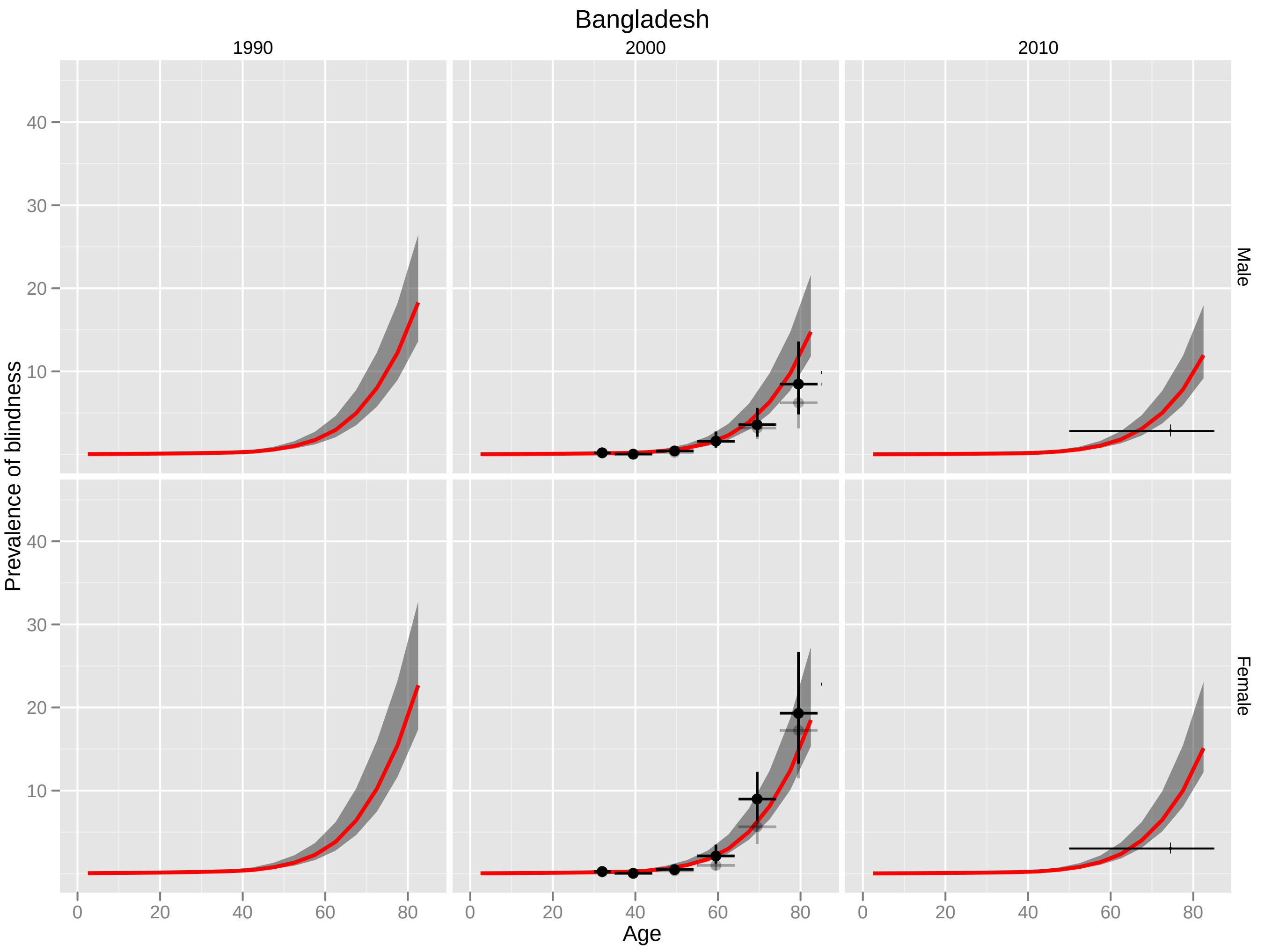
Bangladesh
1990
2000
2010
Male
Female
Prevalence of blindness
Age
10
20
30
40
0
20
40
60
80

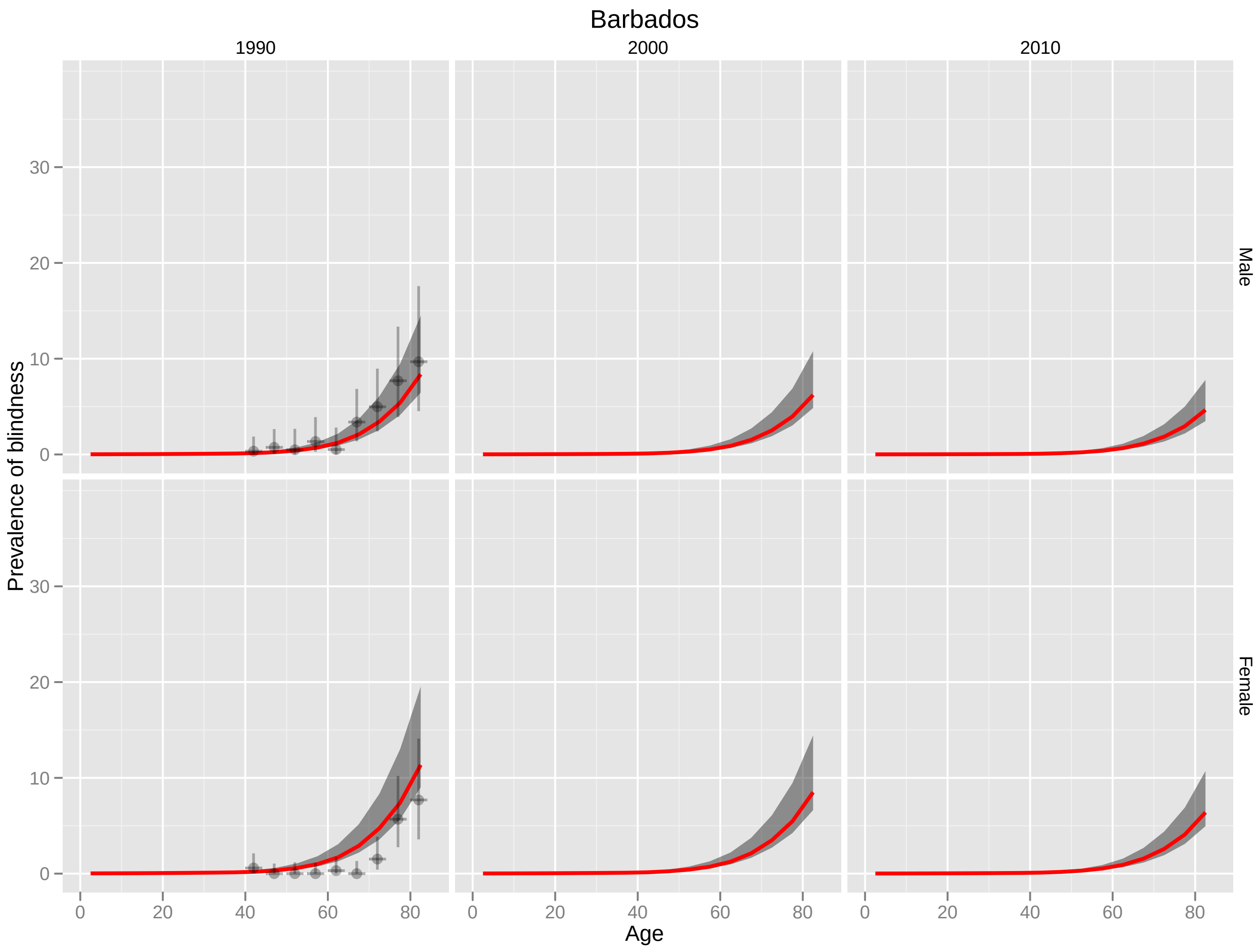
Barbados
1990
2000
2010
Male
Female
Prevalence of blindness
0
10
20
30
Age
0
20
40
60
80

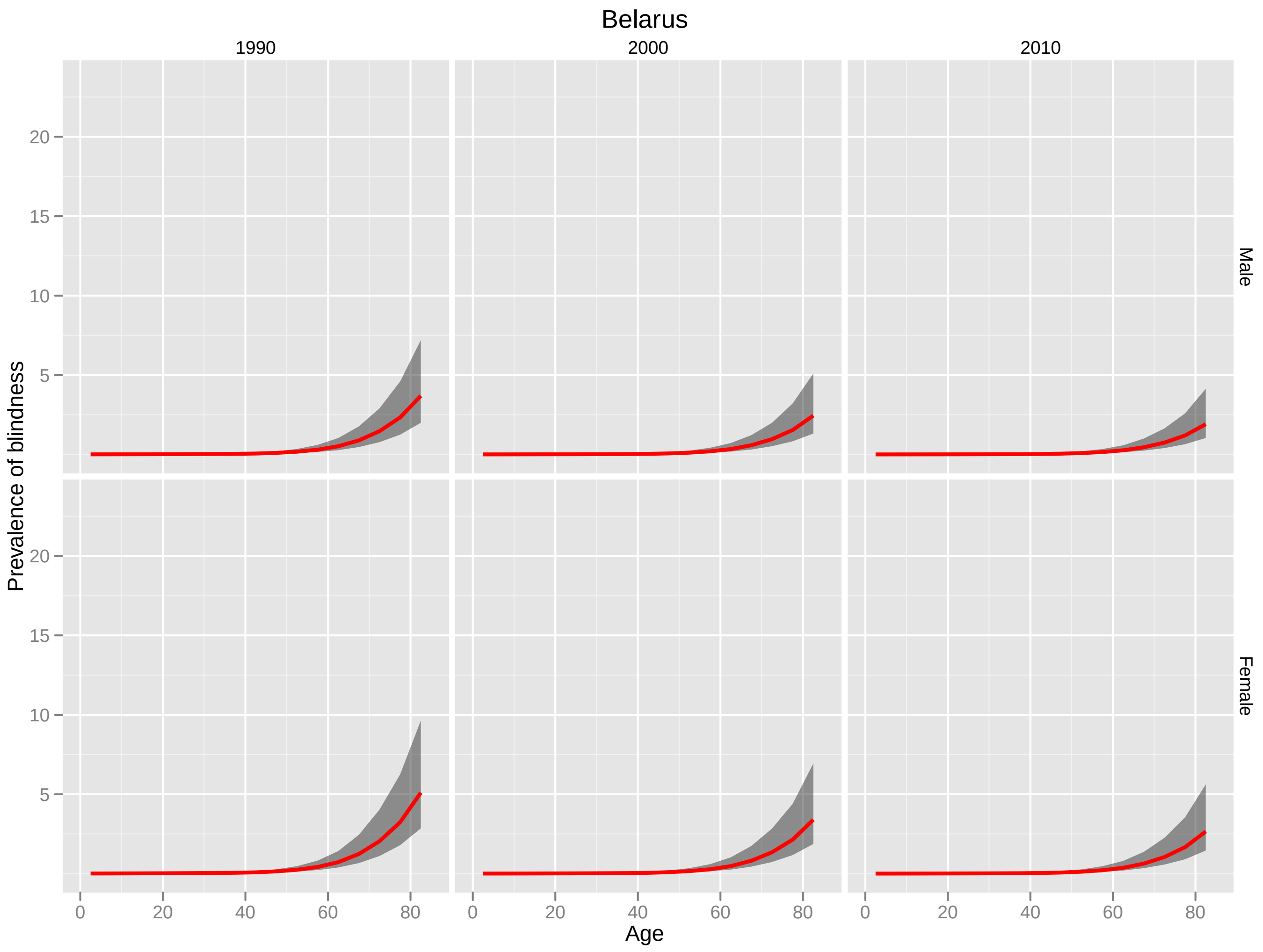
Belarus
1990
2000
2010
Male
Female
Prevalence of blindness
Age
0
20
40
60
80
5
10
15
20

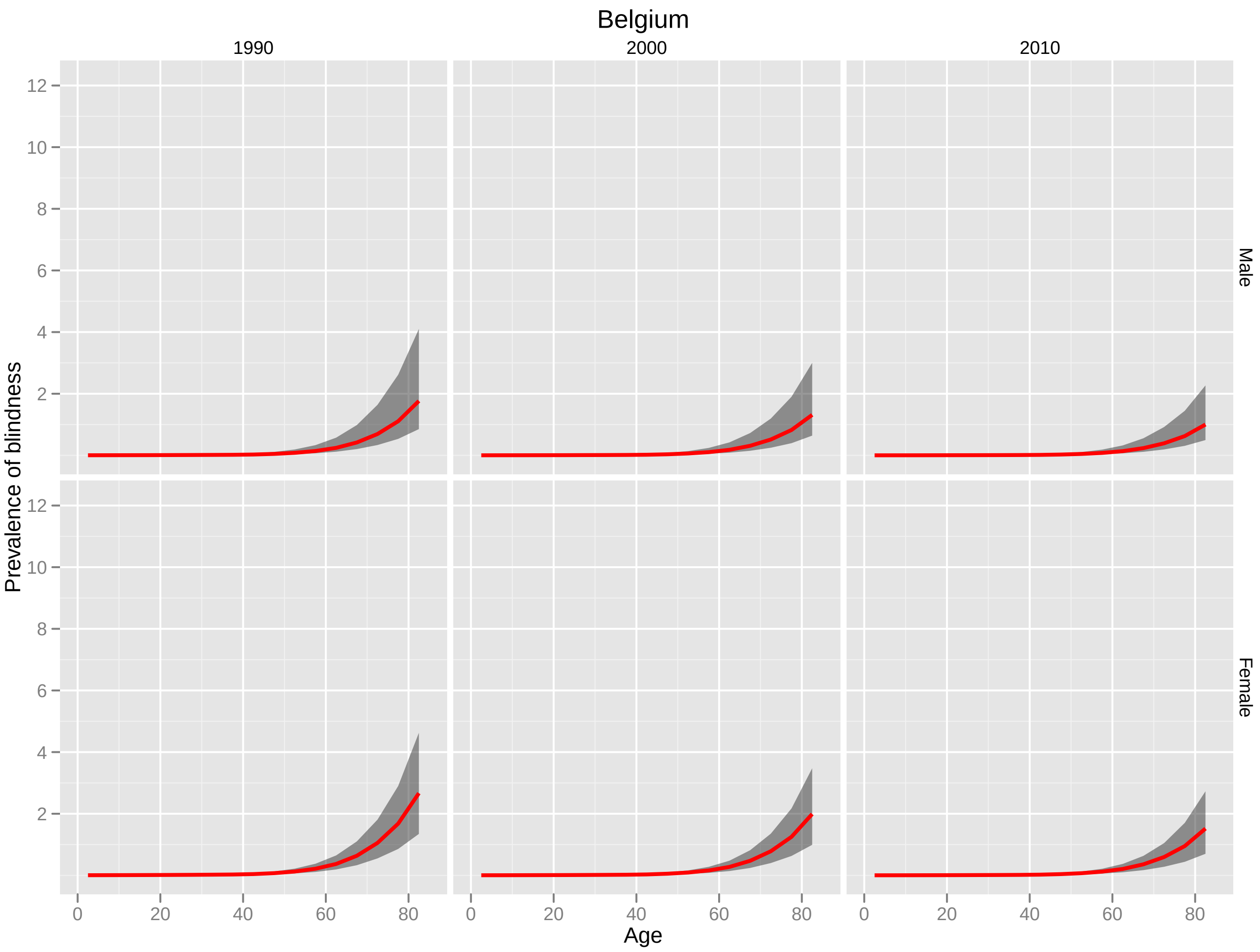
Belgium
1990
2000
2010
Male
Female
Prevalence of blindness
Age
0
20
40
60
80
2
4
6
8
10
12

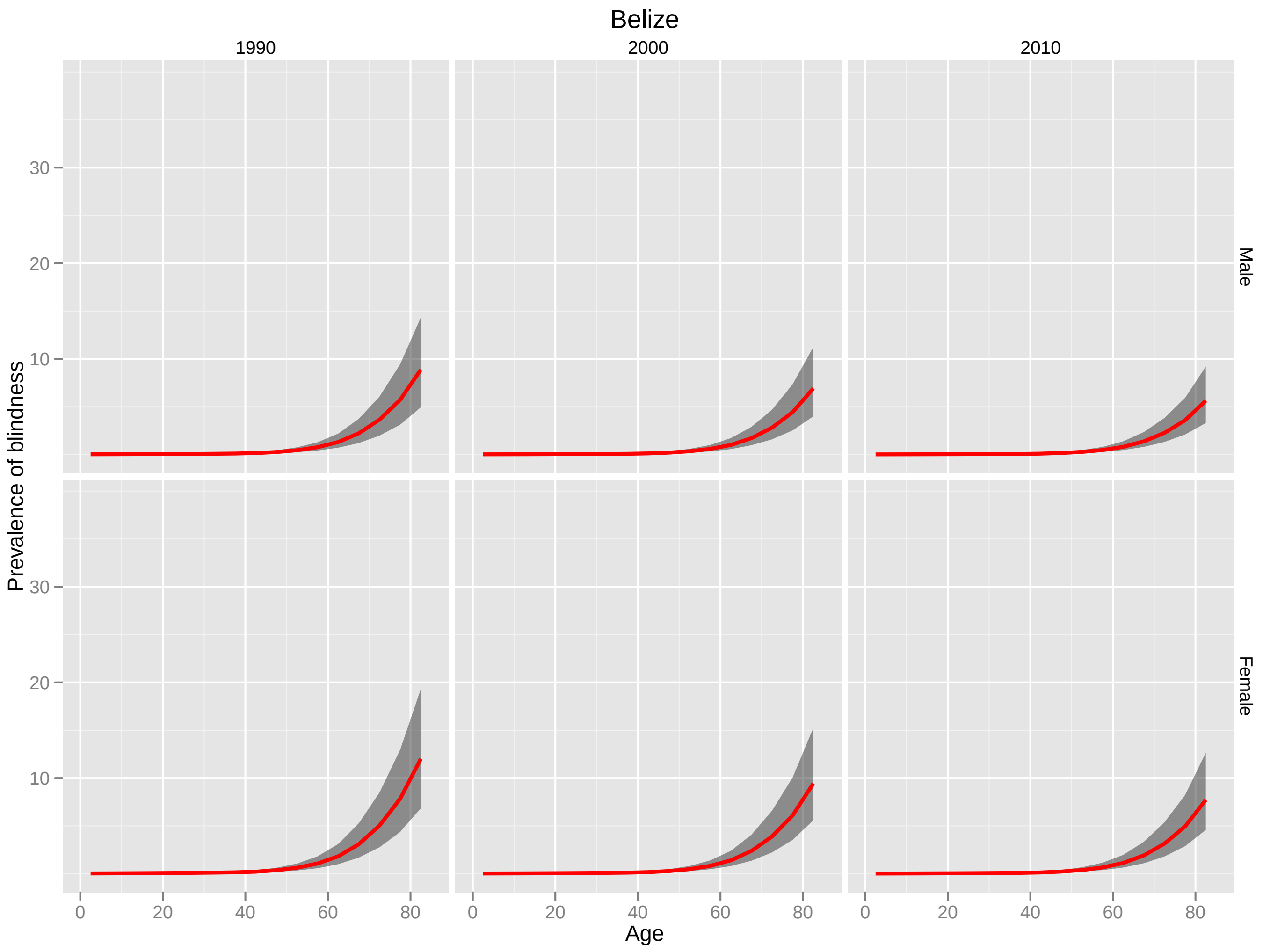

Belize
1990
2000
2010
Male
Female
Prevalence of blindness
Age
30
20
10
0
20
40
60
80

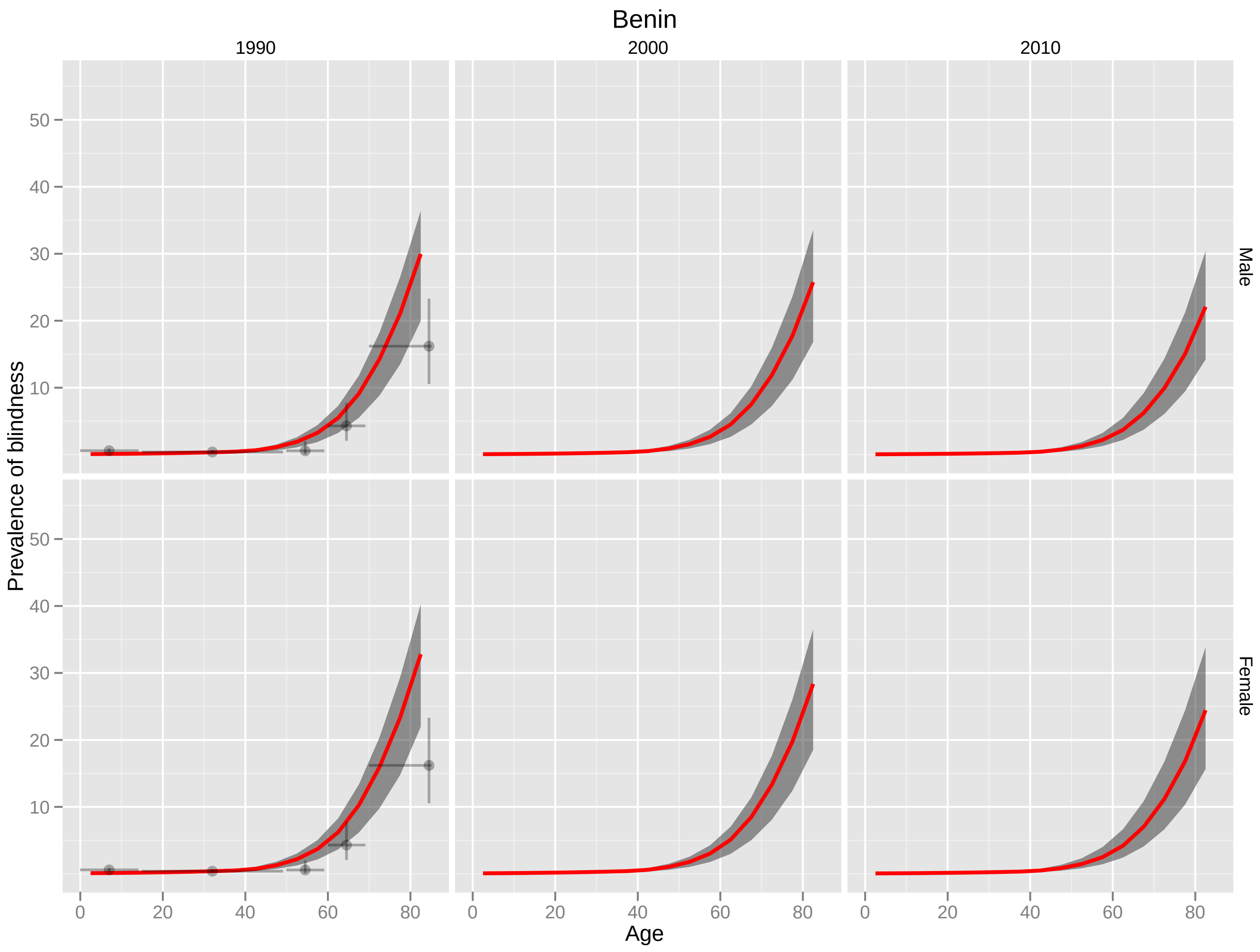
Benin
1990
2000
2010
Male
Female
Prevalence of blindness
Age
10
20
30
40
50
0
20
40
60
80

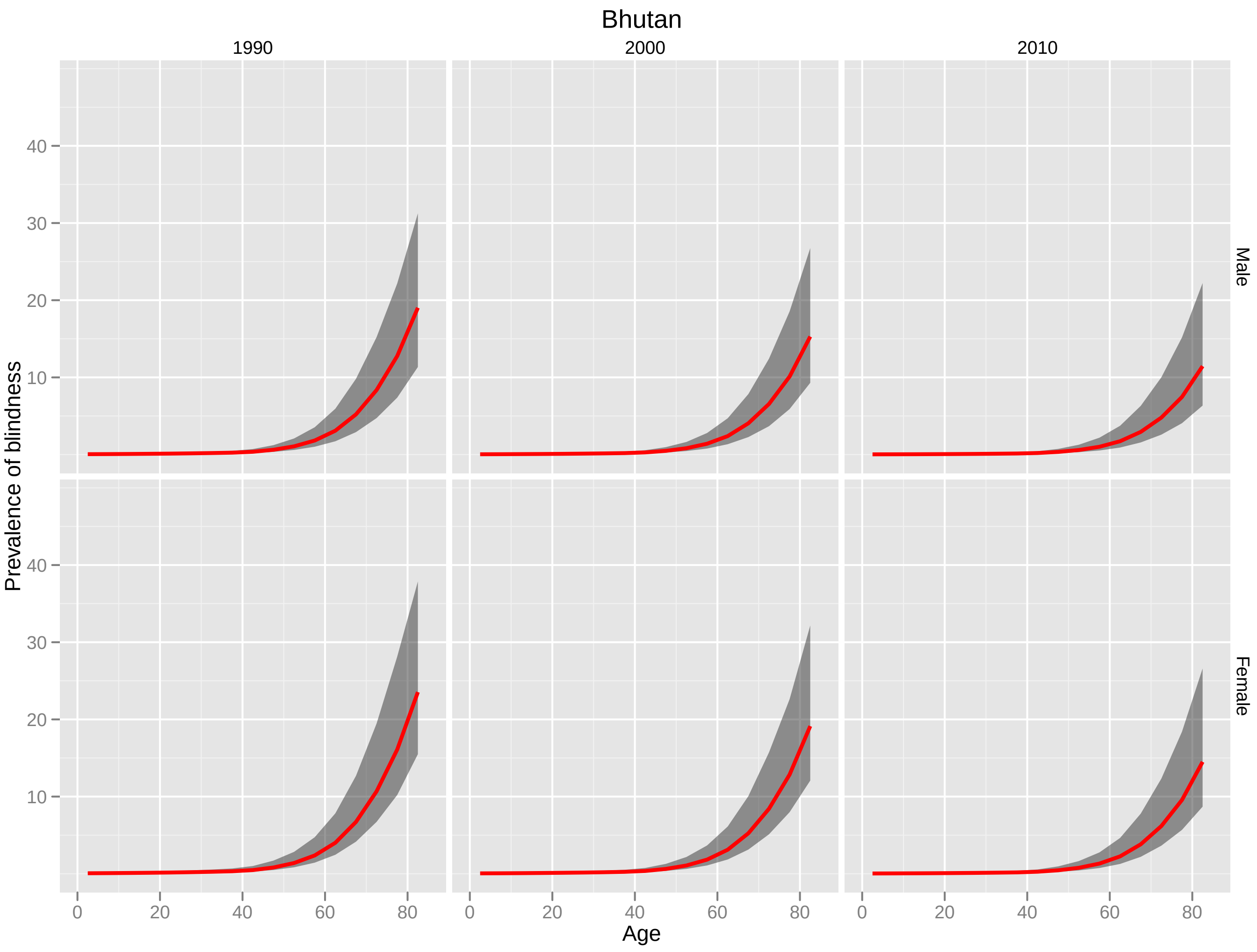

Bhutan
1990
2000
2010
Male
Female
Prevalence of blindness
Age
0
20
40
60
80
10
20
30
40

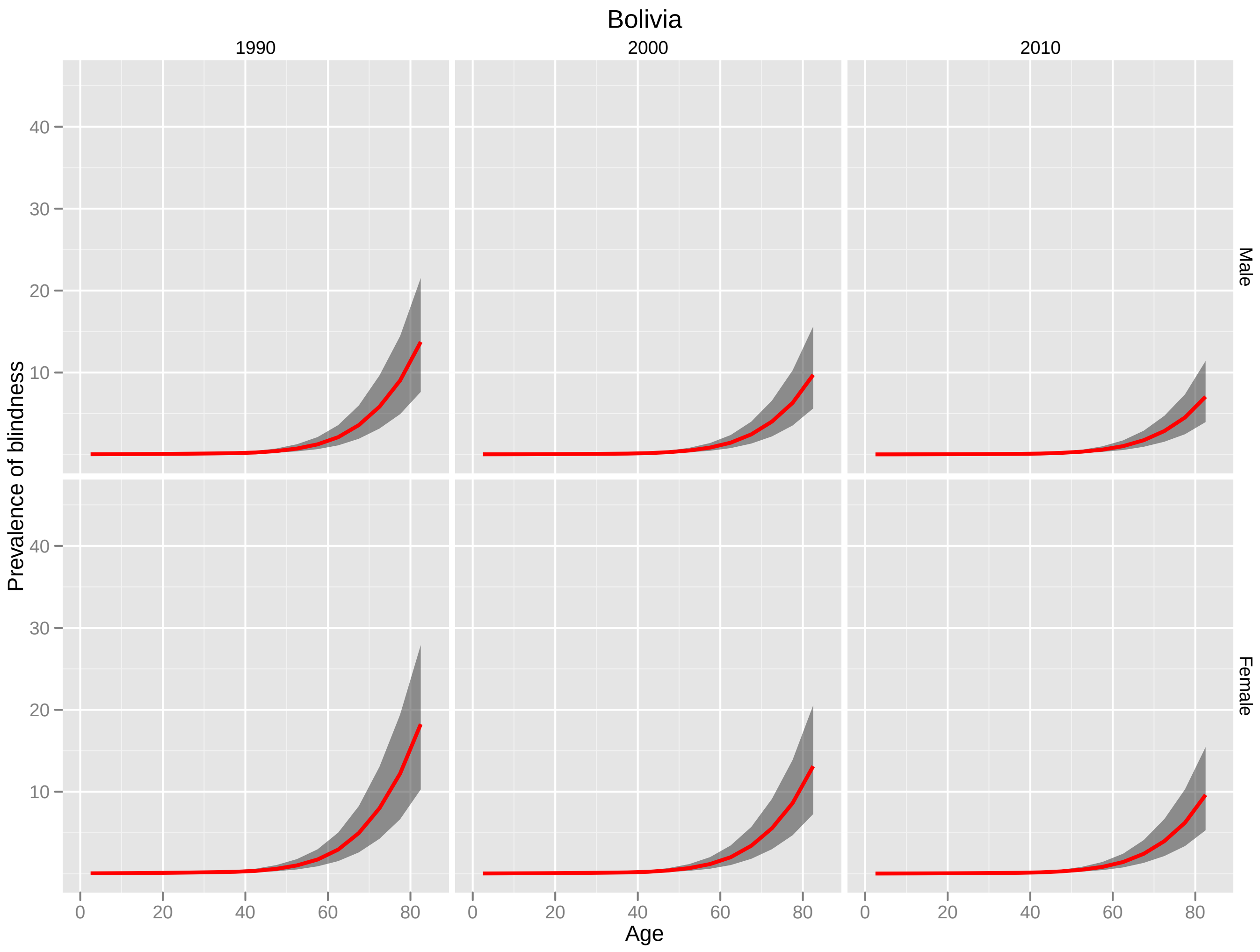
Bolivia
1990
2000
2010
Male
Female
Prevalence of blindness
Age
10
20
30
40
0
20
40
60
80

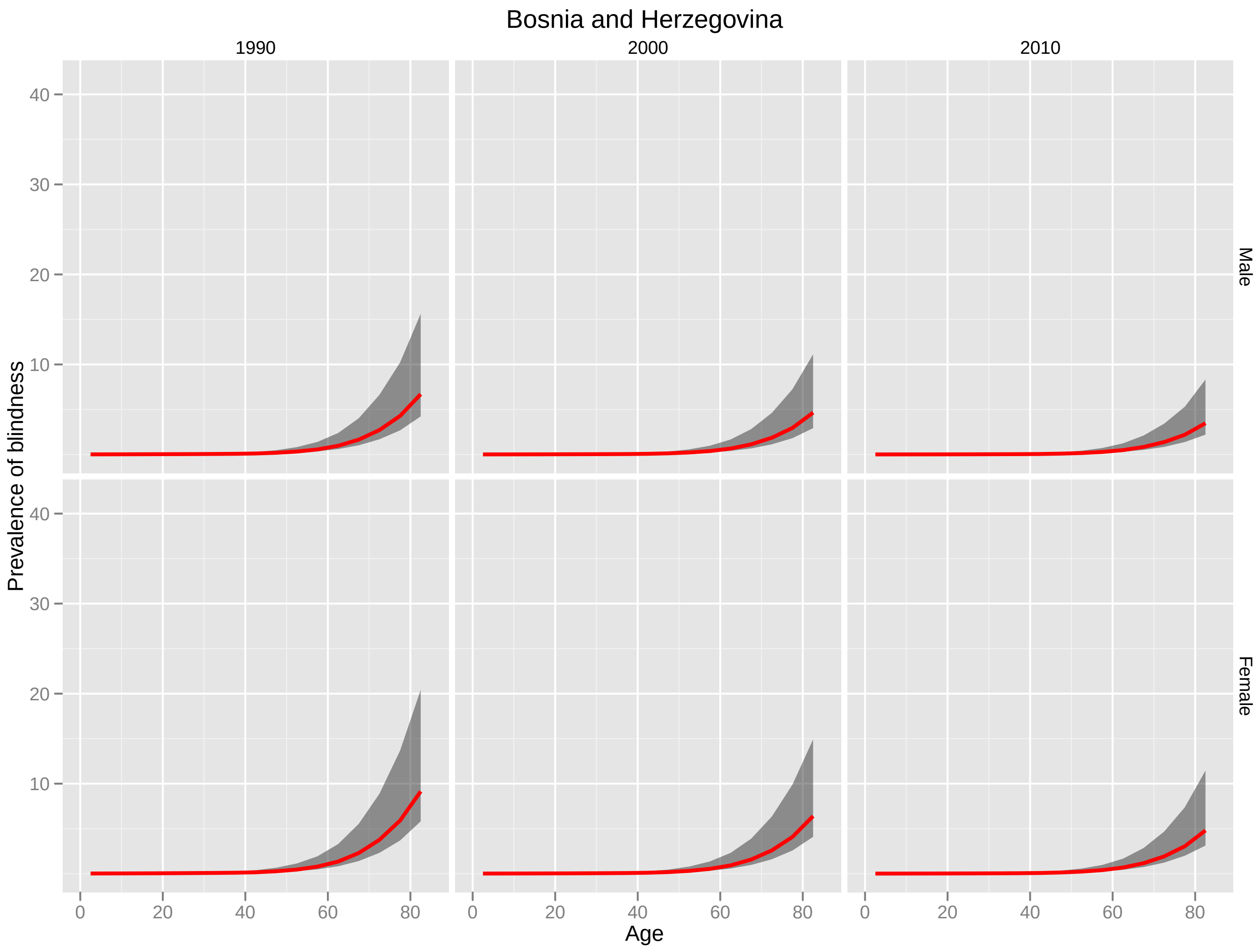
Bosnia and Herzegovina
1990
2000
2010
Male
Female
Prevalence of blindness
Age
0
20
40
60
80
10
20
30
40

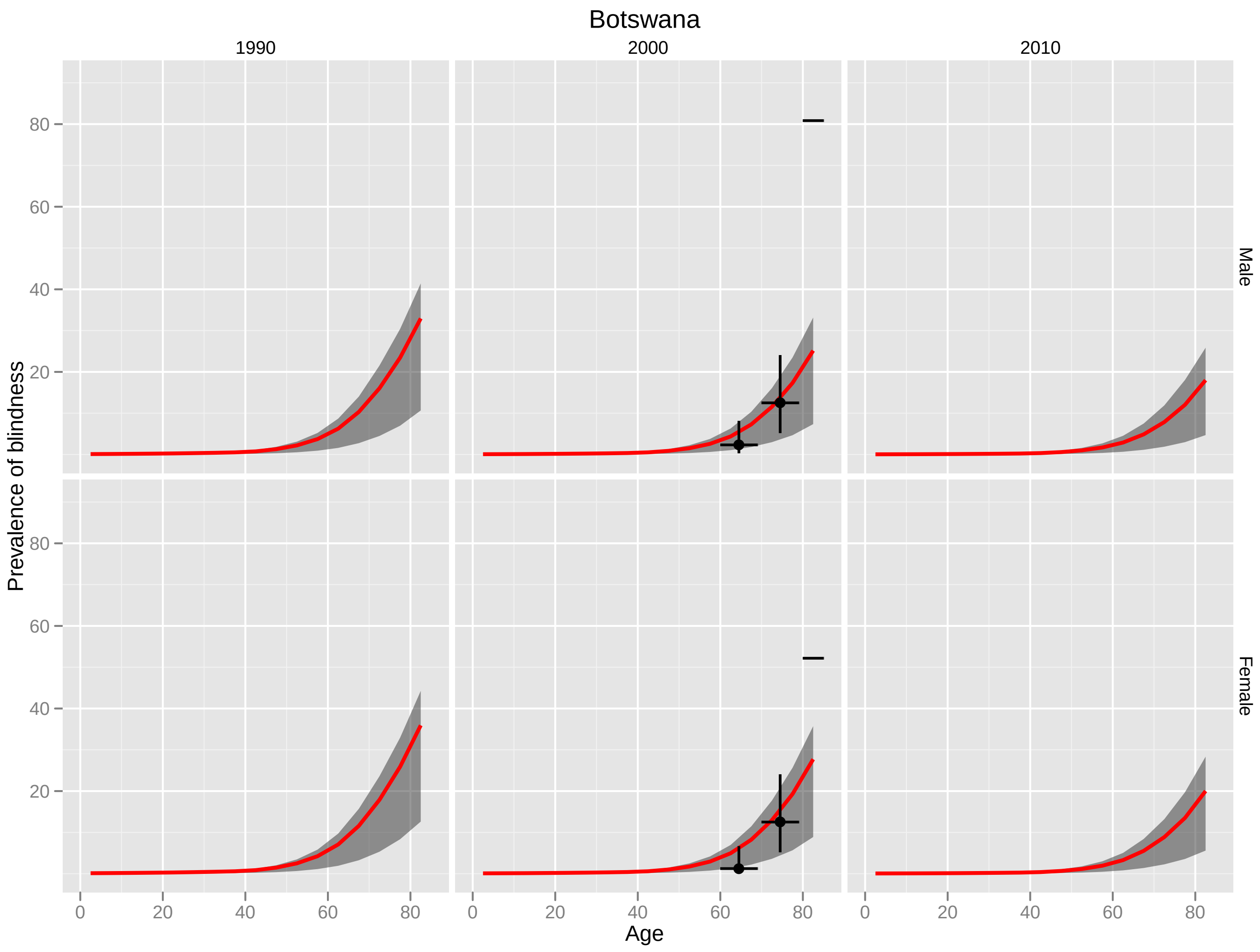
Botswana
1990
2000
2010
Male
Female
Prevalence of blindness
Age
0
20
40
60
80

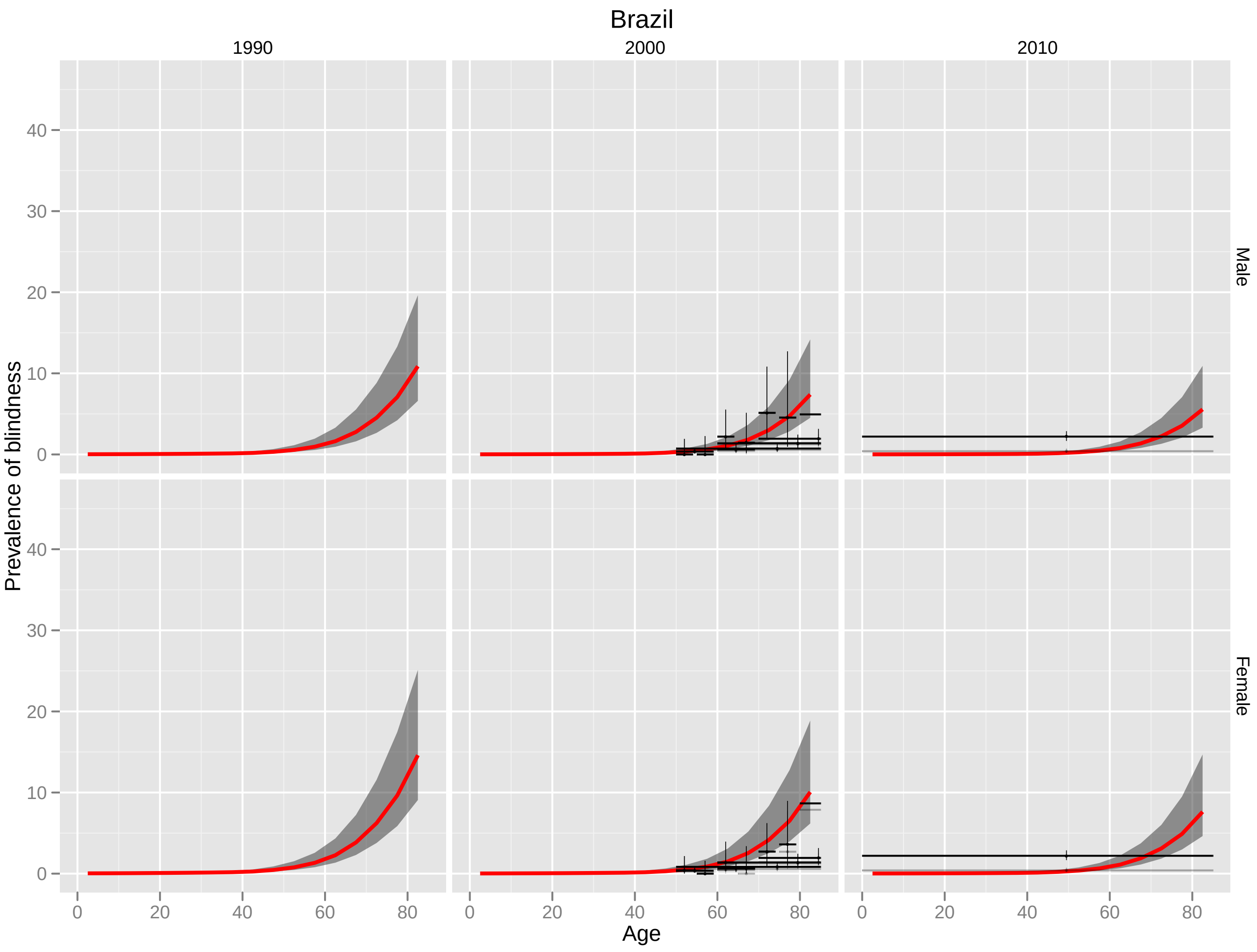

Brazil
1990
2000
2010
Male
Female
Prevalence of blindness
0
10
20
30
40
Age
0
20
40
60
80

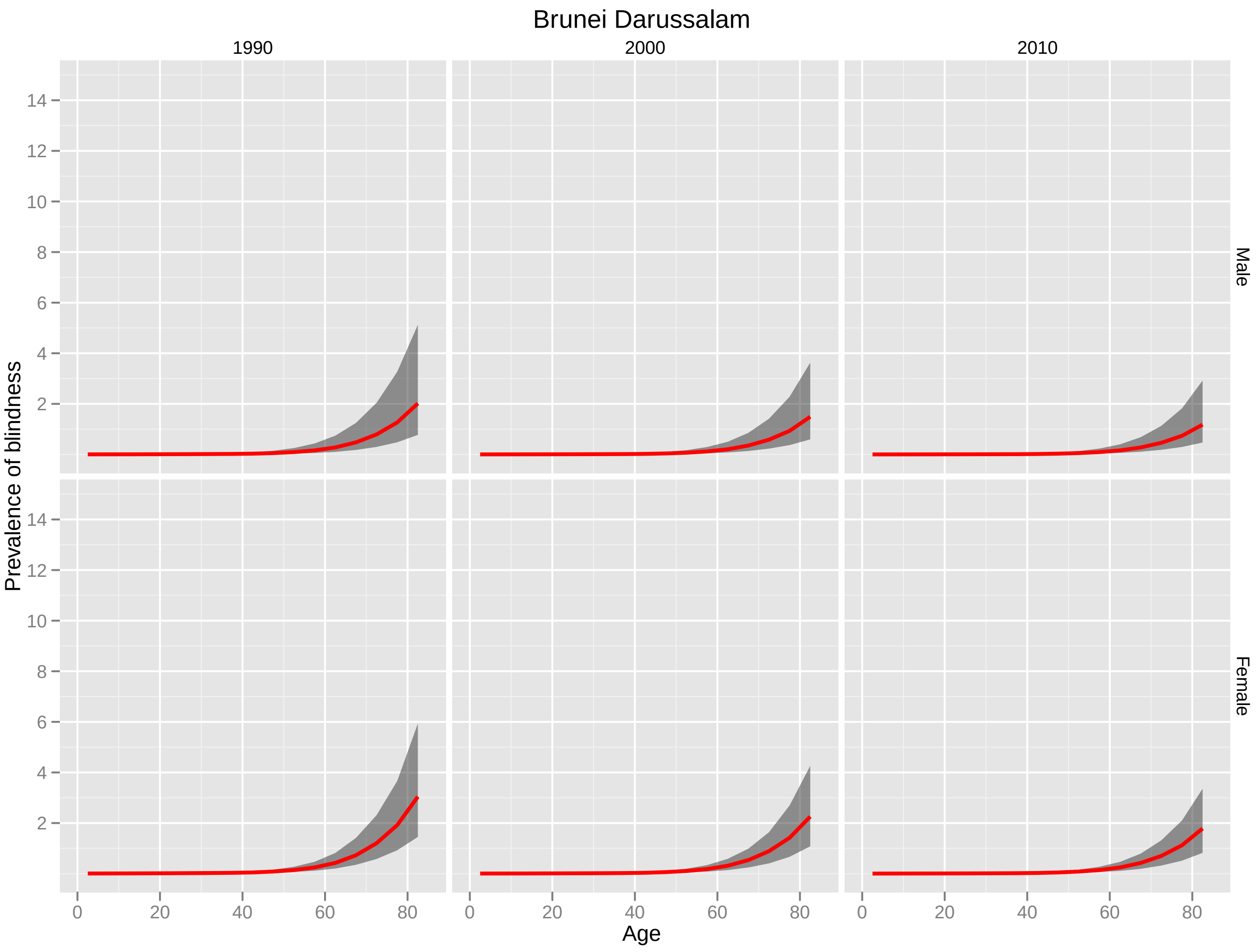

Brunei Darussalam
1990
2000
2010
Male
Female
Prevalence of blindness
Age
0
20
40
60
80
2
4
6
8
10
12
14

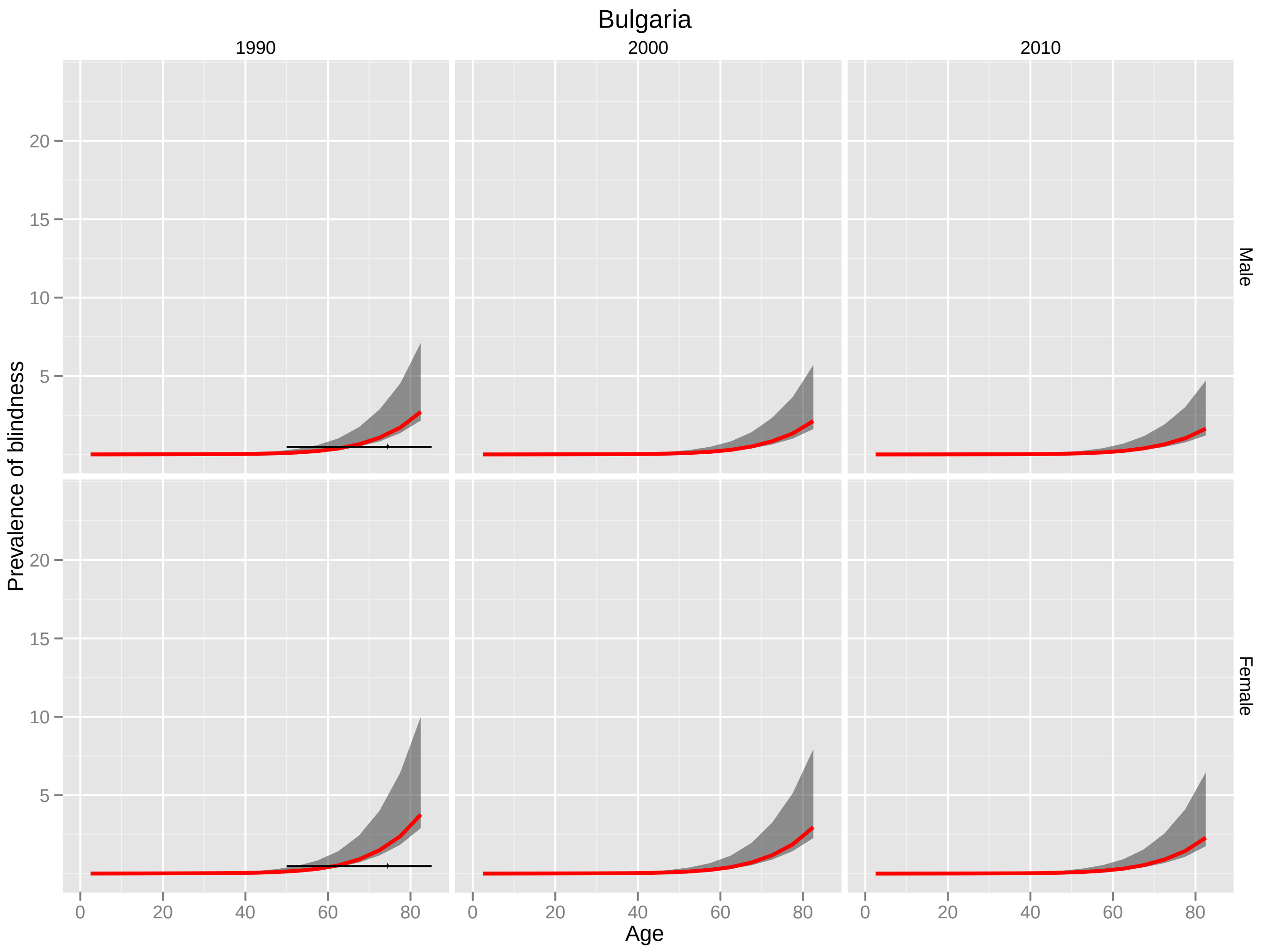
Bulgaria
1990
2000
2010
Male
Female
Prevalence of blindness
Age
0
5
10
15
20
40
60
80

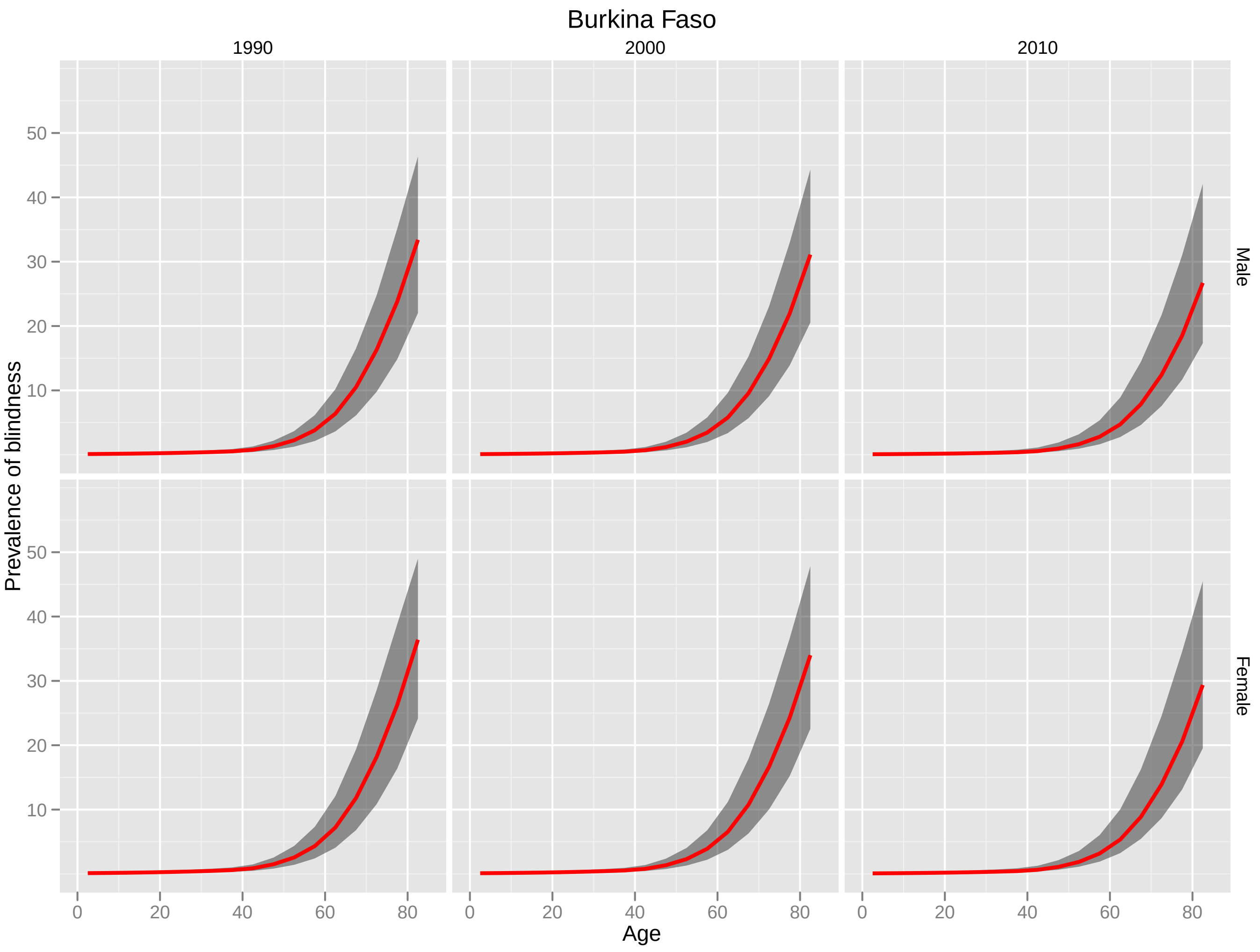
Burkina Faso
1990
2000
2010
Male
Female
Prevalence of blindness
Age
0
20
40
60
80
10
20
30
40
50

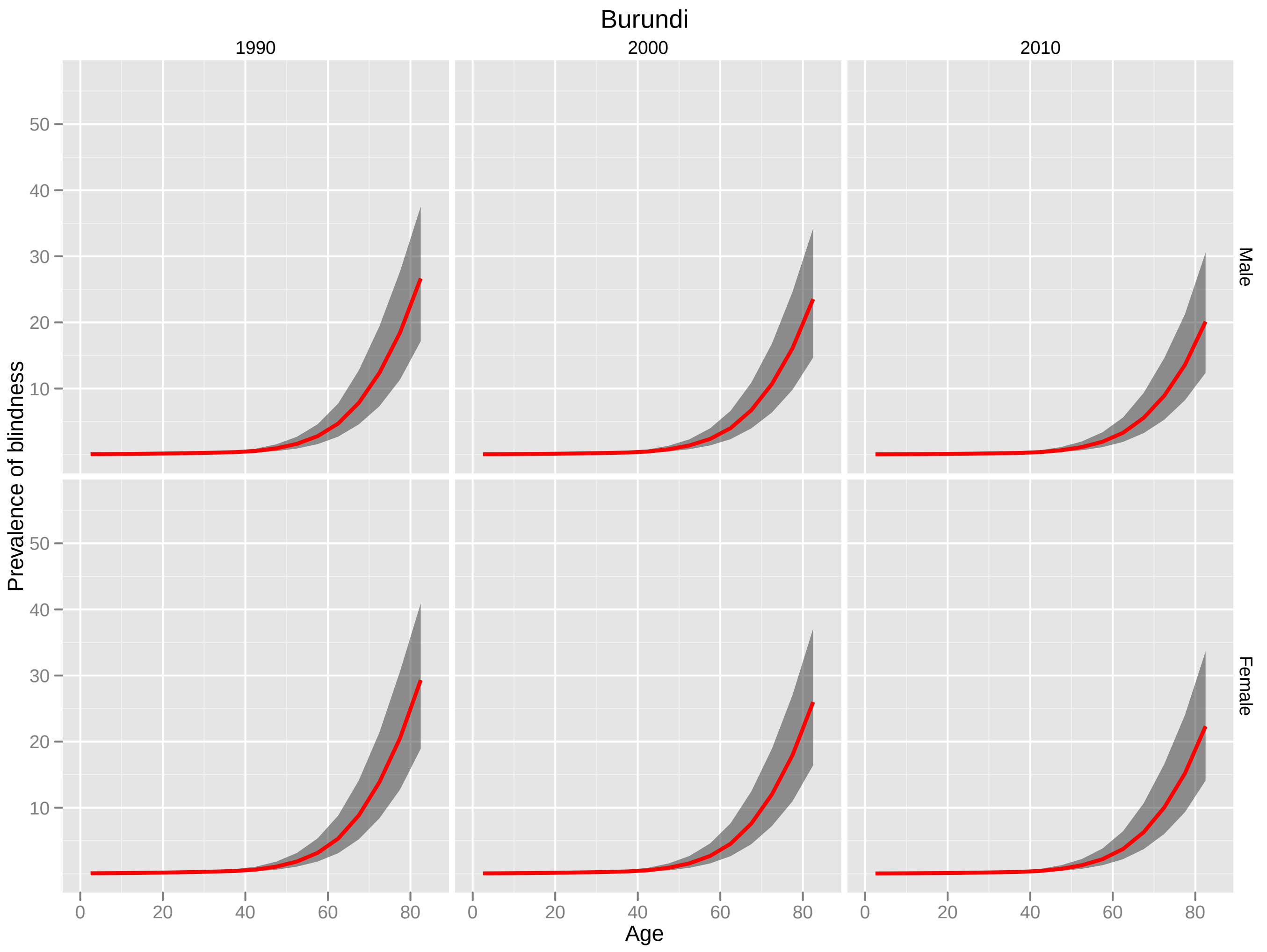
Burundi
1990
2000
2010
Male
Female
Prevalence of blindness
Age
10
20
30
40
50
0
20
40
60
80

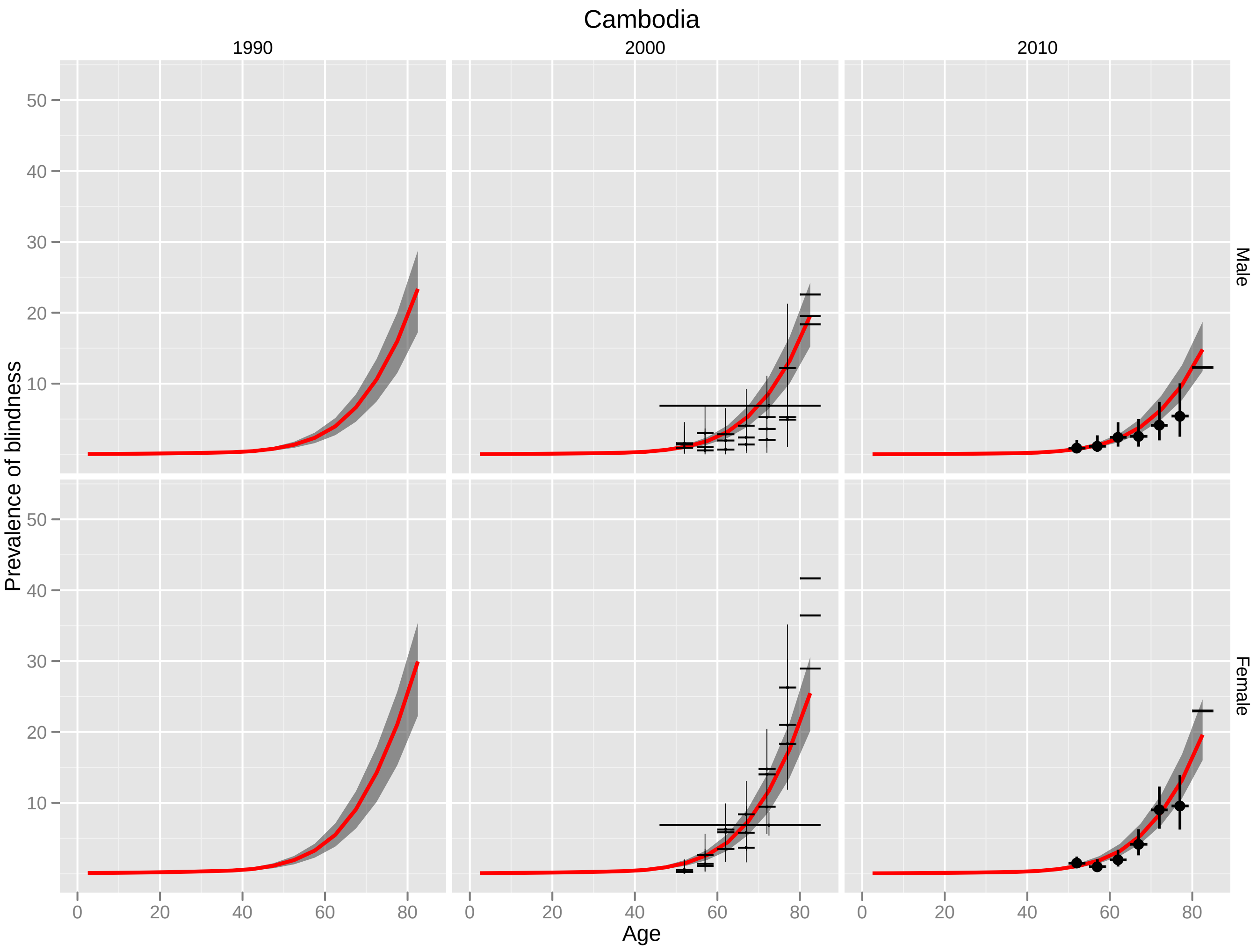
Cambodia
1990
2000
2010
Male
Female
Prevalence of blindness
Age
10
20
30
40
50
0
20
40
60
80

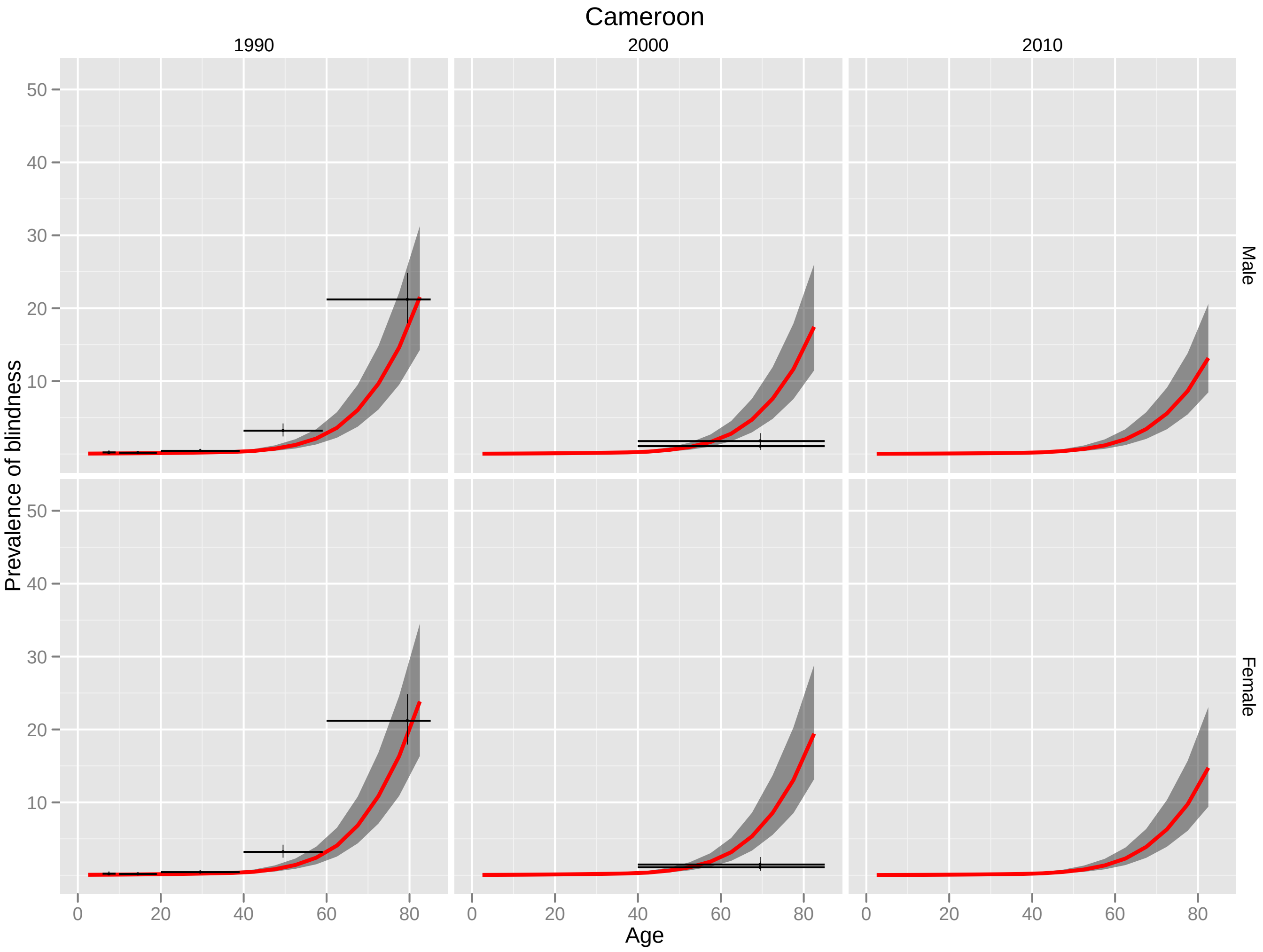
Cameroon
1990
2000
2010
Male
Female
Prevalence of blindness
Age
0
20
40
60
80
10
20
30
40
50

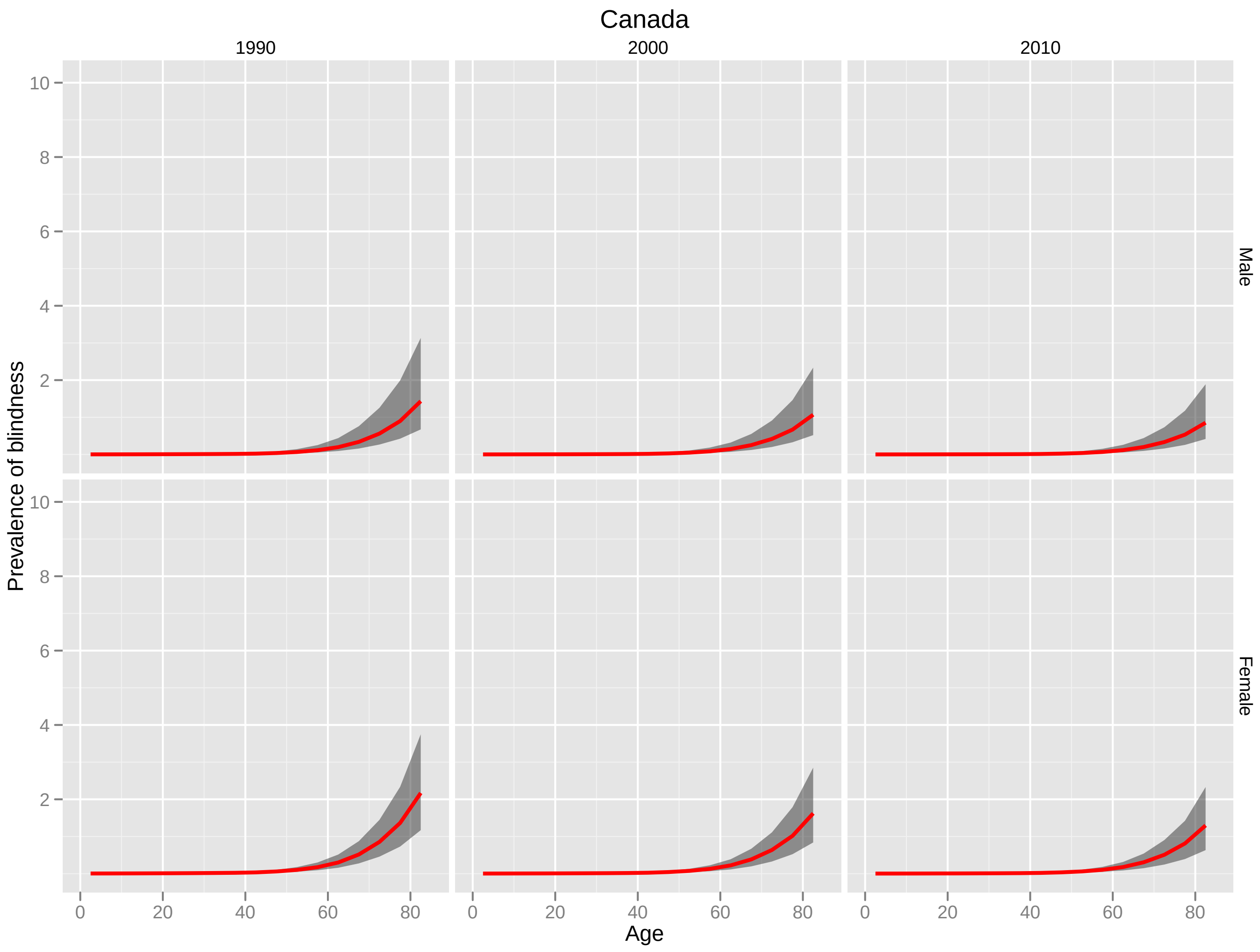
Canada
1990
2000
2010
Male
Female
Prevalence of blindness
Age
0
20
40
60
80
2
4
6
8
10

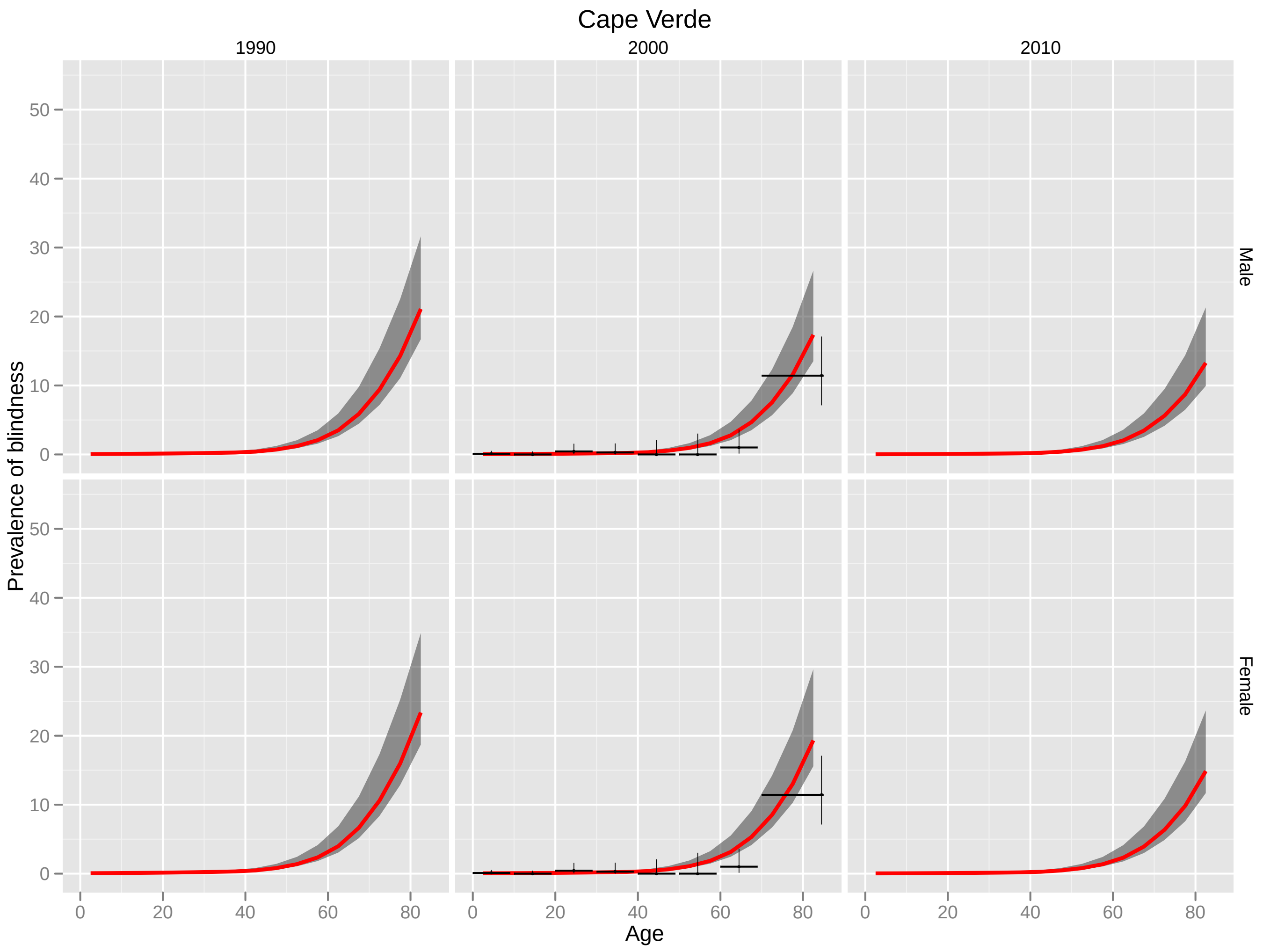
Cape Verde
1990
2000
2010
Male
Female
Prevalence of blindness
Age
0
10
20
30
40
50
0
20
40
60
80

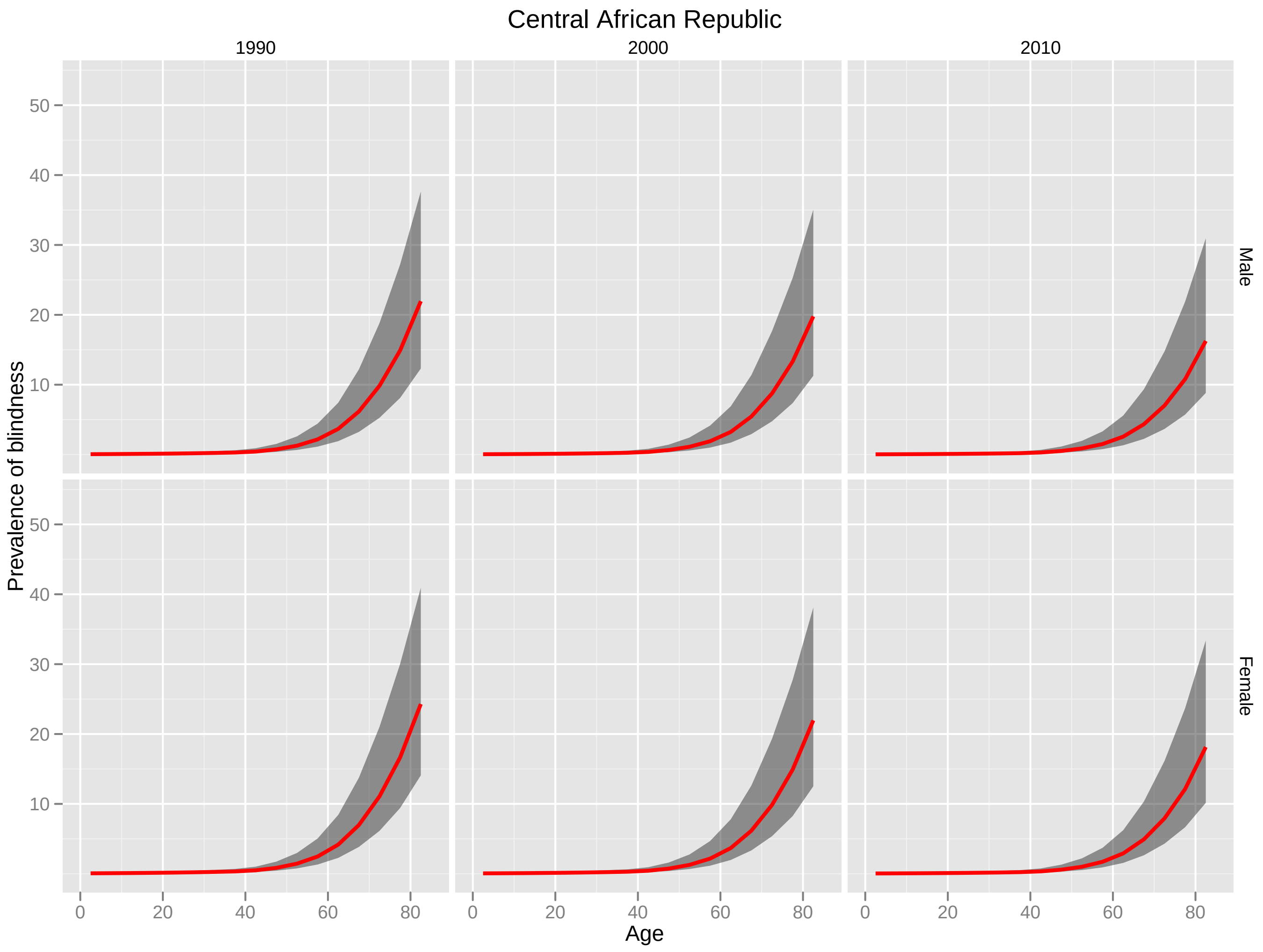
Central African Republic
1990
2000
2010
Male
Female
Prevalence of blindness
Age
10
20
30
40
50
0
20
40
60
80

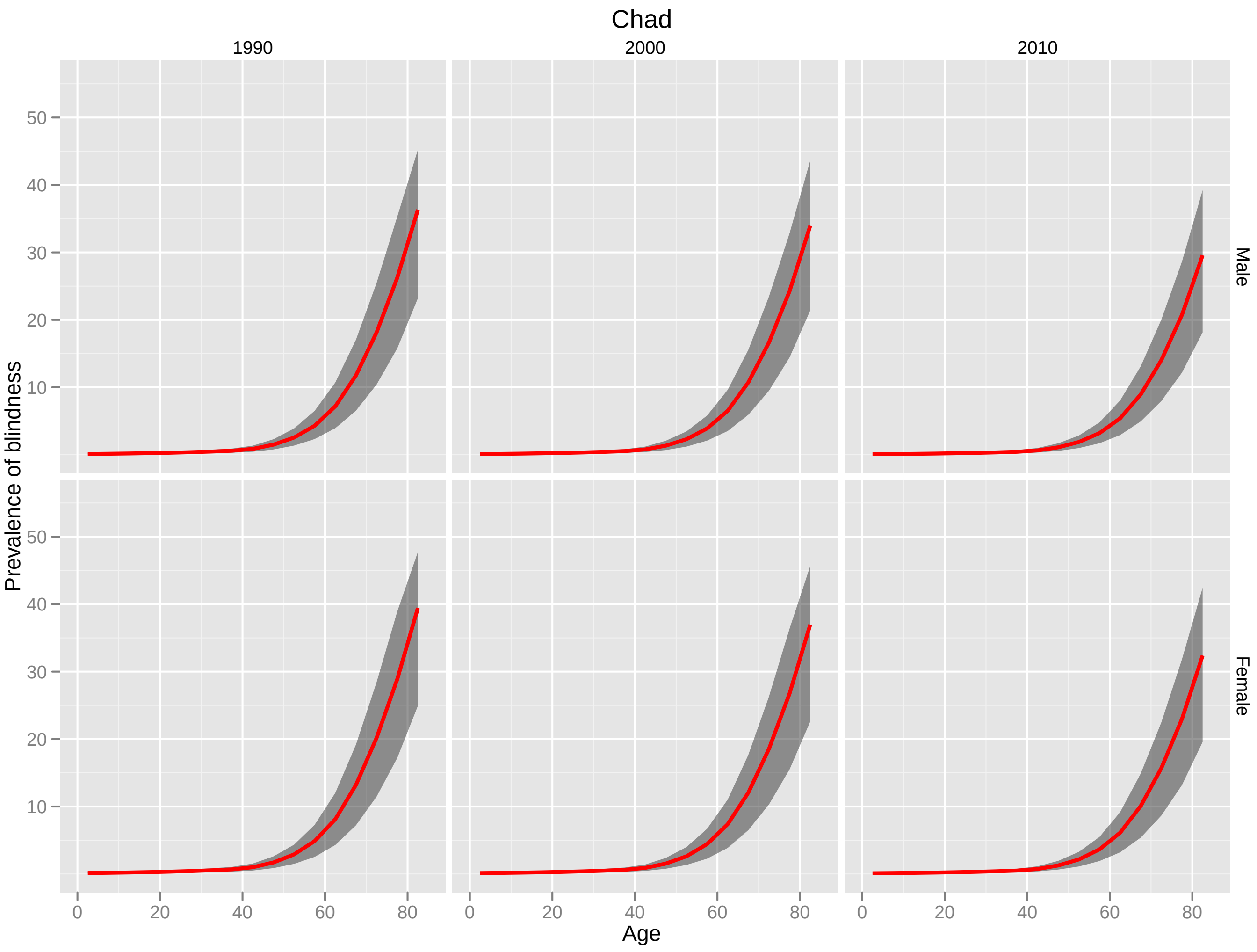
Chad
1990
2000
2010
Male
Female
Prevalence of blindness
10
20
30
40
50
0
20
40
60
80
Age

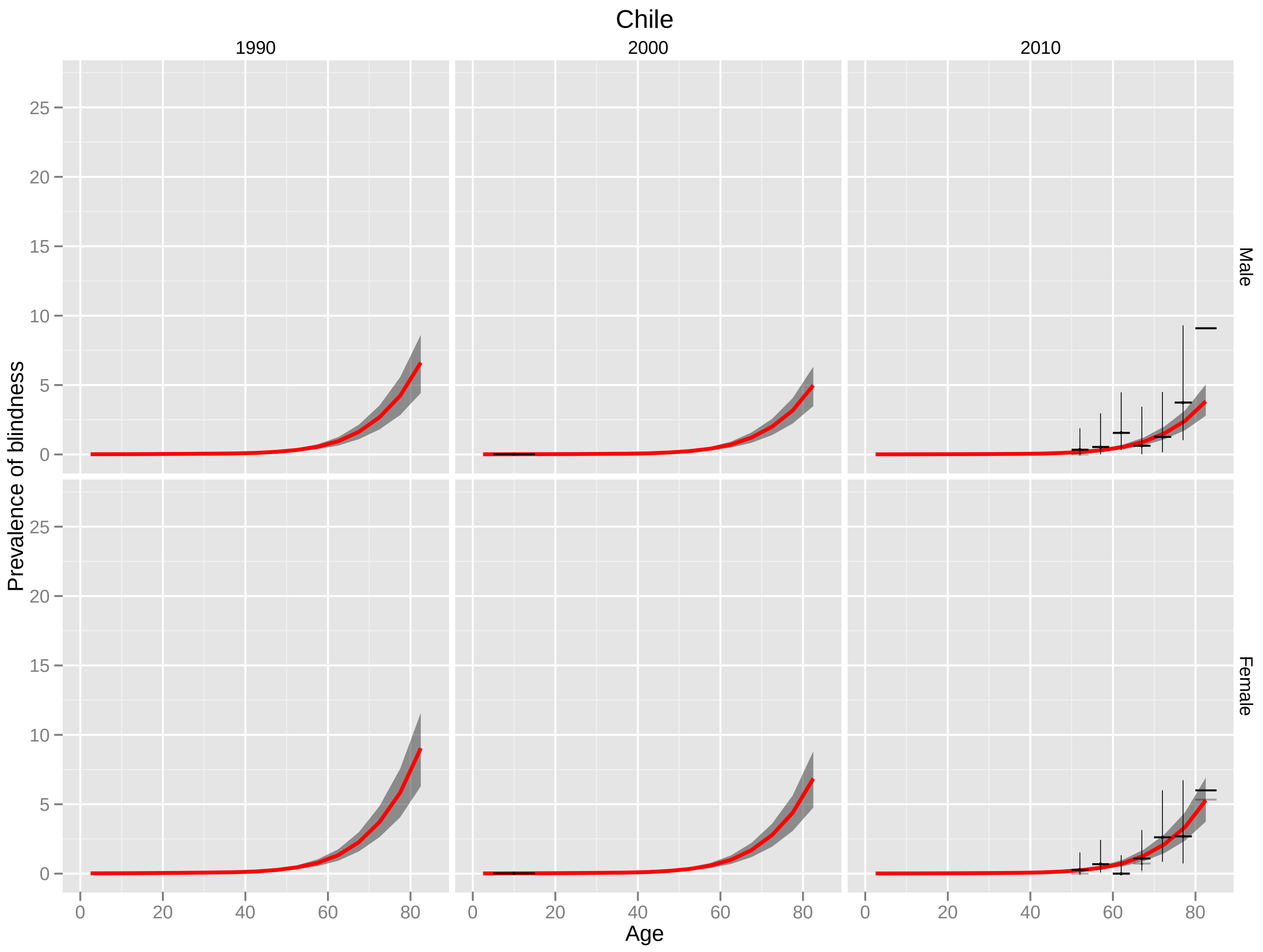
Chile
1990
2000
2010
Male
Female
Prevalence of blindness
Age
0
5
10
15
20
25
0
20
40
60
80

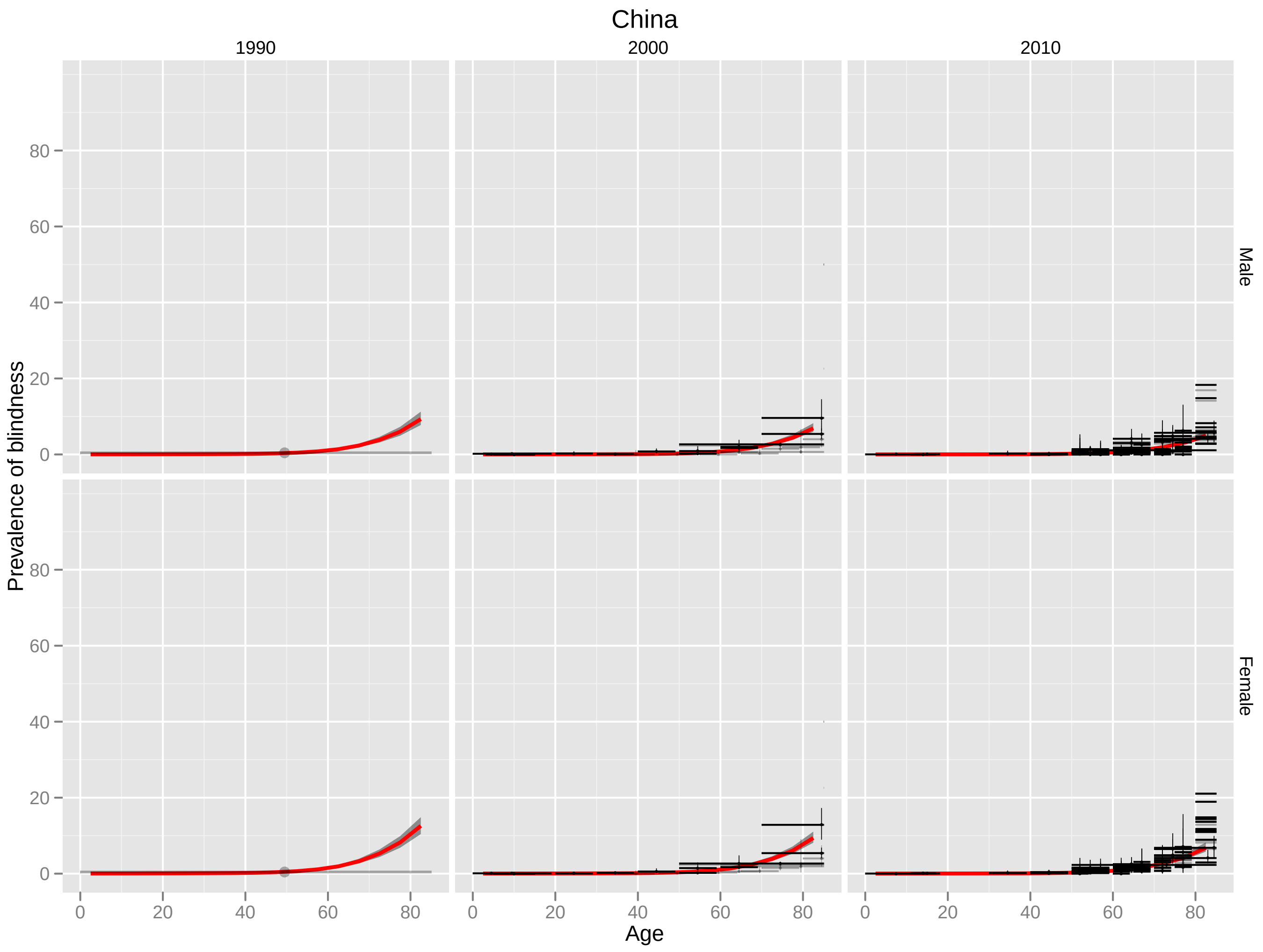
China
1990
2000
2010
Male
Female
Prevalence of blindness
Age
0
20
40
60
80

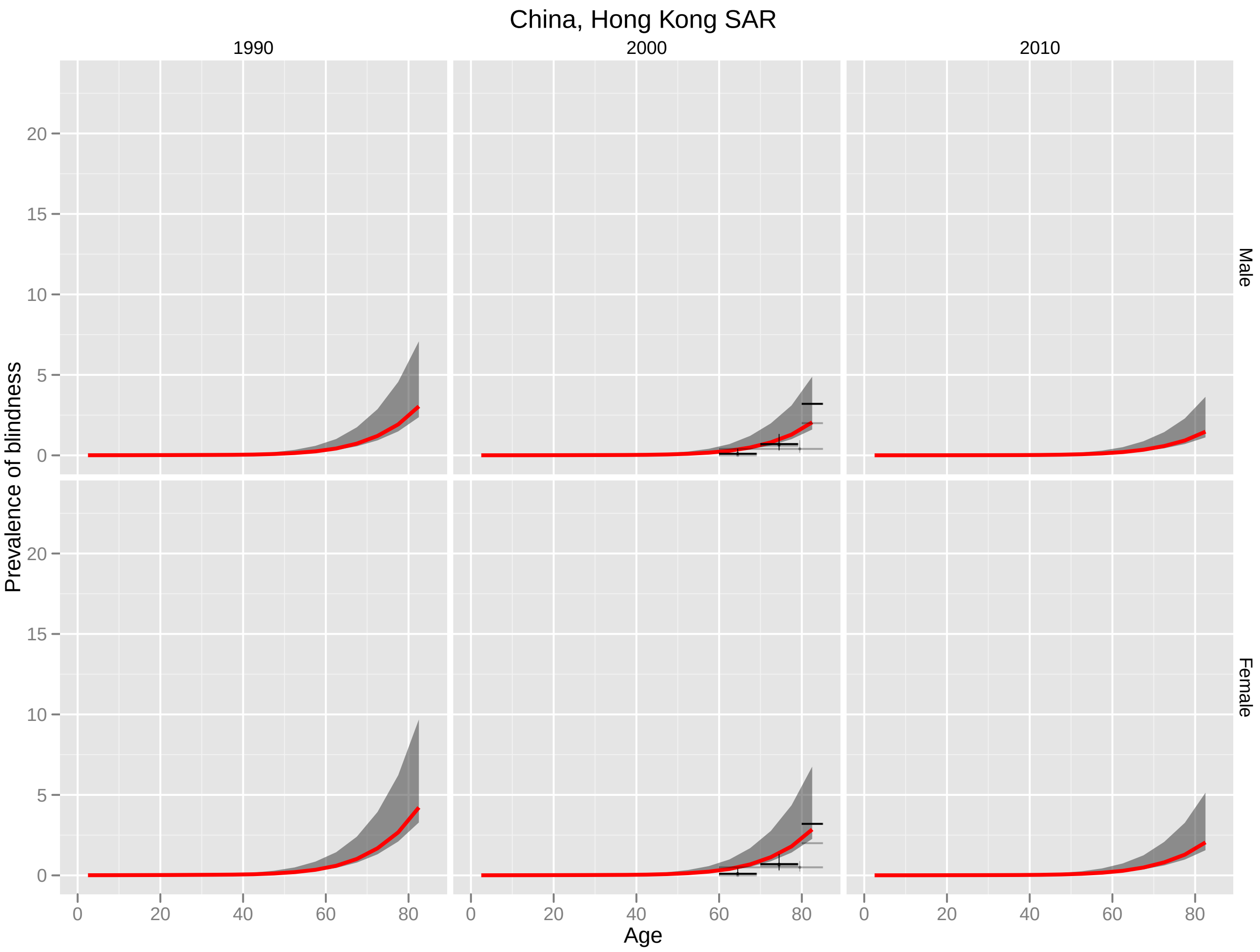
China, Hong Kong SAR
1990
2000
2010
Male
Female
Prevalence of blindness
Age
0
5
10
15
20
0
20
40
60
80

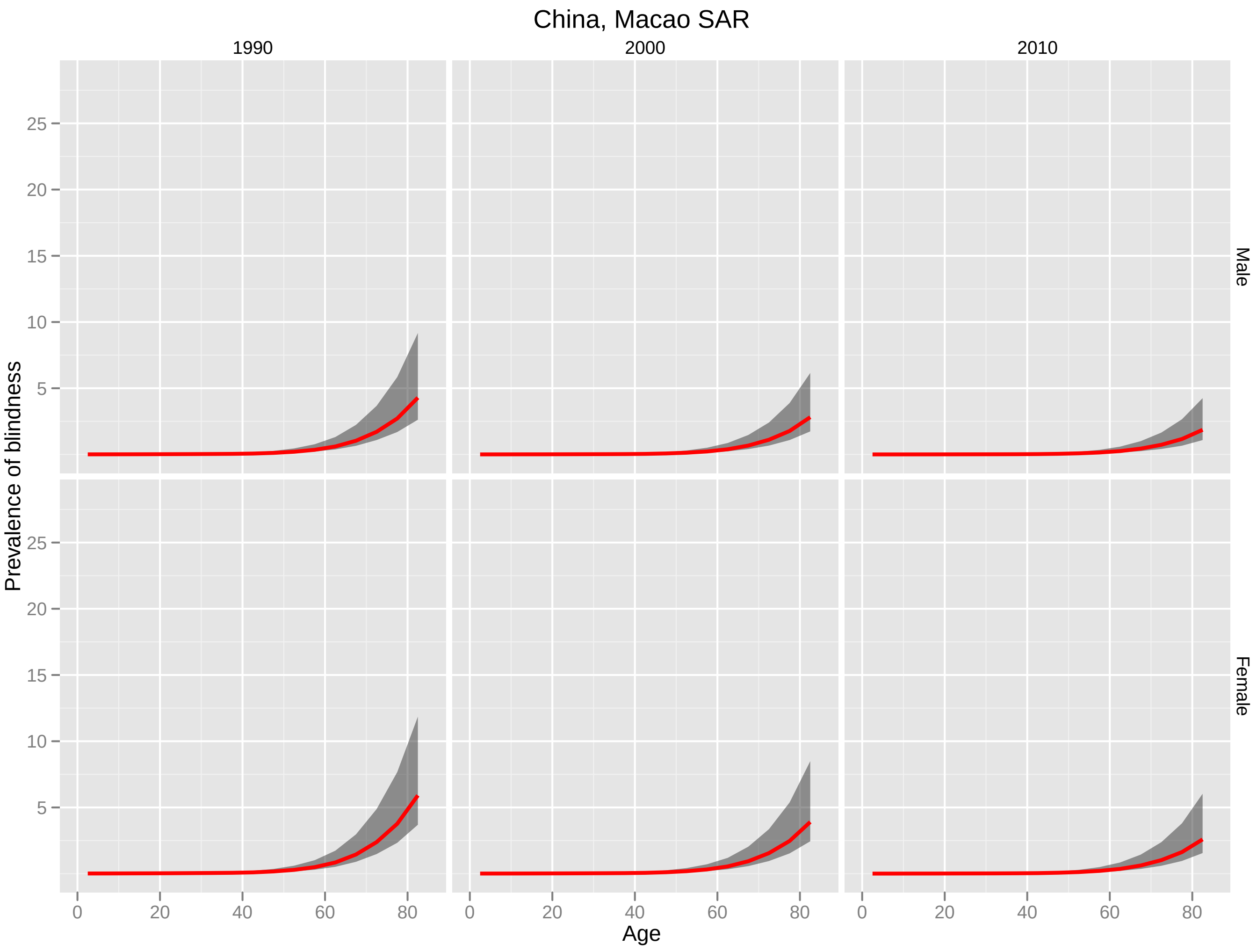
China, Macao SAR
1990
2000
2010
Male
Female
Prevalence of blindness
Age
0
20
40
60
80
5
10
15
20
25

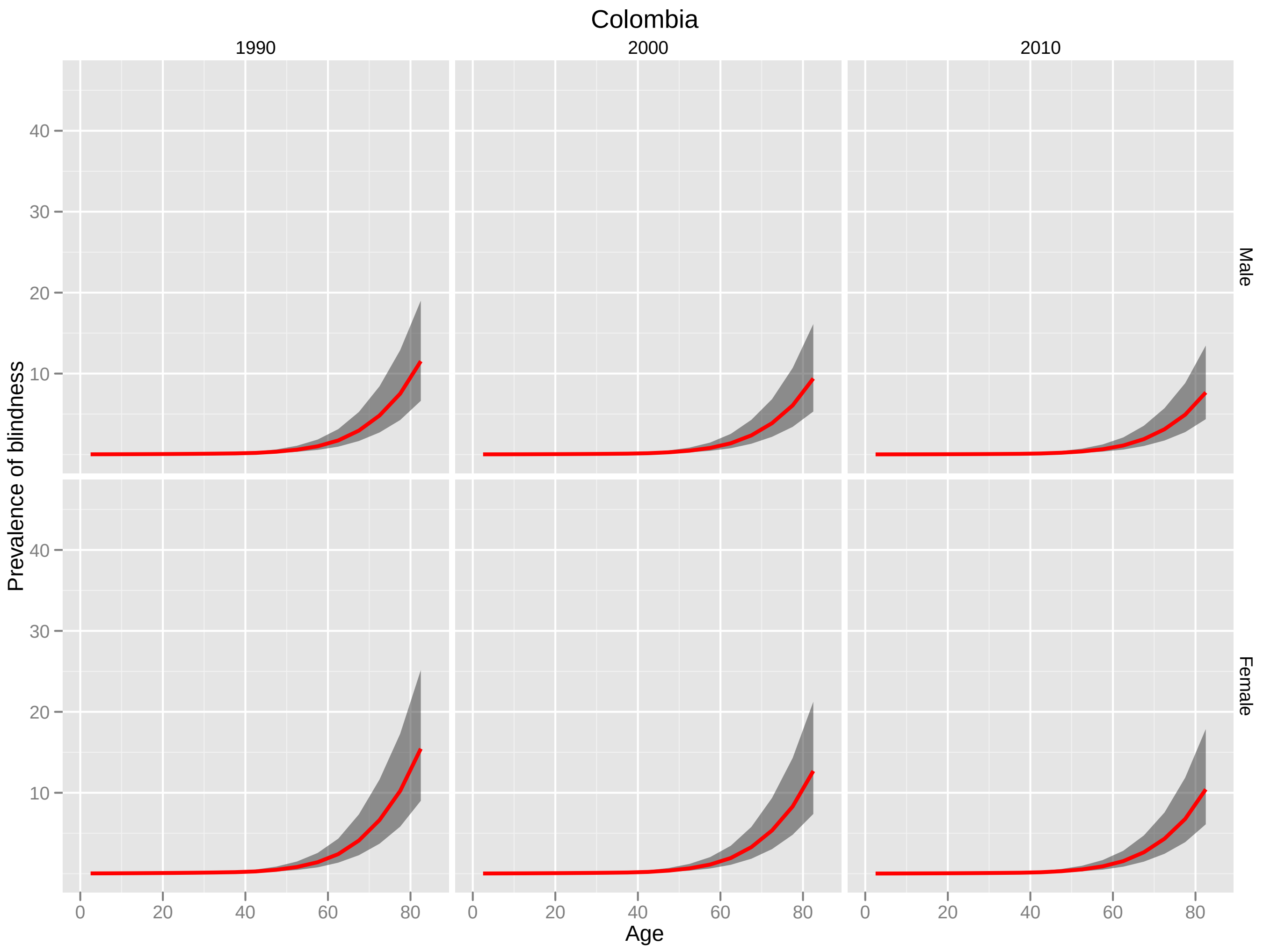

Colombia
1990
2000
2010
Male
Female
Prevalence of blindness
Age
10
20
30
40
0
20
40
60
80

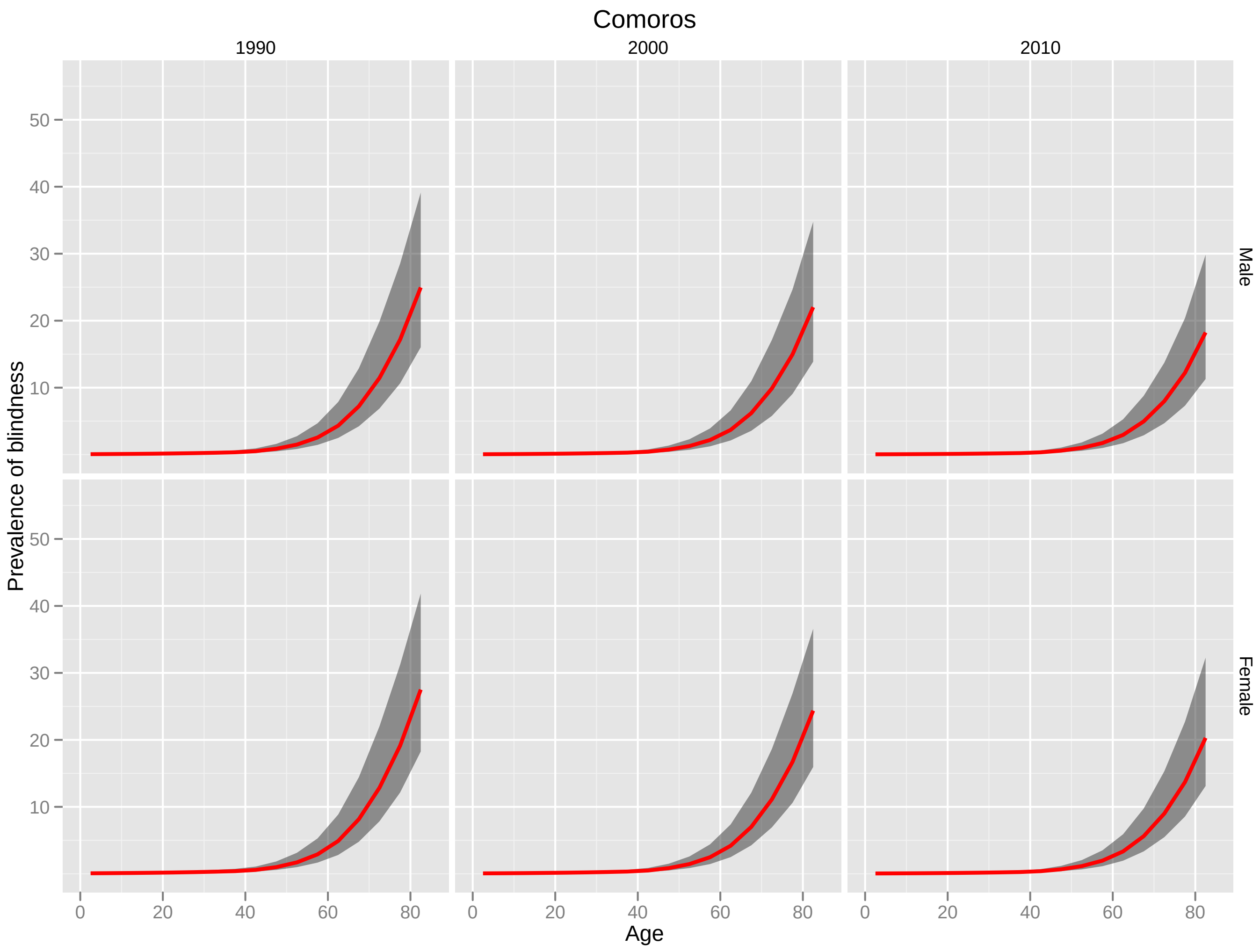
Comoros
1990
2000
2010
Male
Female
Prevalence of blindness
10
20
30
40
50
Age
0
20
40
60
80

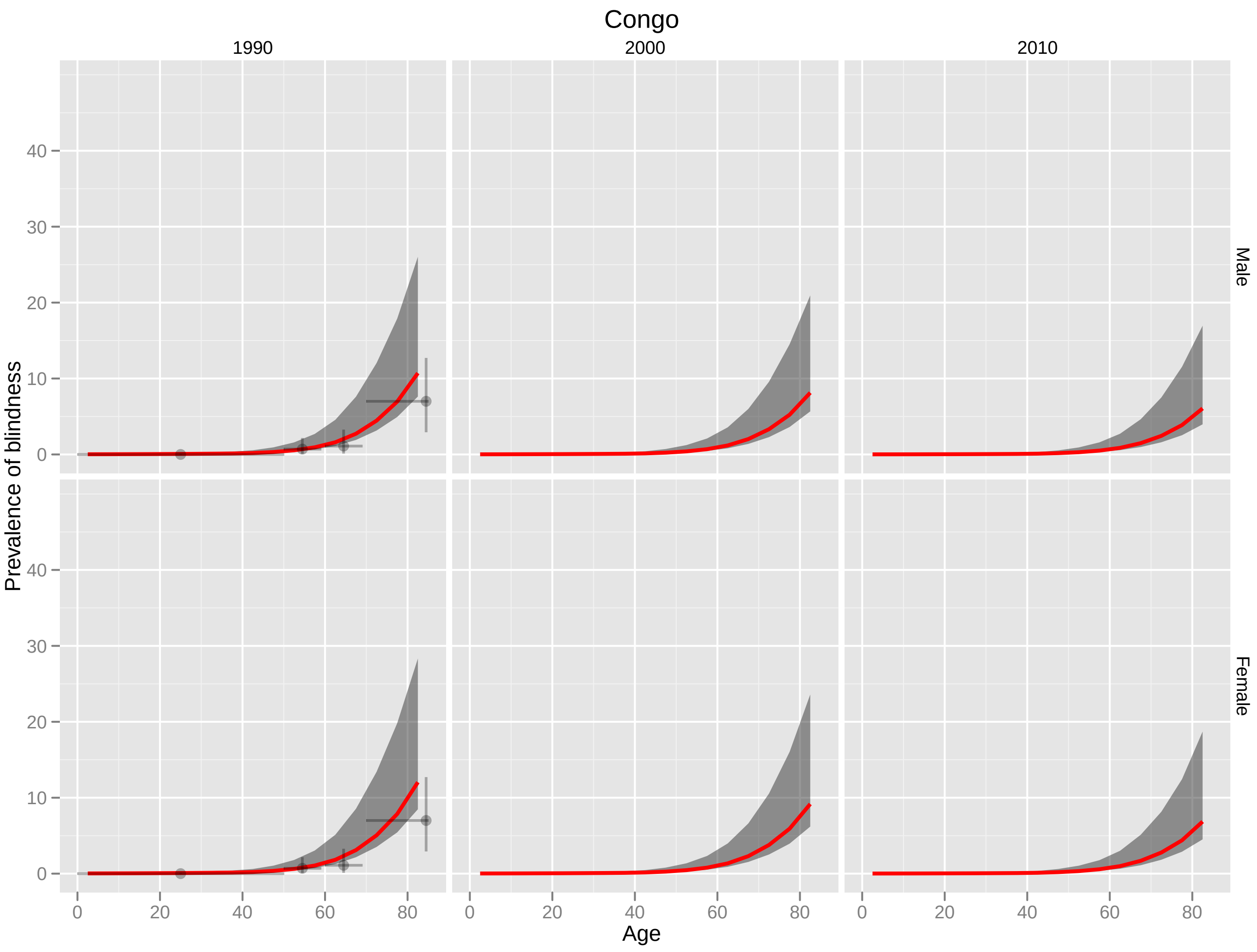
Congo
1990
2000
2010
Male
Female
Prevalence of blindness
Age
0
10
20
30
40
60
80

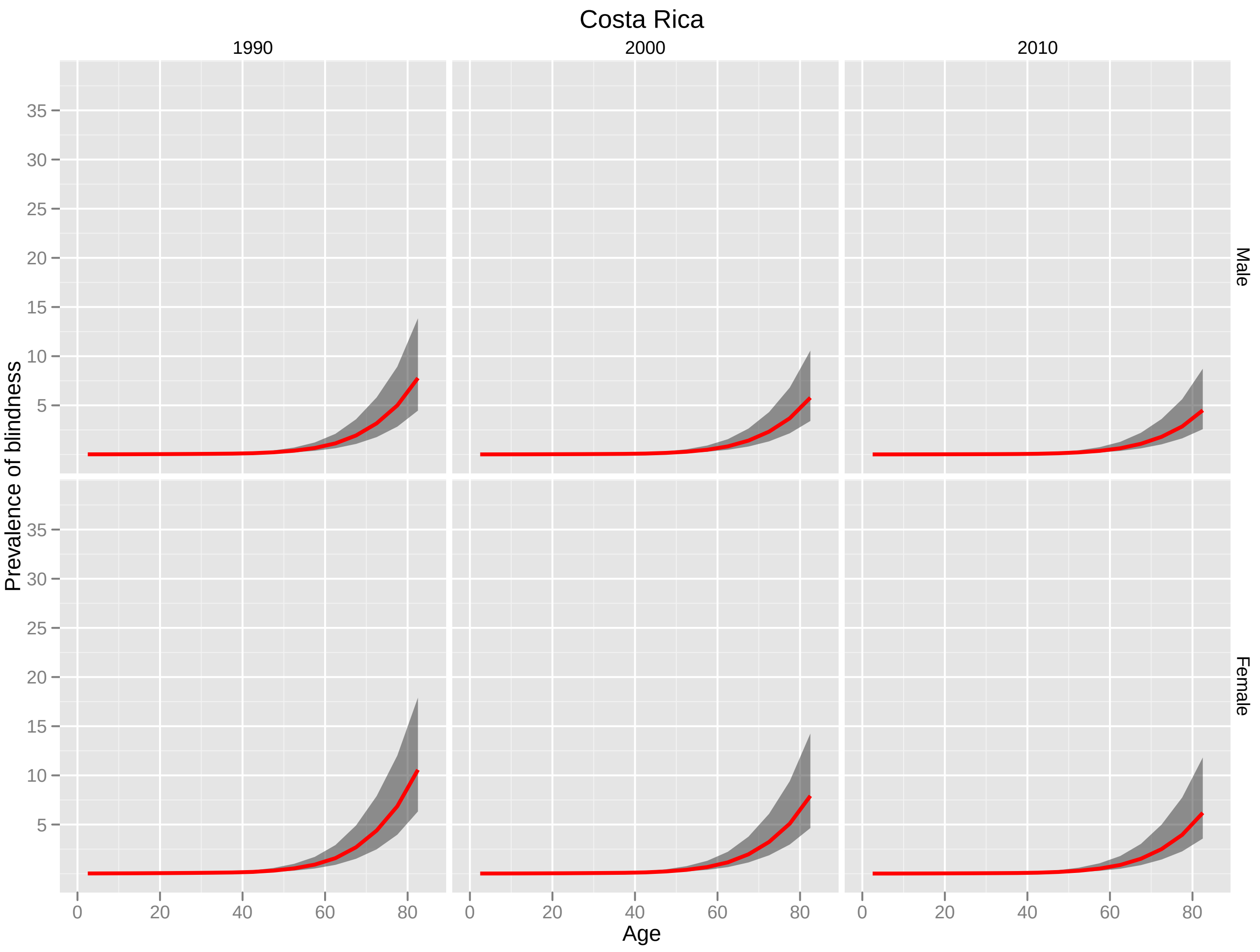
Costa Rica
1990
2000
2010
Male
Female
Prevalence of blindness
35
30
25
20
15
10
5
0
20
40
60
80
Age

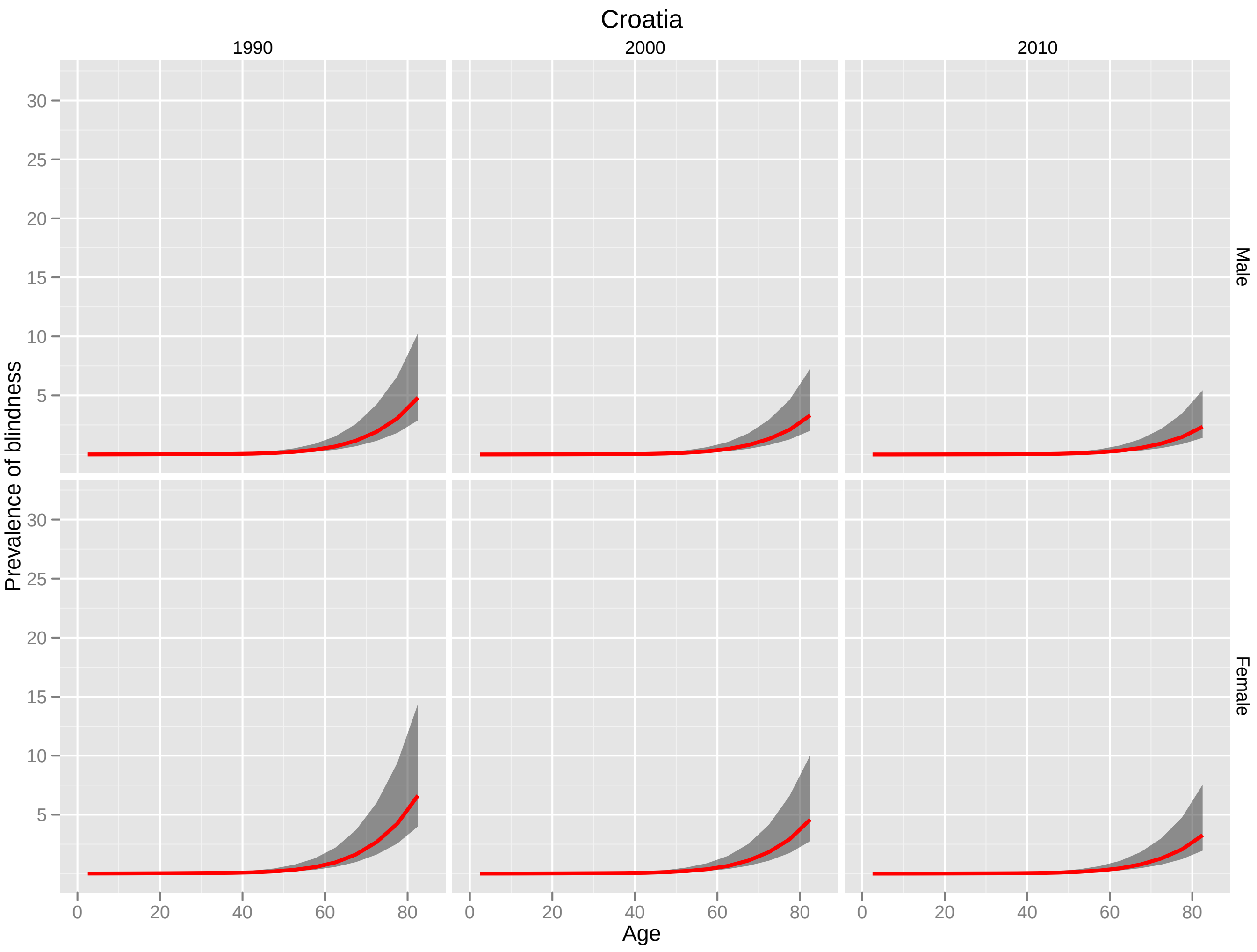
Croatia
1990
2000
2010
Male
Female
Prevalence of blindness
Age
30
25
20
15
10
5
0
20
40
60
80

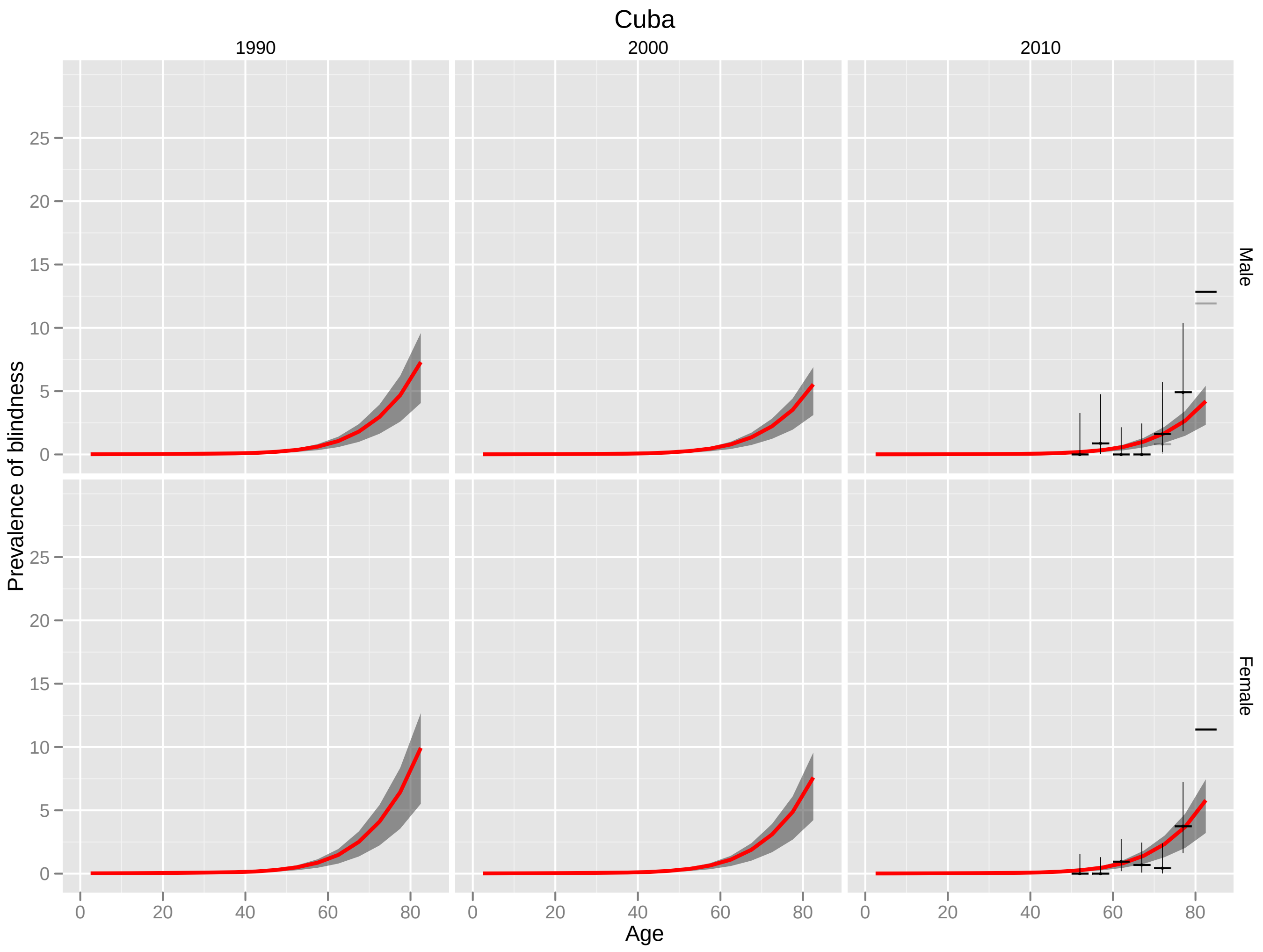
Cuba
1990
2000
2010
Male
Female
Prevalence of blindness
Age
0
5
10
15
20
25
0
20
40
60
80

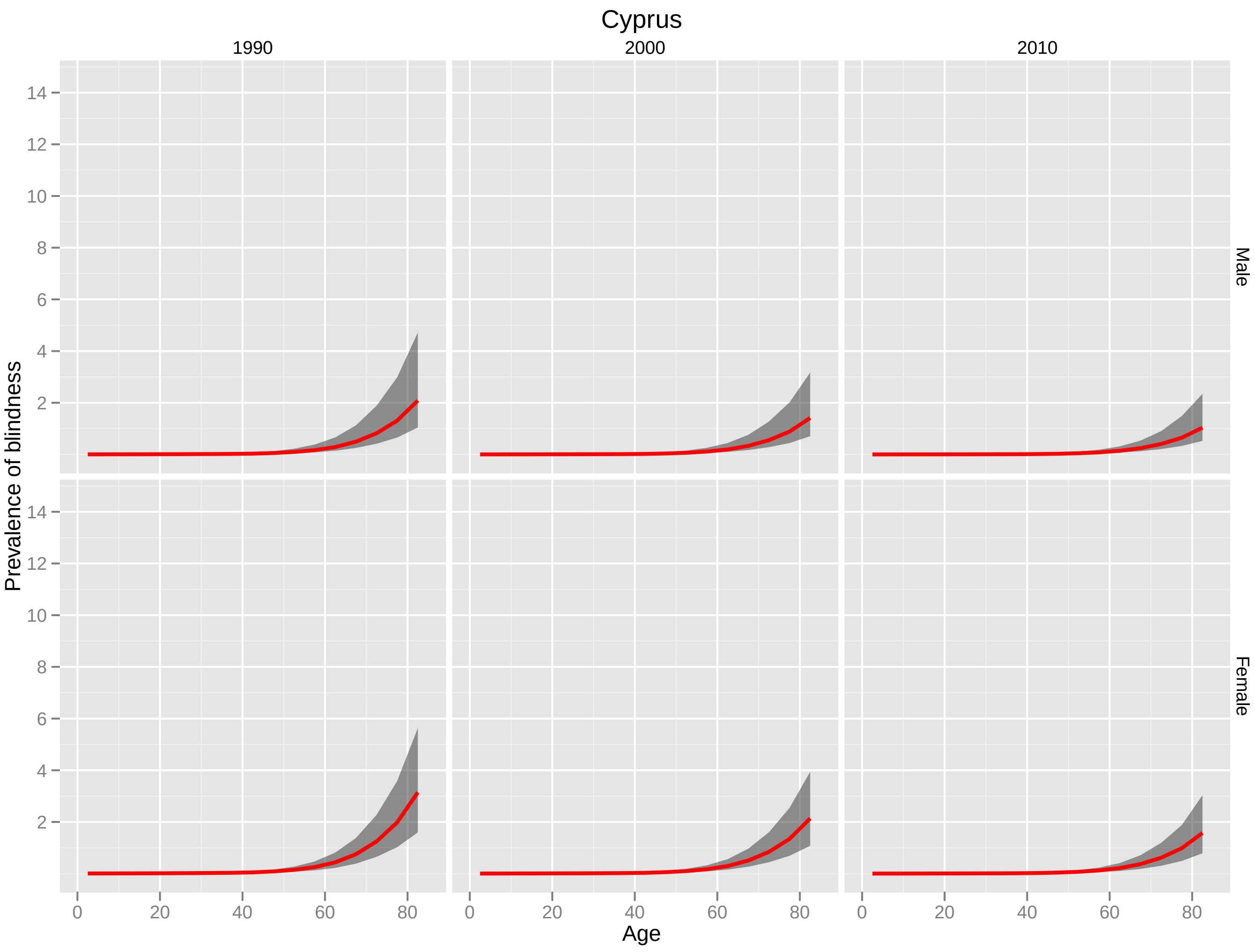
Cyprus
1990
2000
2010
Male
Female
Prevalence of blindness
Age
14
12
10
8
6
4
2
0
20
40
60
80

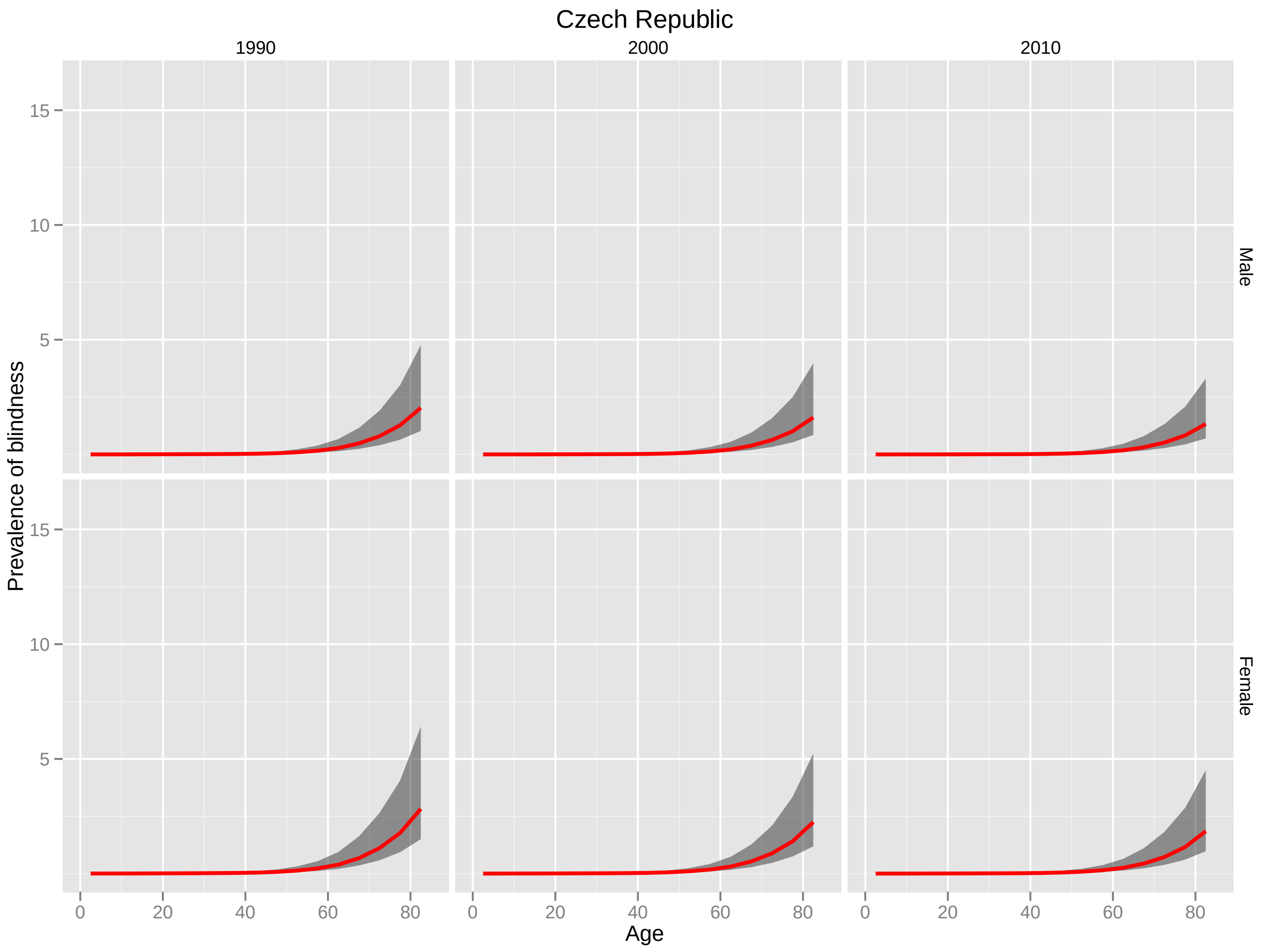
Czech Republic
1990
2000
2010
Male
Female
Prevalence of blindness
Age
0
20
40
60
80
5
10
15

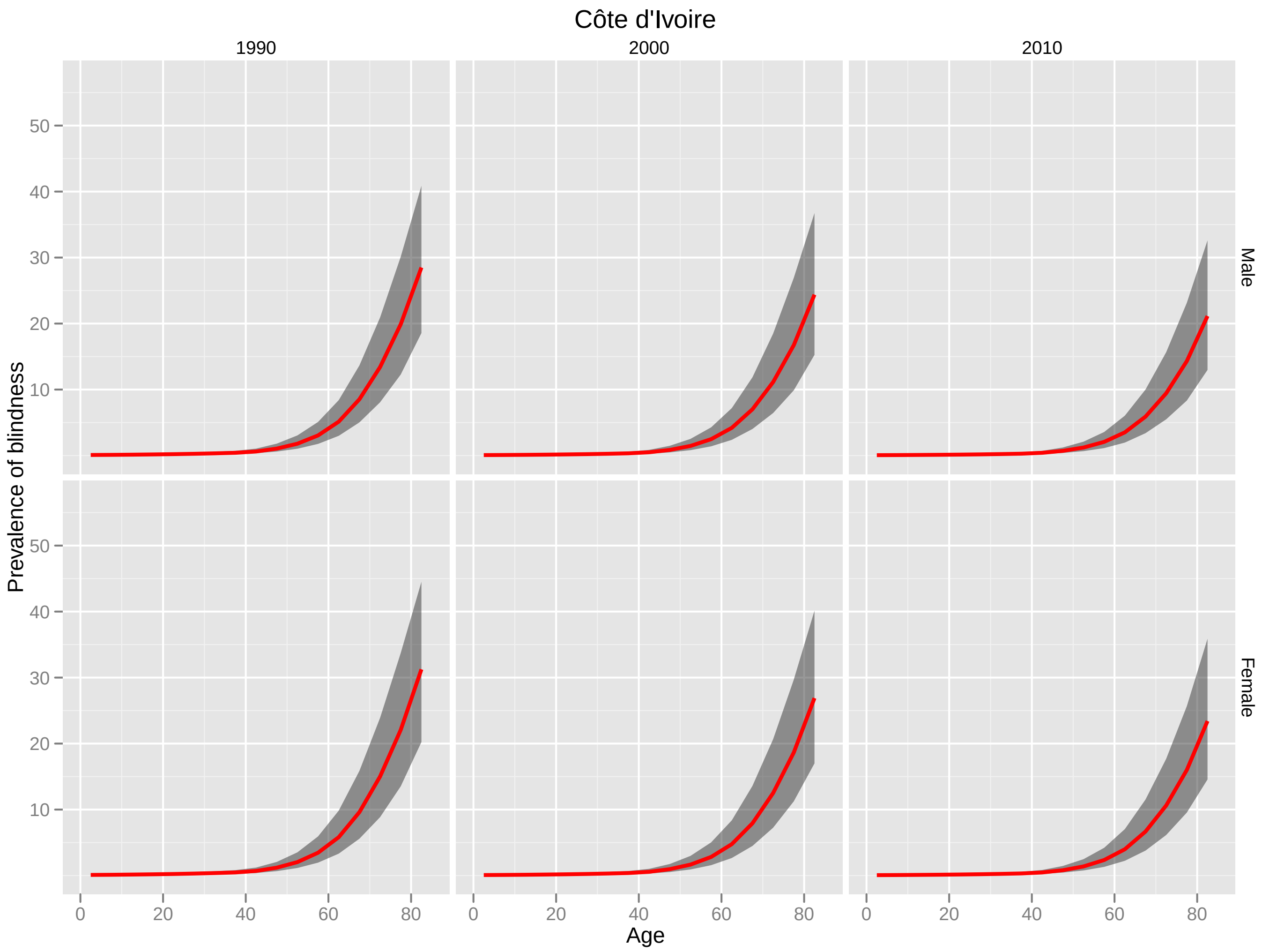
Côte d'Ivoire
1990
2000
2010
Male
Female
Prevalence of blindness
Age
10
20
30
40
50
0
20
40
60
80

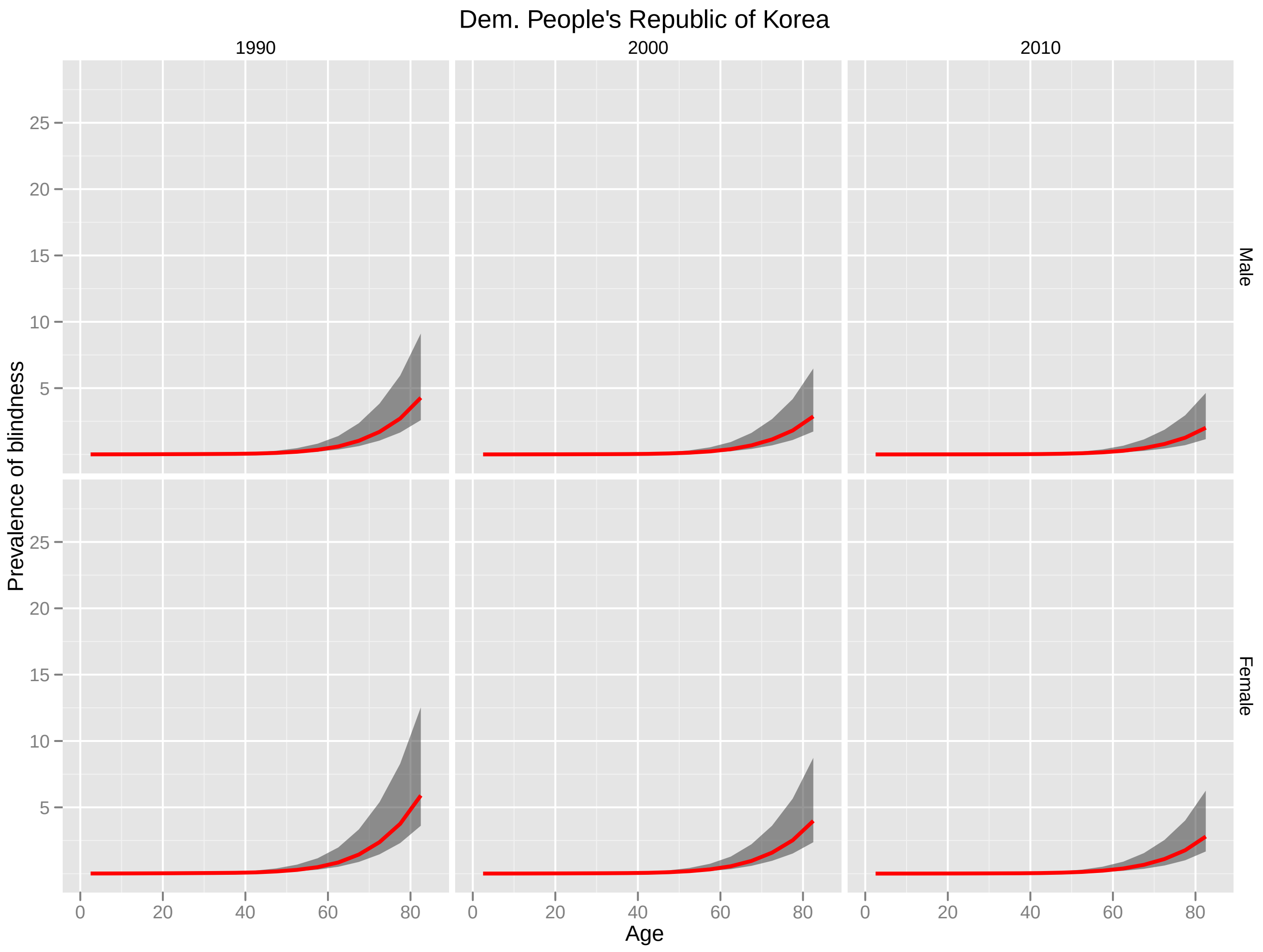

Dem. People's Republic of Korea
1990
2000
2010
Male
Female
Prevalence of blindness
Age
0
20
40
60
80
5
10
15
20
25

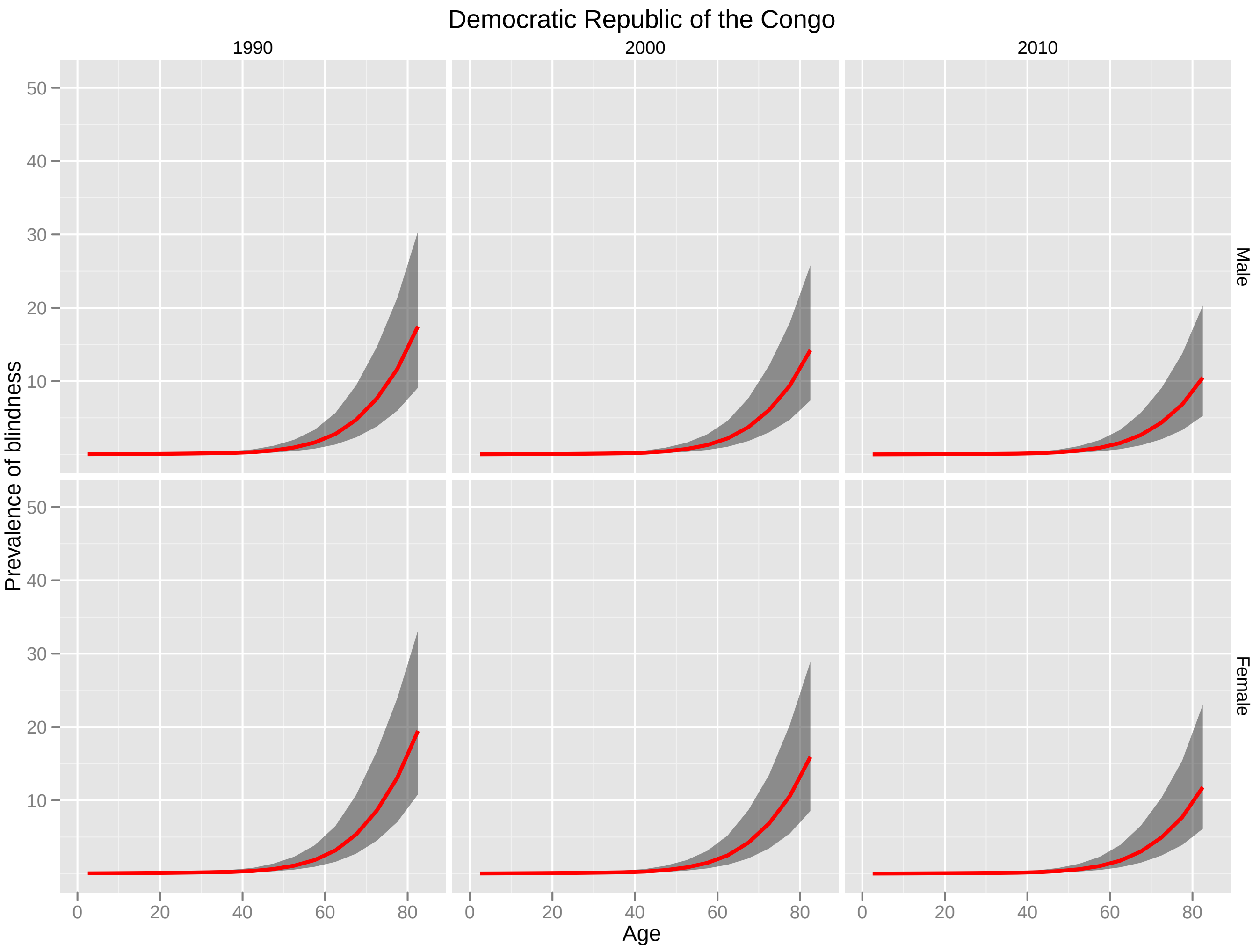
Democratic Republic of the Congo
1990
2000
2010
Male
Female
Prevalence of blindness
Age
10
20
30
40
50
0
20
40
60
80

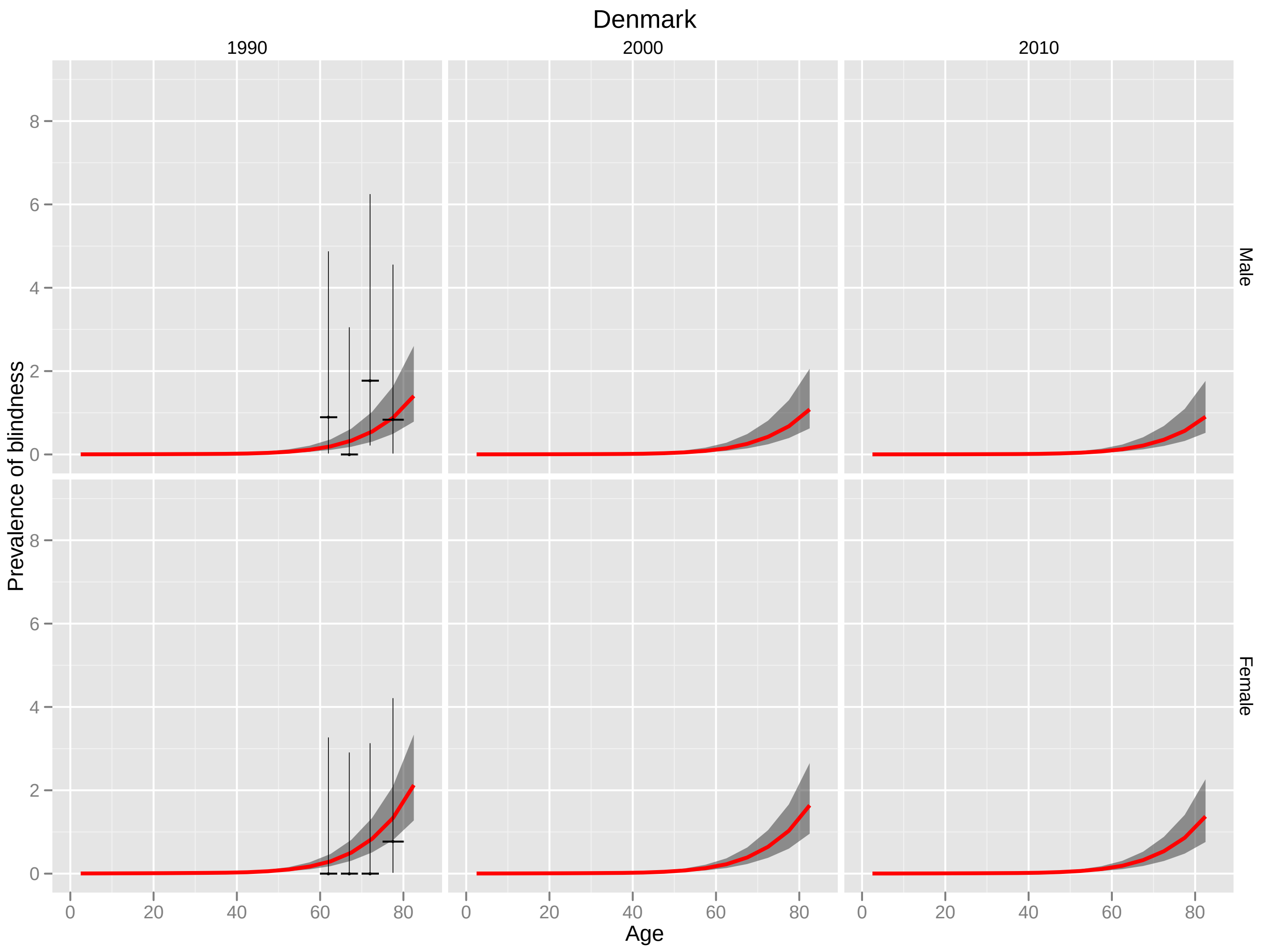
Denmark
1990
2000
2010
Male
Female
Prevalence of blindness
Age
0
2
4
6
8
0
20
40
60
80

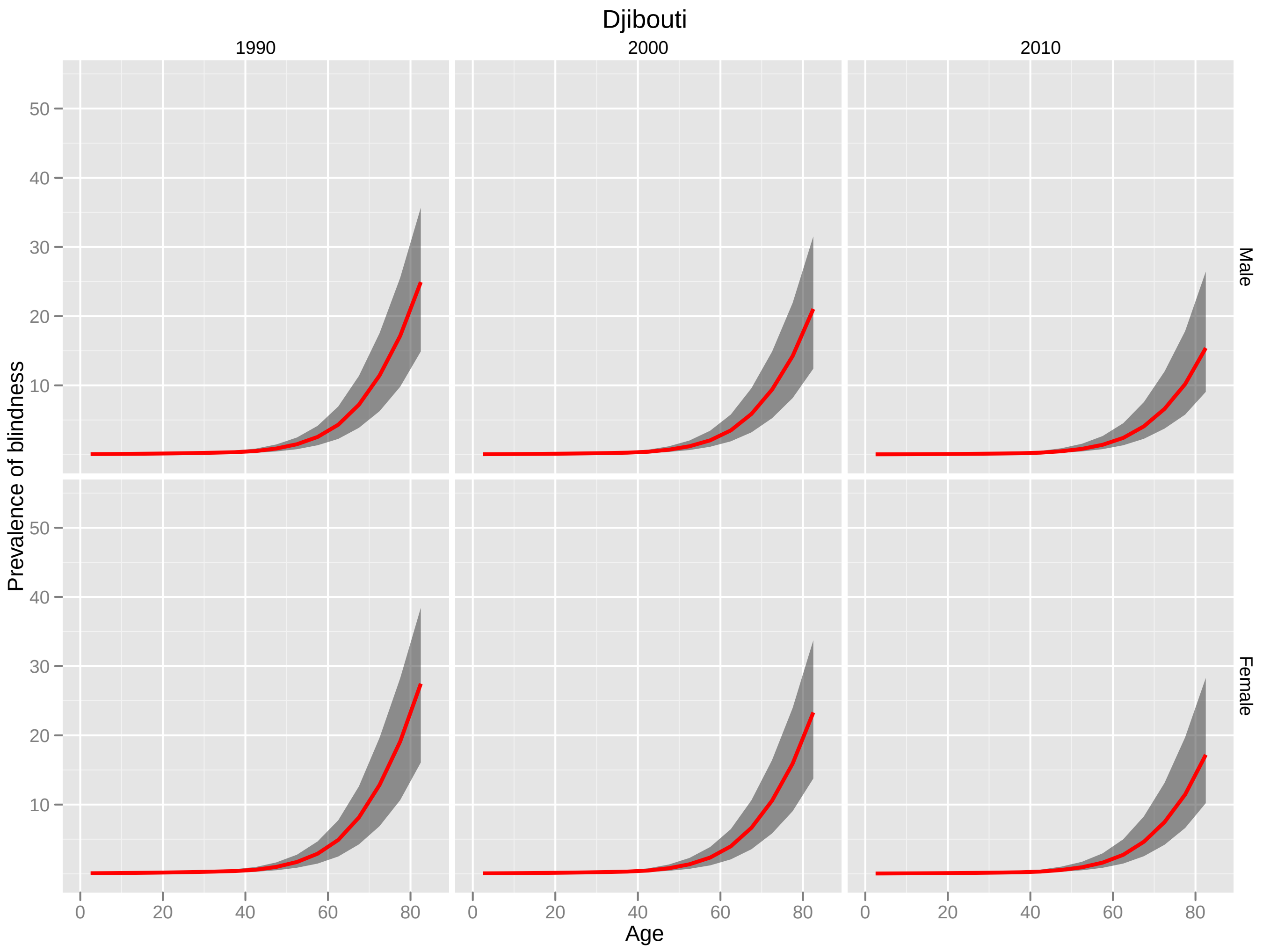
Djibouti
1990
2000
2010
Male
Female
Prevalence of blindness
Age
10
20
30
40
50
0
20
40
60
80

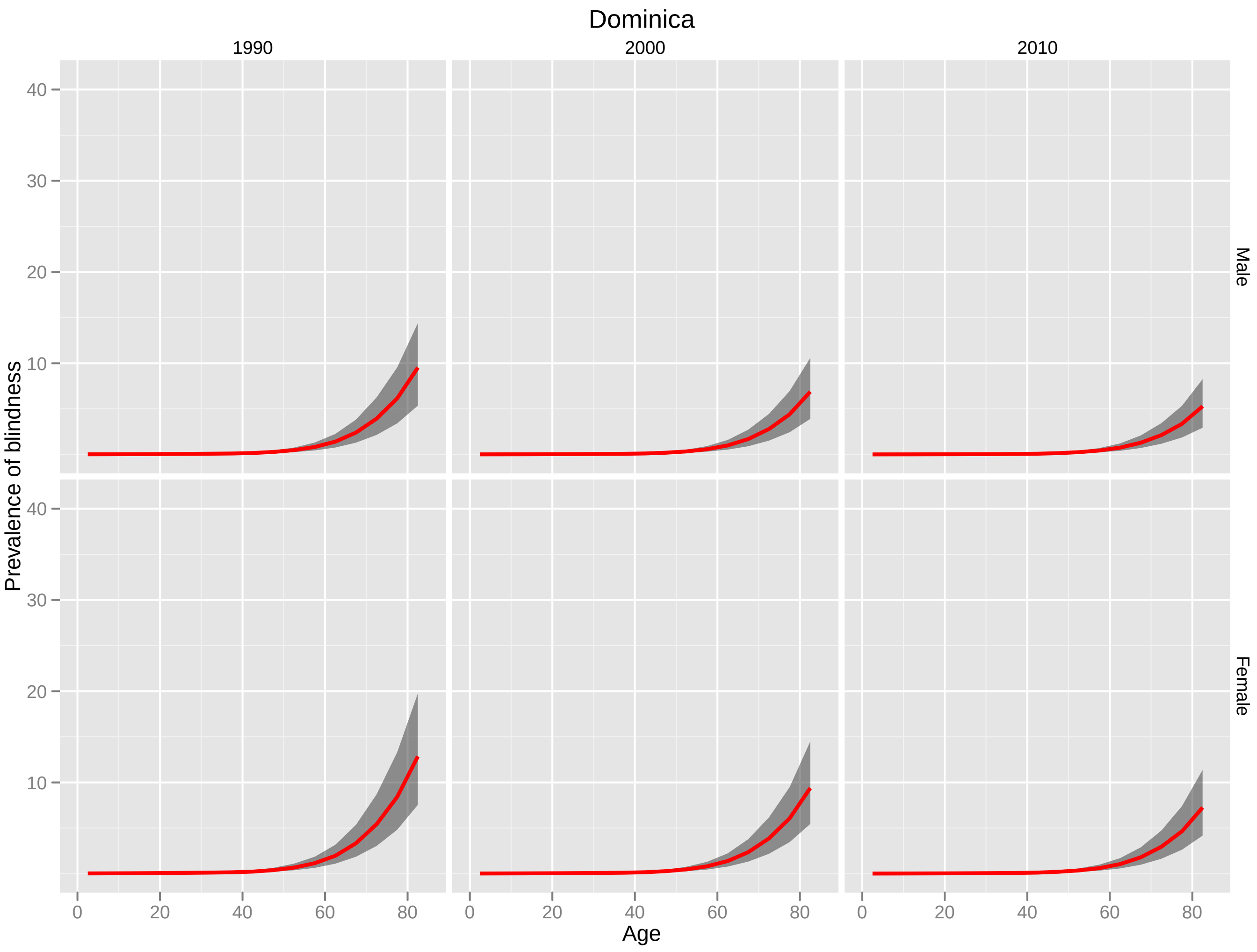
Dominica
1990
2000
2010
Male
Female
Prevalence of blindness
Age
0
10
20
30
40
60
80

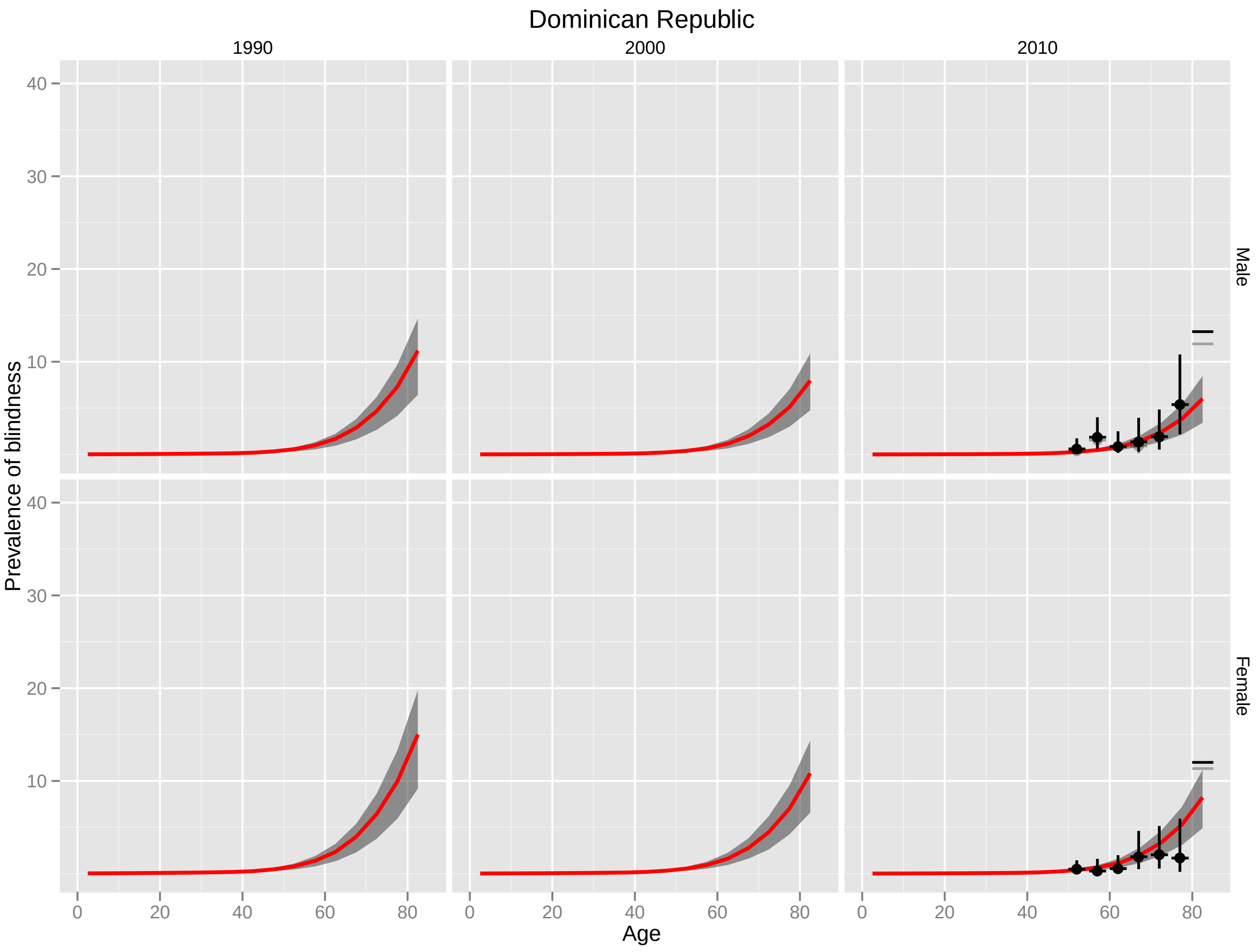

Dominican Republic
1990
2000
2010
Male
Female
Prevalence of blindness
Age
0
10
20
30
40
60
80

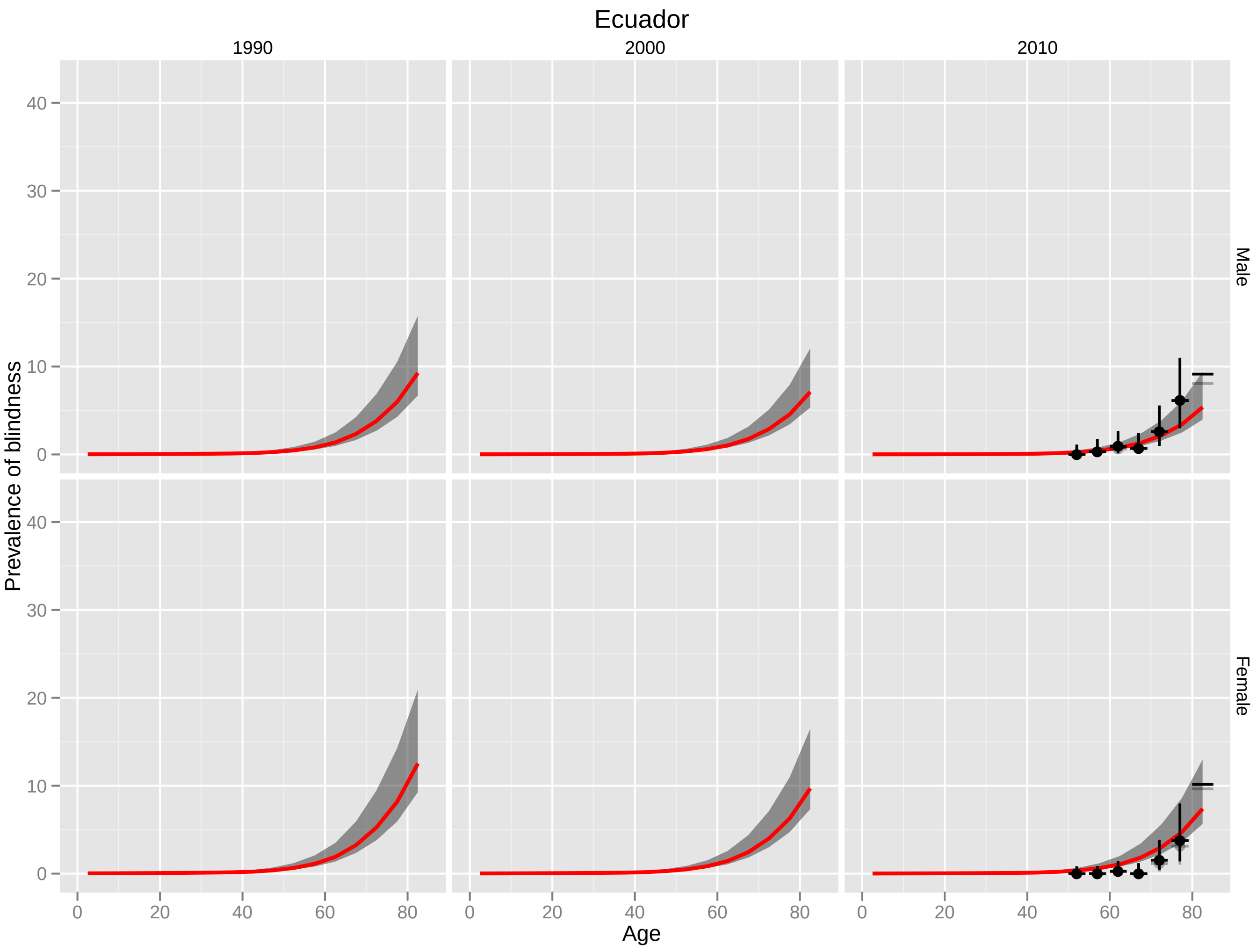

Ecuador
1990
2000
2010
Male
Female
Prevalence of blindness
Age
0
10
20
30
40
0
20
40
60
80

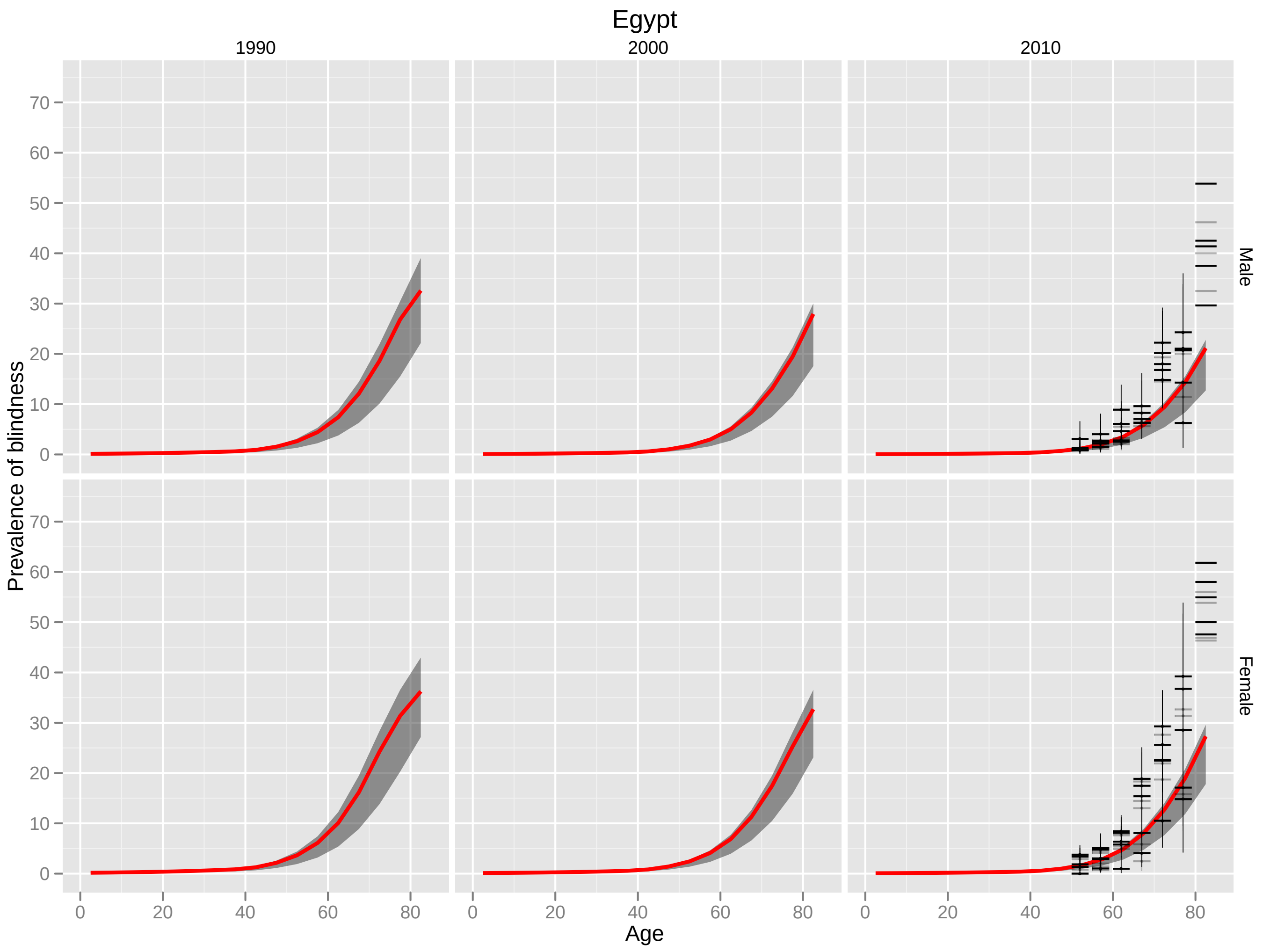

Egypt
1990
2000
2010
Male
Female
Prevalence of blindness
Age
0
10
20
30
40
50
60
70
80

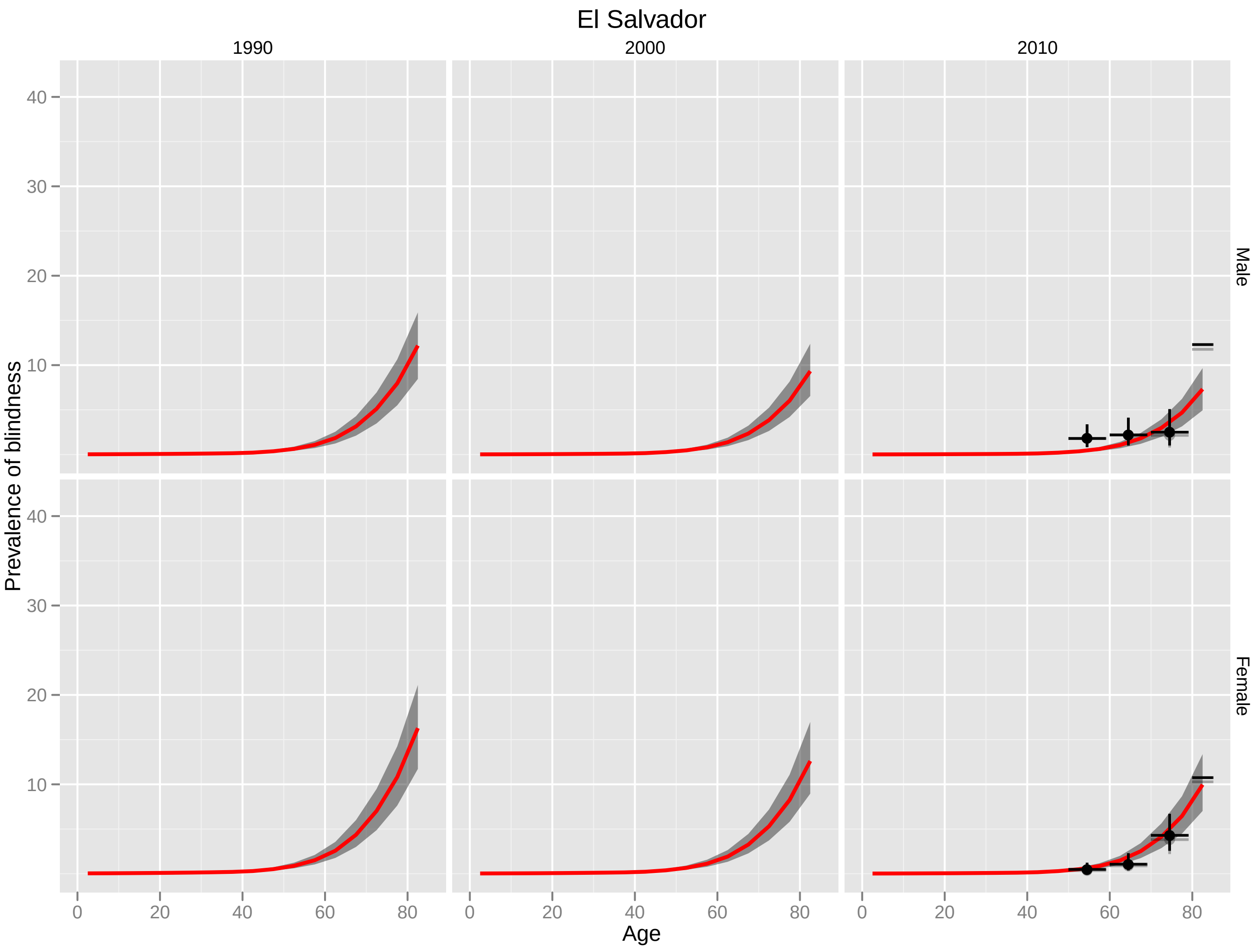
El Salvador
1990
2000
2010
Male
Female
Prevalence of blindness
Age
40
30
20
10
0
20
40
60
80

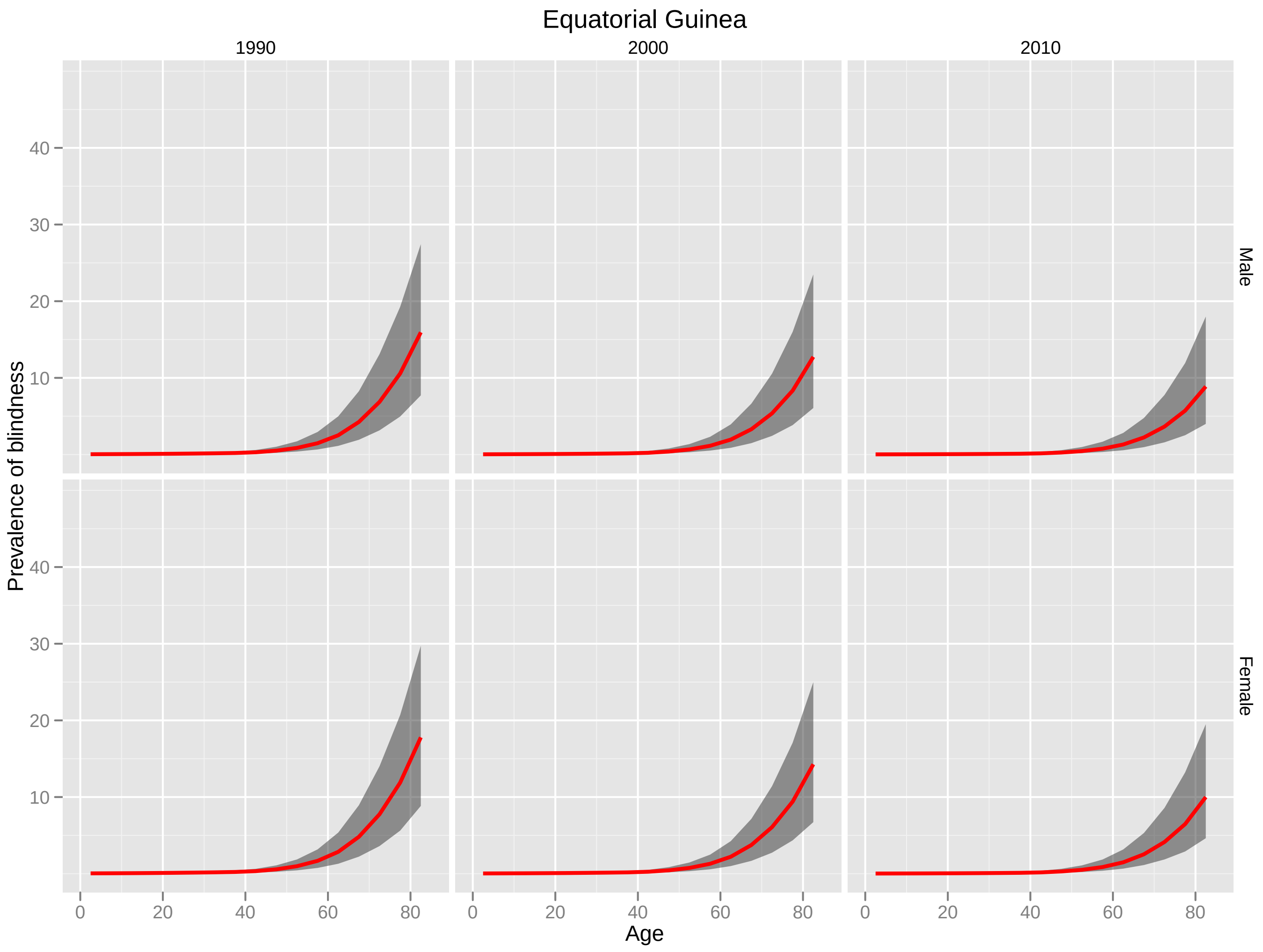
Equatorial Guinea
1990
2000
2010
Male
Female
Prevalence of blindness
10
20
30
40
Age
0
20
40
60
80

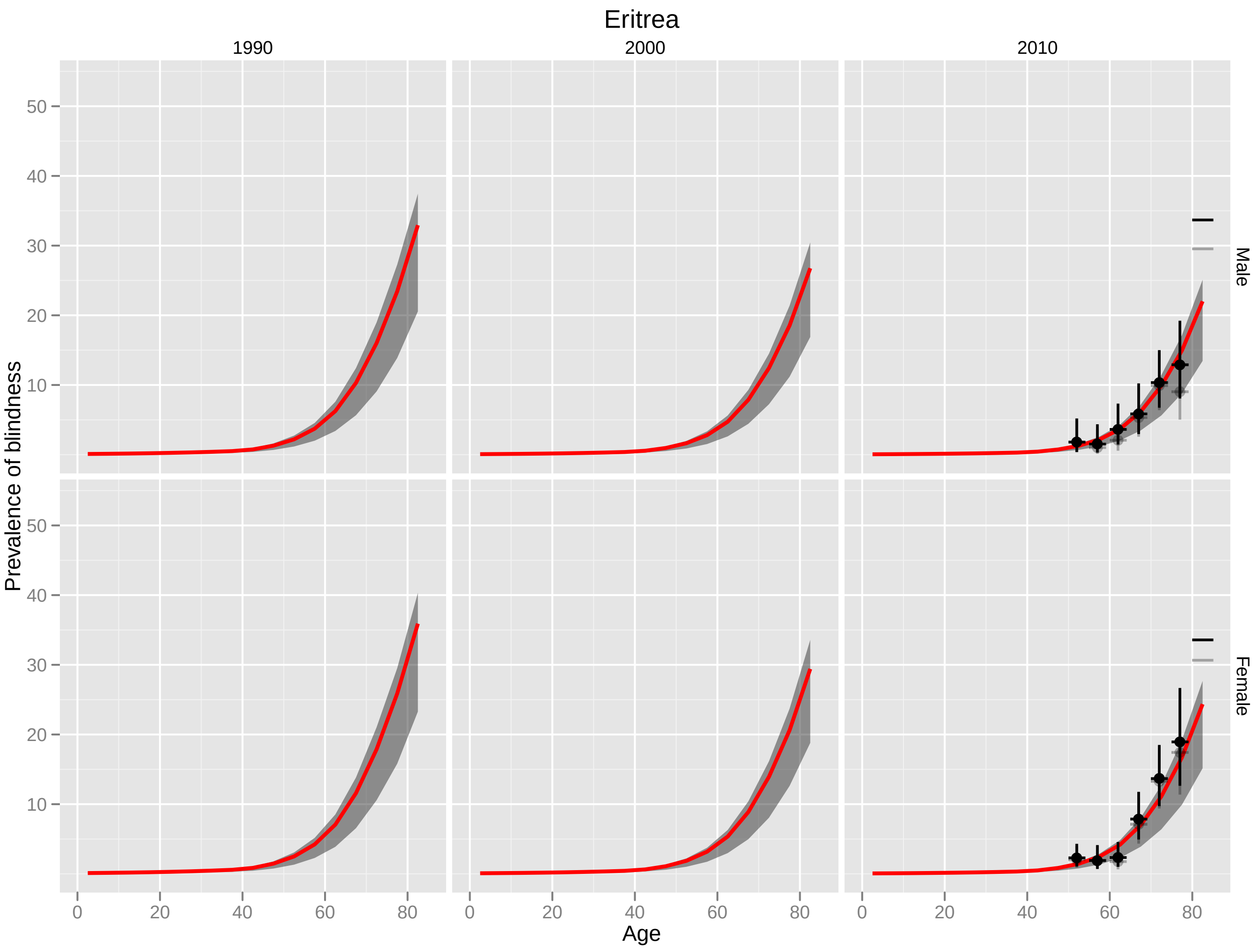
Eritrea
1990
2000
2010
Male
Female
Prevalence of blindness
Age
10
20
30
40
50
0
20
40
60
80

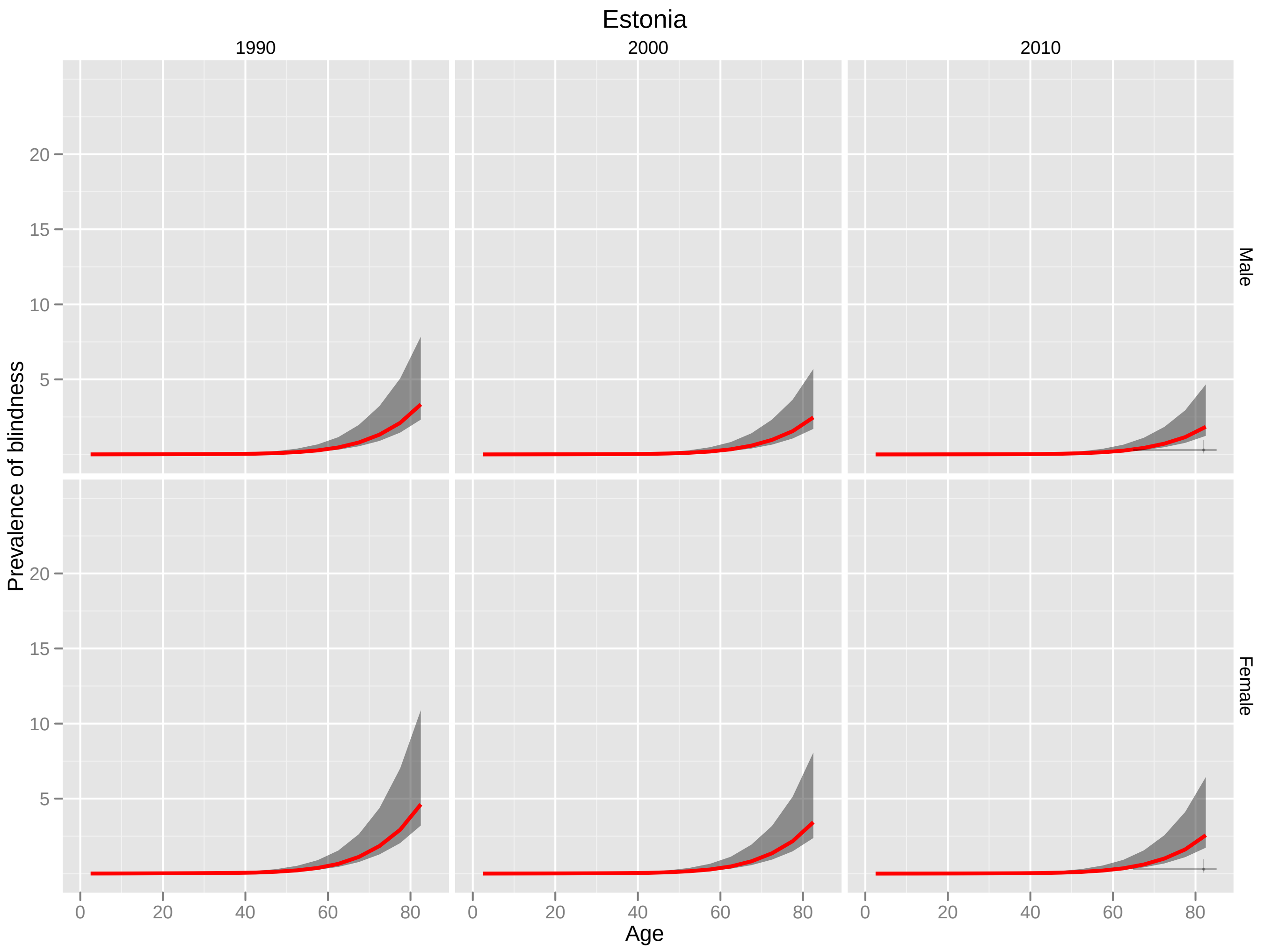
Estonia
1990
2000
2010
Male
Female
Prevalence of blindness
Age
0
20
40
60
80
5
10
15
20

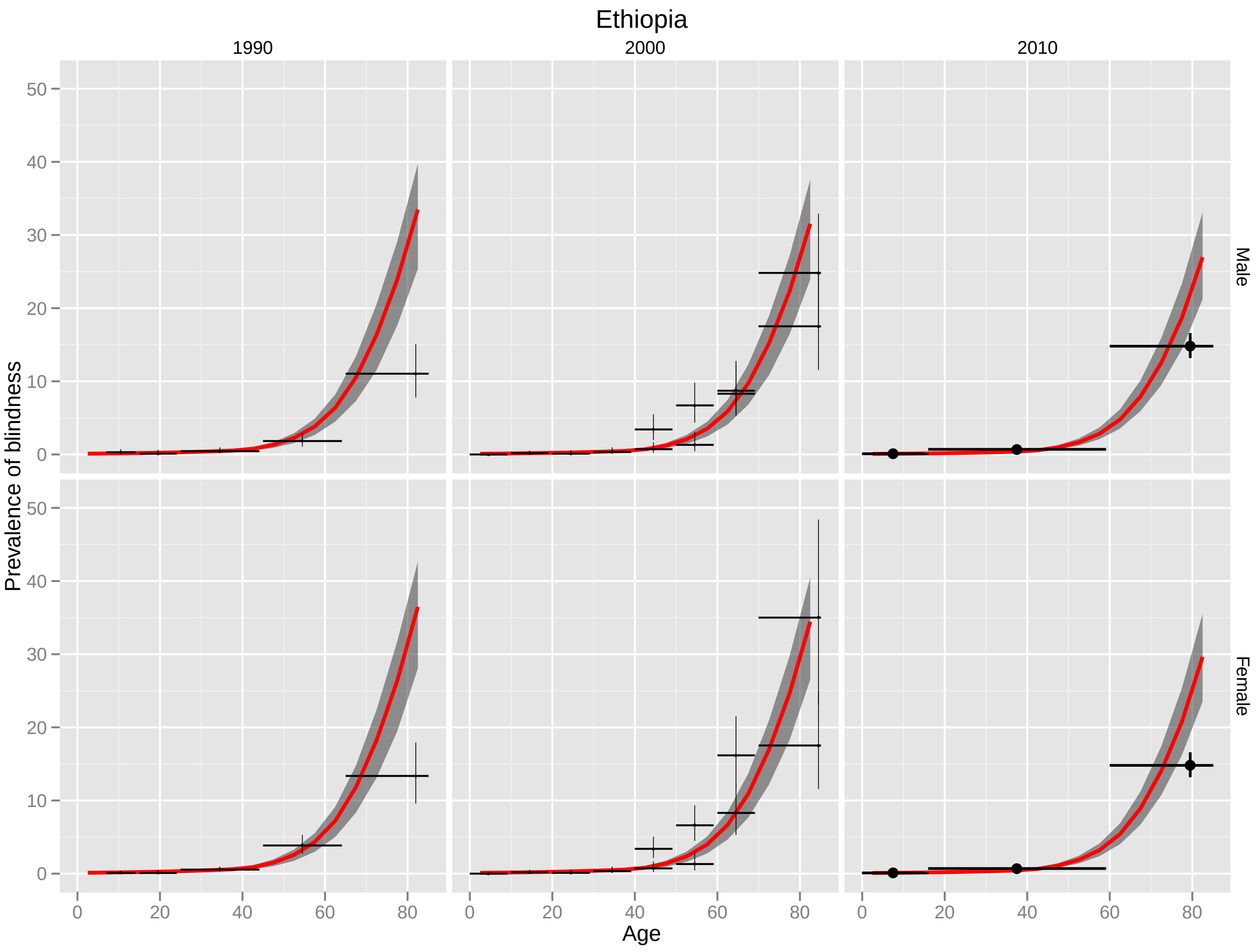

Ethiopia
1990
2000
2010
Male
Female
Prevalence of blindness
Age
0
10
20
30
40
50
0
20
40
60
80

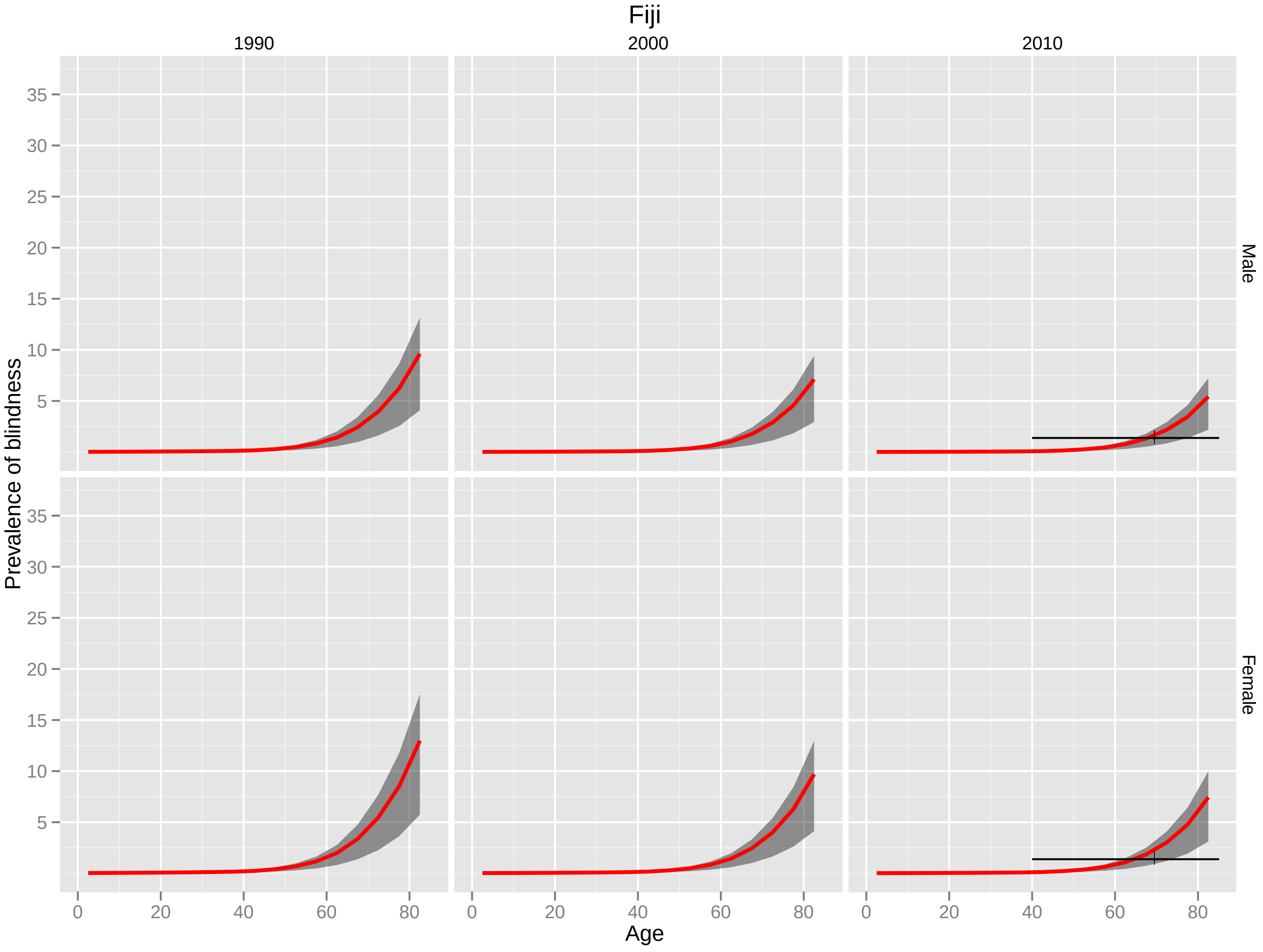
Fiji
1990
2000
2010
Male
Female
Prevalence of blindness
Age
35
30
25
20
15
10
5
0
20
40
60
80

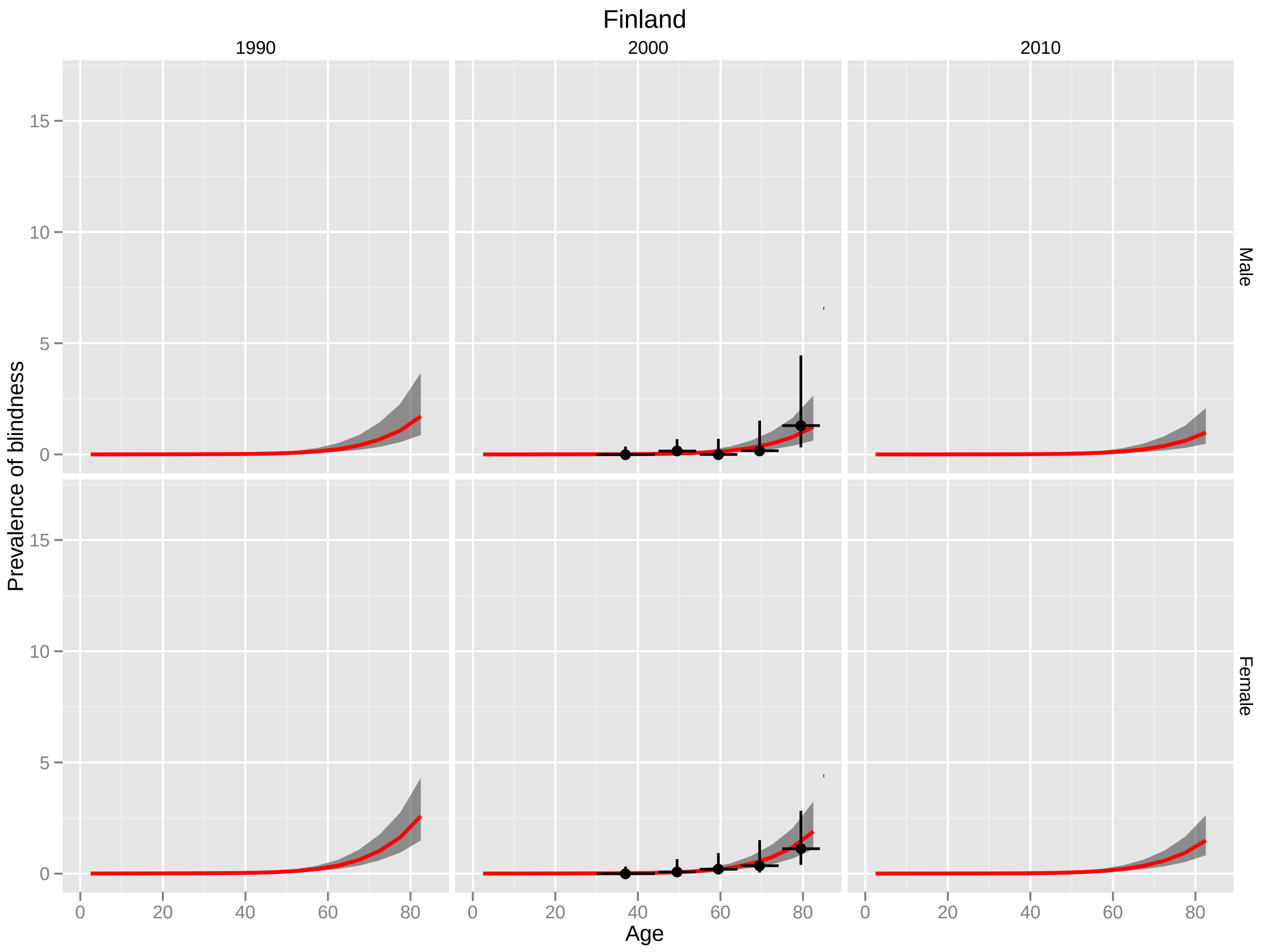
Finland
1990
2000
2010
Male
Female
Prevalence of blindness
0
5
10
15
0
20
40
60
80
Age

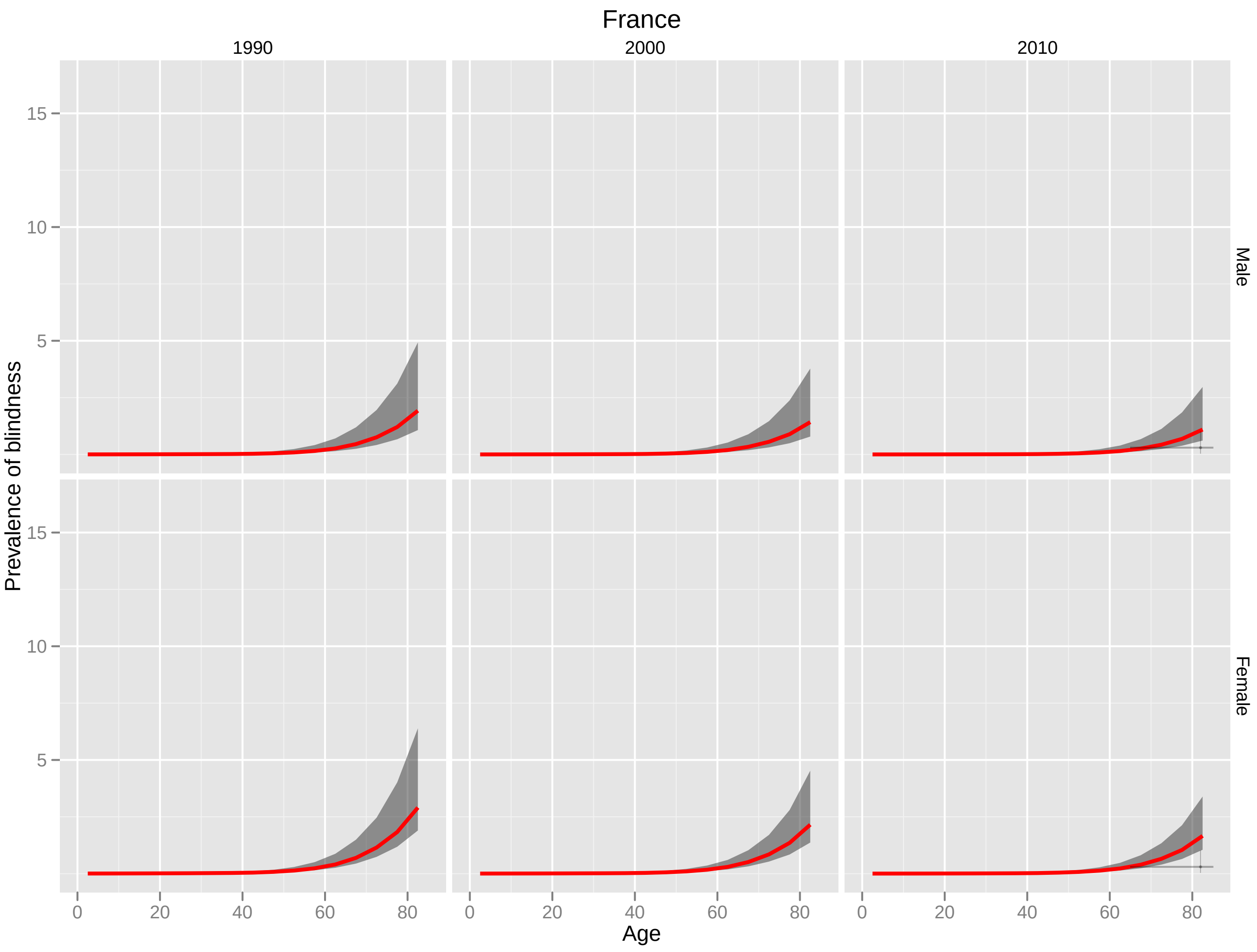
France
1990
2000
2010
Male
Female
Prevalence of blindness
15
10
5
15
10
5
0
20
40
60
80
Age

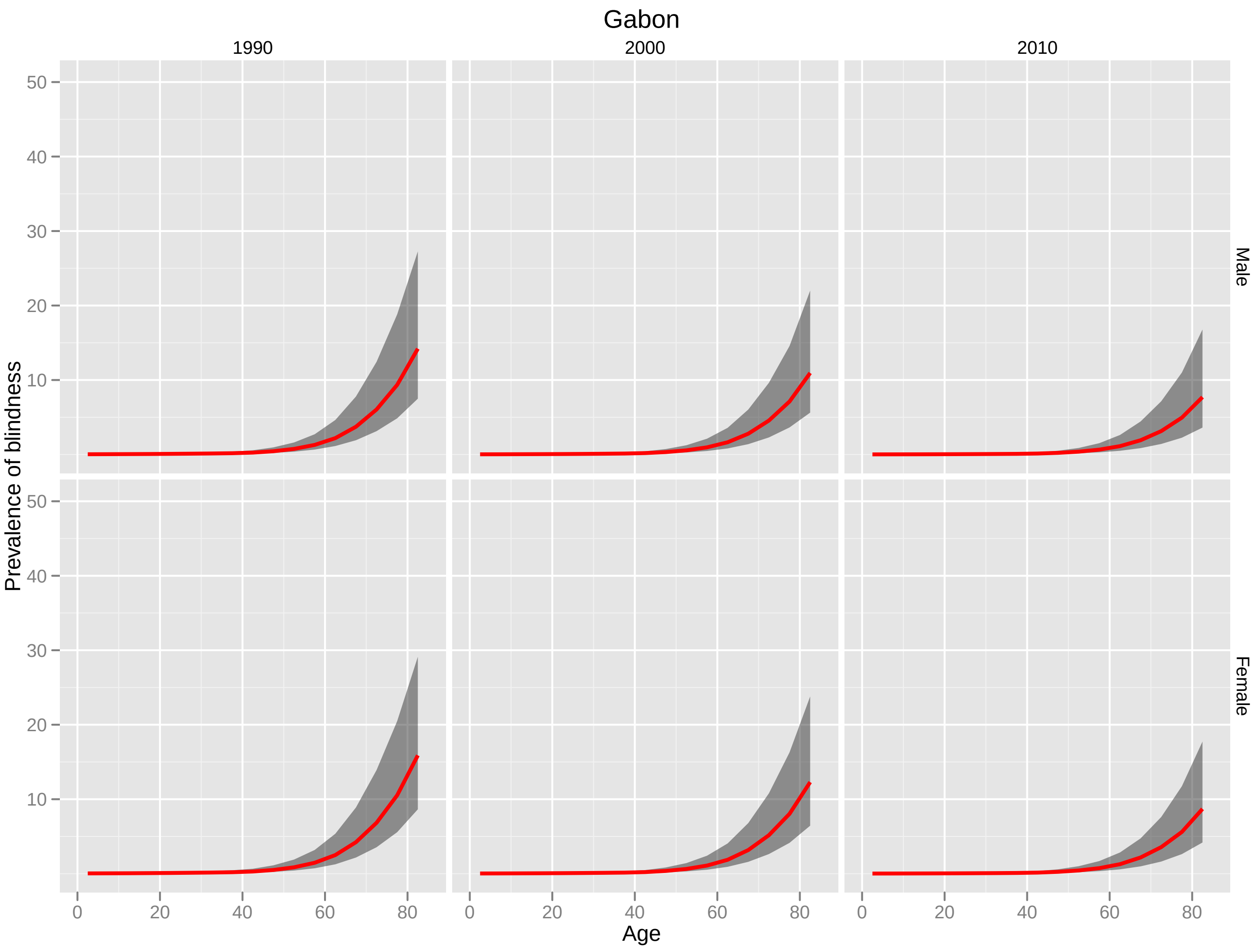
Gabon
1990
2000
2010
Male
Female
Prevalence of blindness
Age
50
40
30
20
10
0
20
40
60
80

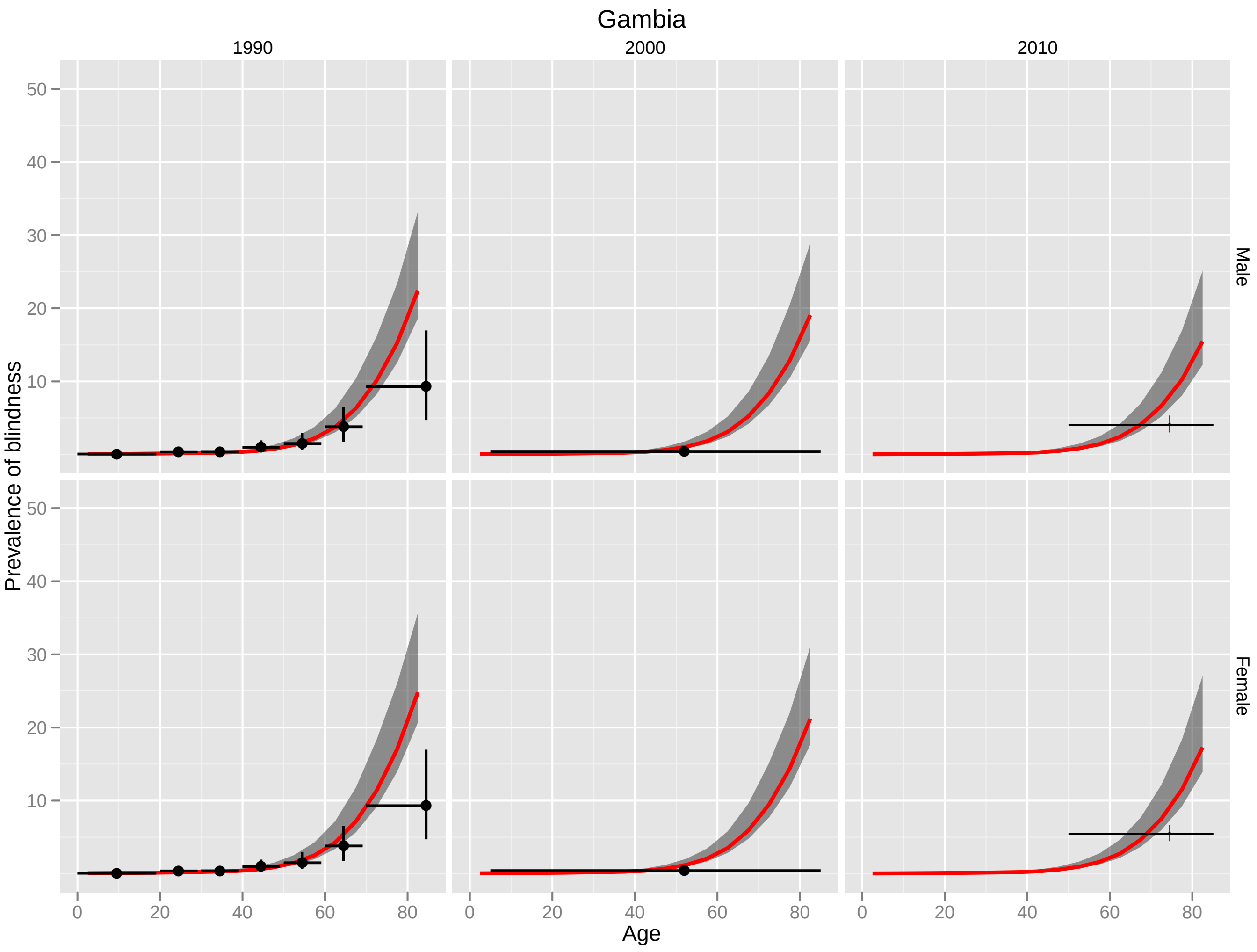
Gambia
1990
2000
2010
Male
Female
Prevalence of blindness
Age
0
20
40
60
80
10
20
30
40
50

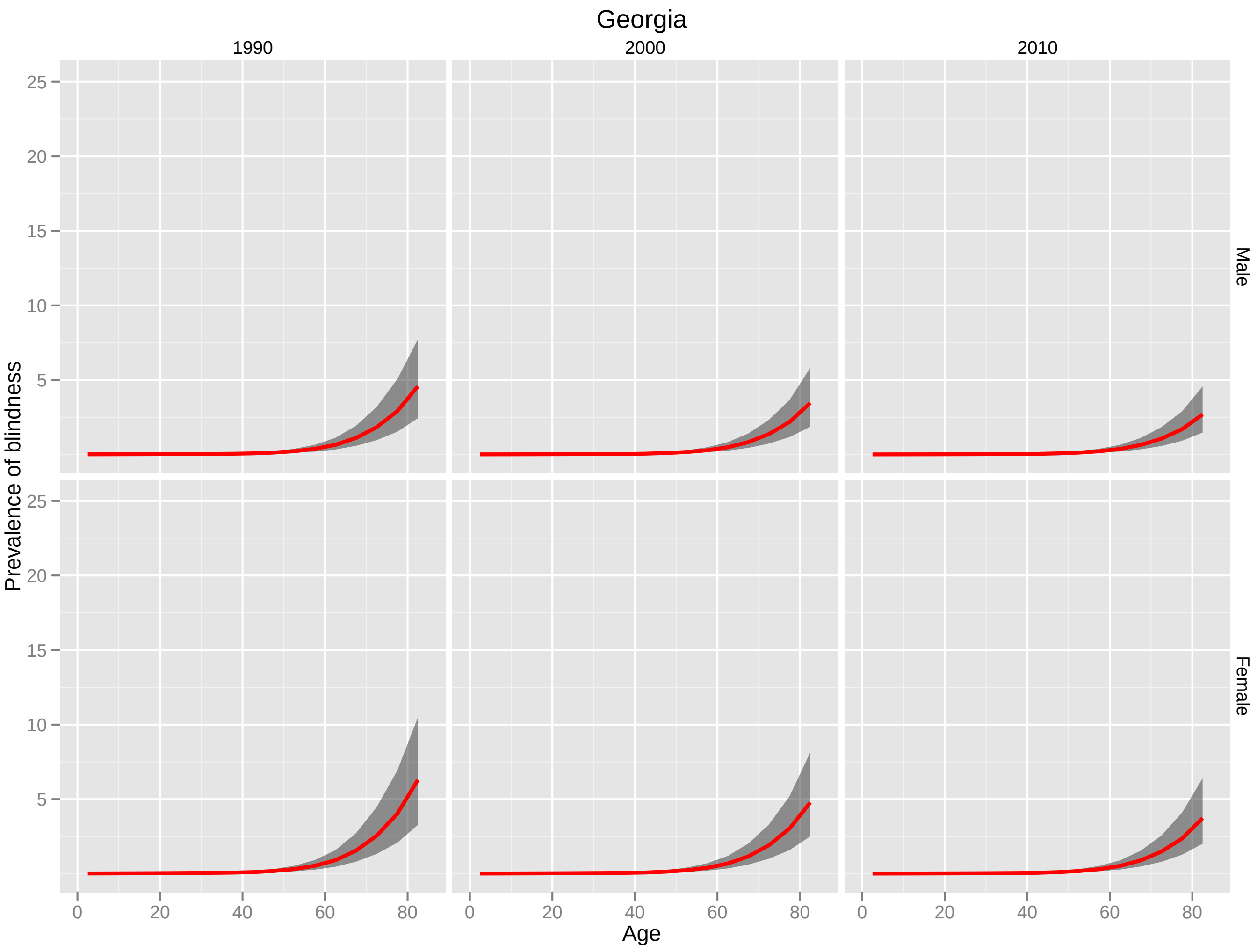
Georgia
1990
2000
2010
Male
Female
Prevalence of blindness
Age
25
20
15
10
5
0
20
40
60
80

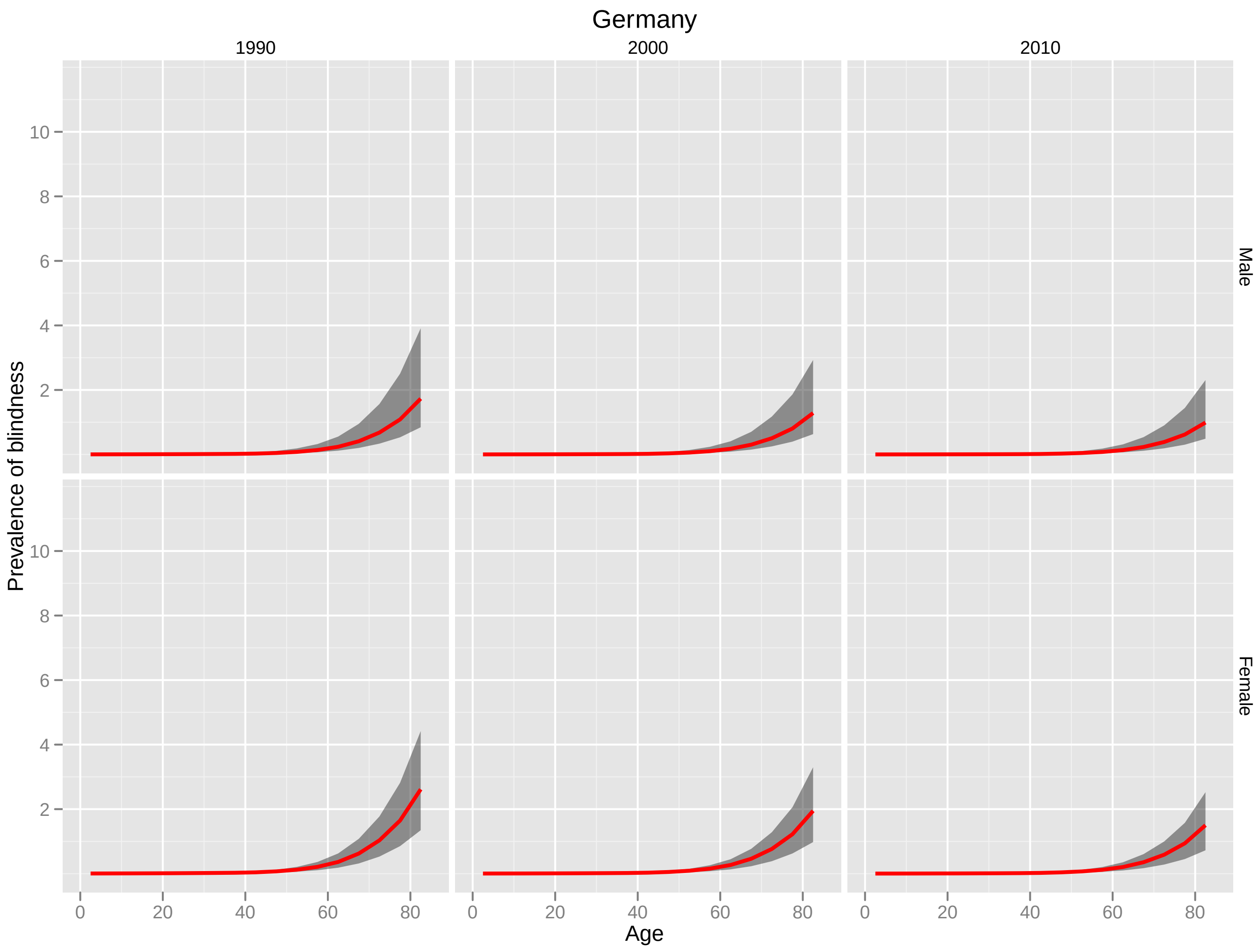
Germany
1990
2000
2010
Male
Female
Prevalence of blindness
2
4
6
8
10
0
20
40
60
80
Age

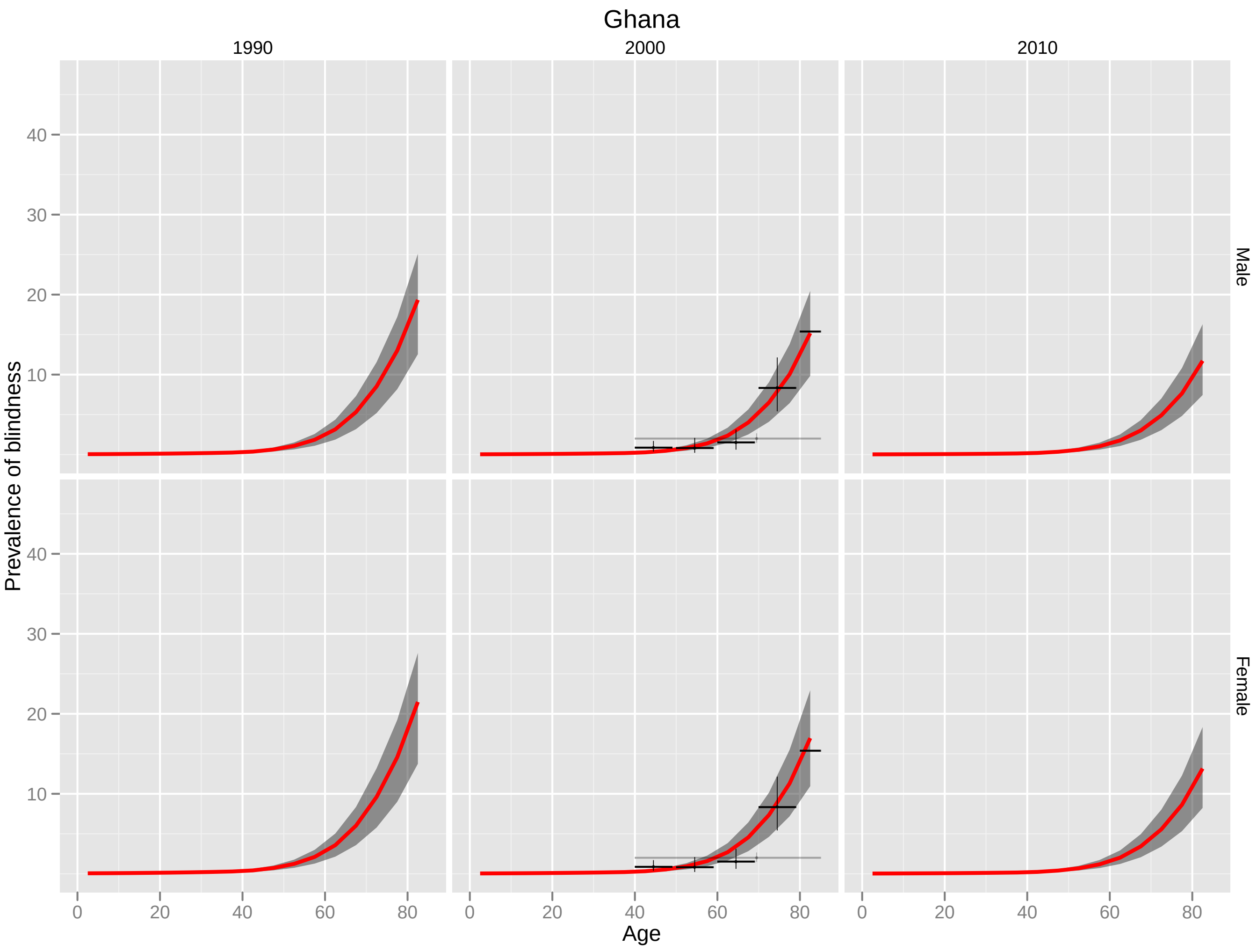
Ghana
1990
2000
2010
Male
Female
Prevalence of blindness
10
20
30
40
Age
0
20
40
60
80

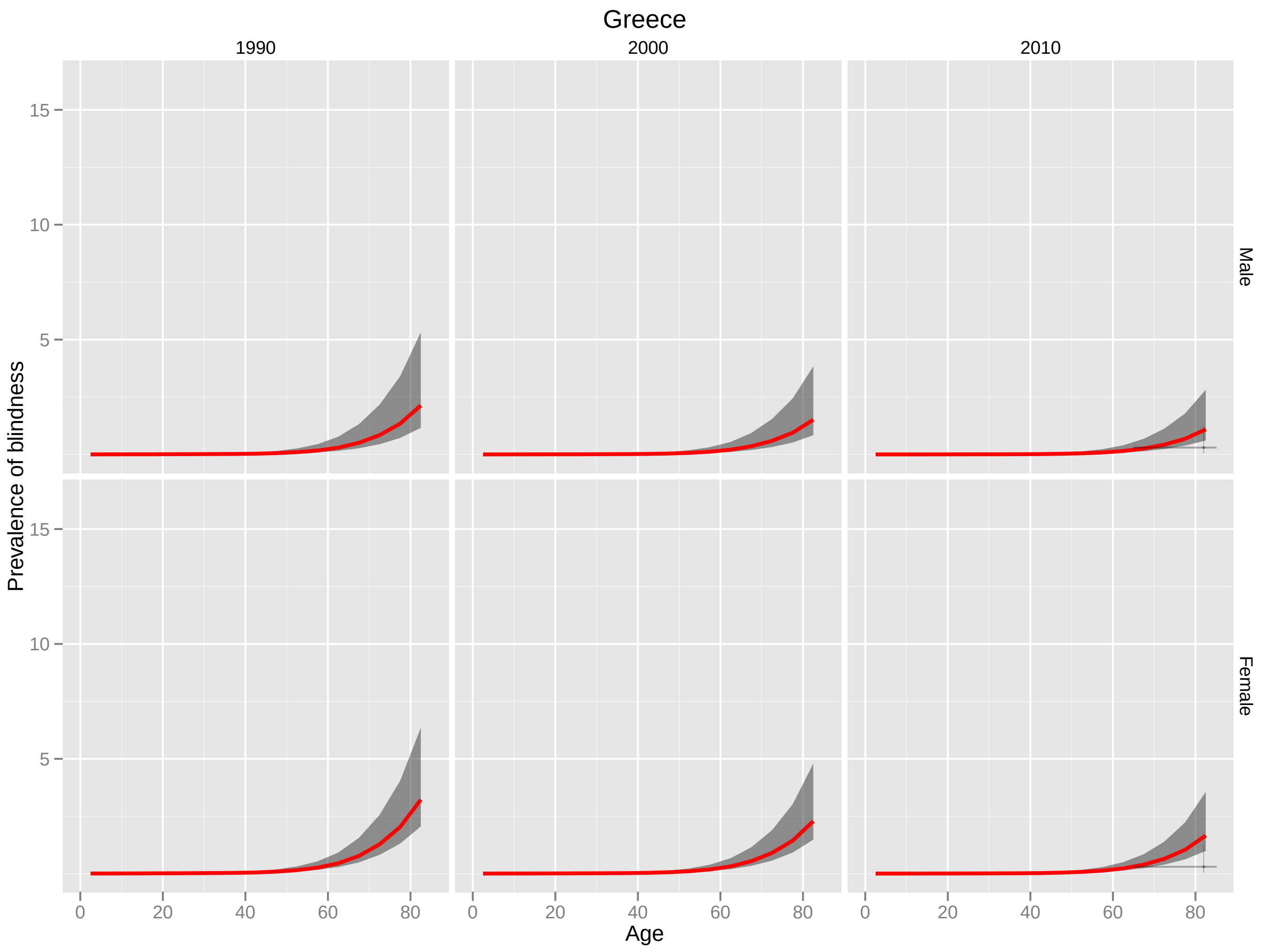
Greece
1990
2000
2010
Male
Female
Prevalence of blindness
15
10
5
0
20
40
60
80
Age

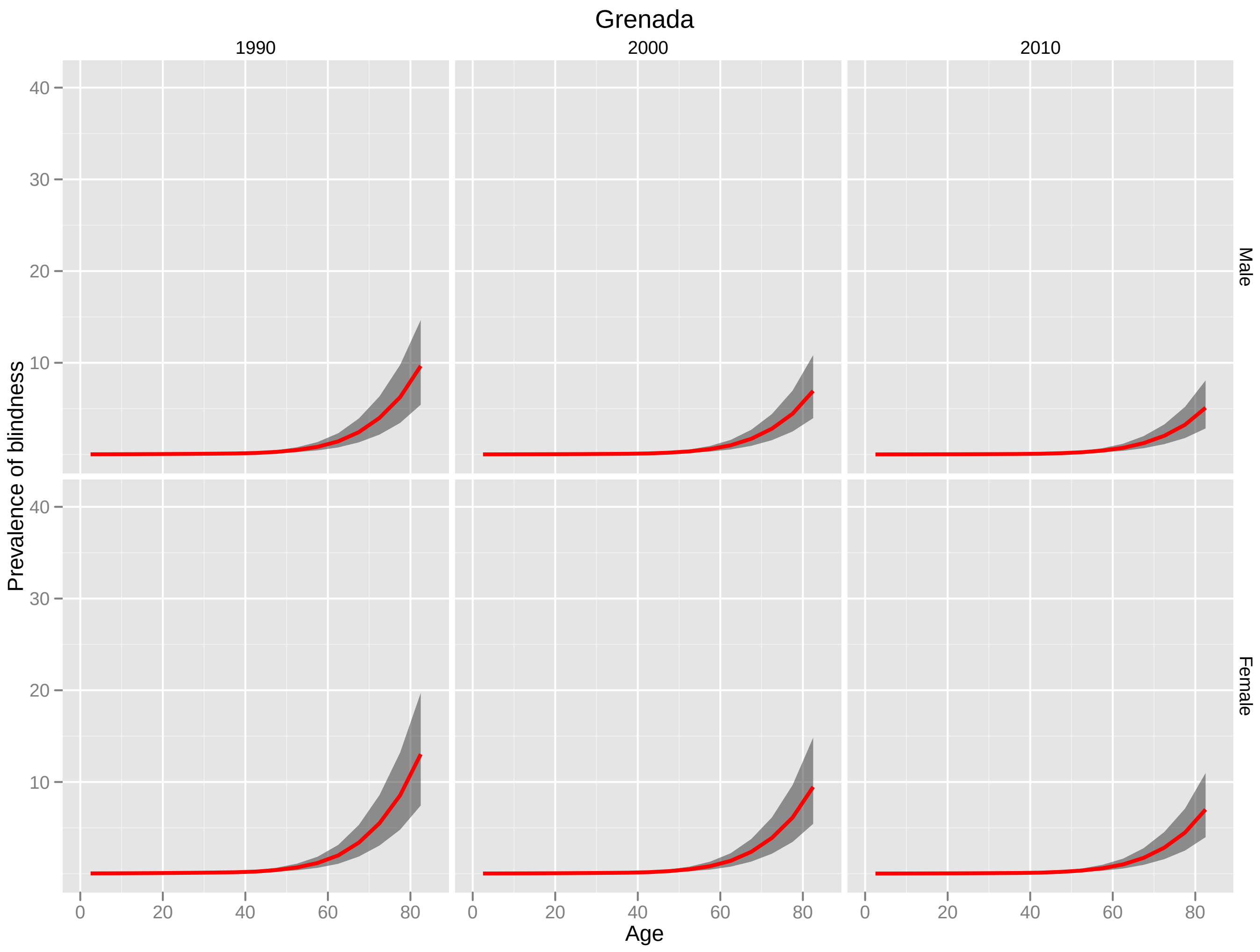
Grenada
1990
2000
2010
Male
Female
Prevalence of blindness
Age
0
10
20
30
40
60
80

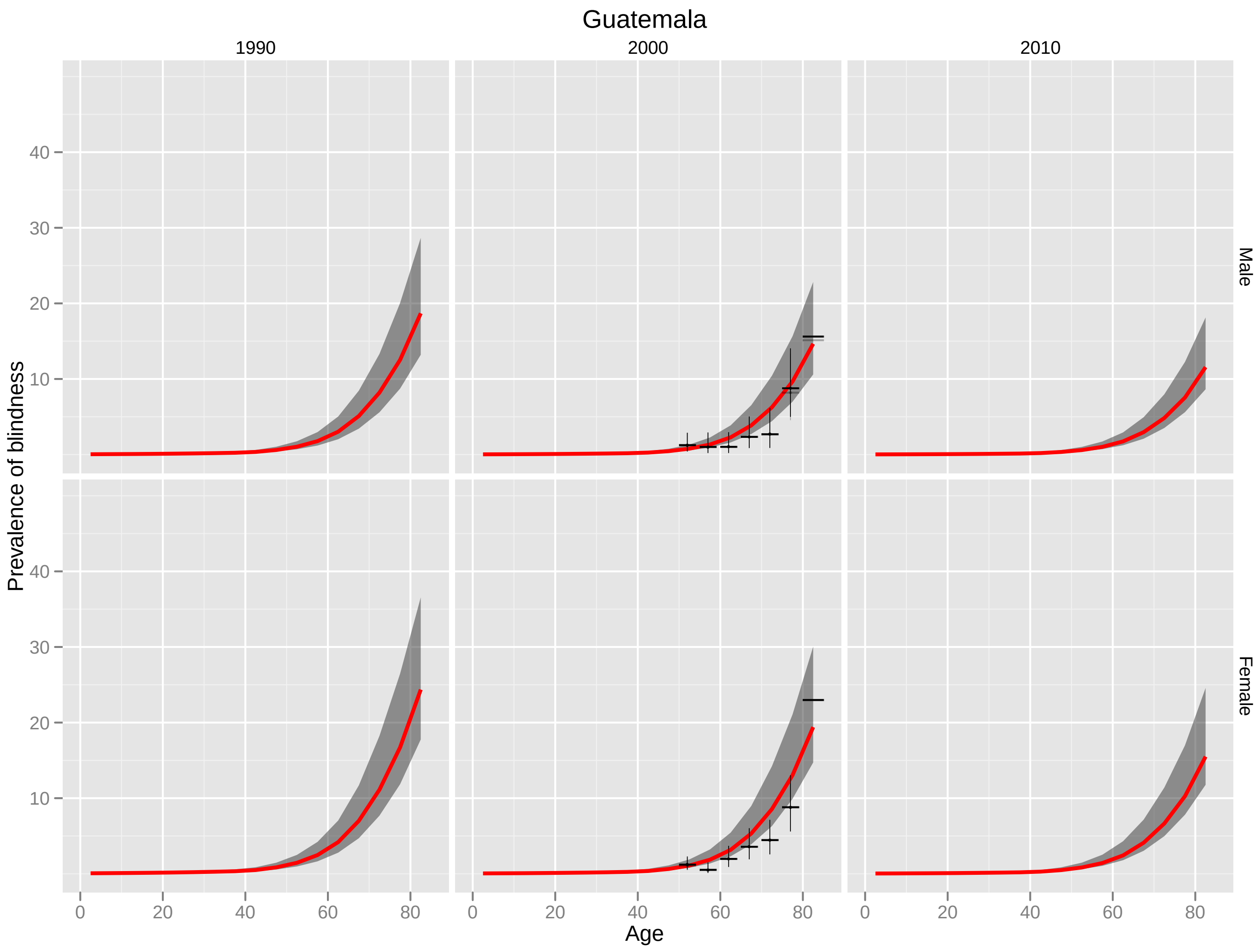
Guatemala
1990
2000
2010
Male
Female
Prevalence of blindness
Age
10
20
30
40
0
20
40
60
80

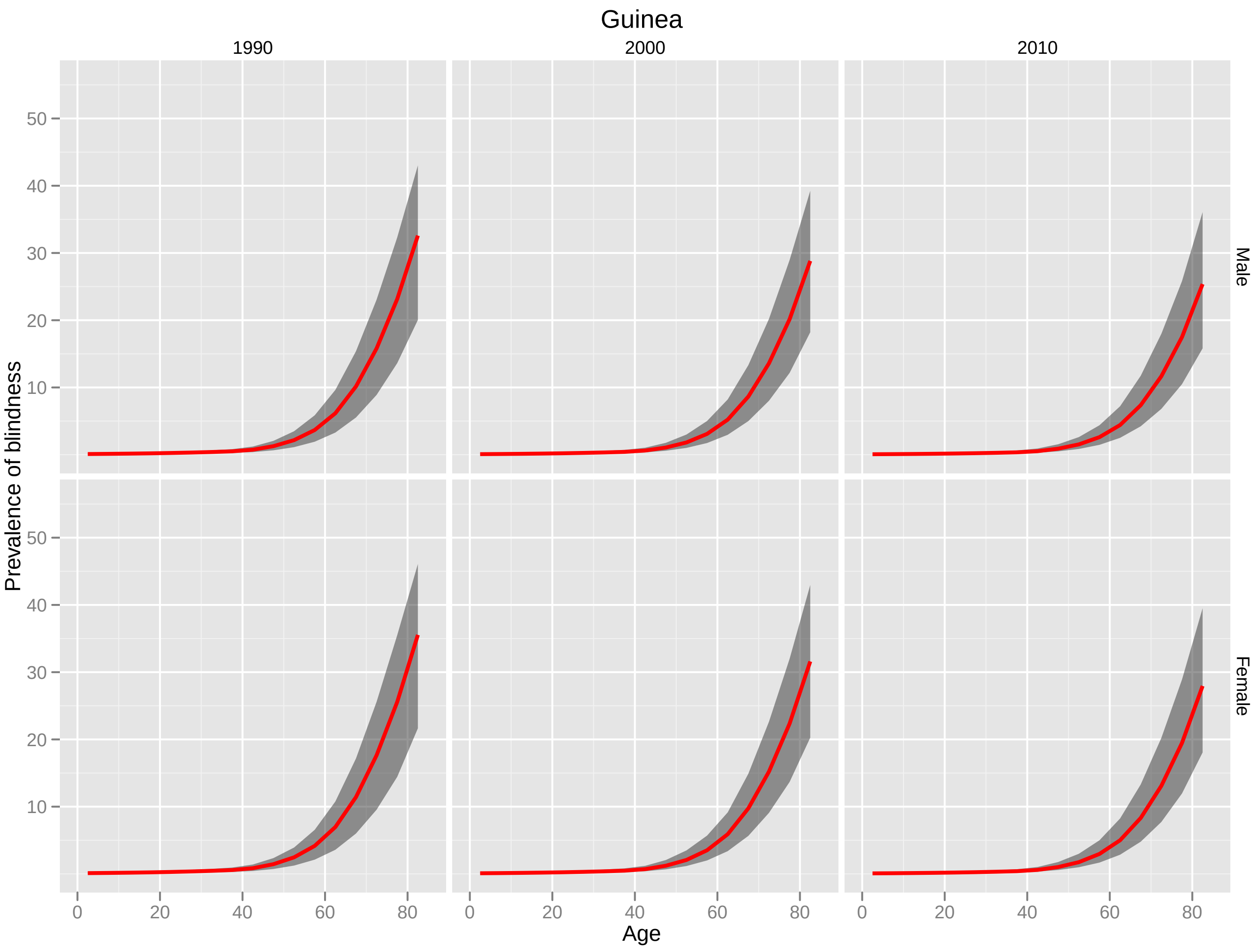
Guinea
1990
2000
2010
Male
Female
Prevalence of blindness
10
20
30
40
50
0
20
40
60
80
Age

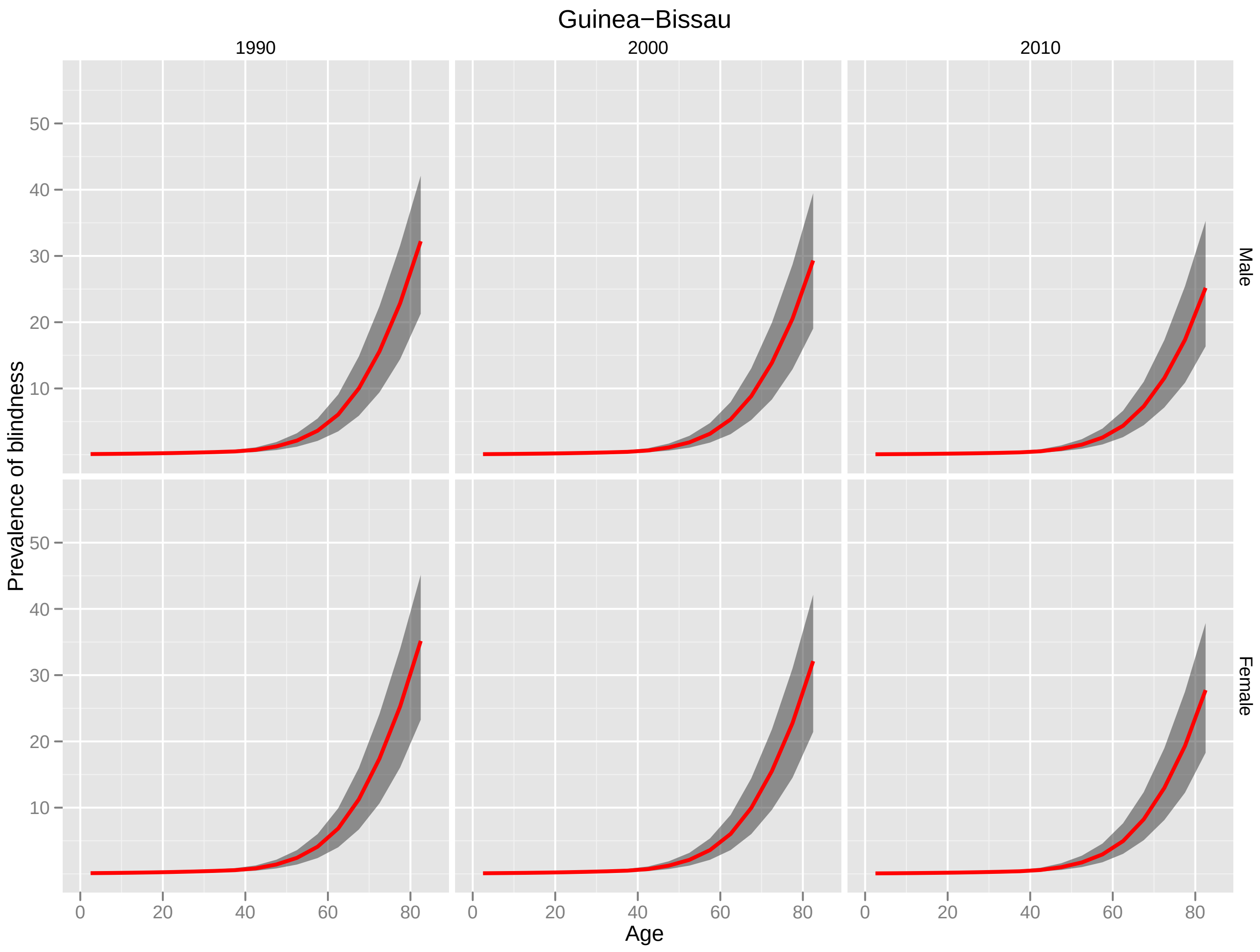
Guinea−Bissau
1990
2000
2010
Male
Female
Prevalence of blindness
Age
10
20
30
40
50
0
20
40
60
80

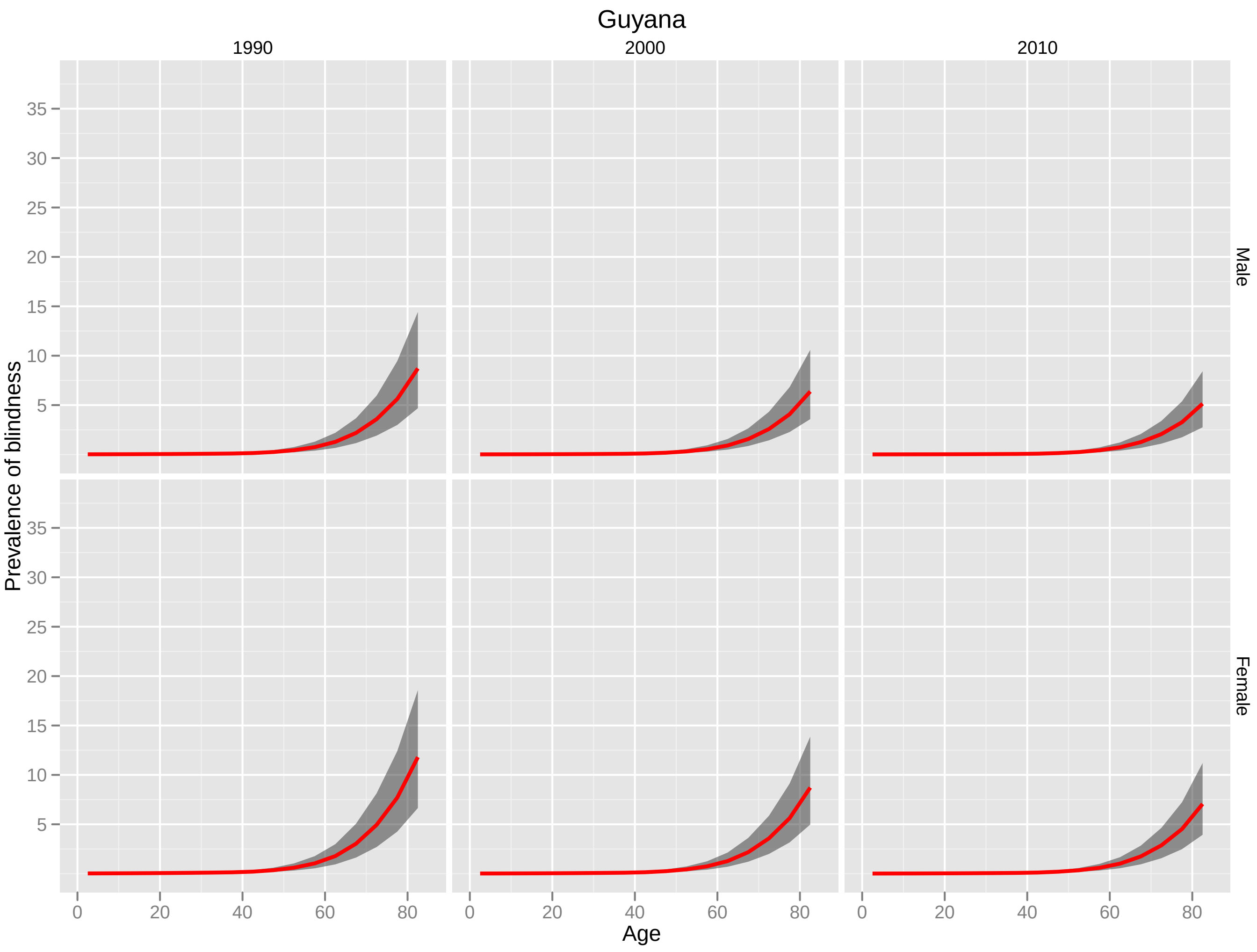

Guyana
1990
2000
2010
Male
Female
Prevalence of blindness
Age
35
30
25
20
15
10
5
0
20
40
60
80

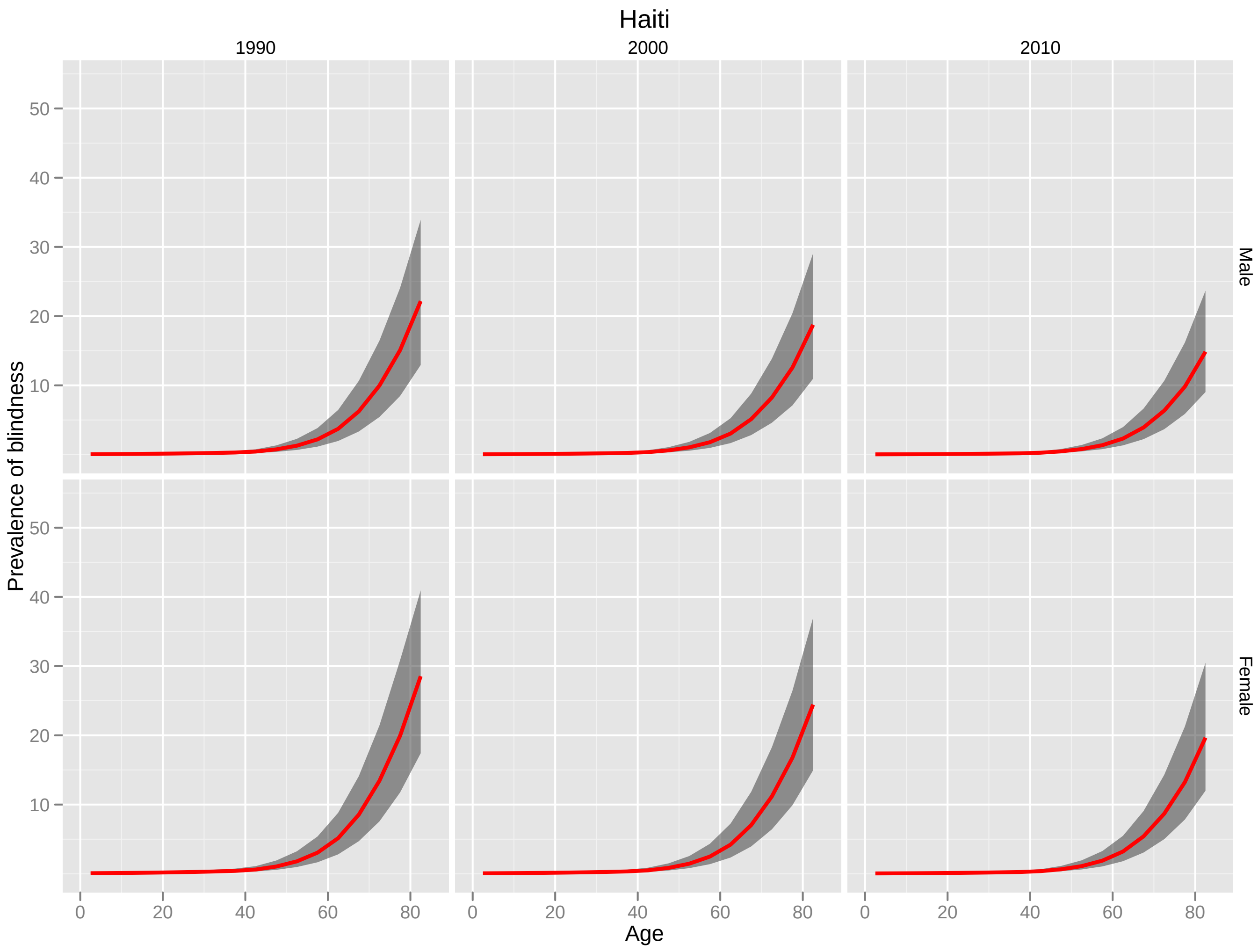
Haiti
1990
2000
2010
Male
Female
Prevalence of blindness
10
20
30
40
50
Age
0
20
40
60
80

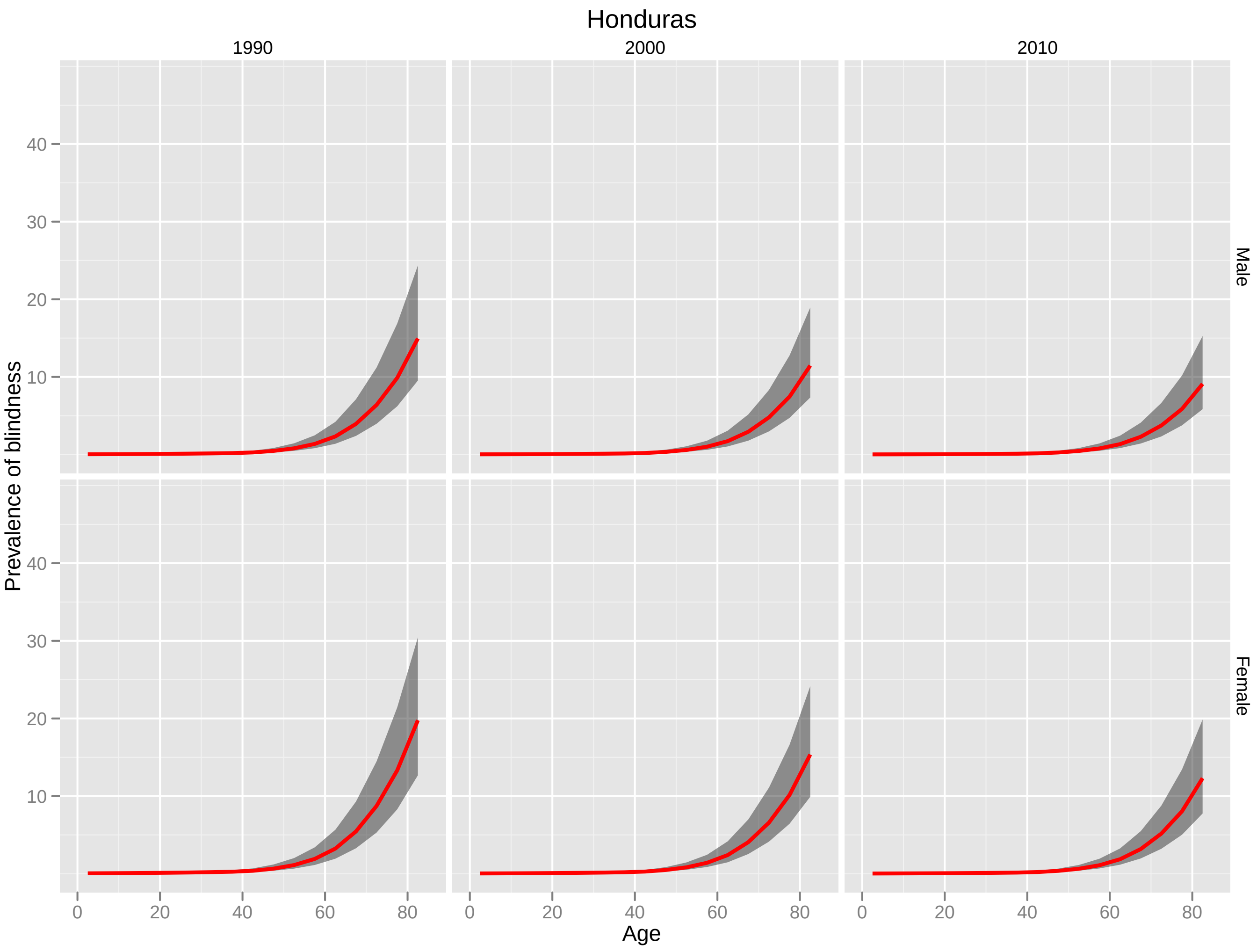
Honduras
1990
2000
2010
Male
Female
Prevalence of blindness
10
20
30
40
0
20
40
60
80
Age

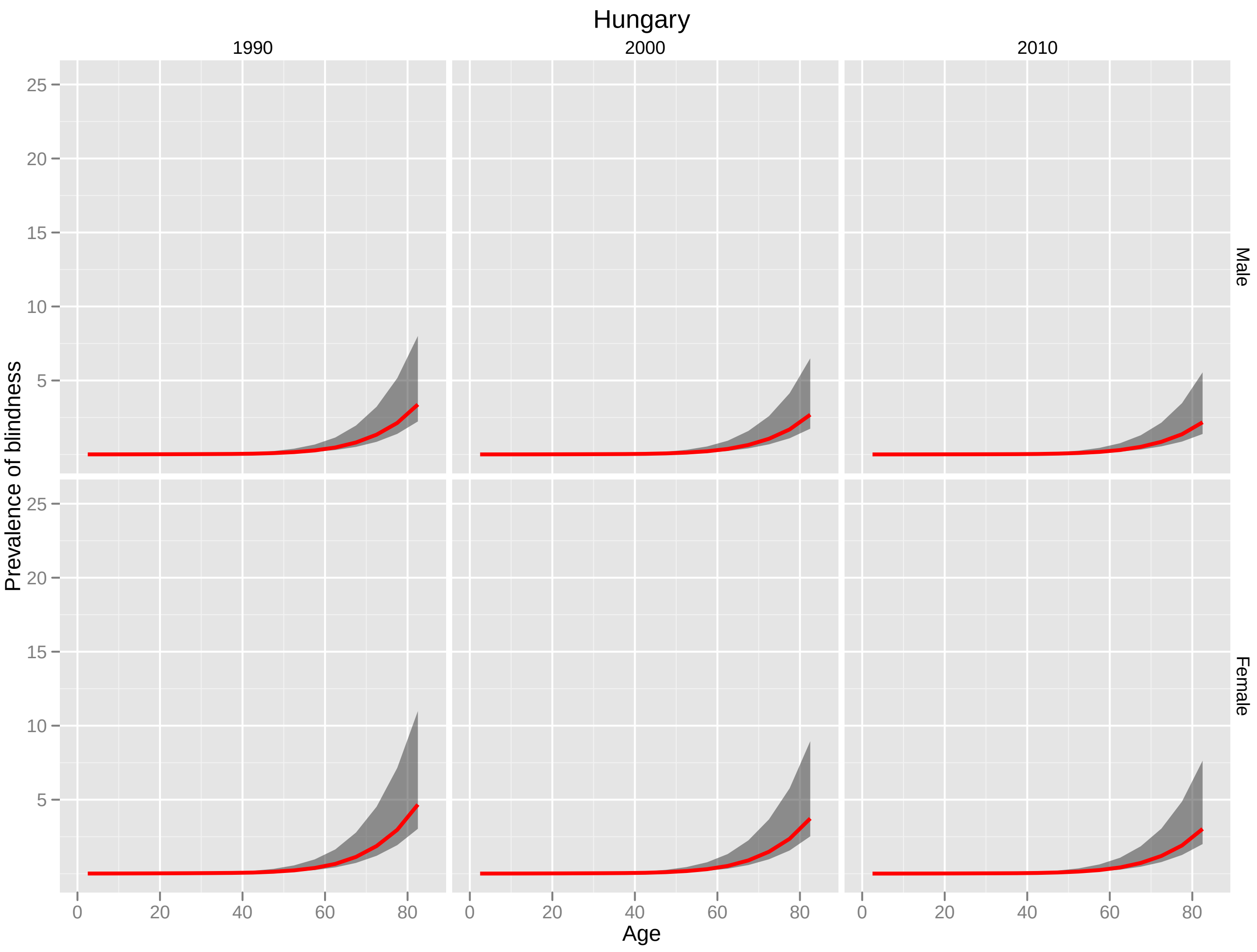
Hungary
1990
2000
2010
Male
Female
Prevalence of blindness
Age
0
20
40
60
80
5
10
15
20
25

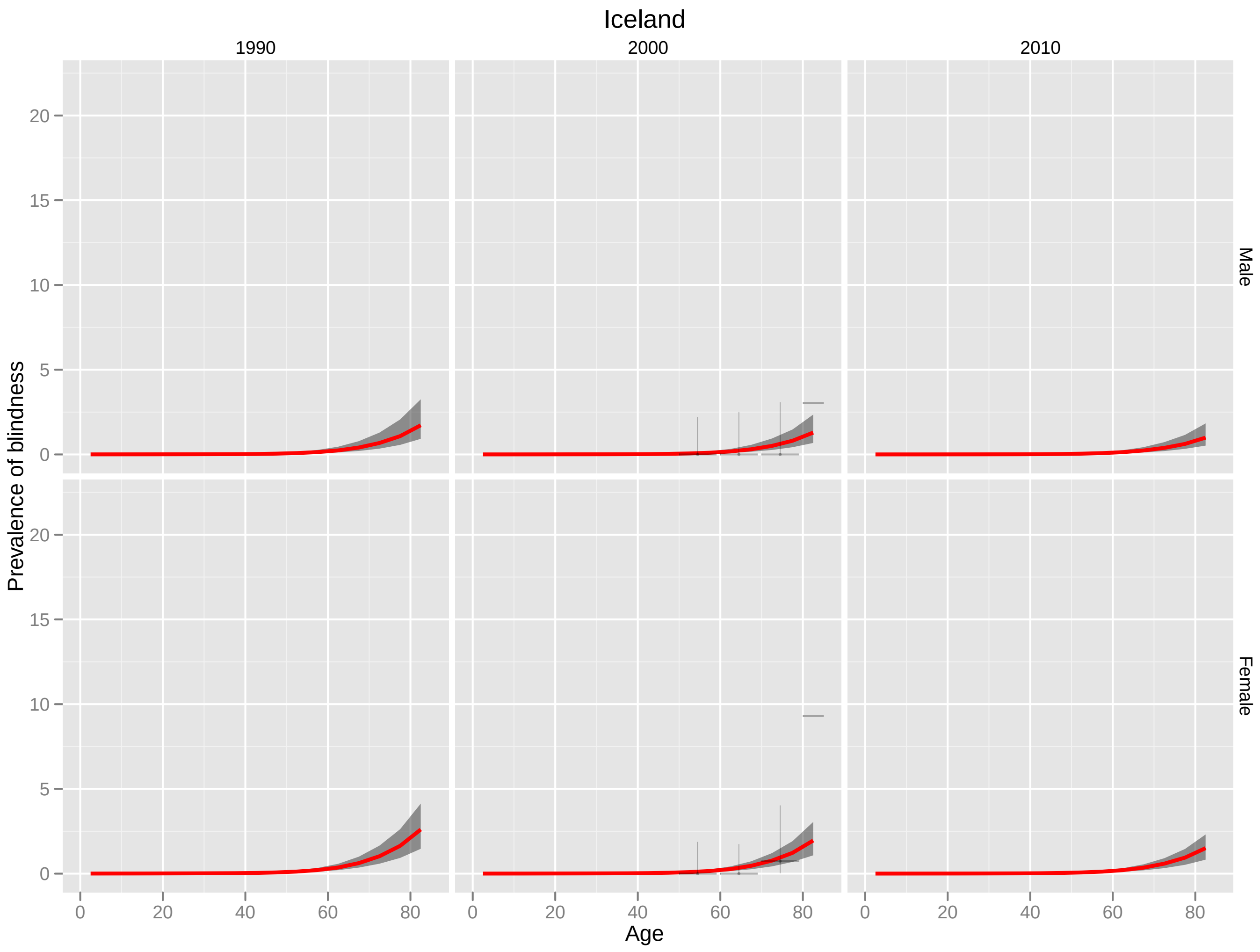
Iceland
1990
2000
2010
Male
Female
Prevalence of blindness
Age
0
5
10
15
20
0
20
40
60
80

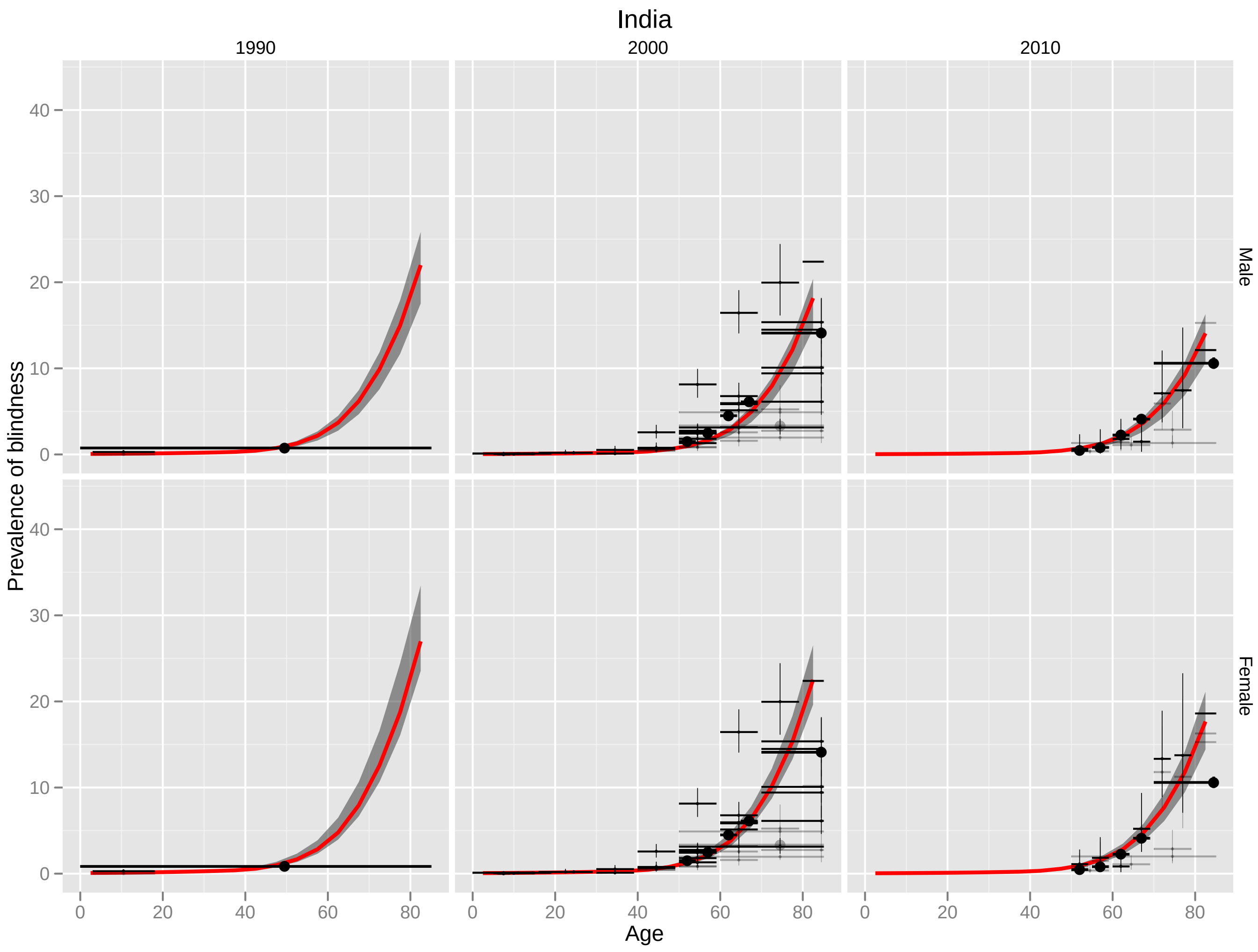
India
1990
2000
2010
Male
Female
Prevalence of blindness
Age
0
10
20
30
40
0
20
40
60
80

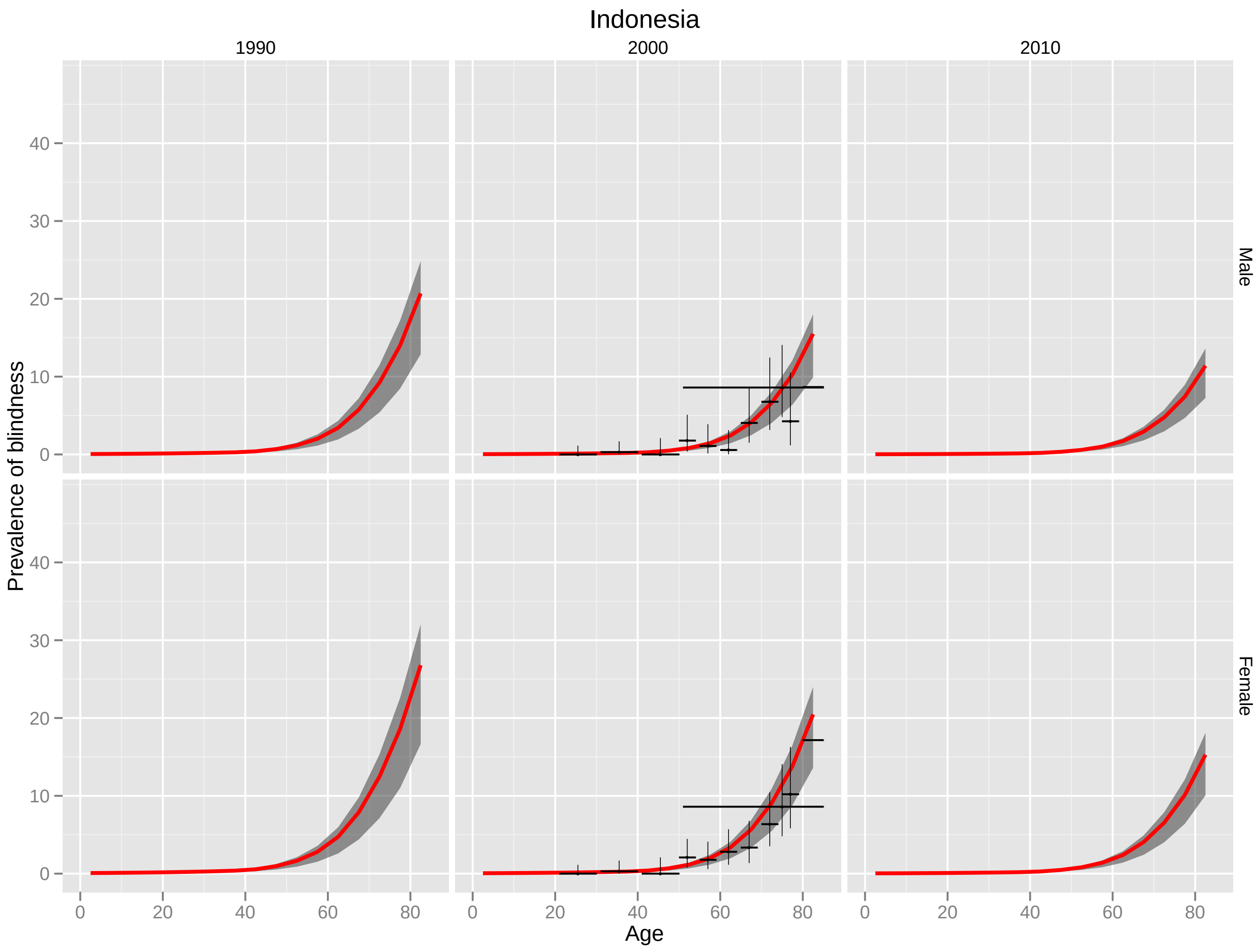
Indonesia
1990
2000
2010
Male
Female
Prevalence of blindness
Age
0
10
20
30
40
0
20
40
60
80

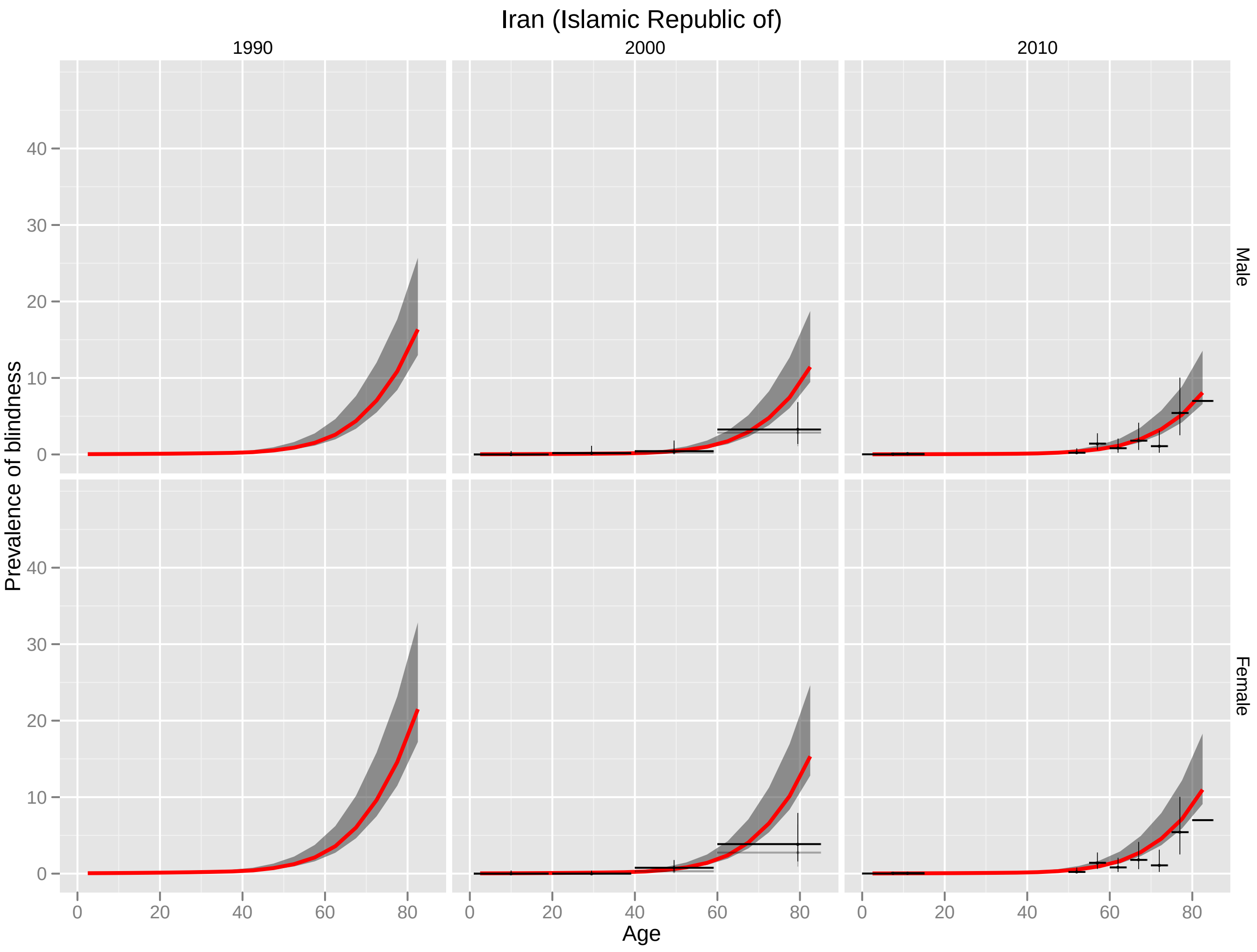
Iran (Islamic Republic of)
1990
2000
2010
Male
Female
Prevalence of blindness
Age
0
10
20
30
40
0
20
40
60
80

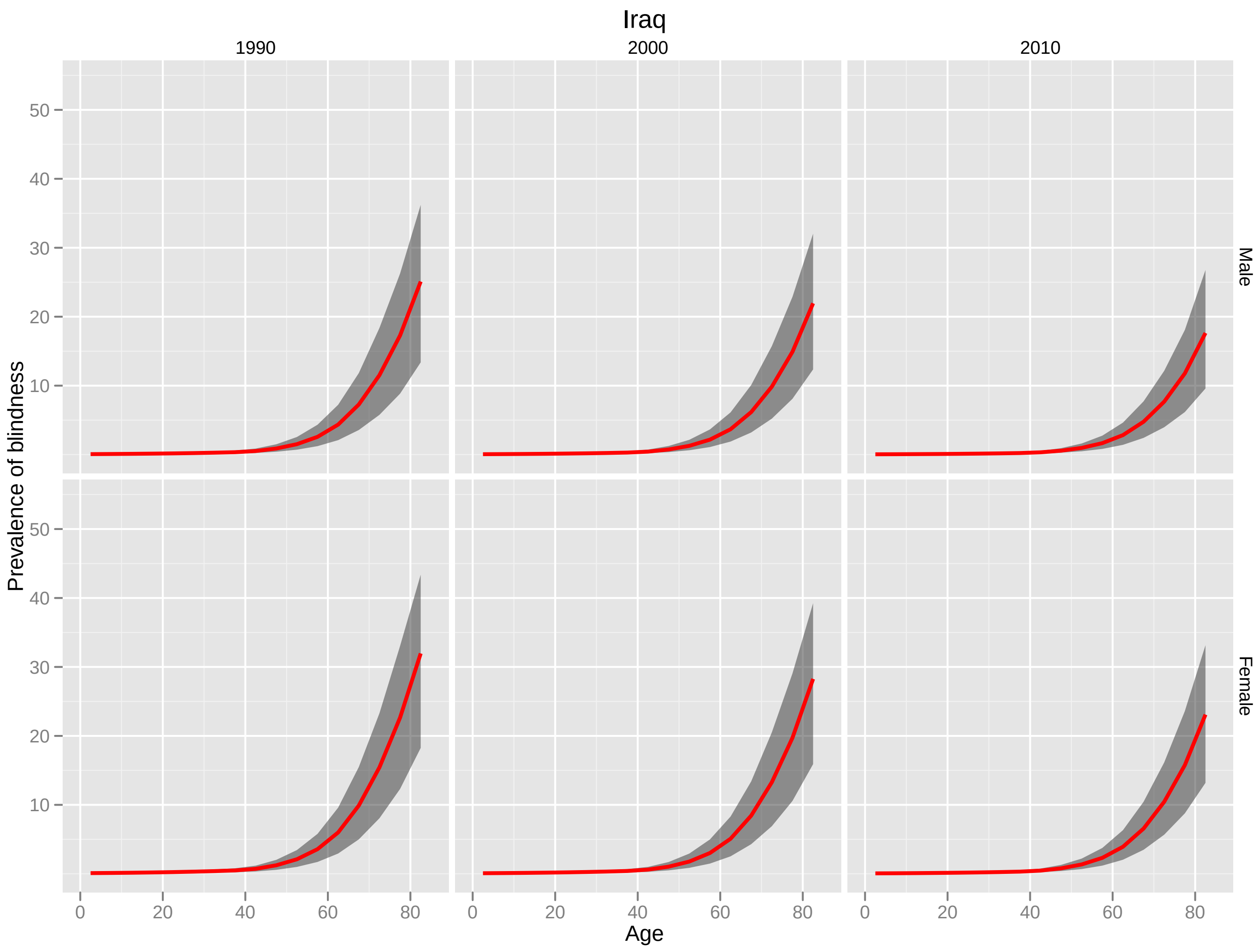
Iraq
1990
2000
2010
Male
Female
Prevalence of blindness
Age
50
40
30
20
10
0
20
40
60
80

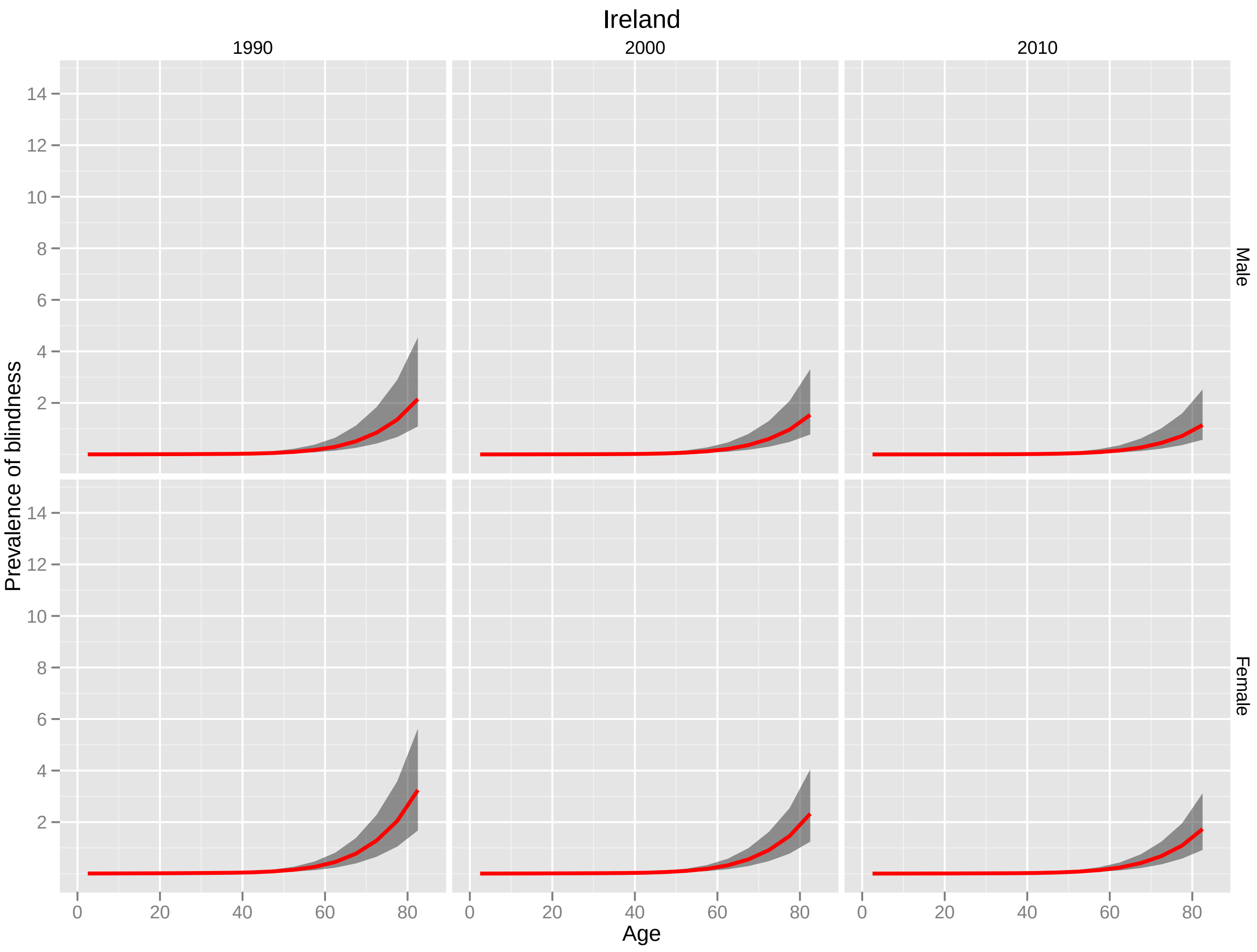

Ireland
1990
2000
2010
Male
Female
Prevalence of blindness
Age
14
12
10
8
6
4
2
0
20
40
60
80

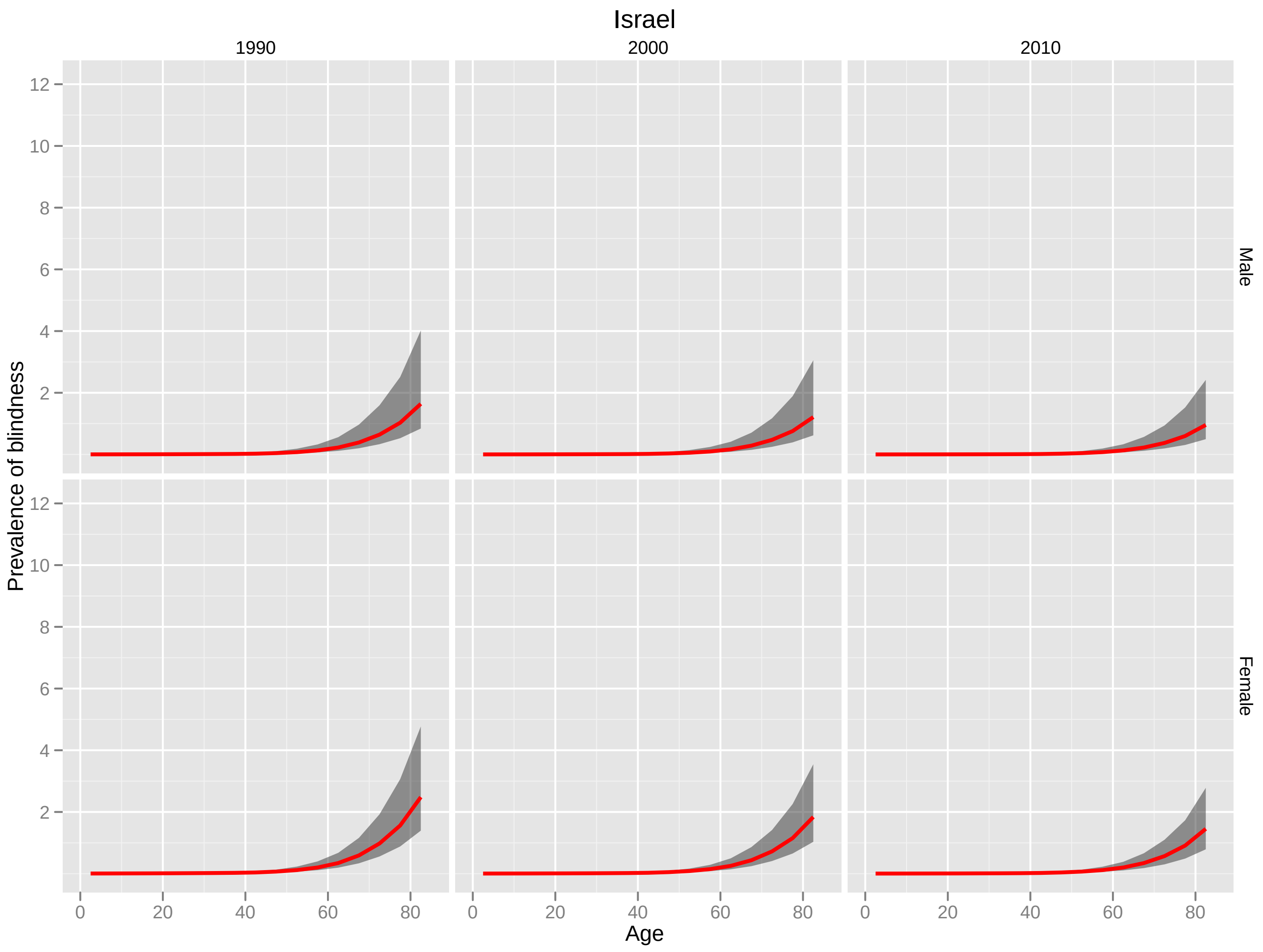
Israel
1990
2000
2010
Male
Female
Prevalence of blindness
Age
0
20
40
60
80
2
4
6
8
10
12

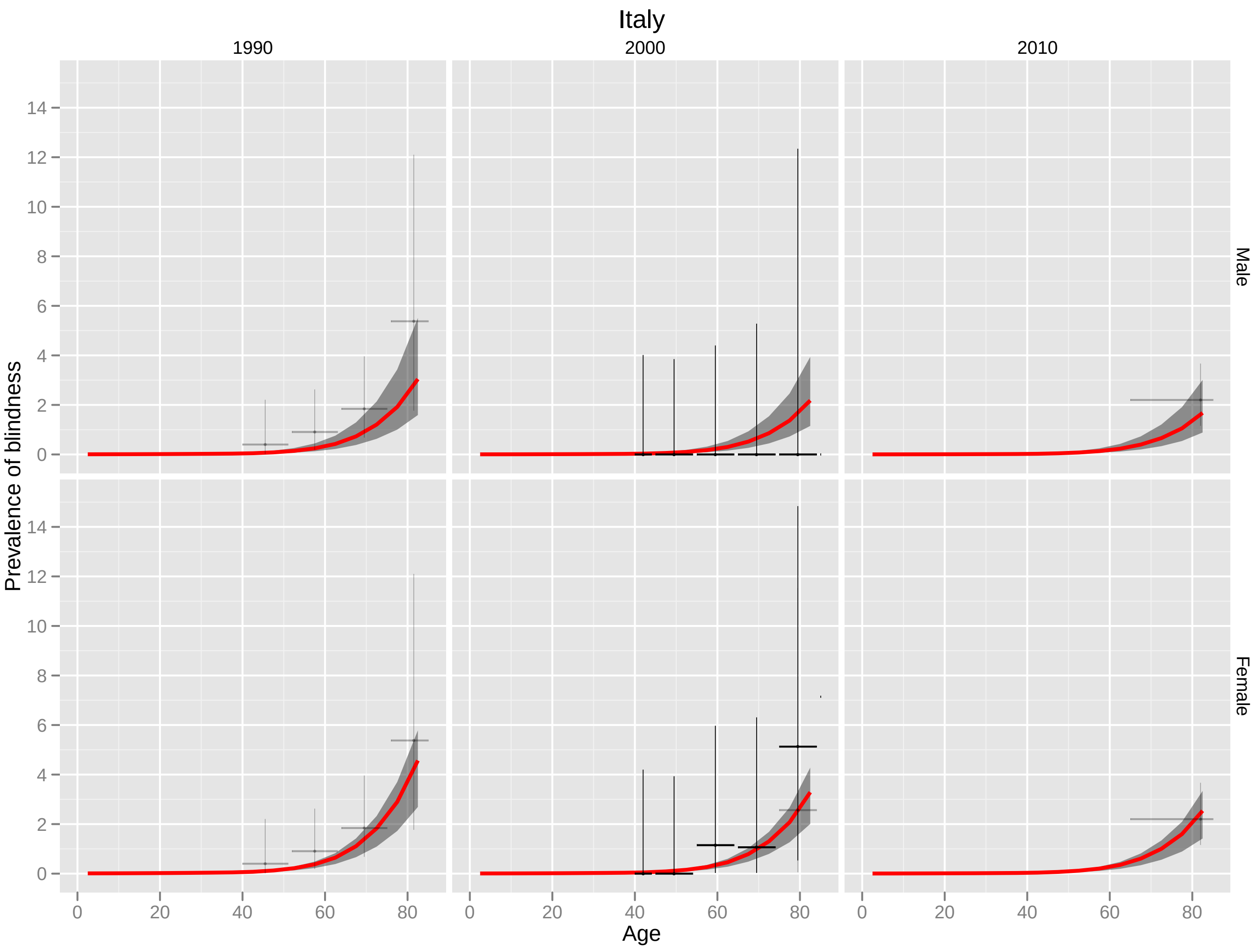
Italy
1990
2000
2010
Male
Female
Prevalence of blindness
Age
0
2
4
6
8
10
12
14
20
40
60
80

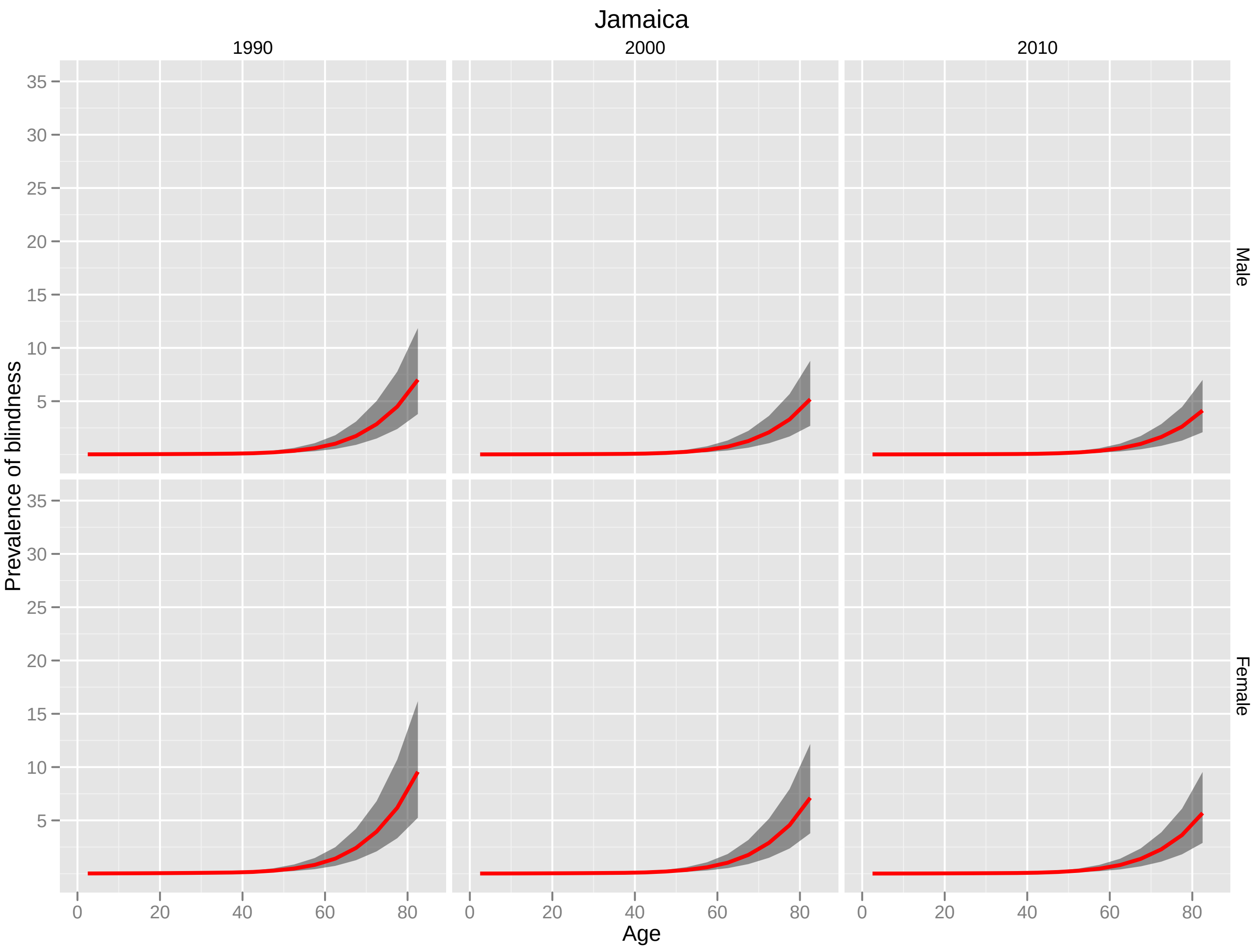
Jamaica
1990
2000
2010
Male
Female
Prevalence of blindness
Age
35
30
25
20
15
10
5
0
20
40
60
80

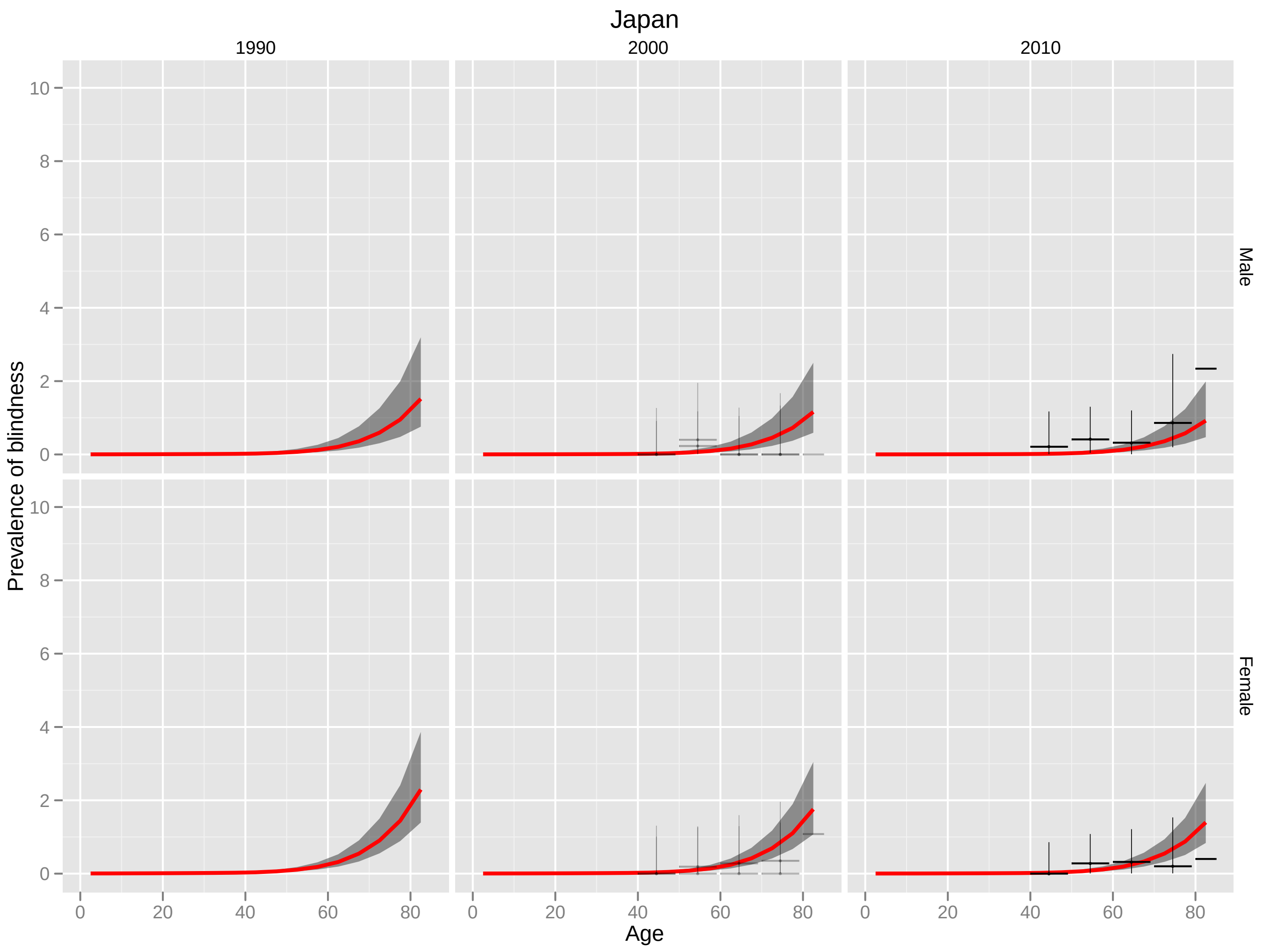

Japan
1990
2000
2010
Male
Female
Prevalence of blindness
Age
0
2
4
6
8
10
0
20
40
60
80

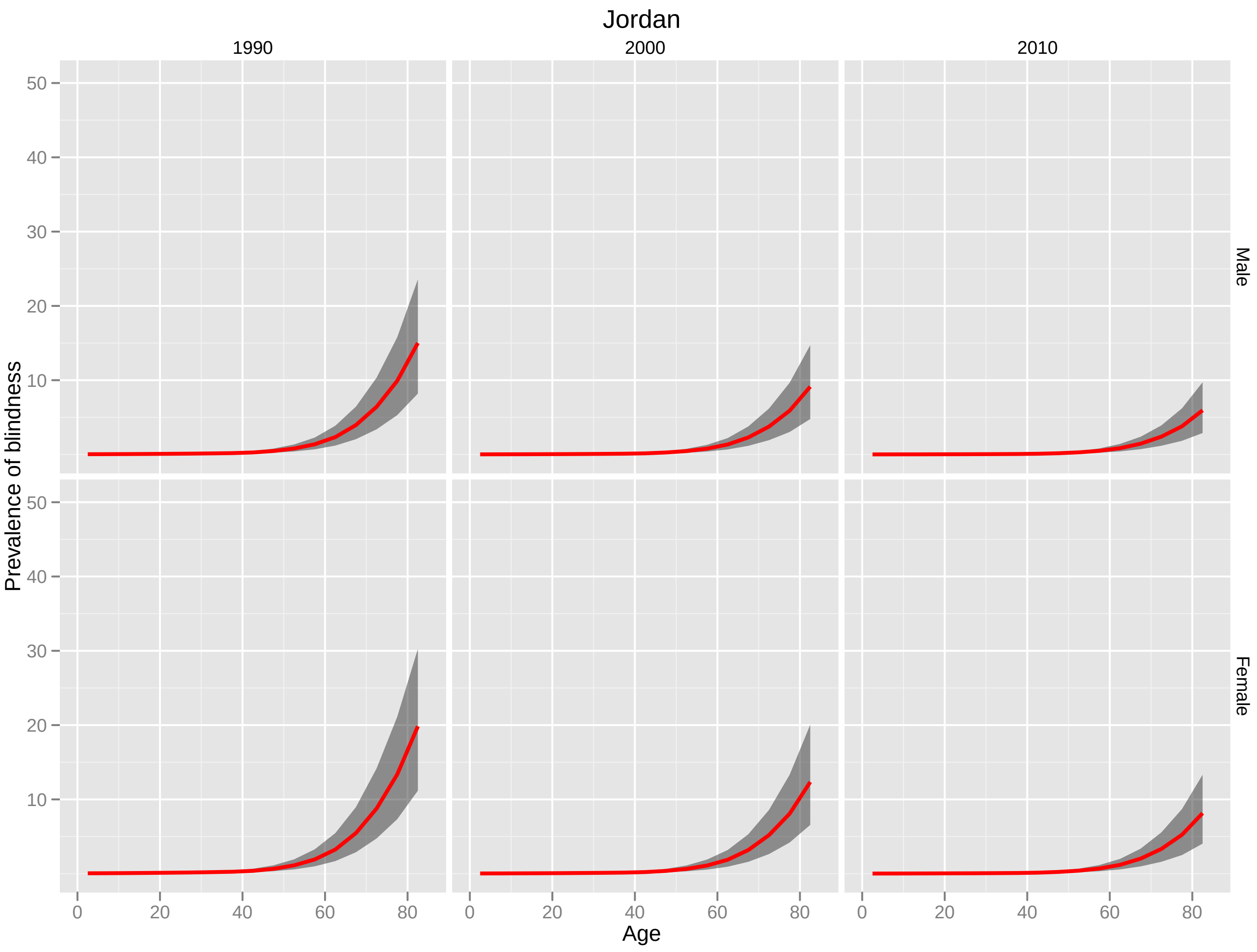
Jordan
1990
2000
2010
Male
Female
Prevalence of blindness
Age
0
20
40
60
80
10
20
30
40
50

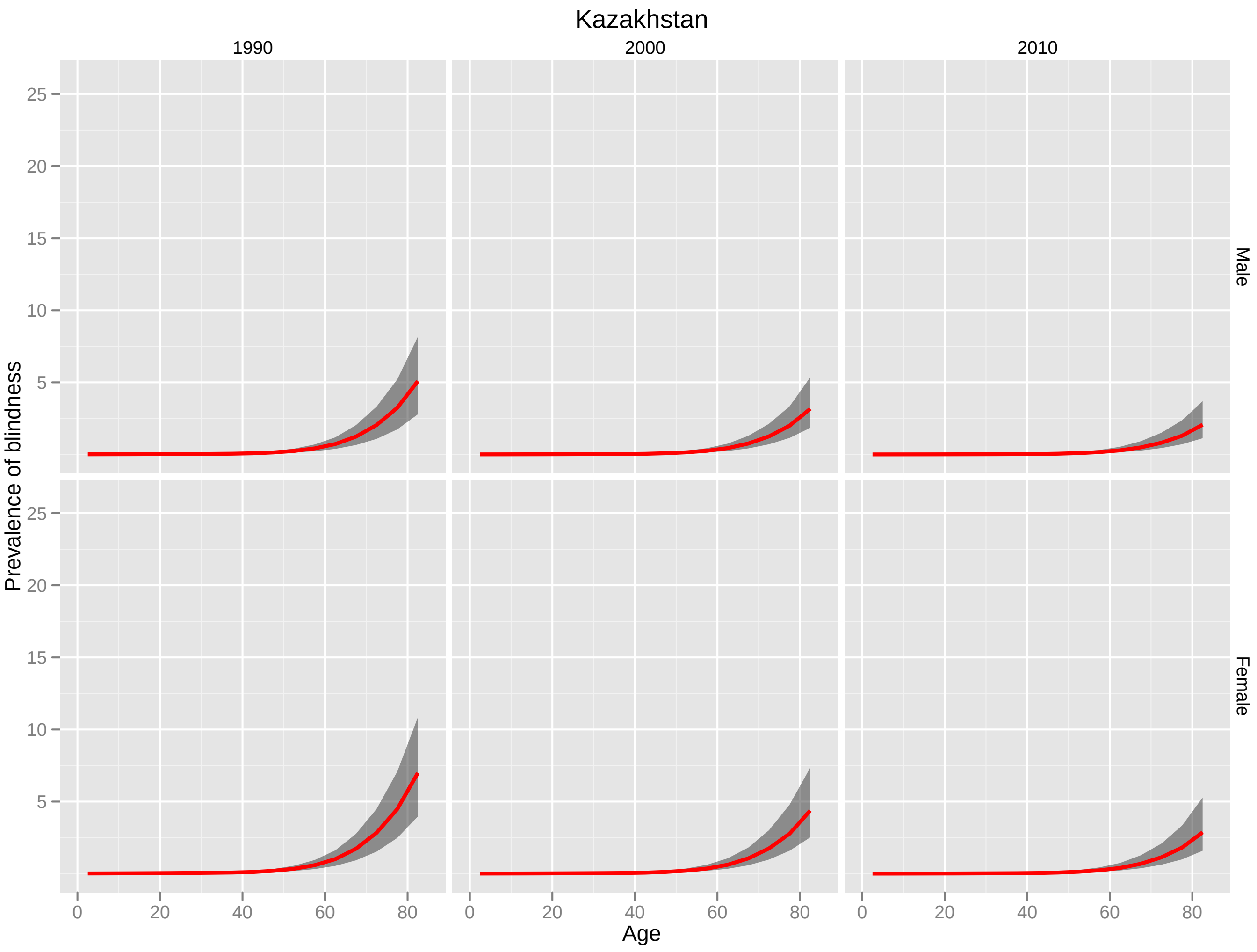
Kazakhstan
1990
2000
2010
Male
Female
Prevalence of blindness
Age
25
20
15
10
5
0
20
40
60
80

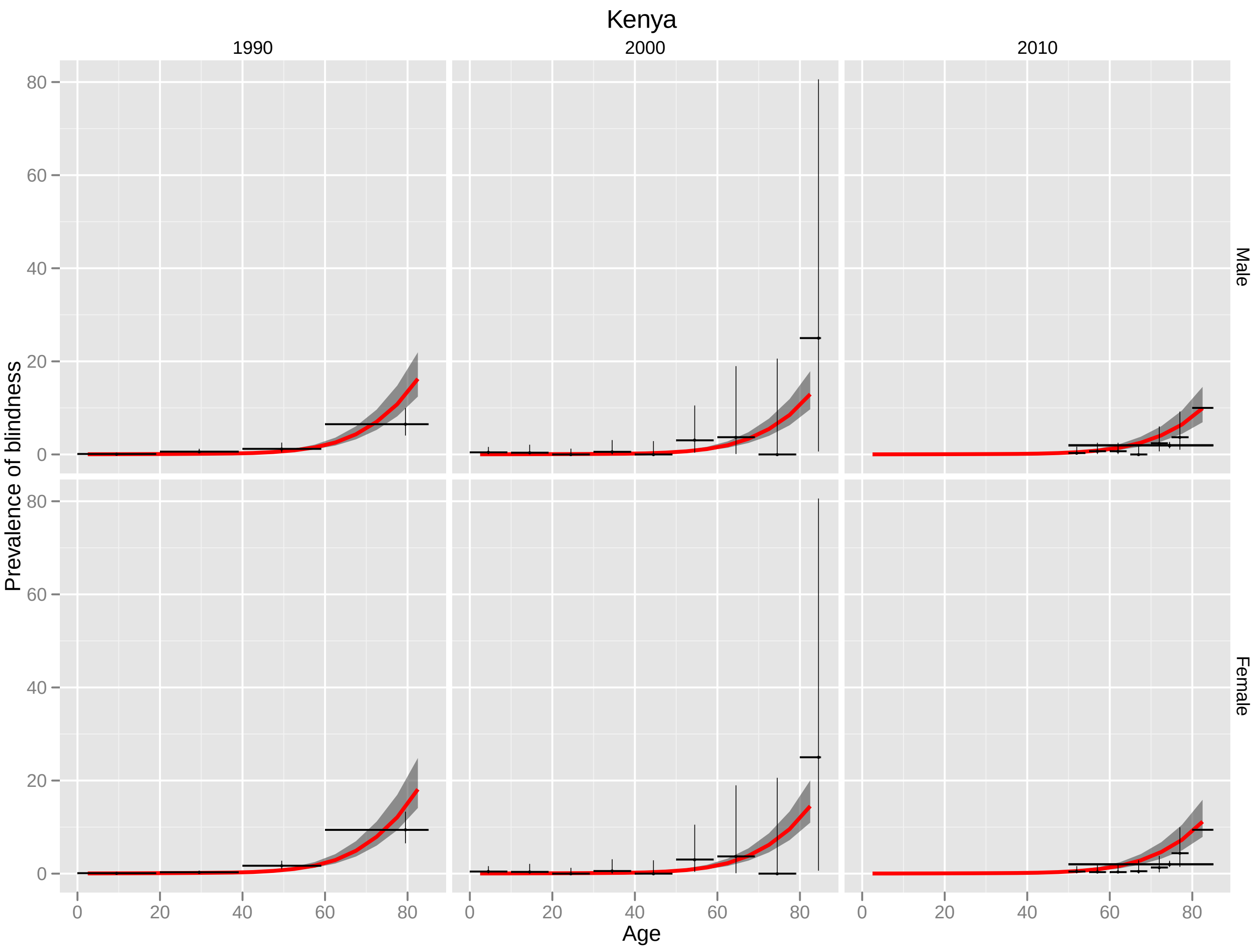

Kenya
1990
2000
2010
Male
Female
Prevalence of blindness
Age
0
20
40
60
80

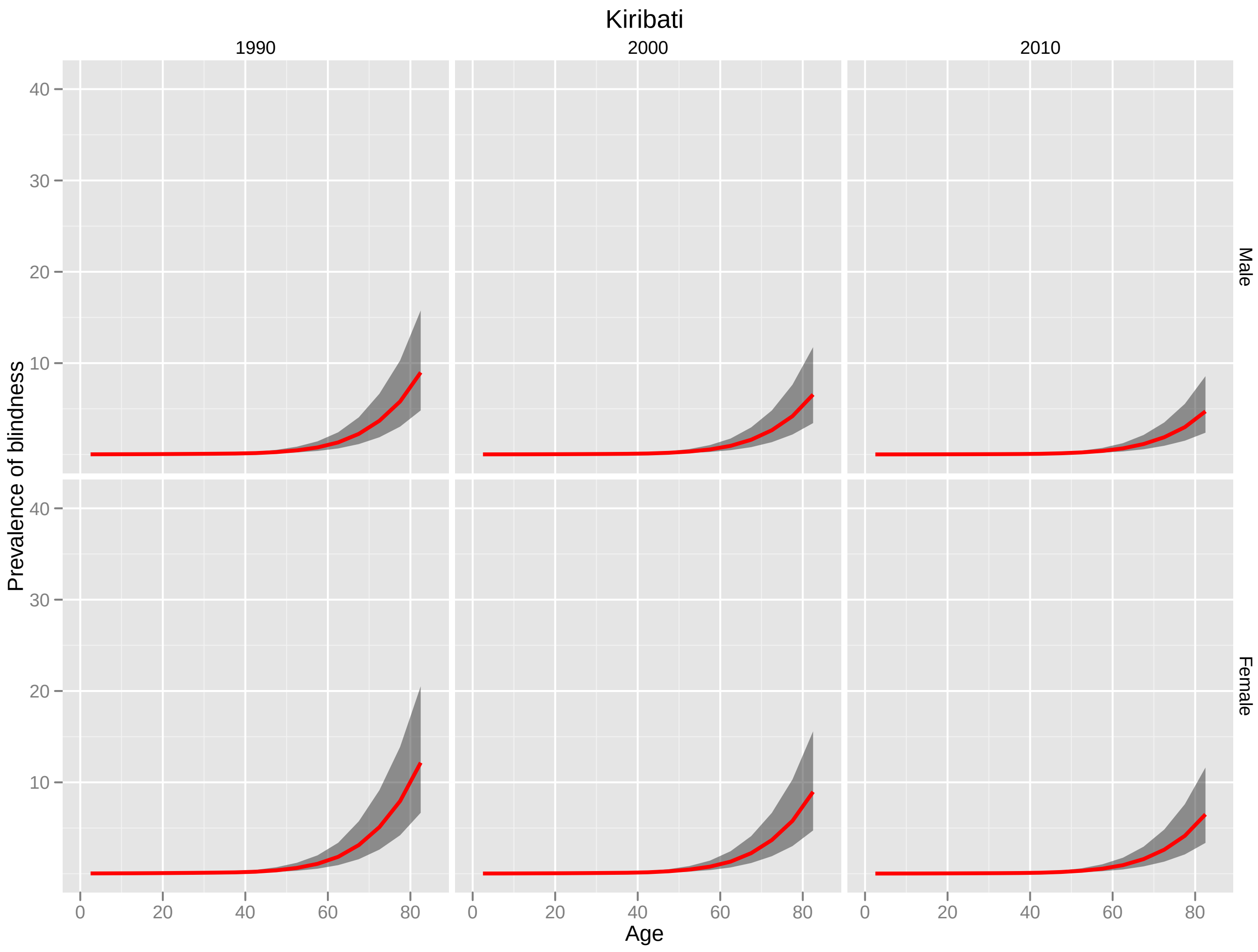
Kiribati
1990
2000
2010
Male
Female
Prevalence of blindness
Age
0
10
20
30
40
60
80

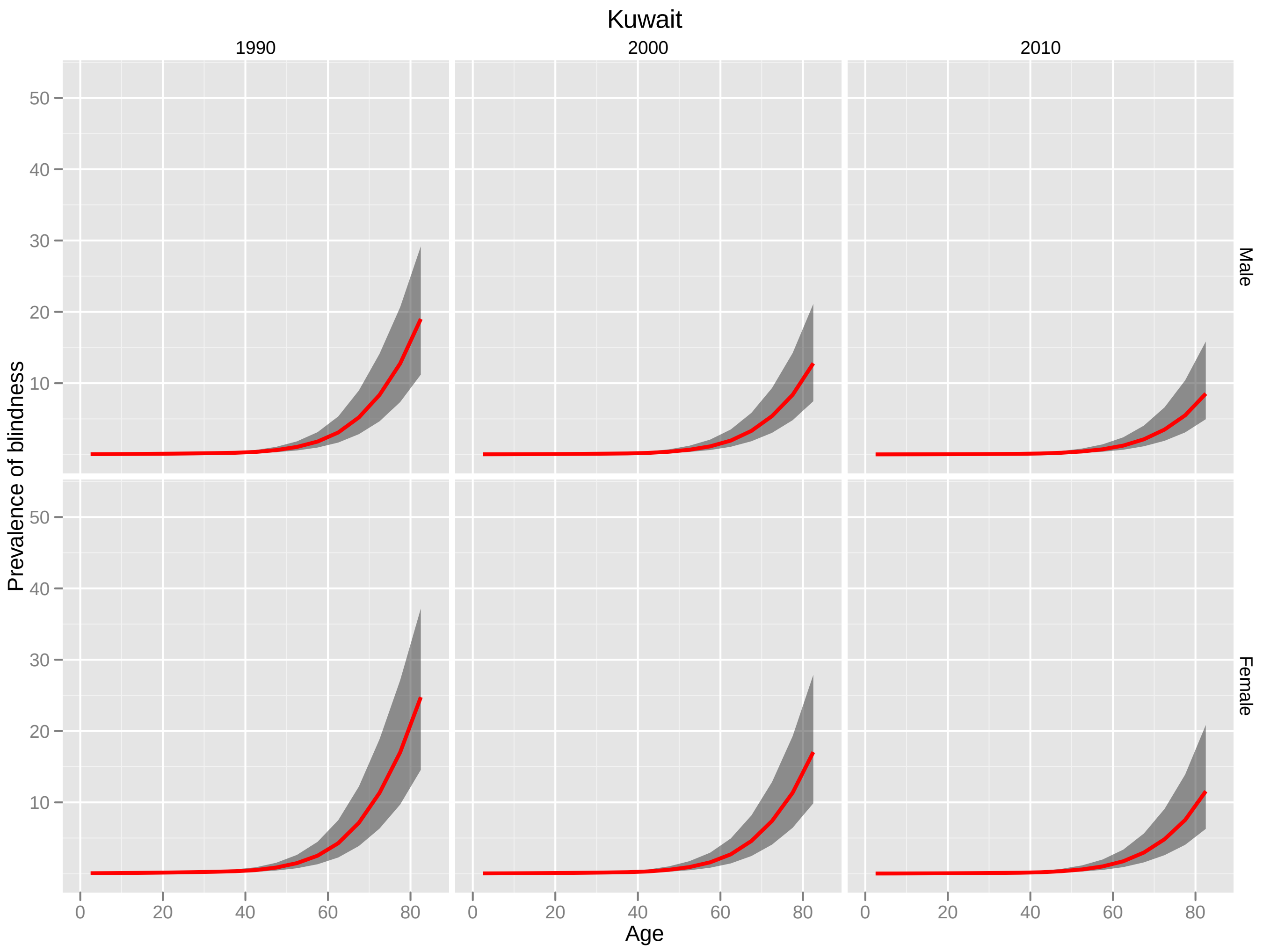

Kuwait
1990
2000
2010
Male
Female
Prevalence of blindness
Age
50
40
30
20
10
0
20
40
60
80

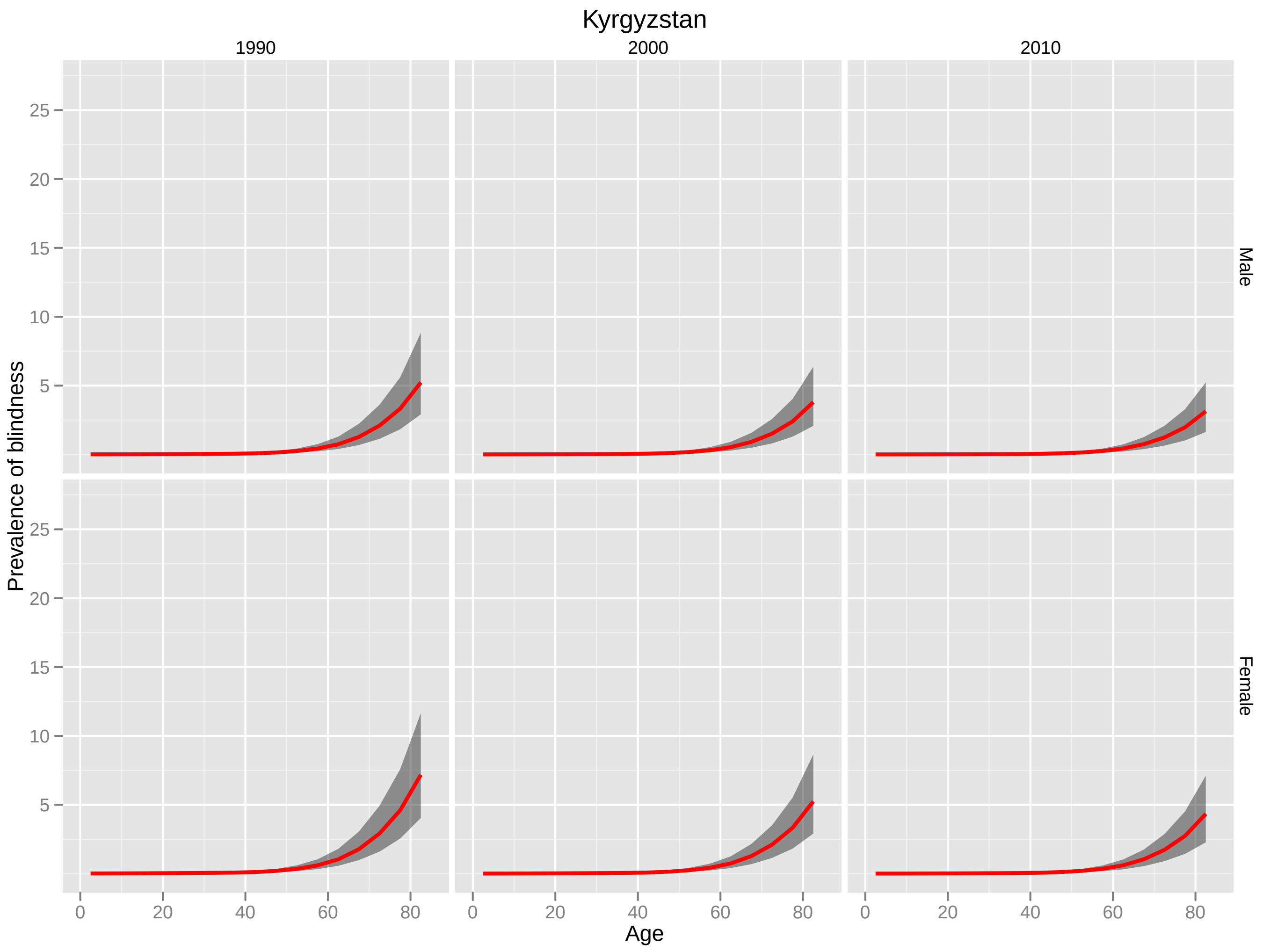
Kyrgyzstan
1990
2000
2010
Male
Female
Prevalence of blindness
5
10
15
20
25
Age
0
20
40
60
80

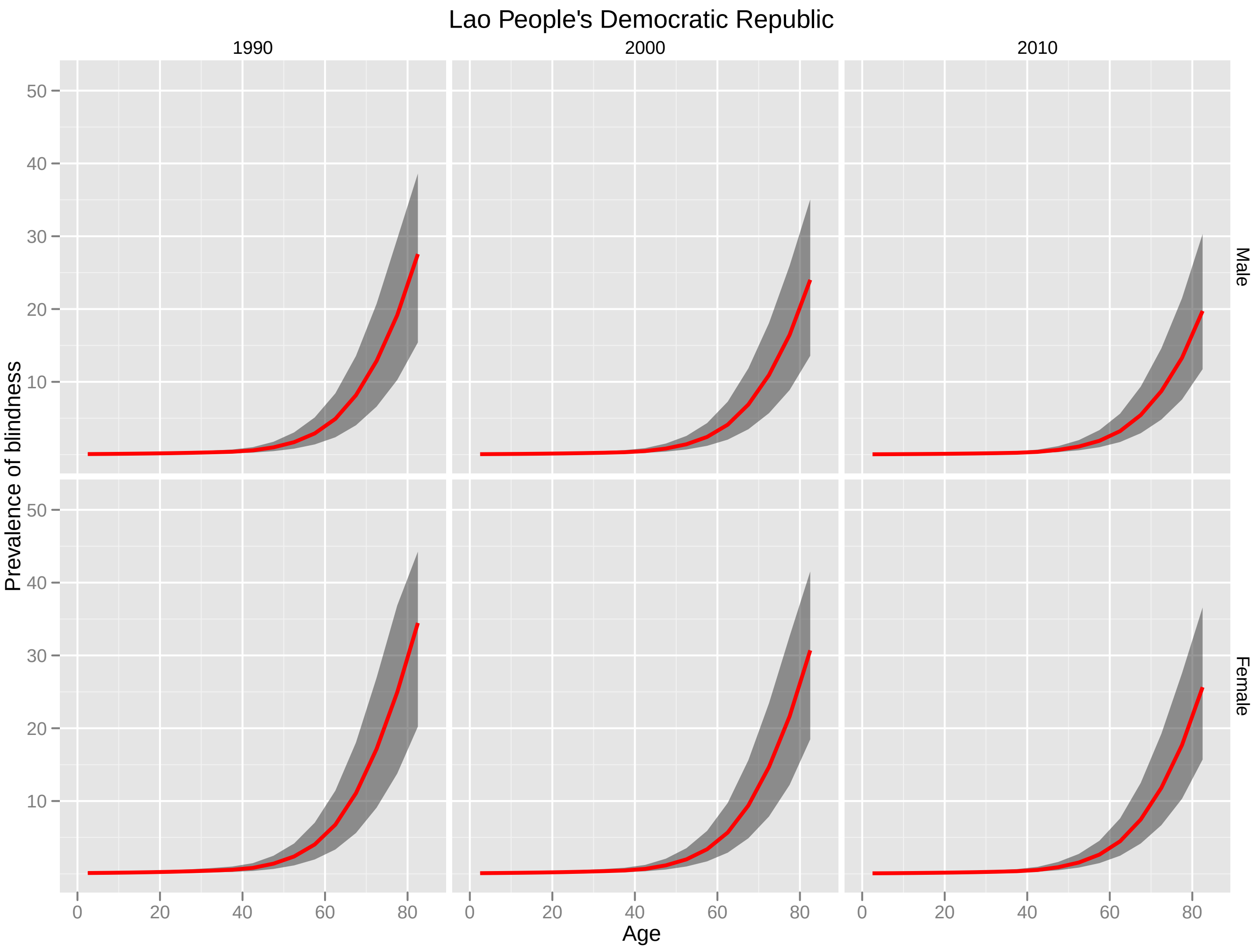
Lao People's Democratic Republic
1990
2000
2010
Male
Female
Prevalence of blindness
Age
10
20
30
40
50
0
20
40
60
80

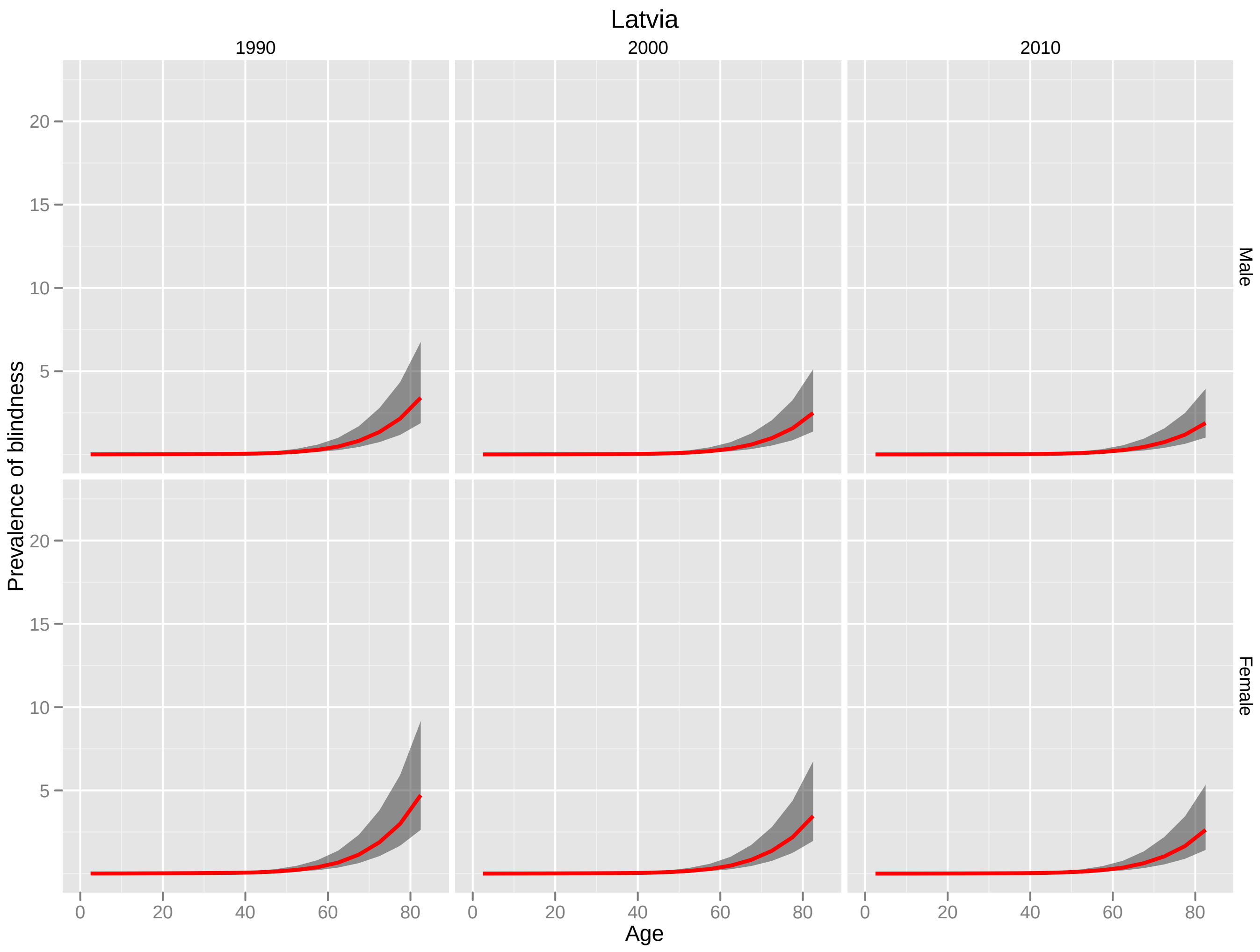
Latvia
1990
2000
2010
Male
Female
Prevalence of blindness
20
15
10
5
Age
0
20
40
60
80

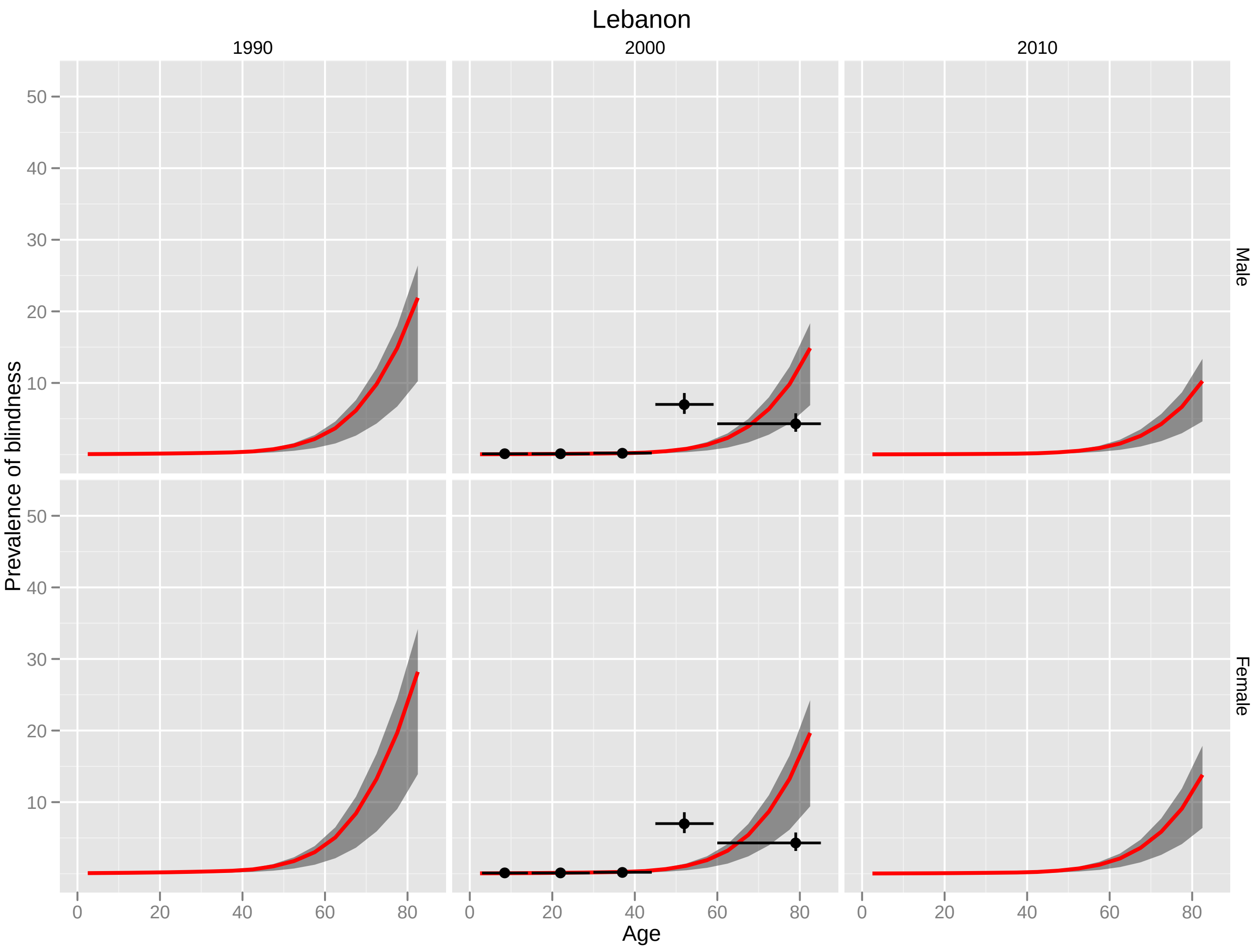
Lebanon
1990
2000
2010
Male
Female
Prevalence of blindness
Age
0
20
40
60
80
10
20
30
40
50

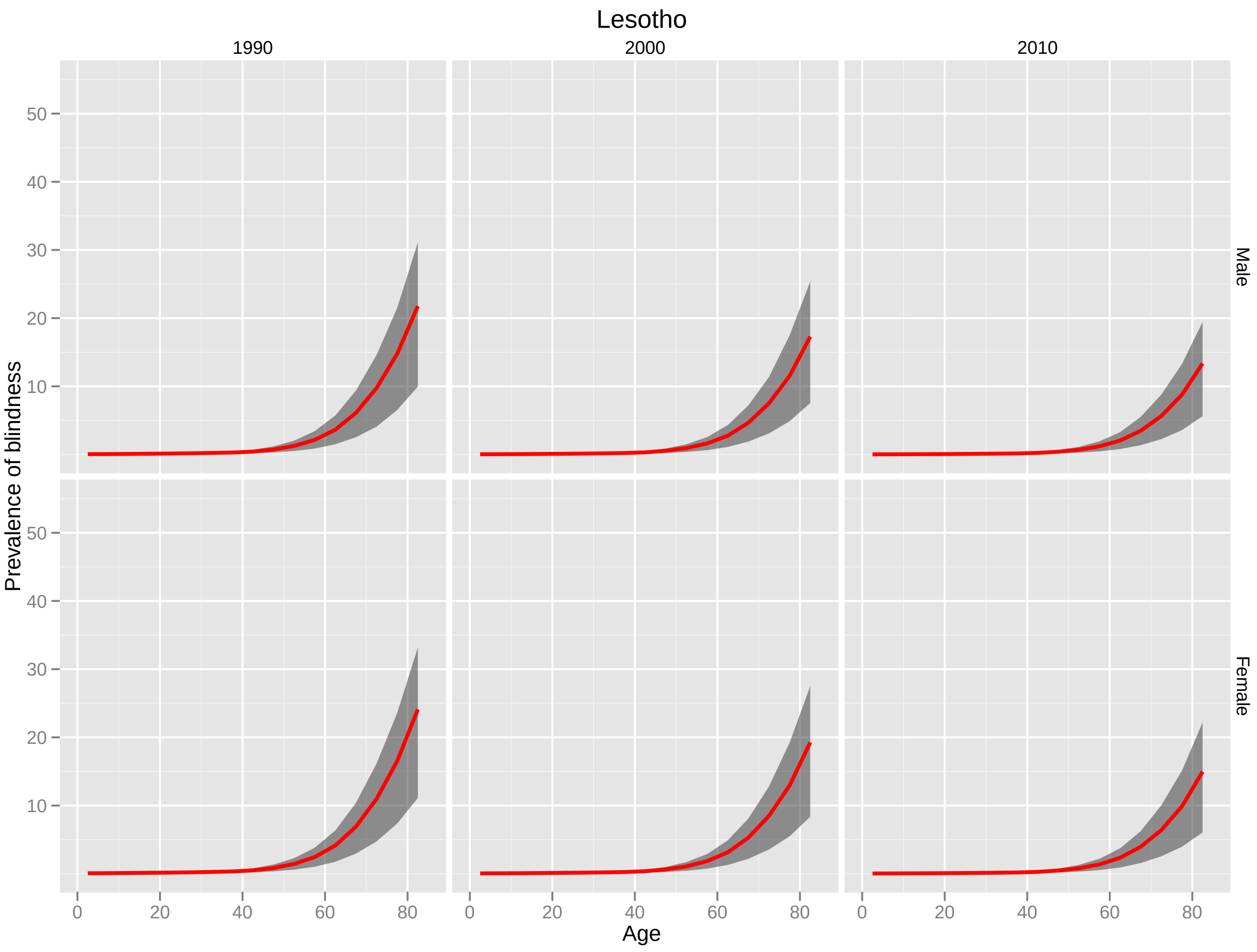
Lesotho
1990
2000
2010
Male
Female
Prevalence of blindness
Age
0
20
40
60
80
10
20
30
40
50

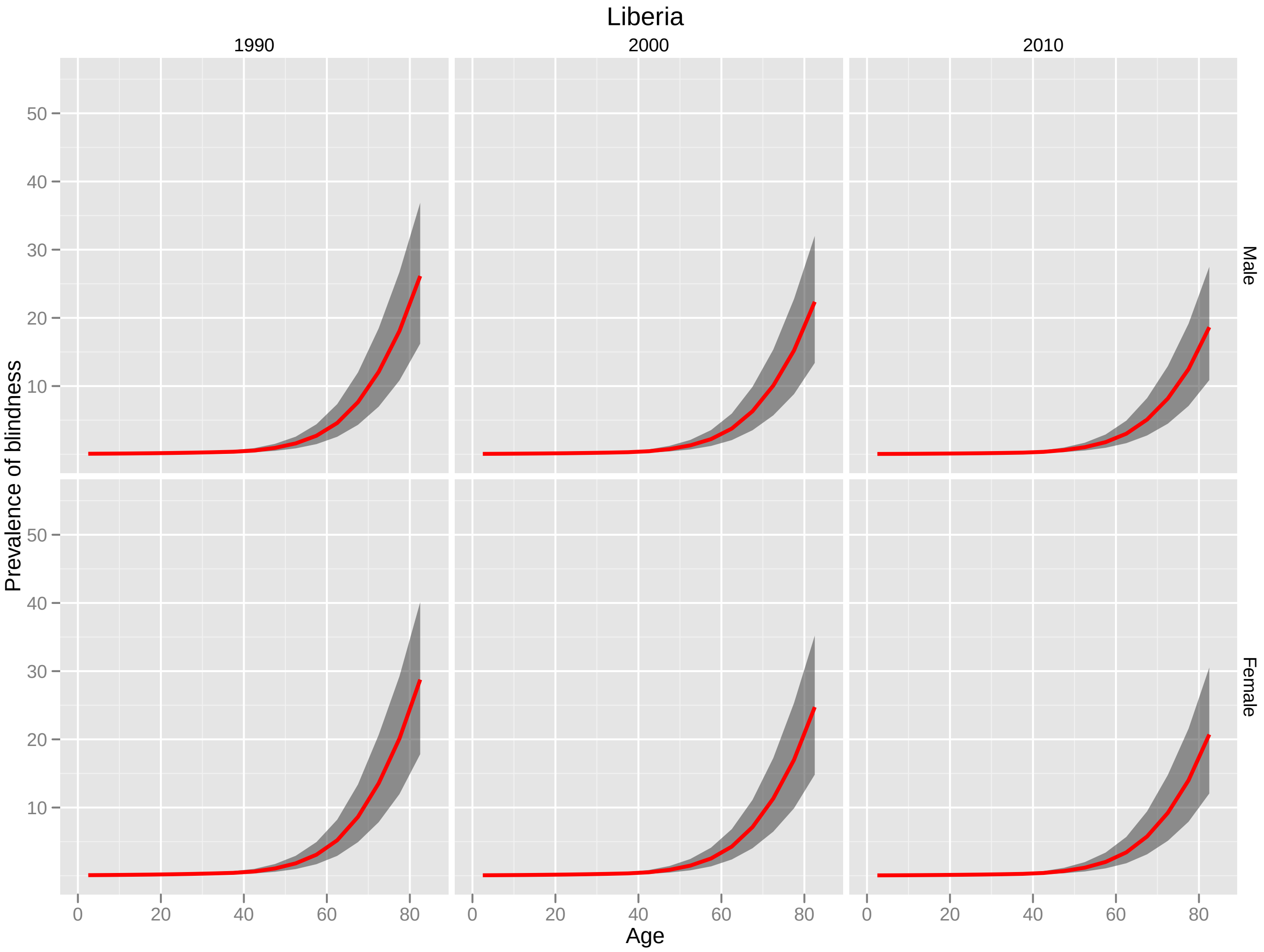
Liberia
1990
2000
2010
Male
Female
Prevalence of blindness
Age
10
20
30
40
50
0
20
40
60
80

Libyan Arab Jamahiriya

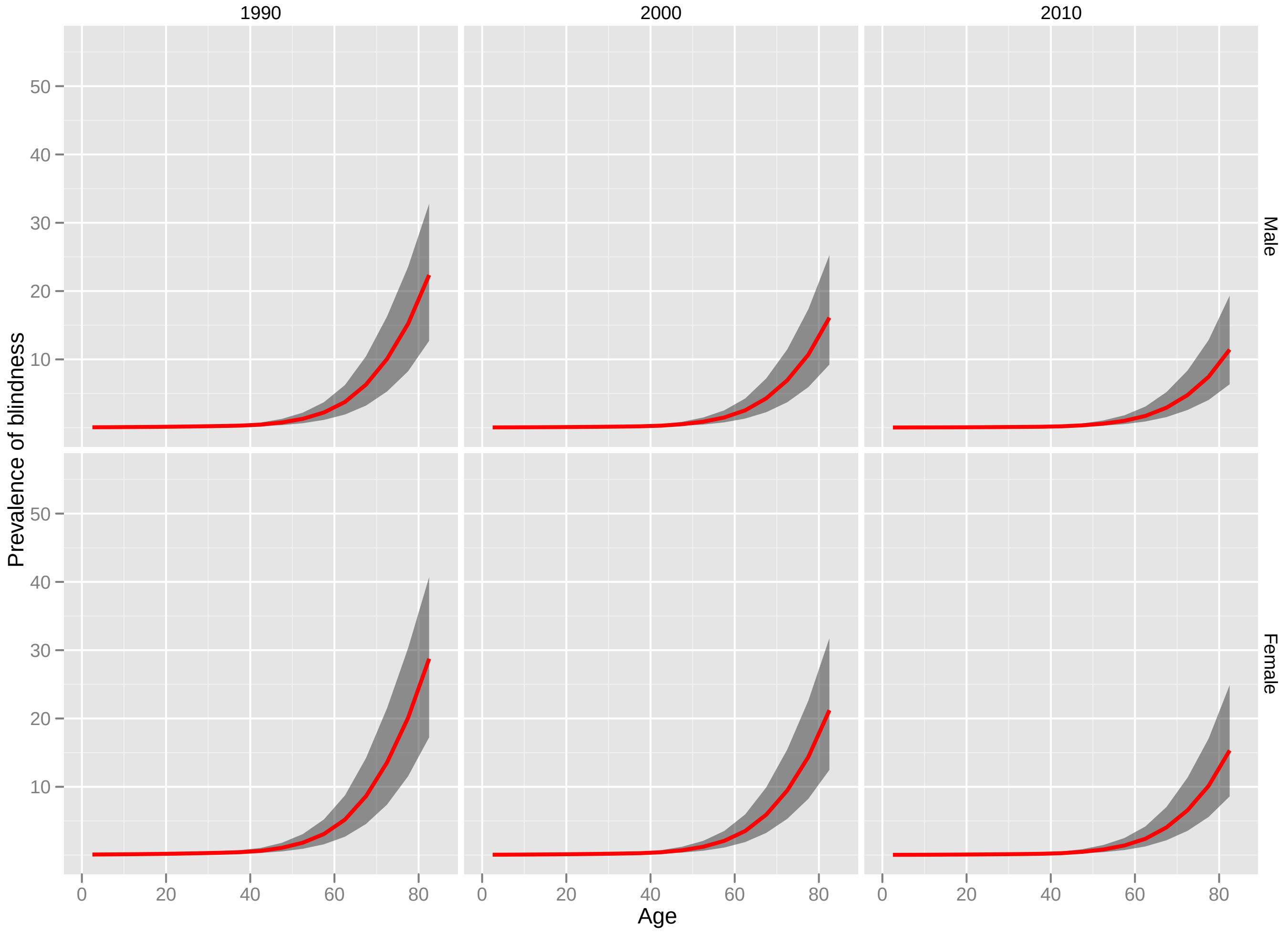

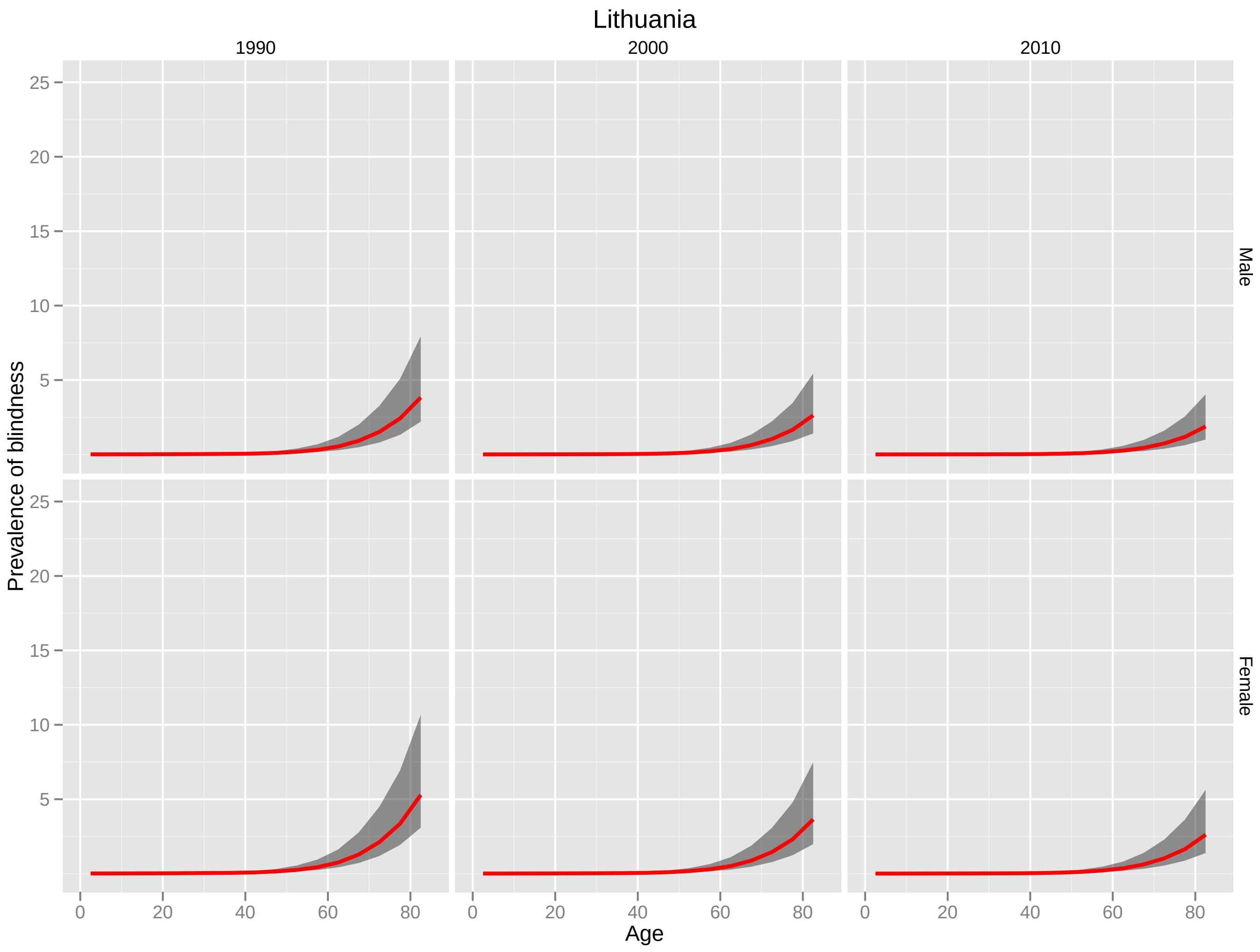

Lithuania
1990
2000
2010
Male
Female
Prevalence of blindness
25
20
15
10
5
0
20
40
60
80
Age

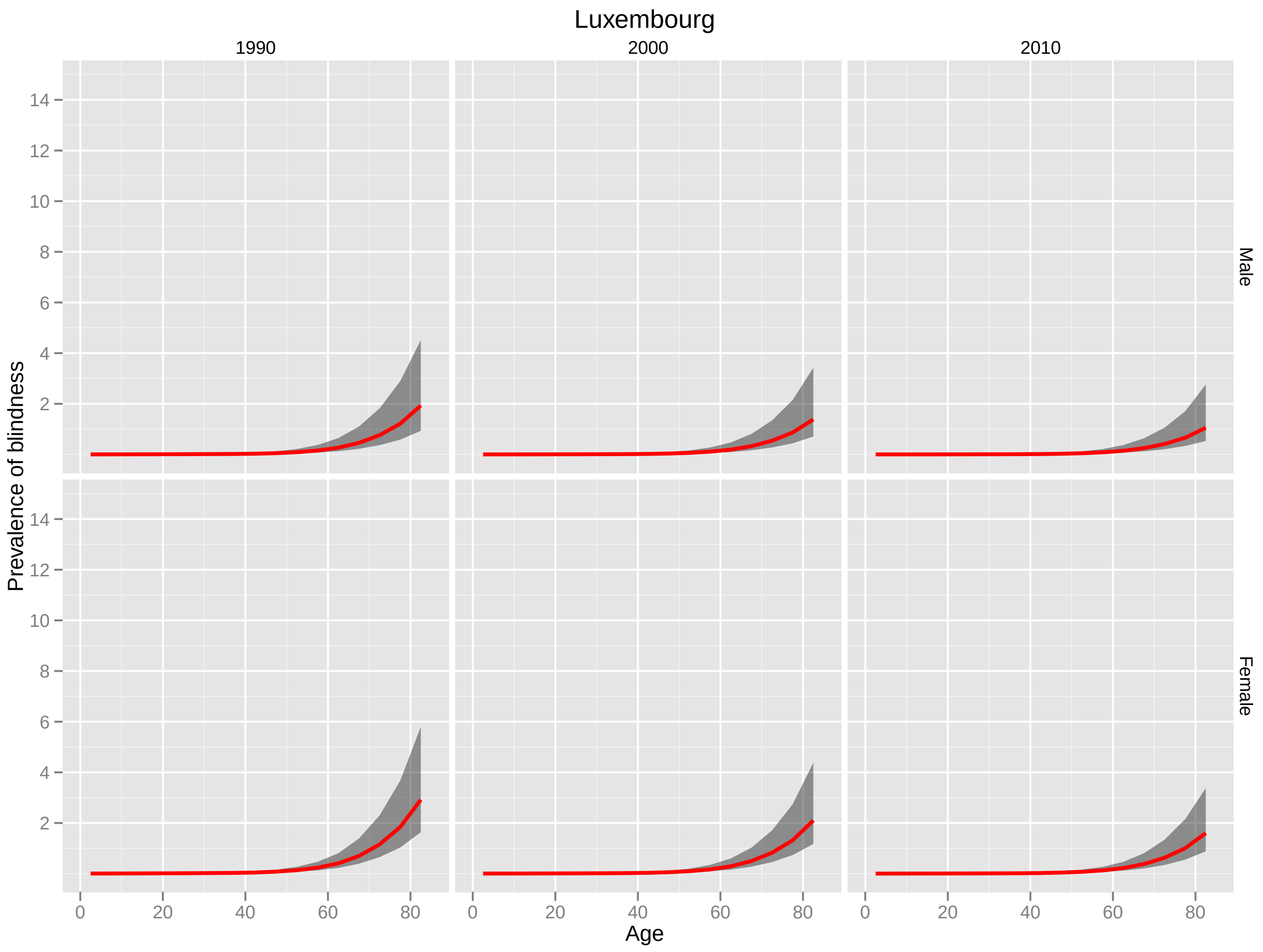
Luxembourg
1990
2000
2010
Male
Female
Prevalence of blindness
Age
14
12
10
8
6
4
2
0
20
40
60
80

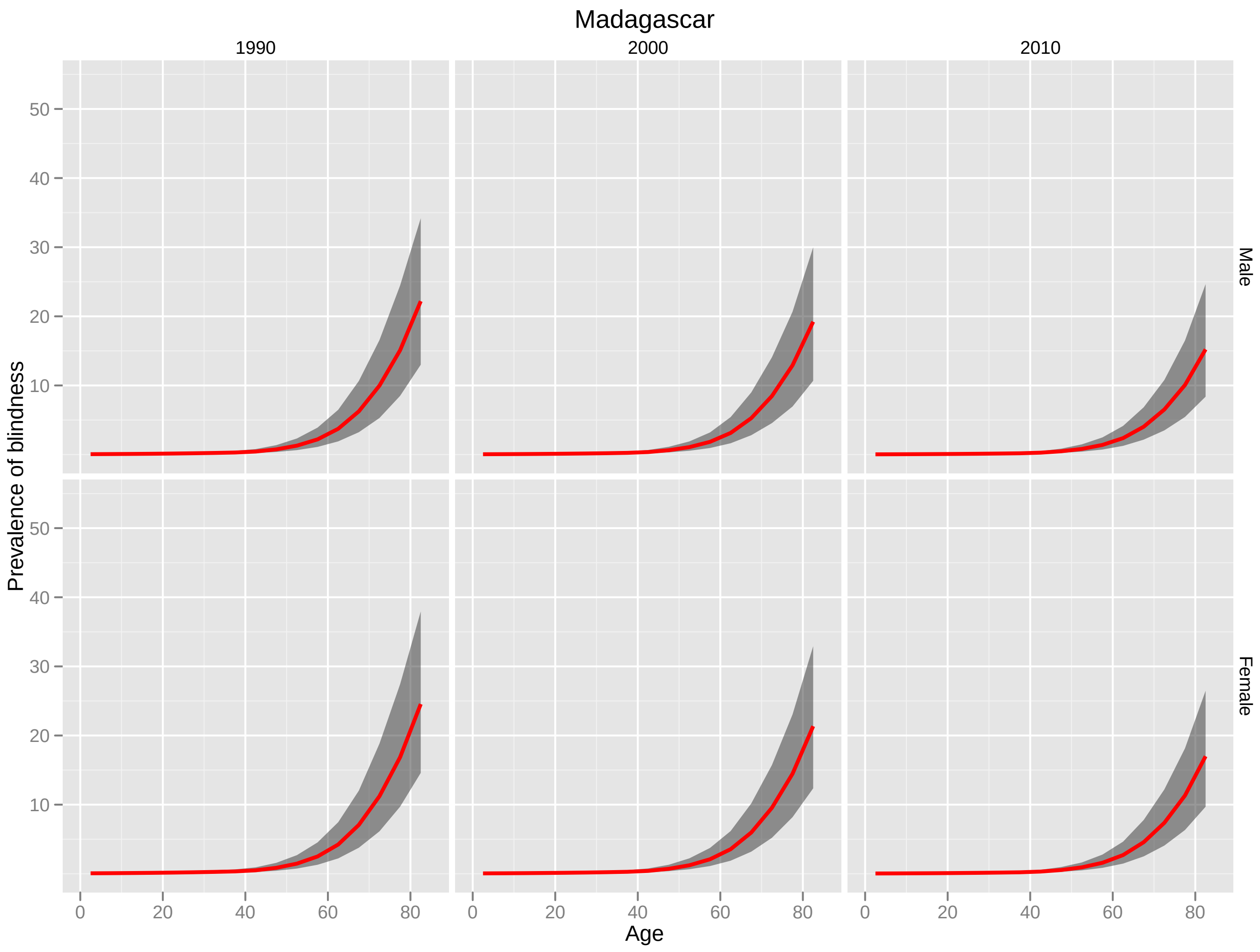
Madagascar
1990
2000
2010
Male
Female
Prevalence of blindness
10
20
30
40
50
0
20
40
60
80
Age

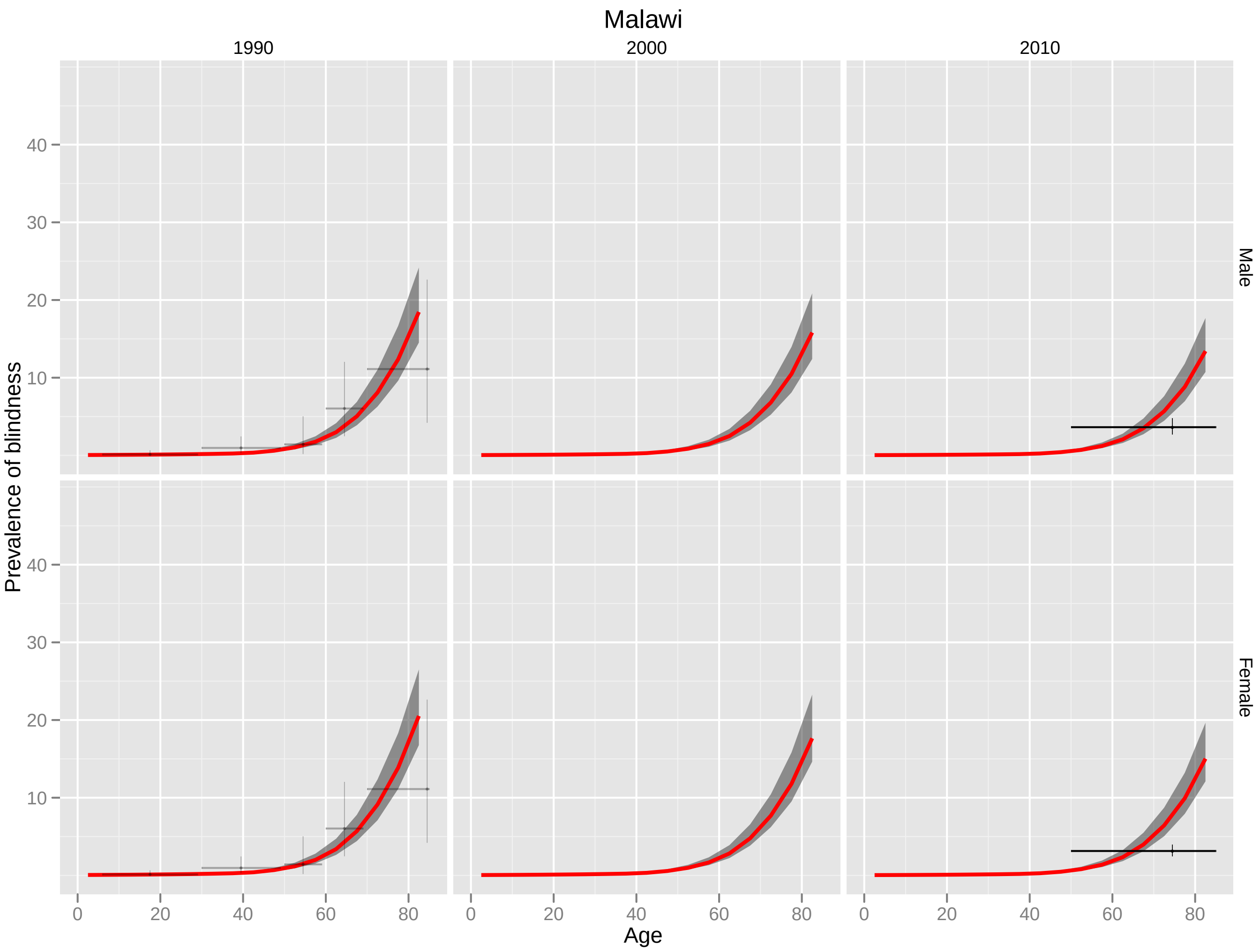
Malawi
1990
2000
2010
Male
Female
Prevalence of blindness
Age
10
20
30
40
0
20
40
60
80

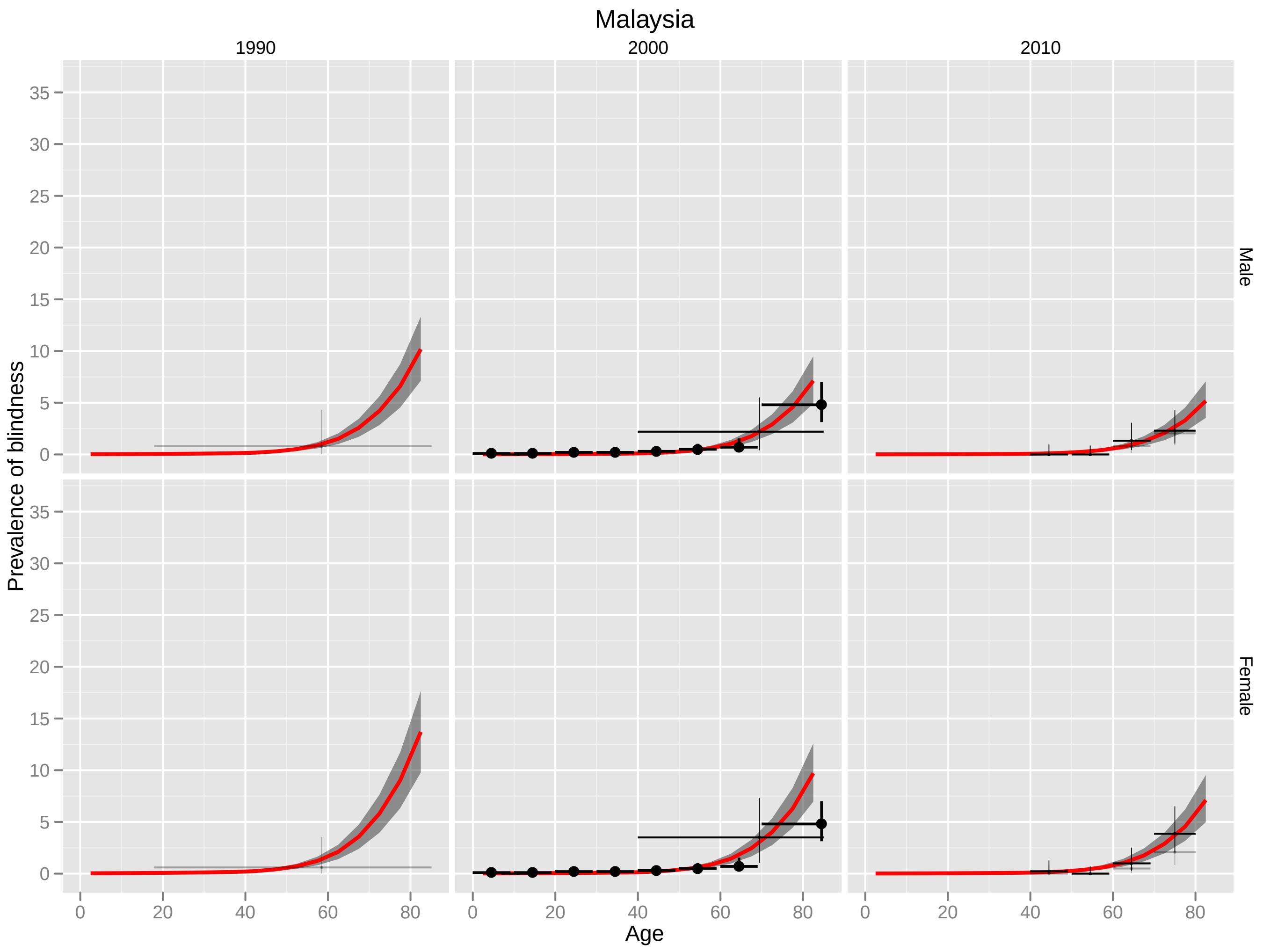
Malaysia
1990
2000
2010
Male
Female
Prevalence of blindness
0
5
10
15
20
25
30
35
Age
0
20
40
60
80

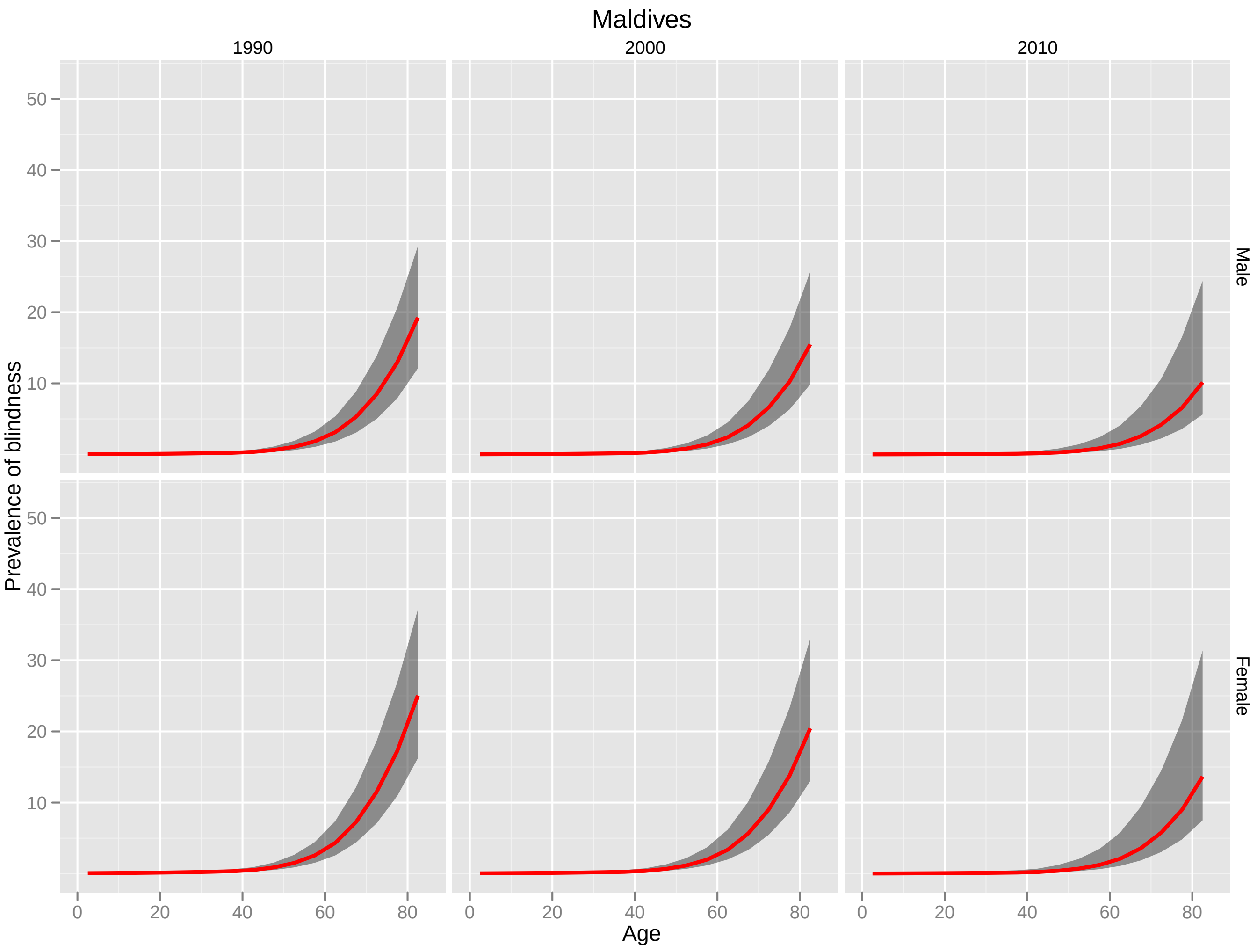

Maldives
1990
2000
2010
Male
Female
Prevalence of blindness
Age
10
20
30
40
50
0
20
40
60
80

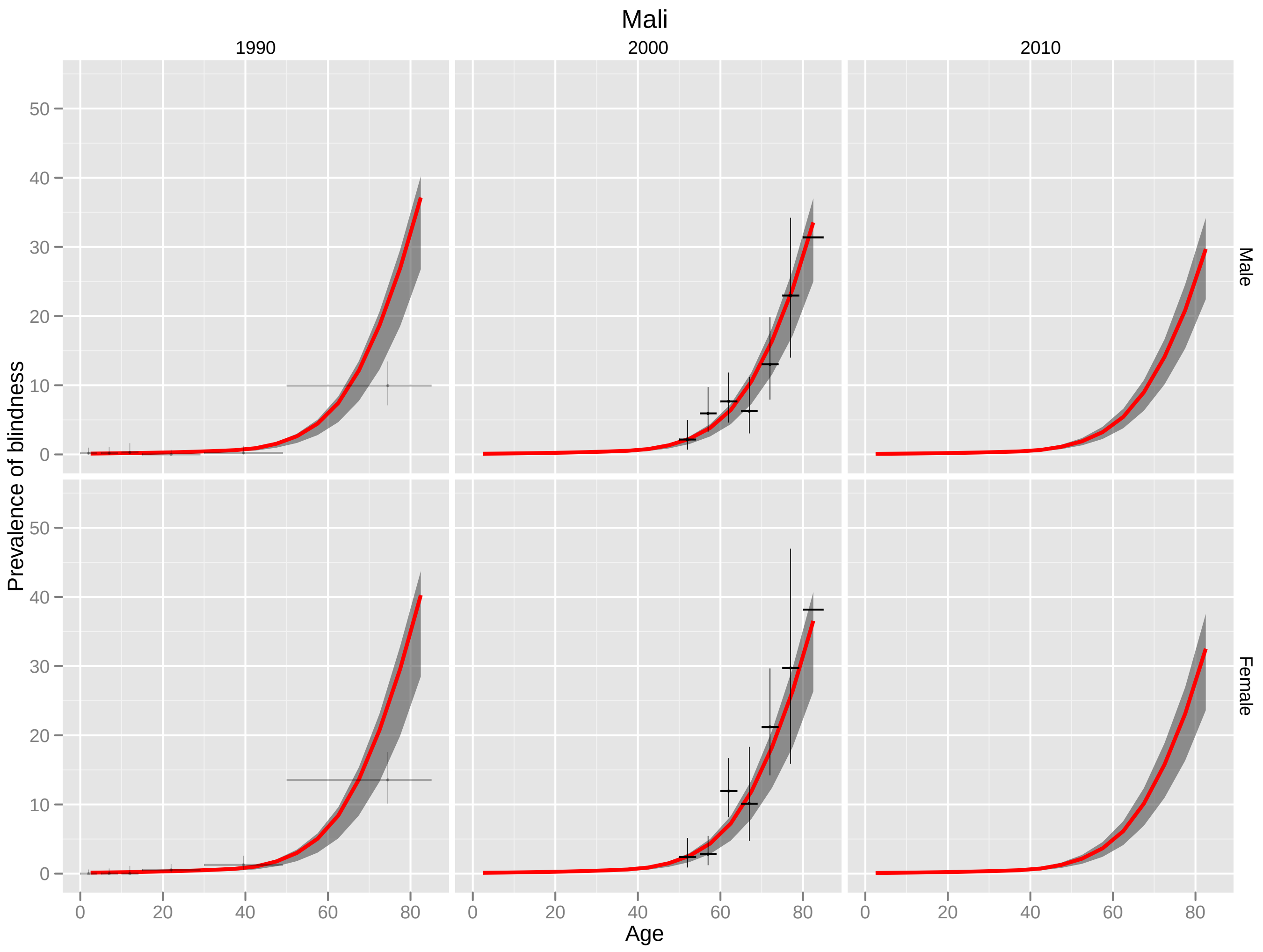
Mali
1990
2000
2010
Male
Female
Prevalence of blindness
Age
0
10
20
30
40
50
0
20
40
60
80

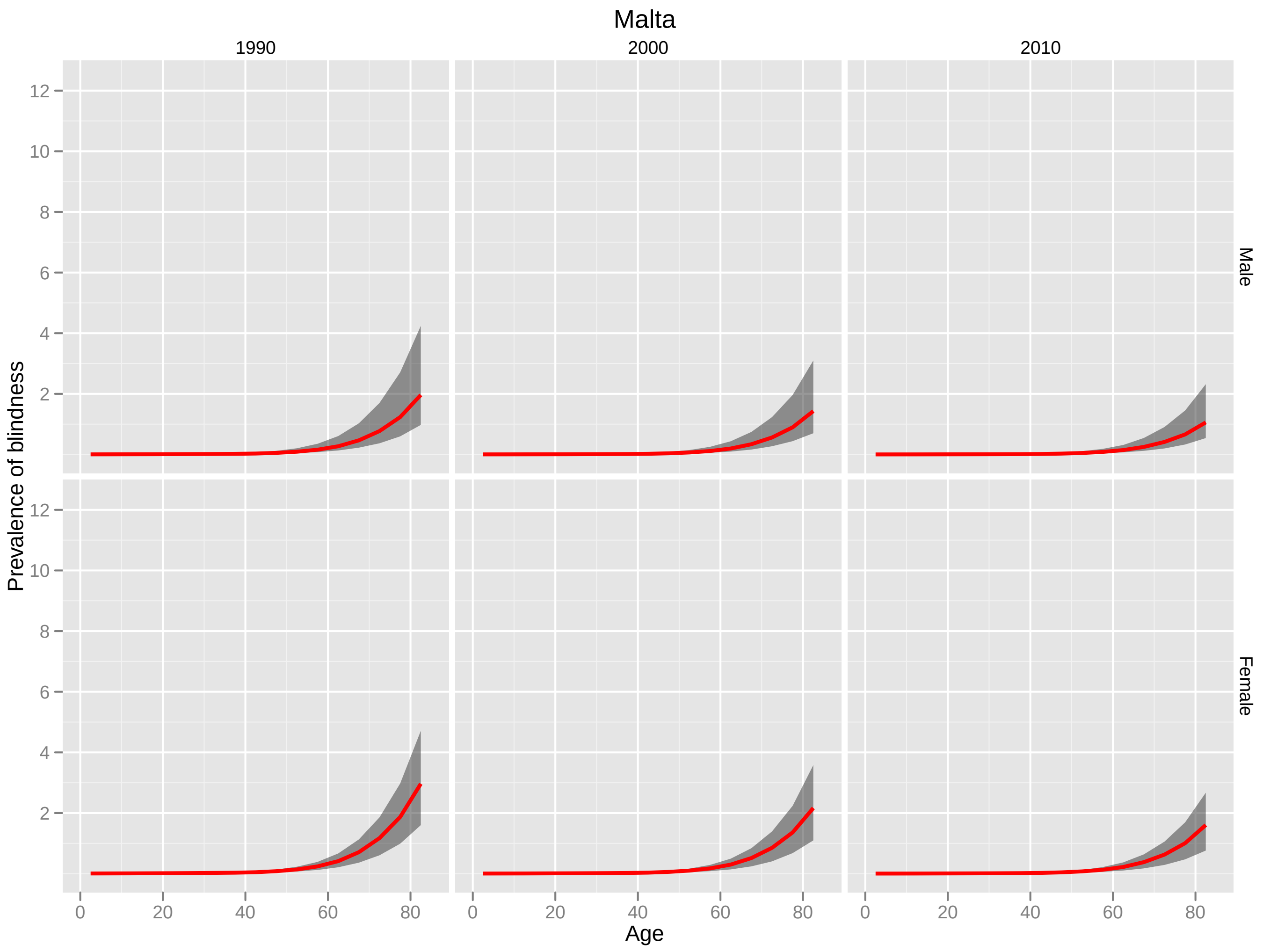
Malta
1990
2000
2010
Male
Female
Prevalence of blindness
Age
12
10
8
6
4
2
0
20
40
60
80

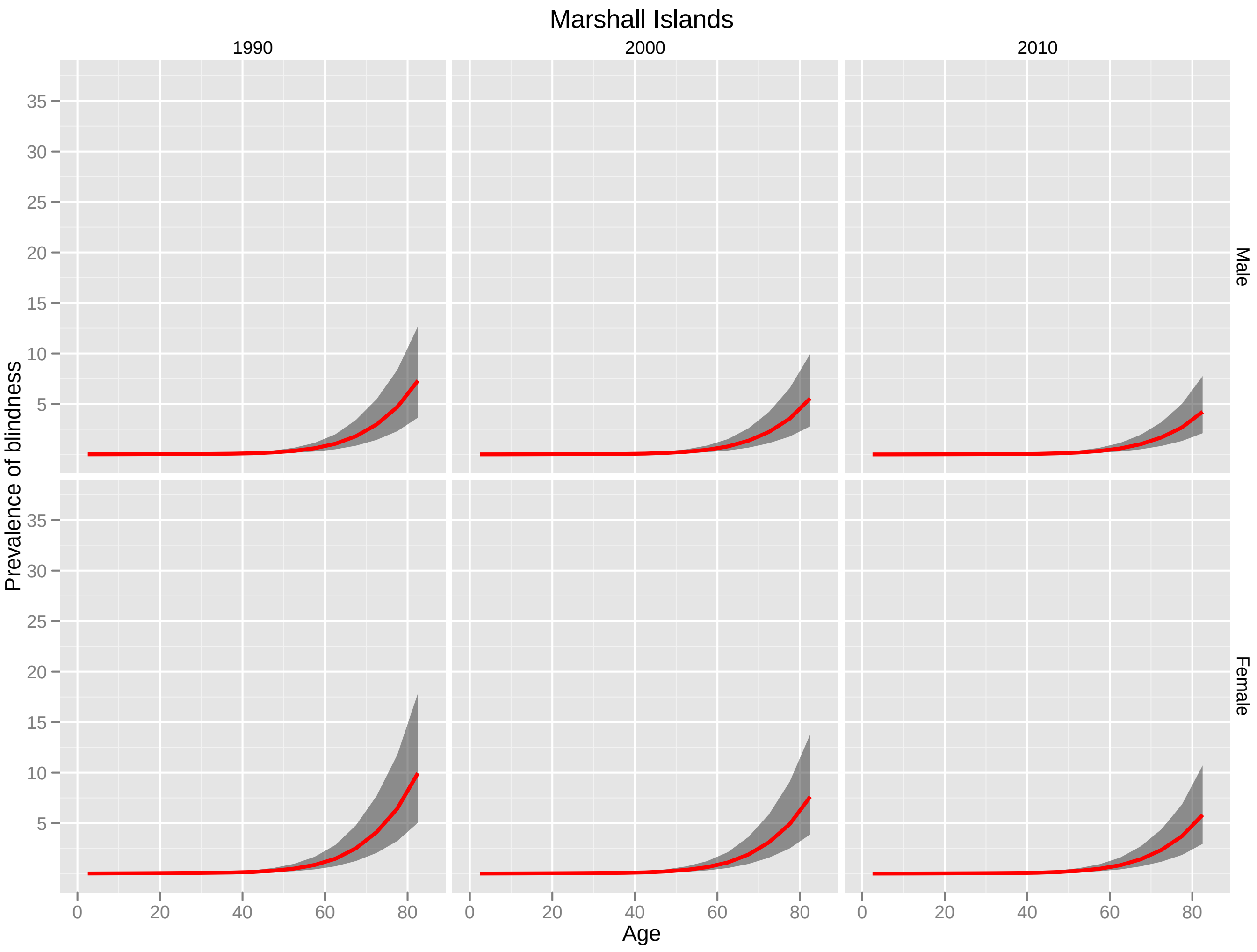

Marshall Islands
1990
2000
2010
Male
Female
Prevalence of blindness
Age
0
20
40
60
80
5
10
15
20
25
30
35

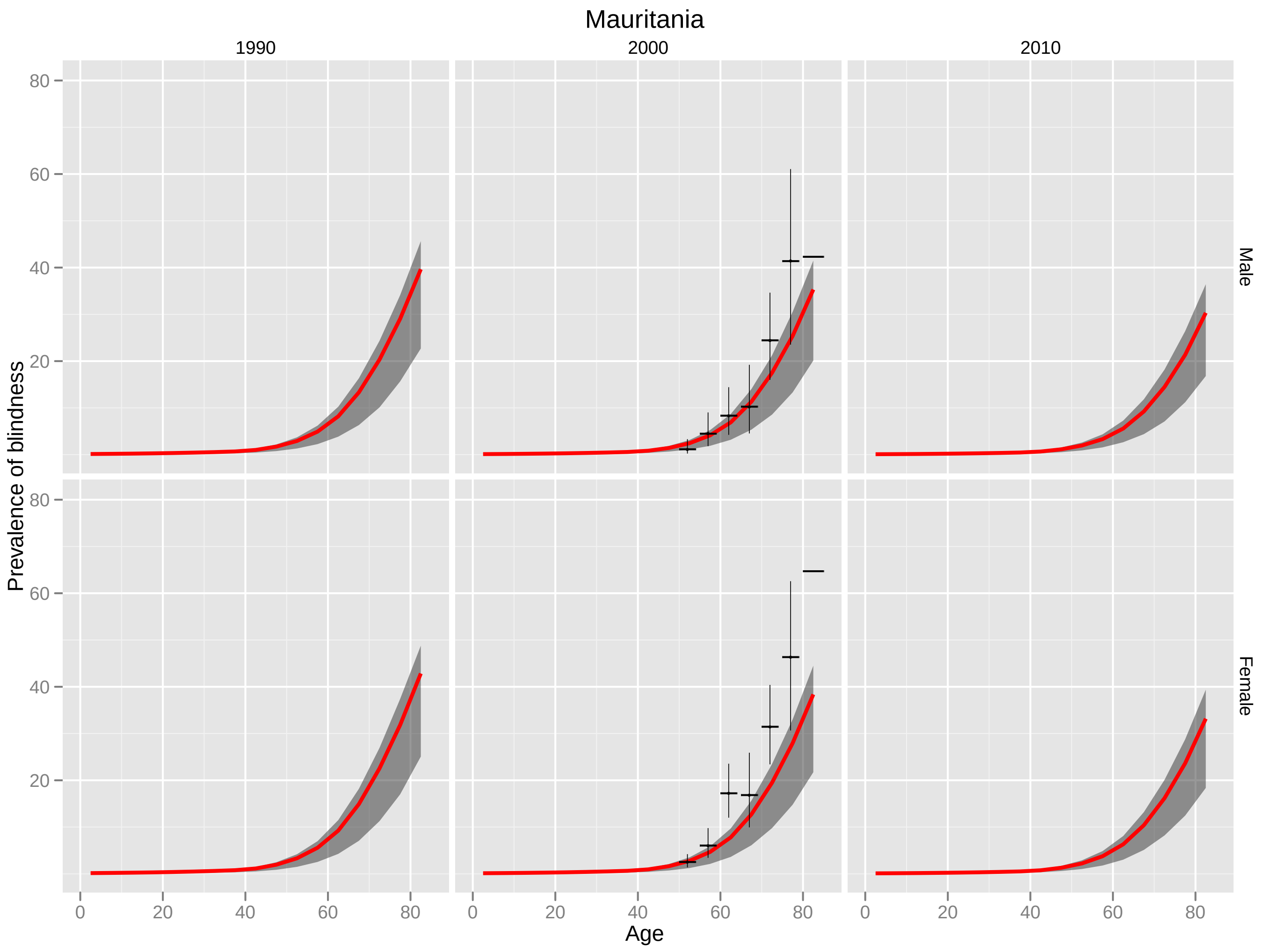
Mauritania
1990
2000
2010
Male
Female
Prevalence of blindness
Age
0
20
40
60
80

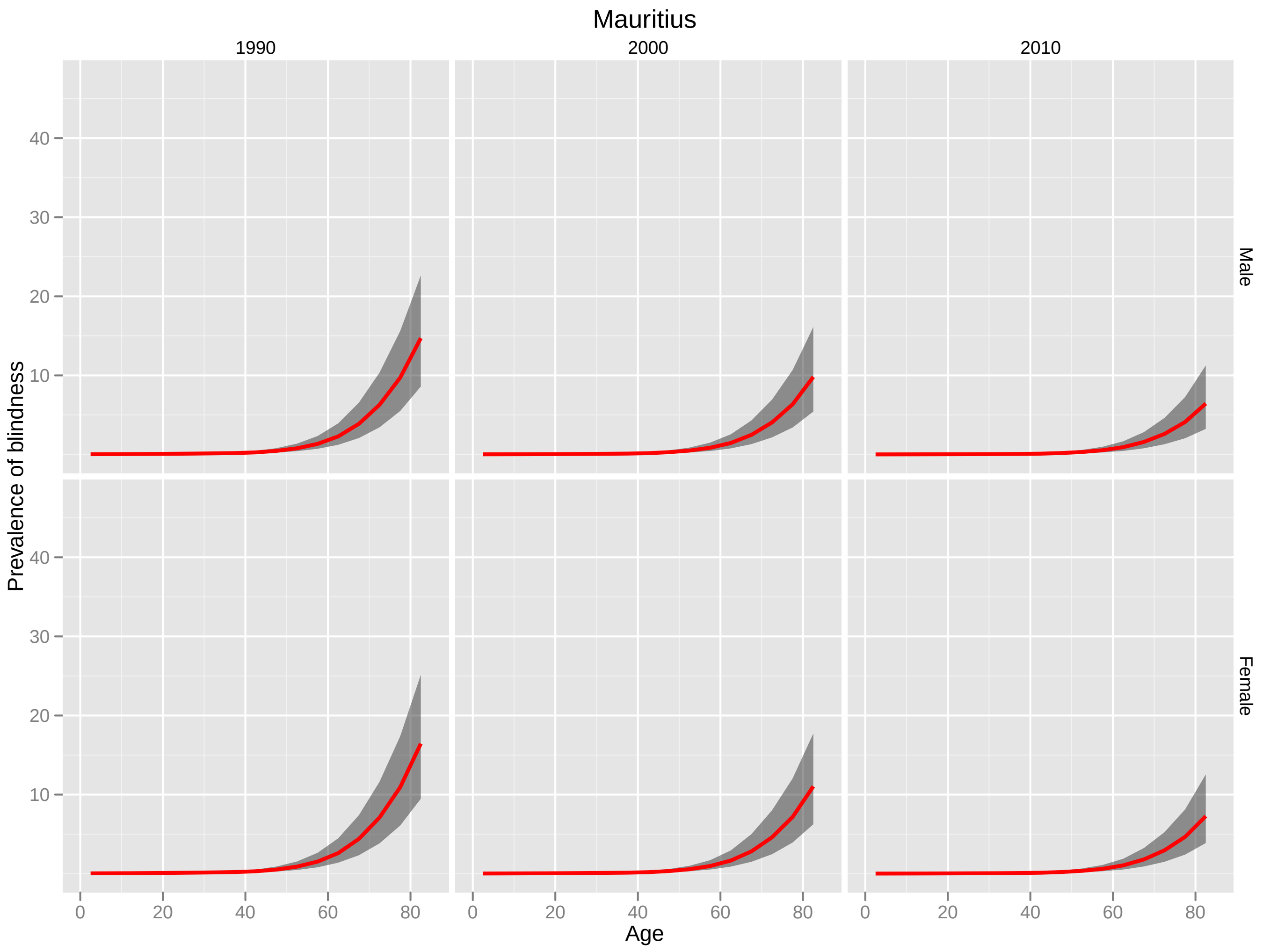
Mauritius
1990
2000
2010
Male
Female
Prevalence of blindness
10
20
30
40
0
20
40
60
80
Age

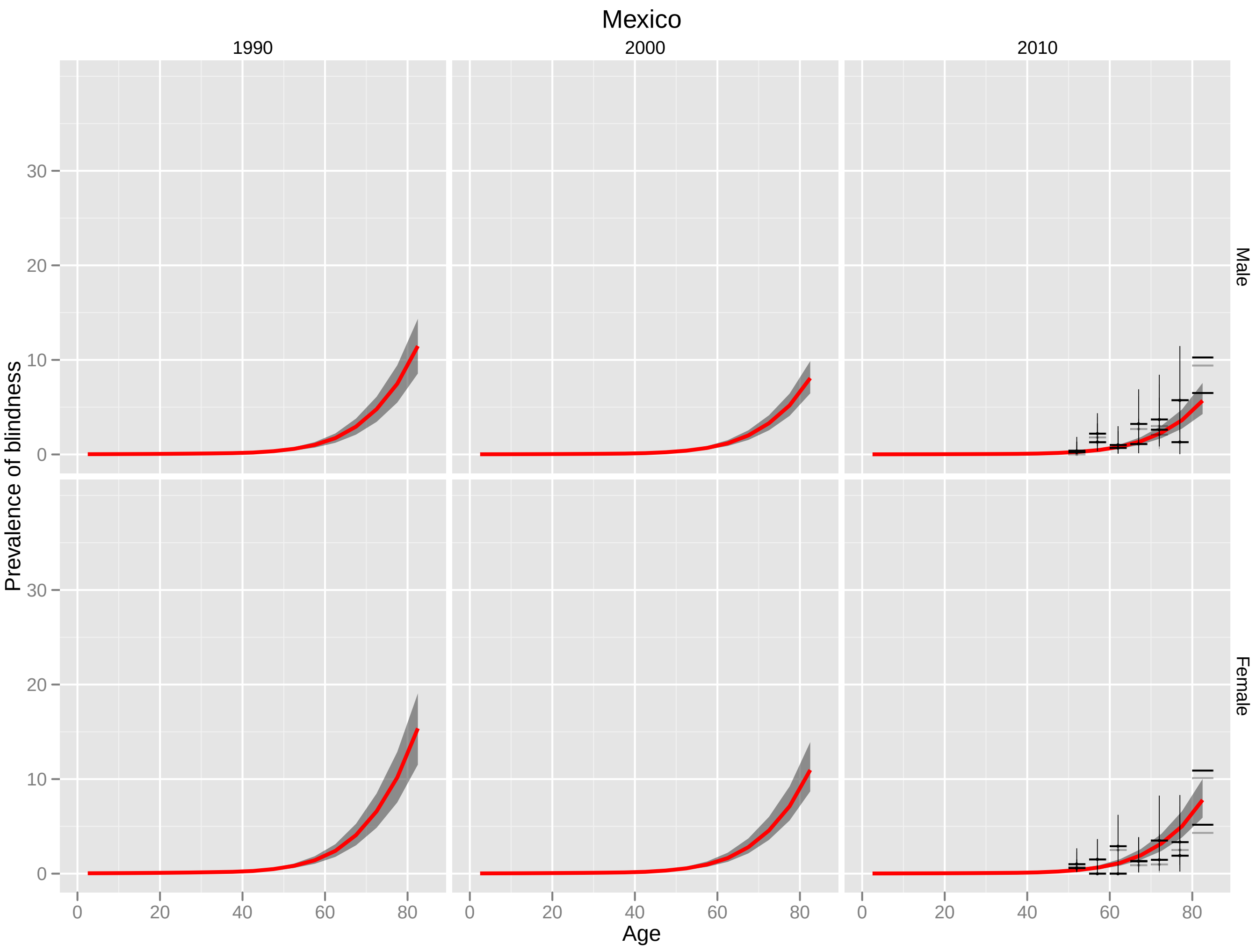
Mexico
1990
2000
2010
Male
Female
Prevalence of blindness
Age
0
10
20
30
0
20
40
60
80

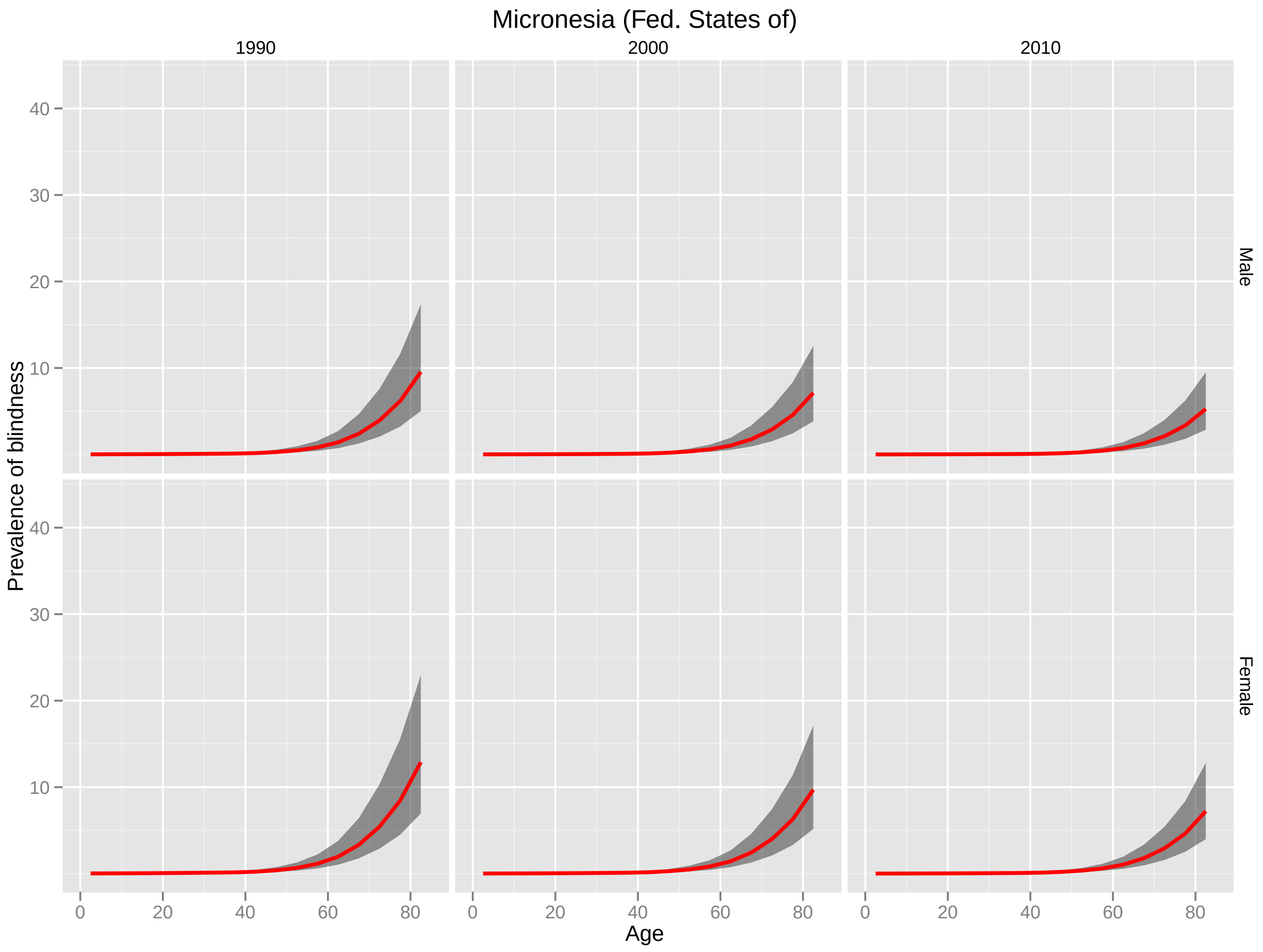
Micronesia (Fed. States of)
1990
2000
2010
Male
Female
Prevalence of blindness
Age
0
20
40
60
80
10
20
30
40

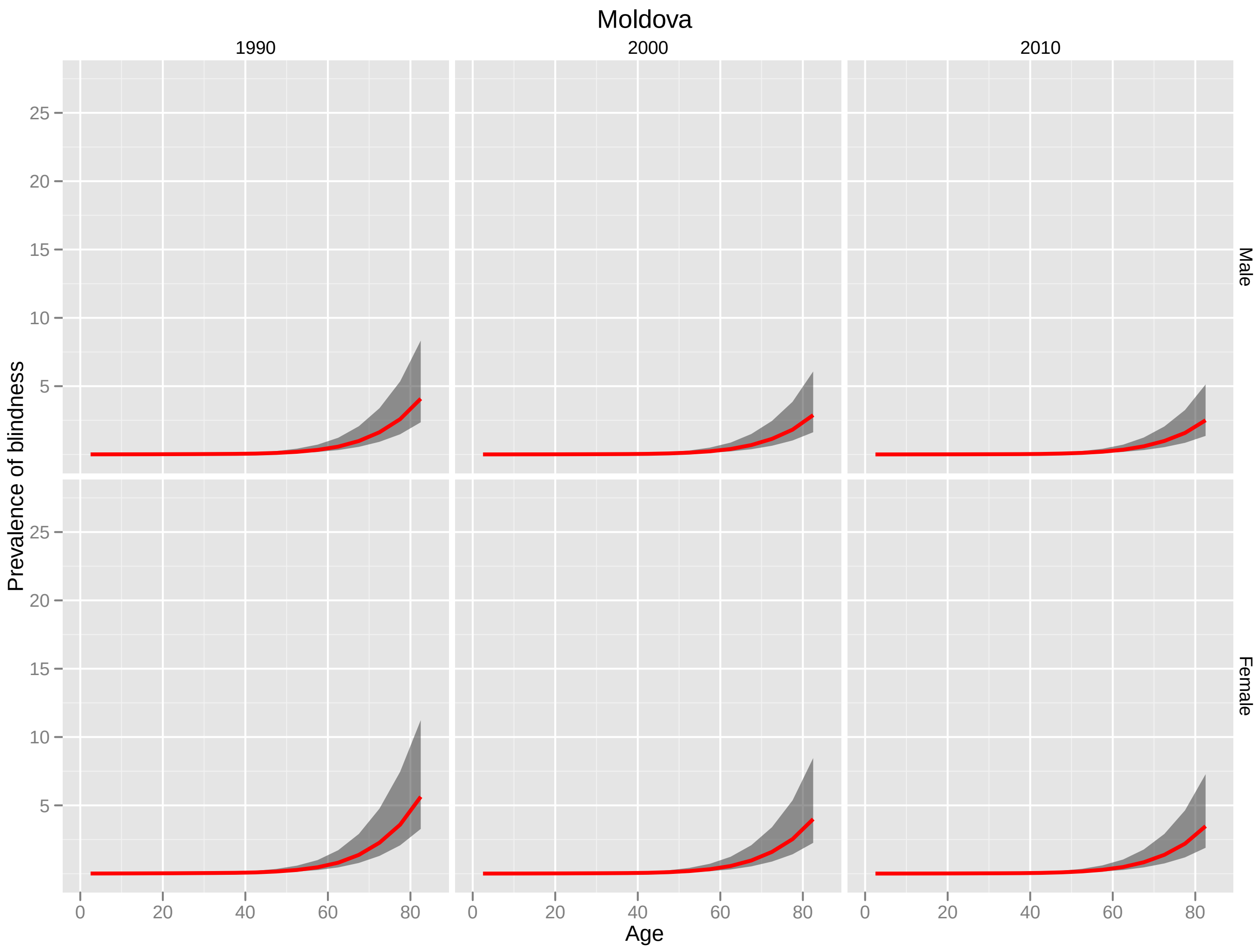
Moldova
1990
2000
2010
Male
Female
Prevalence of blindness
Age
25
20
15
10
5
0
20
40
60
80

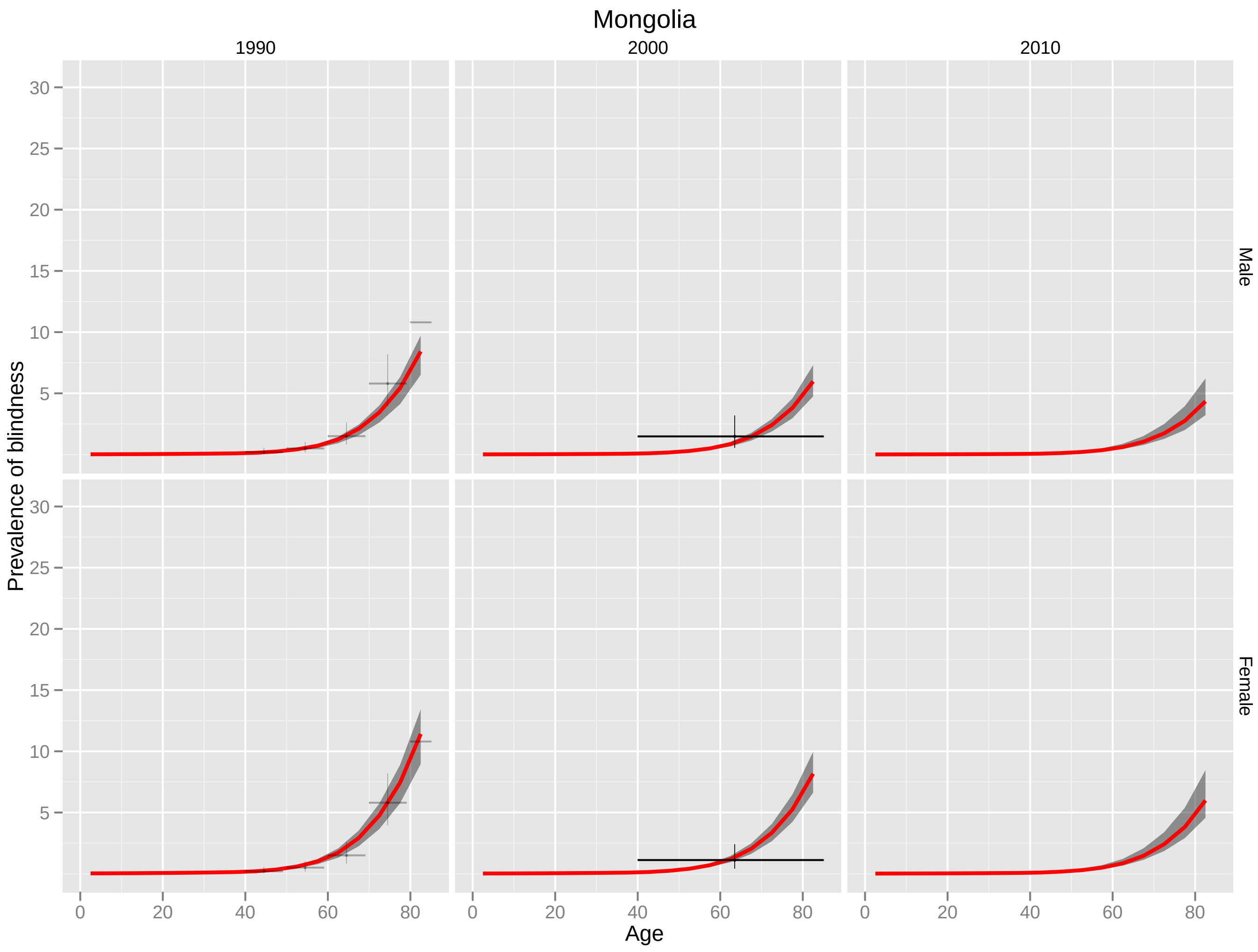
Mongolia
1990
2000
2010
Male
Female
Prevalence of blindness
Age
30
25
20
15
10
5
0
20
40
60
80

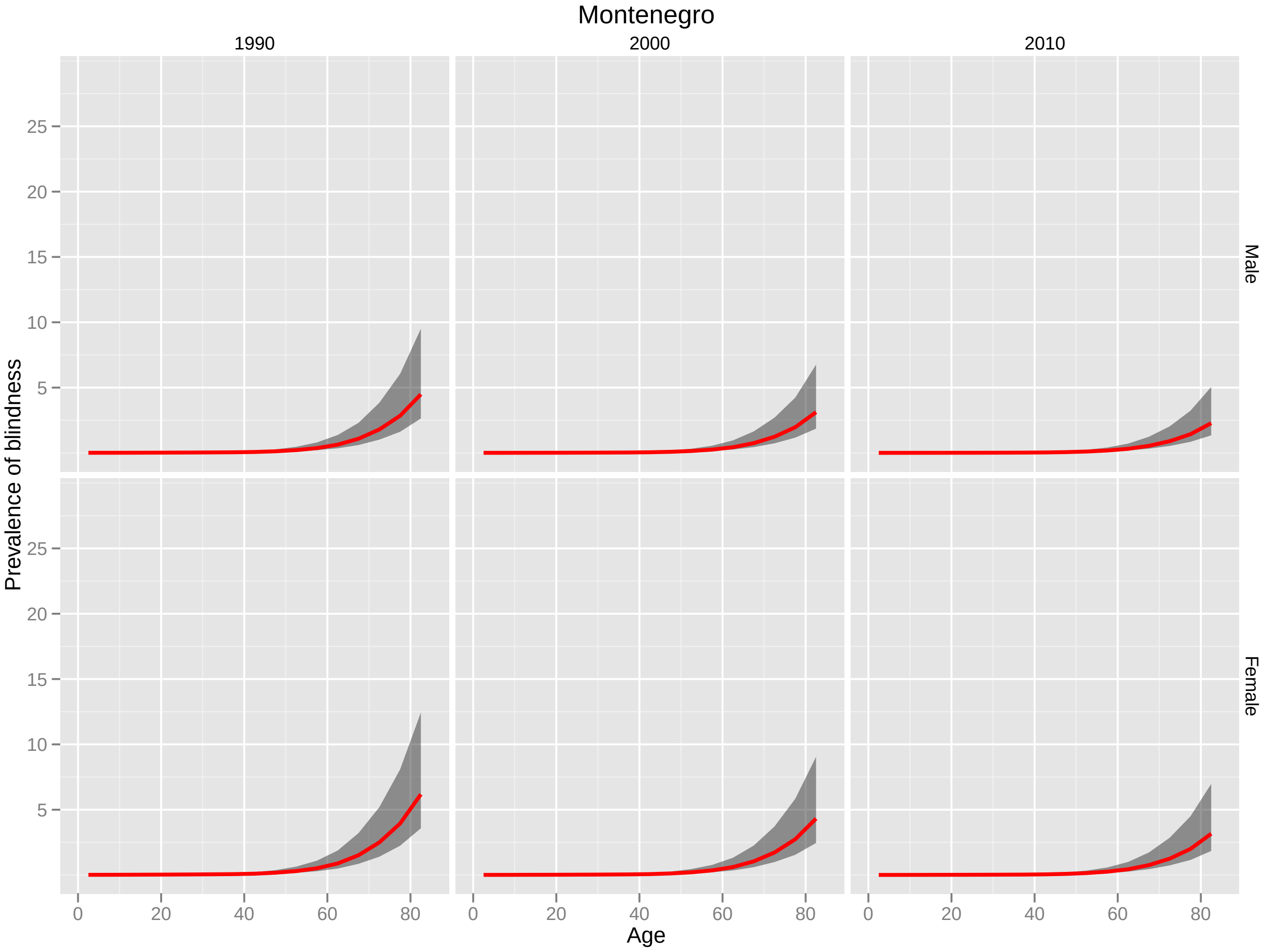
Montenegro
1990
2000
2010
Male
Female
Prevalence of blindness
Age
0
20
40
60
80
5
10
15
20
25

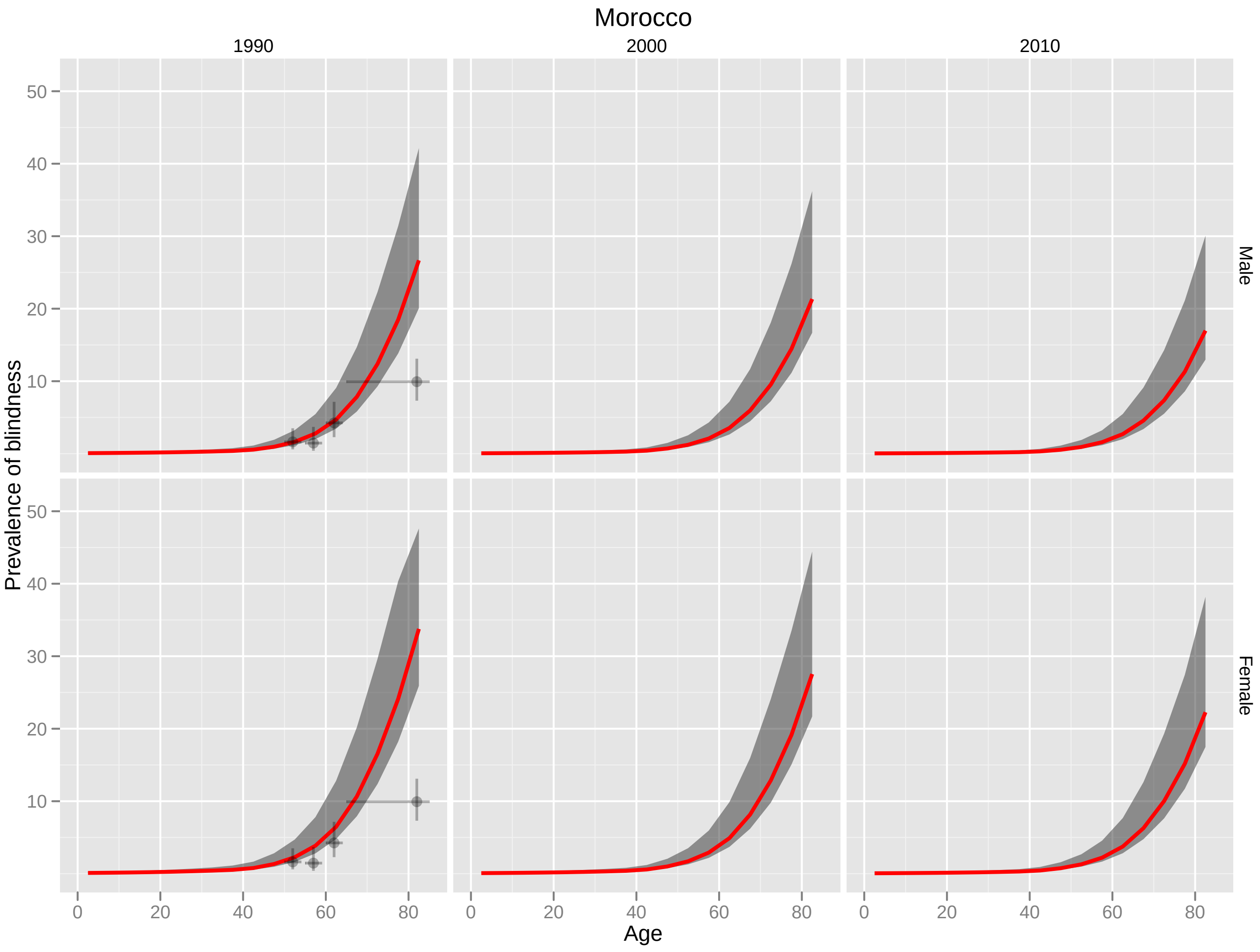
Morocco
1990
2000
2010
Male
Female
Prevalence of blindness
10
20
30
40
50
Age
0
20
40
60
80

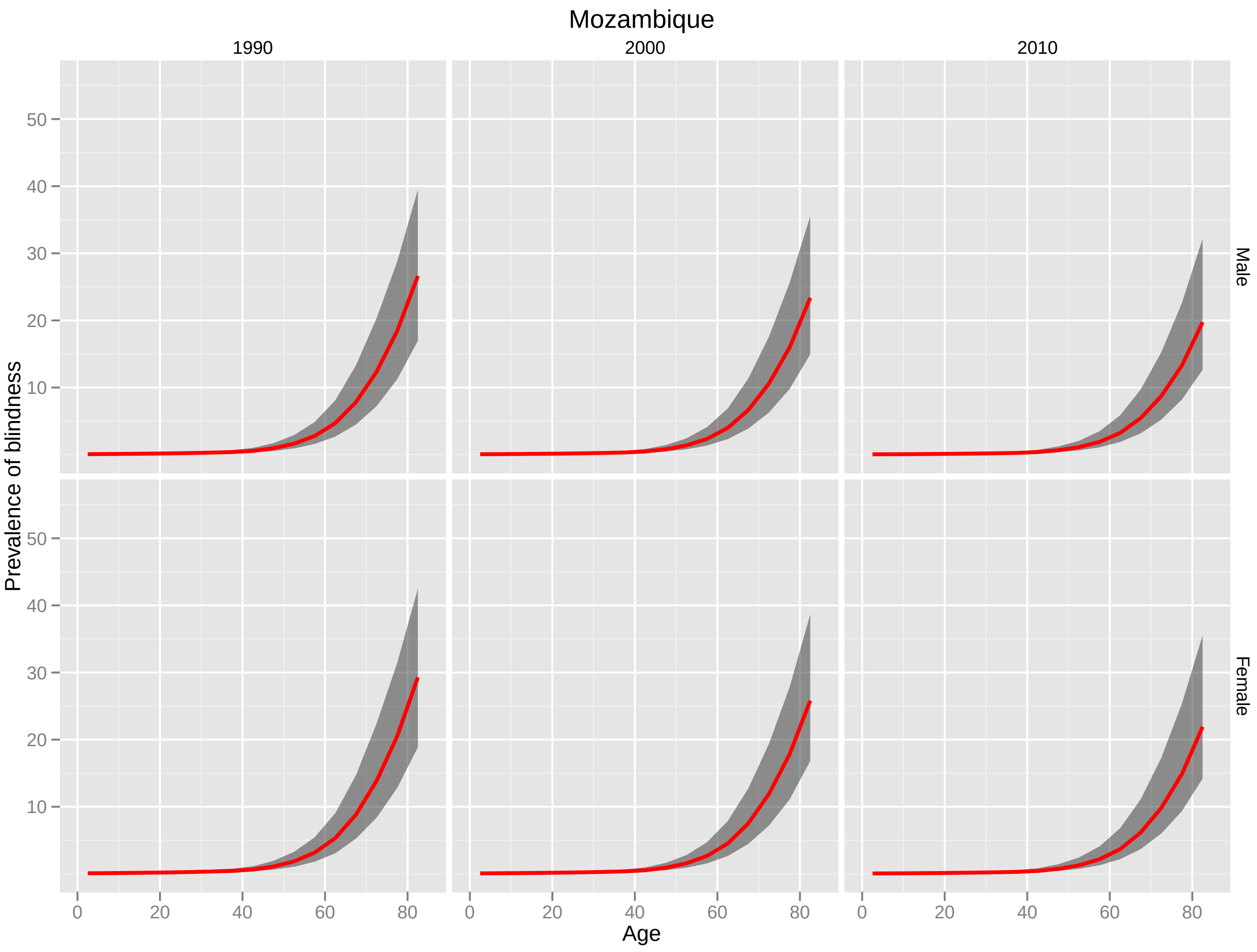
Mozambique
1990
2000
2010
Male
Female
Prevalence of blindness
Age
10
20
30
40
50
0
20
40
60
80

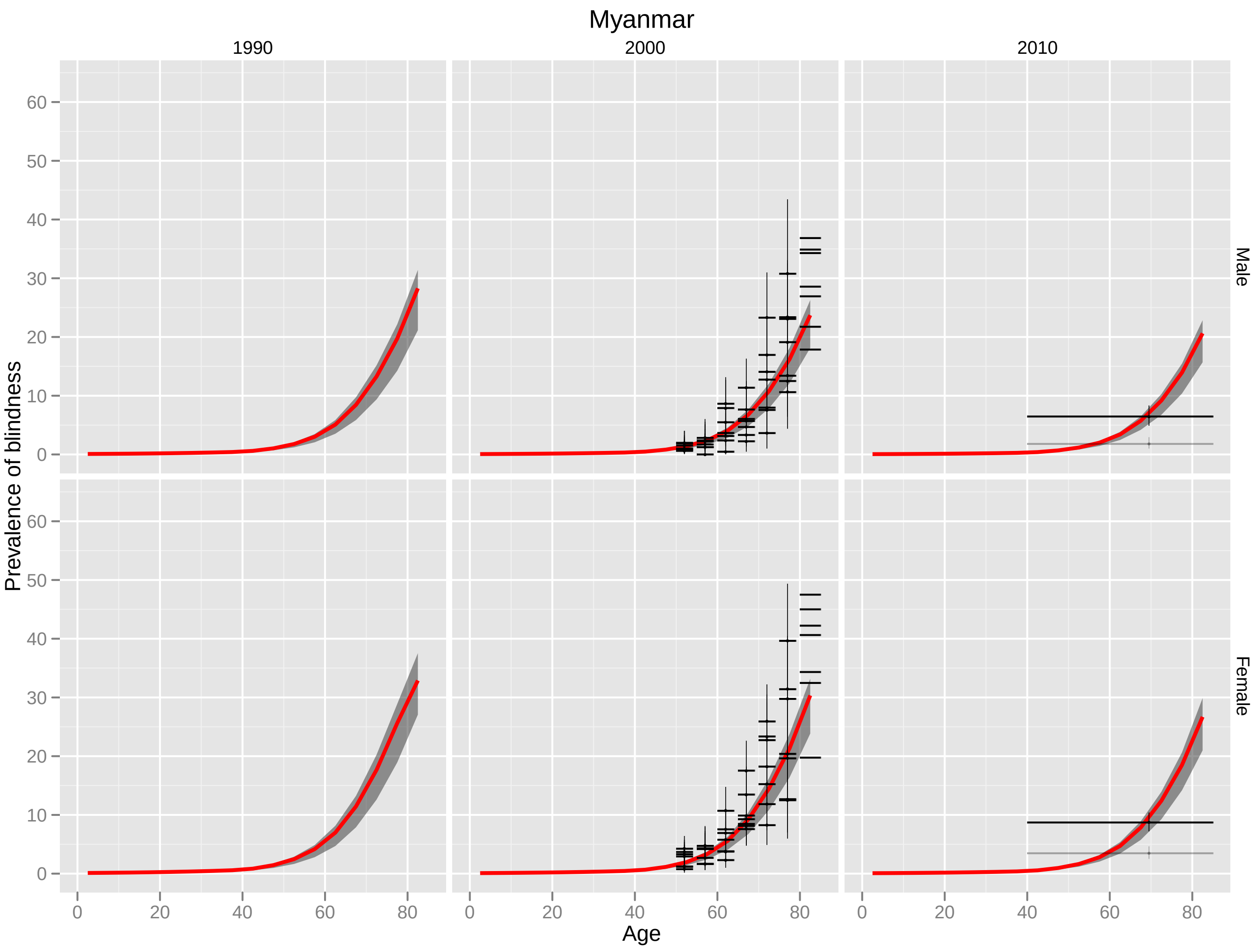
Myanmar
1990
2000
2010
Male
Female
Prevalence of blindness
Age
0
10
20
30
40
50
60
0
20
40
60
80

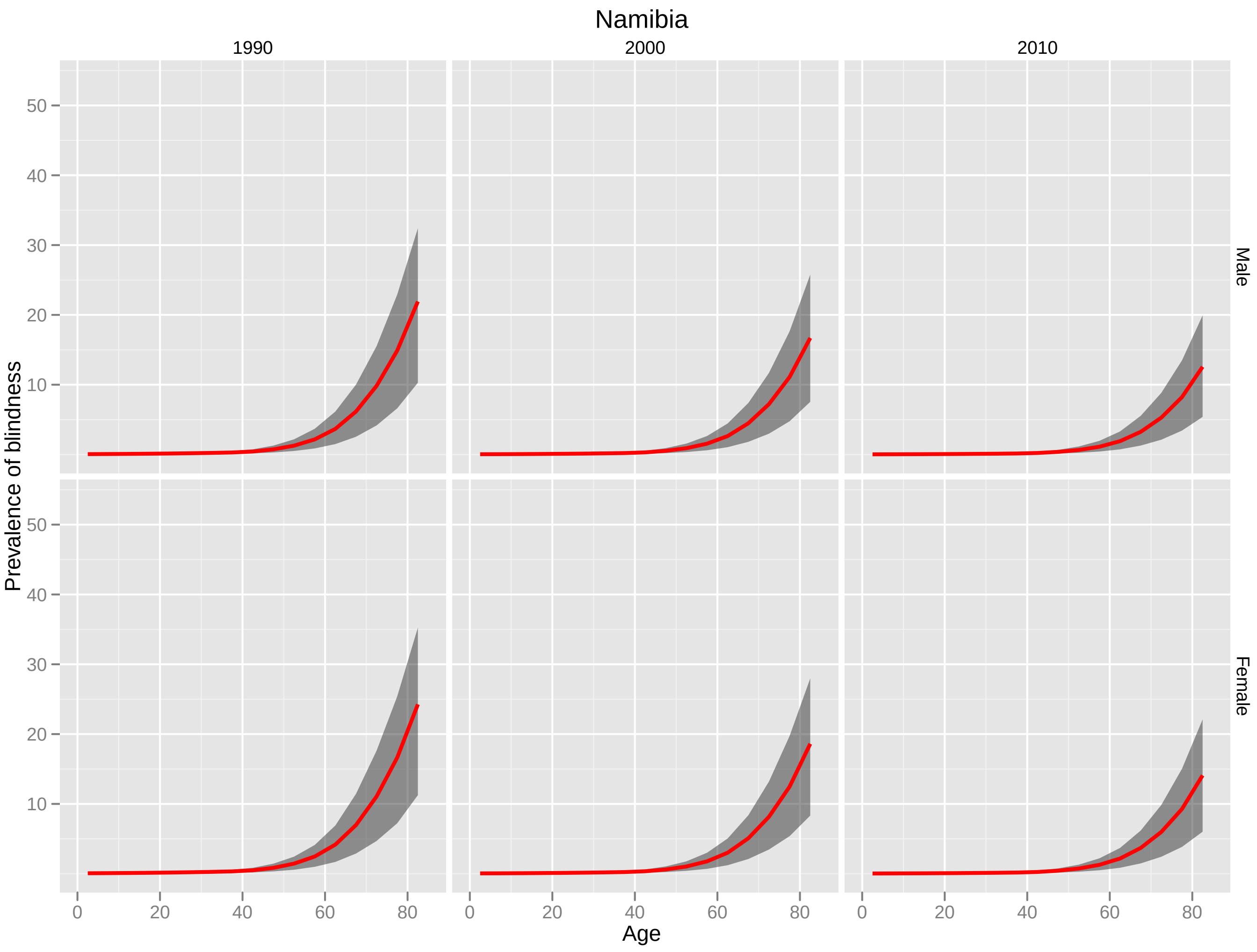
Namibia
1990
2000
2010
Male
Female
Prevalence of blindness
Age
10
20
30
40
50
0
20
40
60
80

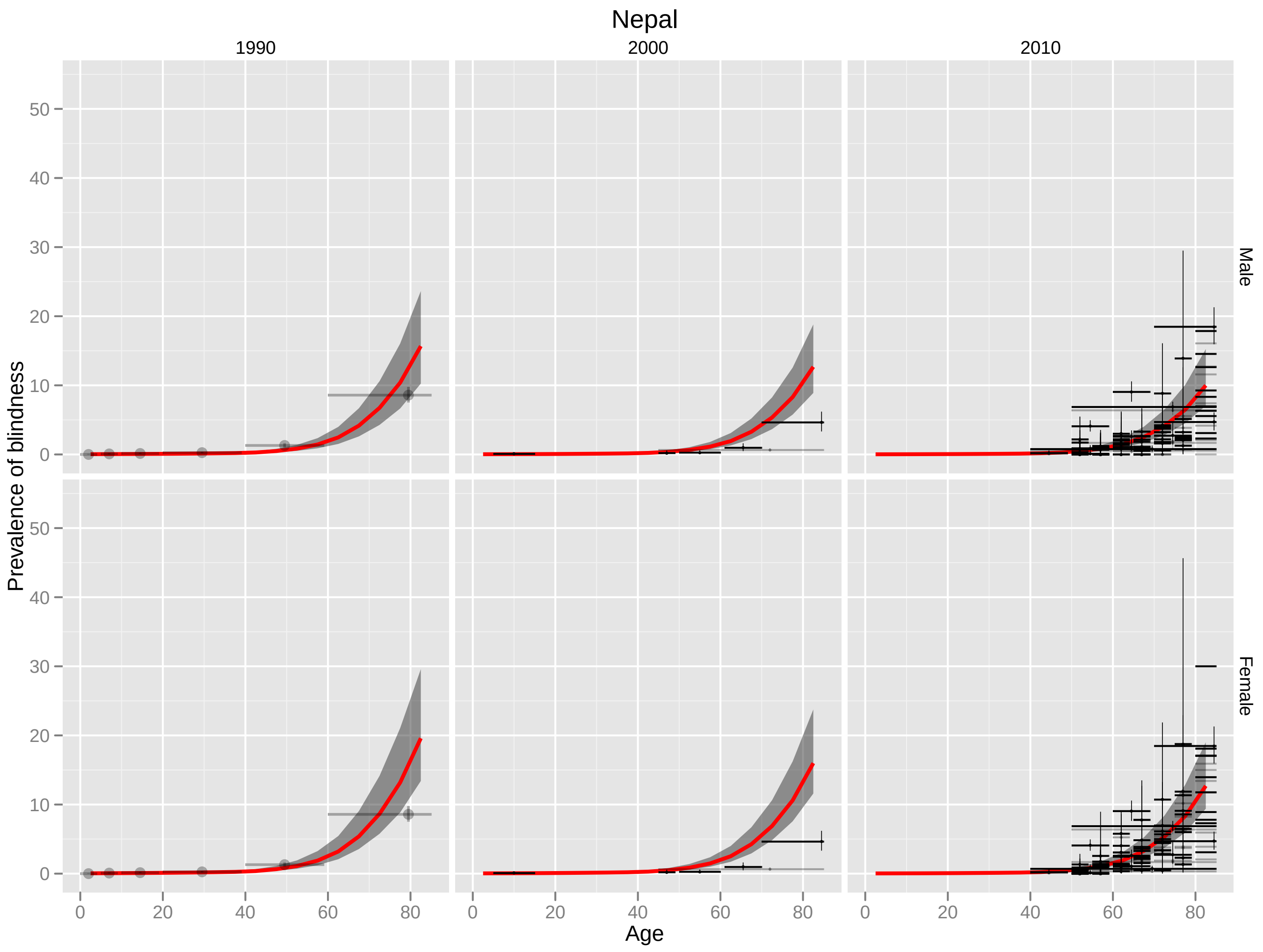
Nepal
1990
2000
2010
Male
Female
Prevalence of blindness
Age
0
10
20
30
40
50
0
20
40
60
80

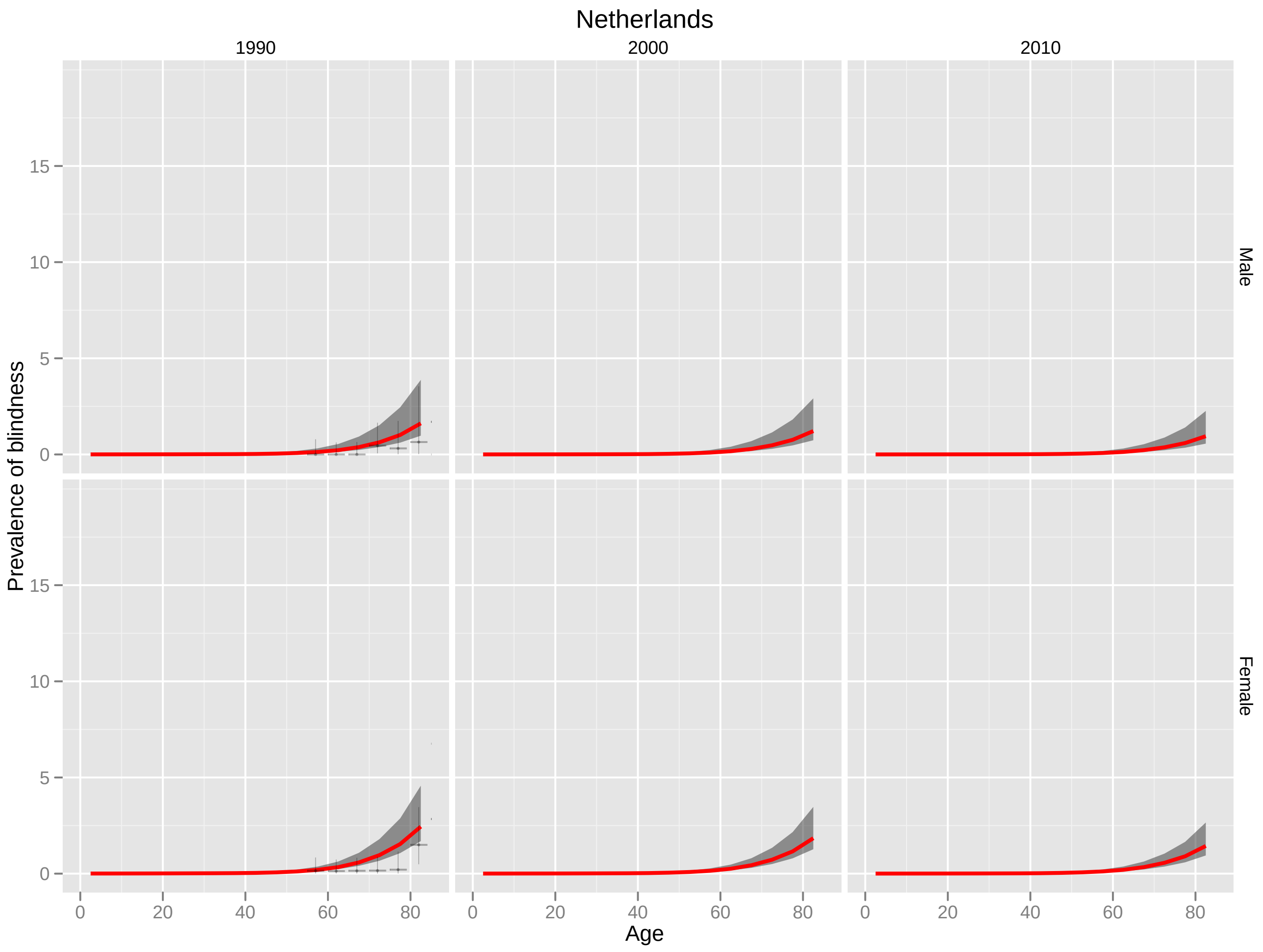
Netherlands
1990
2000
2010
Male
Female
Prevalence of blindness
0
5
10
15
0
20
40
60
80
Age

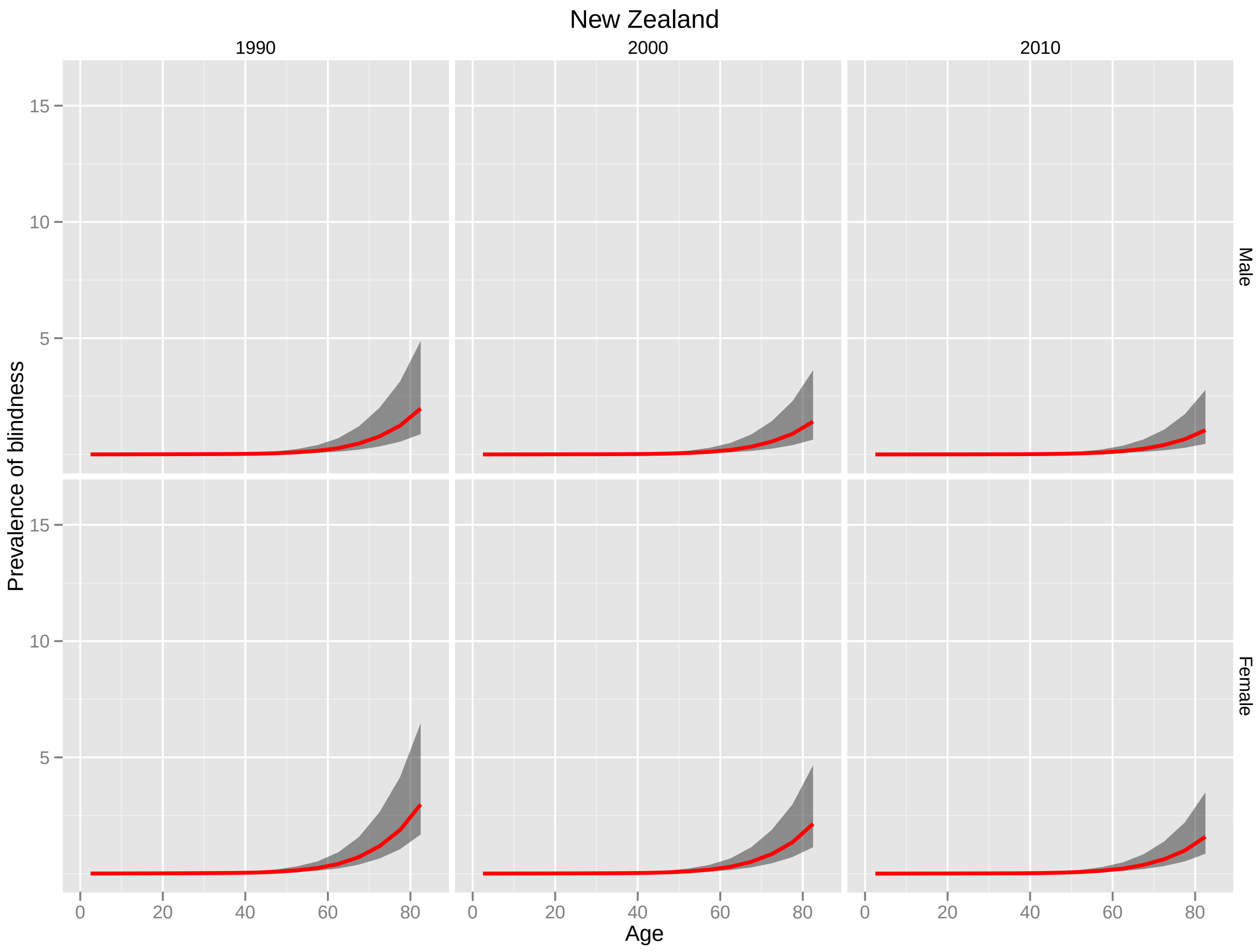
New Zealand
1990
2000
2010
Male
Female
Prevalence of blindness
Age
15
10
5
0
20
40
60
80

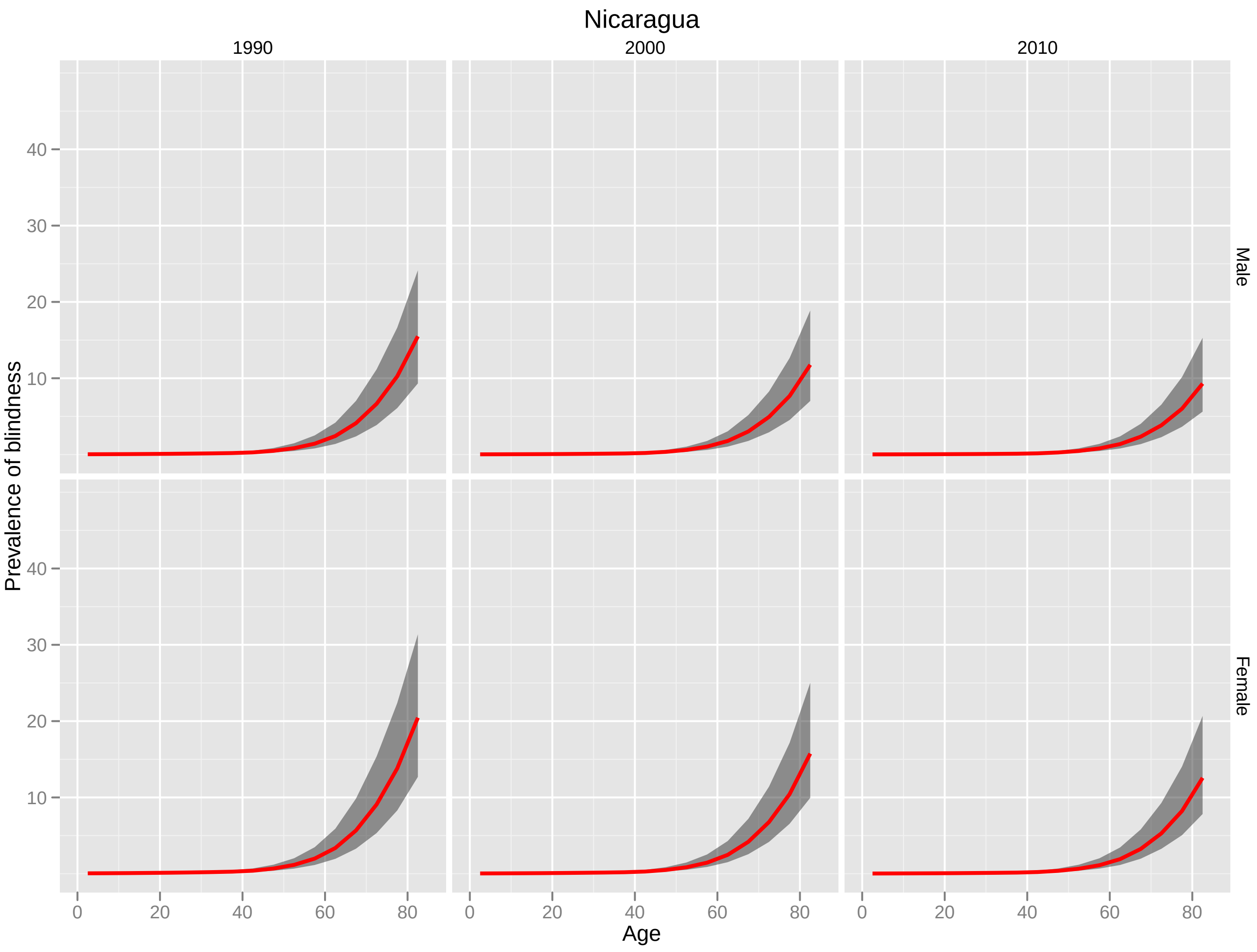
Nicaragua
1990
2000
2010
Male
Female
Prevalence of blindness
Age
10
20
30
40
0
20
40
60
80

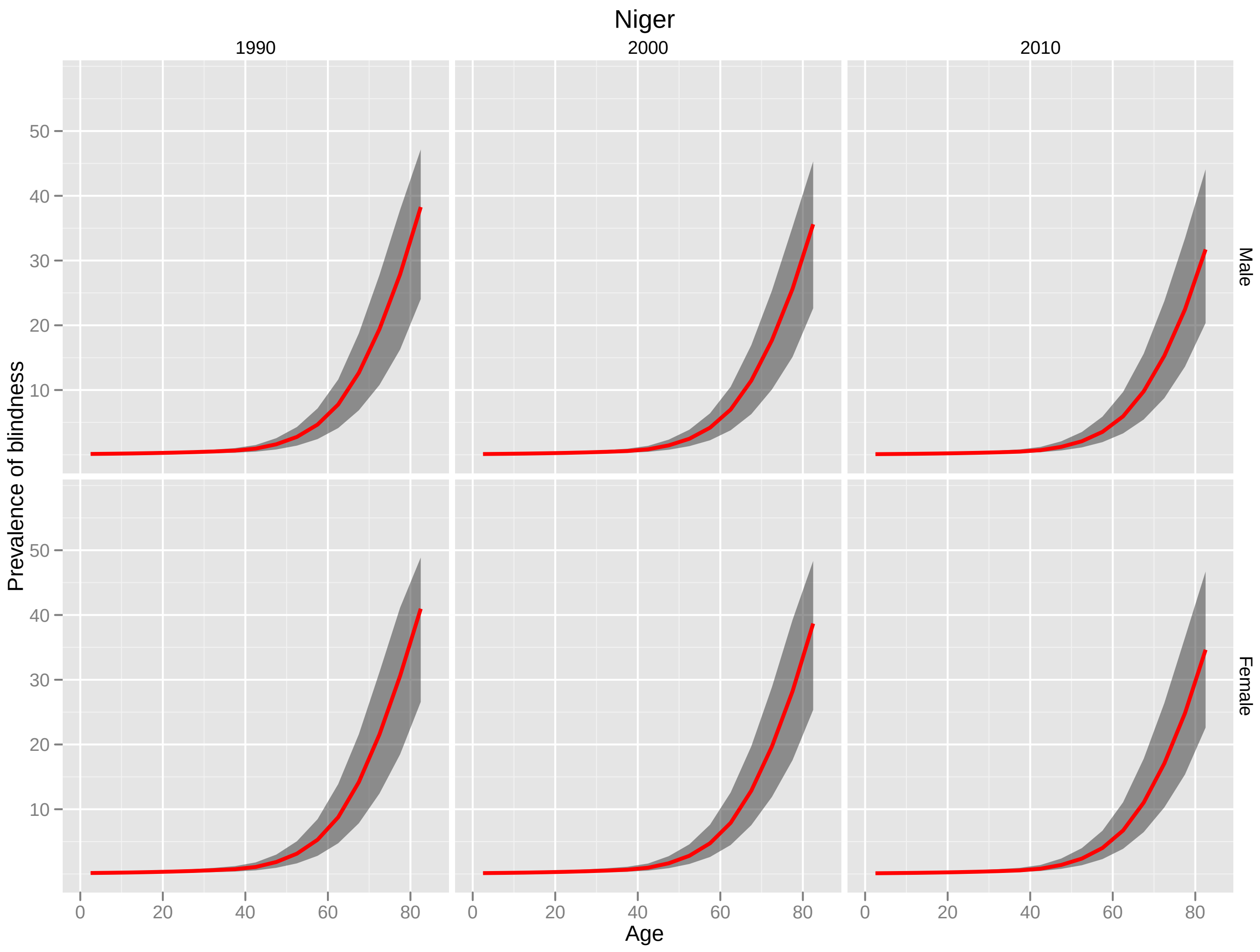
Niger
1990
2000
2010
Male
Female
Prevalence of blindness
Age
0
20
40
60
80
10
20
30
40
50

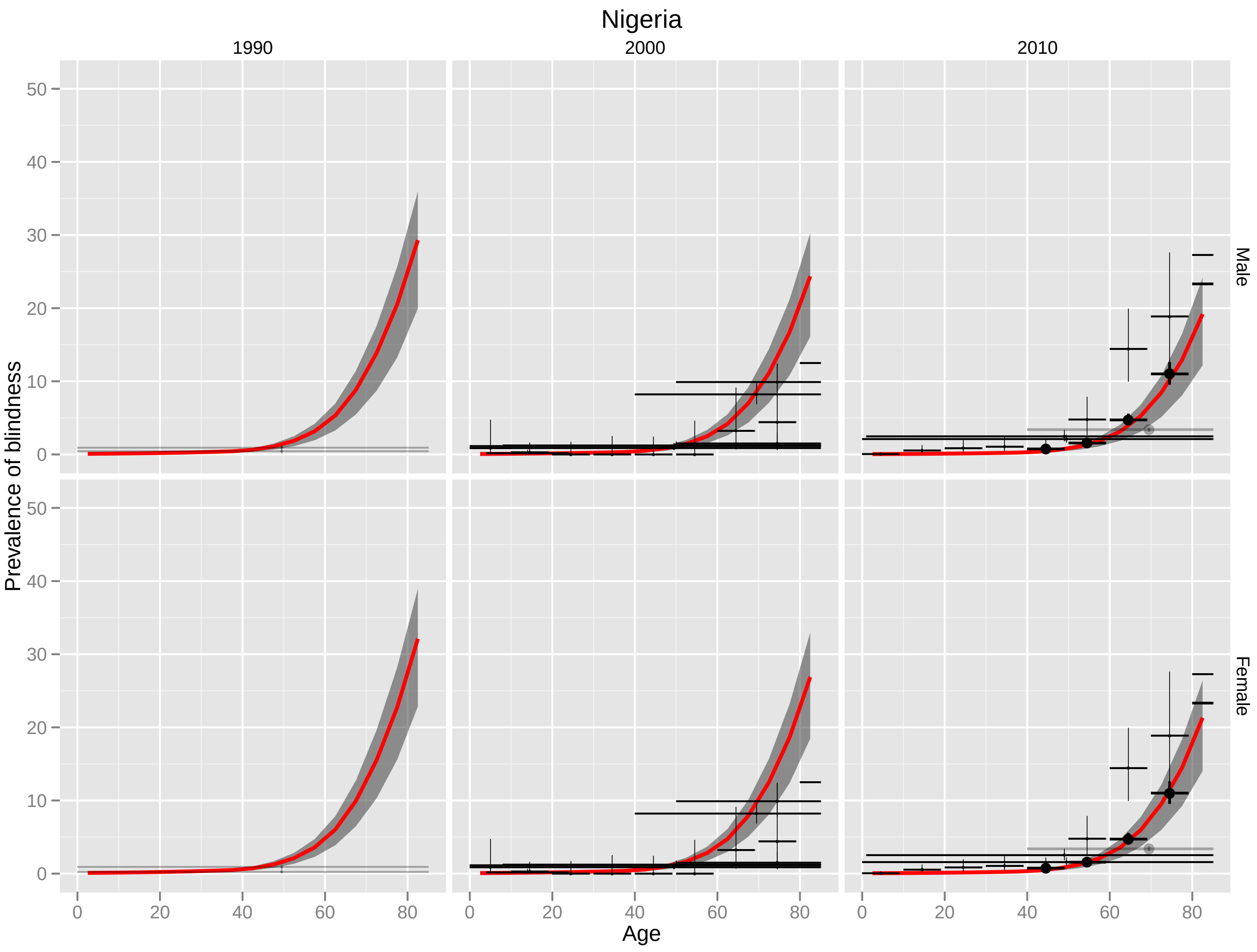
Nigeria
1990
2000
2010
Male
Female
Prevalence of blindness
Age
0
10
20
30
40
50
0
20
40
60
80

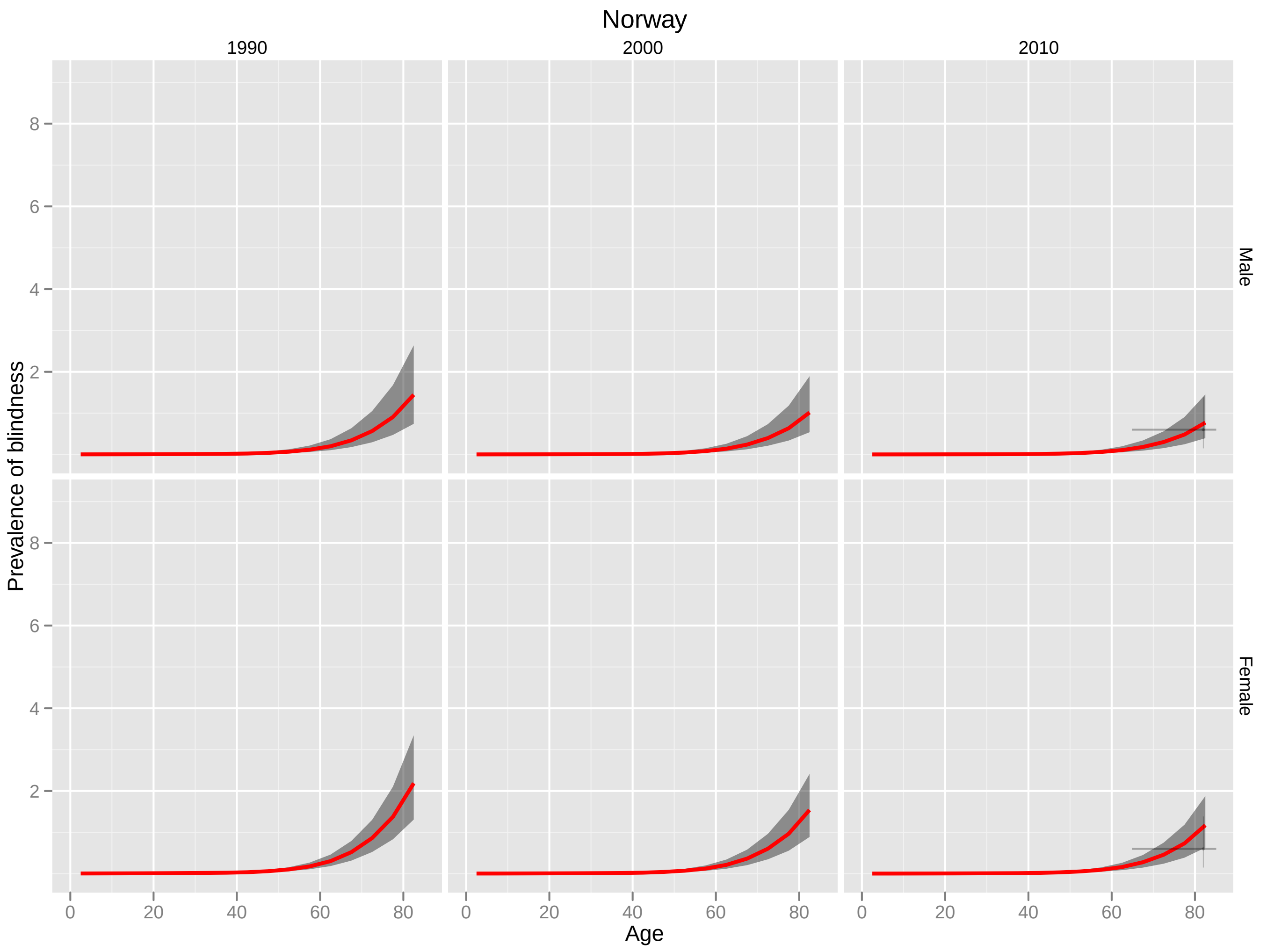
Norway
1990
2000
2010
Male
Female
Prevalence of blindness
Age
0
20
40
60
80
2
4
6
8

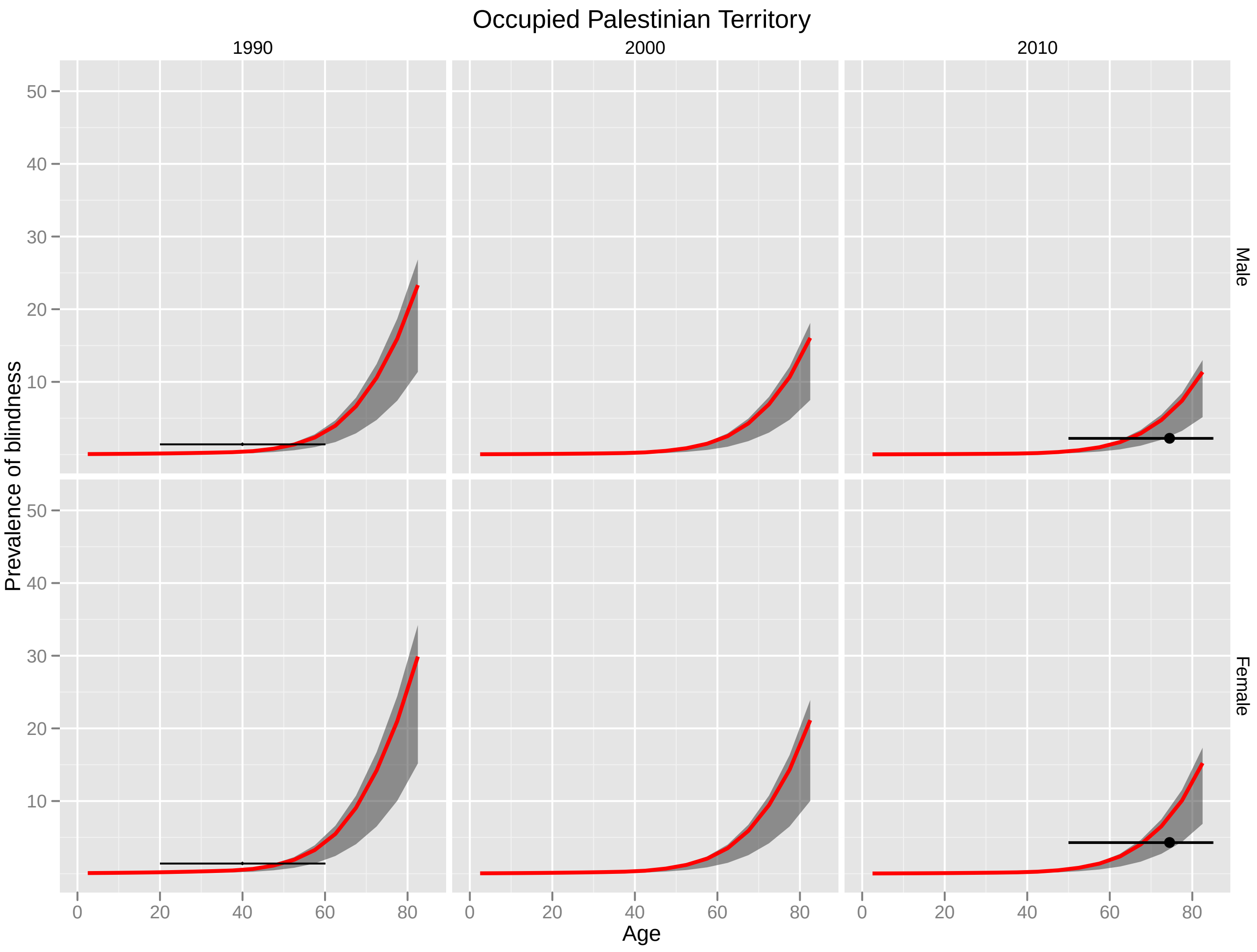
Occupied Palestinian Territory
1990
2000
2010
Male
Female
Prevalence of blindness
Age
0
10
20
30
40
50
60
80

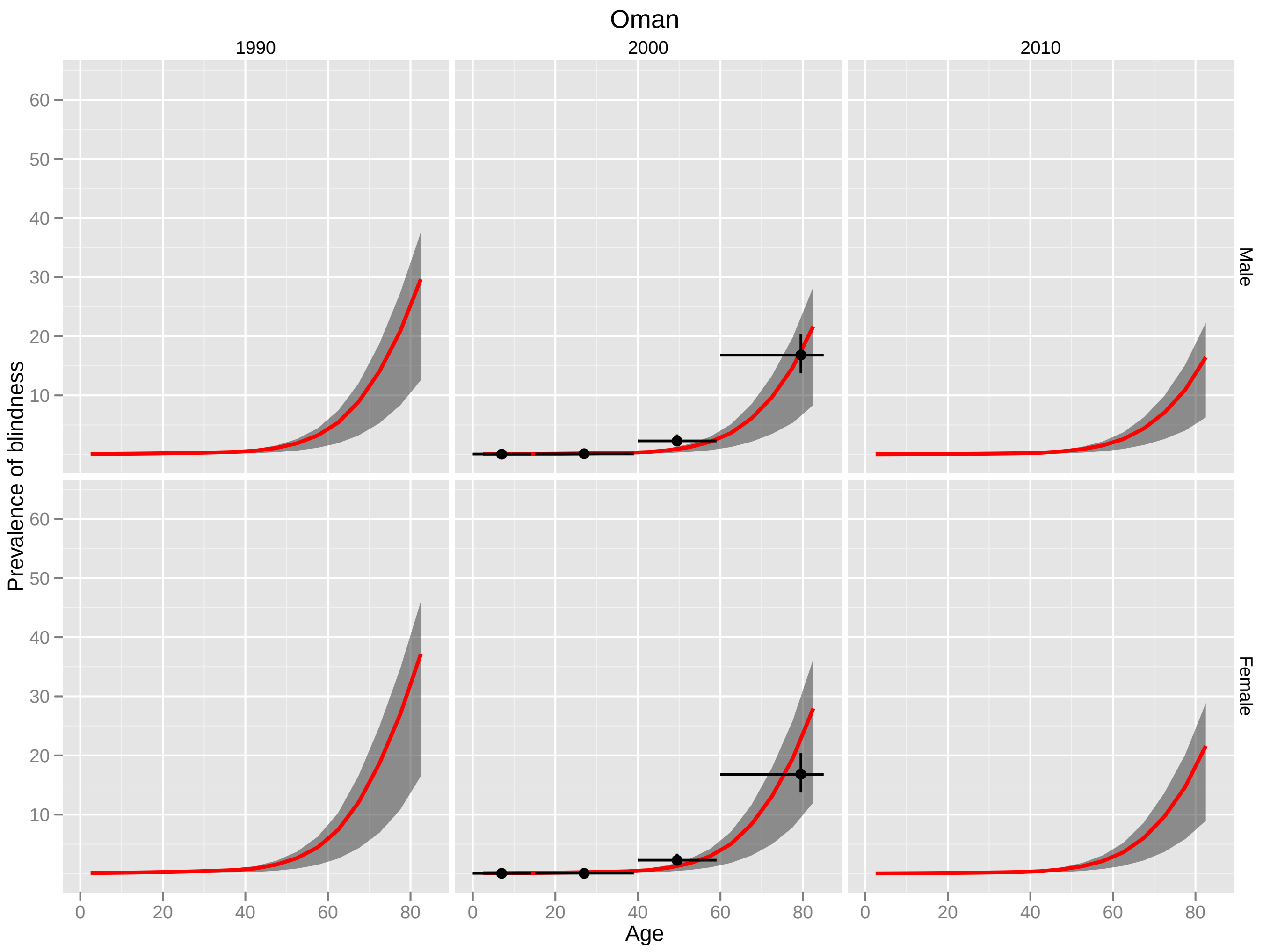
Oman
1990
2000
2010
Male
Female
Prevalence of blindness
Age
0
20
40
60
80
10
20
30
40
50
60

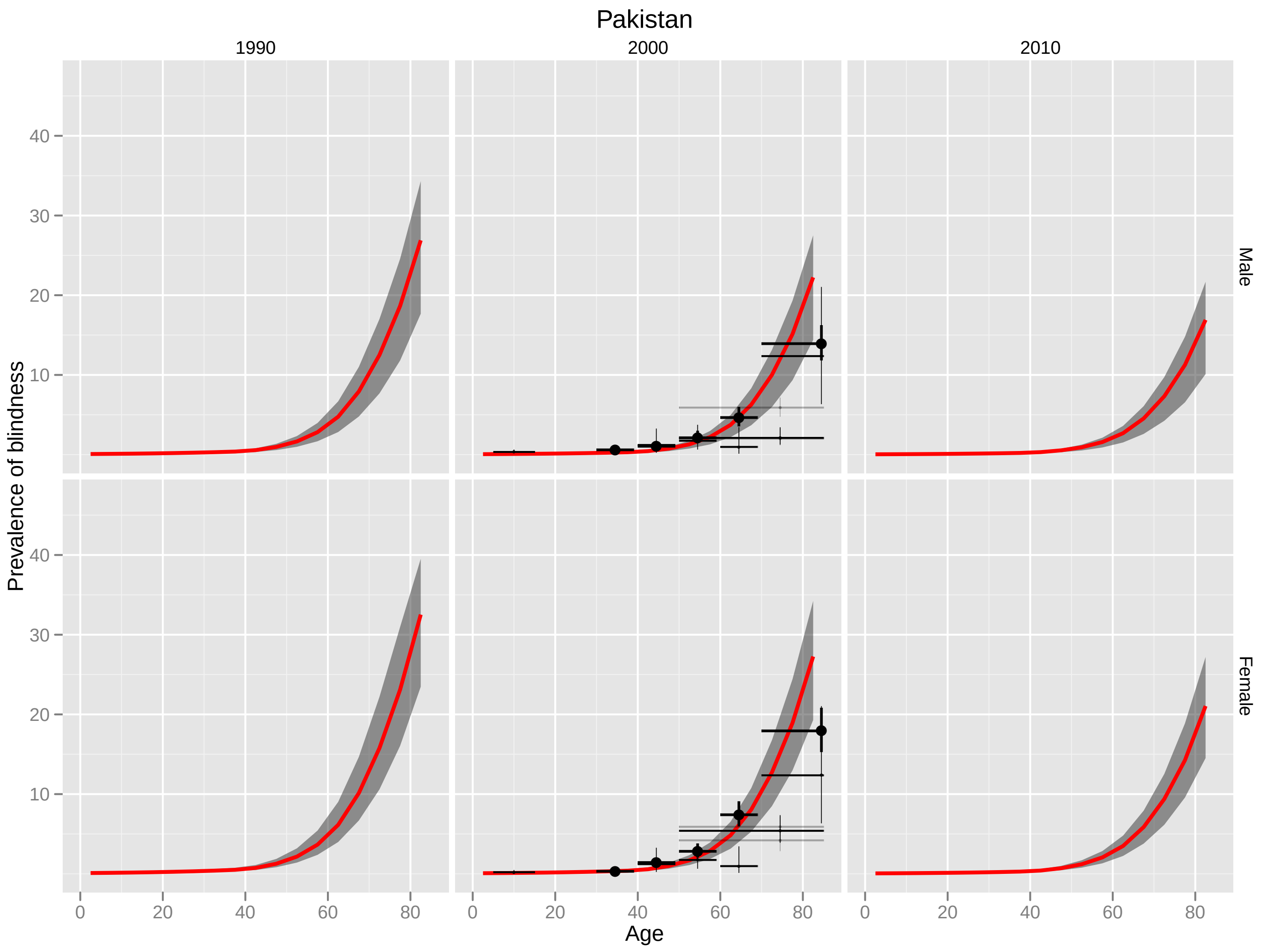
Pakistan
1990
2000
2010
Male
Female
Prevalence of blindness
10
20
30
40
Age
0
20
40
60
80

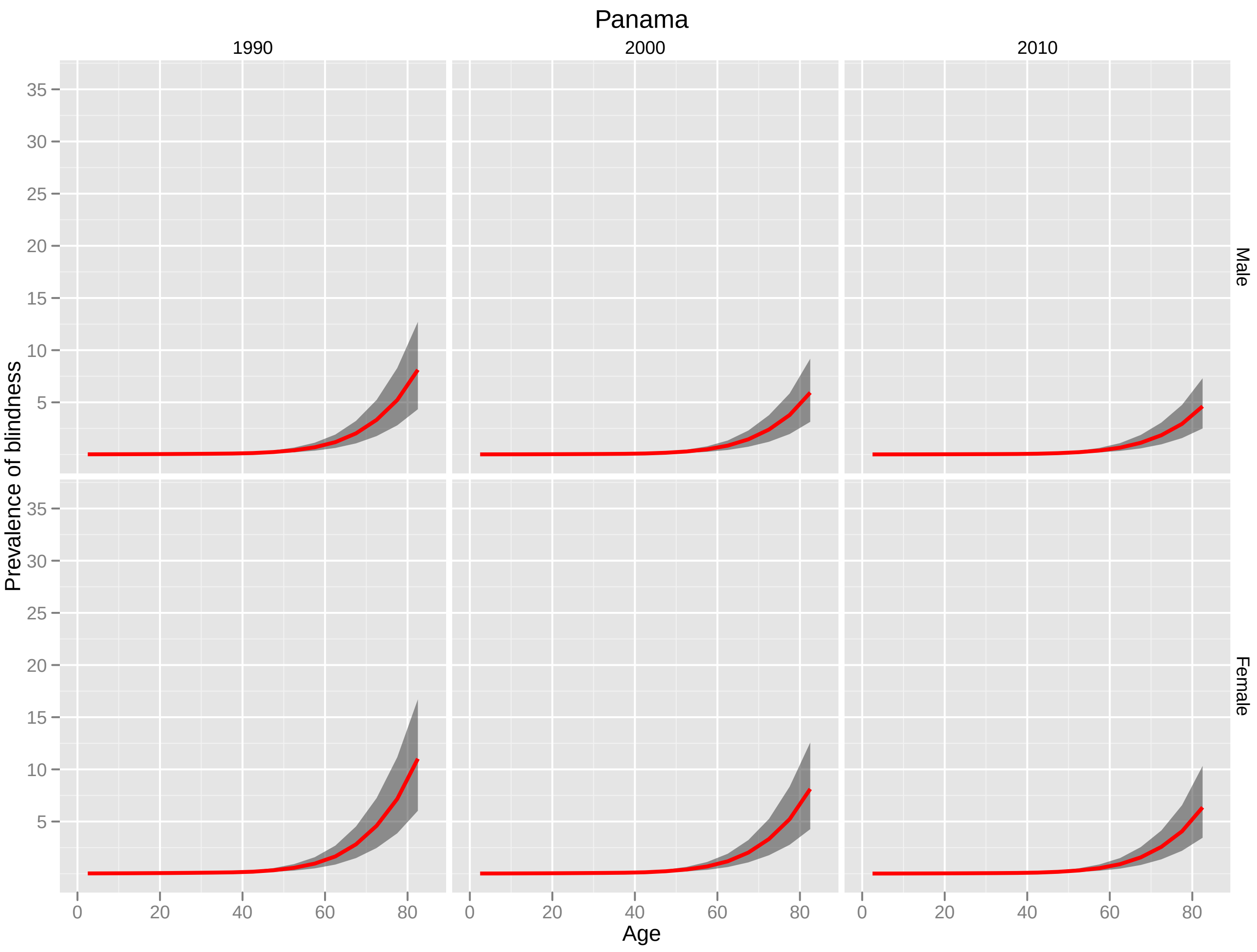
Panama
1990
2000
2010
Male
Female
Prevalence of blindness
Age
35
30
25
20
15
10
5
0
20
40
60
80

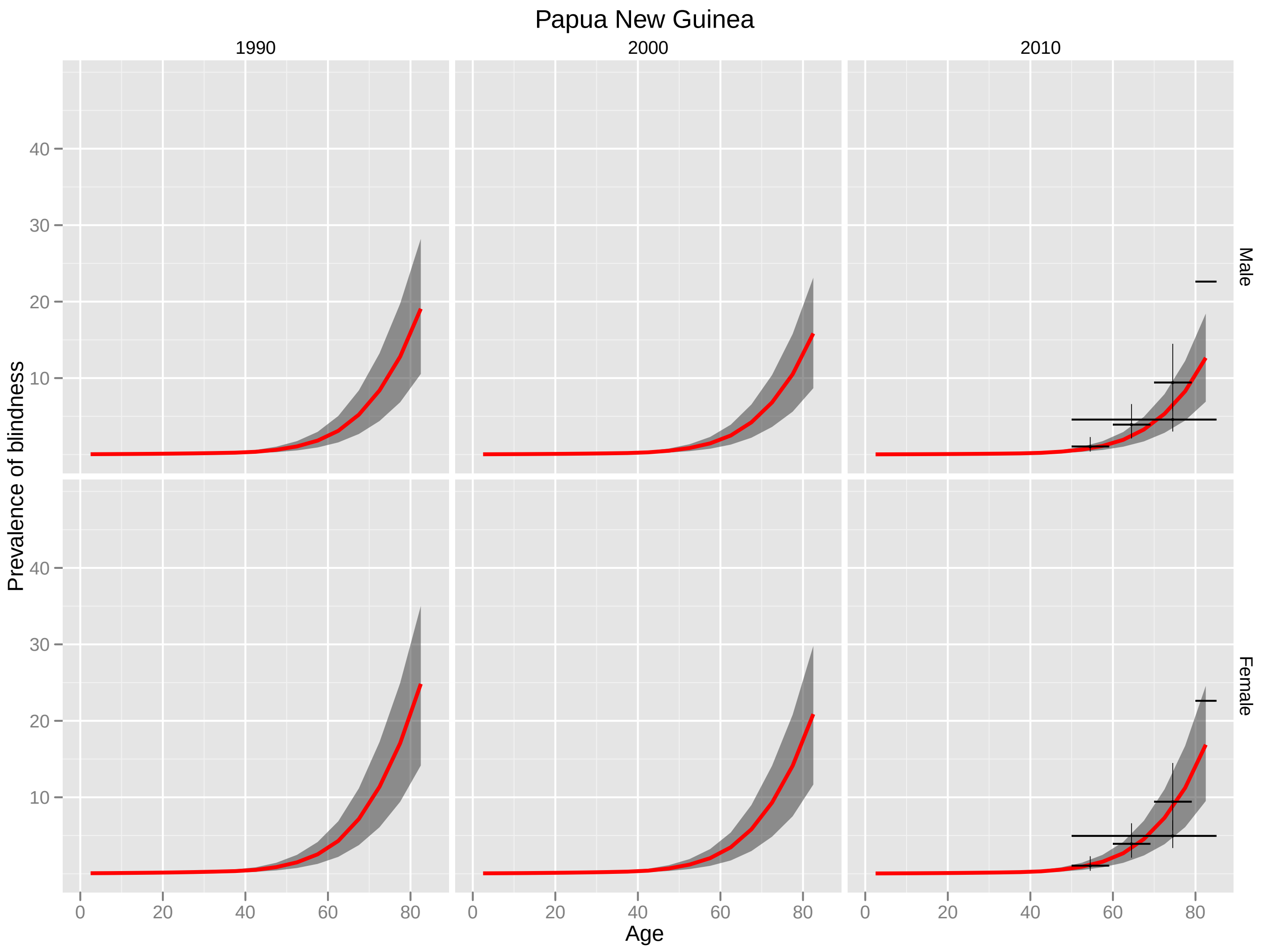
Papua New Guinea
1990
2000
2010
Male
Female
Prevalence of blindness
Age
10
20
30
40
0
20
40
60
80

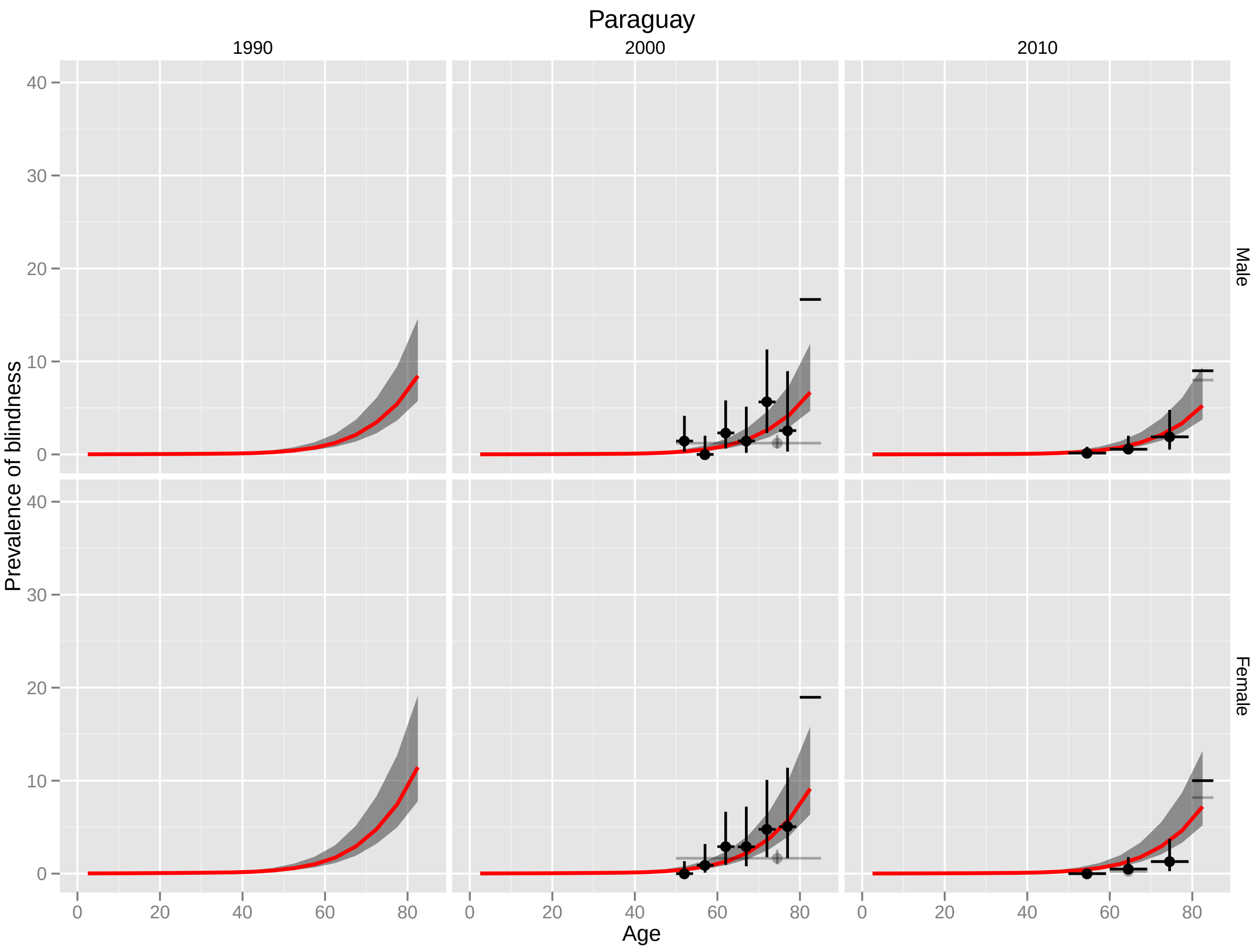

Paraguay
1990
2000
2010
Male
Female
Prevalence of blindness
Age
0
10
20
30
40
0
20
40
60
80

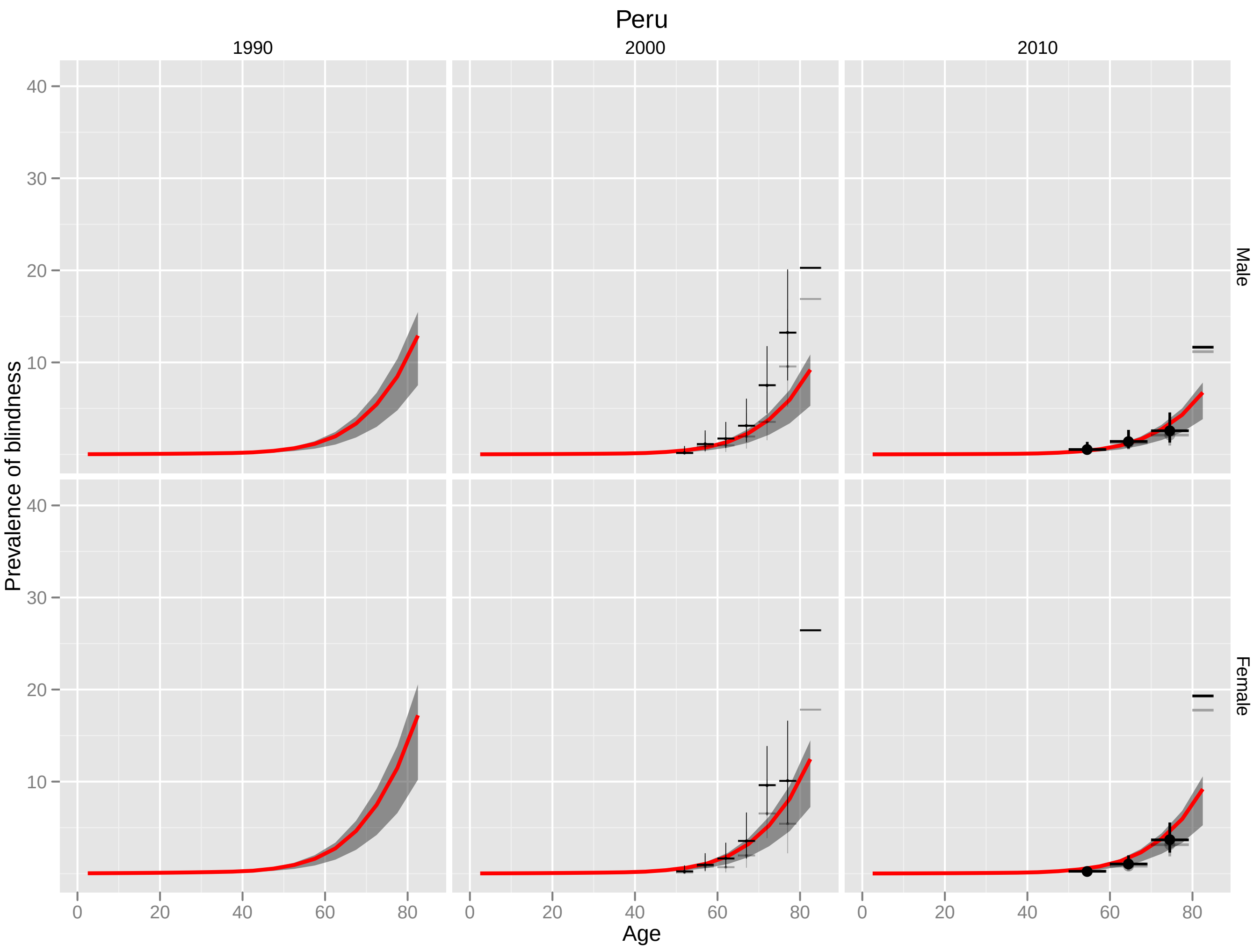
Peru
1990
2000
2010
Male
Female
Prevalence of blindness
Age
0
10
20
30
40
60
80

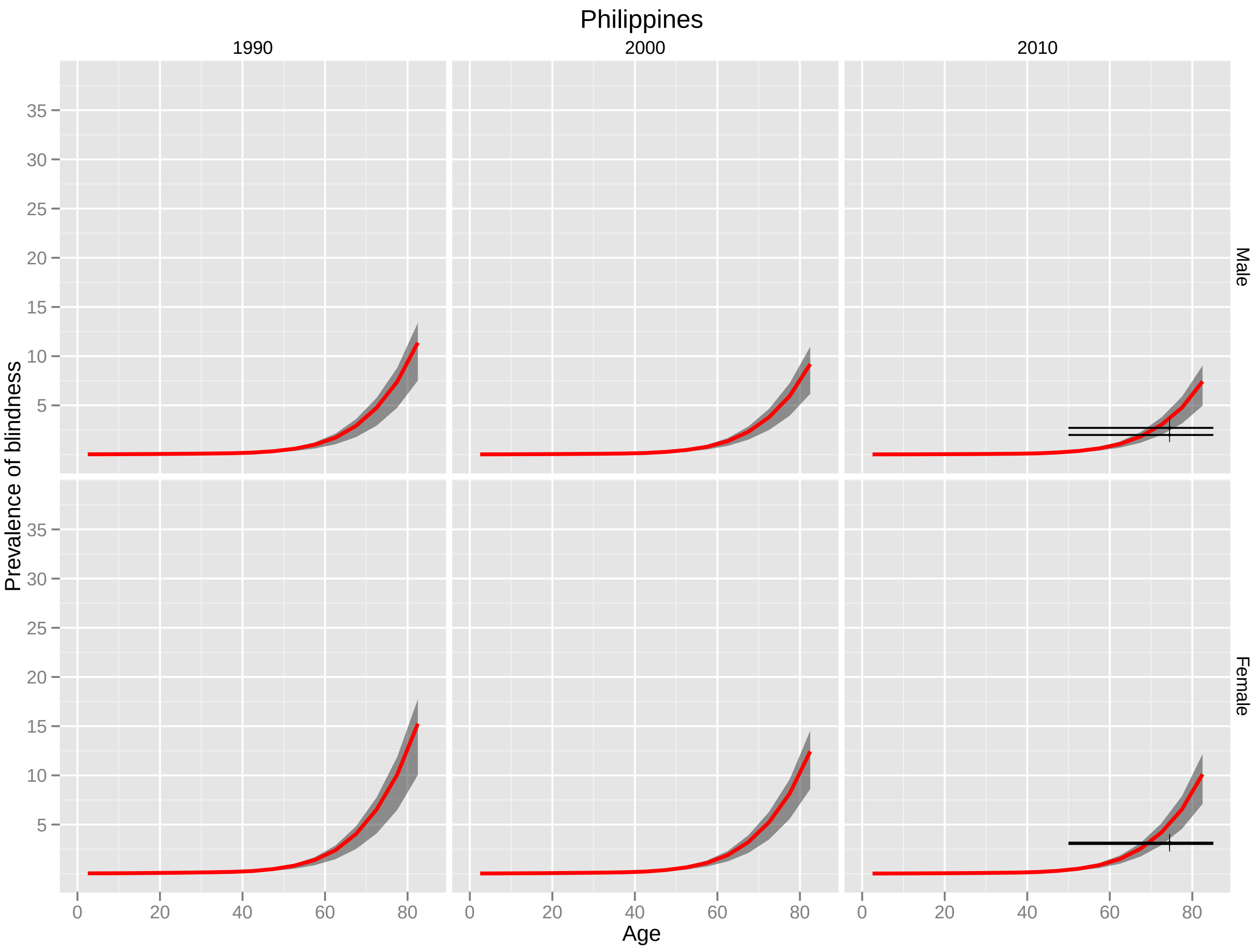
Philippines
1990
2000
2010
Male
Female
Prevalence of blindness
35
30
25
20
15
10
5
0
20
40
60
80
Age

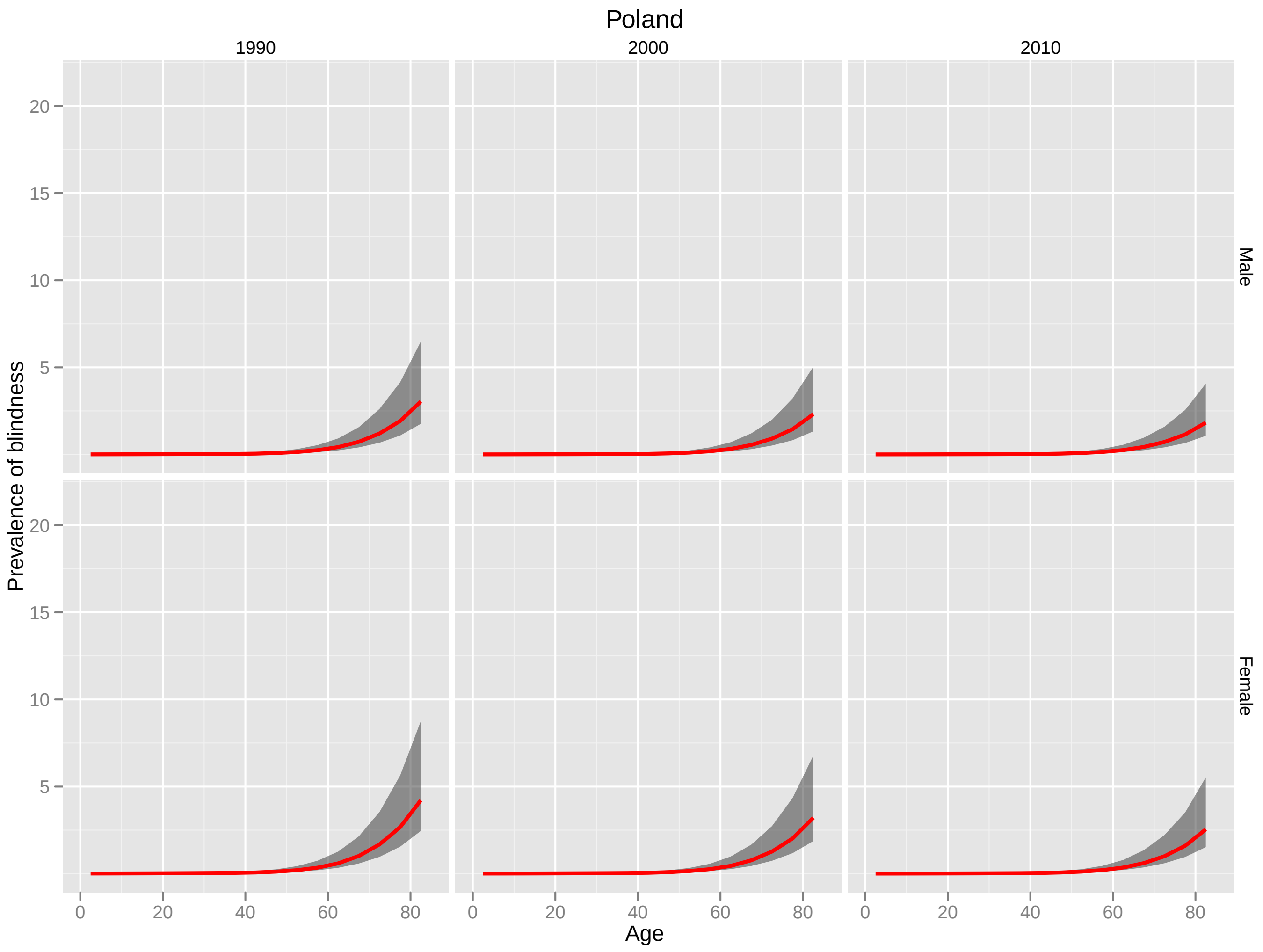
Poland
1990
2000
2010
Male
Female
Prevalence of blindness
20
15
10
5
0
20
40
60
80
Age

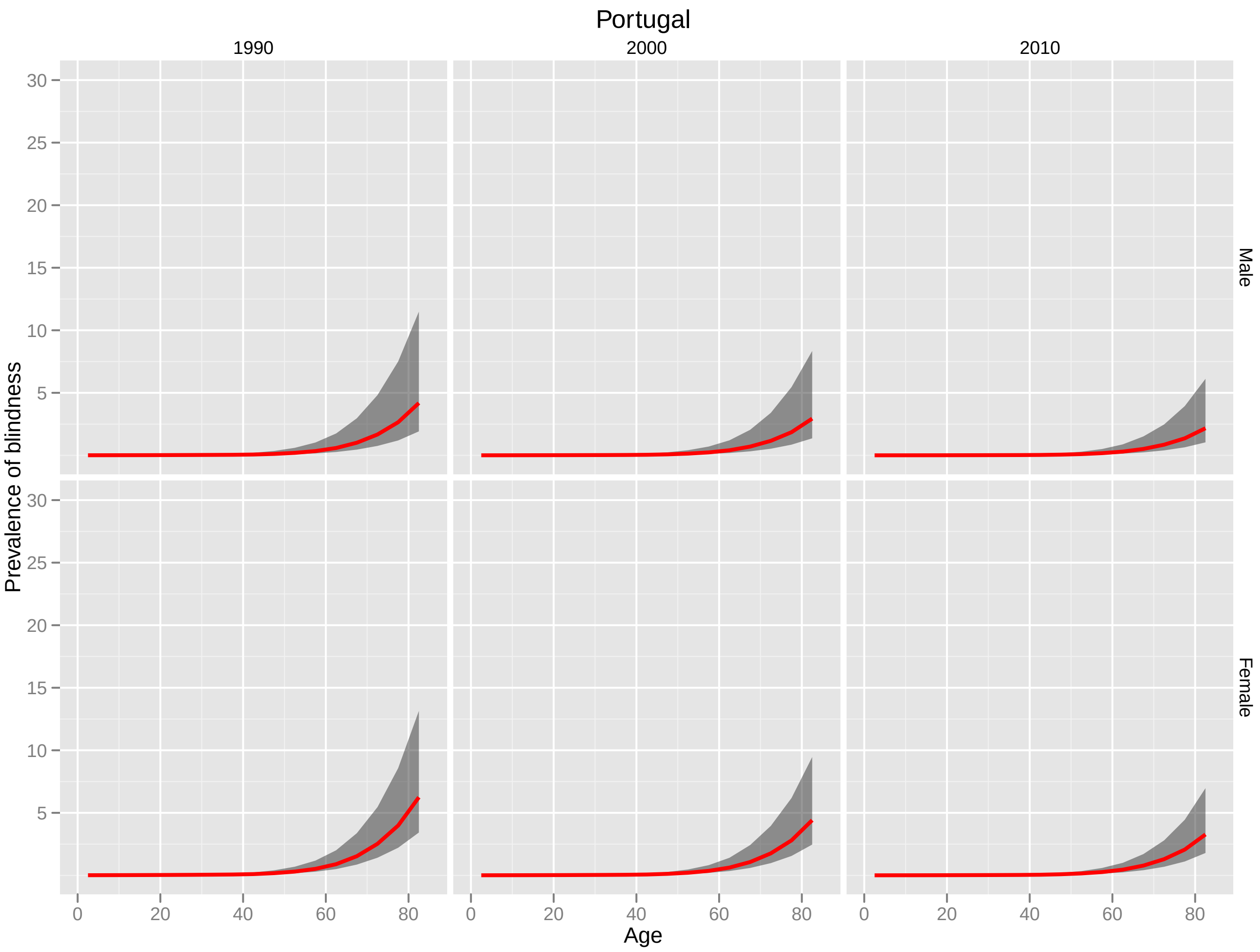

Portugal
1990
2000
2010
Male
Female
Prevalence of blindness
Age
30
25
20
15
10
5
0
20
40
60
80

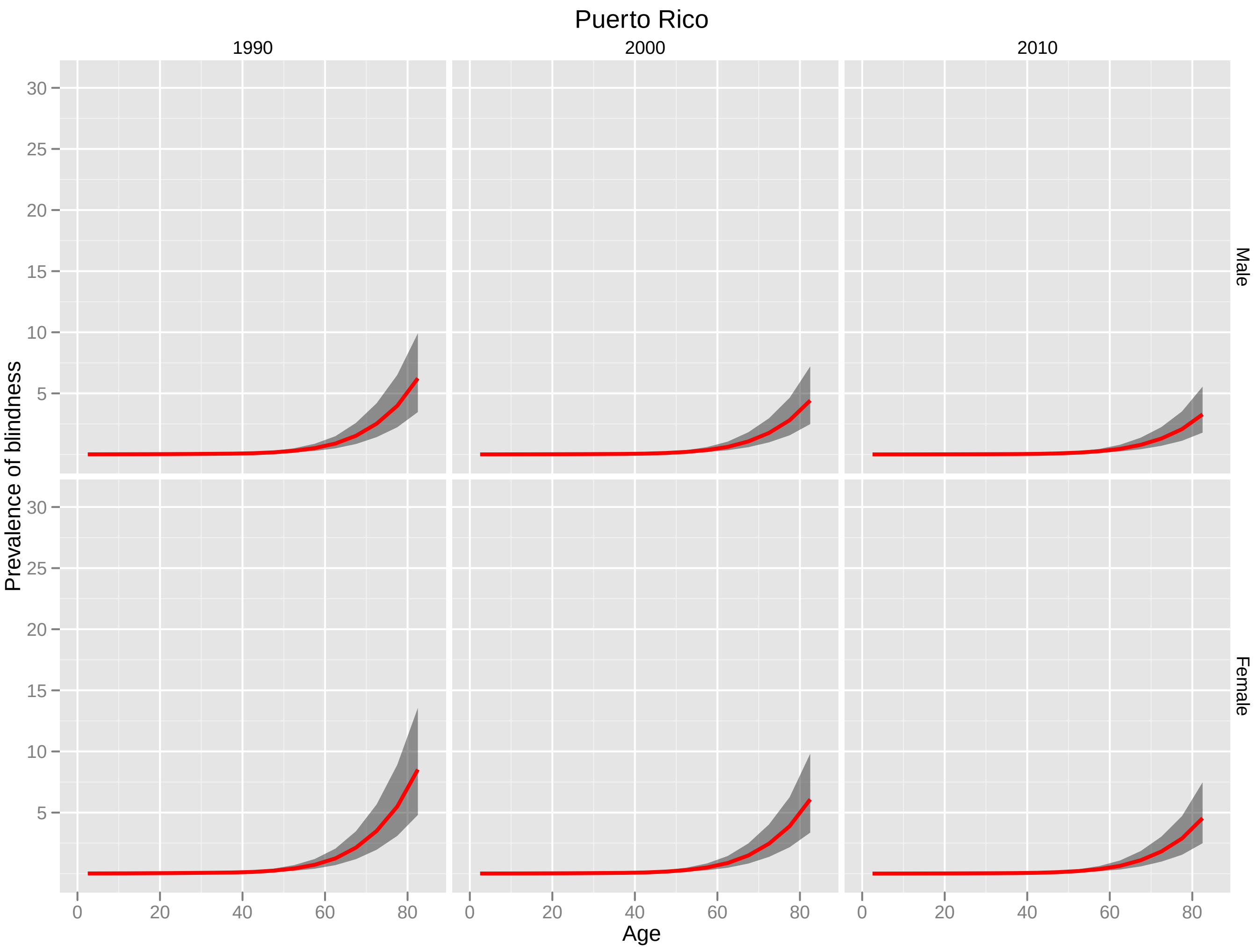

Puerto Rico
1990
2000
2010
Male
Female
Prevalence of blindness
Age
0
20
40
60
80
5
10
15
20
25
30

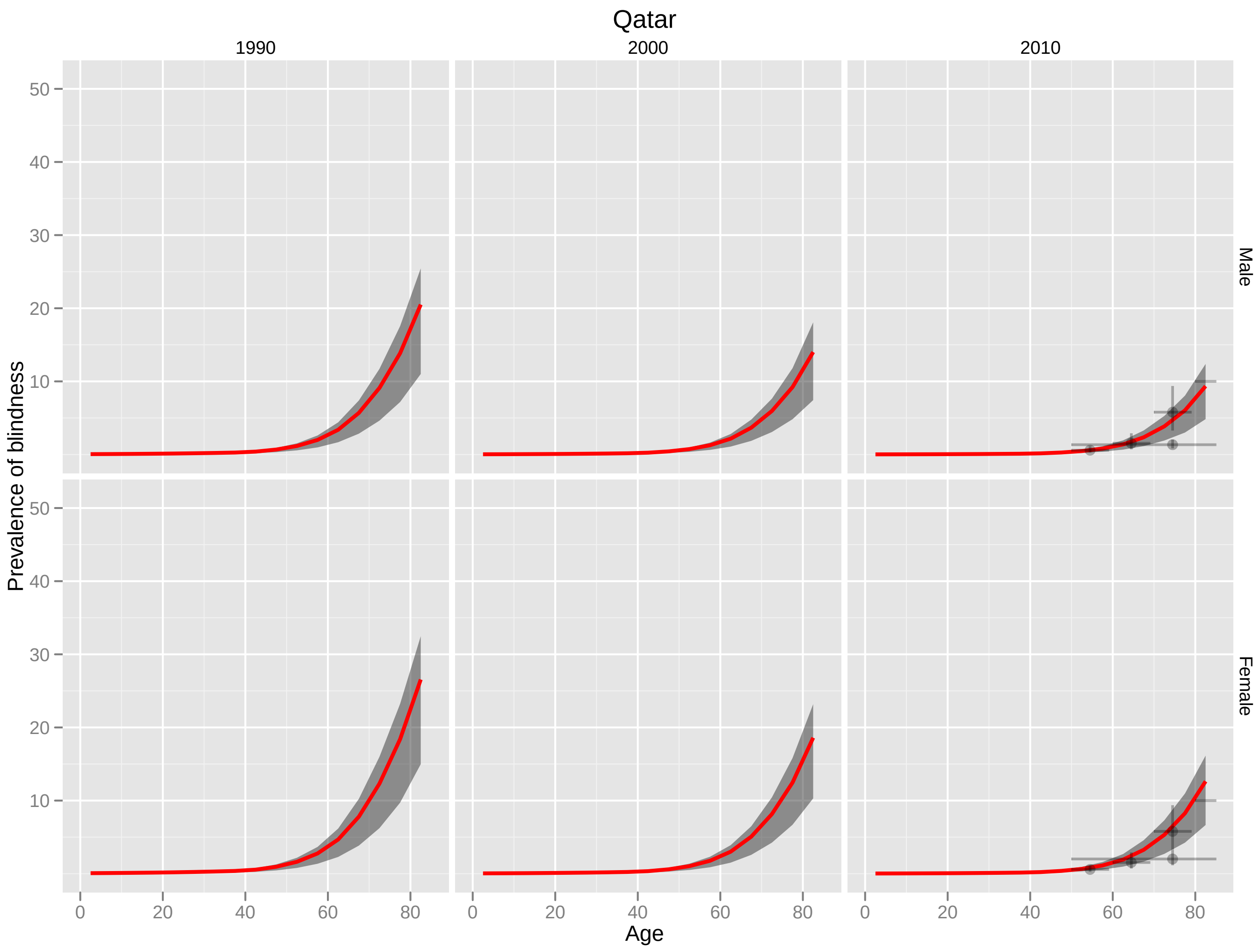
Qatar
1990
2000
2010
Male
Female
Prevalence of blindness
Age
0
20
40
60
80
10
20
30
40
50

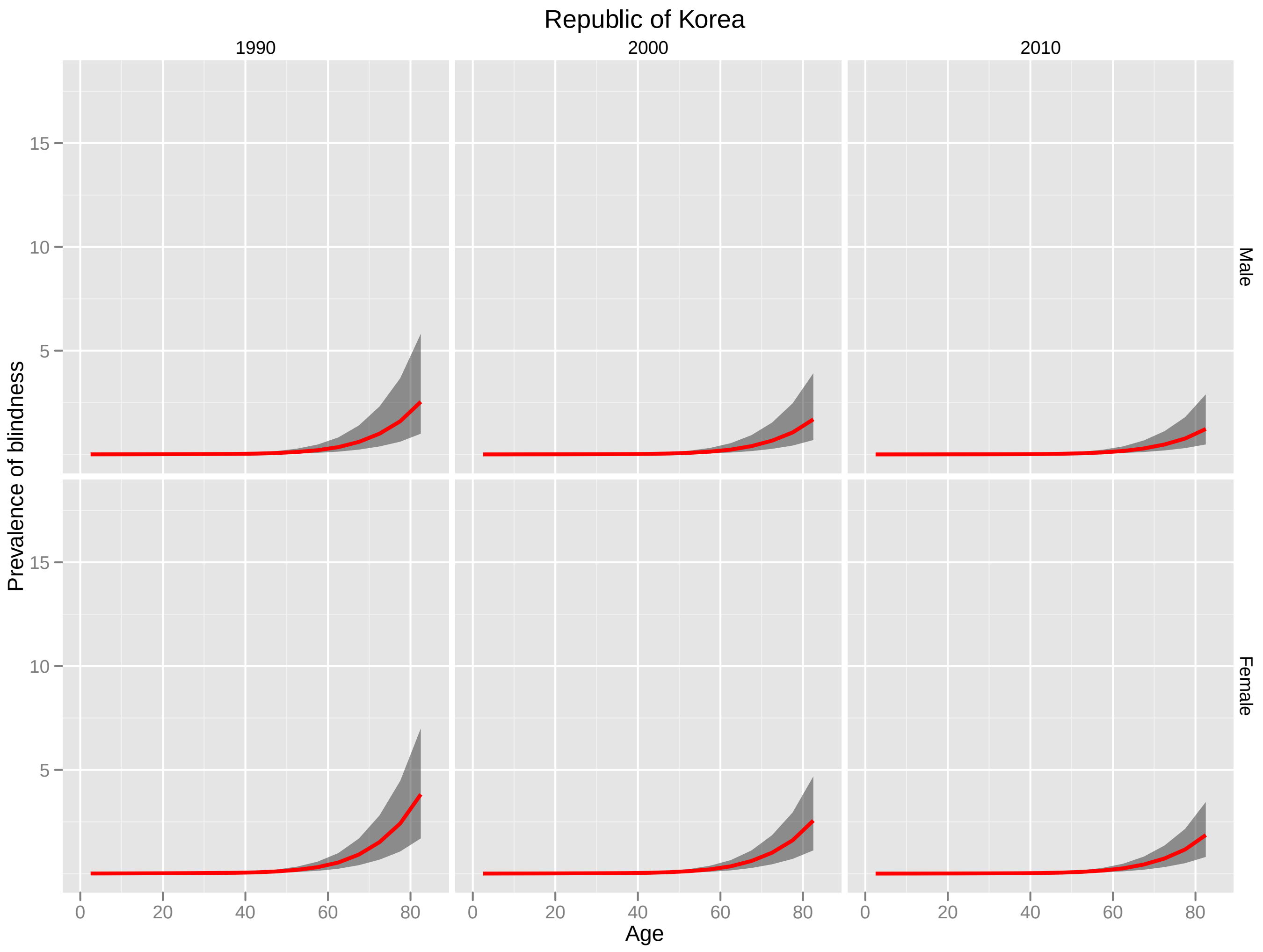
Republic of Korea
1990
2000
2010
Male
Female
Prevalence of blindness
Age
0
20
40
60
80
5
10
15

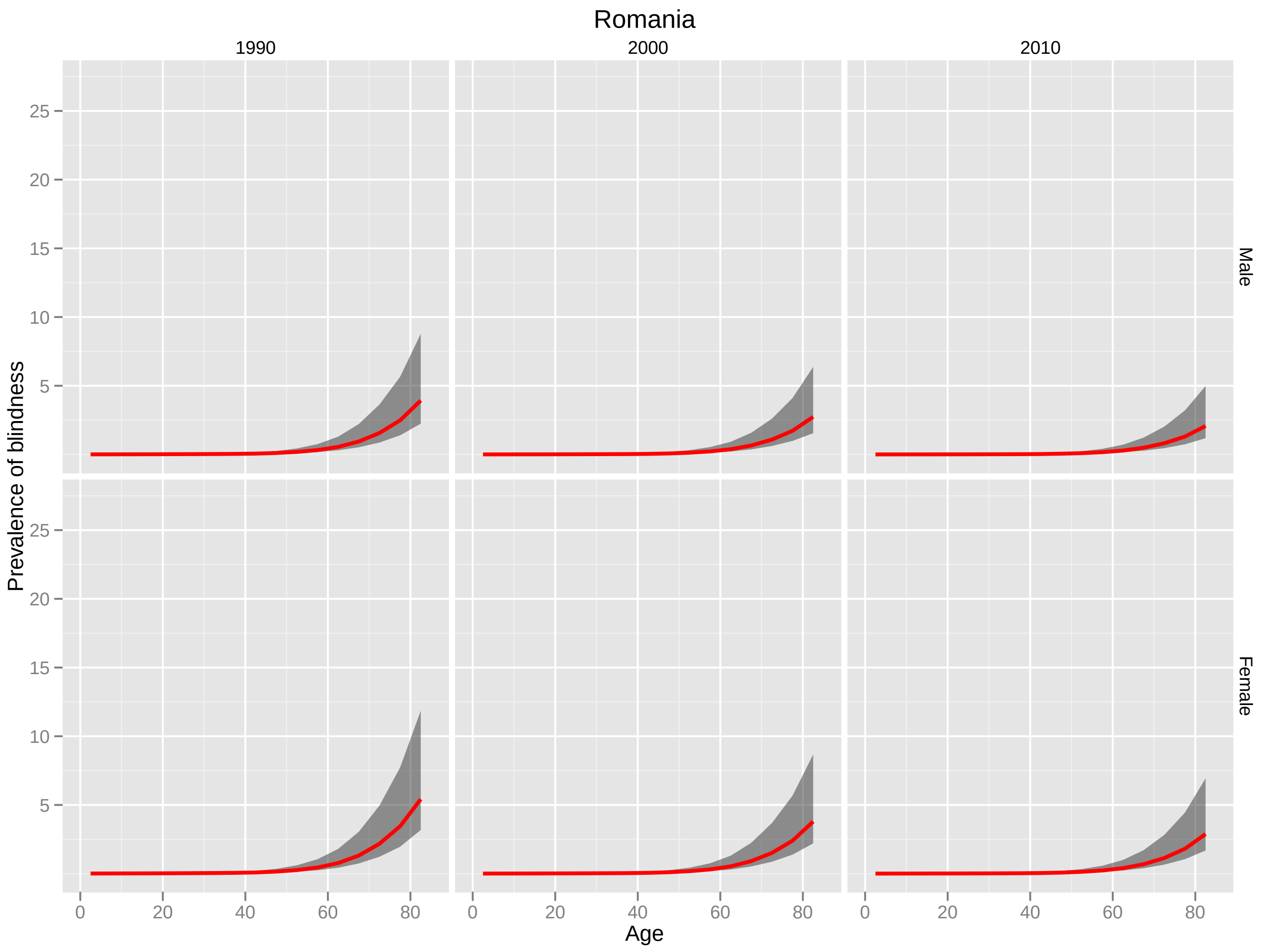

Romania
1990
2000
2010
Male
Female
Prevalence of blindness
Age
25
20
15
10
5
0
20
40
60
80

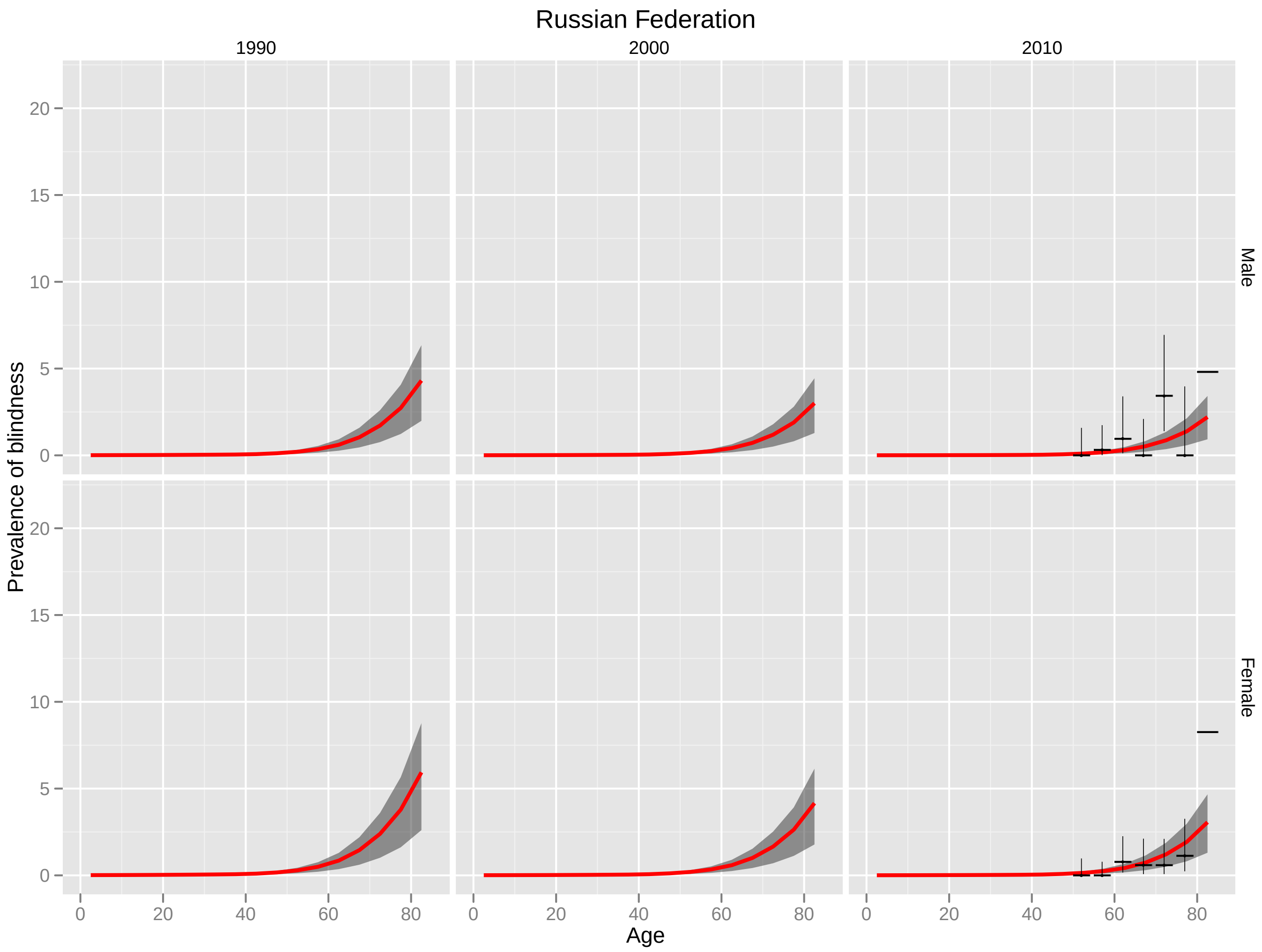
Russian Federation
1990
2000
2010
Male
Female
Prevalence of blindness
Age
0
5
10
15
20
0
20
40
60
80

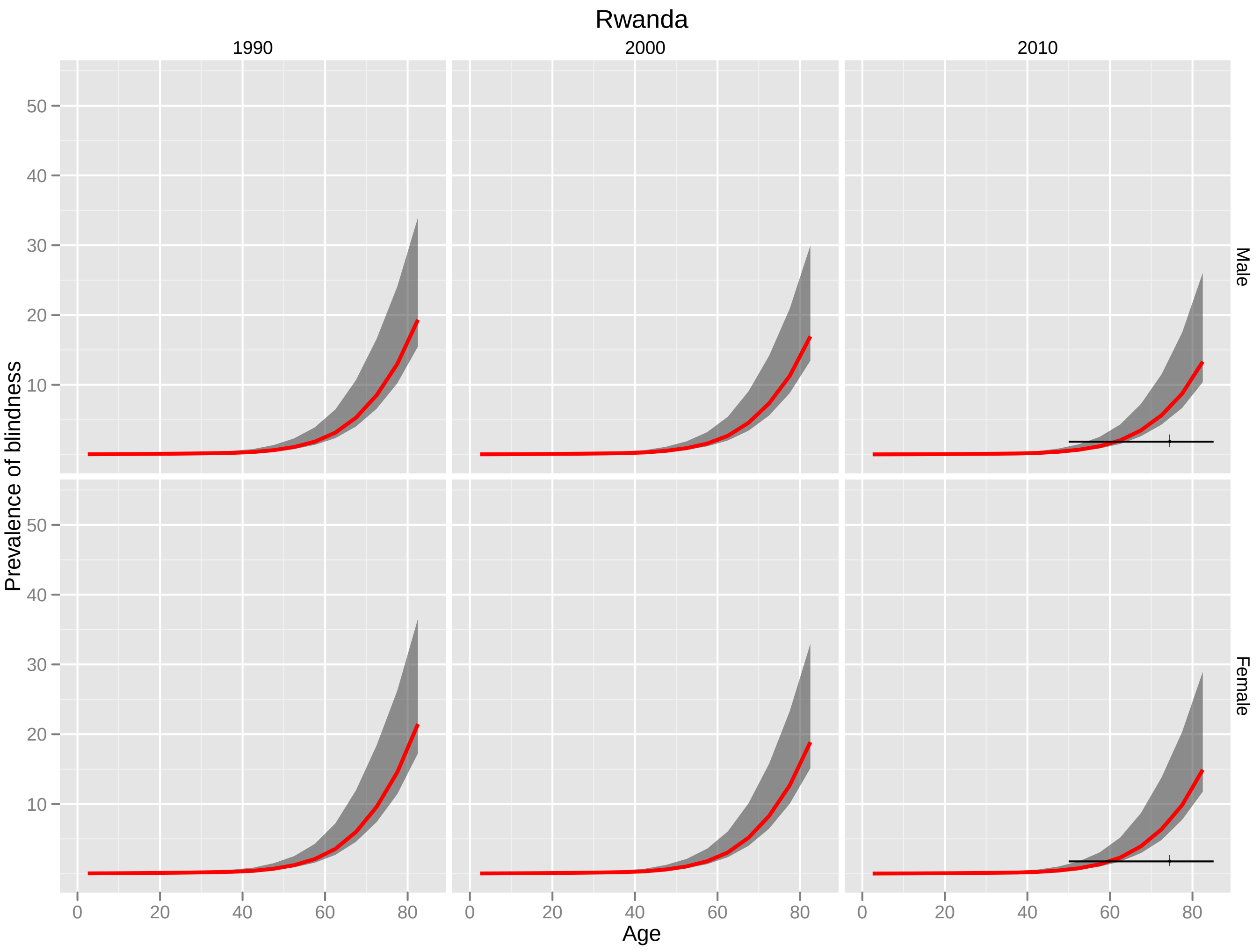

Rwanda
1990
2000
2010
Male
Female
Prevalence of blindness
Age
10
20
30
40
50
0
20
40
60
80

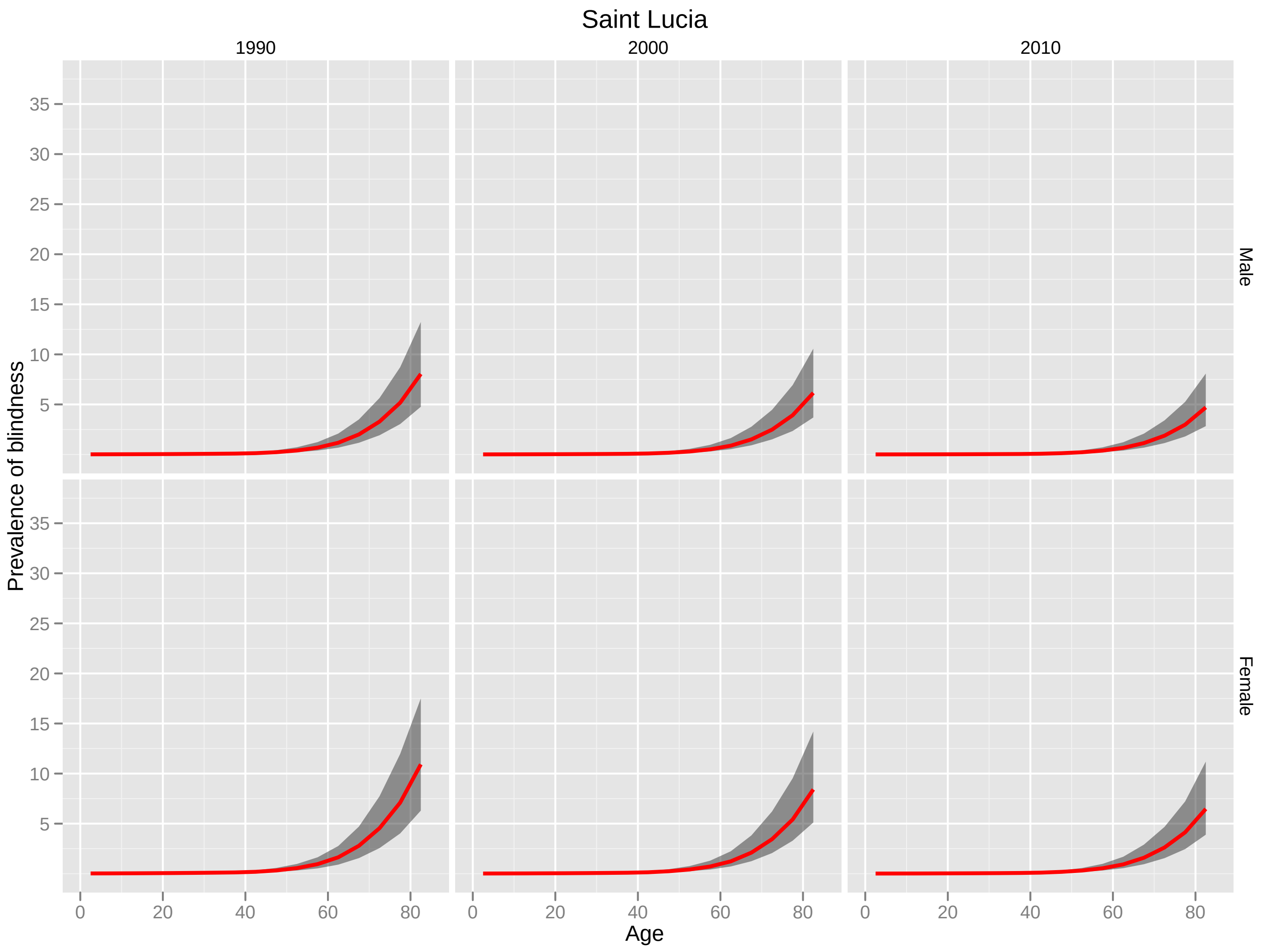
Saint Lucia
1990
2000
2010
Male
Female
Prevalence of blindness
Age
35
30
25
20
15
10
5
0
20
40
60
80

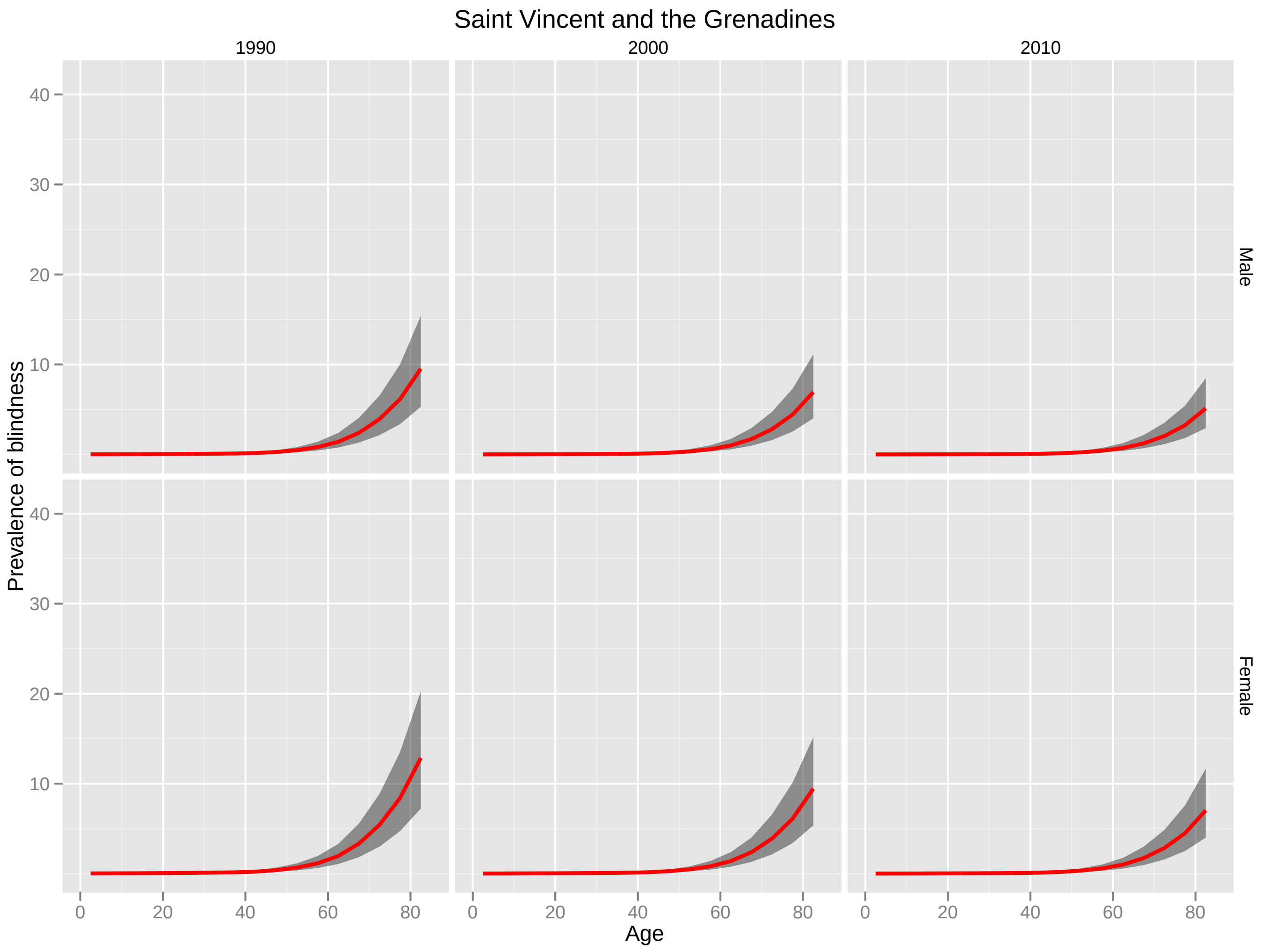
Saint Vincent and the Grenadines
1990
2000
2010
Male
Female
Prevalence of blindness
40
30
20
10
0
20
40
60
80
Age

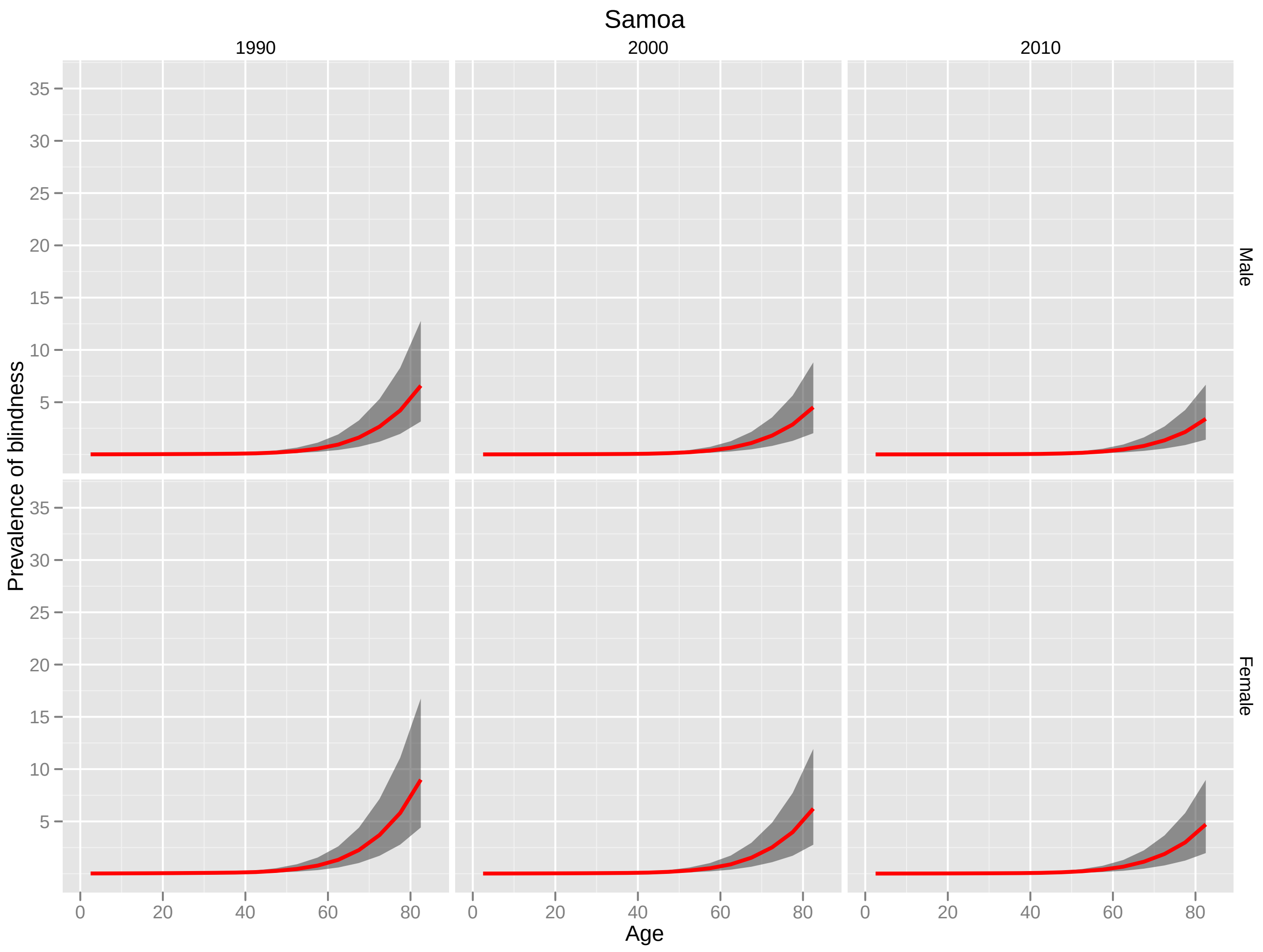
Samoa
1990
2000
2010
Male
Female
Prevalence of blindness
Age
35
30
25
20
15
10
5
0
20
40
60
80

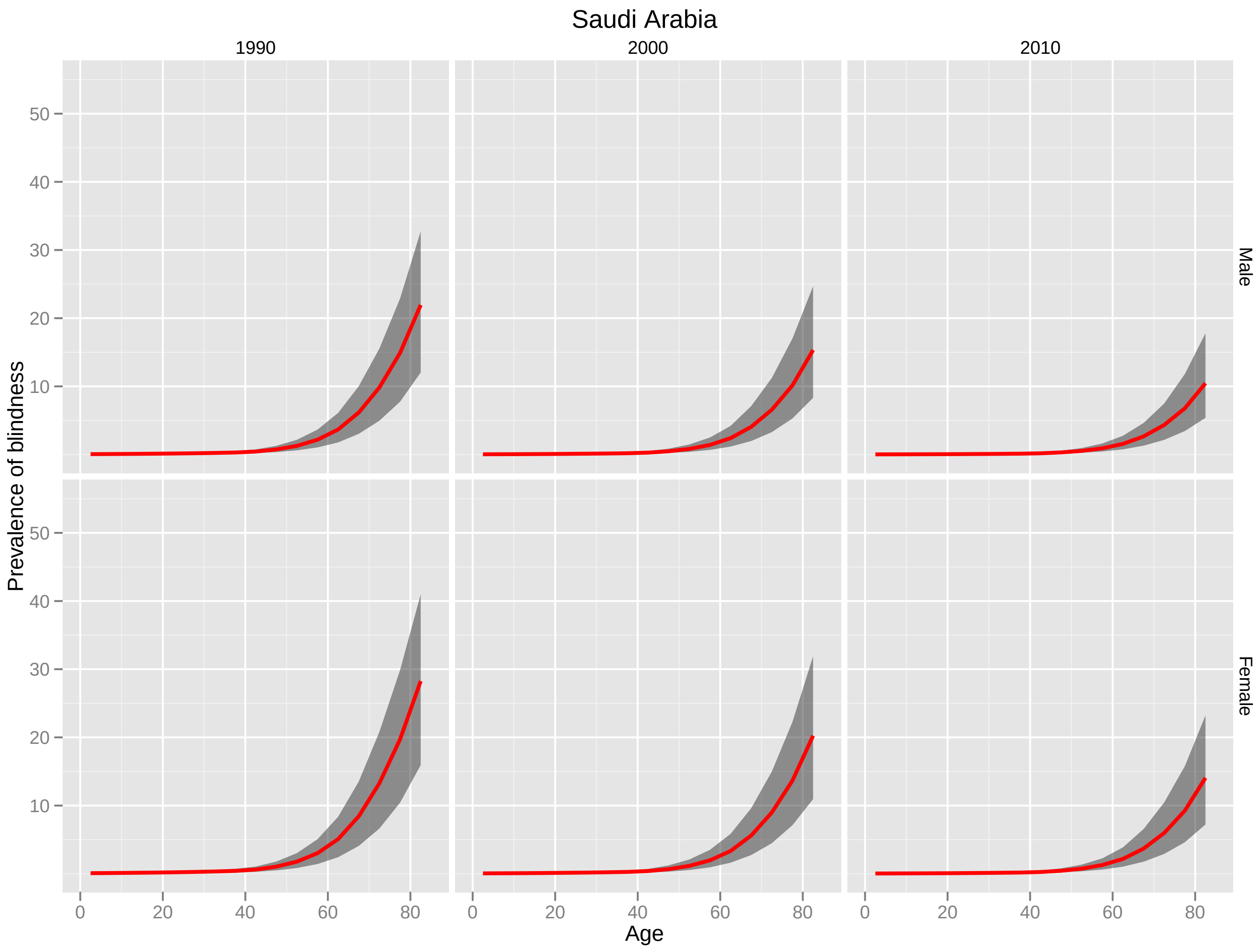
Saudi Arabia
1990
2000
2010
Male
Female
Prevalence of blindness
Age
10
20
30
40
50
0
20
40
60
80

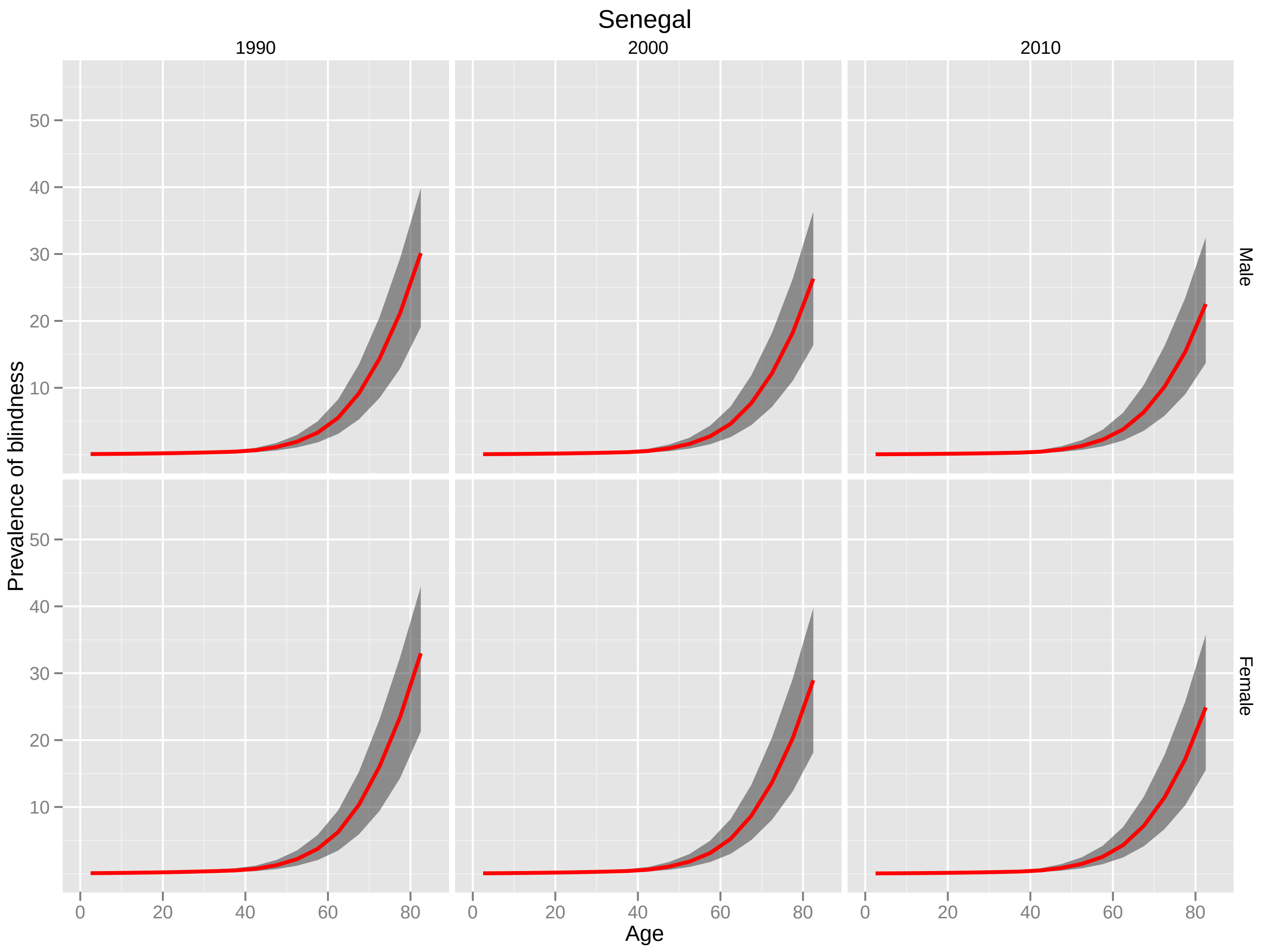
Senegal
1990
2000
2010
Male
Female
Prevalence of blindness
Age
10
20
30
40
50
0
20
40
60
80

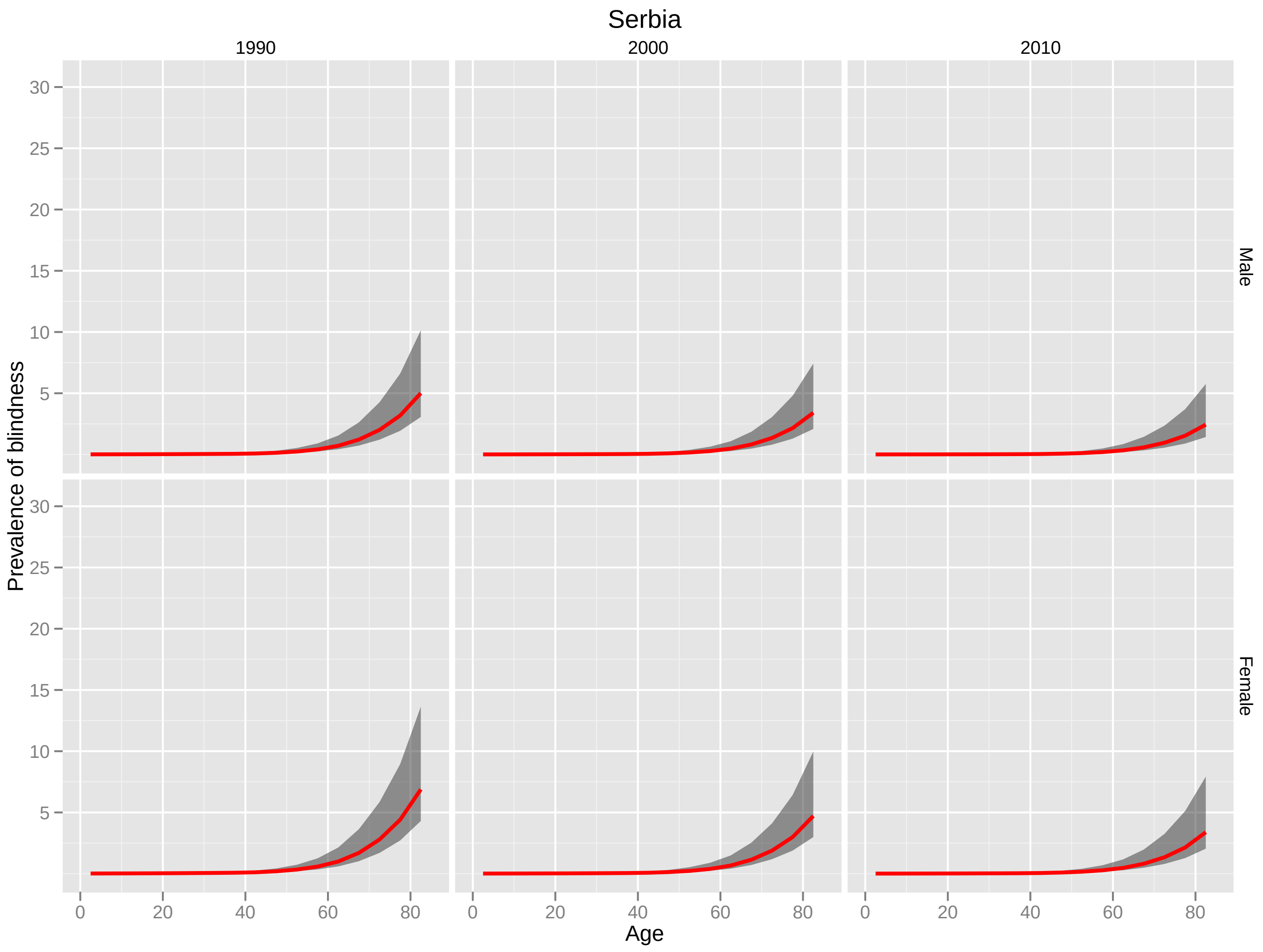
Serbia
1990
2000
2010
Male
Female
Prevalence of blindness
Age
30
25
20
15
10
5
0
20
40
60
80

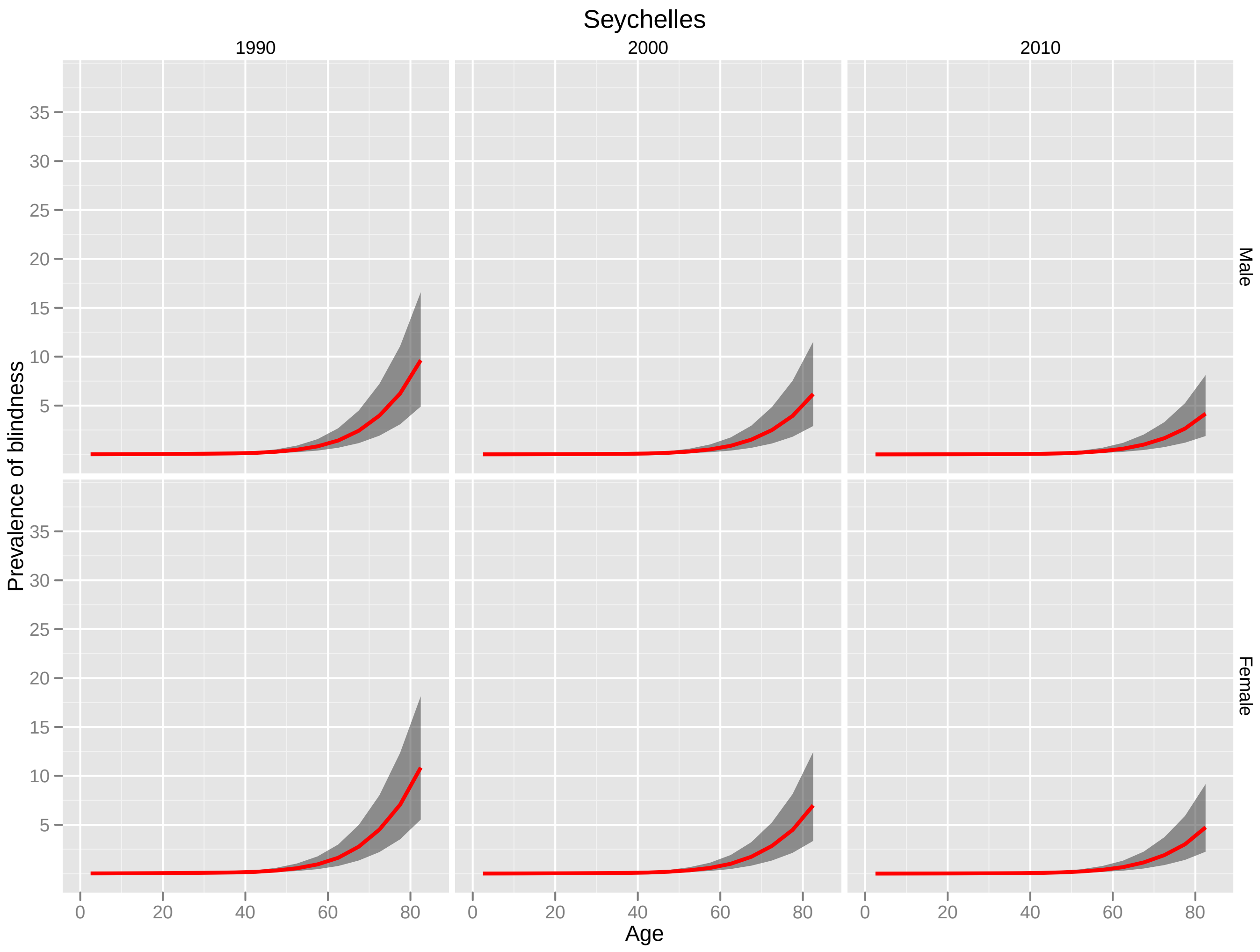
Seychelles
1990
2000
2010
Male
Female
Prevalence of blindness
35
30
25
20
15
10
5
0
20
40
60
80
Age

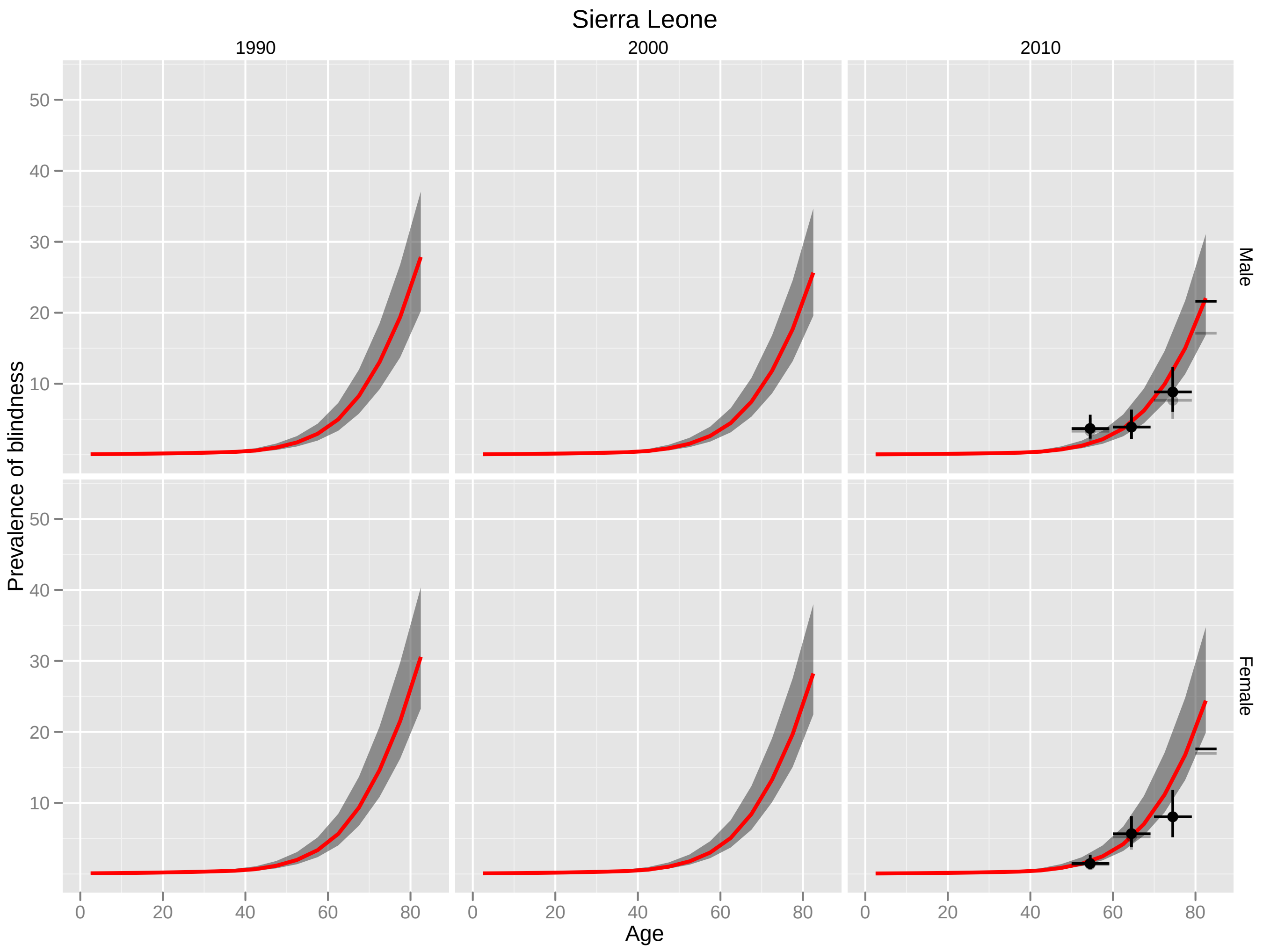

Sierra Leone
1990
2000
2010
Male
Female
Prevalence of blindness
Age
0
20
40
60
80
10
20
30
40
50

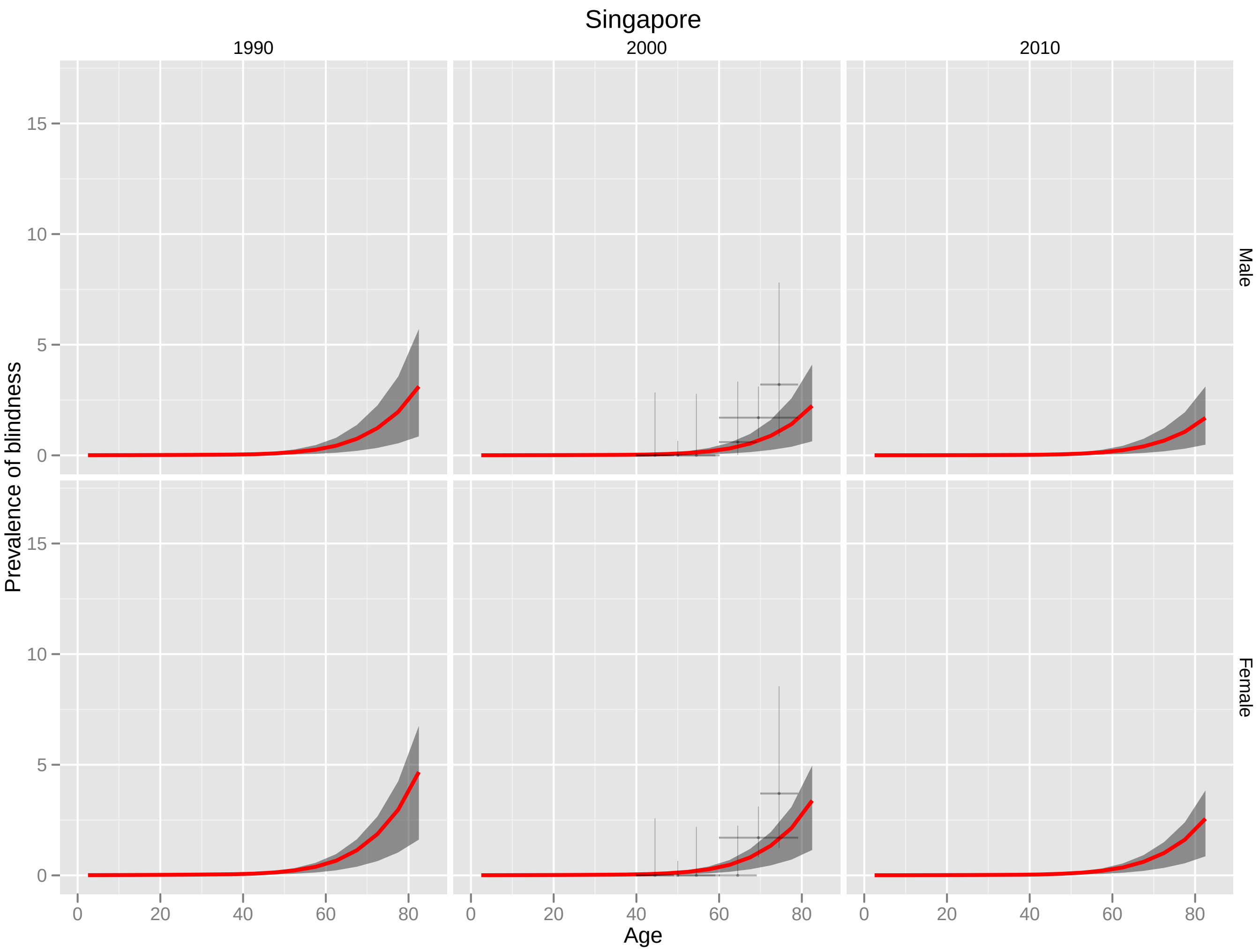
Singapore
1990
2000
2010
Male
Female
Prevalence of blindness
Age
0
5
10
15
0
20
40
60
80

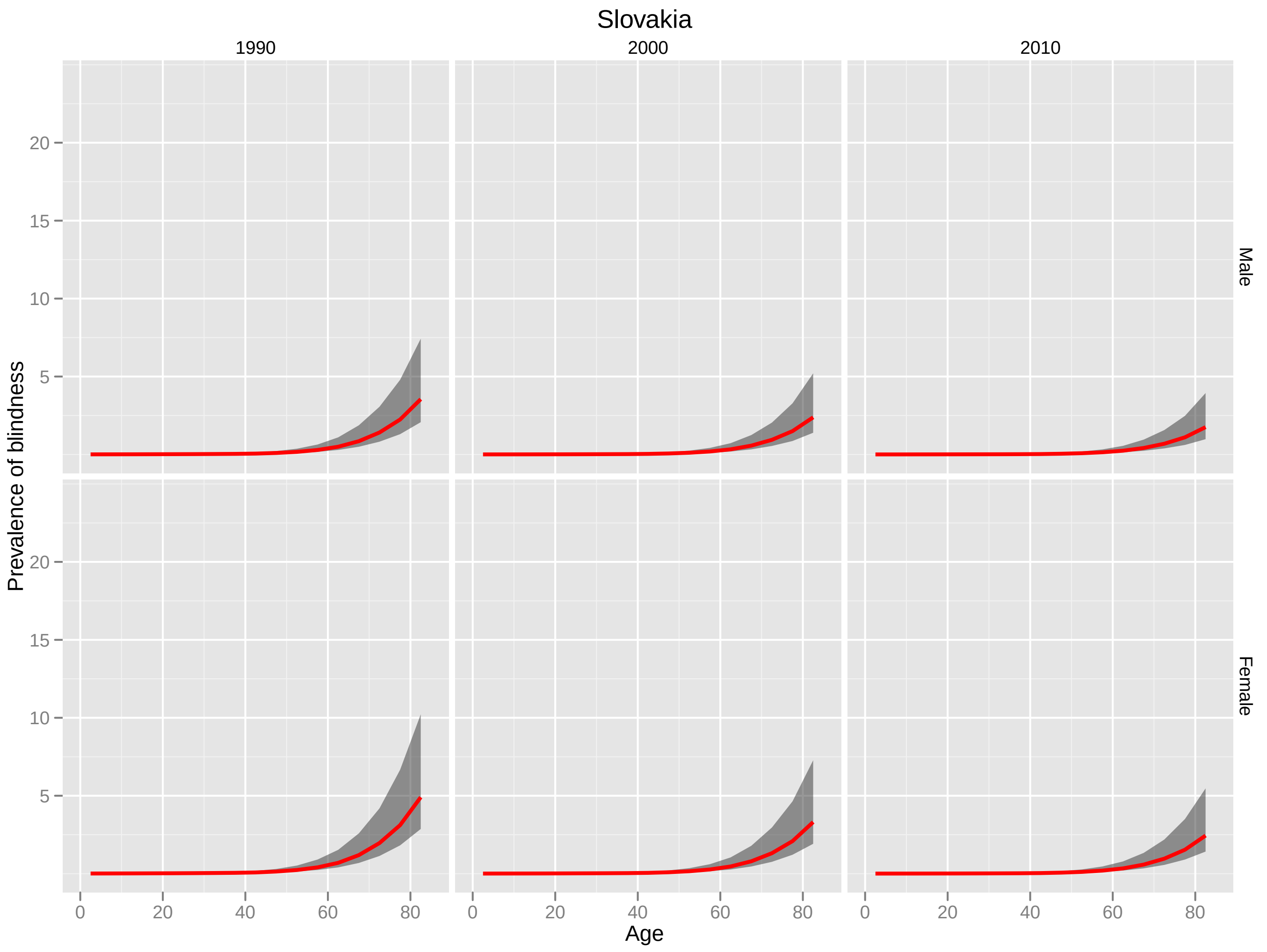
Slovakia
1990
2000
2010
Male
Female
Prevalence of blindness
Age
0
20
40
60
80
5
10
15
20

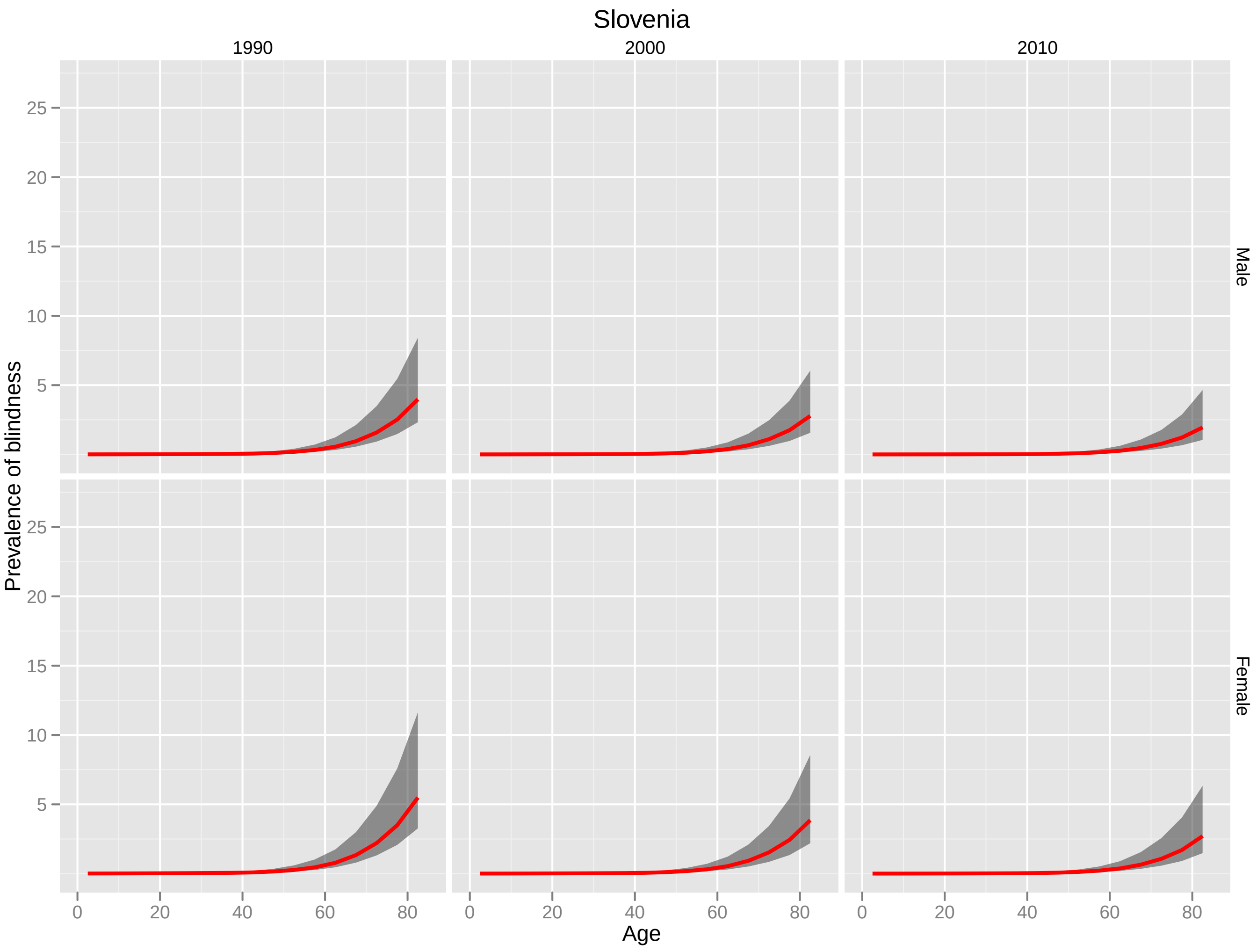
Slovenia
1990
2000
2010
Male
Female
Prevalence of blindness
Age
25
20
15
10
5
0
20
40
60
80

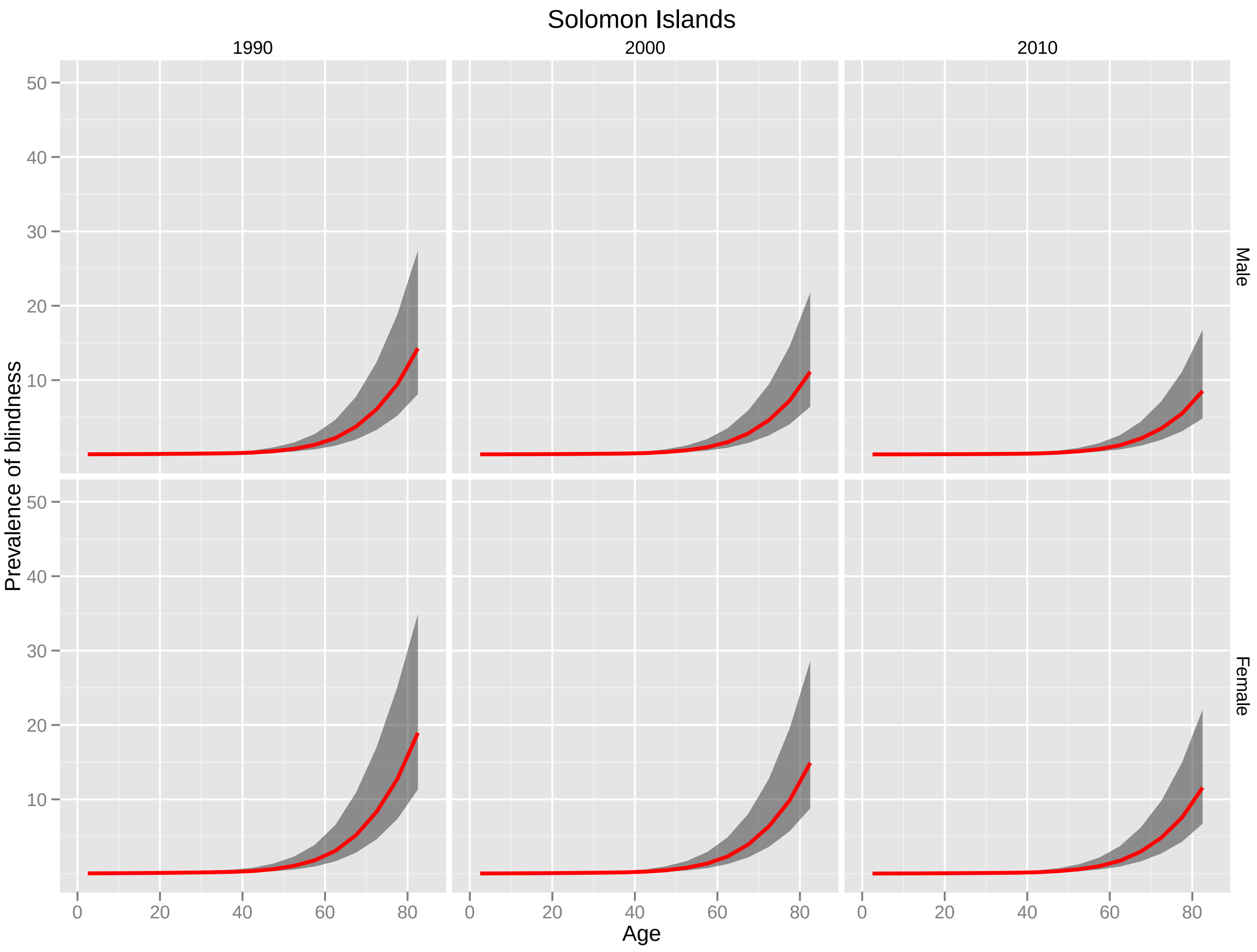
Solomon Islands
1990
2000
2010
Male
Female
Prevalence of blindness
Age
10
20
30
40
50
0
20
40
60
80

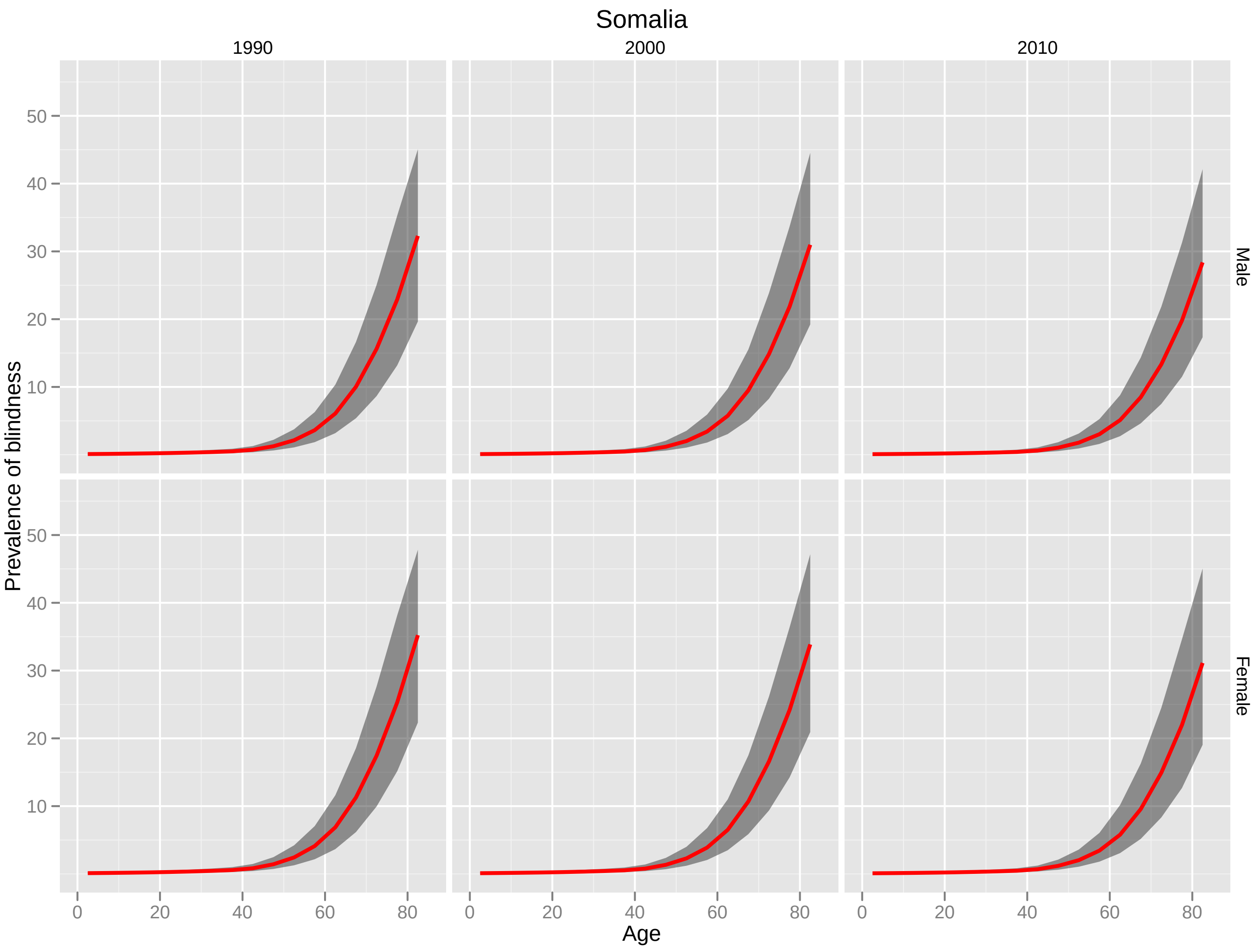
Somalia
1990
2000
2010
Male
Female
Prevalence of blindness
Age
10
20
30
40
50
0
20
40
60
80

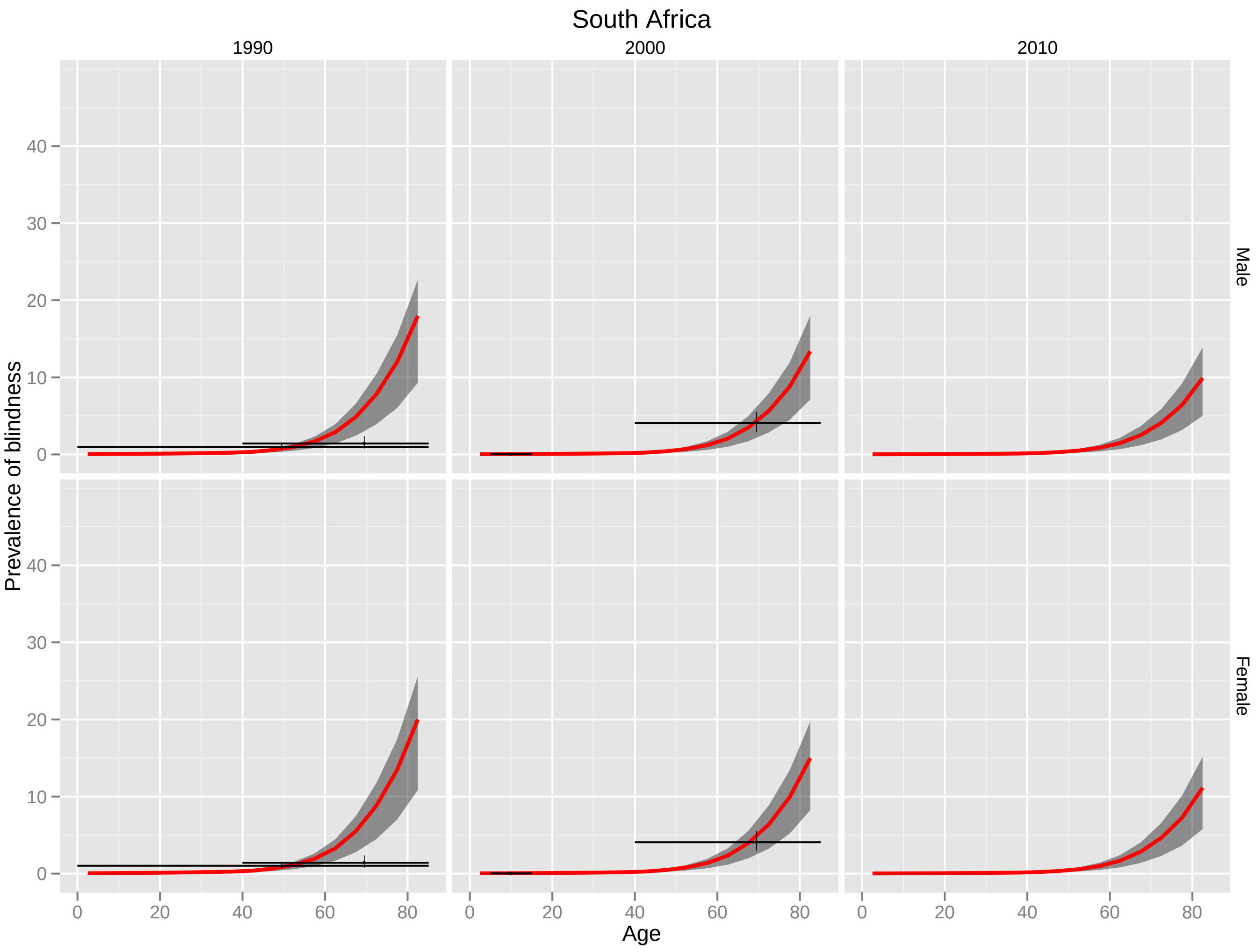
South Africa
1990
2000
2010
Male
Female
Prevalence of blindness
0
10
20
30
40
Age
0
20
40
60
80

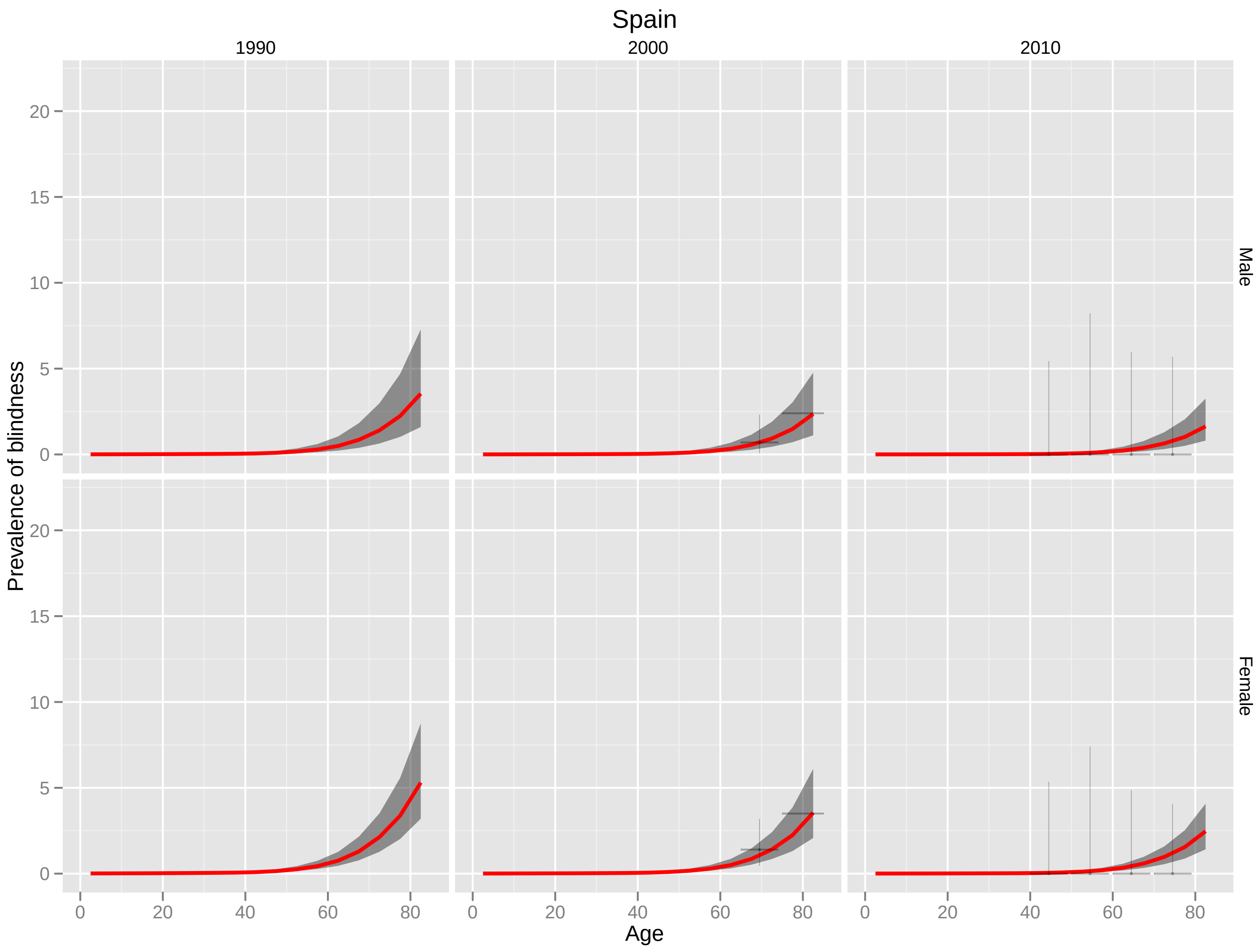
Spain
1990
2000
2010
Male
Female
Prevalence of blindness
Age
0
5
10
15
20
0
20
40
60
80

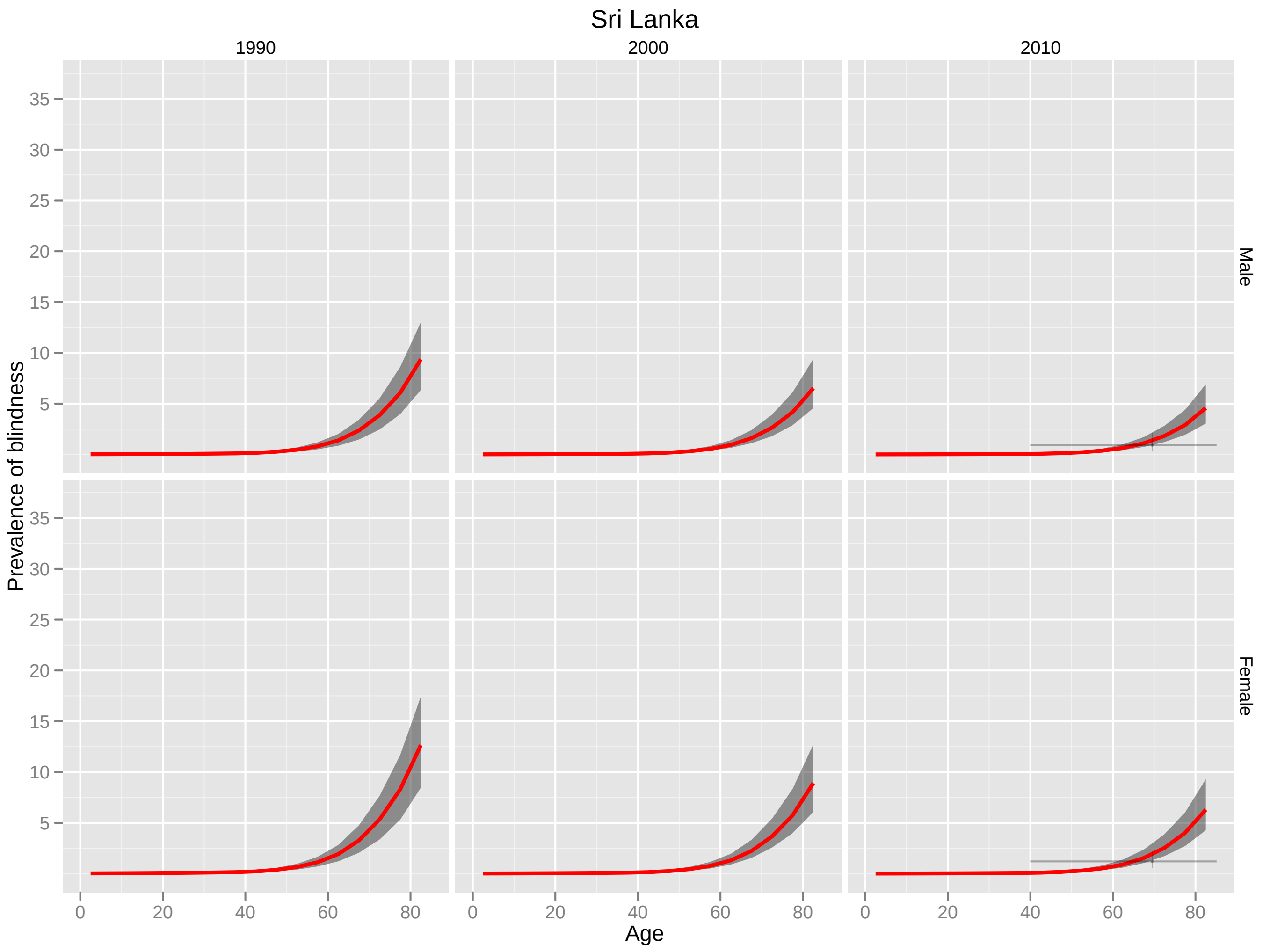

Sri Lanka
1990
2000
2010
Male
Female
Prevalence of blindness
Age
0
20
40
60
80
5
10
15
20
25
30
35

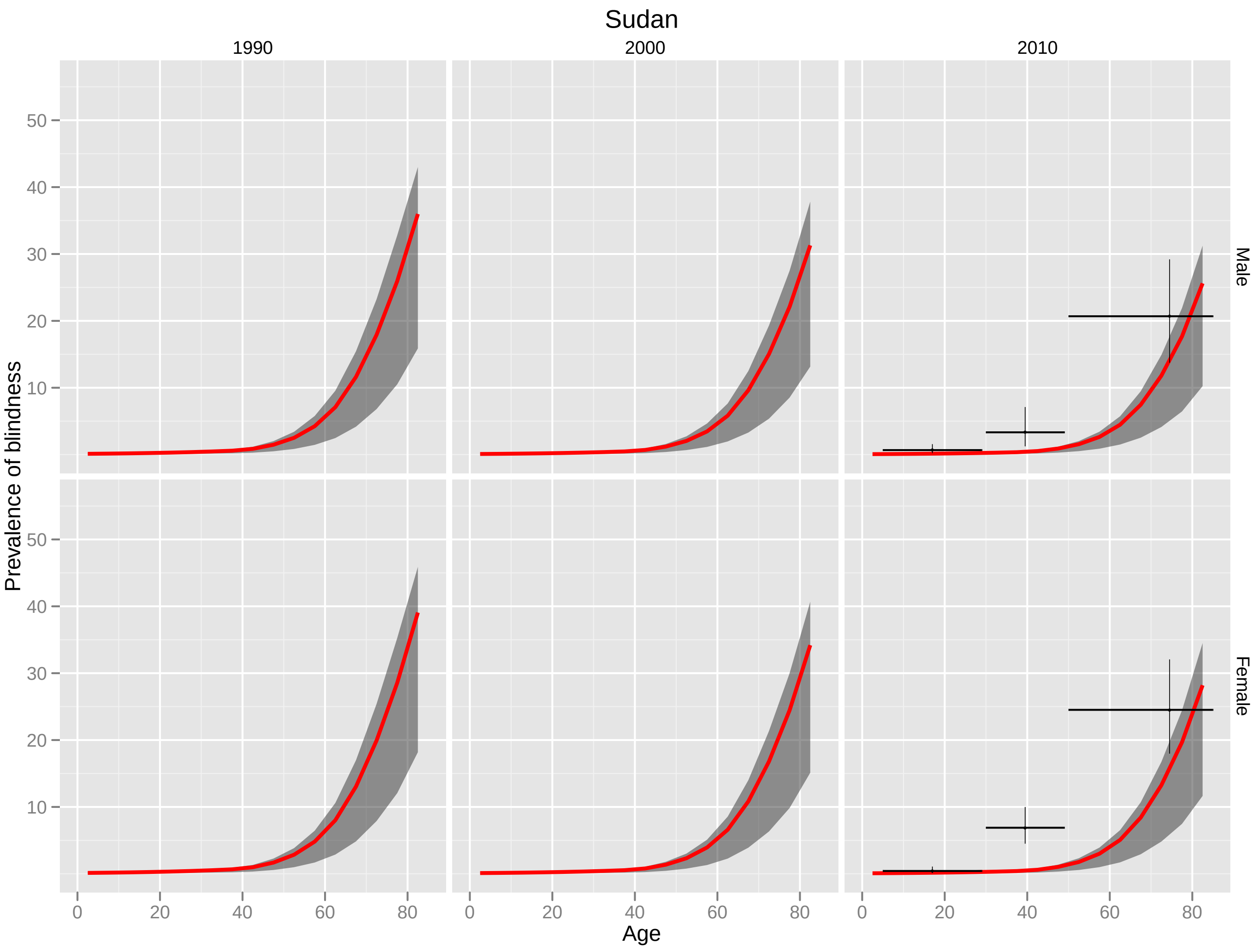
Sudan
1990
2000
2010
Male
Female
Prevalence of blindness
Age
10
20
30
40
50
0
20
40
60
80

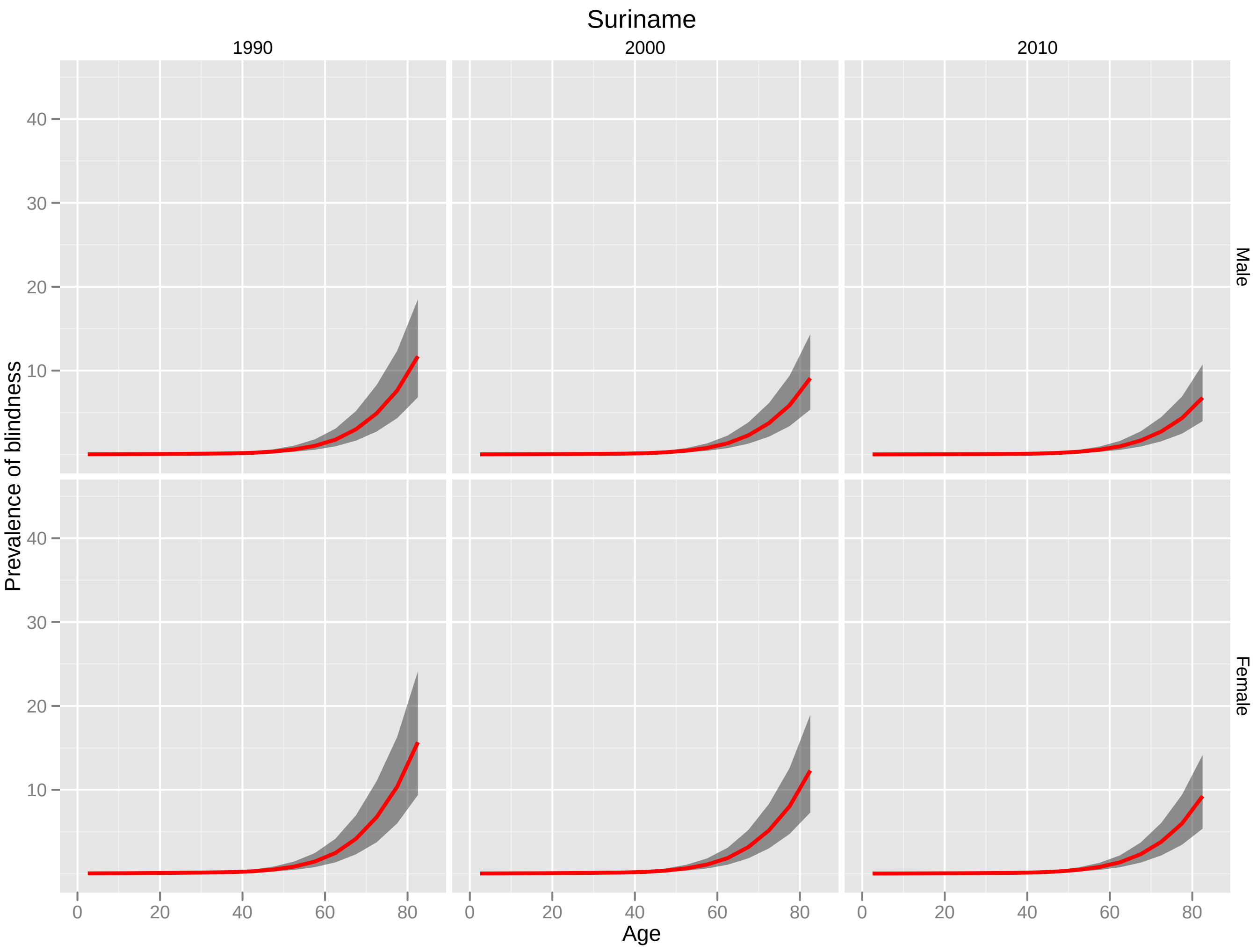
Suriname
1990
2000
2010
Male
Female
Prevalence of blindness
Age
0
10
20
30
40
60
80

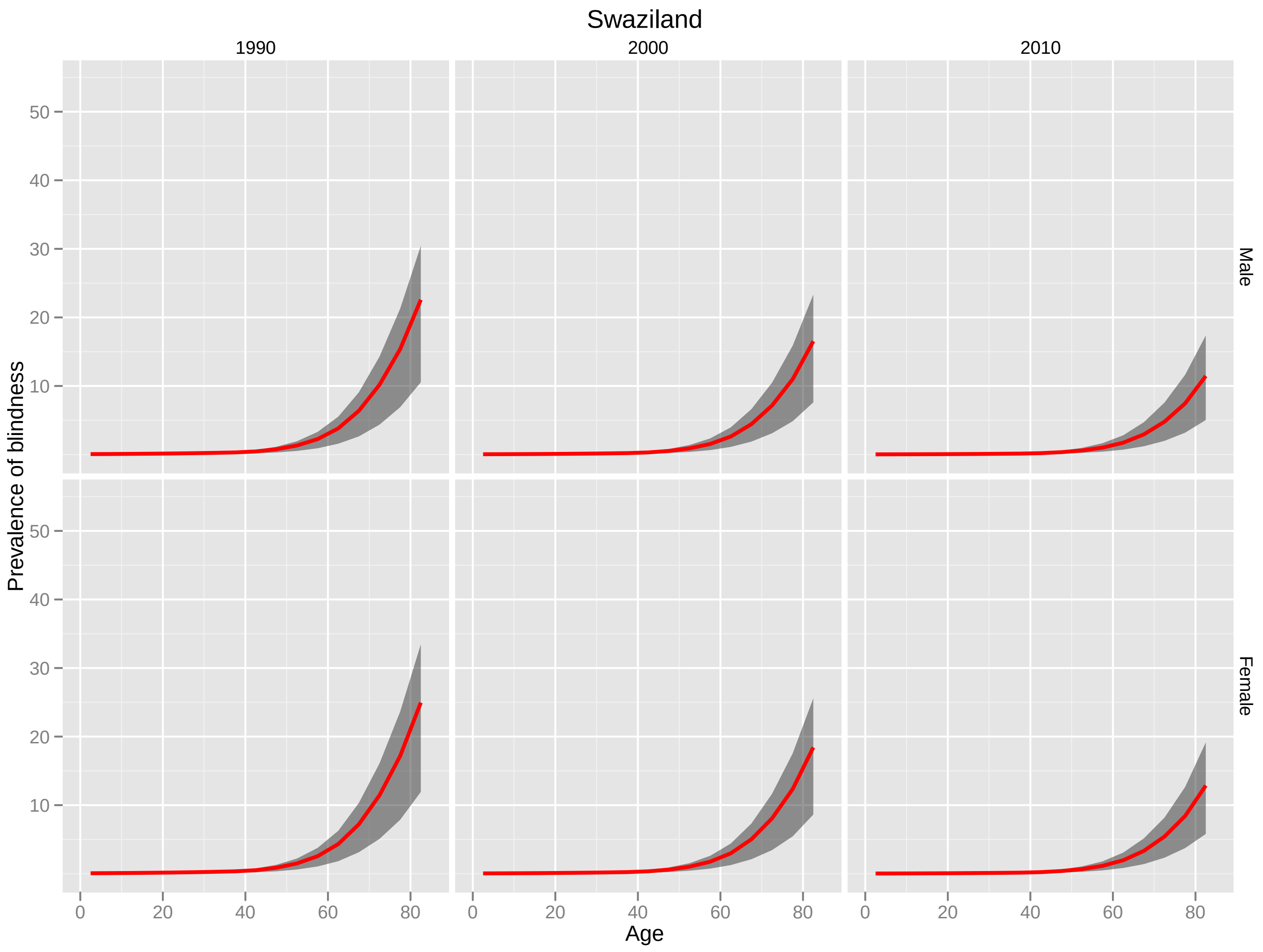
Swaziland
1990
2000
2010
Male
Female
Prevalence of blindness
10
20
30
40
50
Age
0
20
40
60
80

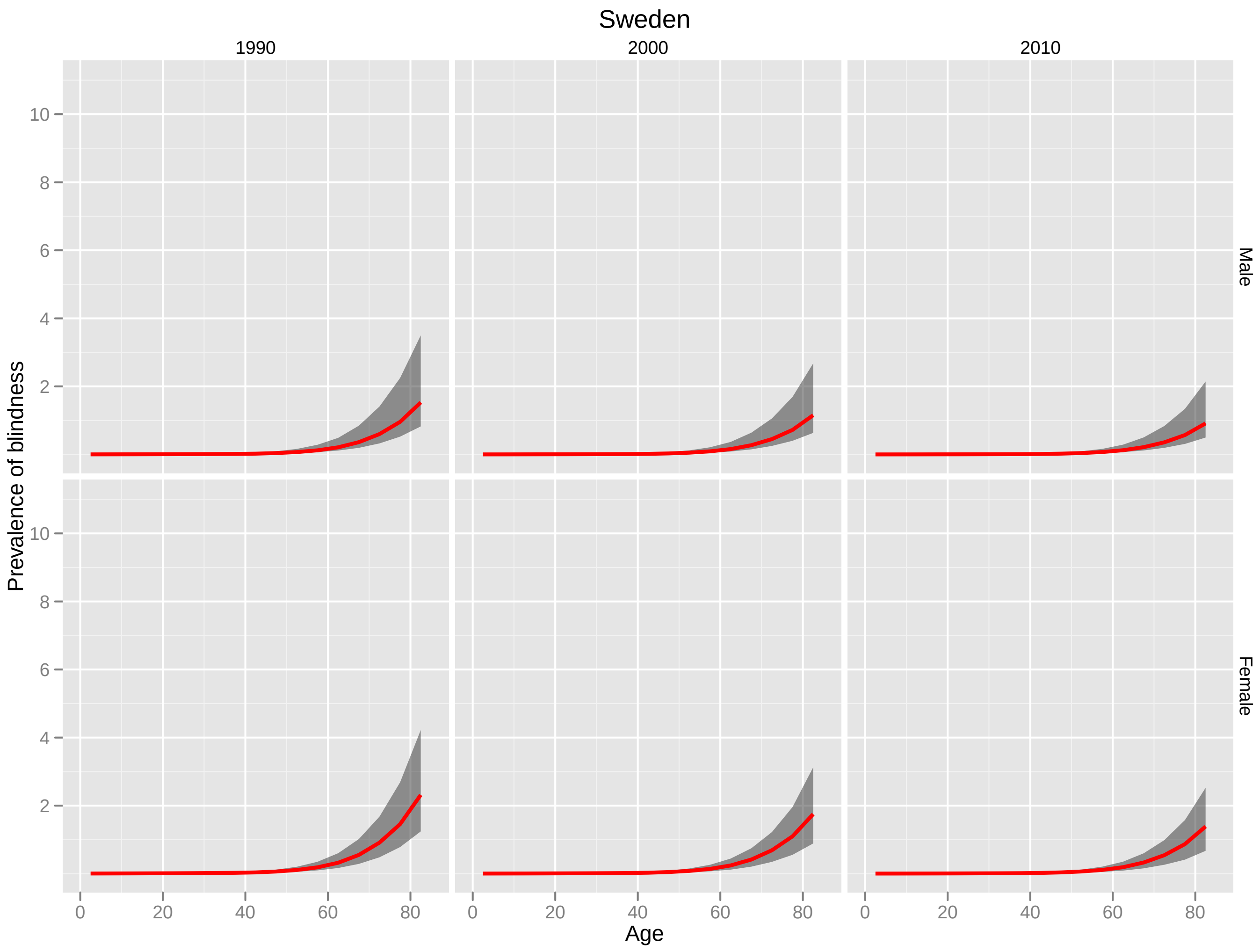
Sweden
1990
2000
2010
Male
Female
Prevalence of blindness
Age
10
8
6
4
2
0
20
40
60
80

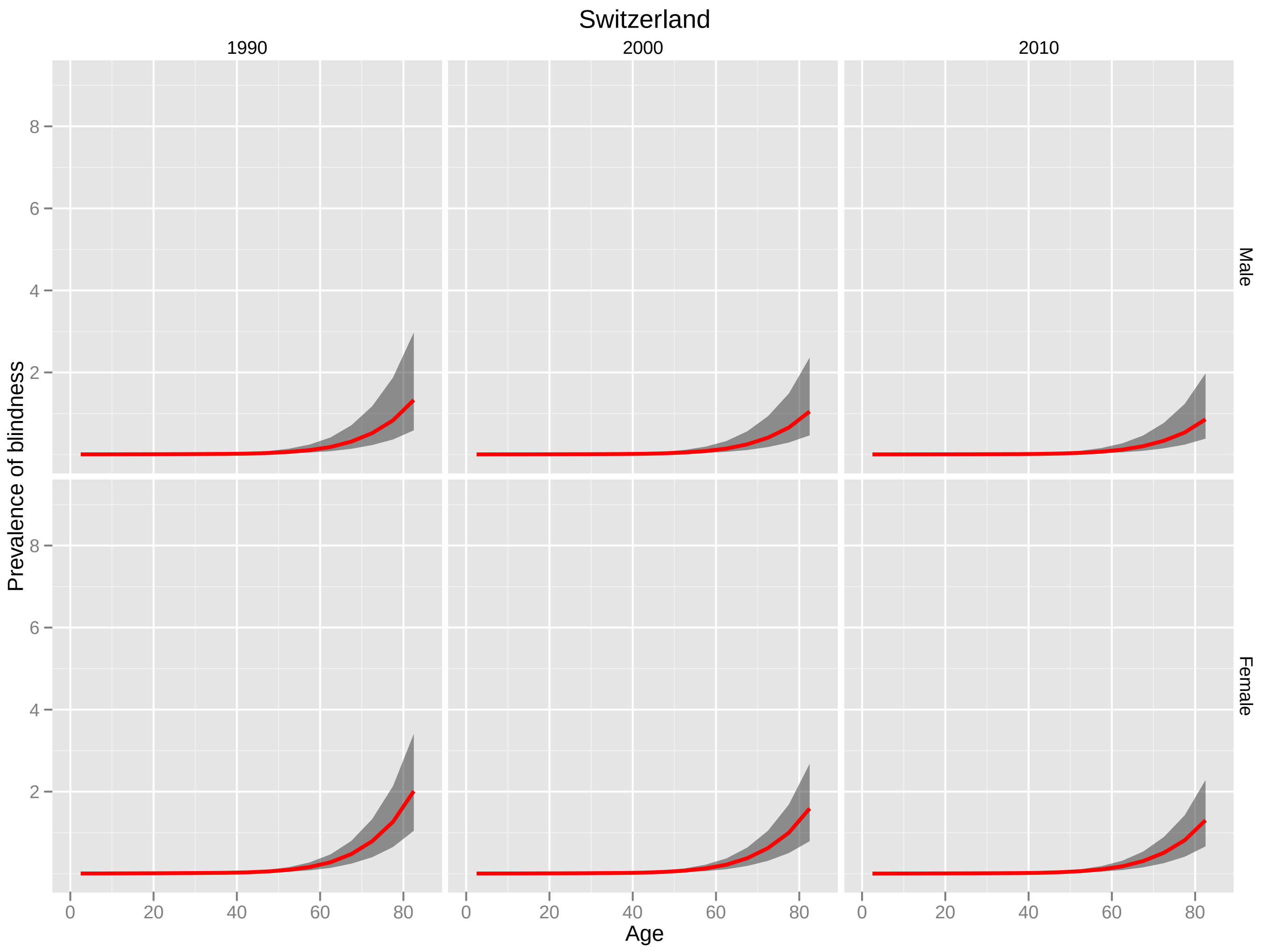
Switzerland
1990
2000
2010
Male
Female
Prevalence of blindness
Age
0
20
40
60
80
2
4
6
8

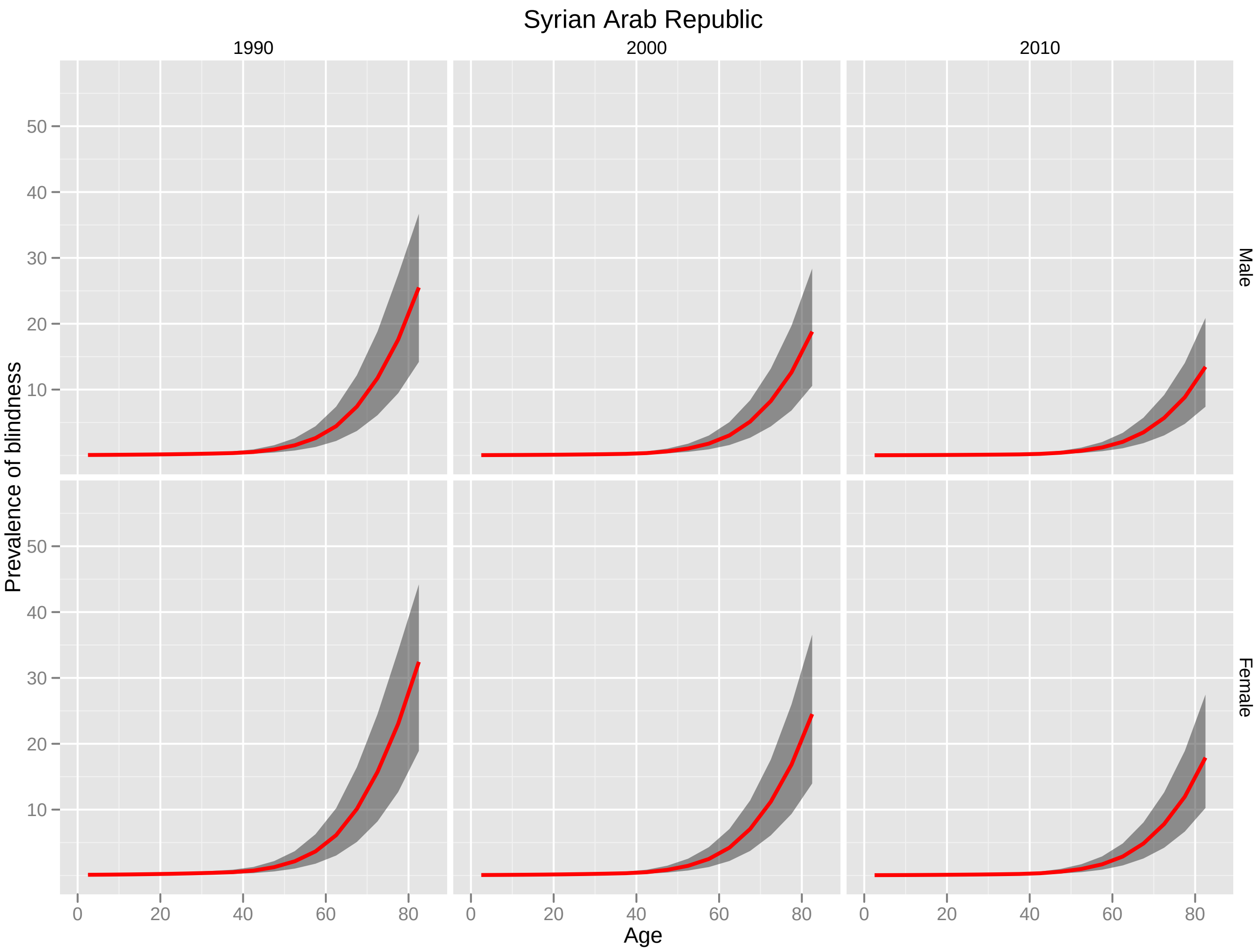
Syrian Arab Republic
1990
2000
2010
Male
Female
Prevalence of blindness
Age
10
20
30
40
50
0
20
40
60
80

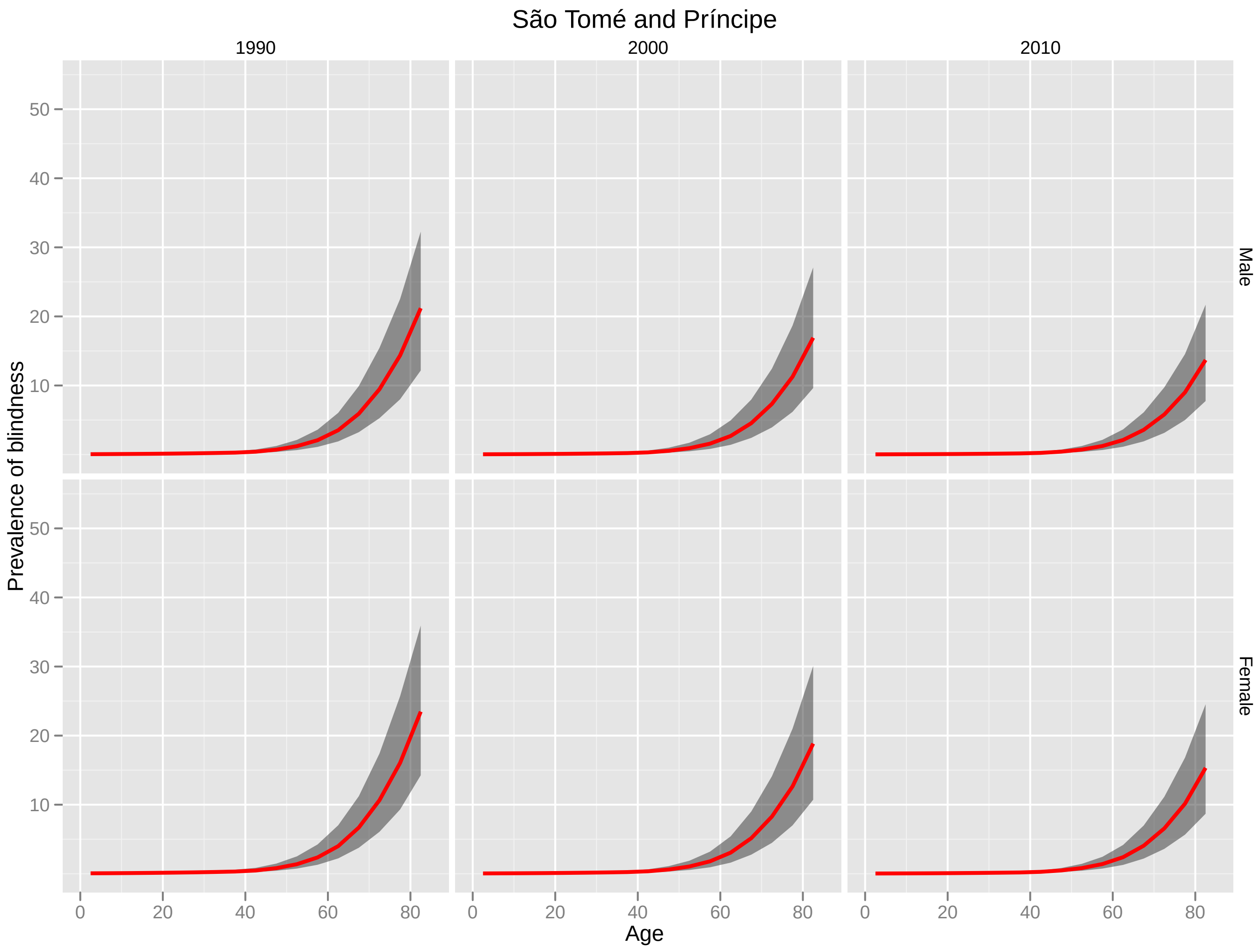
São Tomé and Príncipe
1990
2000
2010
Male
Female
Prevalence of blindness
Age
10
20
30
40
50
0
20
40
60
80

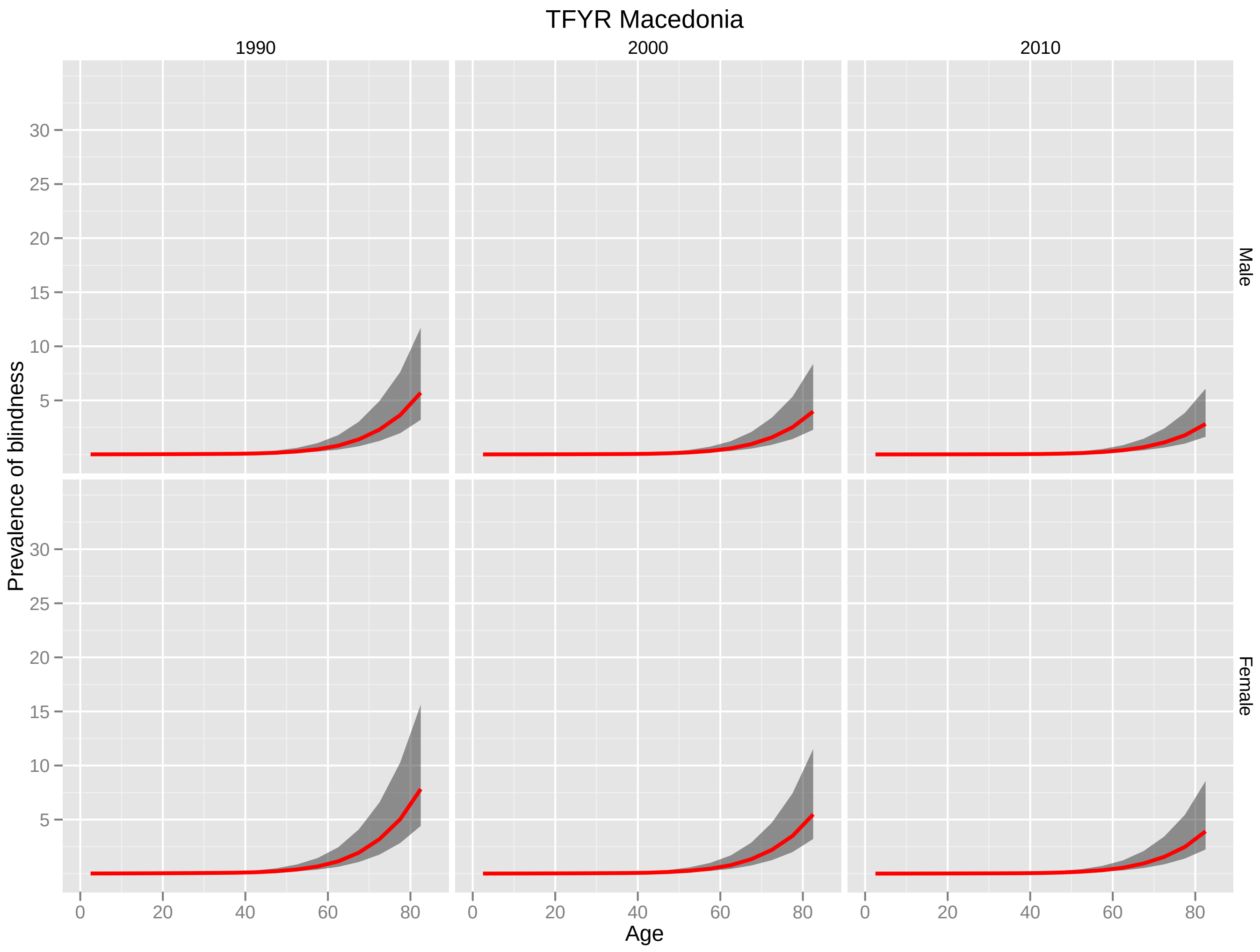

TFYR Macedonia
1990
2000
2010
Male
Female
Prevalence of blindness
Age
30
25
20
15
10
5
0
20
40
60
80

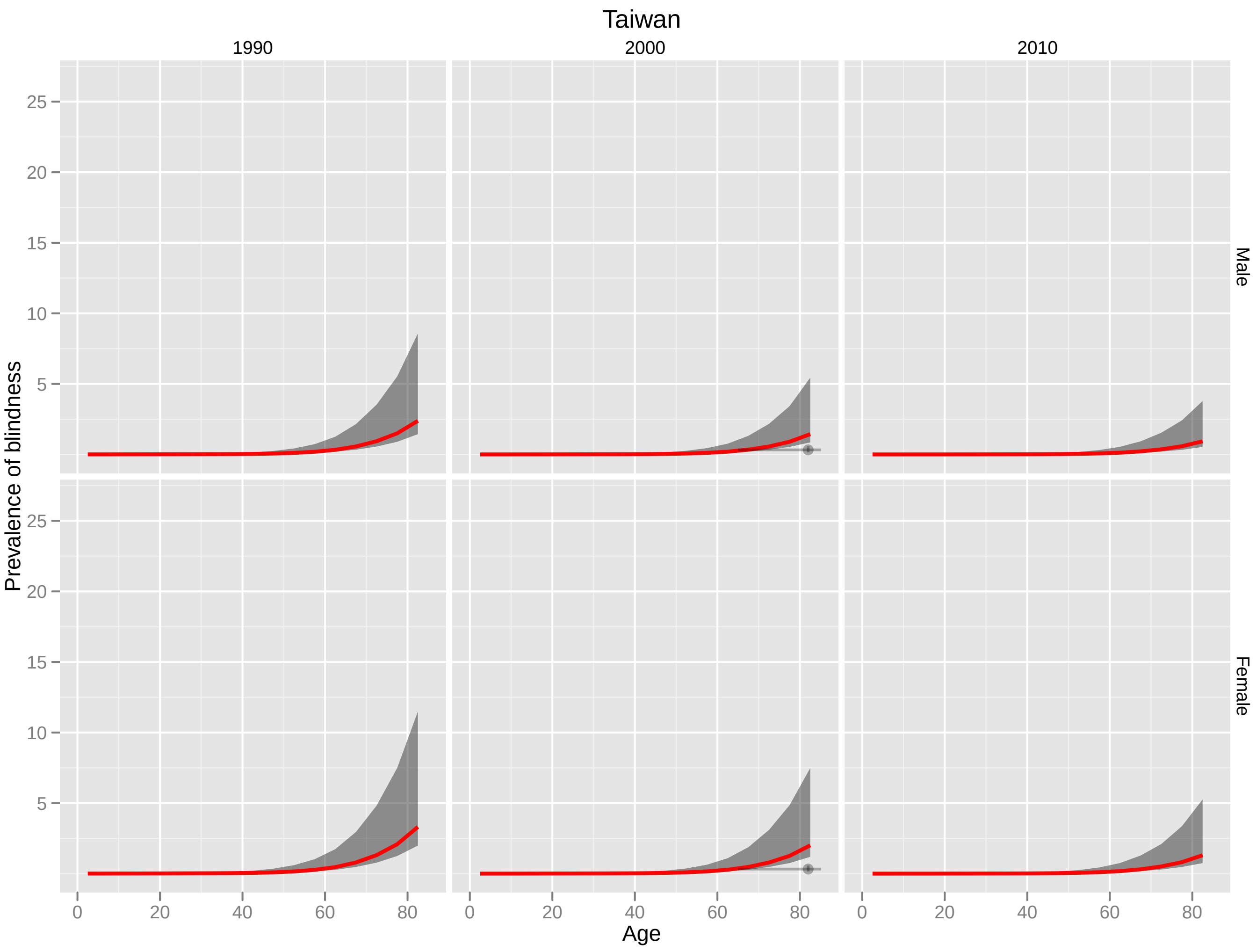
Taiwan
1990
2000
2010
Male
Female
Prevalence of blindness
Age
5
10
15
20
25
0
20
40
60
80

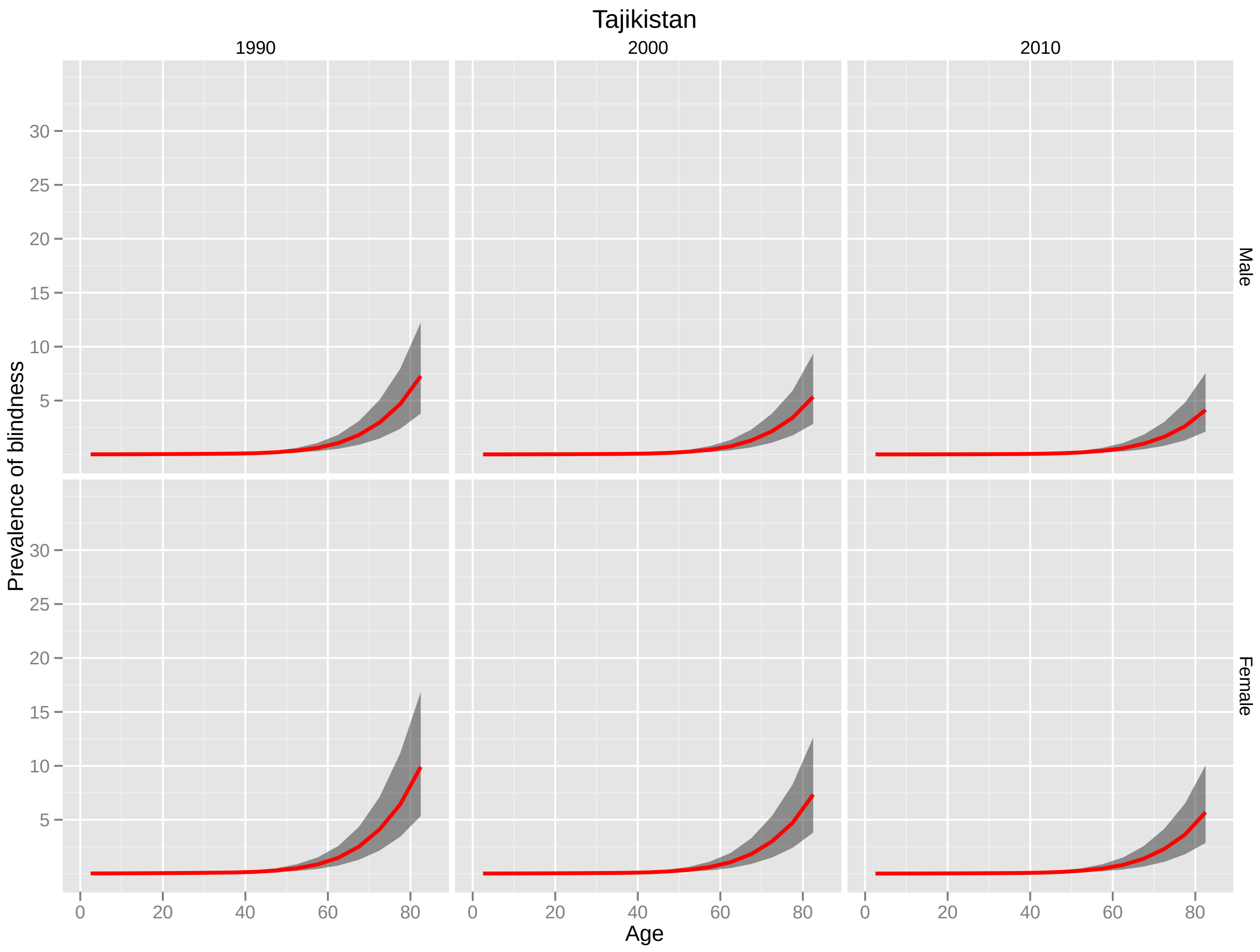
Tajikistan
1990
2000
2010
Male
Female
Prevalence of blindness
Age
30
25
20
15
10
5
0
20
40
60
80

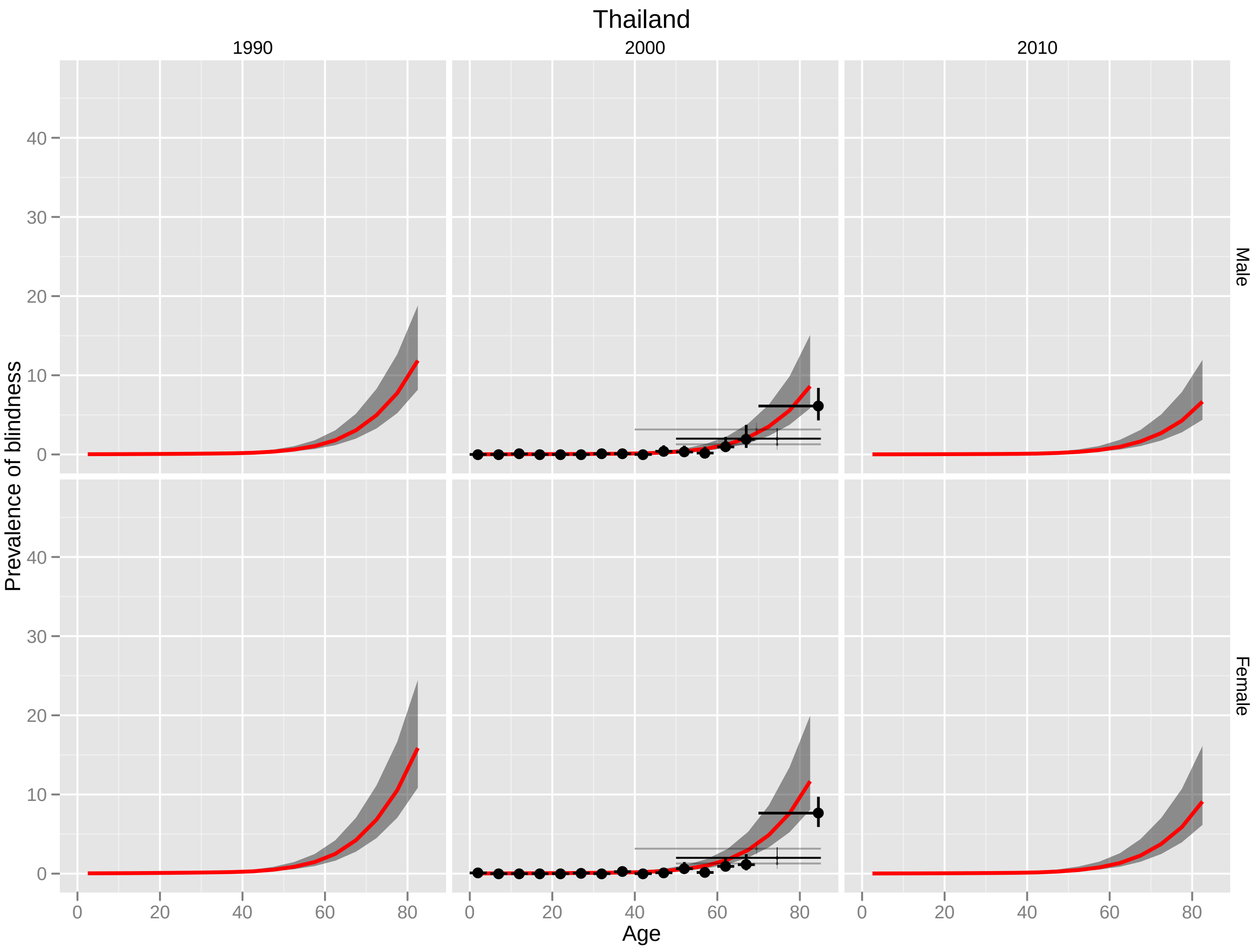

Thailand
1990
2000
2010
Male
Female
Prevalence of blindness
Age
0
10
20
30
40
0
20
40
60
80

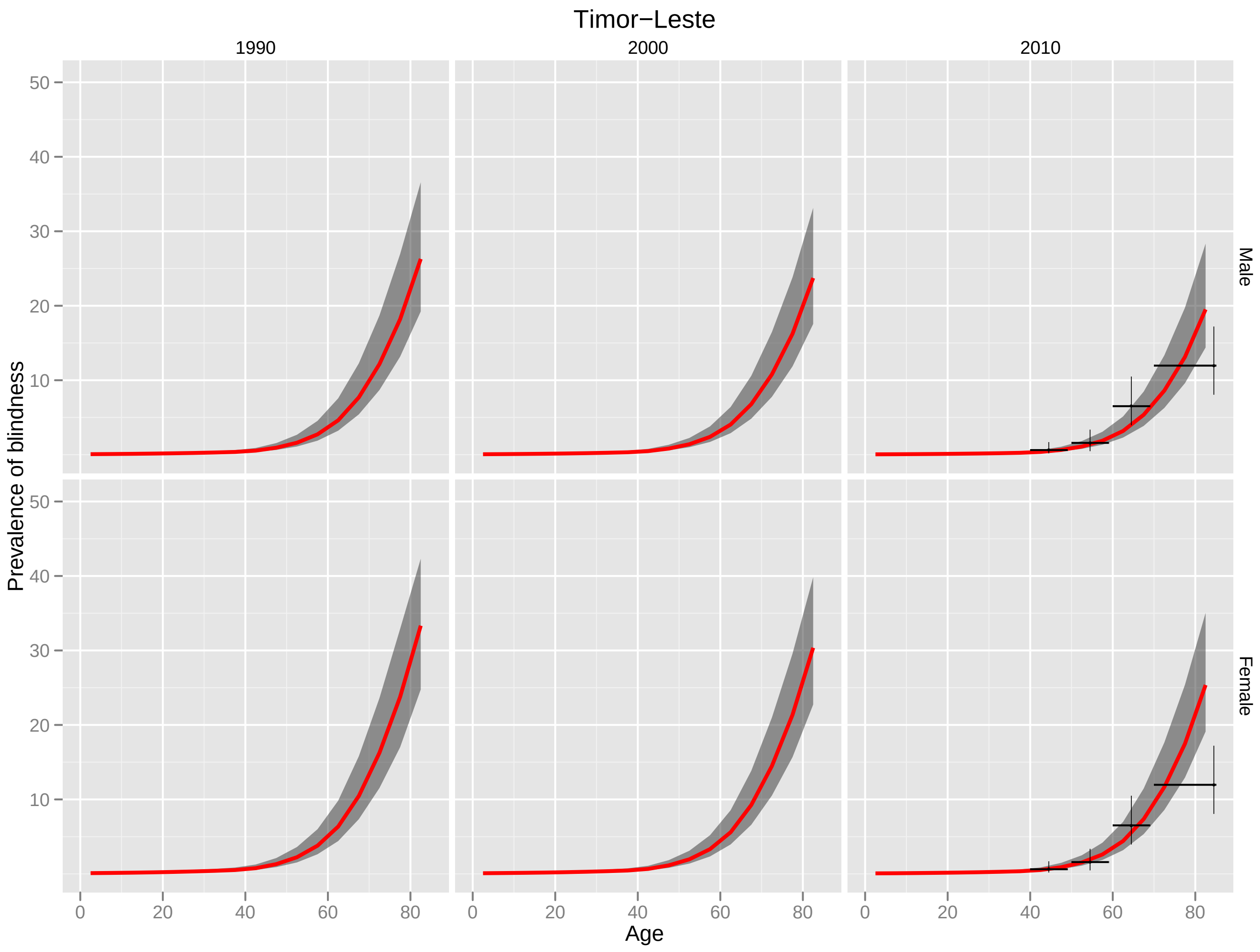

Timor−Leste
1990
2000
2010
Male
Female
Prevalence of blindness
Age
0
20
40
60
80
10
20
30
40
50

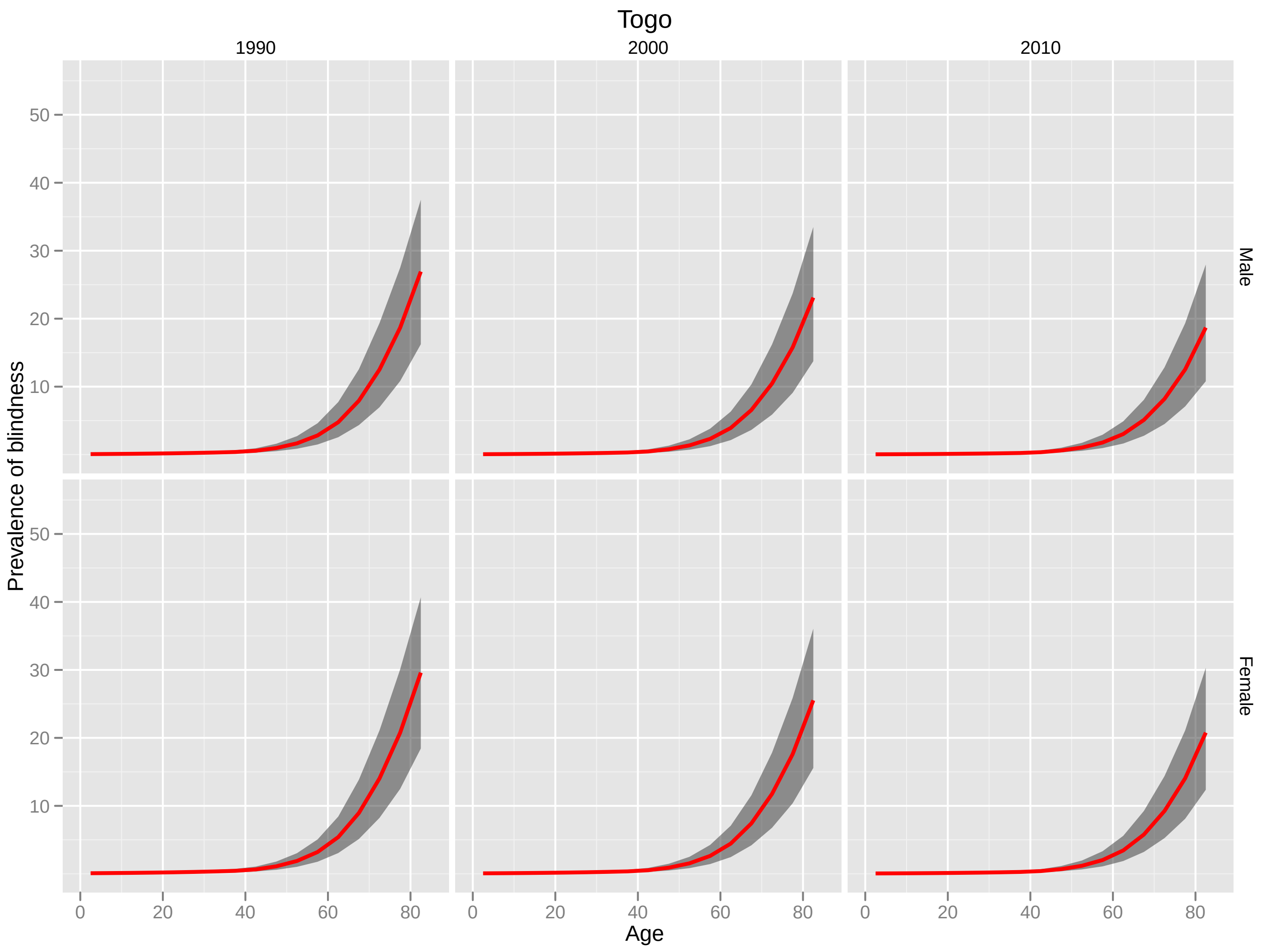
Togo
1990
2000
2010
Male
Female
Prevalence of blindness
Age
0
20
40
60
80
10
20
30
40
50

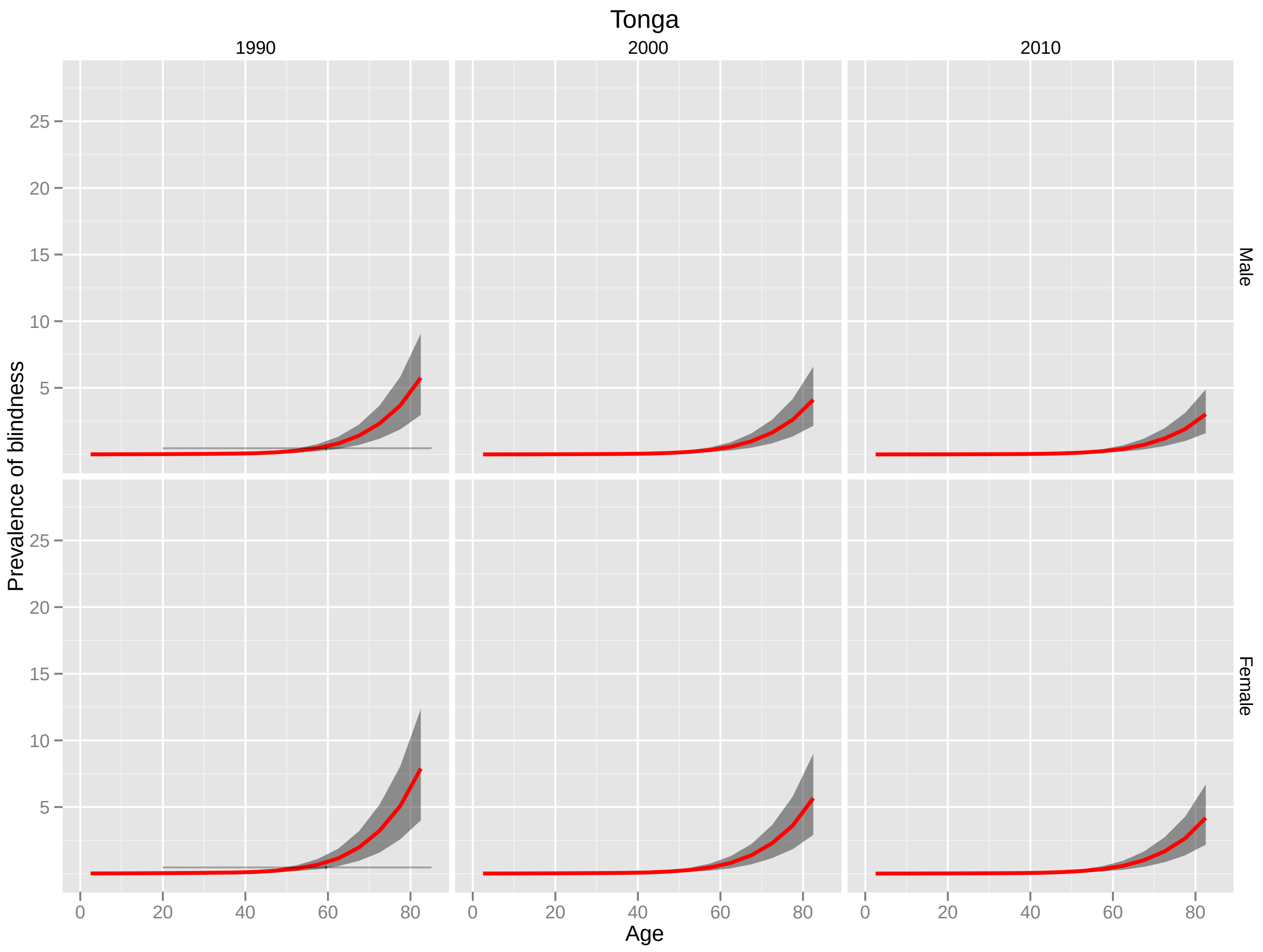
Tonga
1990
2000
2010
Male
Female
Prevalence of blindness
Age
0
20
40
60
80
5
10
15
20
25

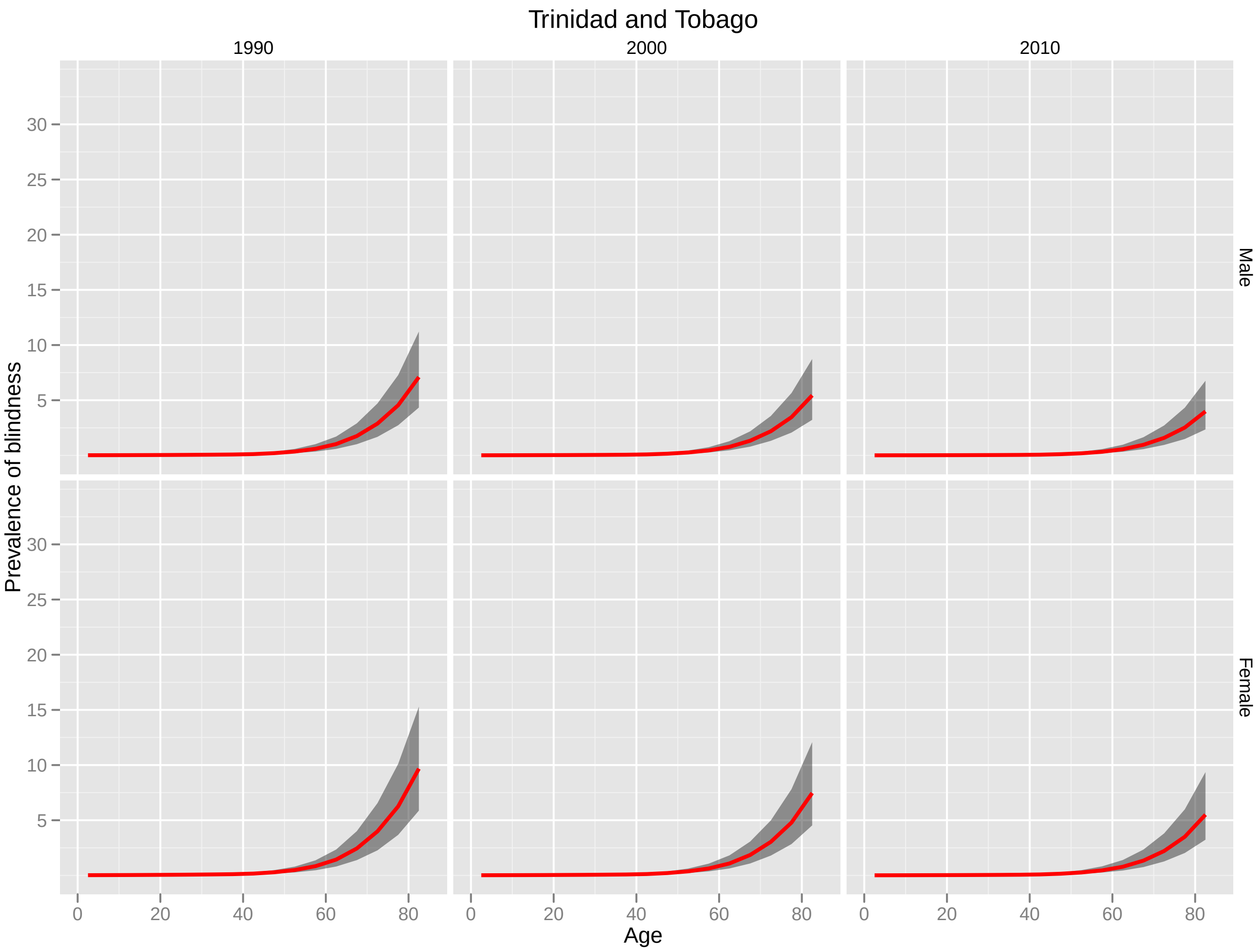
Trinidad and Tobago
1990
2000
2010
Male
Female
Prevalence of blindness
Age
30
25
20
15
10
5
0
20
40
60
80

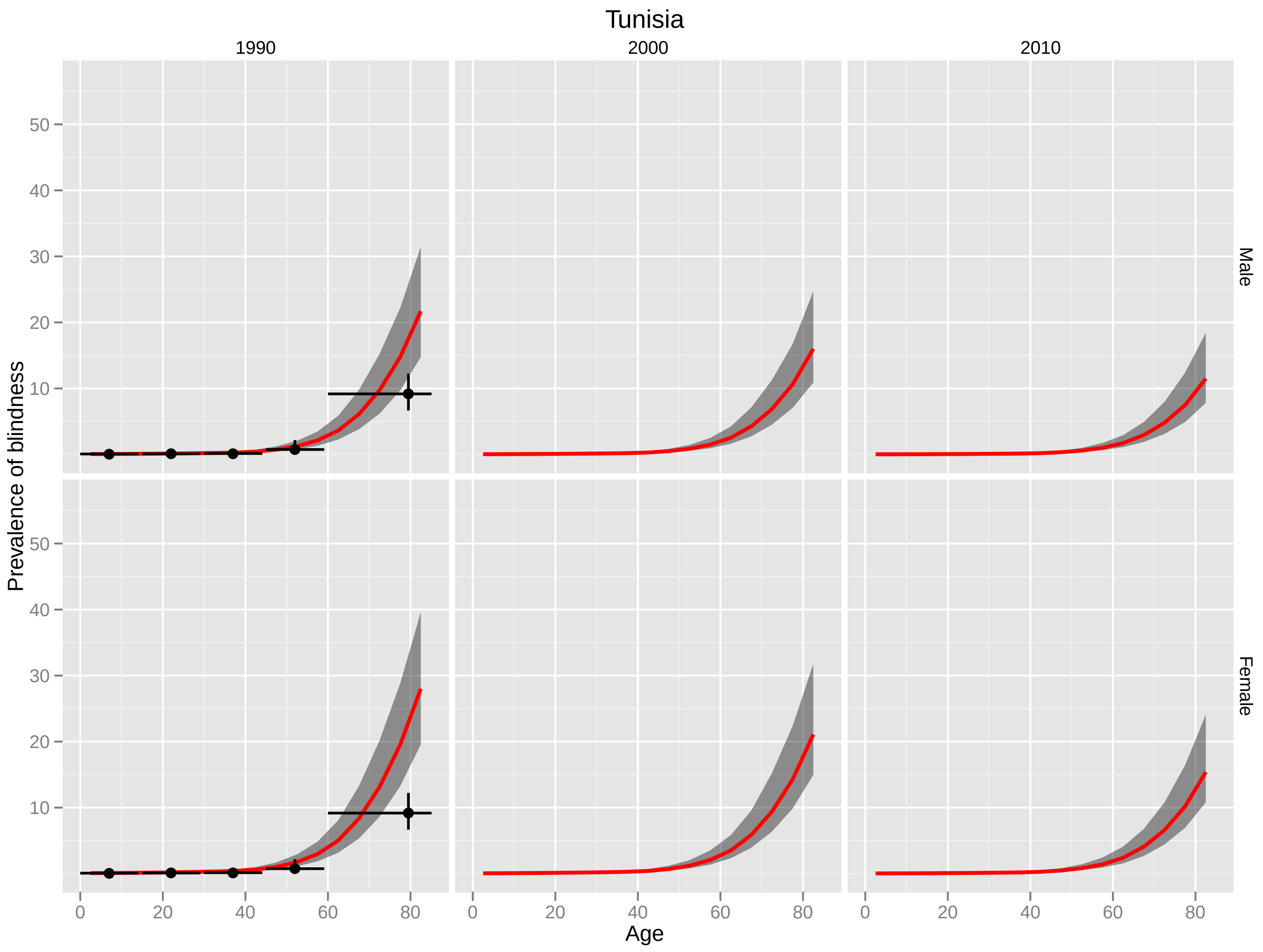
Tunisia
1990
2000
2010
Male
Female
Prevalence of blindness
10
20
30
40
50
0
20
40
60
80
Age

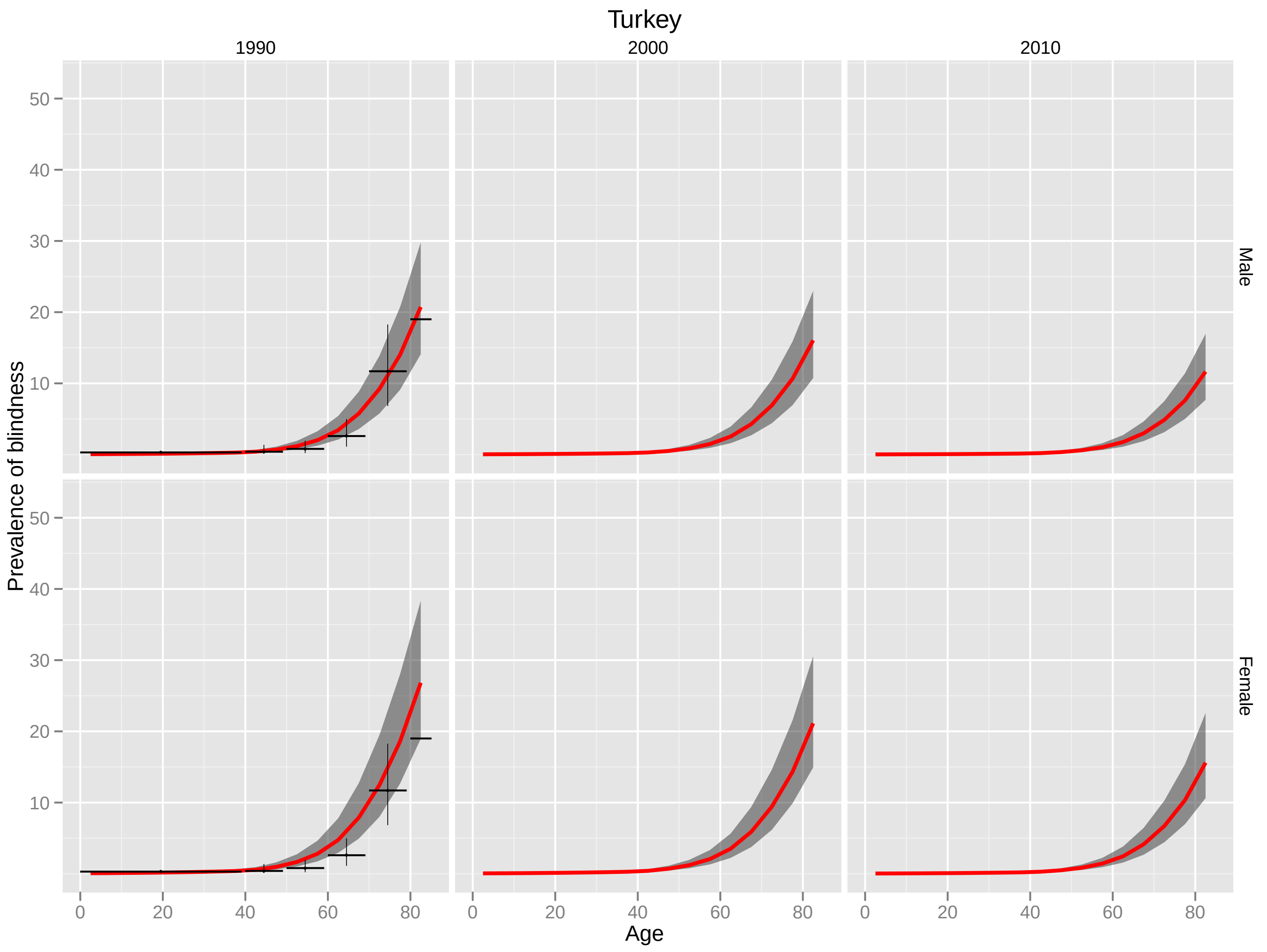
Turkey
1990
2000
2010
Male
Female
Prevalence of blindness
Age
10
20
30
40
50
0
20
40
60
80

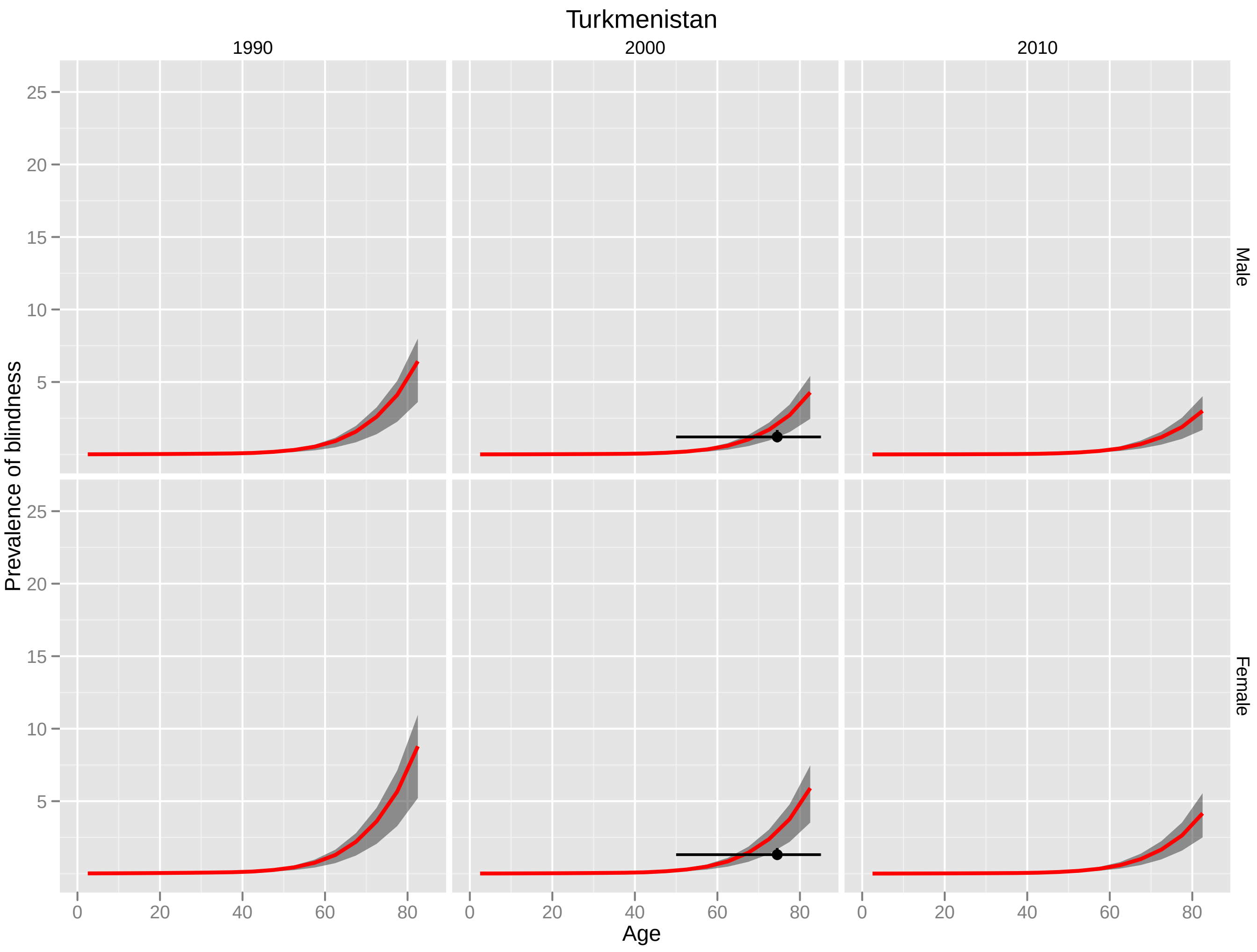
Turkmenistan
1990
2000
2010
Male
Female
Prevalence of blindness
Age
25
20
15
10
5
0
20
40
60
80

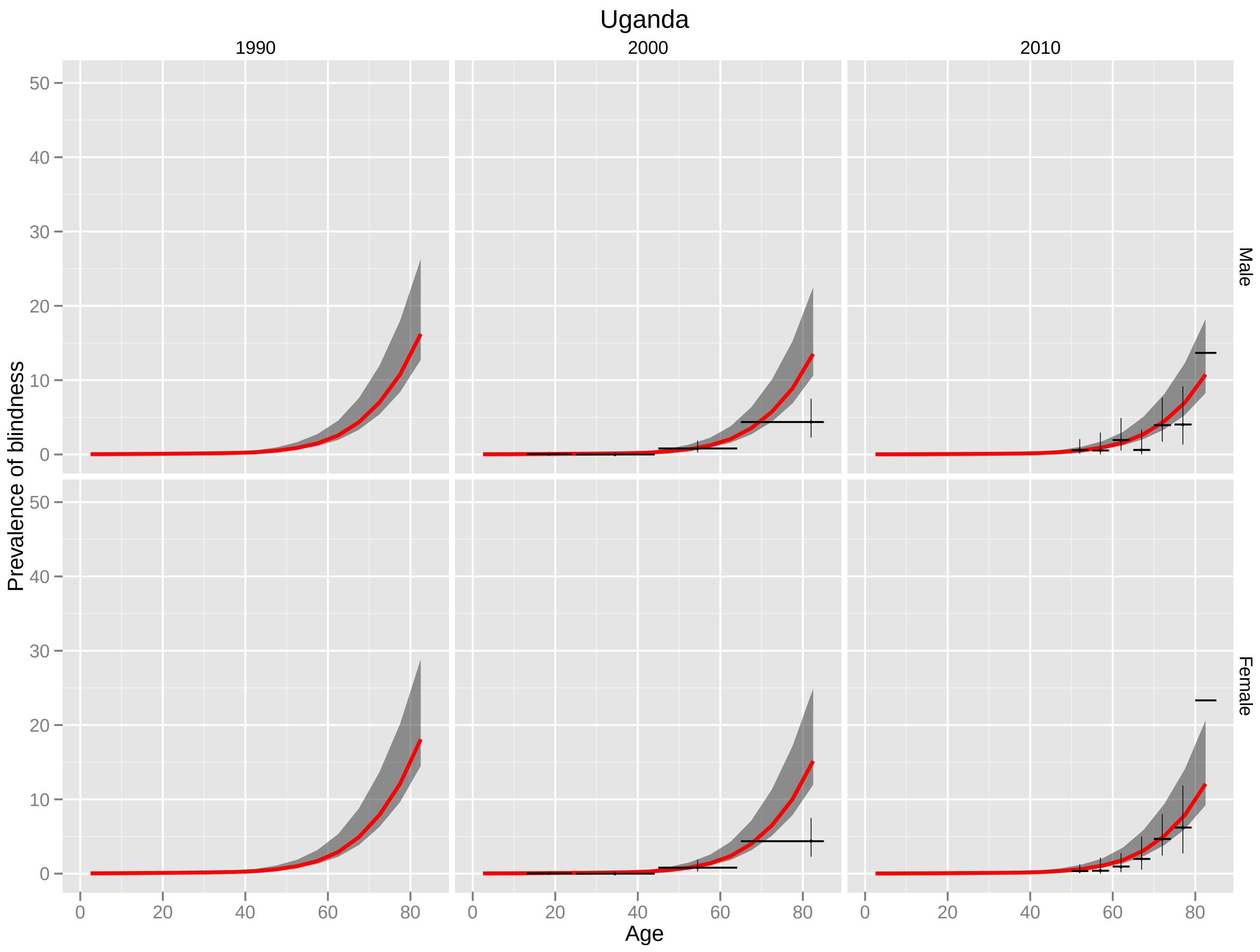
Uganda
1990
2000
2010
Male
Female
Prevalence of blindness
Age
0
10
20
30
40
50
0
20
40
60
80

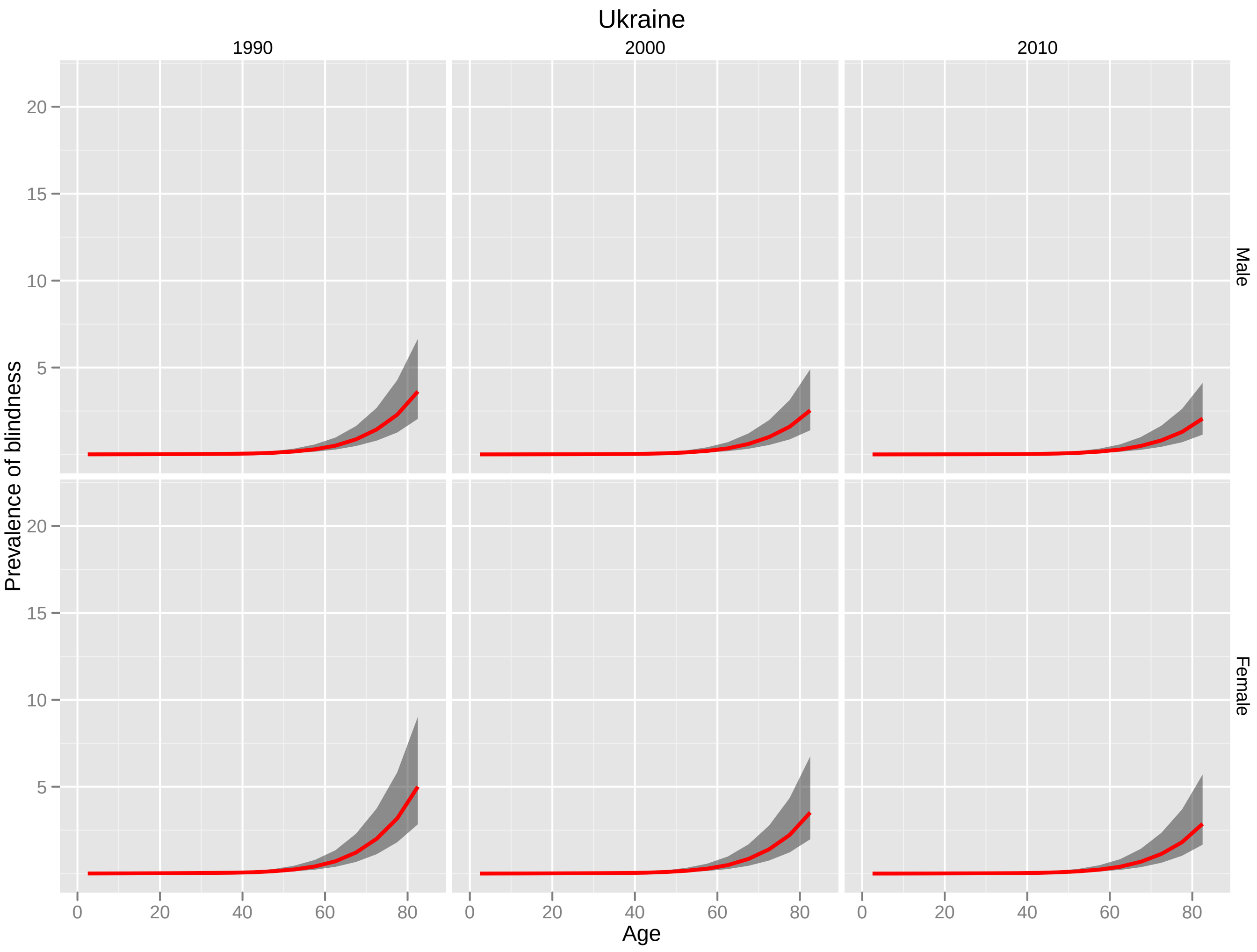
Ukraine
1990
2000
2010
Male
Female
Prevalence of blindness
Age
20
15
10
5
0
20
40
60
80

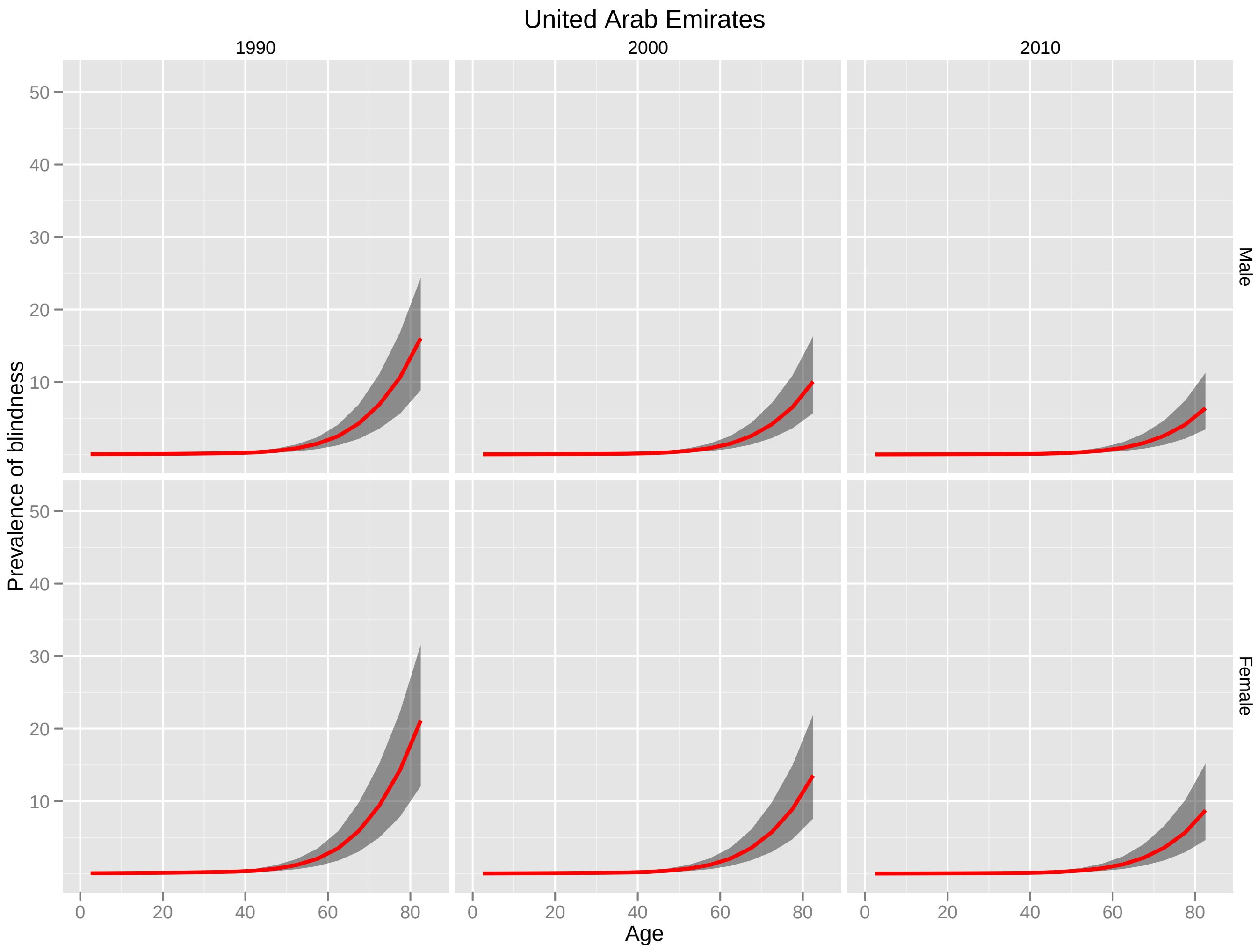

United Arab Emirates
1990
2000
2010
Male
Female
Prevalence of blindness
Age
50
40
30
20
10
0
20
40
60
80

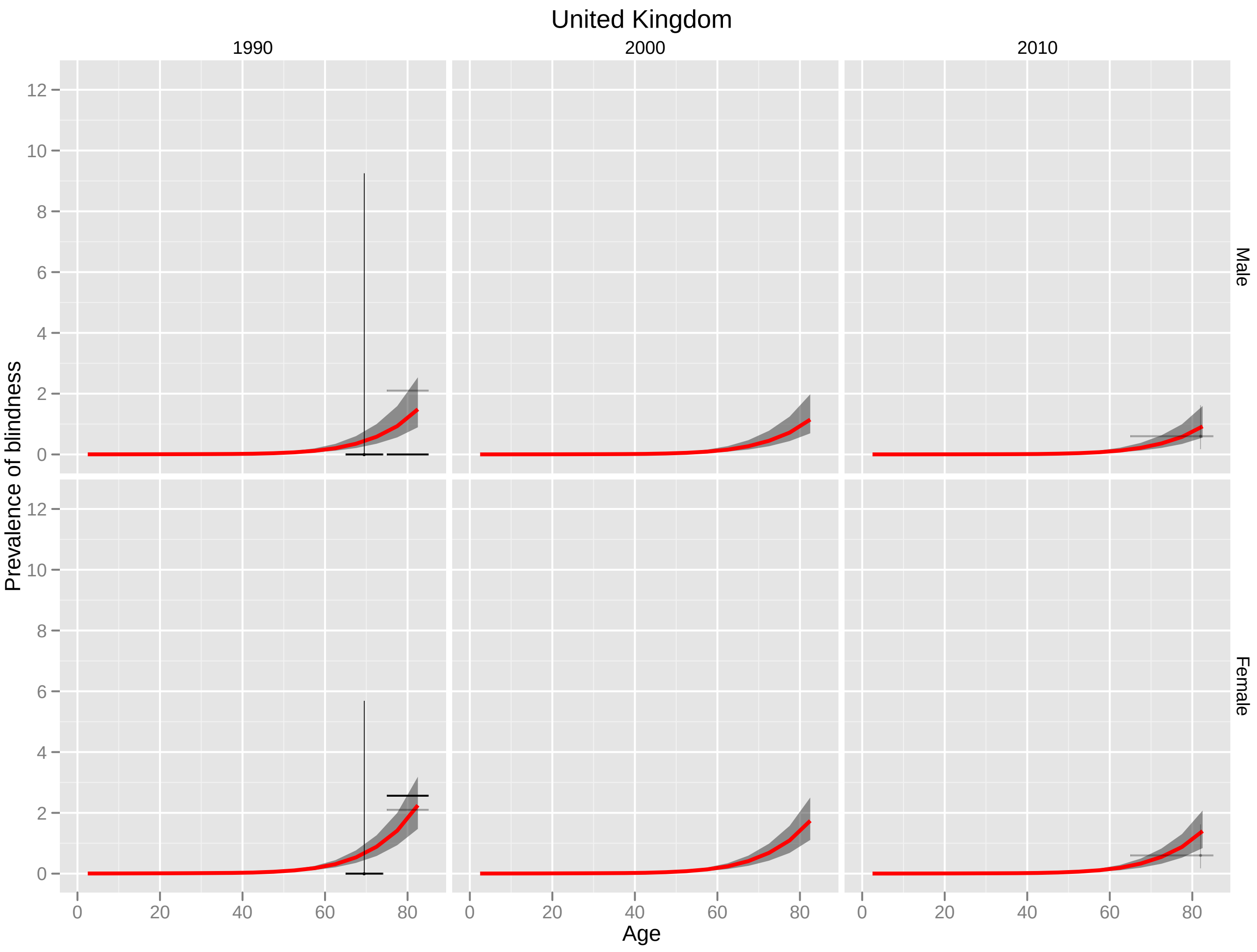
United Kingdom
1990
2000
2010
Male
Female
Prevalence of blindness
Age
0
2
4
6
8
10
12
0
20
40
60
80

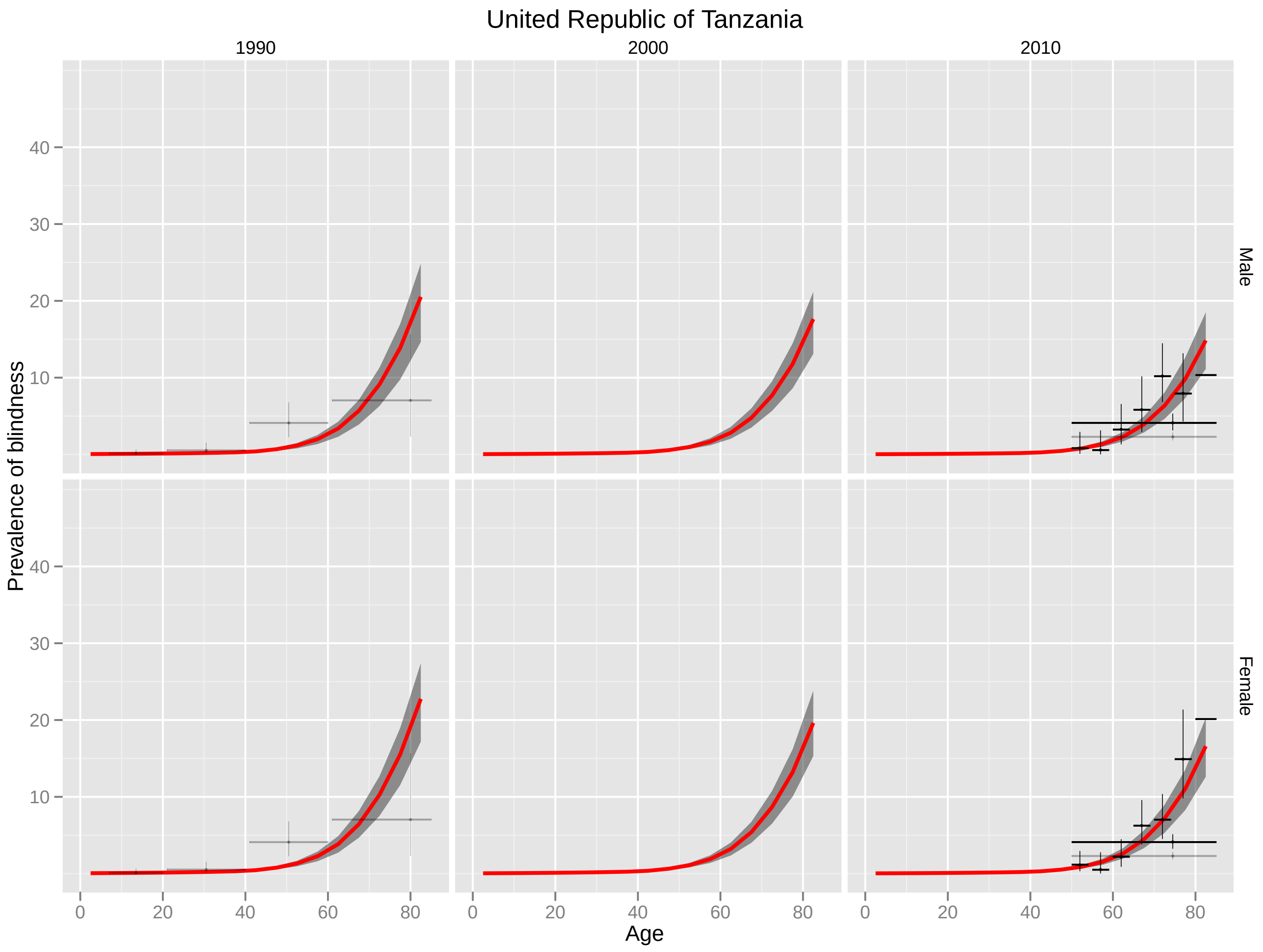

United Republic of Tanzania
1990
2000
2010
Male
Female
Prevalence of blindness
Age
10
20
30
40
0
20
40
60
80

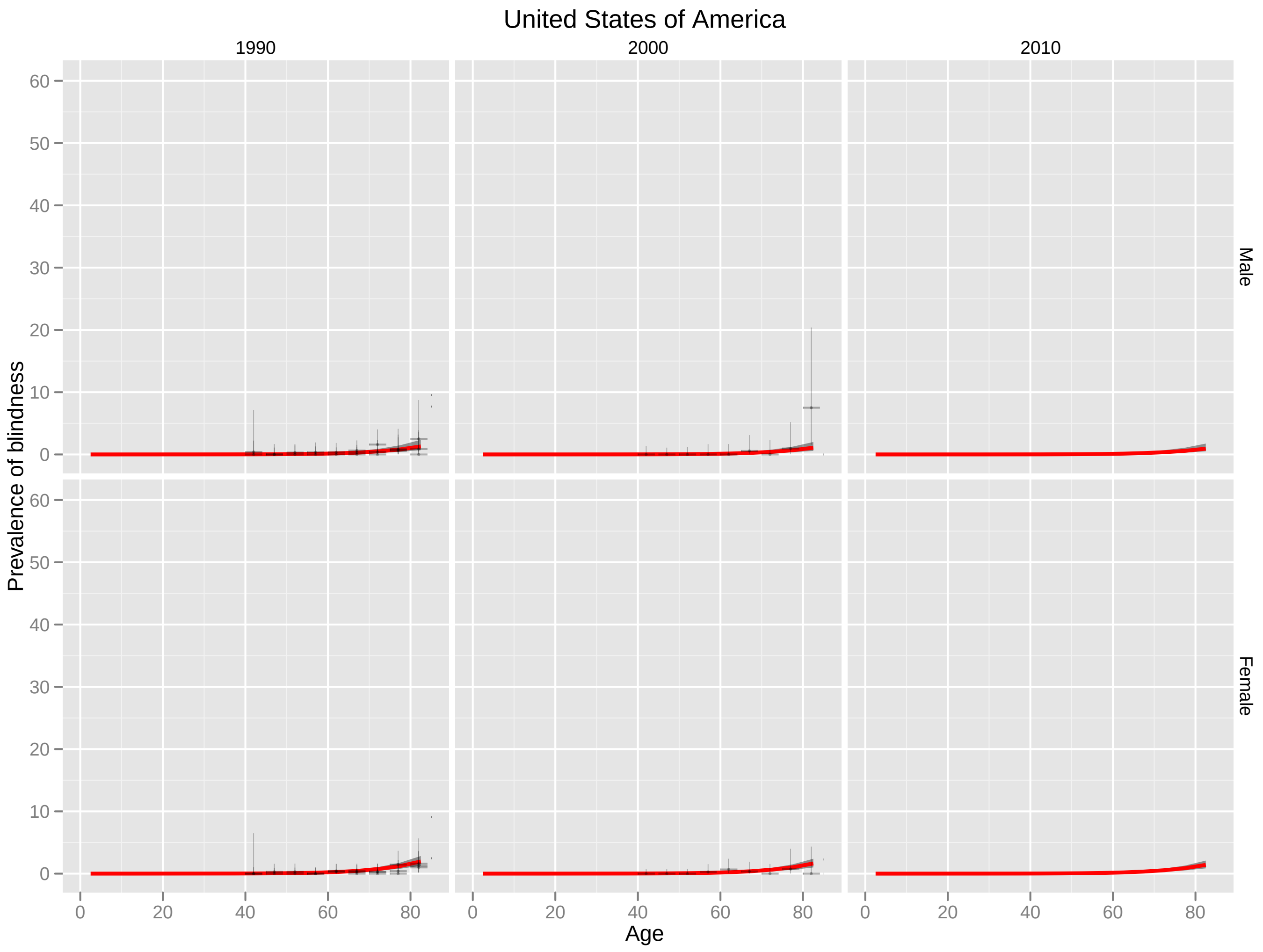

United States of America
1990
2000
2010
Male
Female
Prevalence of blindness
Age
0
10
20
30
40
50
60
80

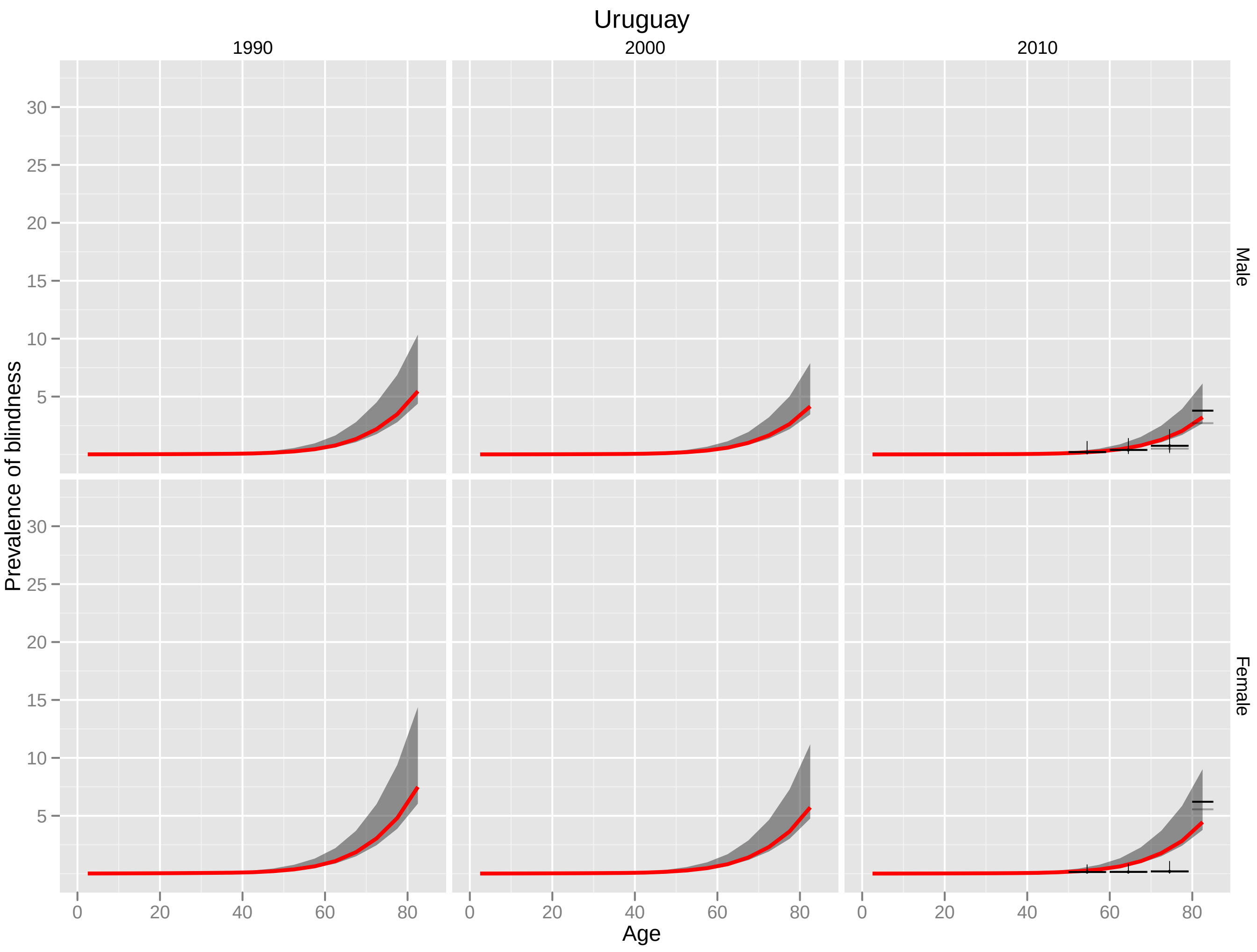
Uruguay
1990
2000
2010
Male
Female
Prevalence of blindness
Age
5
10
15
20
25
30
0
20
40
60
80

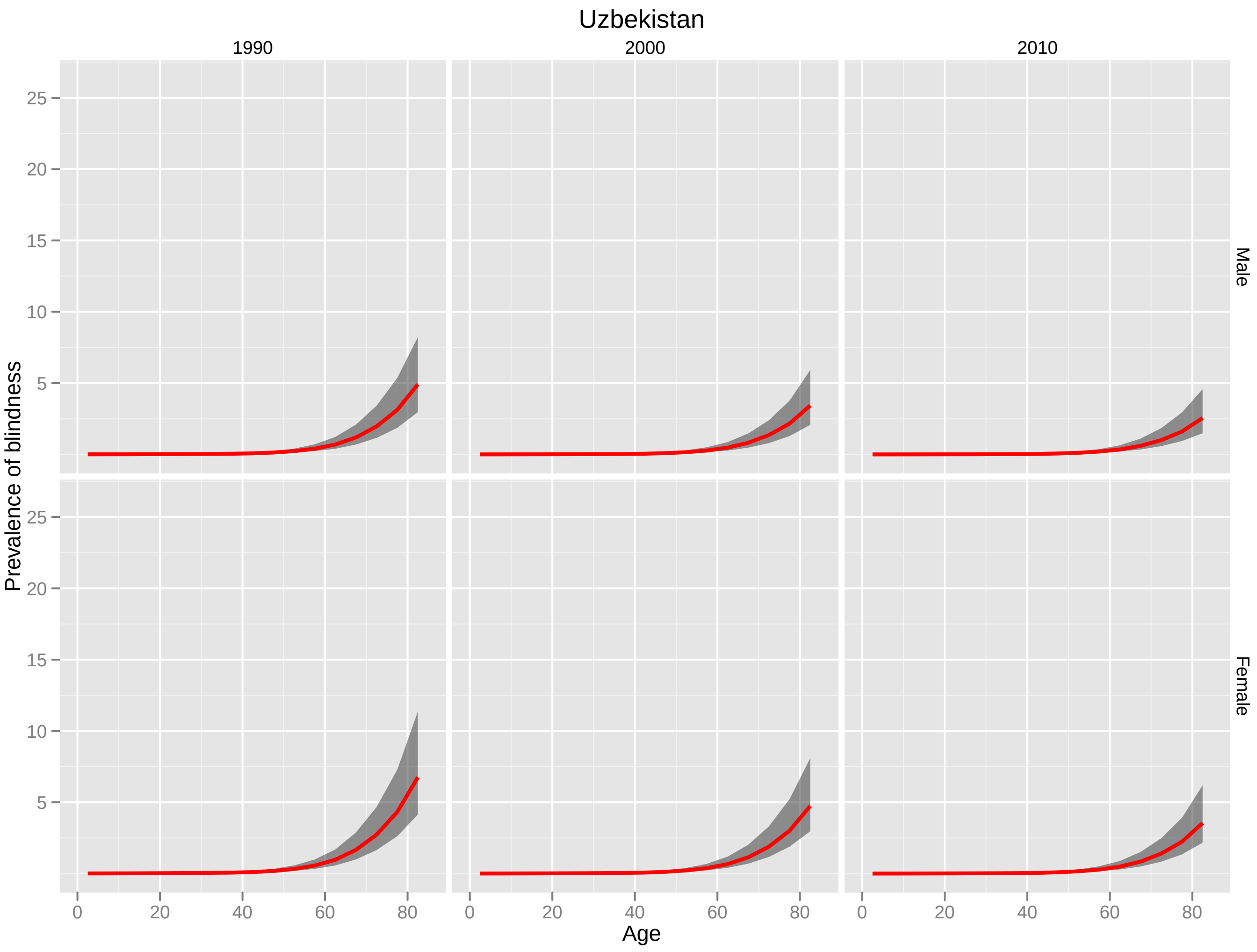
Uzbekistan
1990
2000
2010
Male
Female
Prevalence of blindness
25
20
15
10
5
0
20
40
60
80
Age

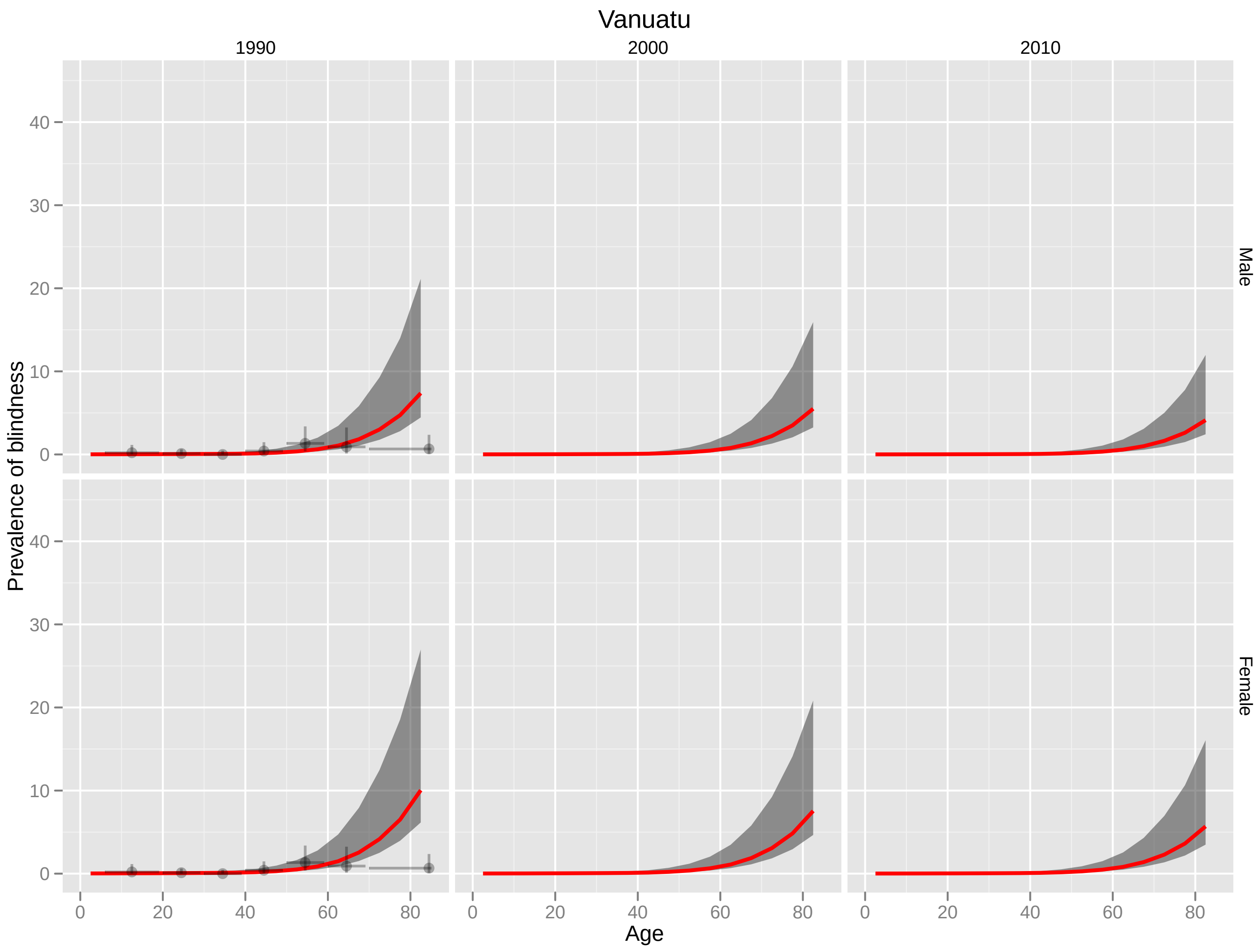
Vanuatu
1990
2000
2010
Male
Female
Prevalence of blindness
Age
0
10
20
30
40
0
20
40
60
80

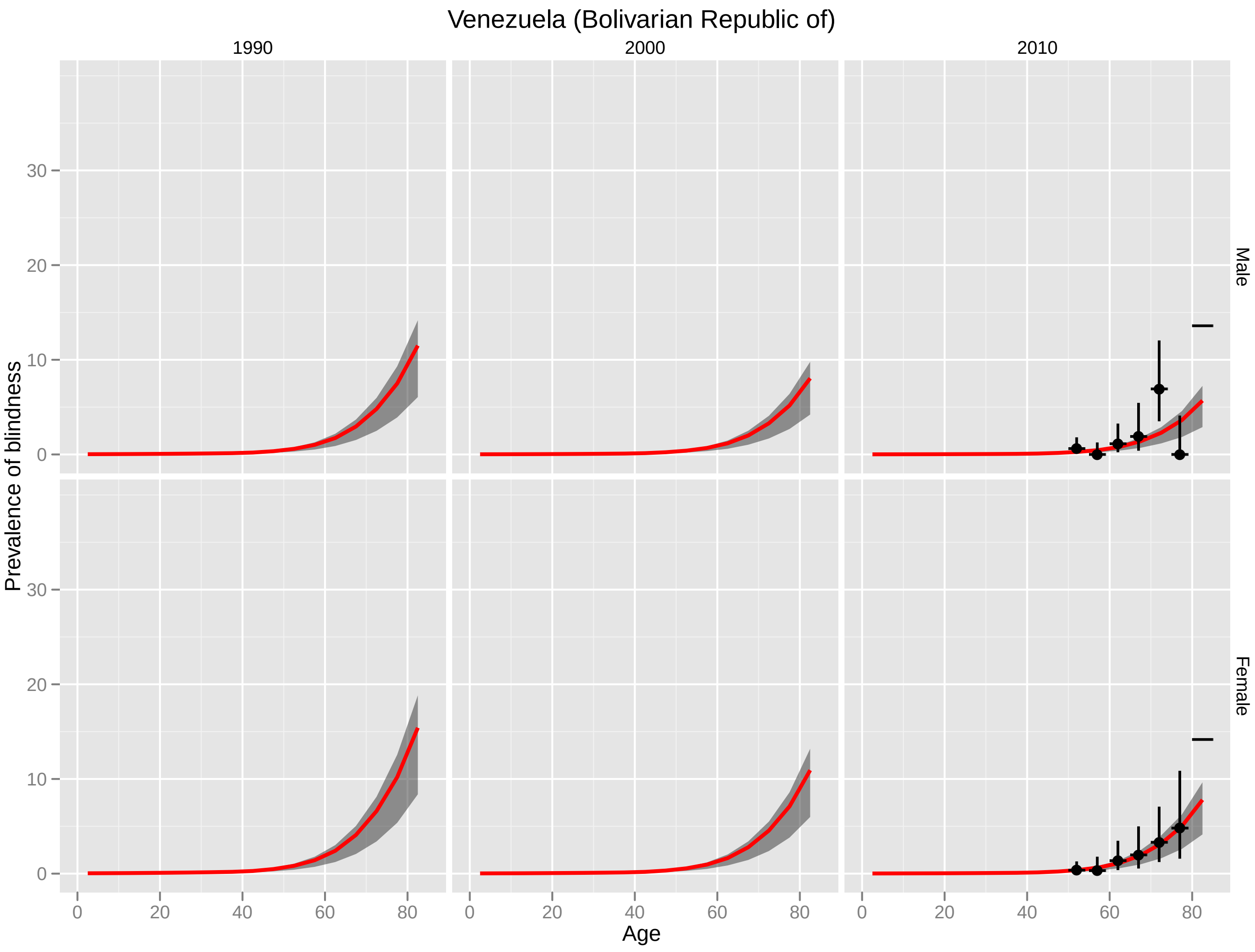
Venezuela (Bolivarian Republic of)
1990
2000
2010
Male
Female
Prevalence of blindness
0
10
20
30
Age
0
20
40
60
80

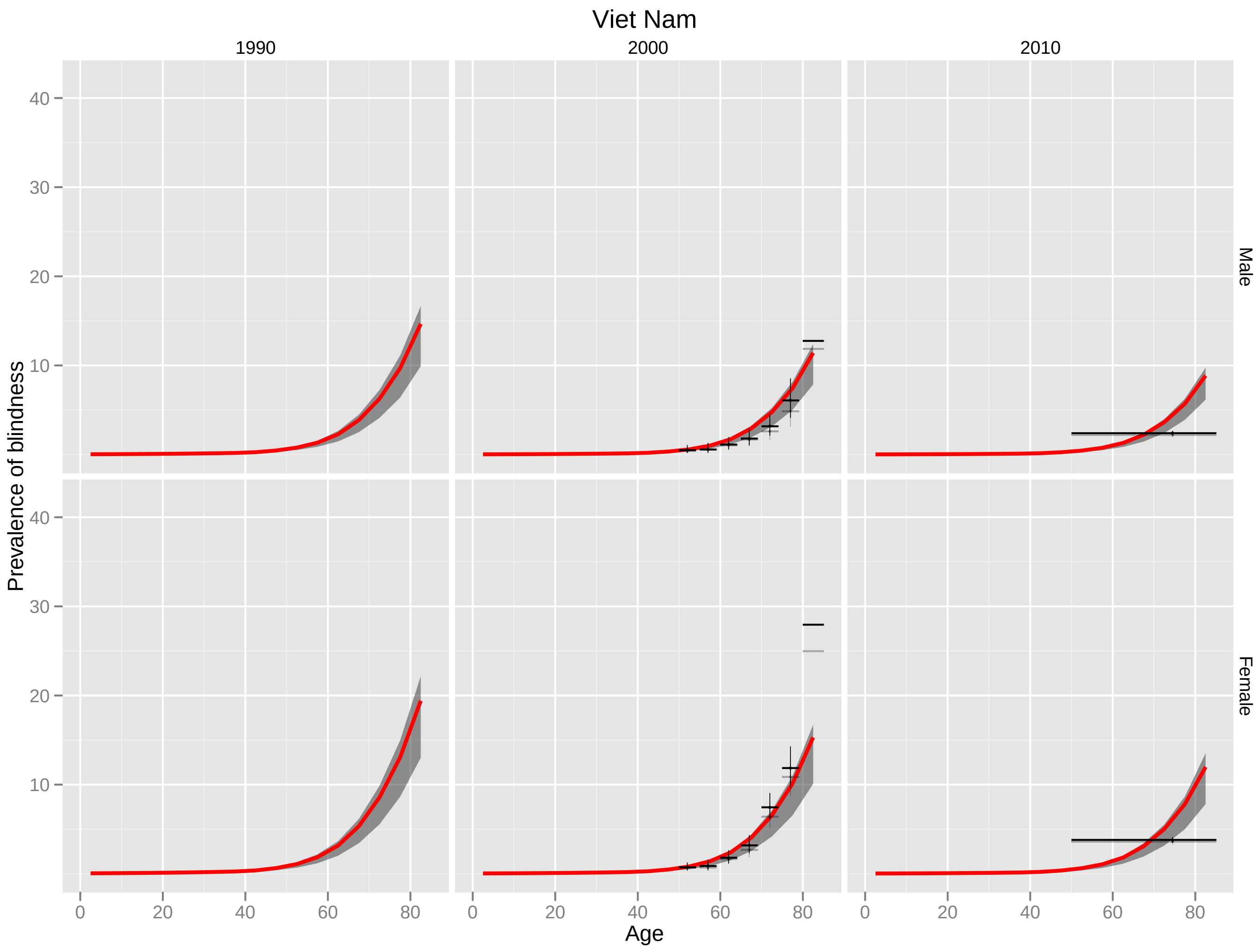
Viet Nam
1990
2000
2010
Male
Female
Prevalence of blindness
40
30
20
10
0
20
40
60
80
Age

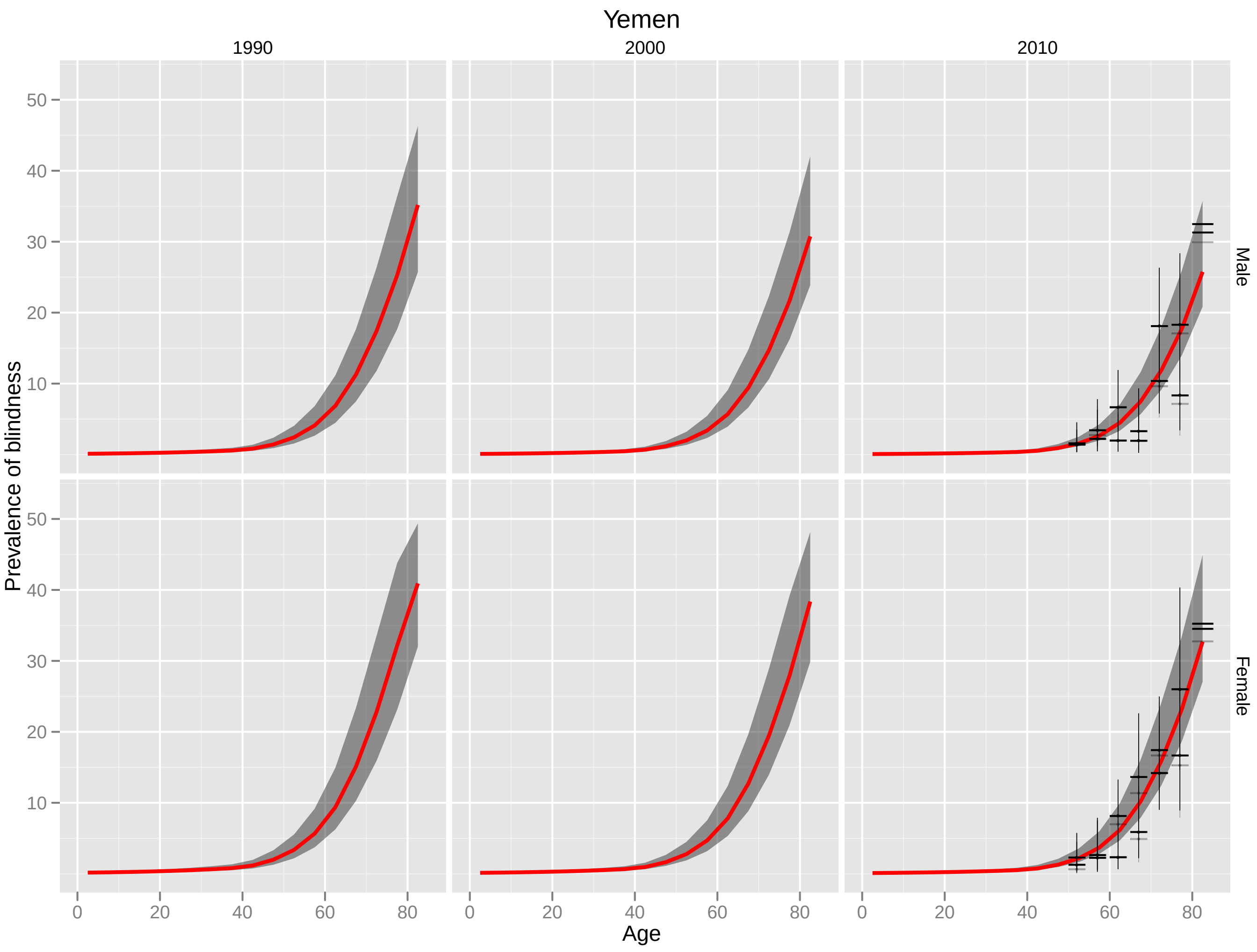
Yemen
1990
2000
2010
Male
Female
Prevalence of blindness
Age
50
40
30
20
10
0
20
40
60
80

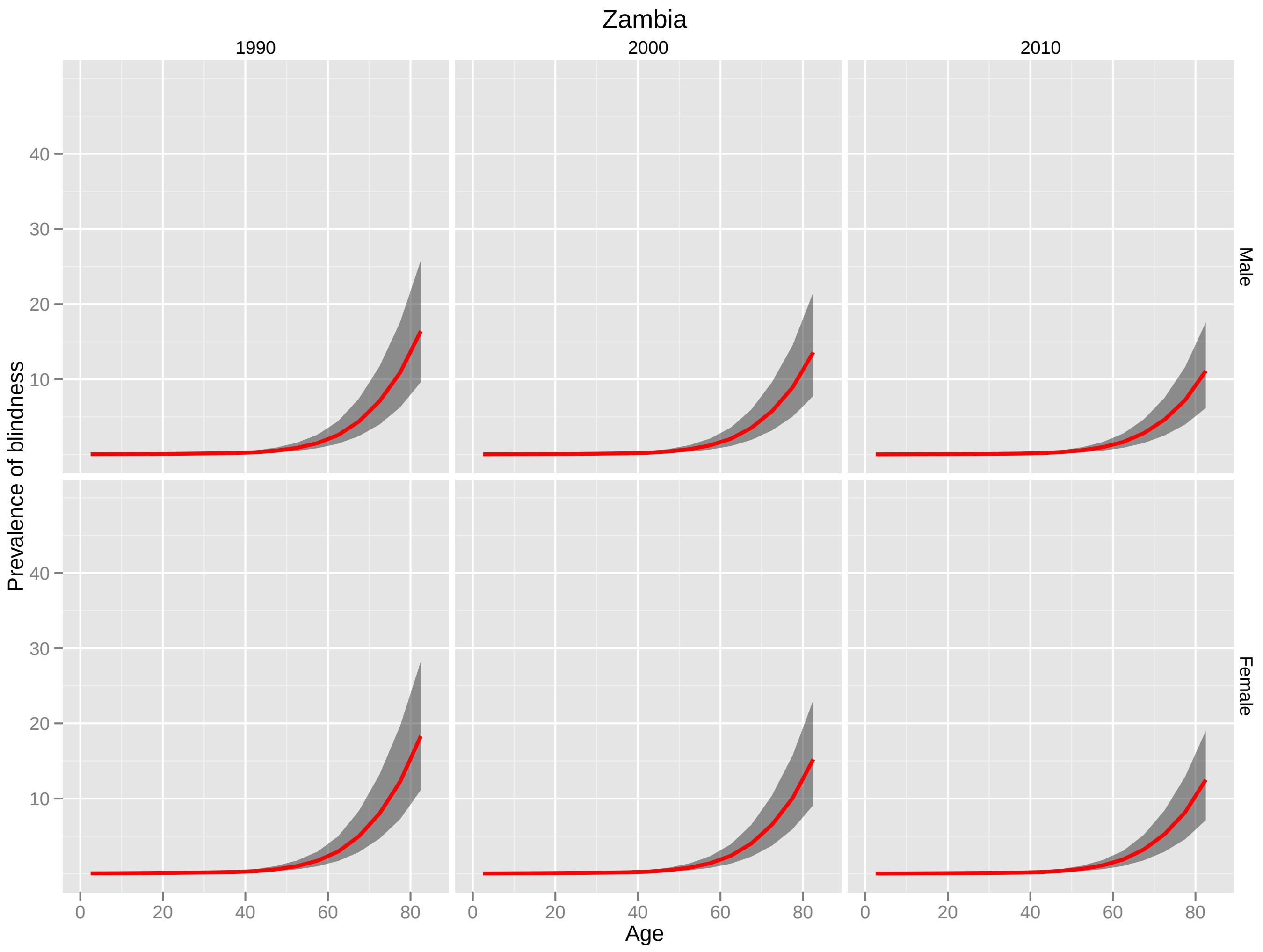

Zambia
1990
2000
2010
Male
Female
Prevalence of blindness
Age
10
20
30
40
0
20
40
60
80

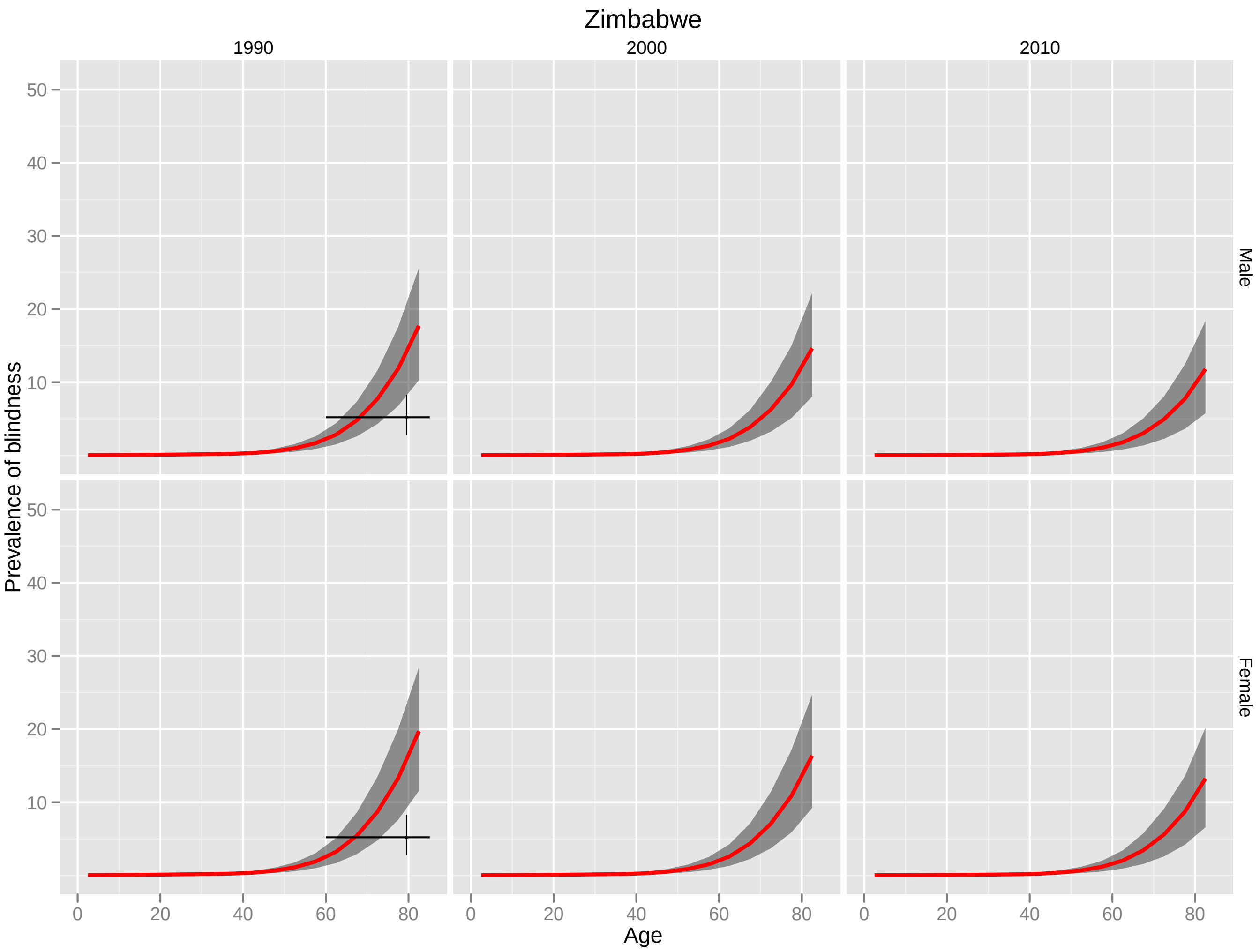
Zimbabwe
1990
2000
2010
Male
Female
Prevalence of blindness
Age
10
20
30
40
50
0
20
40
60
80

+ Presenting
+ Best corrected

National
Sub−national

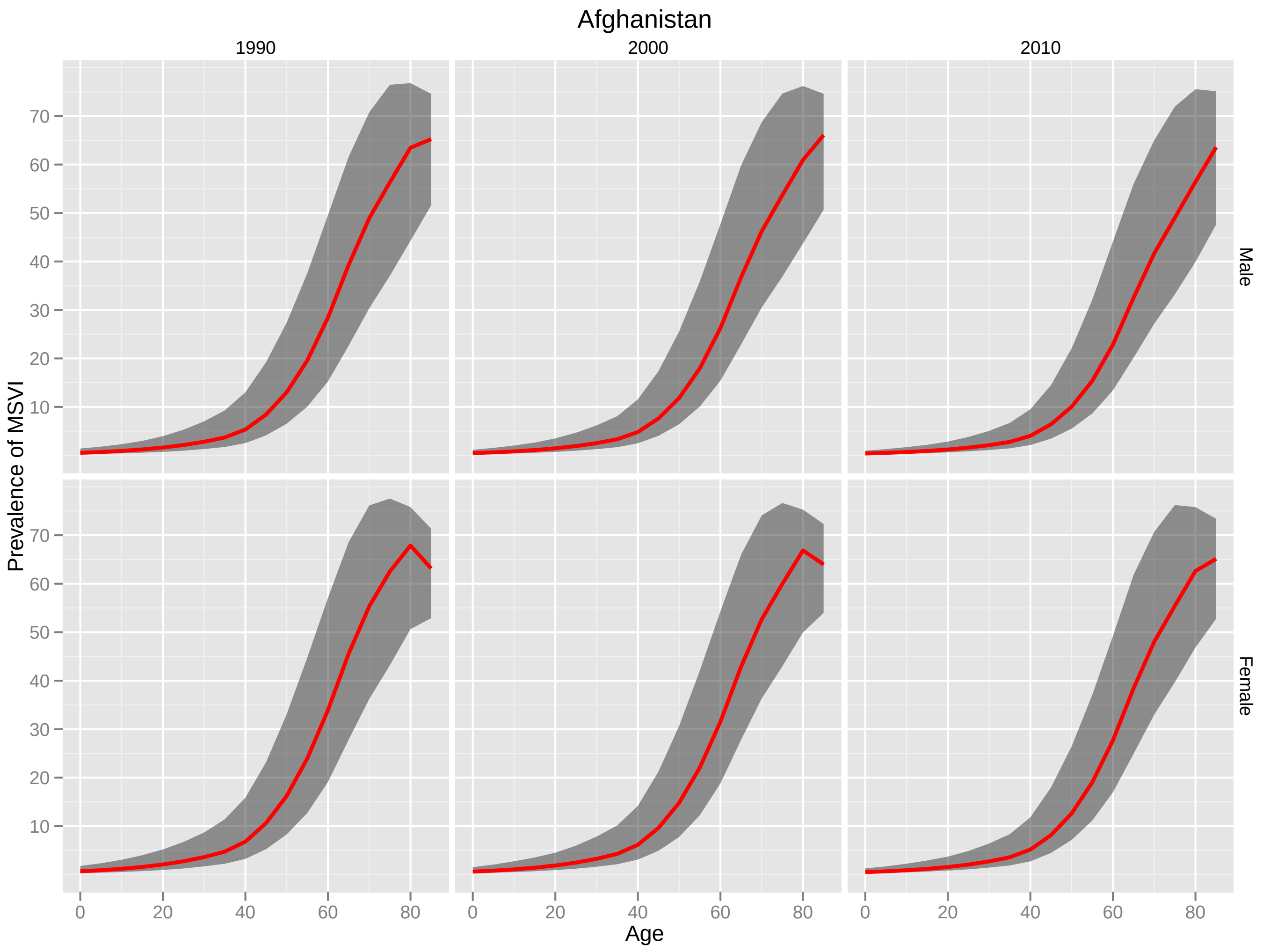

Afghanistan
1990
2000
2010
Male
Female
Prevalence of MSVI
Age
10
20
30
40
50
60
70
0
20
40
60
80

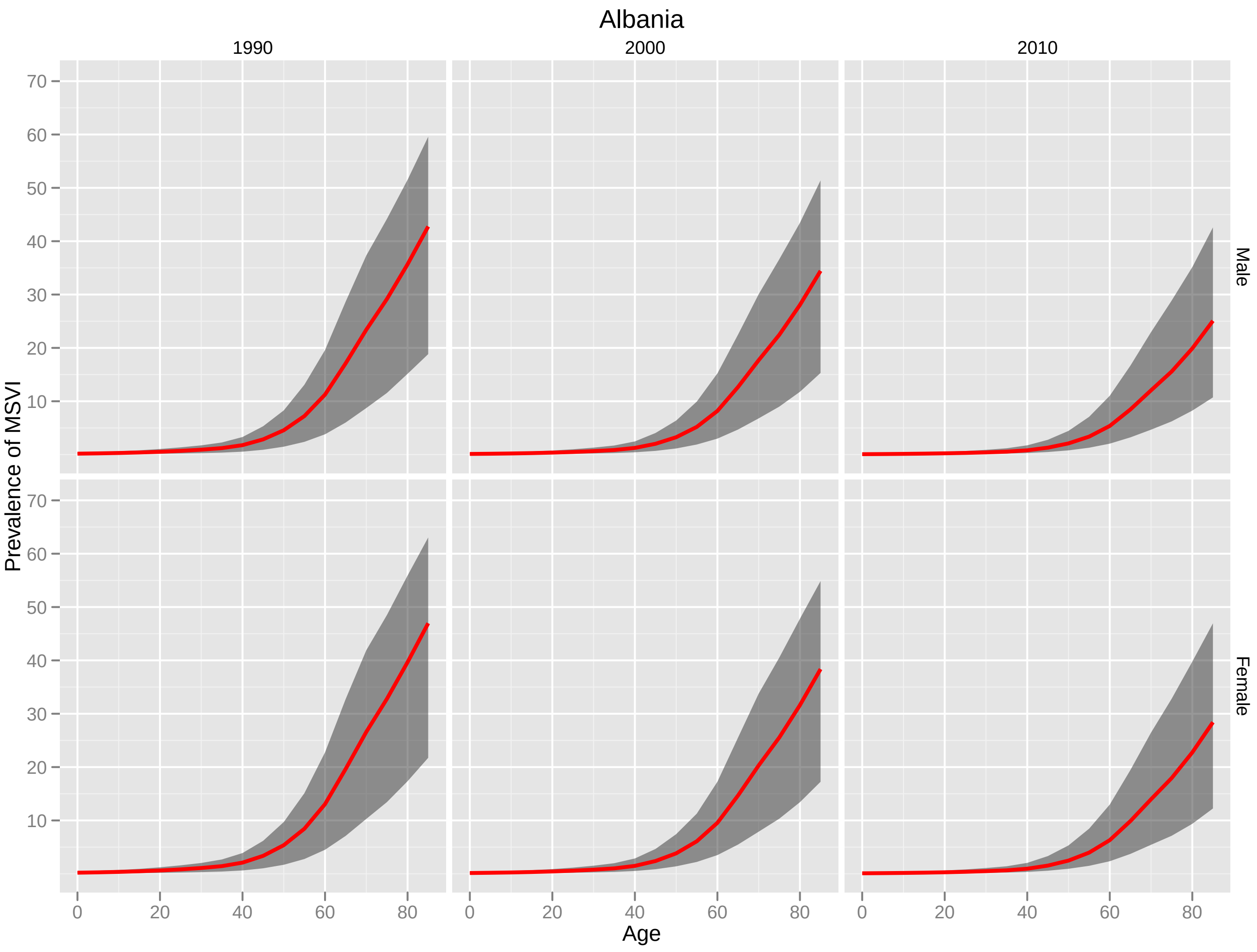

Albania
1990
2000
2010
Male
Female
Prevalence of MSVI
Age
10
20
30
40
50
60
70
0
20
40
60
80

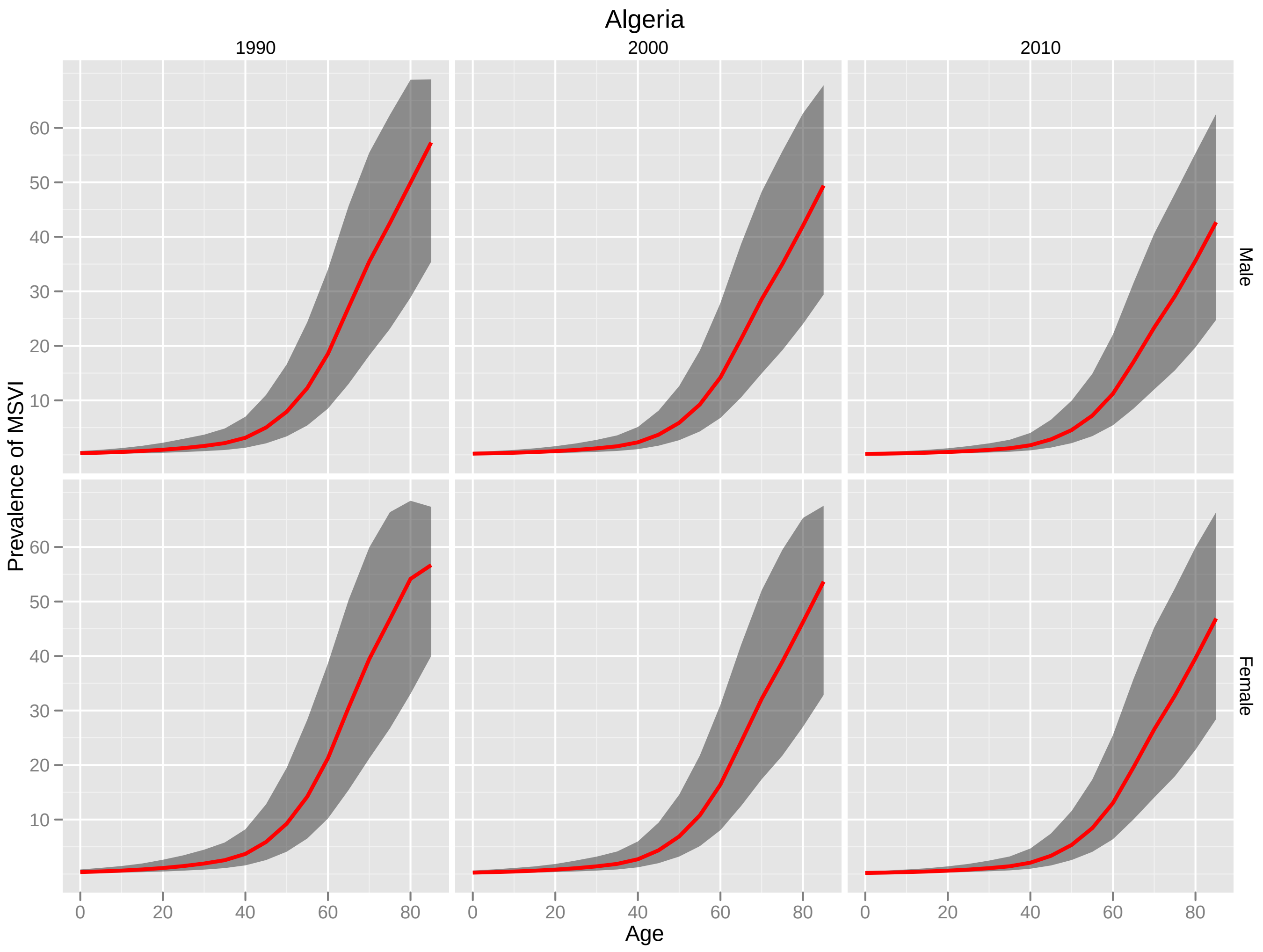
Algeria
1990
2000
2010
Male
Female
Prevalence of MSVI
Age
10
20
30
40
50
60
0
20
40
60
80

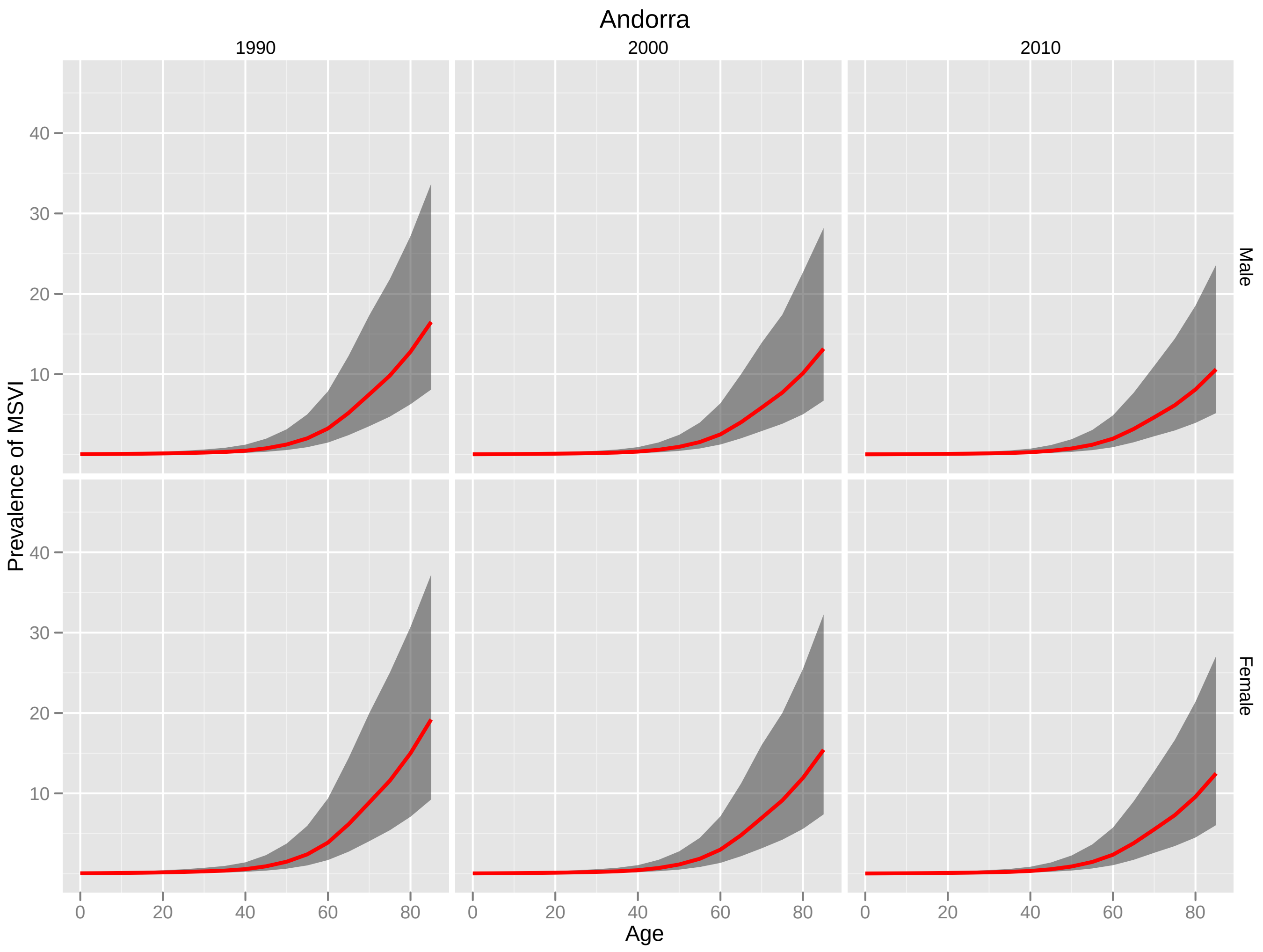
Andorra
1990
2000
2010
Male
Female
Prevalence of MSVI
Age
0
20
40
60
80
10
20
30
40

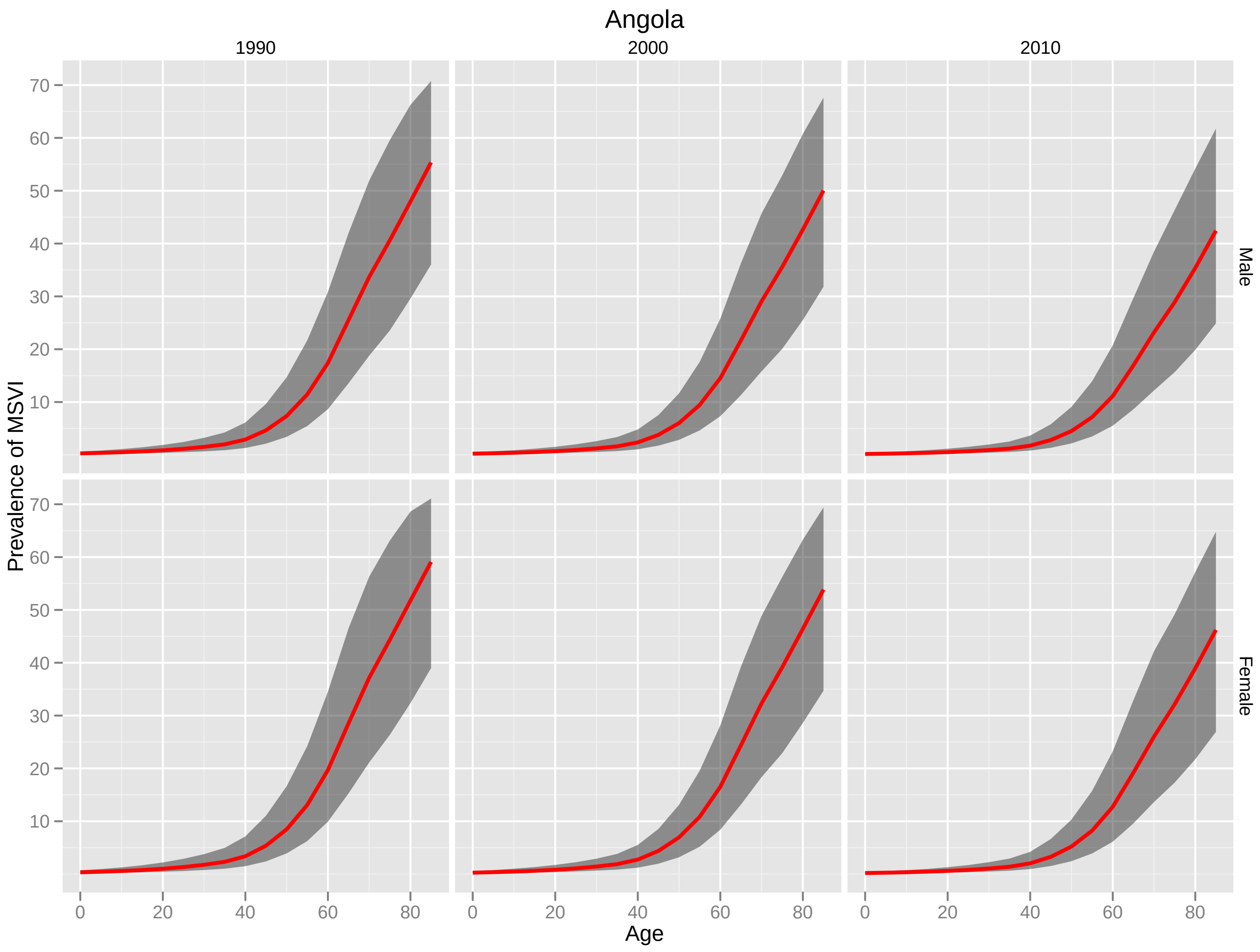
Angola
1990
2000
2010
Male
Female
Prevalence of MSVI
Age
0
20
40
60
80
10
20
30
40
50
60
70

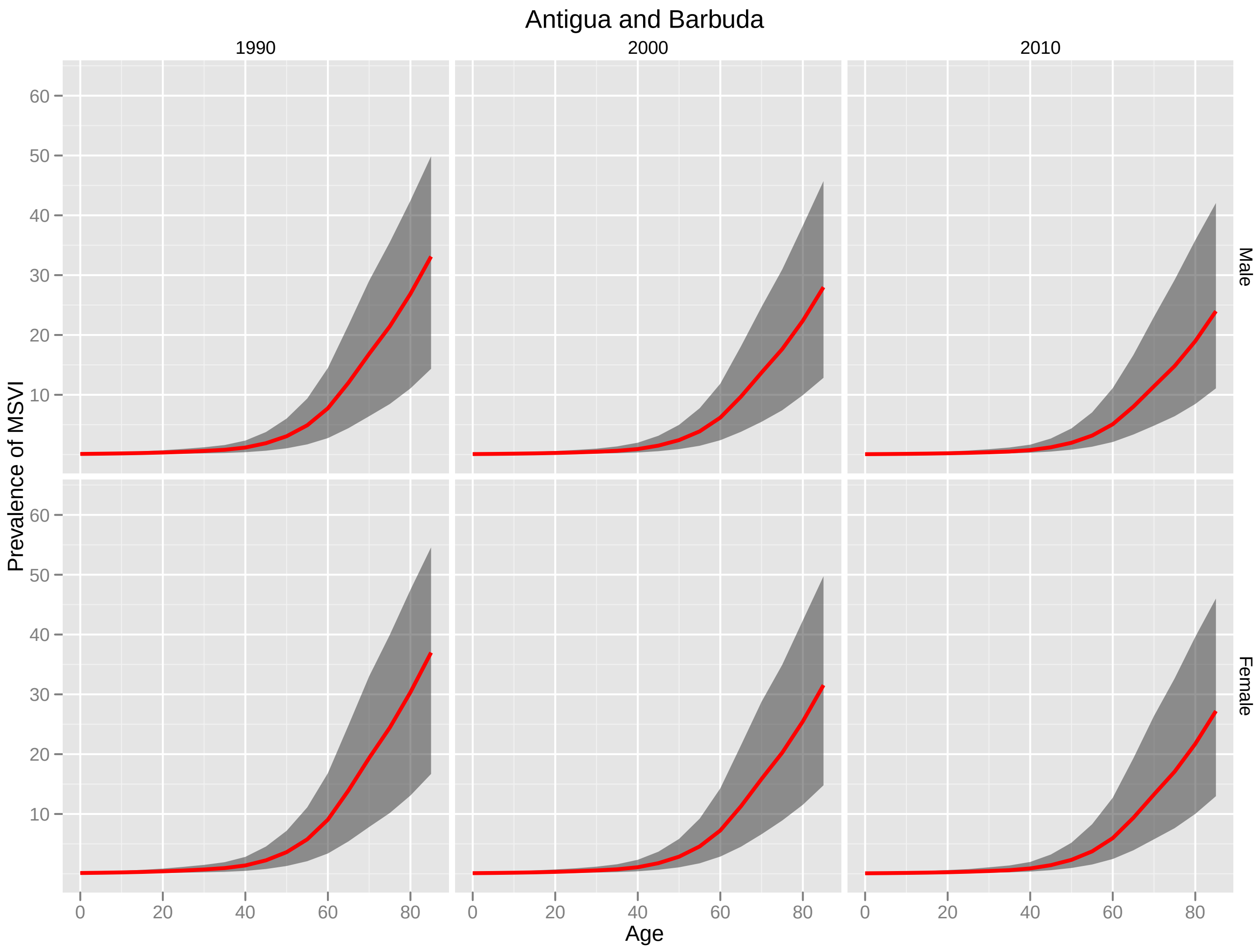

Antigua and Barbuda
1990
2000
2010
Male
Female
Prevalence of MSVI
Age

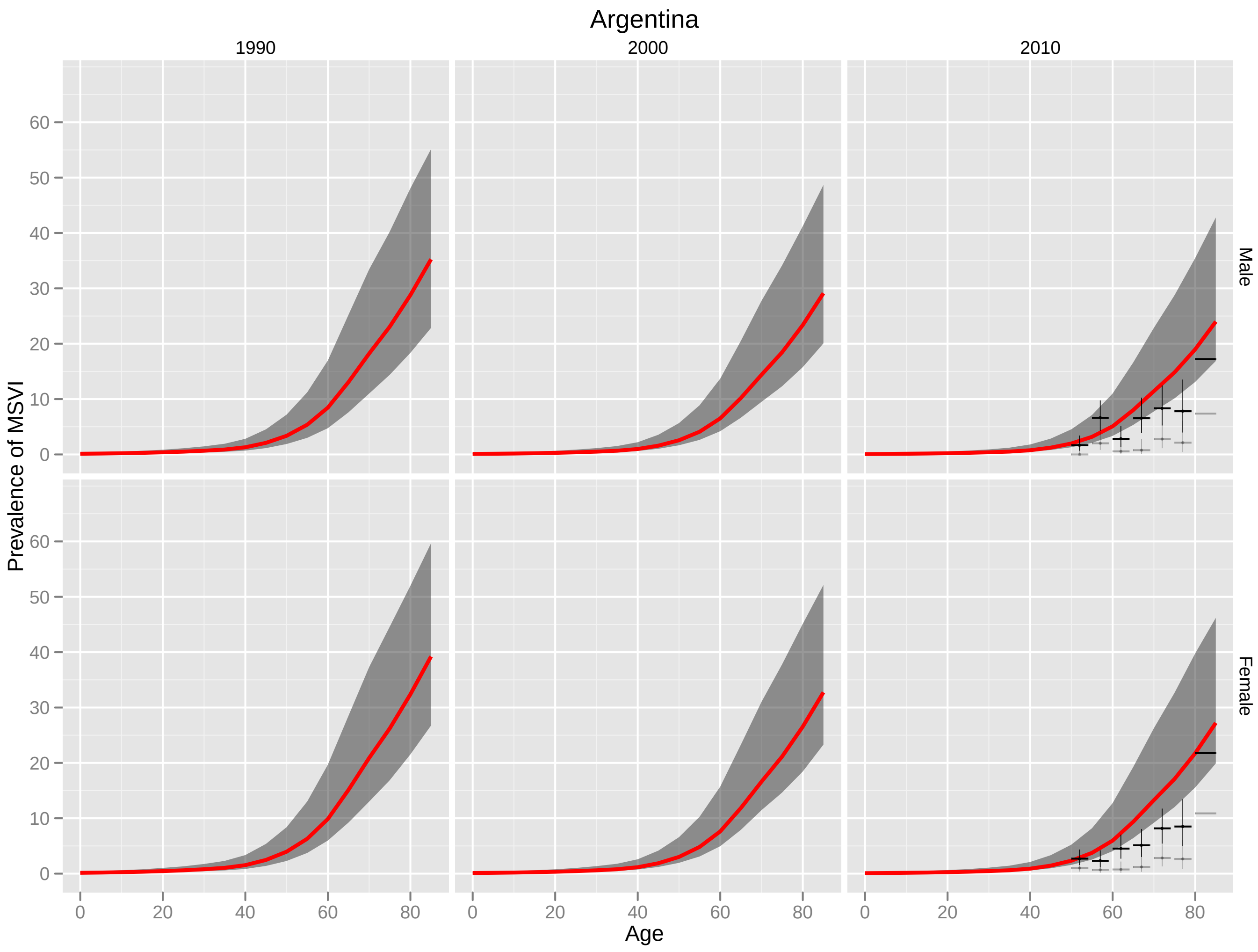
Argentina
1990
2000
2010
Male
Female
Prevalence of MSVI
Age
0
10
20
30
40
50
60
80

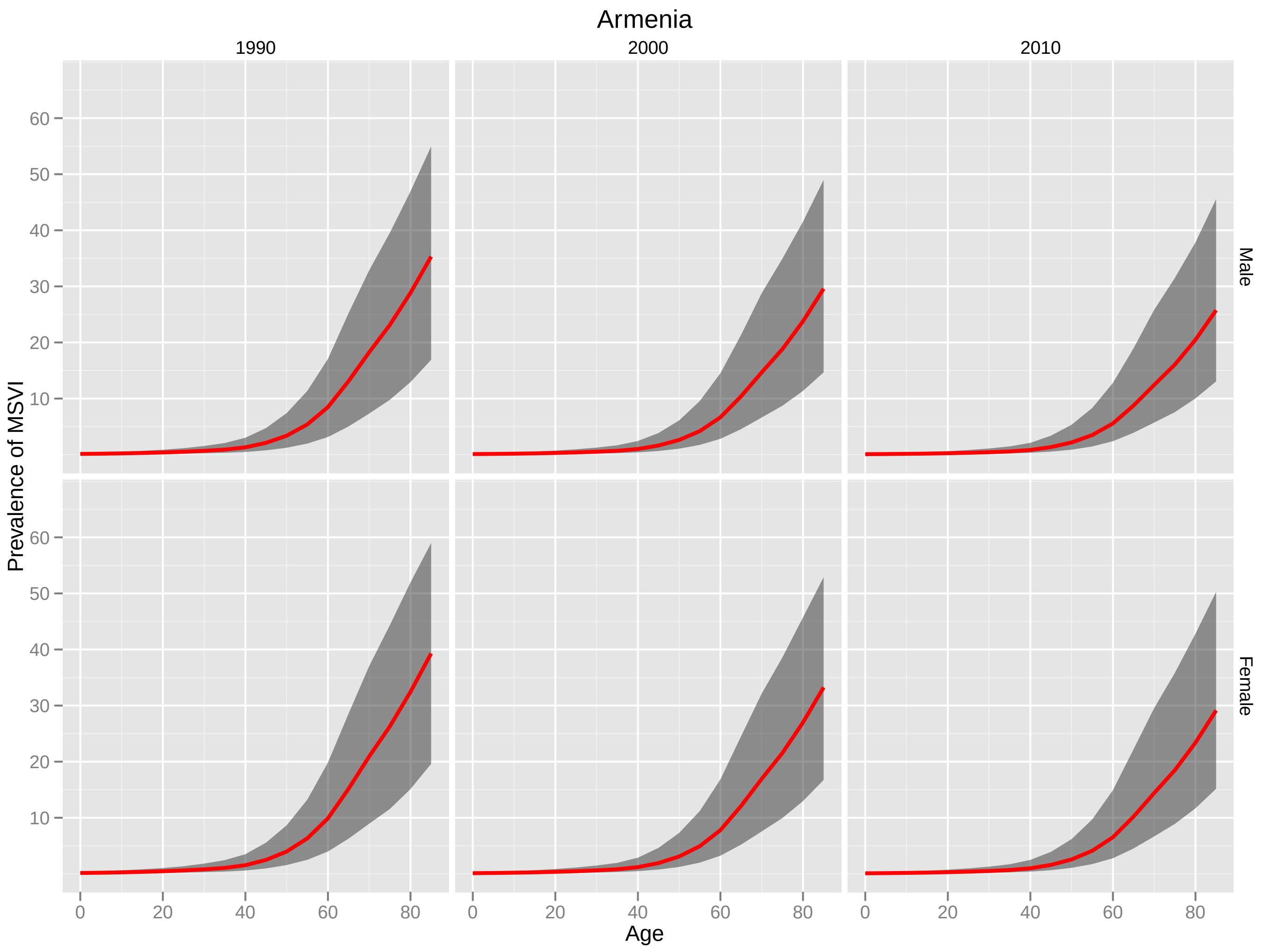
Armenia
1990
2000
2010
Male
Female
Prevalence of MSVI
Age
10
20
30
40
50
60
0
20
40
60
80

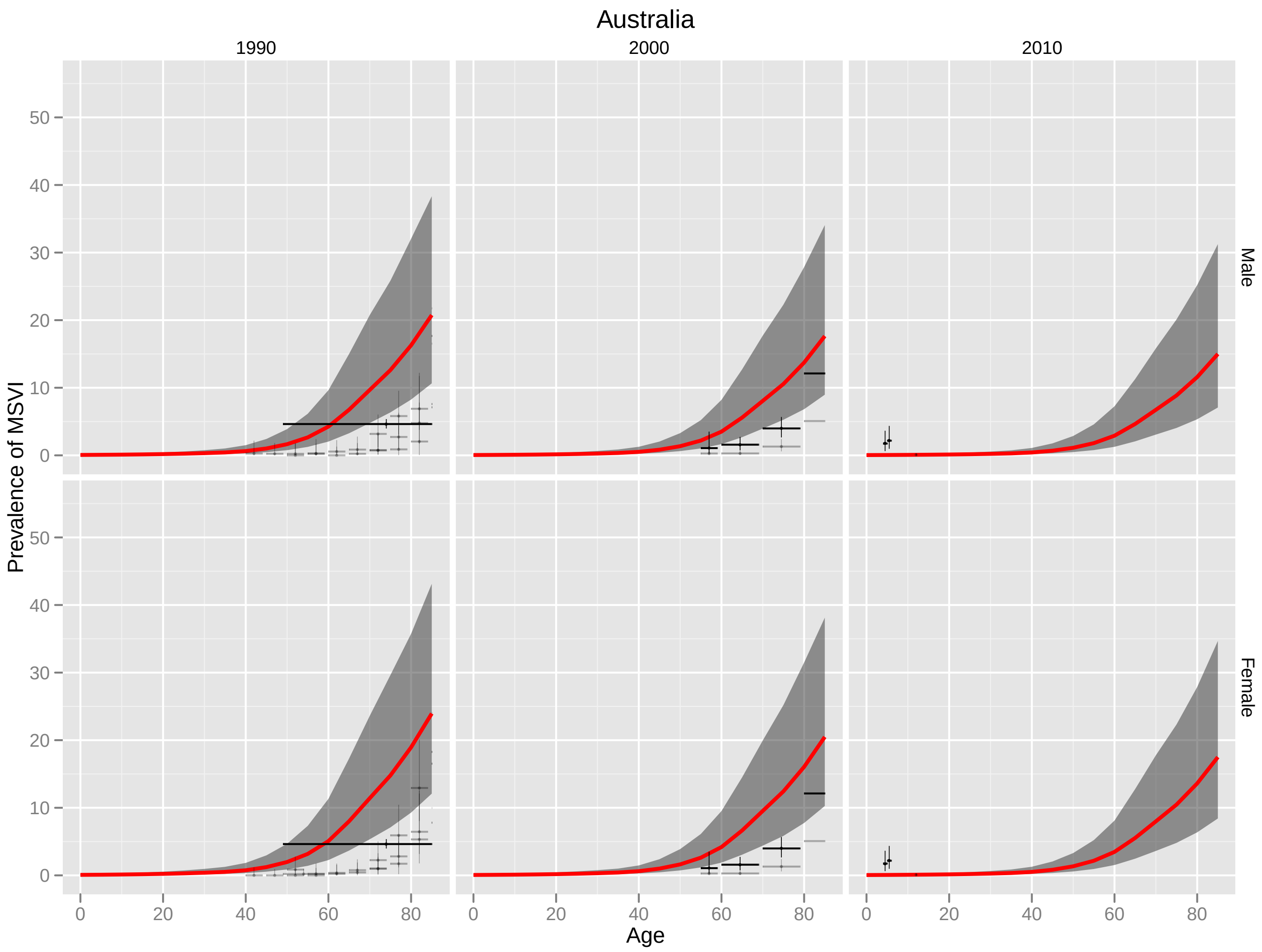

Australia
1990
2000
2010
Male
Female
Prevalence of MSVI
Age
0
10
20
30
40
50
0
20
40
60
80

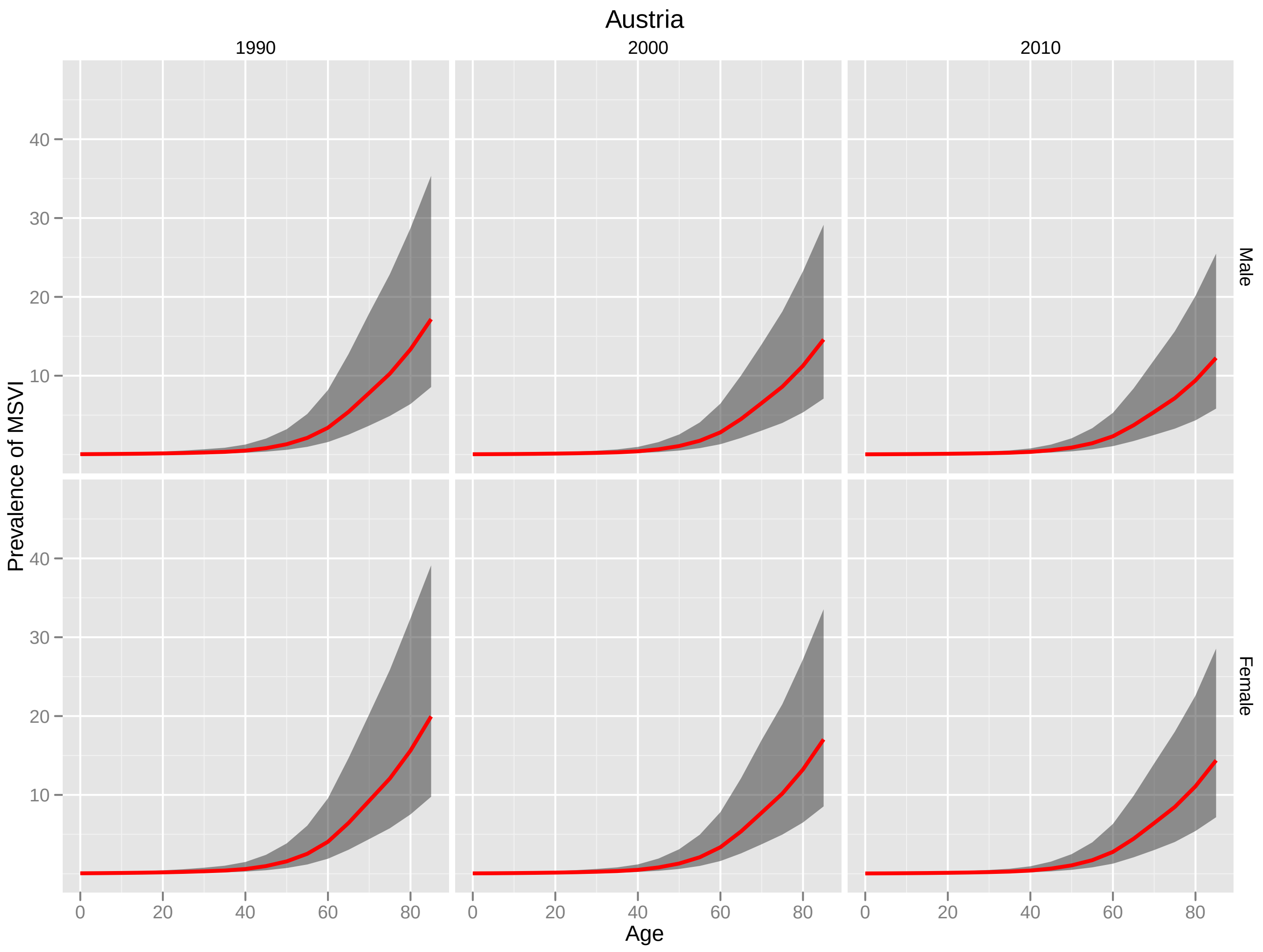
Austria
1990
2000
2010
Male
Female
Prevalence of MSVI
Age
10
20
30
40
0
20
40
60
80

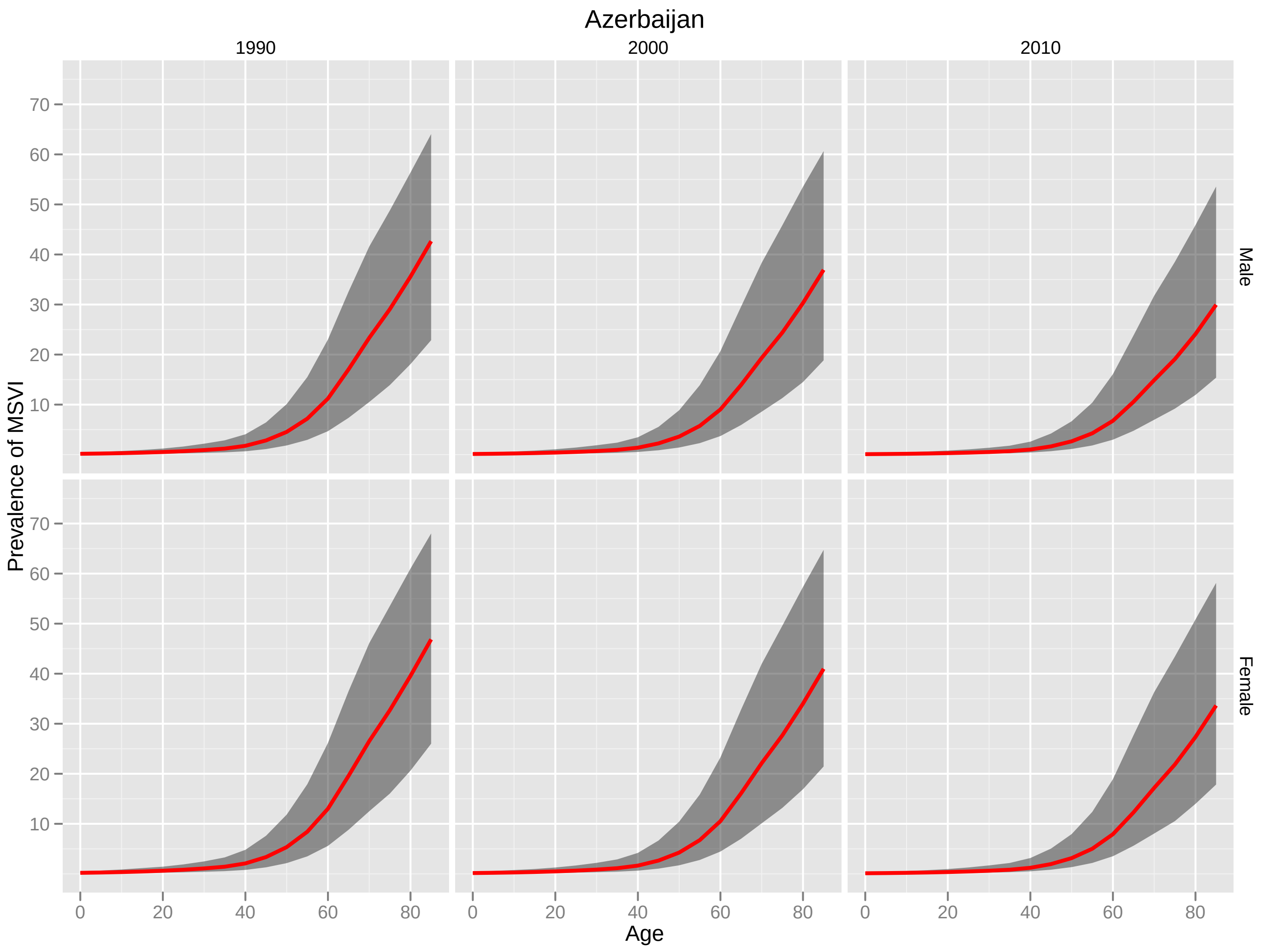
Azerbaijan
1990
2000
2010
Male
Female
Prevalence of MSVI
Age
10
20
30
40
50
60
70
0
20
40
60
80

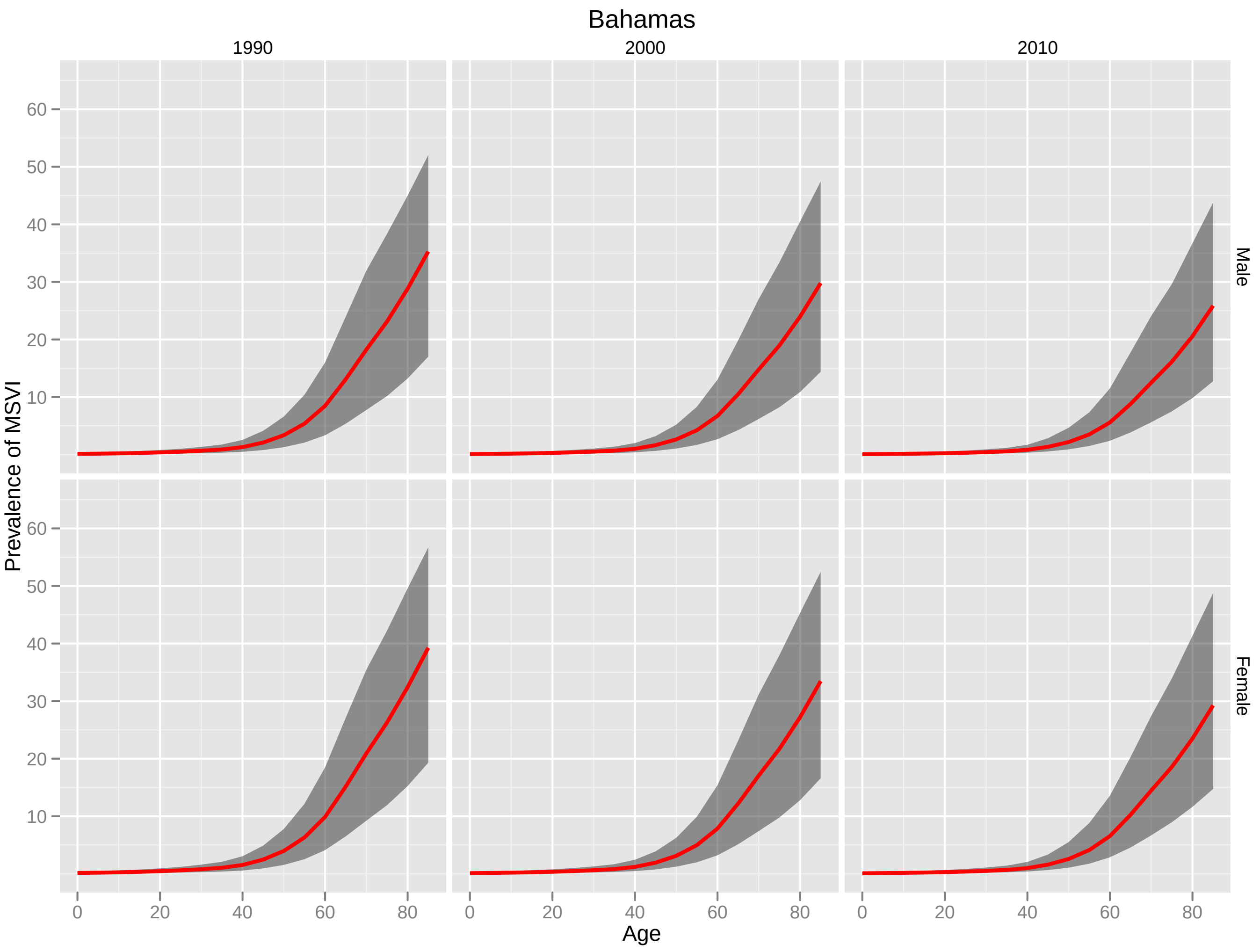

Bahamas
1990
2000
2010
Male
Female
Prevalence of MSVI
Age
10
20
30
40
50
60
0
20
40
60
80

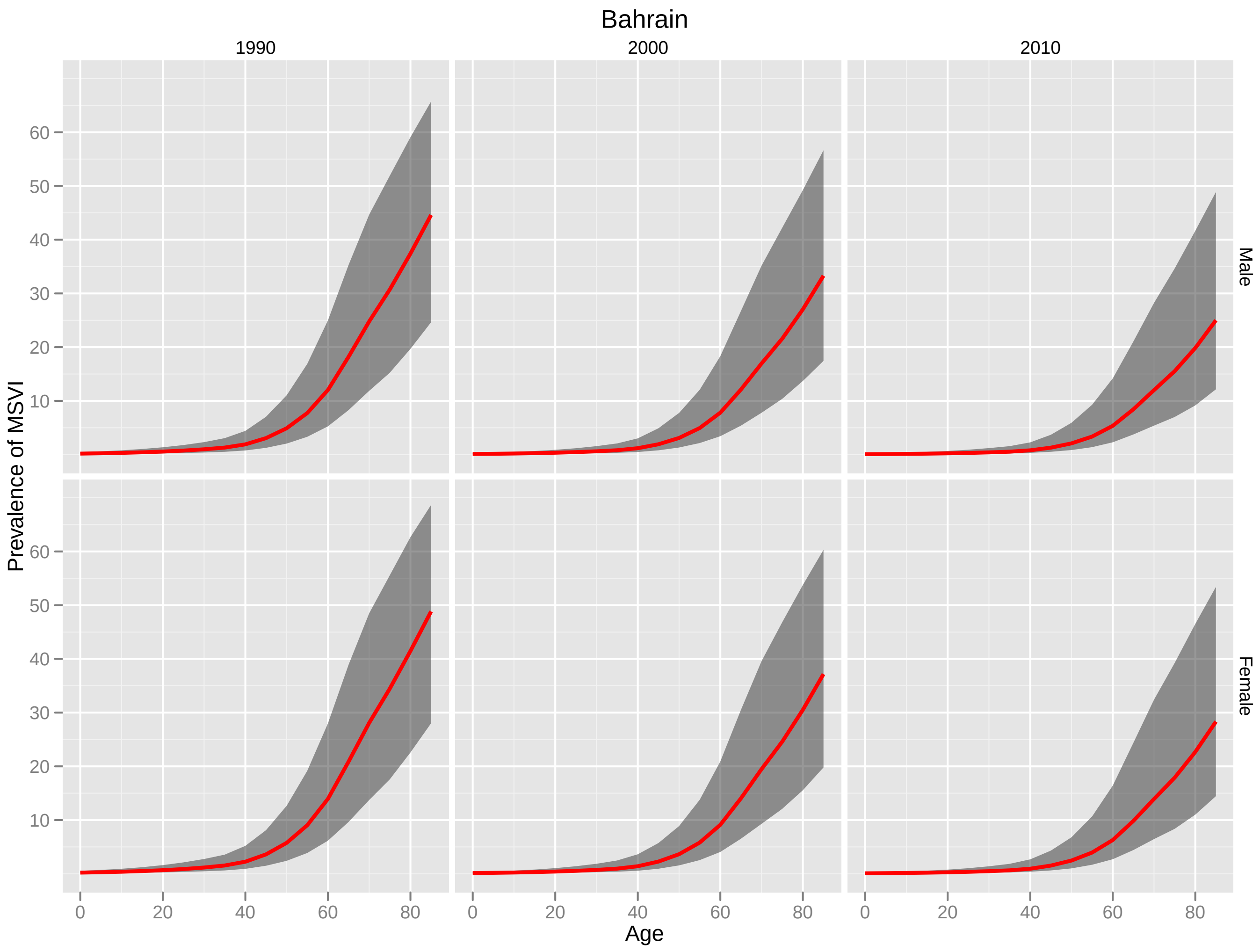
Bahrain
1990
2000
2010
Male
Female
Prevalence of MSVI
Age
10
20
30
40
50
60
0
20
40
60
80

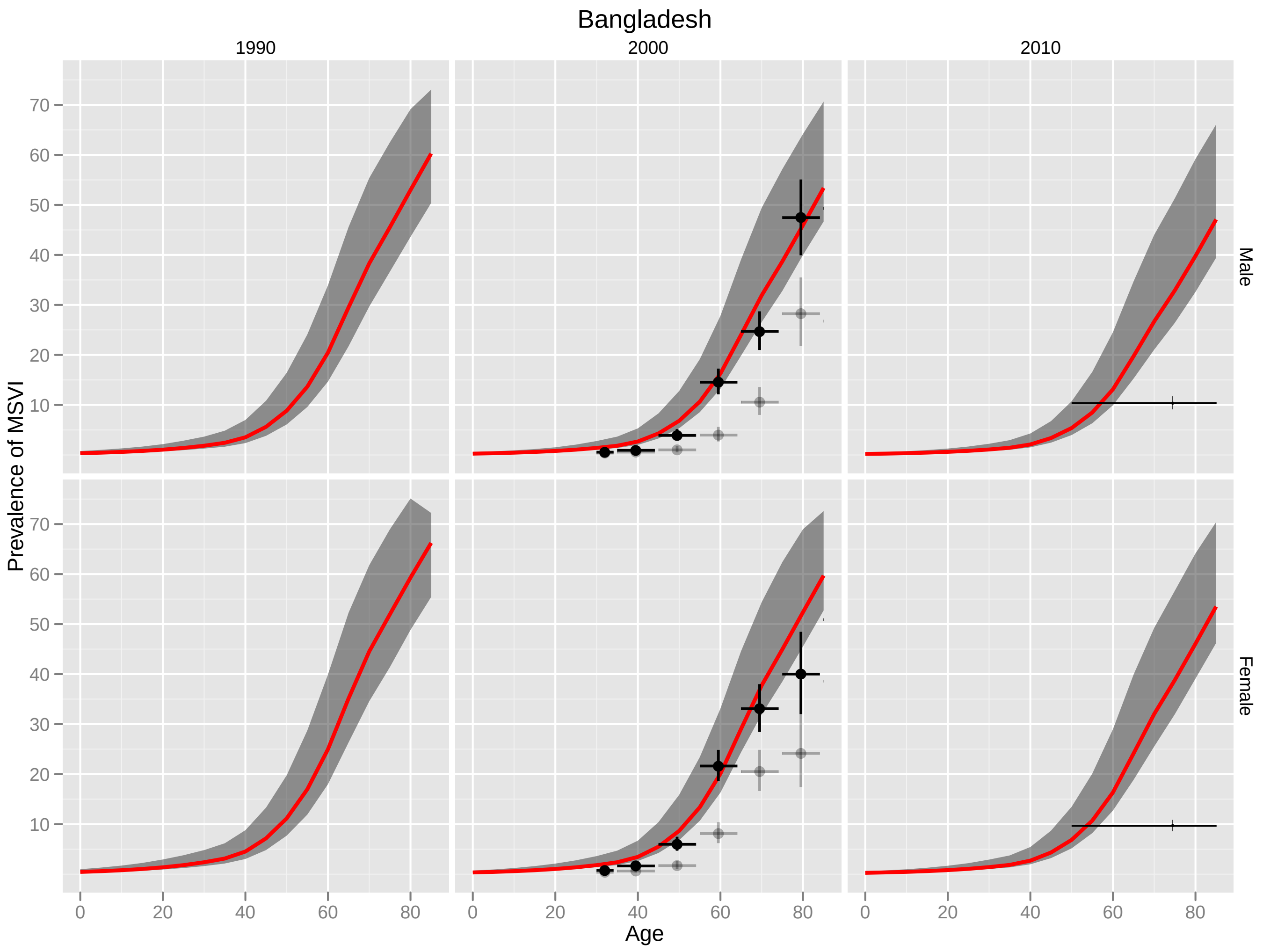
Bangladesh
1990
2000
2010
Male
Female
Prevalence of MSVI
Age
10
20
30
40
50
60
70
0
20
40
60
80

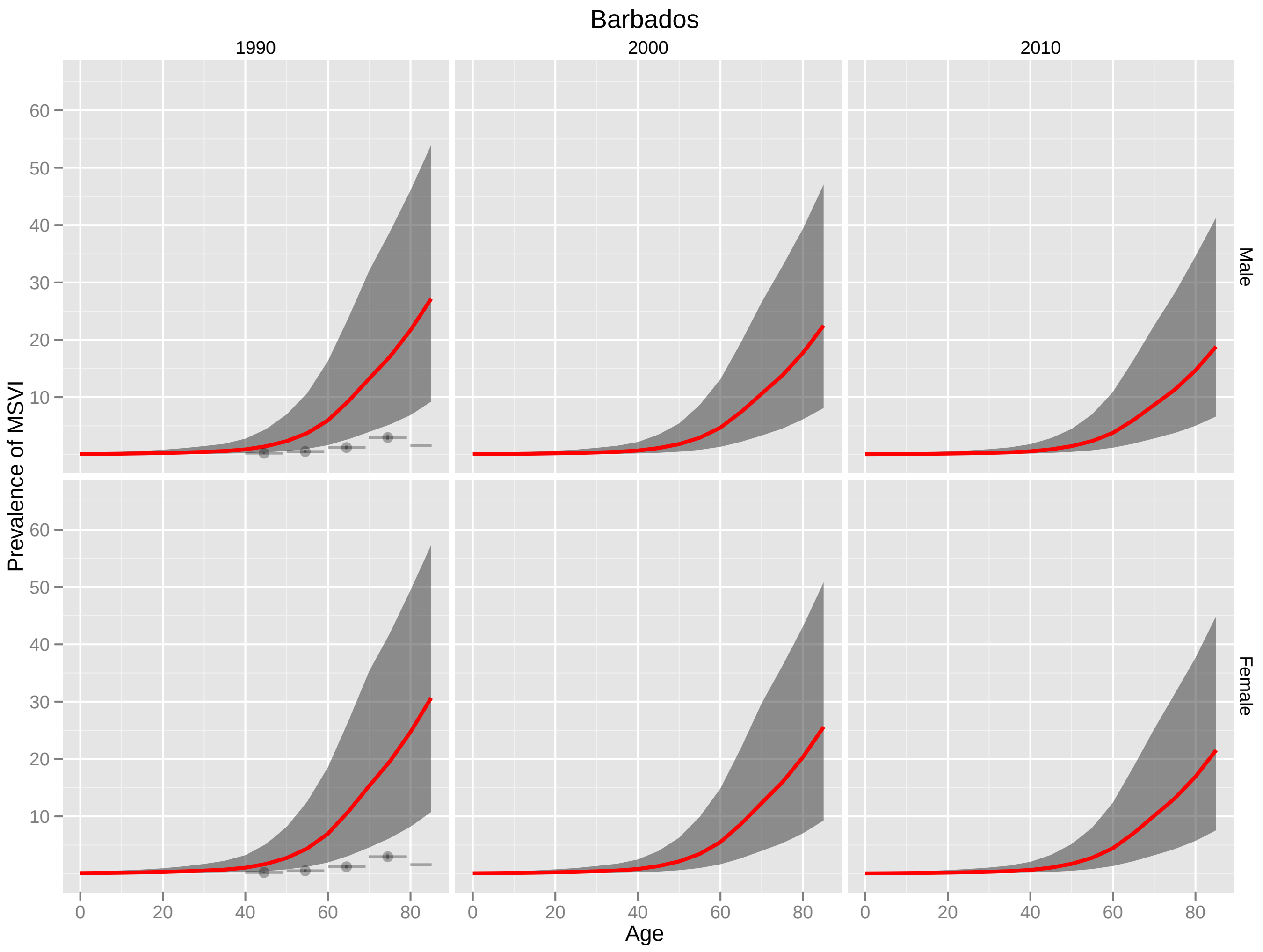
Barbados
1990
2000
2010
Male
Female
Prevalence of MSVI
Age
10
20
30
40
50
60
0
20
40
60
80

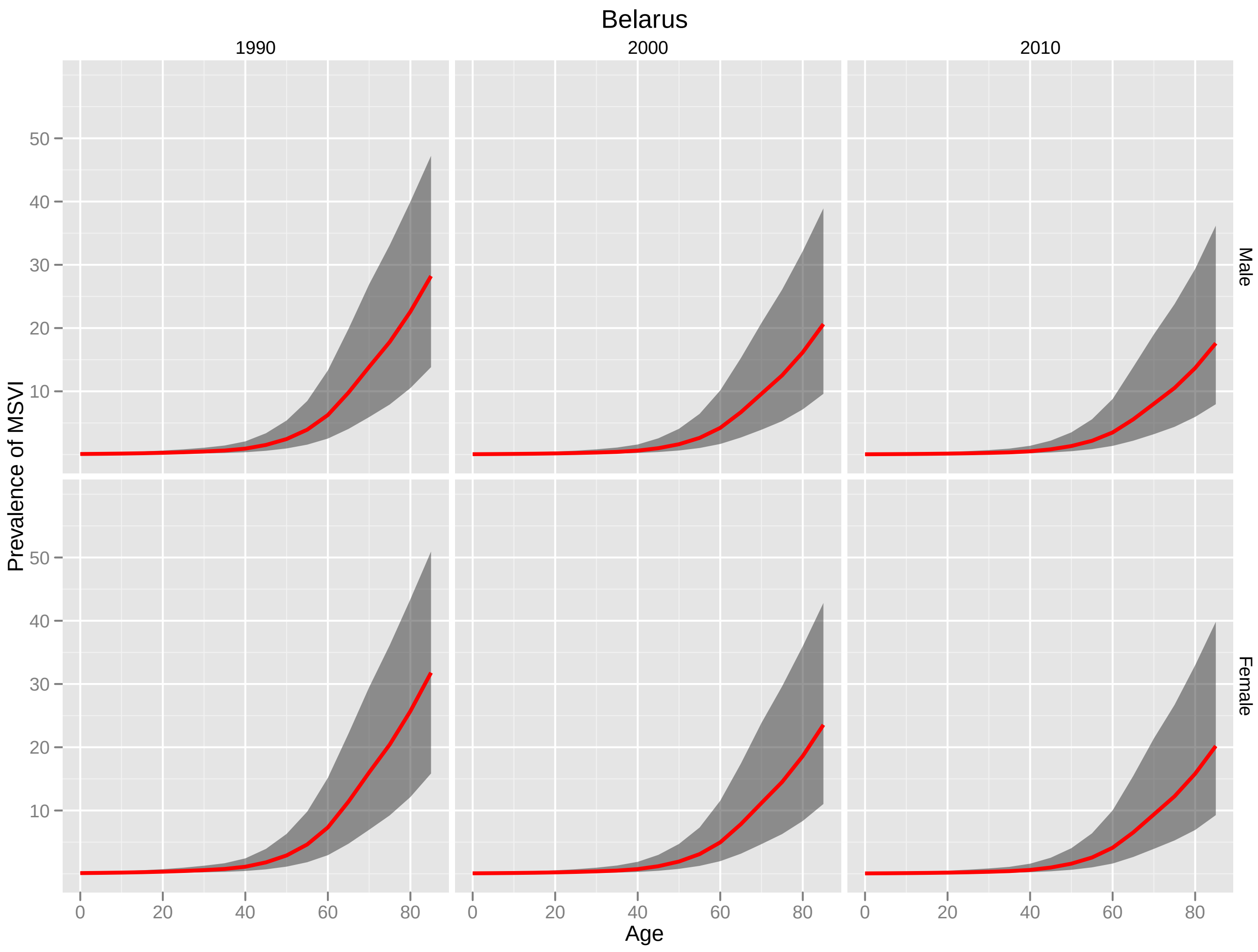

Belarus
1990
2000
2010
Male
Female
Prevalence of MSVI
Age
10
20
30
40
50
0
20
40
60
80

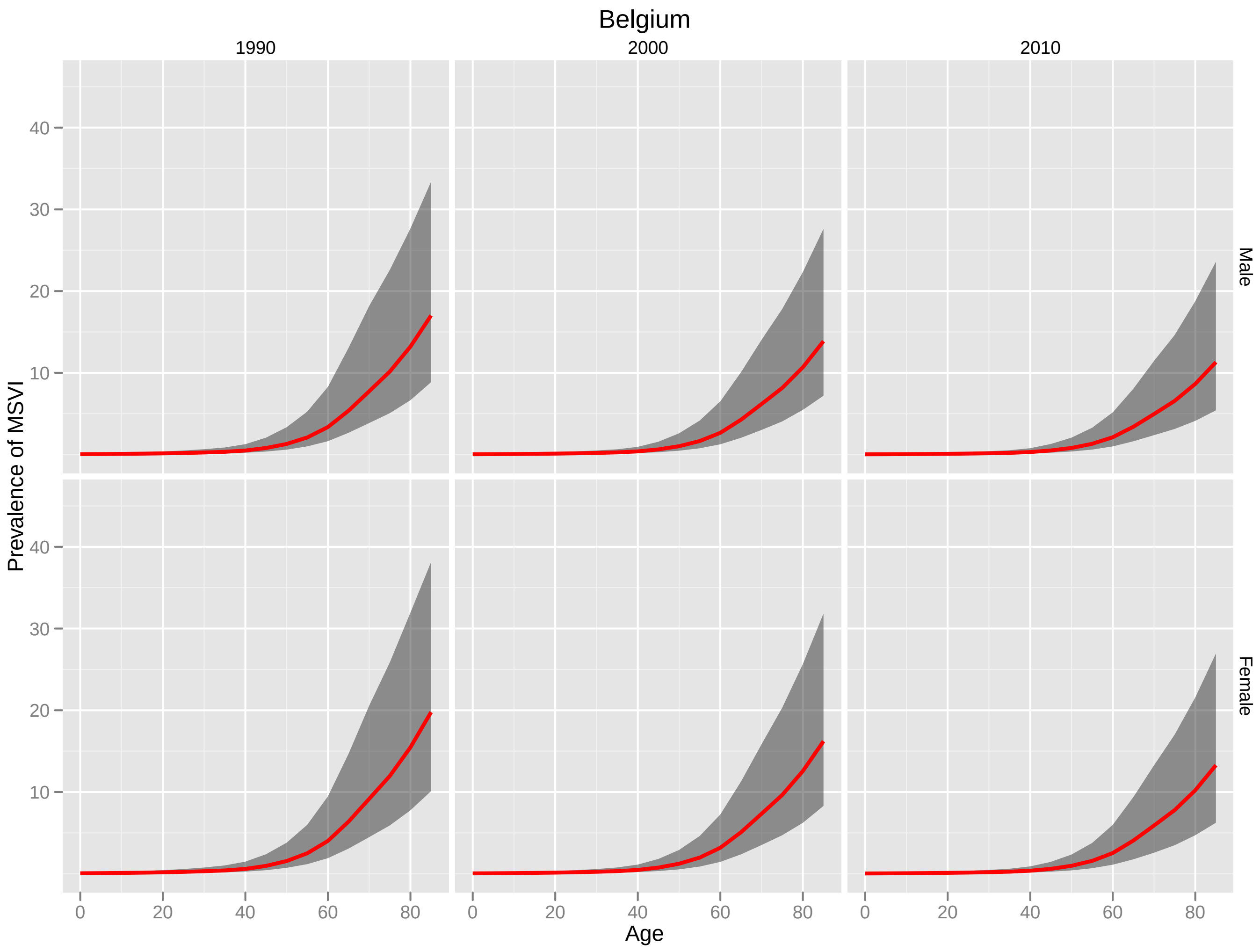
Belgium
1990
2000
2010
Male
Female
Prevalence of MSVI
Age
0
20
40
60
80
10
20
30
40

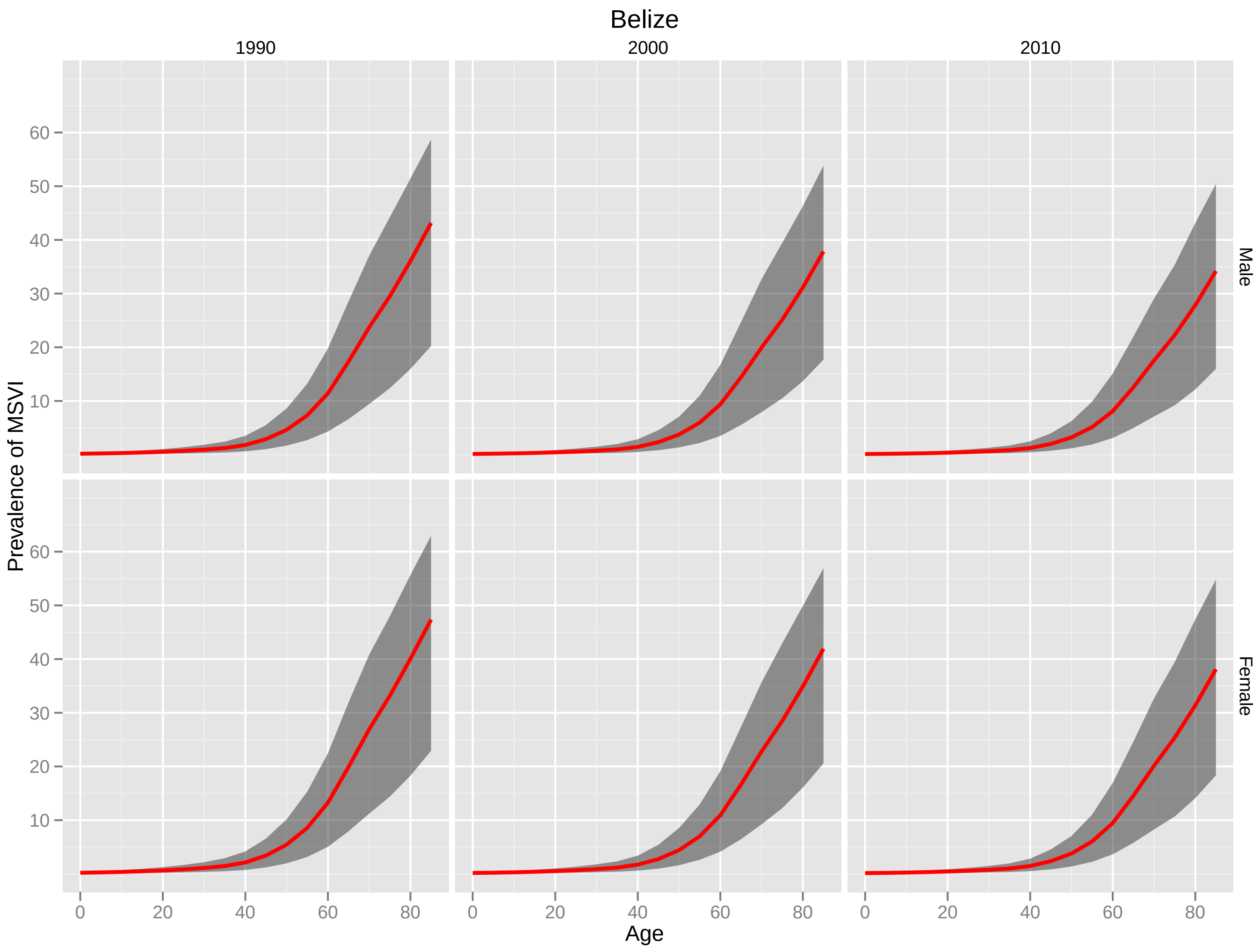

Belize
1990
2000
2010
Male
Female
Prevalence of MSVI
Age
10
20
30
40
50
60
0
20
40
60
80

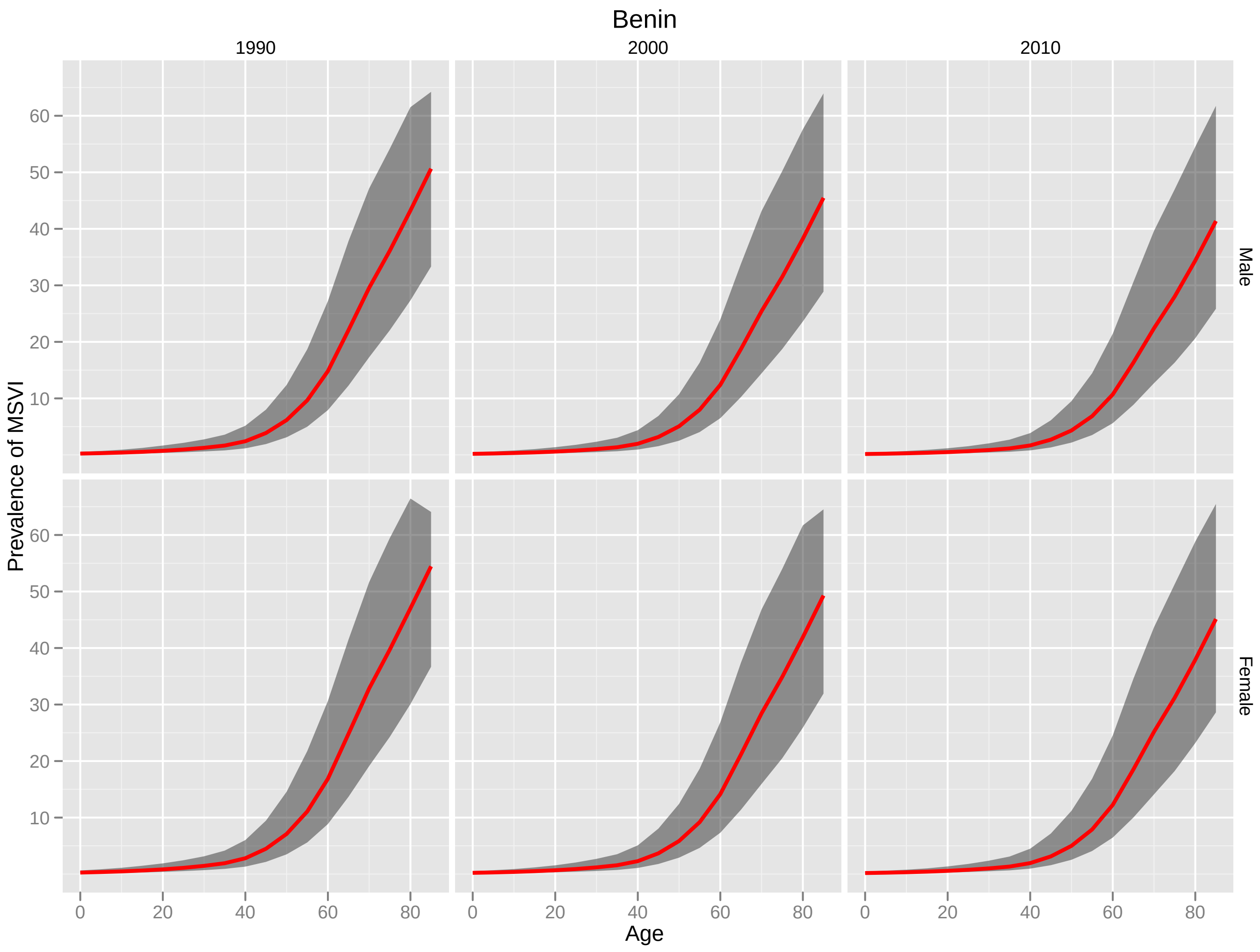
Benin
1990
2000
2010
Male
Female
Prevalence of MSVI
Age
10
20
30
40
50
60
0
20
40
60
80

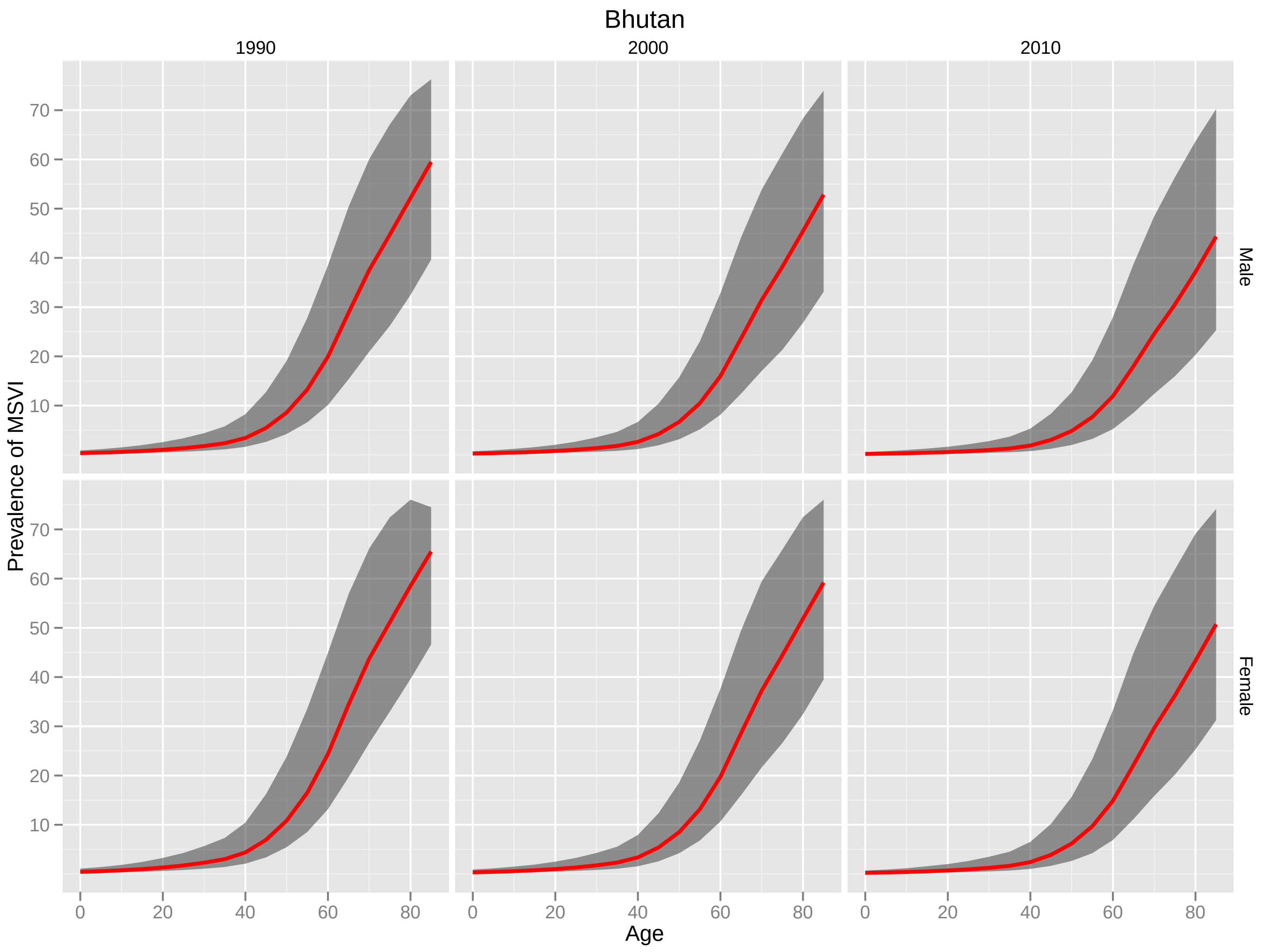
Bhutan
1990
2000
2010
Male
Female
Prevalence of MSVI
Age
10
20
30
40
50
60
70
0
20
40
60
80

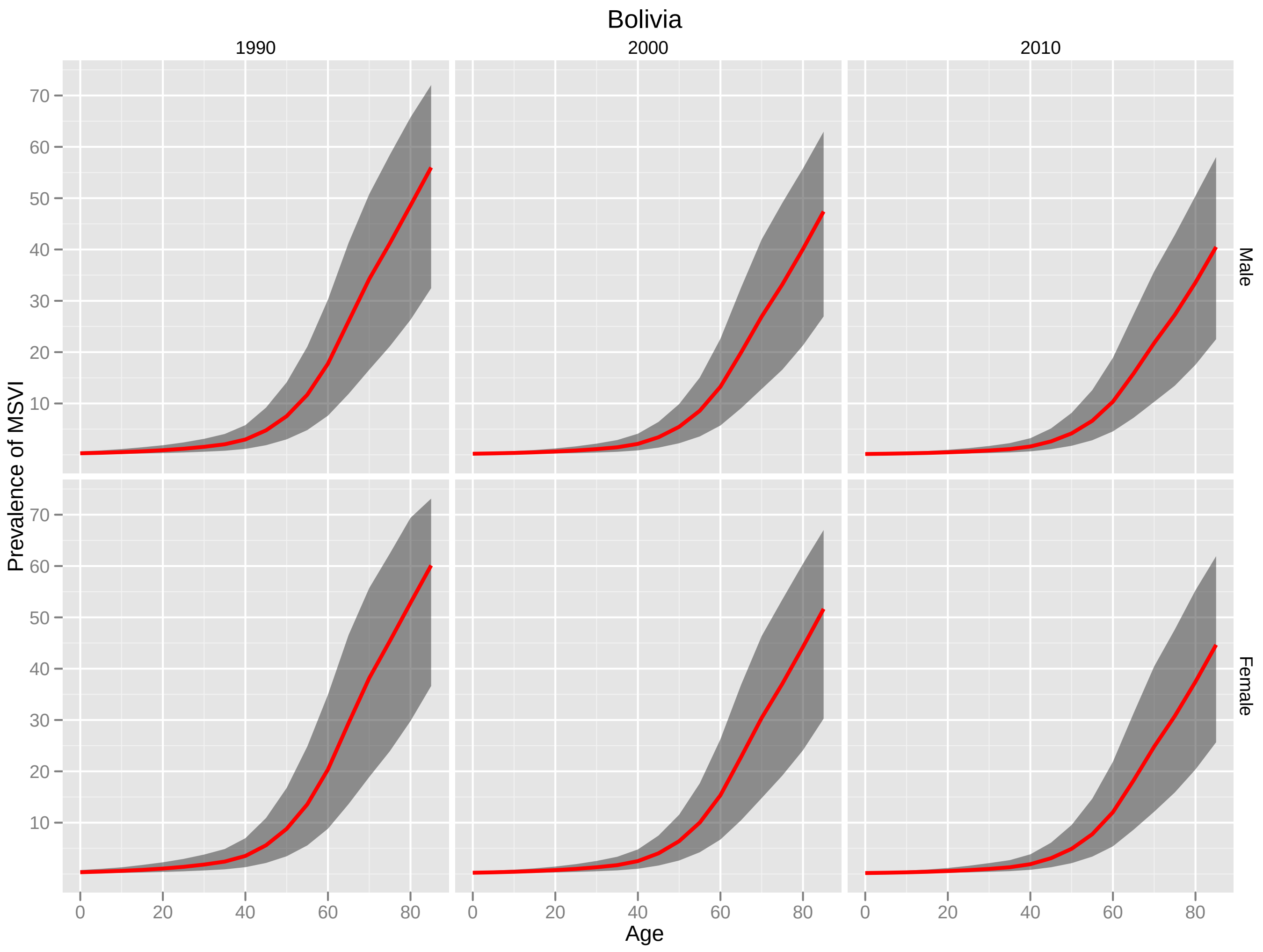
Bolivia
1990
2000
2010
Male
Female
Prevalence of MSVI
Age
10
20
30
40
50
60
70
0
20
40
60
80

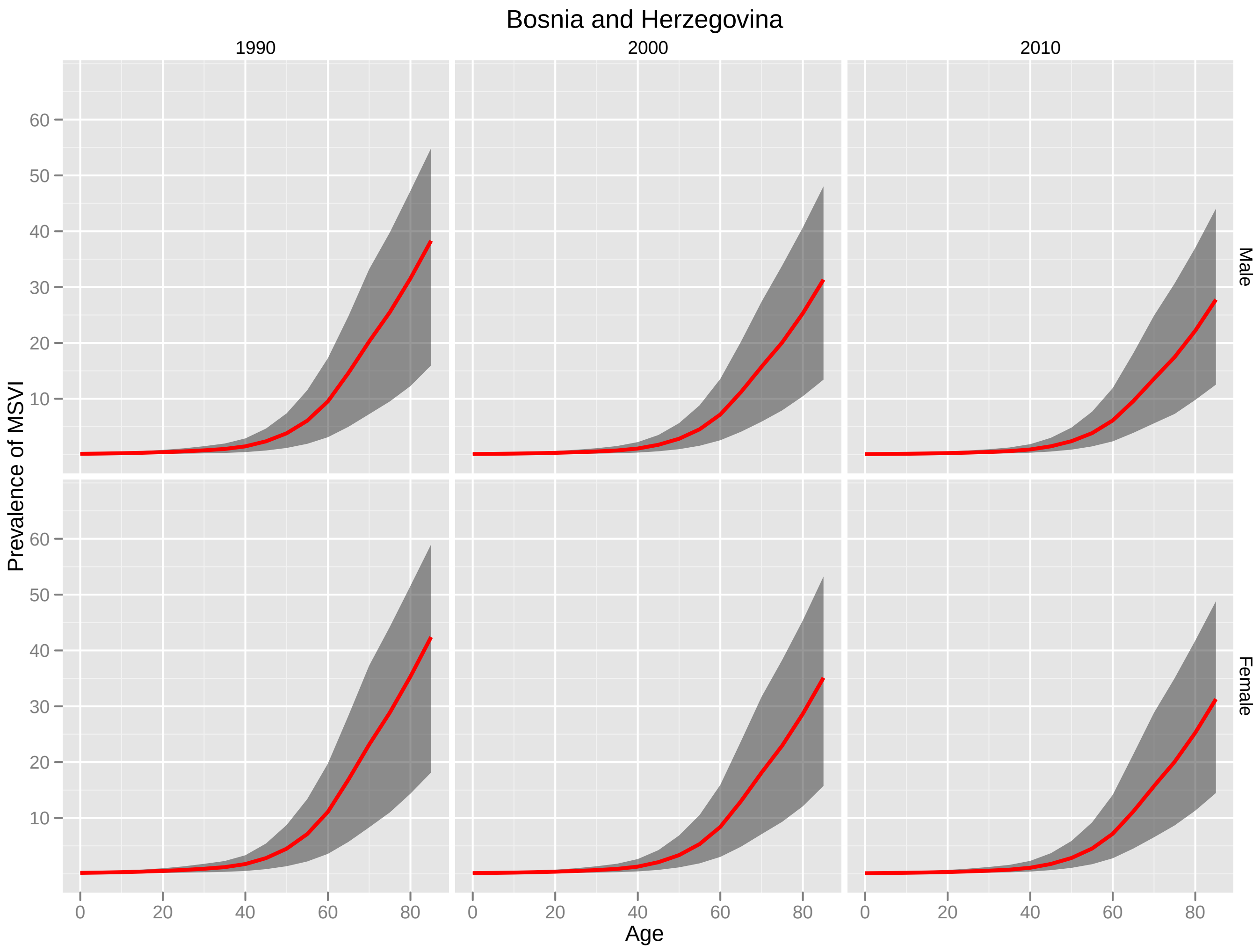
Bosnia and Herzegovina
1990
2000
2010
Male
Female
Prevalence of MSVI
Age
10
20
30
40
50
60
0
20
40
60
80

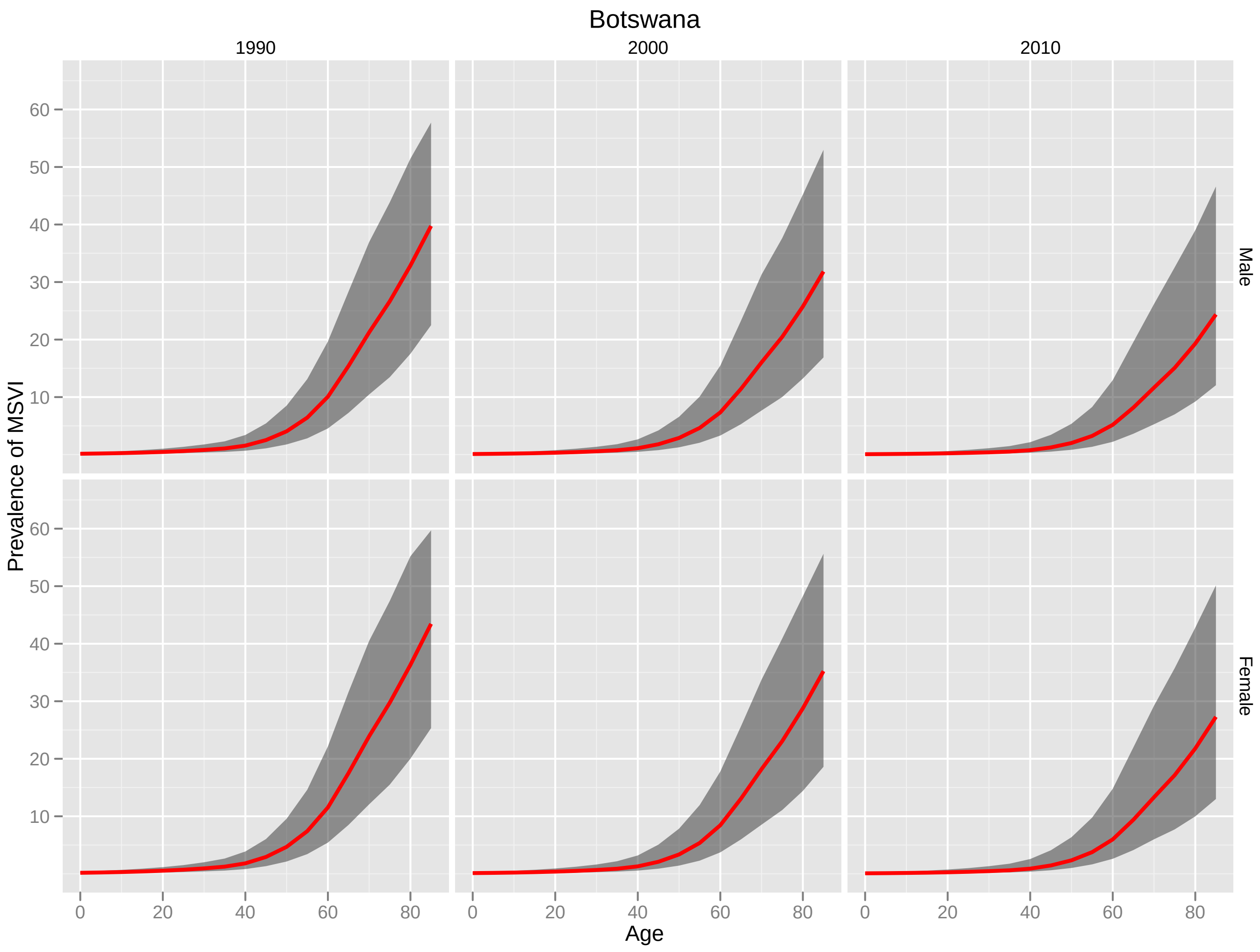
Botswana
1990
2000
2010
Male
Female
Prevalence of MSVI
10
20
30
40
50
60
Age
0
20
40
60
80

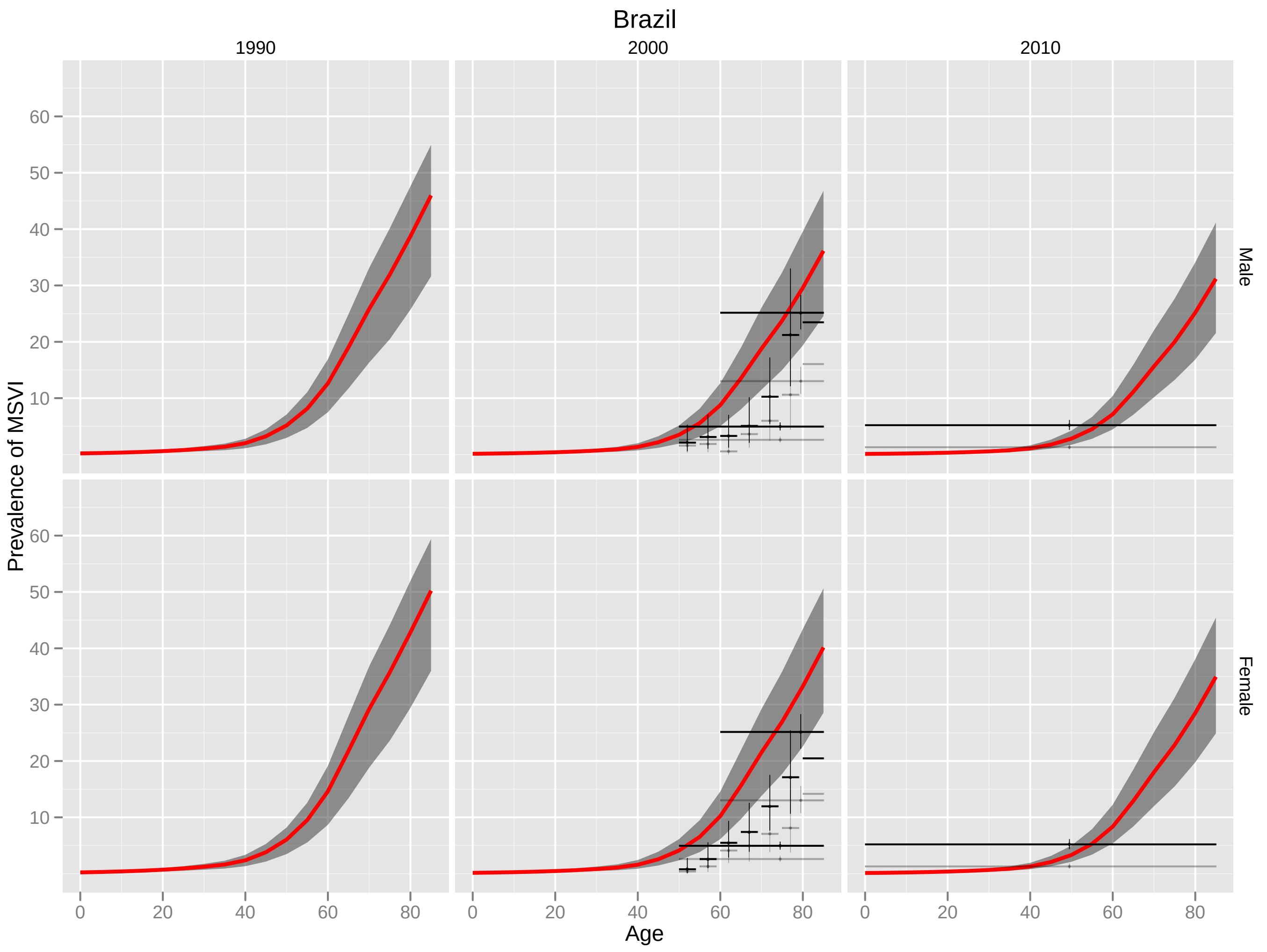
Brazil
1990
2000
2010
Male
Female
Prevalence of MSVI
10
20
30
40
50
60
0
20
40
60
80
Age

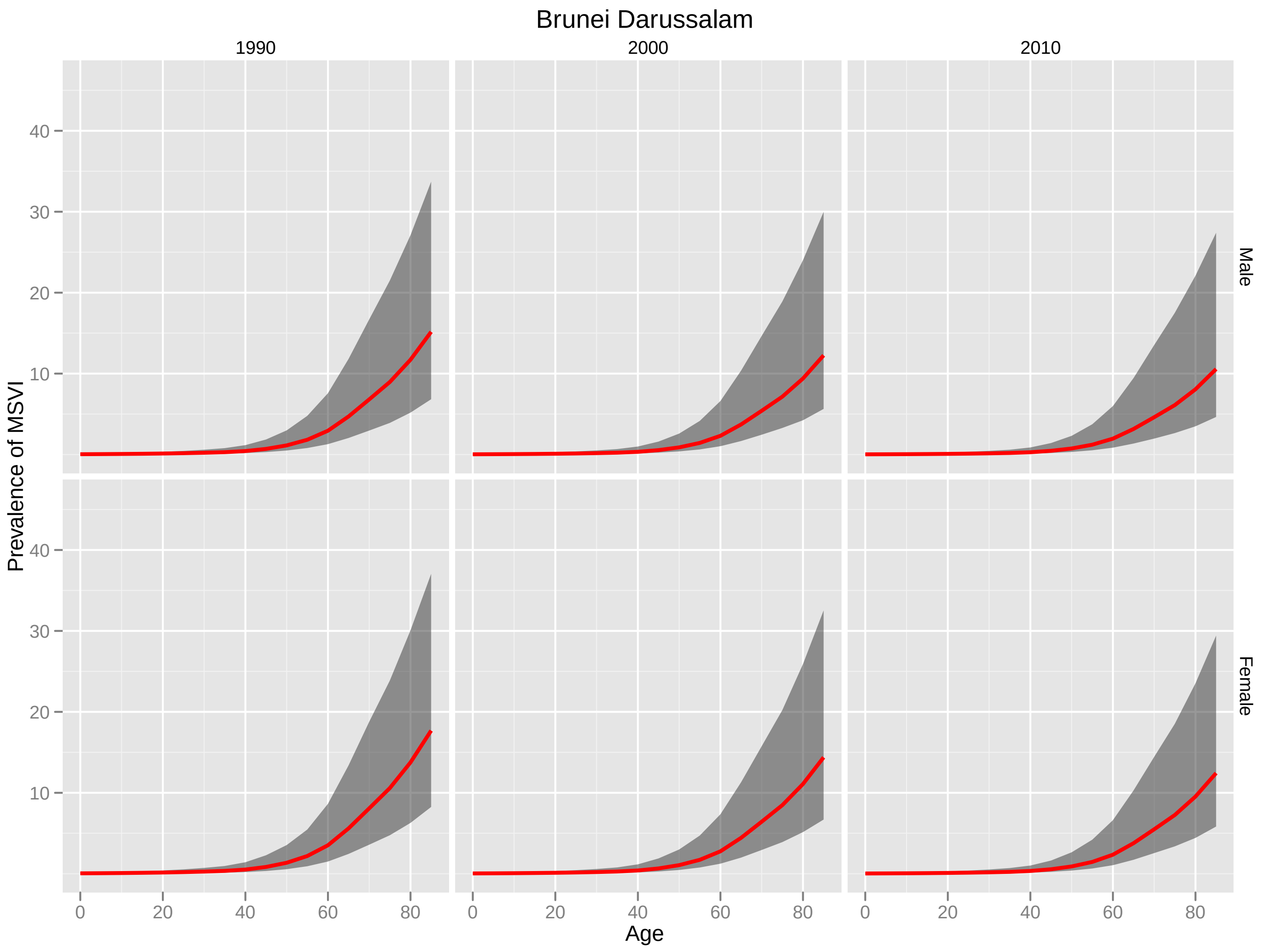
Brunei Darussalam
1990
2000
2010
Male
Female
Prevalence of MSVI
Age
0
20
40
60
80
10
20
30
40

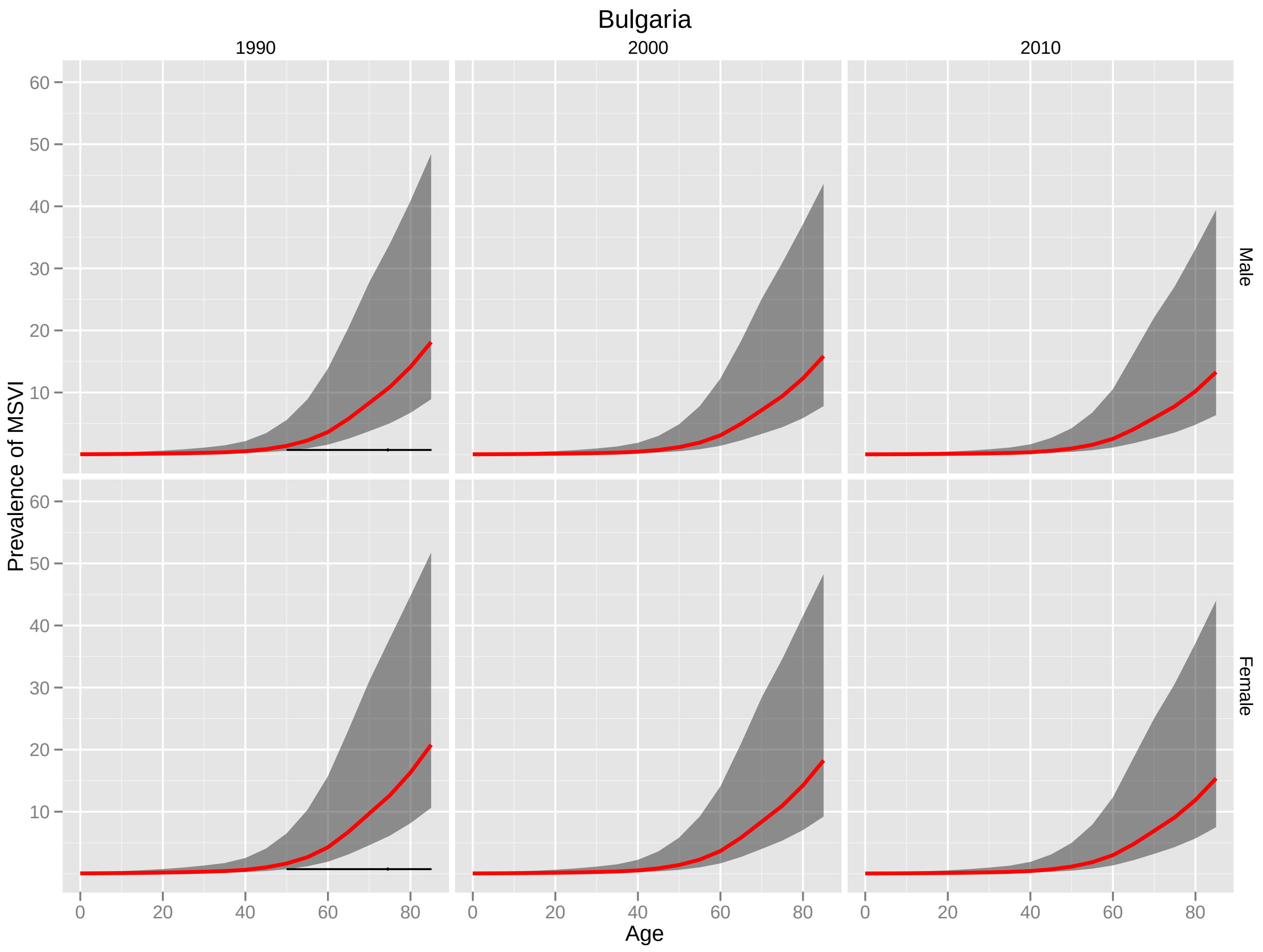
Bulgaria
1990
2000
2010
Male
Female
Prevalence of MSVI
Age
10
20
30
40
50
60
0
20
40
60
80

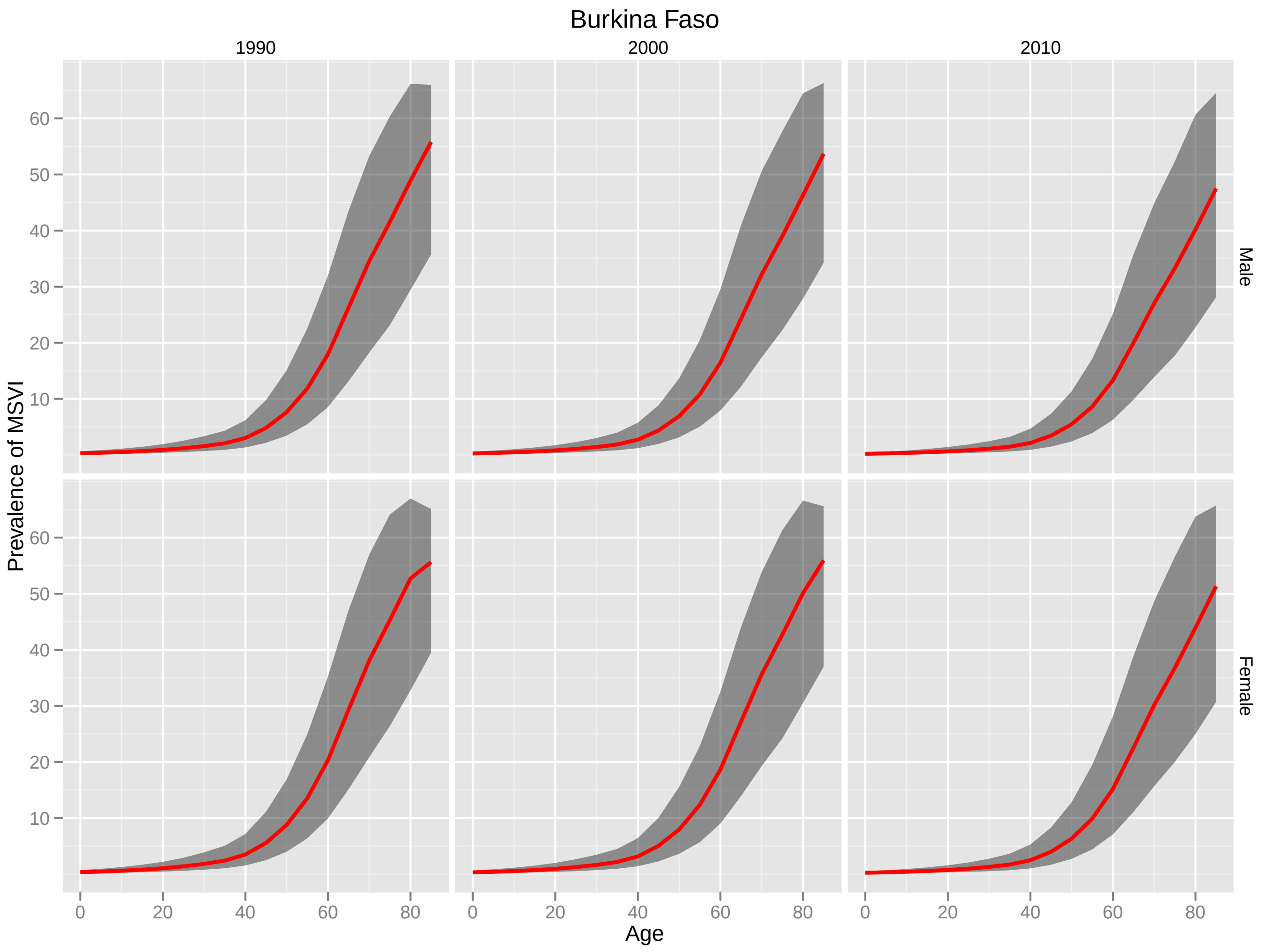
Burkina Faso
1990
2000
2010
Male
Female
Prevalence of MSVI
Age
10
20
30
40
50
60
0
20
40
60
80

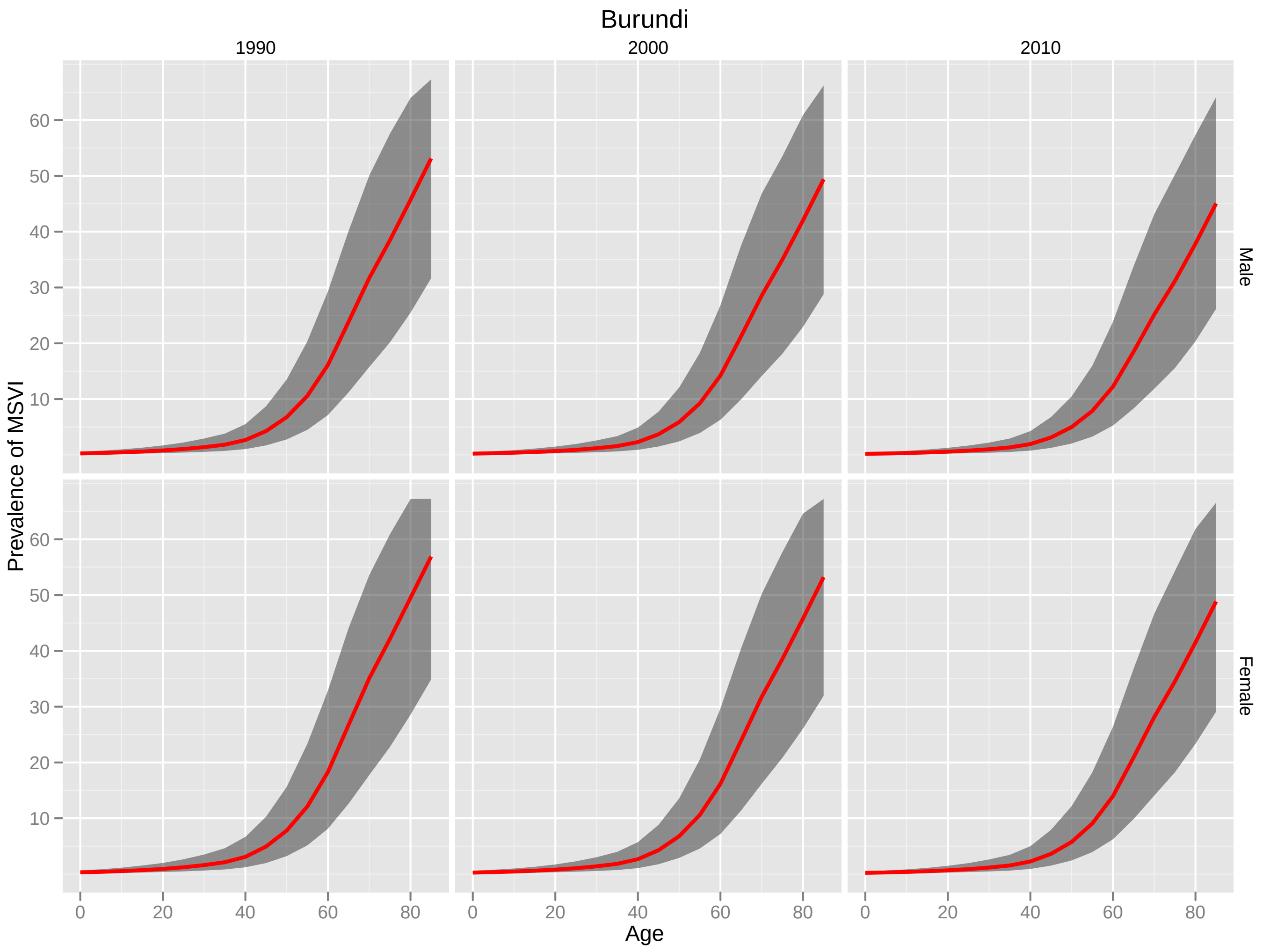
Burundi
1990
2000
2010
Male
Female
Prevalence of MSVI
10
20
30
40
50
60
Age
0
20
40
60
80

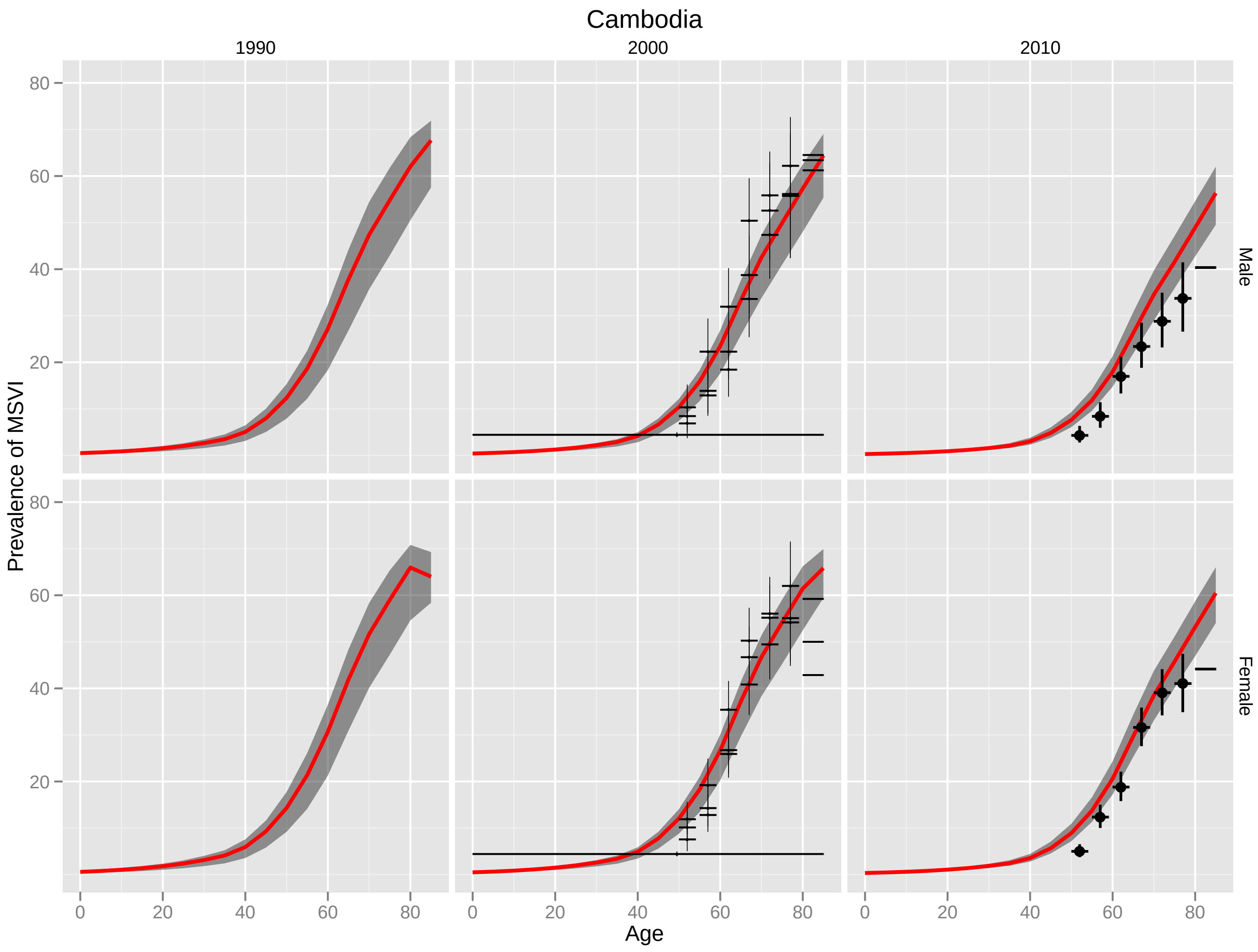
Cambodia
1990
2000
2010
Male
Female
Prevalence of MSVI
Age
0
20
40
60
80

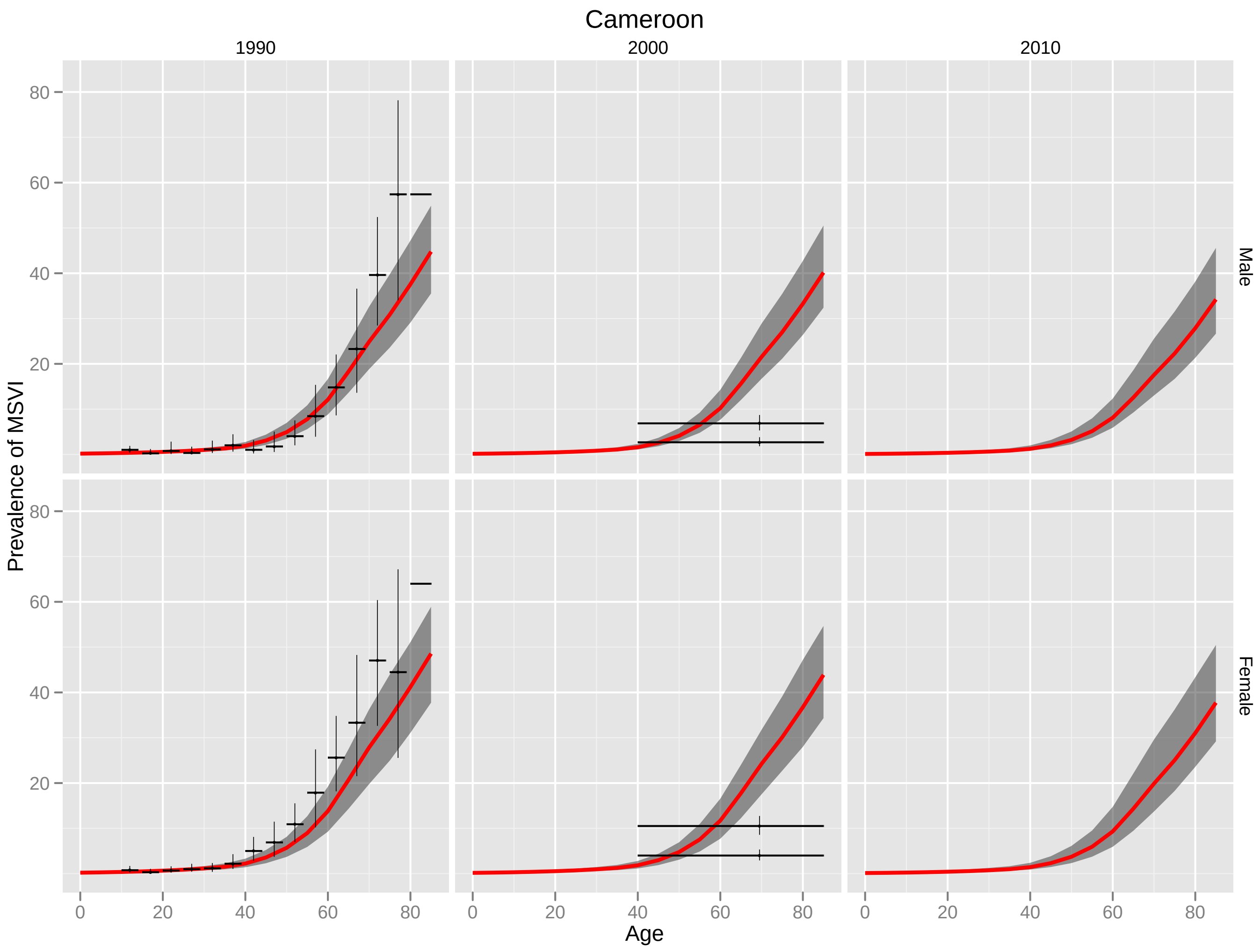
Cameroon
1990
2000
2010
Male
Female
Prevalence of MSVI
Age
0
20
40
60
80

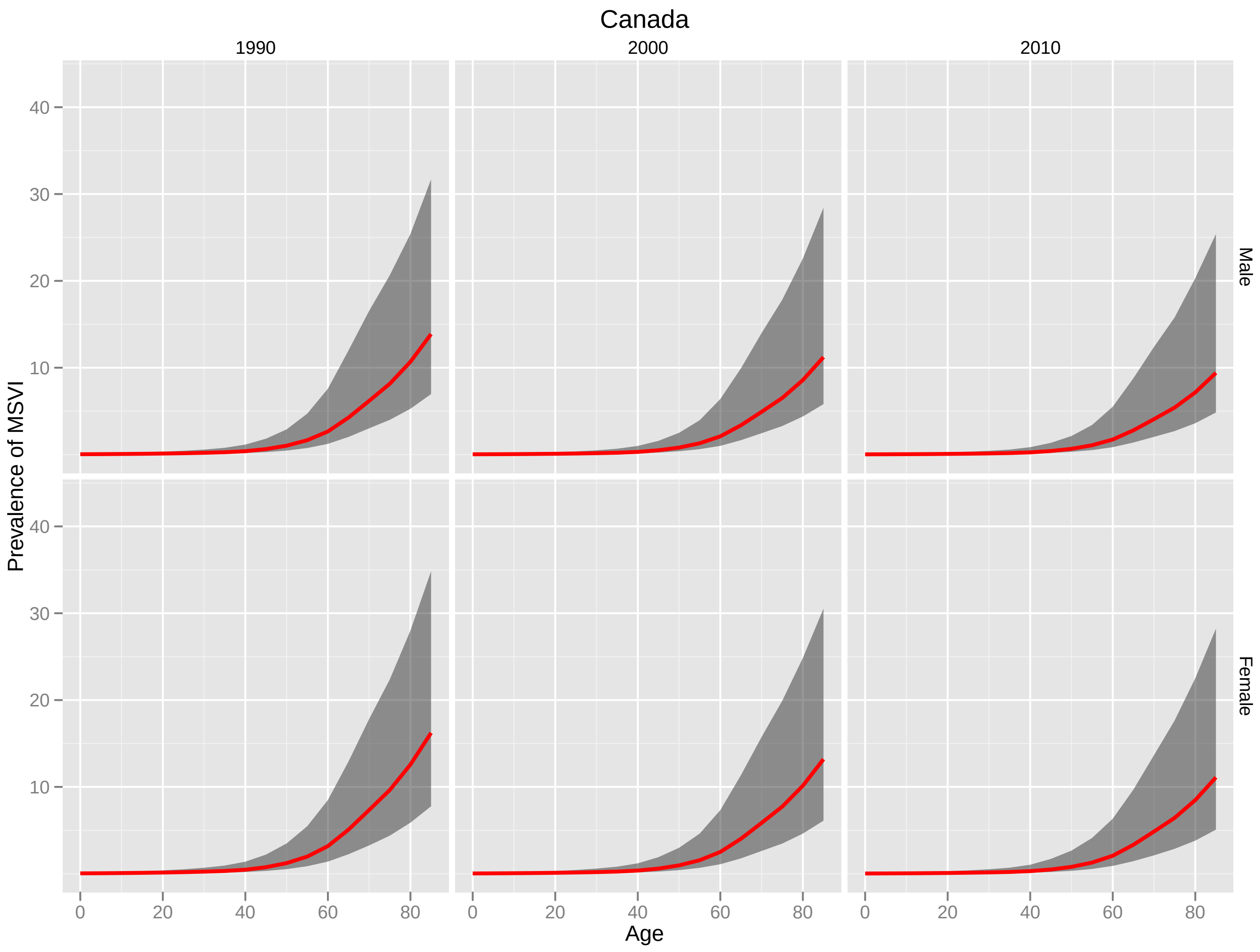
Canada
1990
2000
2010
Male
Female
Prevalence of MSVI
Age
0
20
40
60
80
10
20
30
40

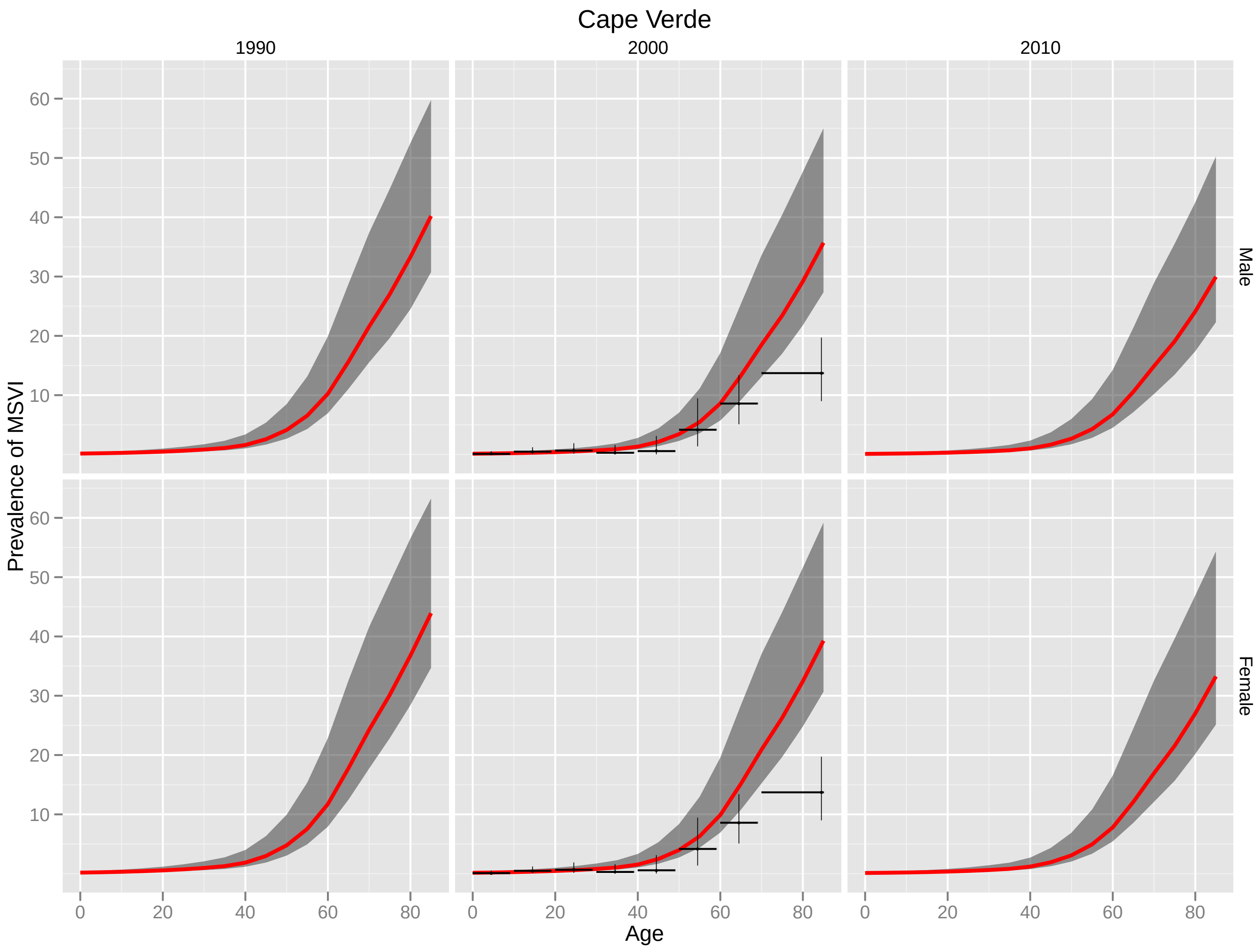
Cape Verde
1990
2000
2010
Male
Female
Prevalence of MSVI
Age
10
20
30
40
50
60
0
20
40
60
80

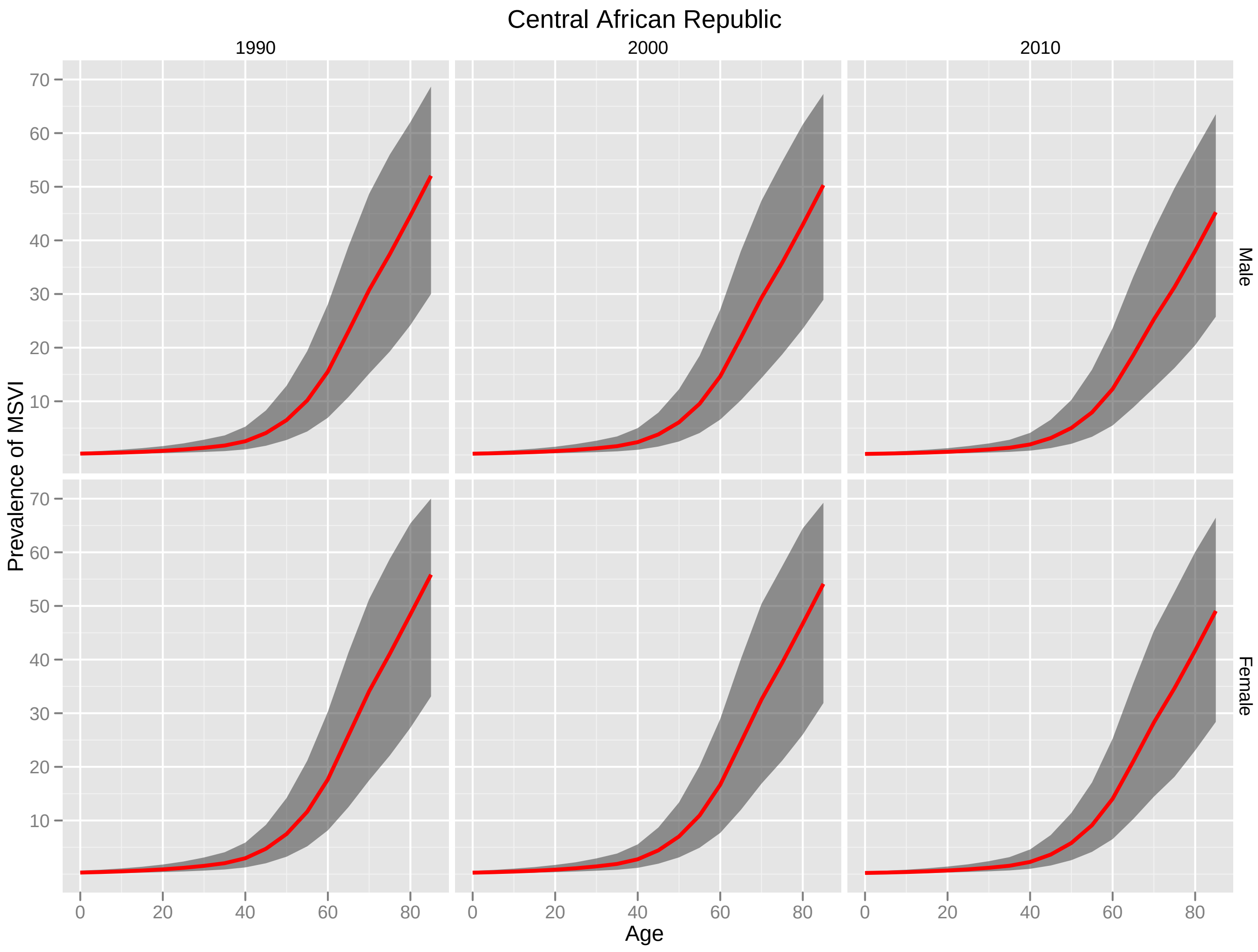
Central African Republic
1990
2000
2010
Male
Female
Prevalence of MSVI
Age
10
20
30
40
50
60
70
0
20
40
60
80

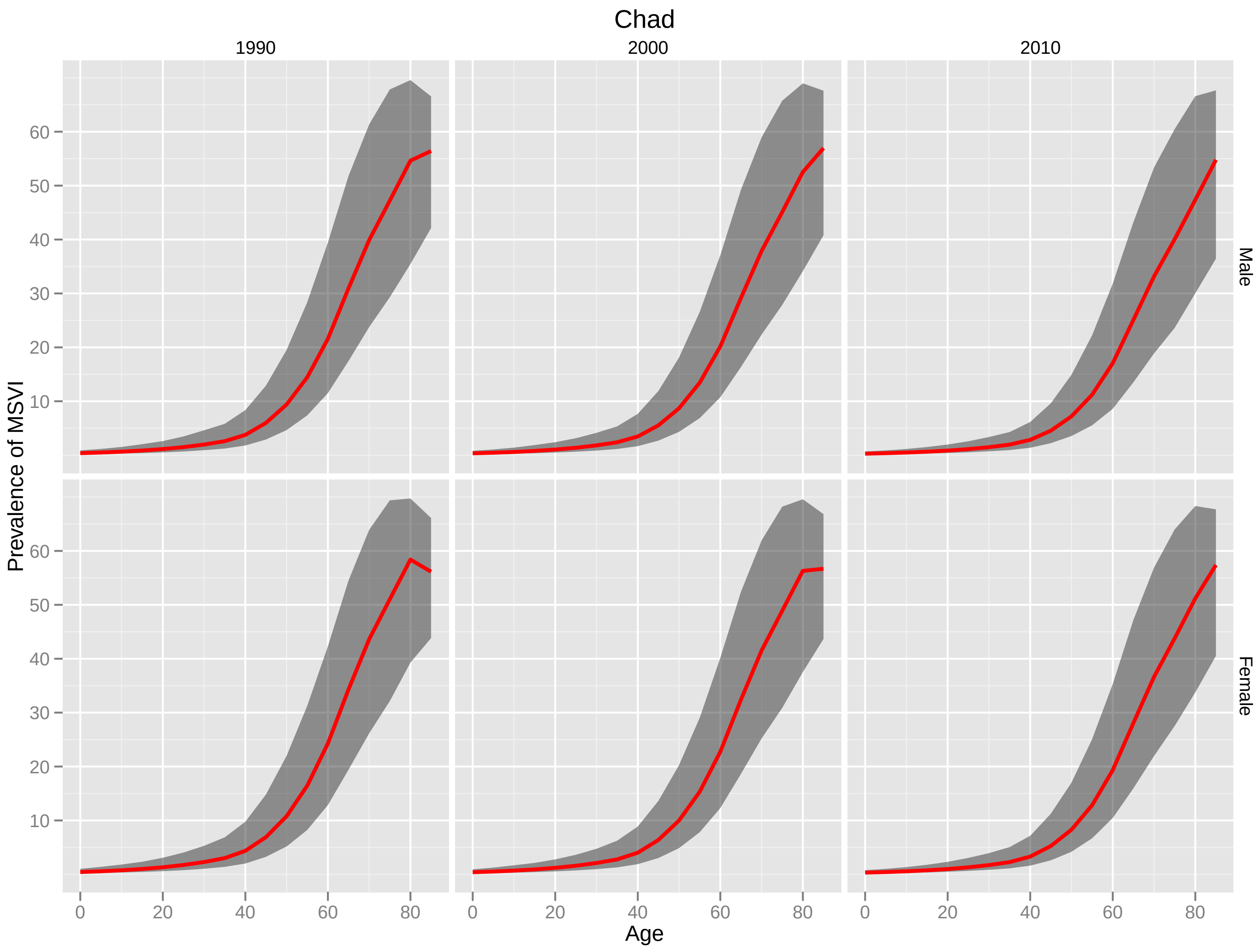
Chad
1990
2000
2010
Male
Female
Prevalence of MSVI
Age
10
20
30
40
50
60
0
20
40
60
80

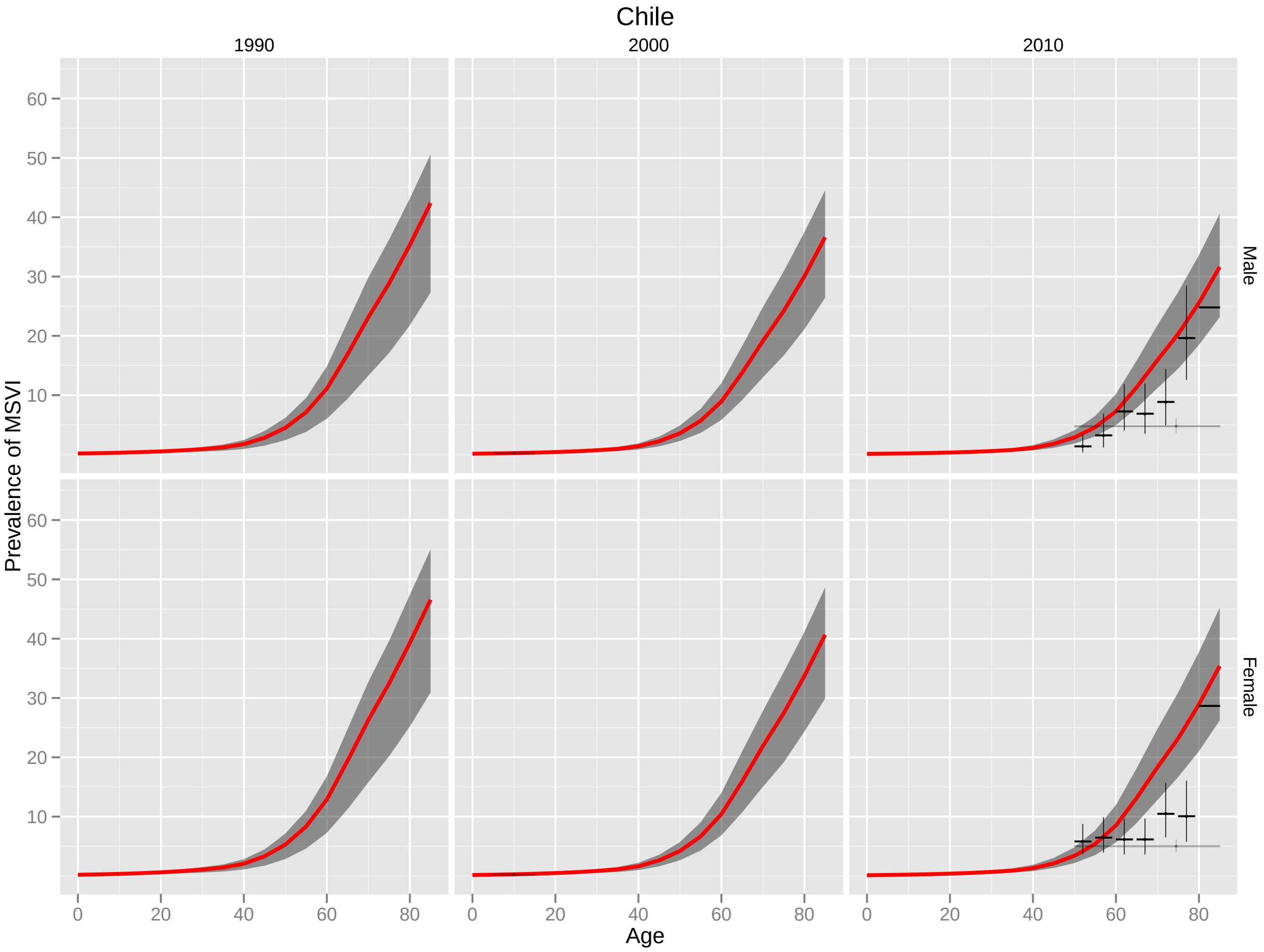
Chile
1990
2000
2010
Male
Female
Prevalence of MSVI
Age
0
20
40
60
80
10
20
30
40
50
60

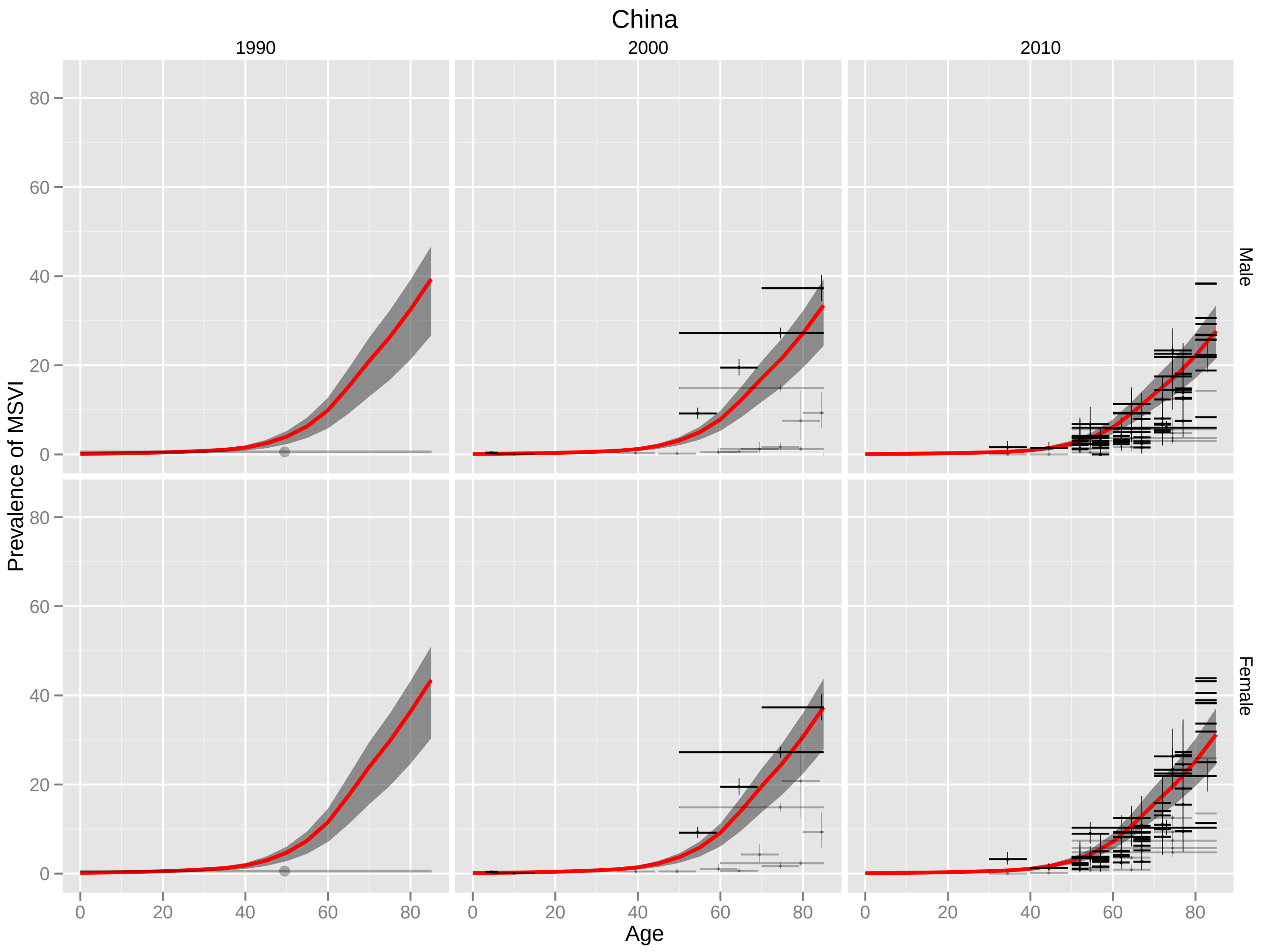
China
1990
2000
2010
Male
Female
Prevalence of MSVI
Age
0
20
40
60
80

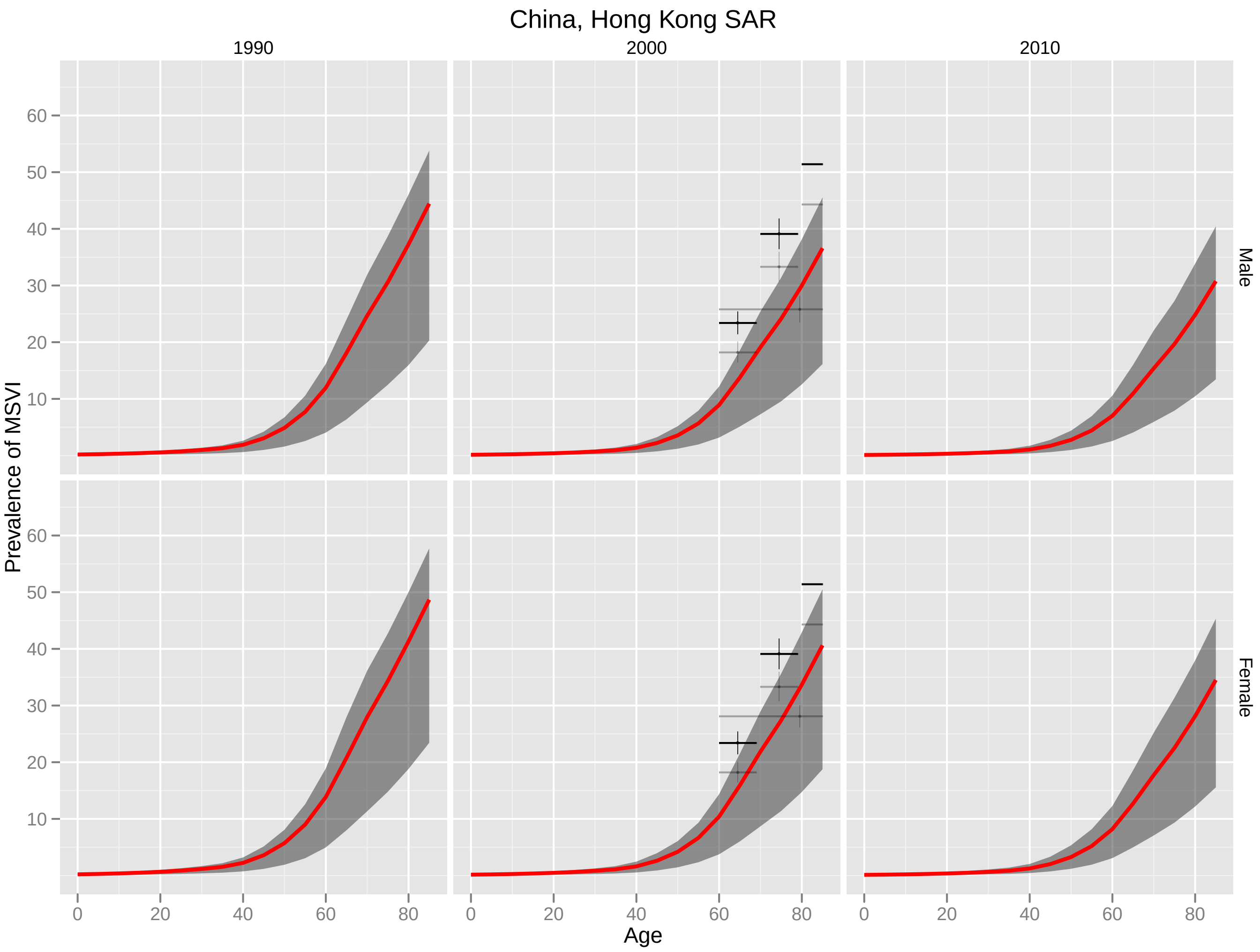
China, Hong Kong SAR
1990
2000
2010
Male
Female
Prevalence of MSVI
Age
0
20
40
60
80
10
20
30
40
50
60

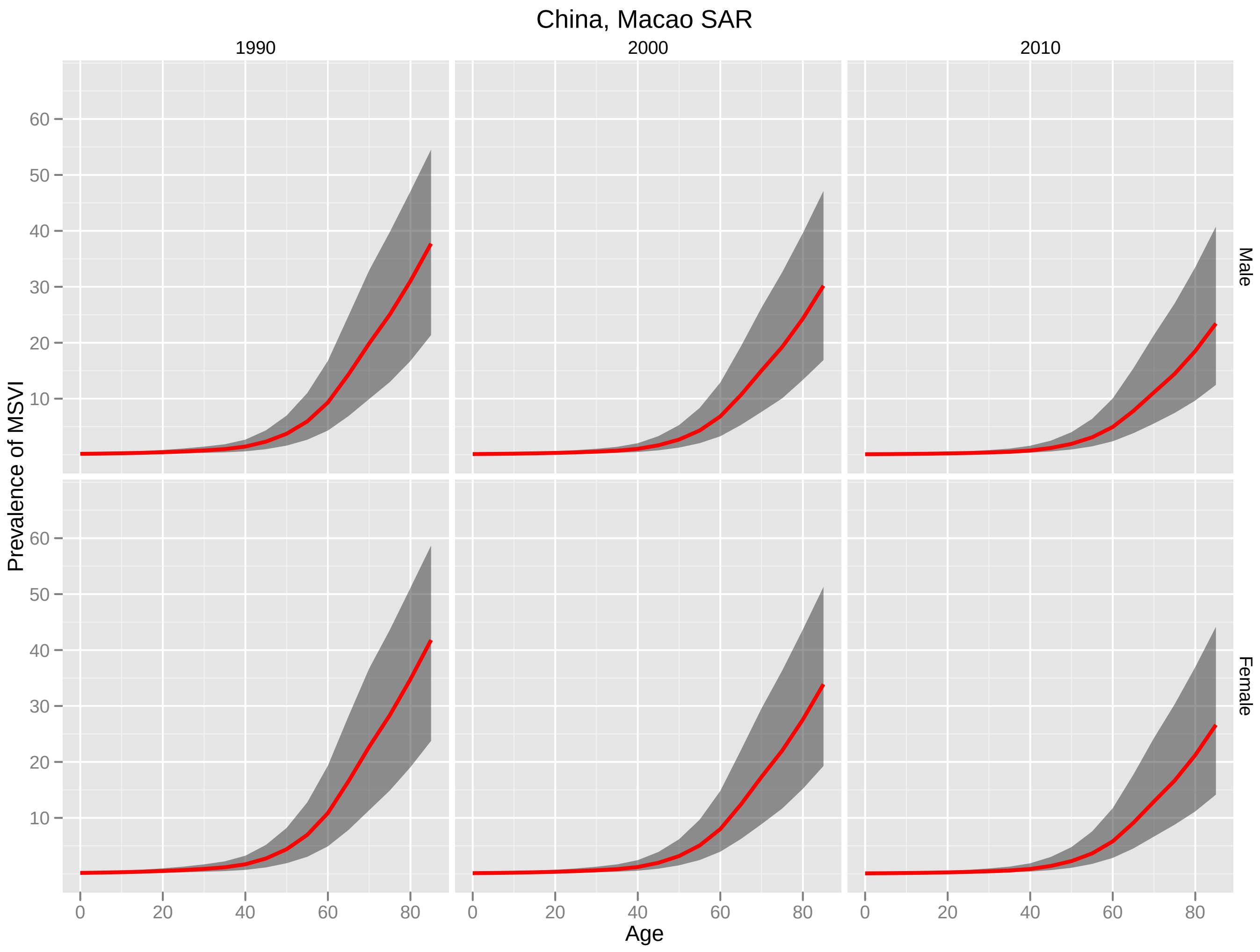
China, Macao SAR
1990
2000
2010
Male
Female
Prevalence of MSVI
Age
10
20
30
40
50
60
0
20
40
60
80

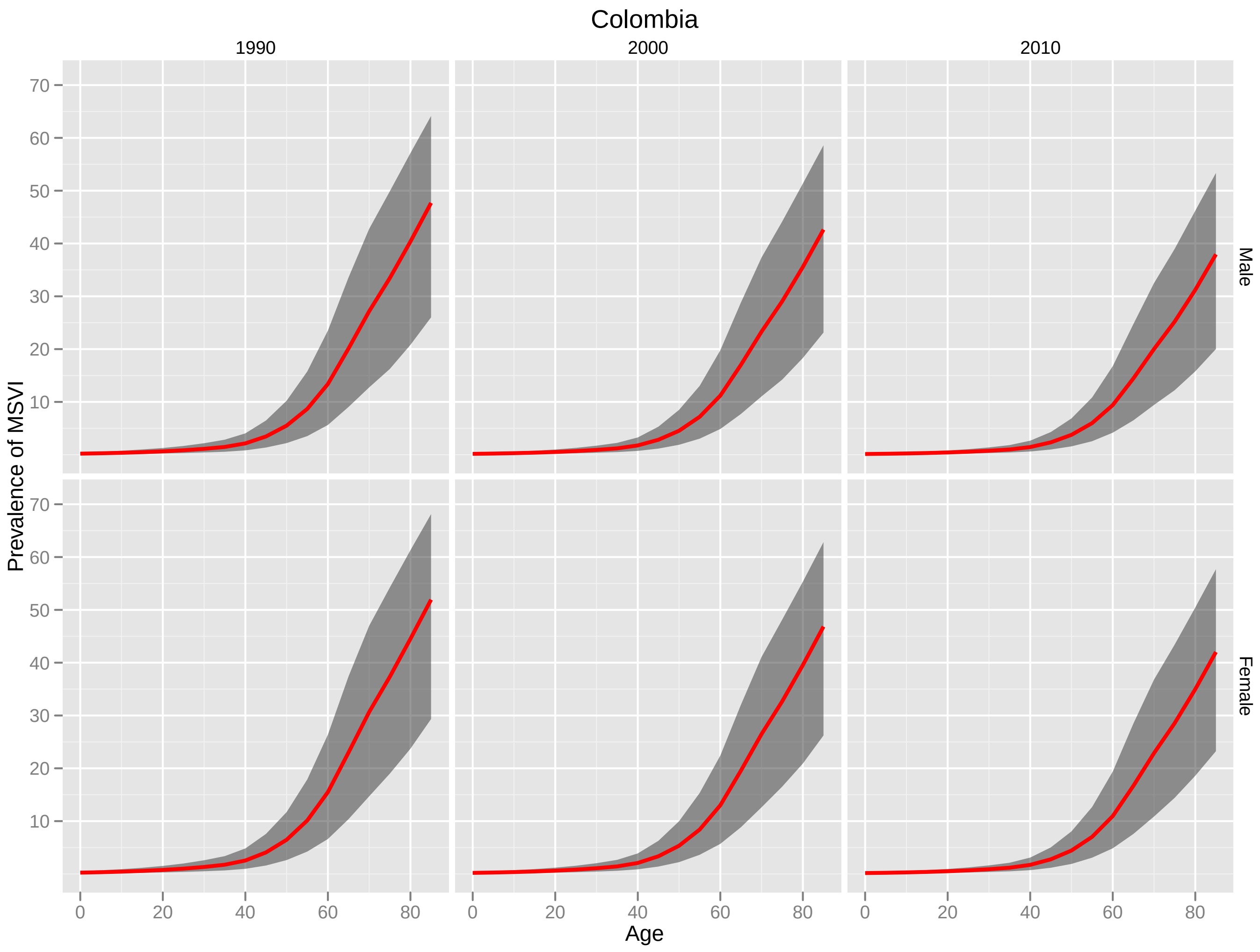
Colombia
1990
2000
2010
Male
Female
Prevalence of MSVI
Age
10
20
30
40
50
60
70
0
20
40
60
80

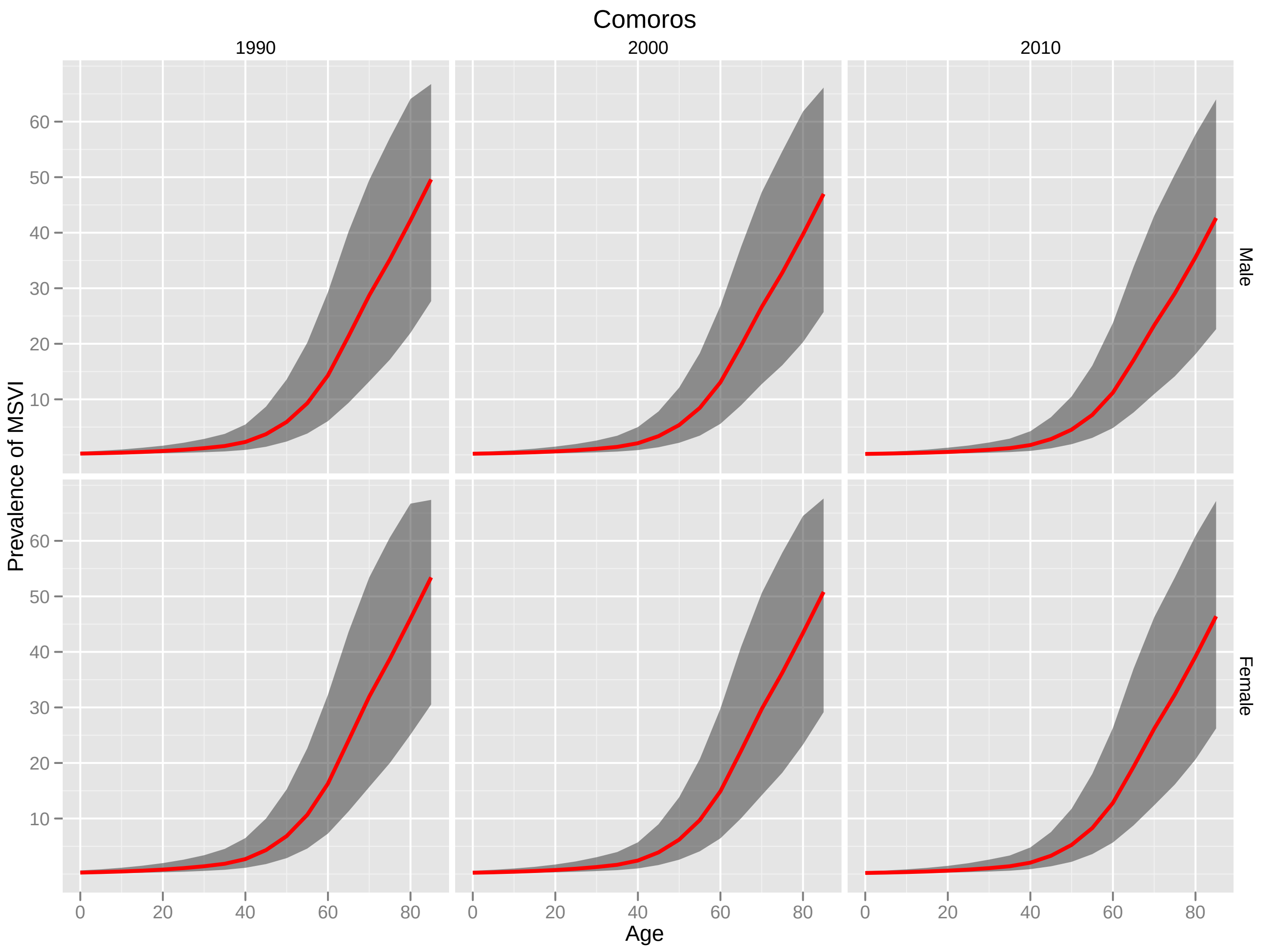

Comoros
1990
2000
2010
Male
Female
Prevalence of MSVI
Age

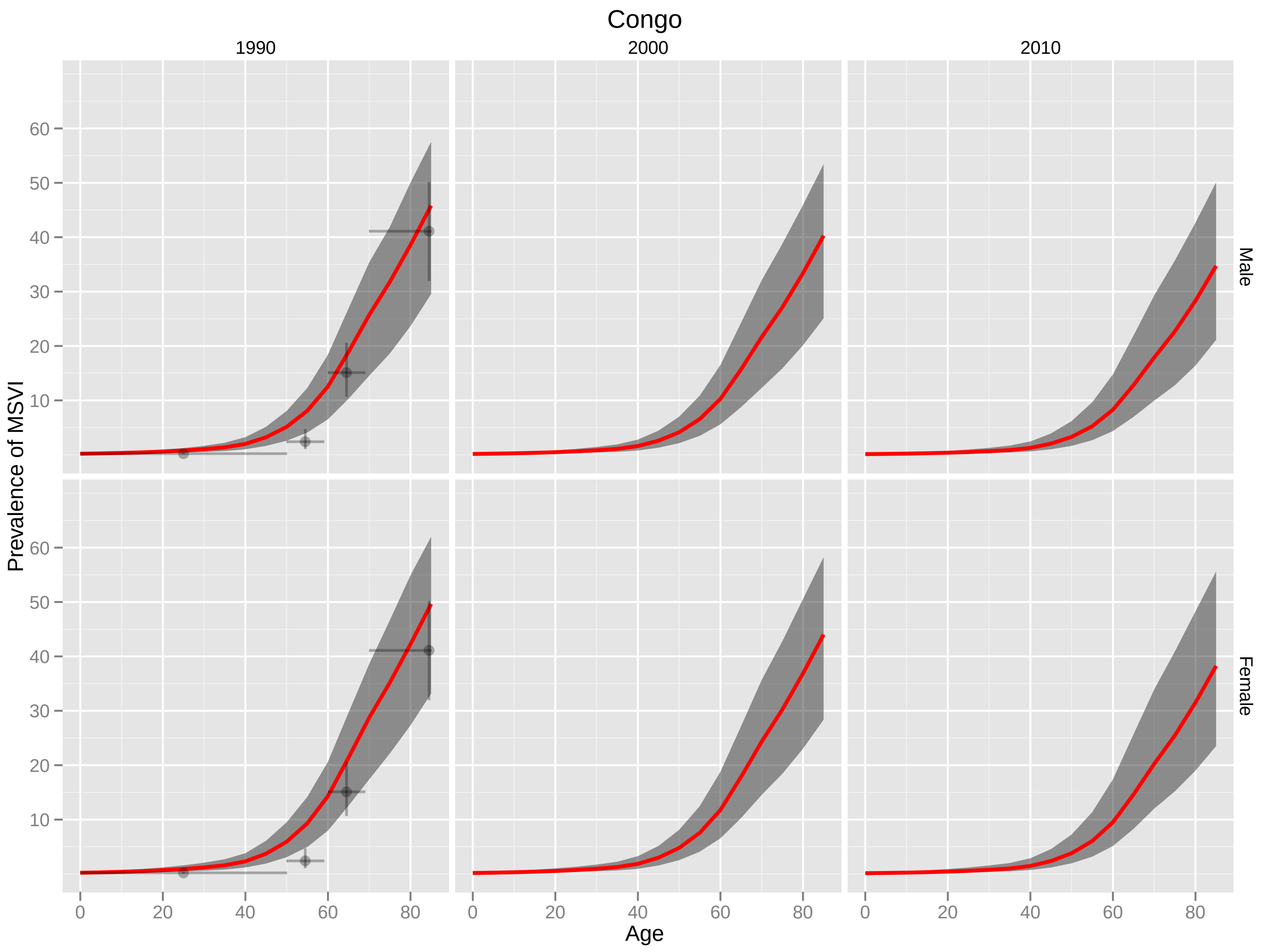

Congo
1990
2000
2010
Male
Female
Prevalence of MSVI
Age
10
20
30
40
50
60
0
20
40
60
80

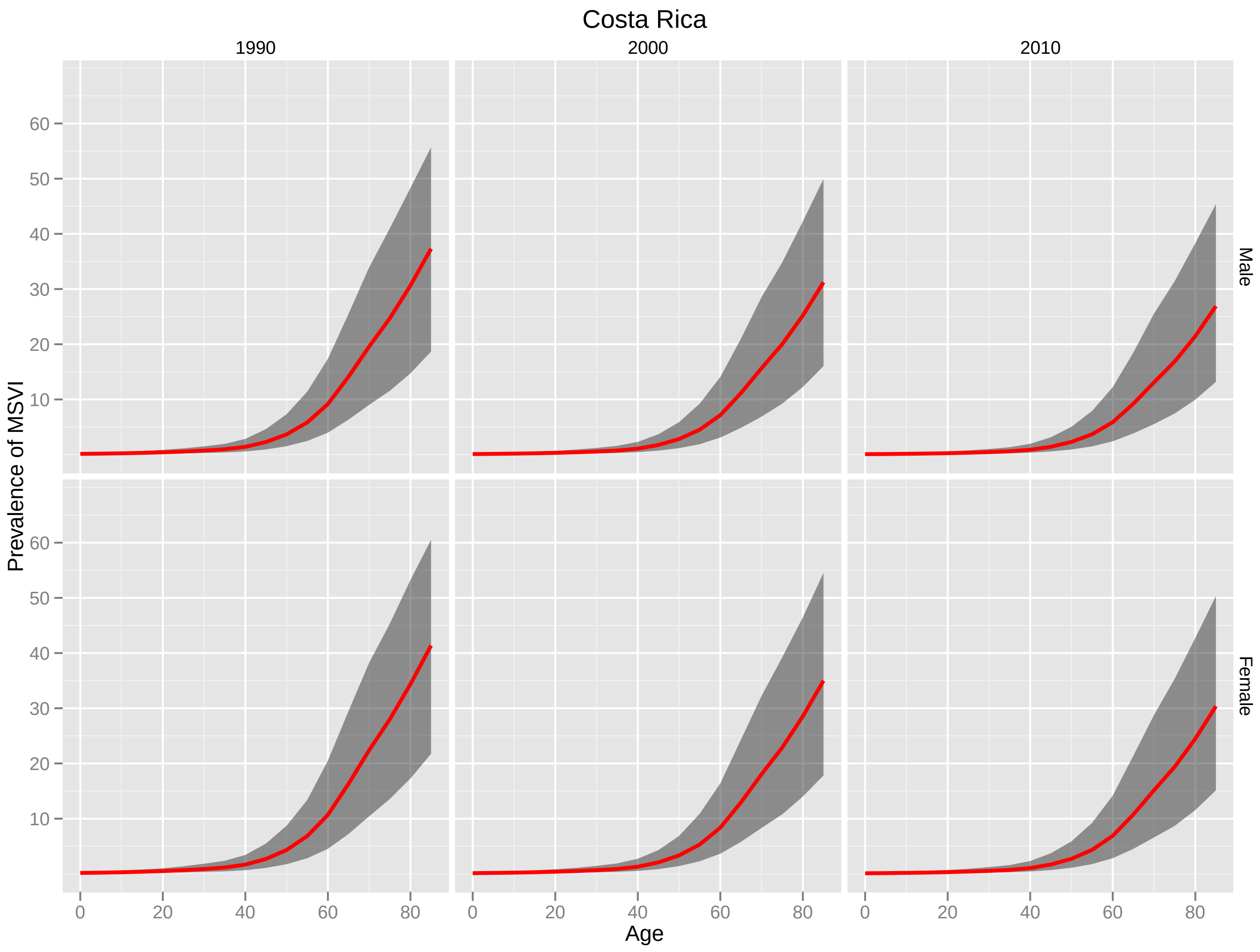
Costa Rica
1990
2000
2010
Male
Female
Prevalence of MSVI
Age
10
20
30
40
50
60
0
20
40
60
80

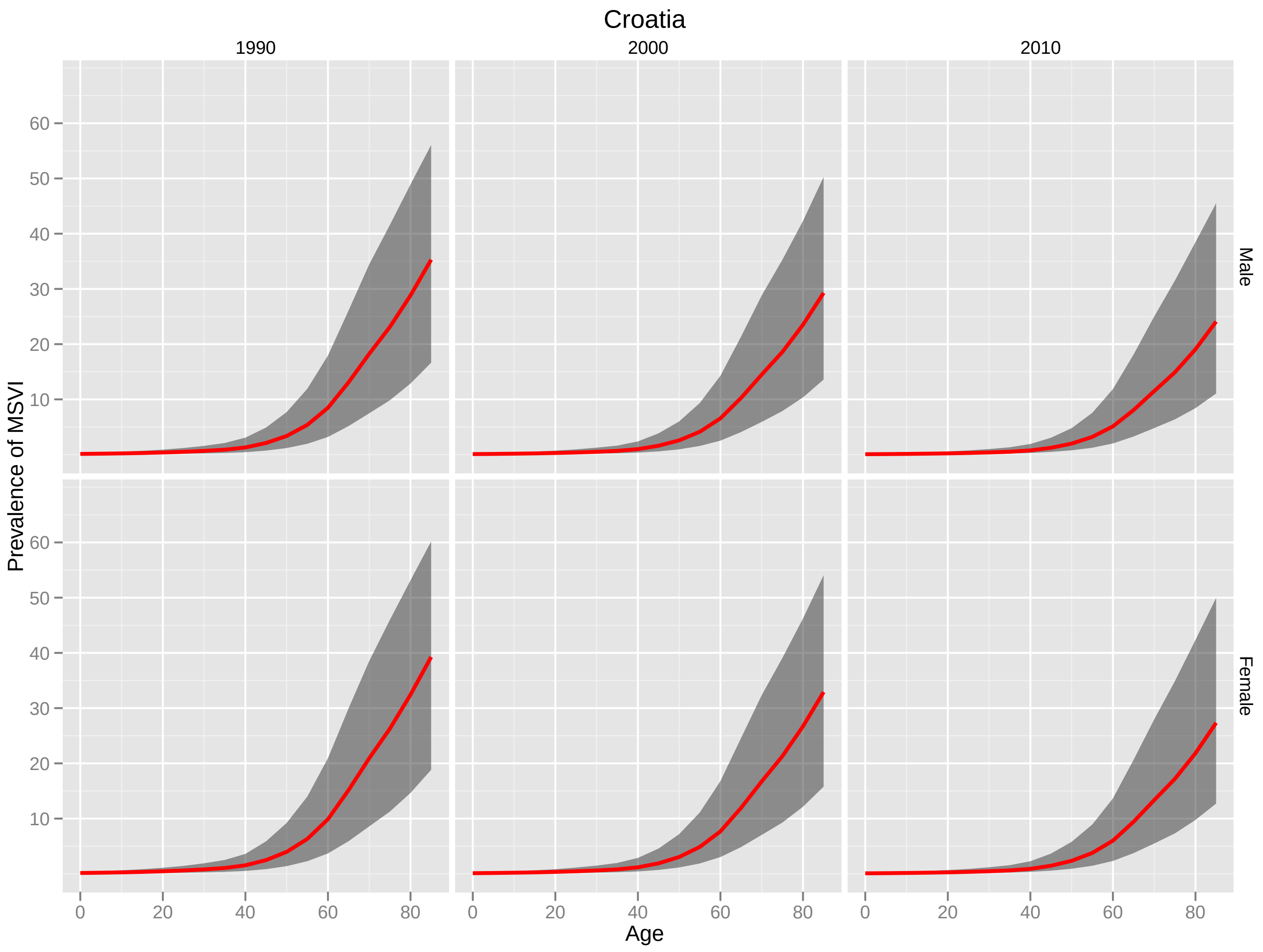
Croatia
1990
2000
2010
Male
Female
Prevalence of MSVI
10
20
30
40
50
60
0
20
40
60
80
Age

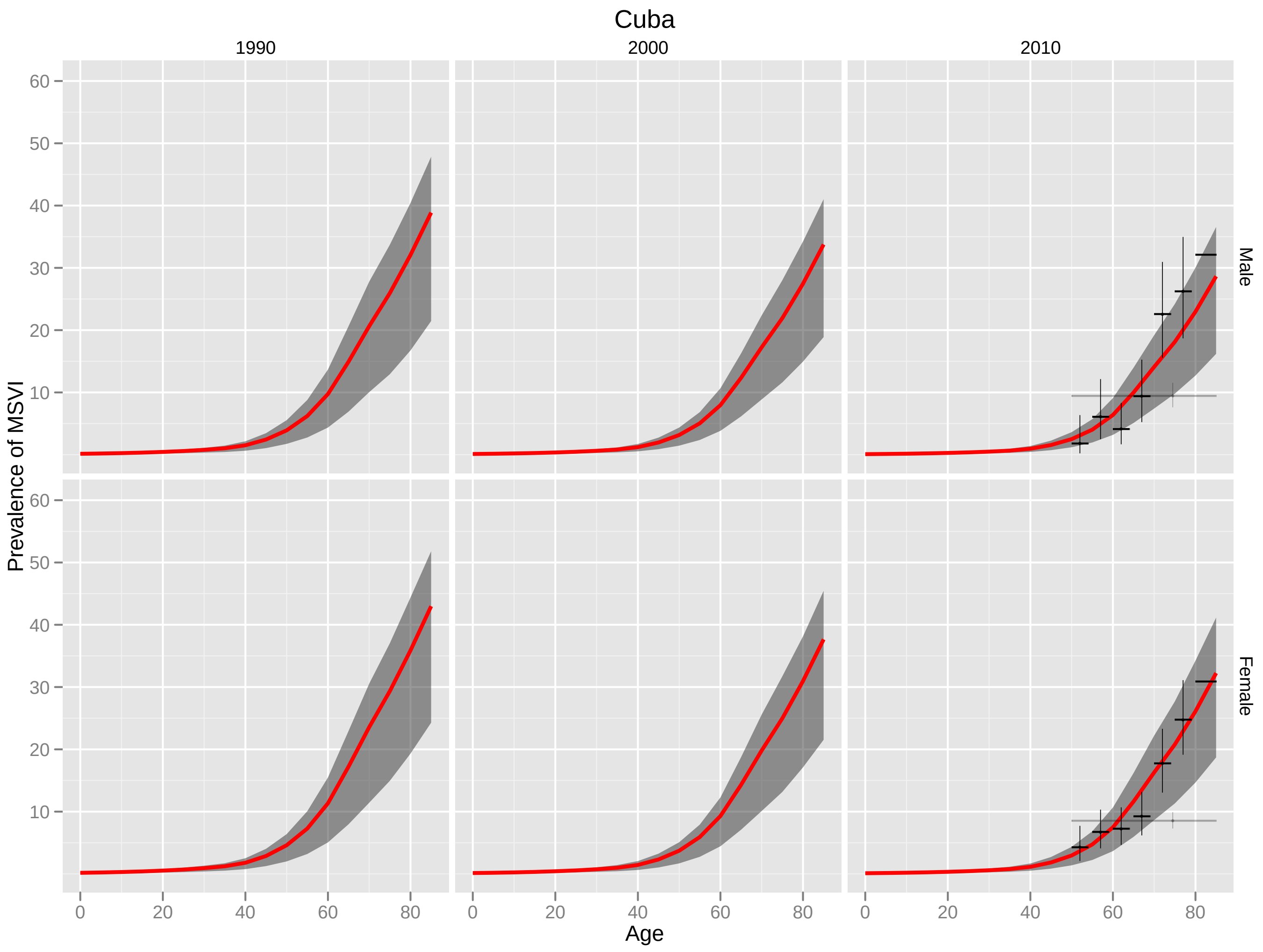
Cuba
1990
2000
2010
Male
Female
Prevalence of MSVI
Age
10
20
30
40
50
60
0
20
40
60
80

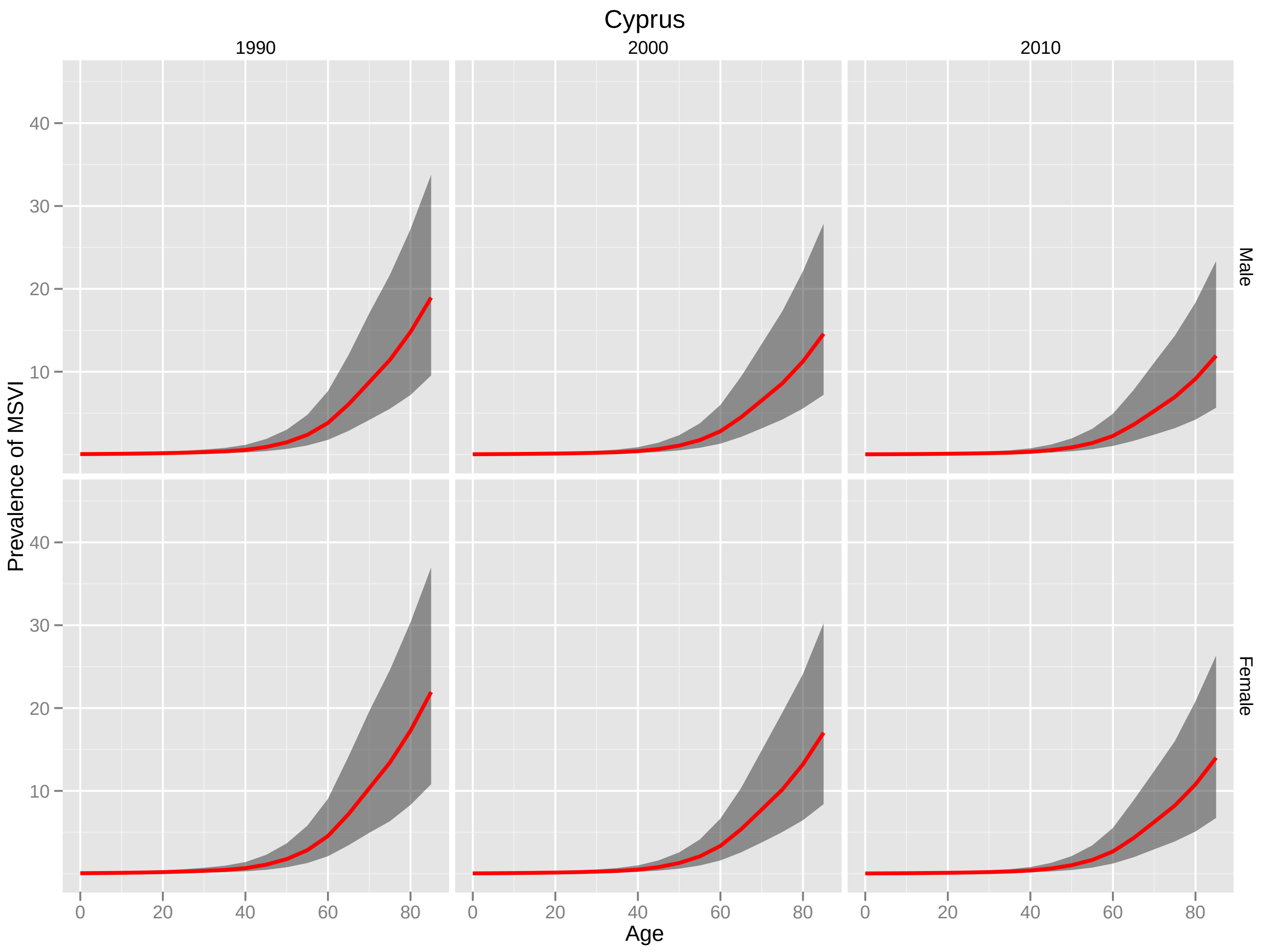
Cyprus
1990
2000
2010
Male
Female
Prevalence of MSVI
Age
10
20
30
40
0
20
40
60
80

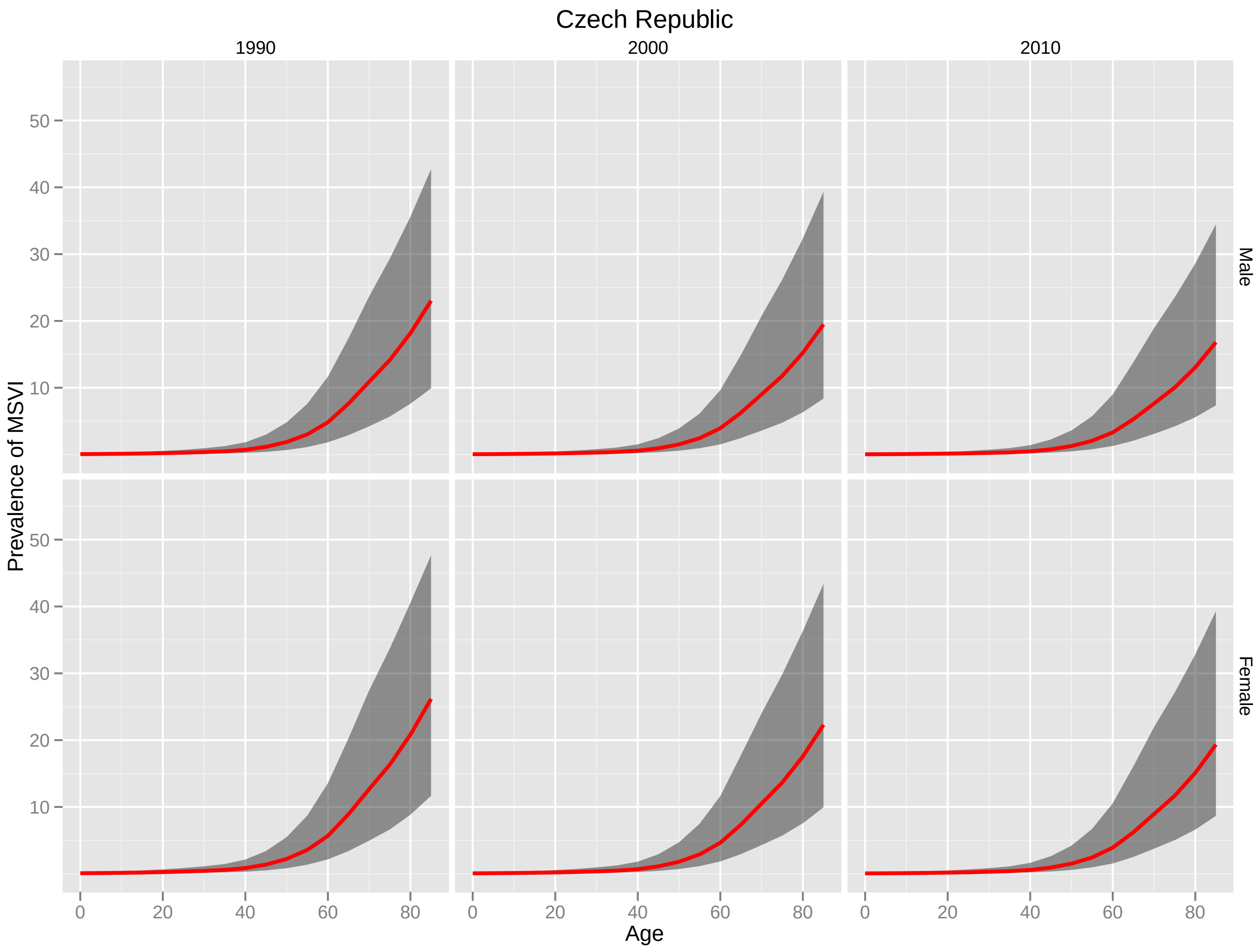

Czech Republic
1990
2000
2010
Male
Female
Prevalence of MSVI
Age
10
20
30
40
50
0
20
40
60
80

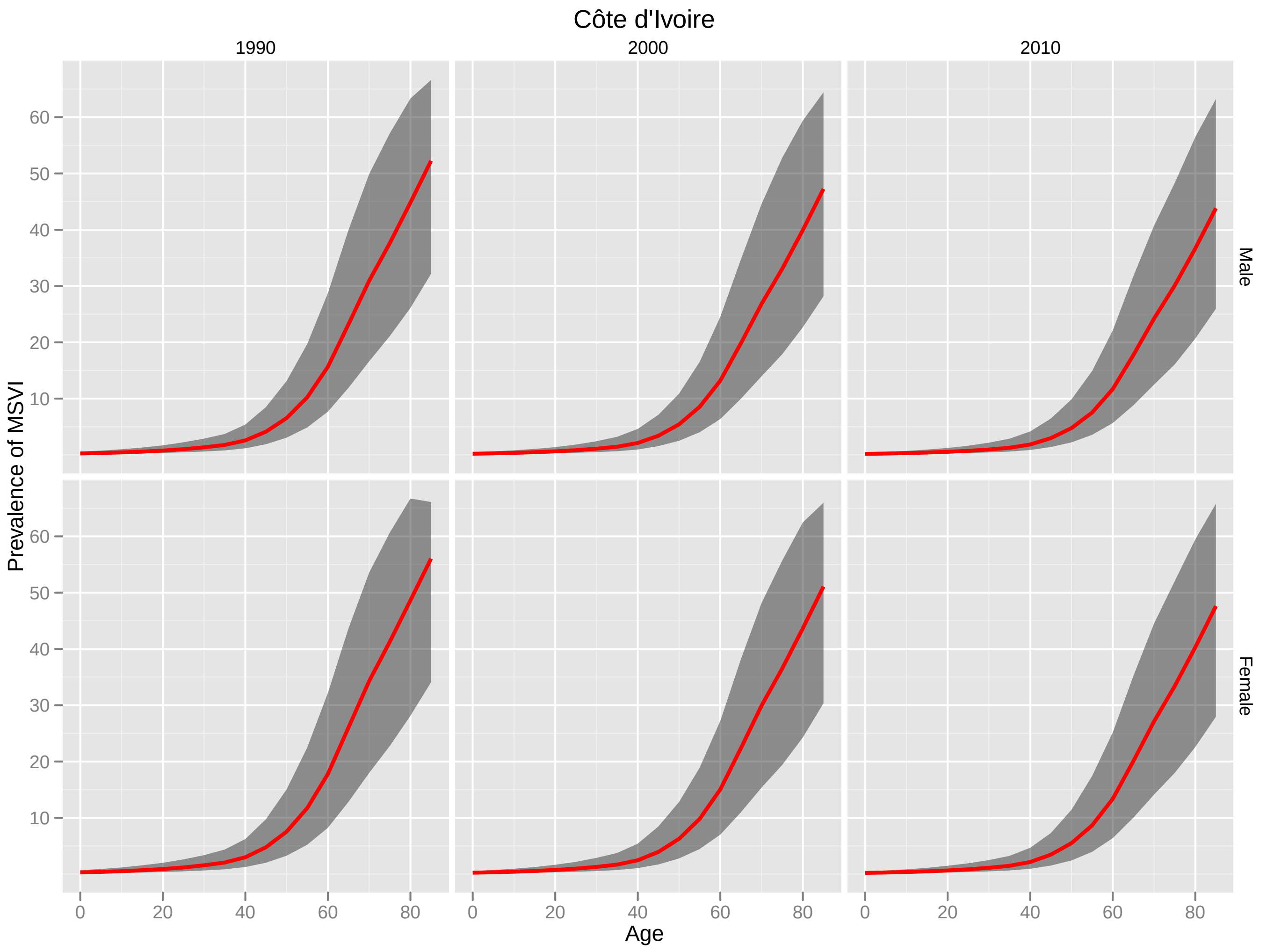
Côte d'Ivoire
1990
2000
2010
Male
Female
Prevalence of MSVI
Age
0
20
40
60
80
10
20
30
40
50
60

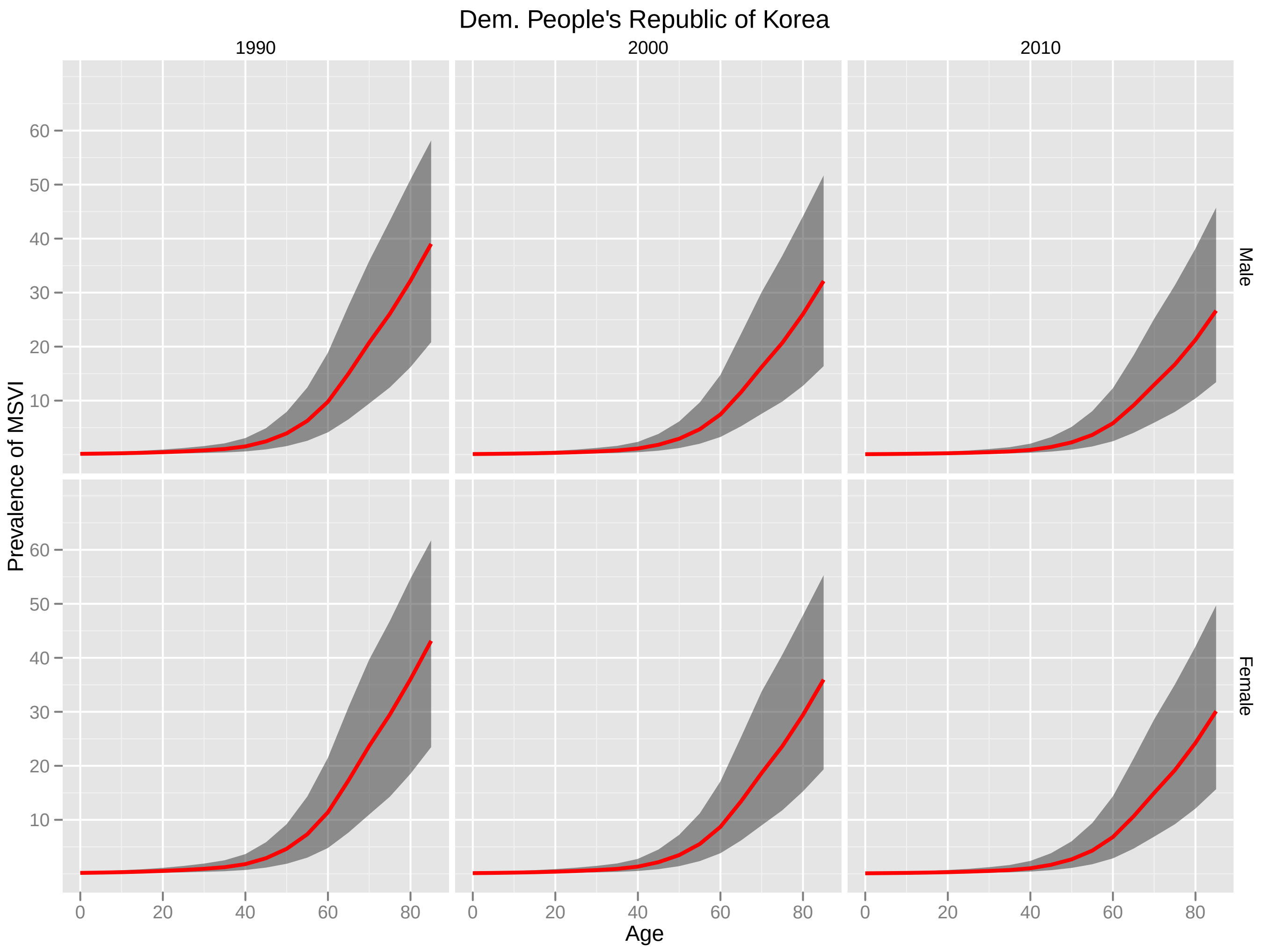
Dem. People's Republic of Korea
1990
2000
2010
Male
Female
Prevalence of MSVI
Age
10
20
30
40
50
60
0
20
40
60
80

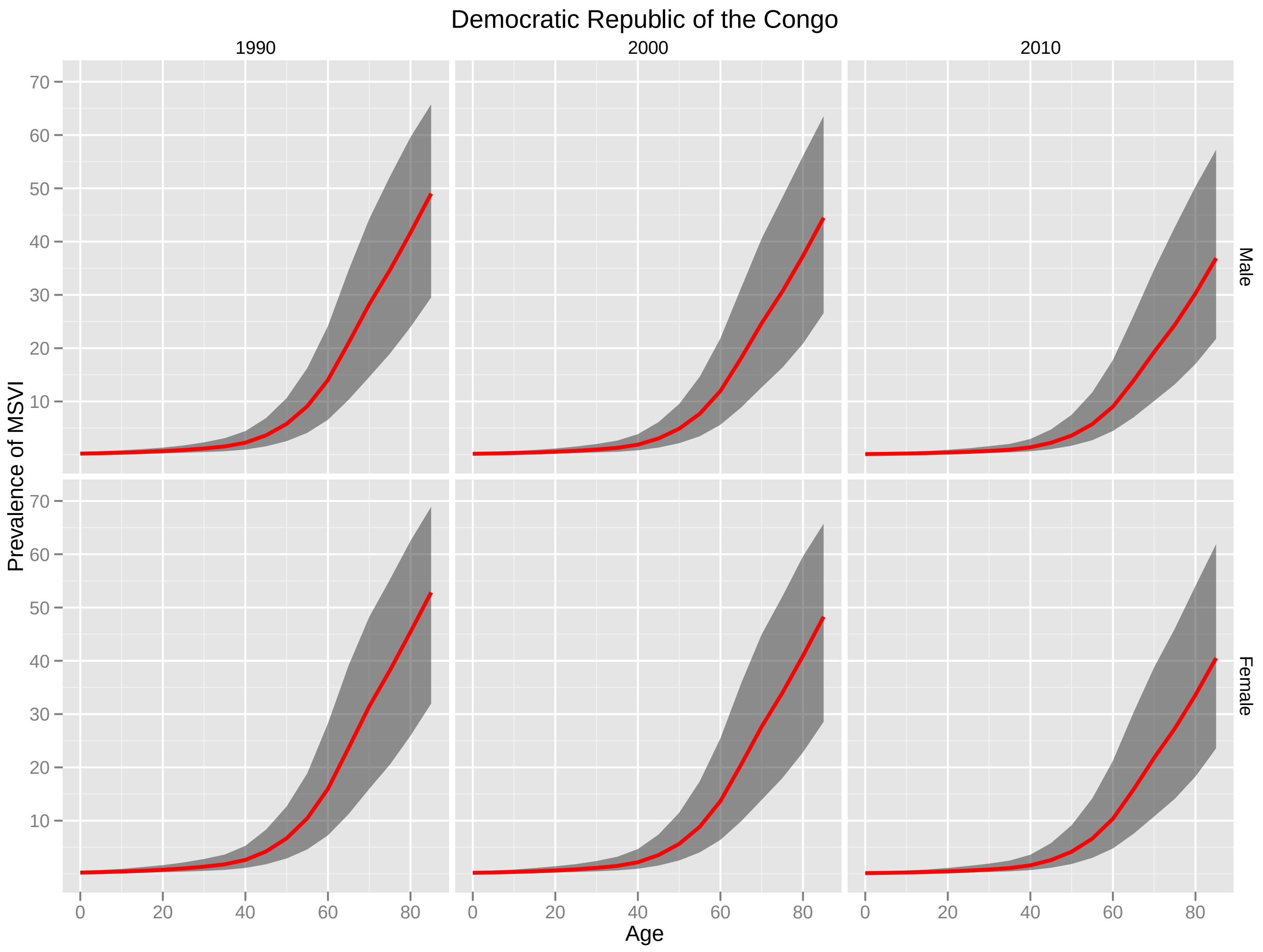
Democratic Republic of the Congo
1990
2000
2010
Male
Female
Prevalence of MSVI
Age
10
20
30
40
50
60
70
0
20
40
60
80

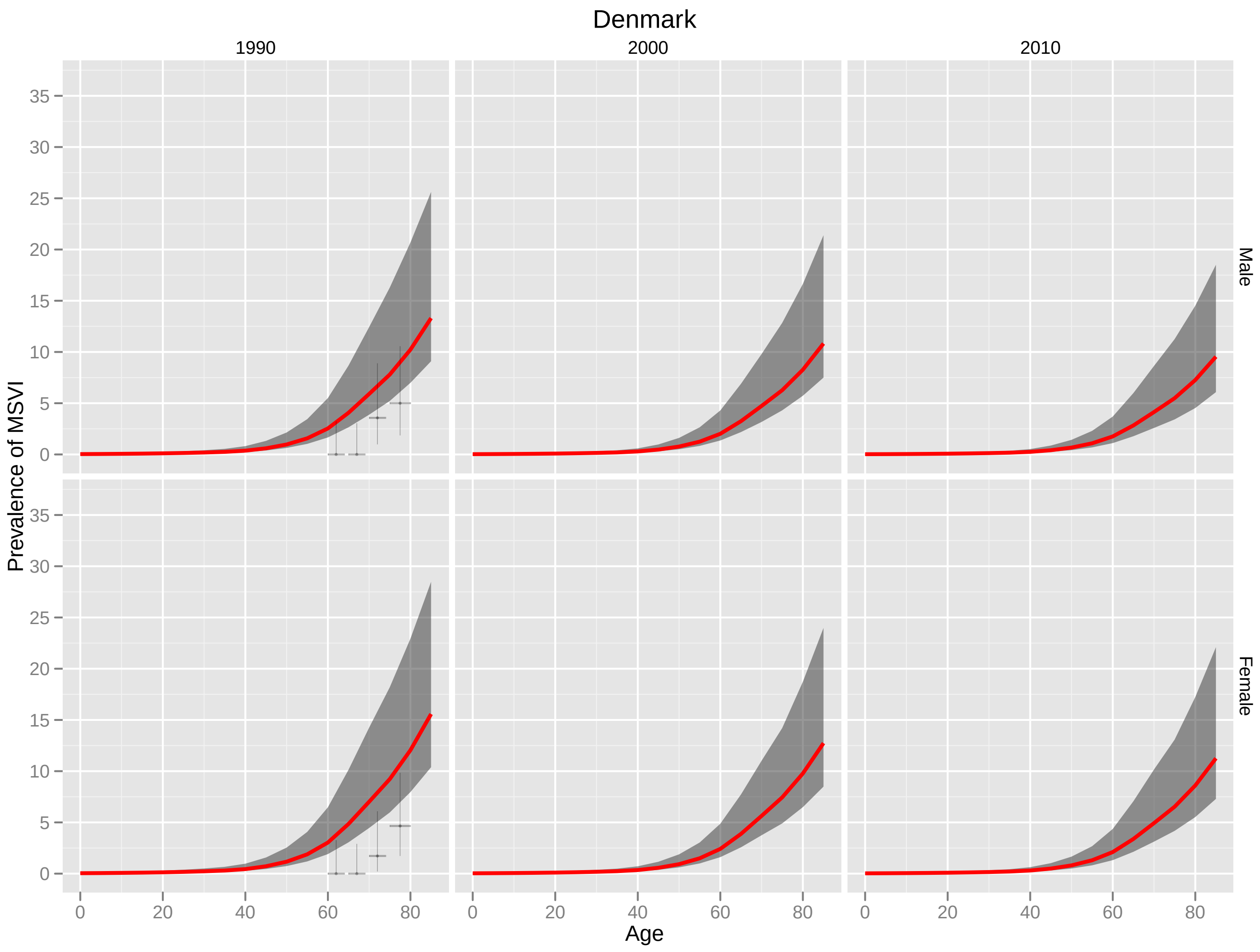
Denmark
1990
2000
2010
Male
Female
Prevalence of MSVI
Age
0
5
10
15
20
25
30
35
0
20
40
60
80

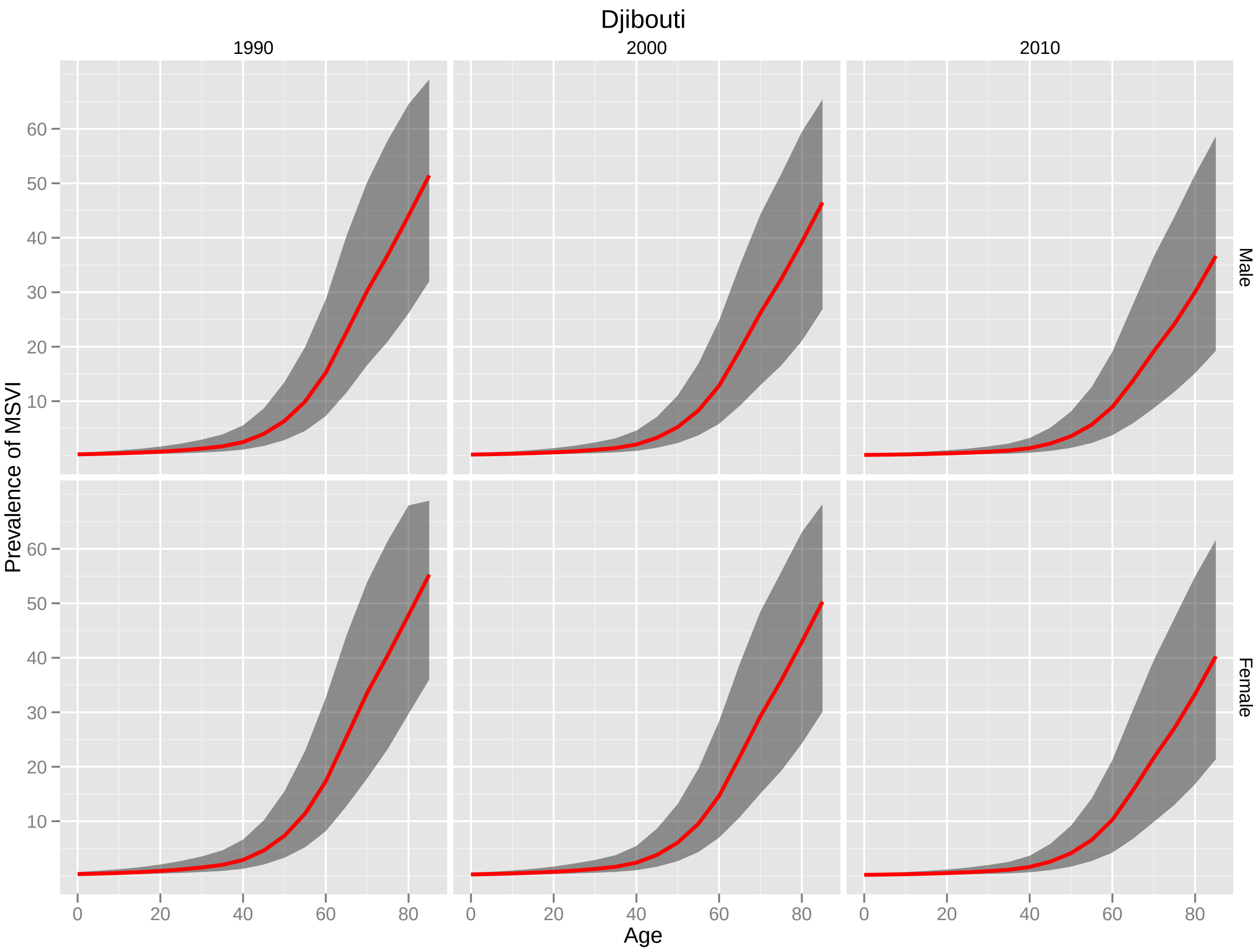
Djibouti
1990
2000
2010
Prevalence of MSVI
Age
Male
Female
0
20
40
60
80
10
20
30
40
50
60

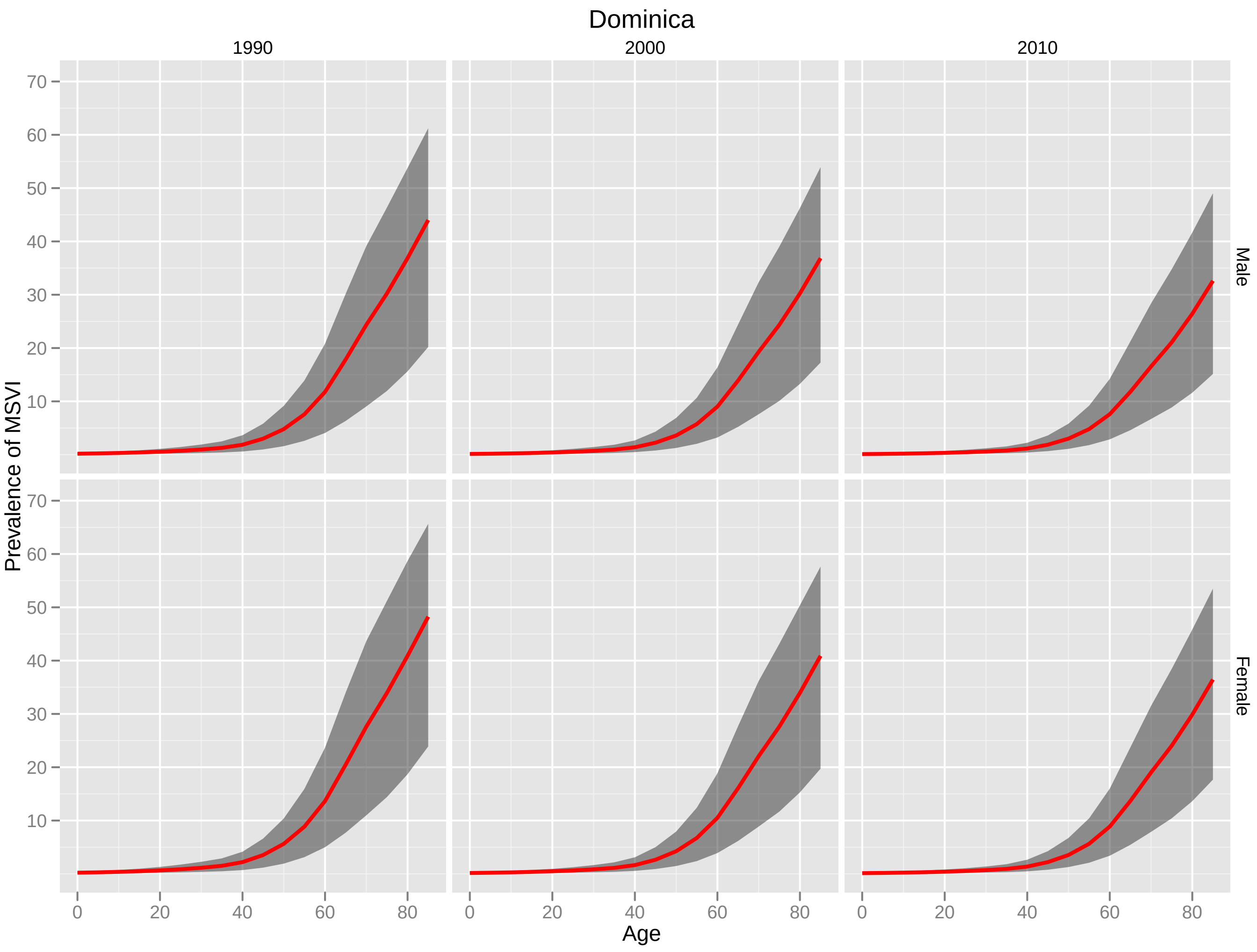
Dominica
1990
2000
2010
Male
Female
Prevalence of MSVI
Age
10
20
30
40
50
60
70
0
20
40
60
80

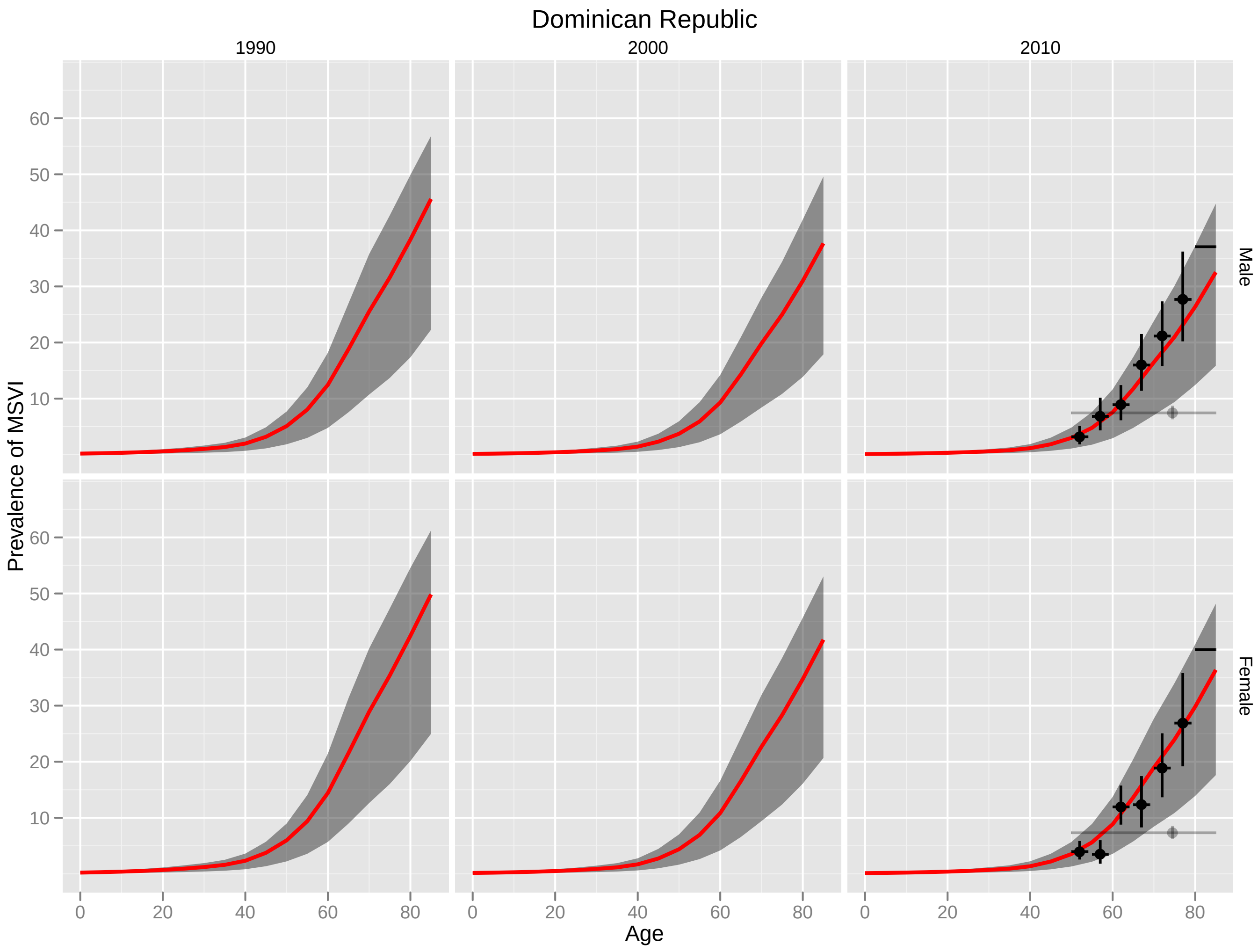
Dominican Republic
1990
2000
2010
Male
Female
Prevalence of MSVI
Age

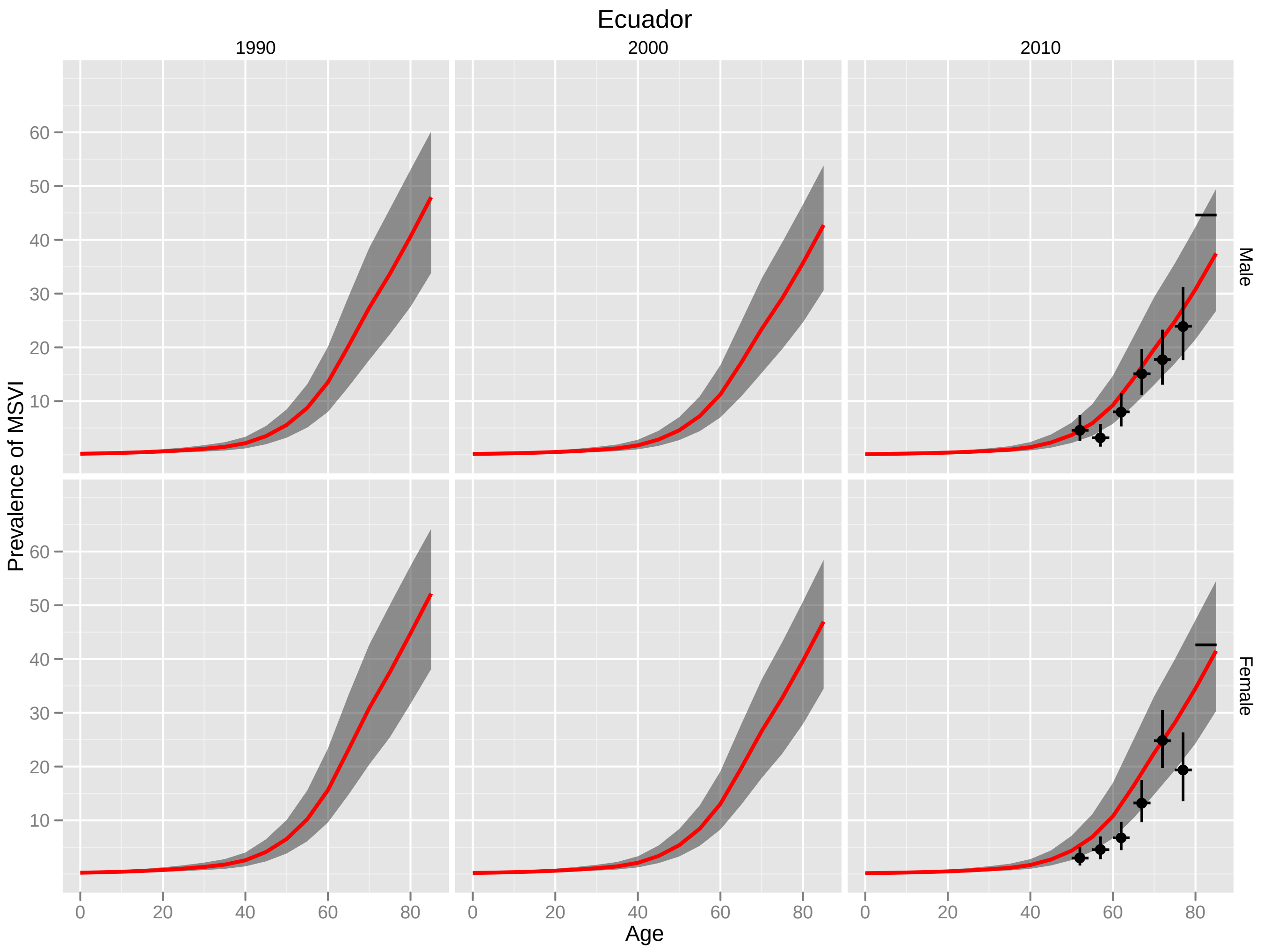

Ecuador
1990
2000
2010
Male
Female
Prevalence of MSVI
Age
10
20
30
40
50
60
0
20
40
60
80

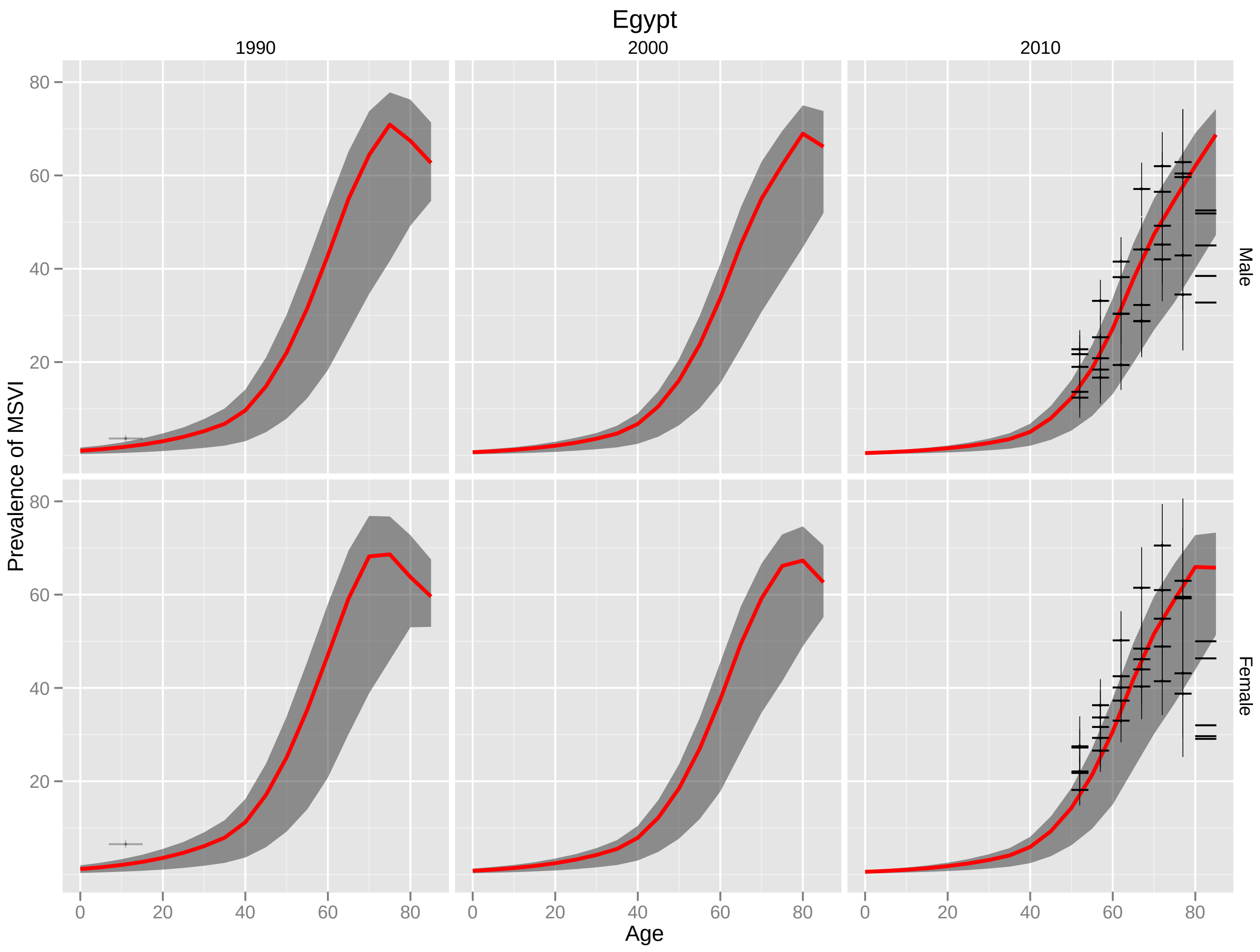
Egypt
1990
2000
2010
Male
Female
Prevalence of MSVI
Age
0
20
40
60
80

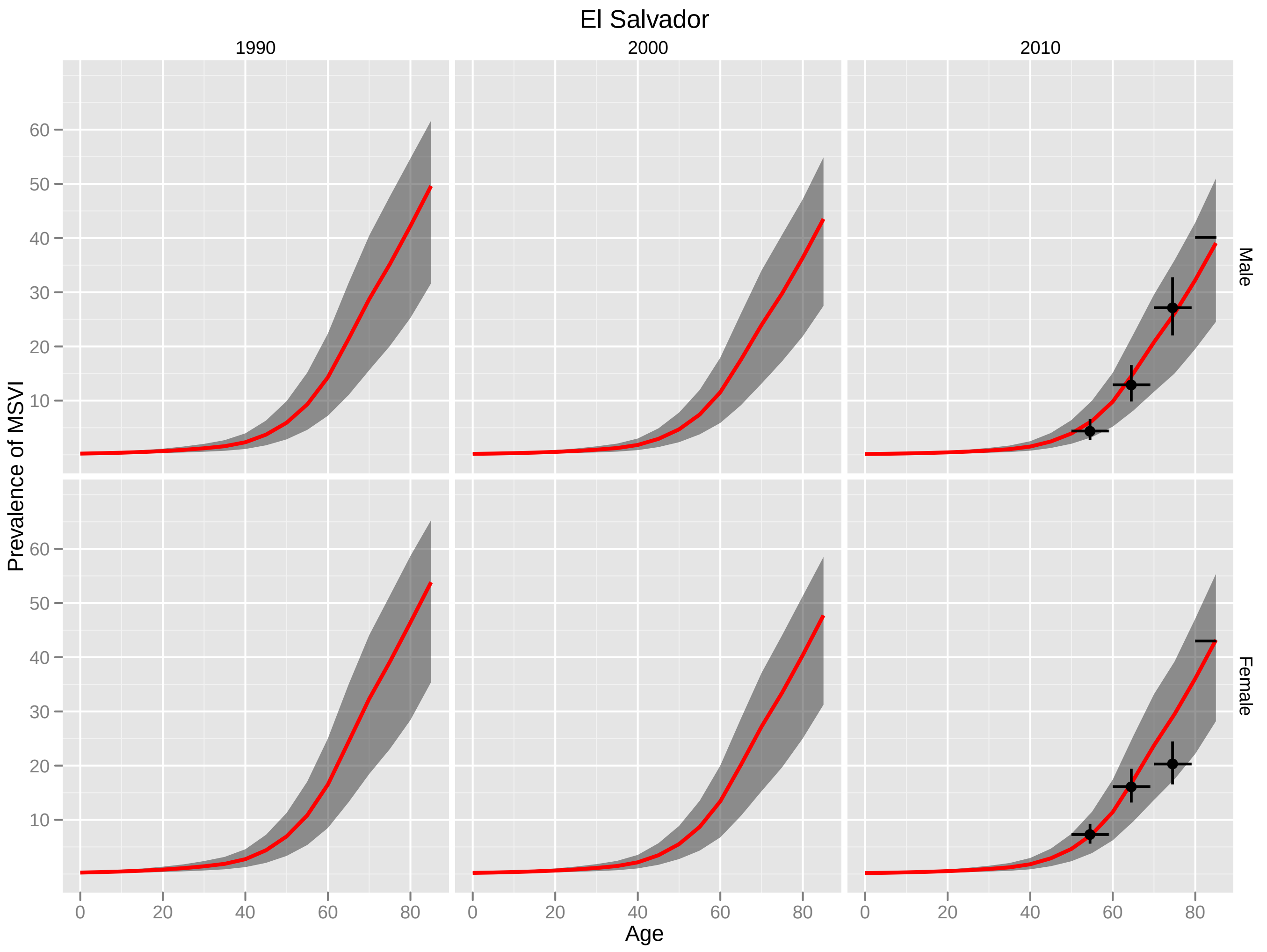
El Salvador
1990
2000
2010
Male
Female
Prevalence of MSVI
Age
10
20
30
40
50
60
0
20
40
60
80

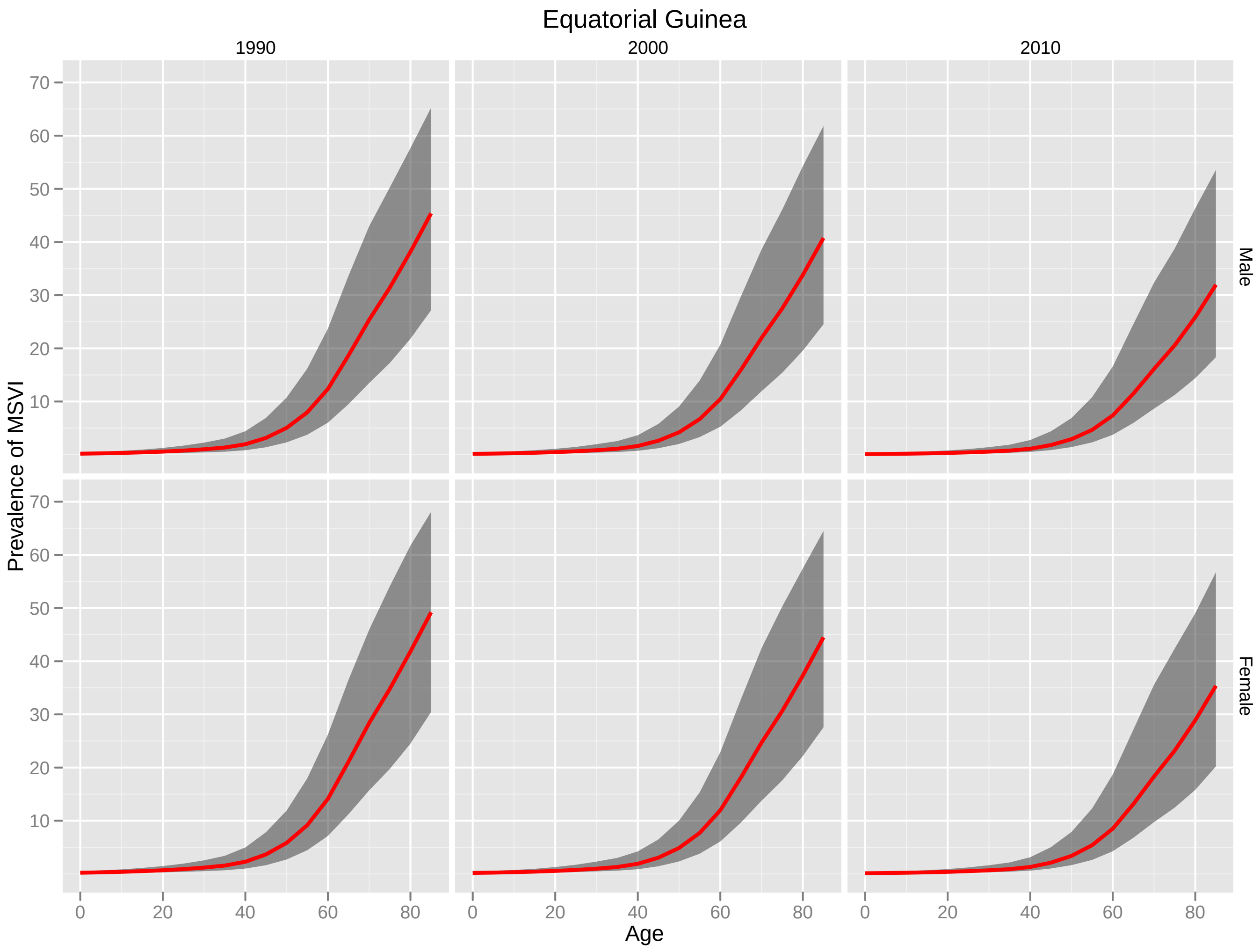
Equatorial Guinea
1990
2000
2010
Male
Female
Prevalence of MSVI
Age
0
20
40
60
80
10
20
30
40
50
60
70

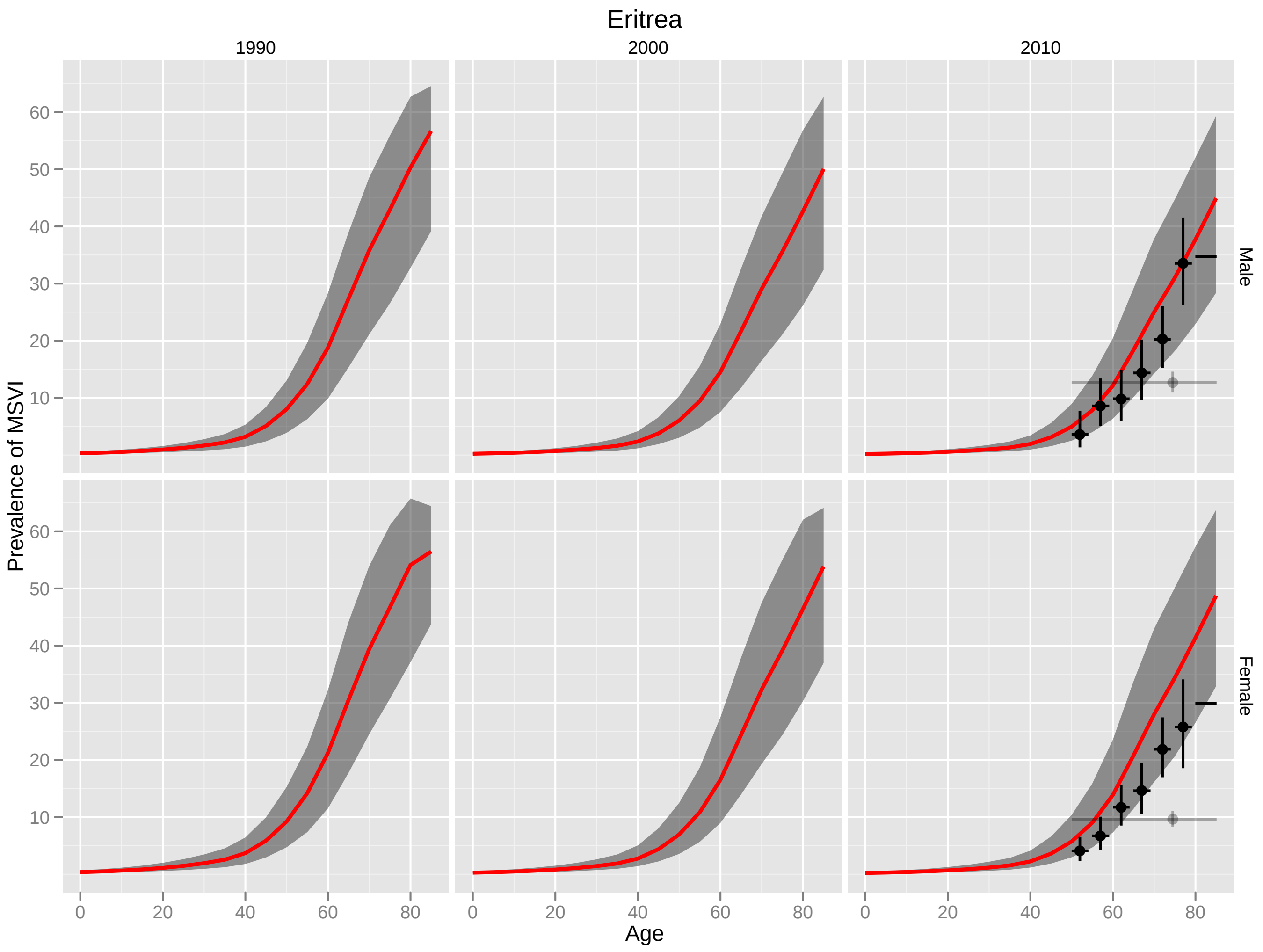

Eritrea
1990
2000
2010
Male
Female
Prevalence of MSVI
Age
0
20
40
60
80
10
20
30
40
50
60

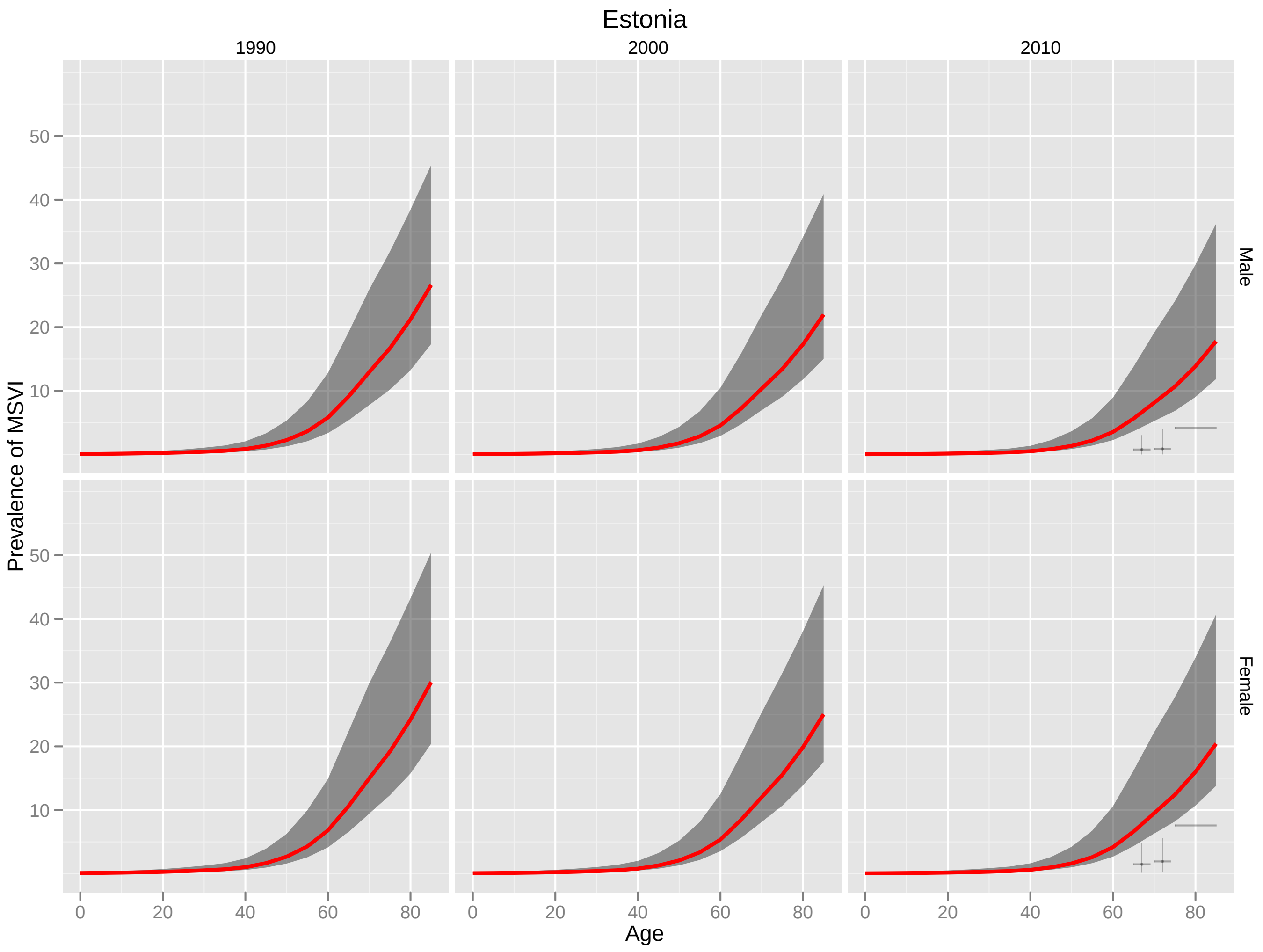
Estonia
1990
2000
2010
Male
Female
Prevalence of MSVI
Age
10
20
30
40
50
0
20
40
60
80

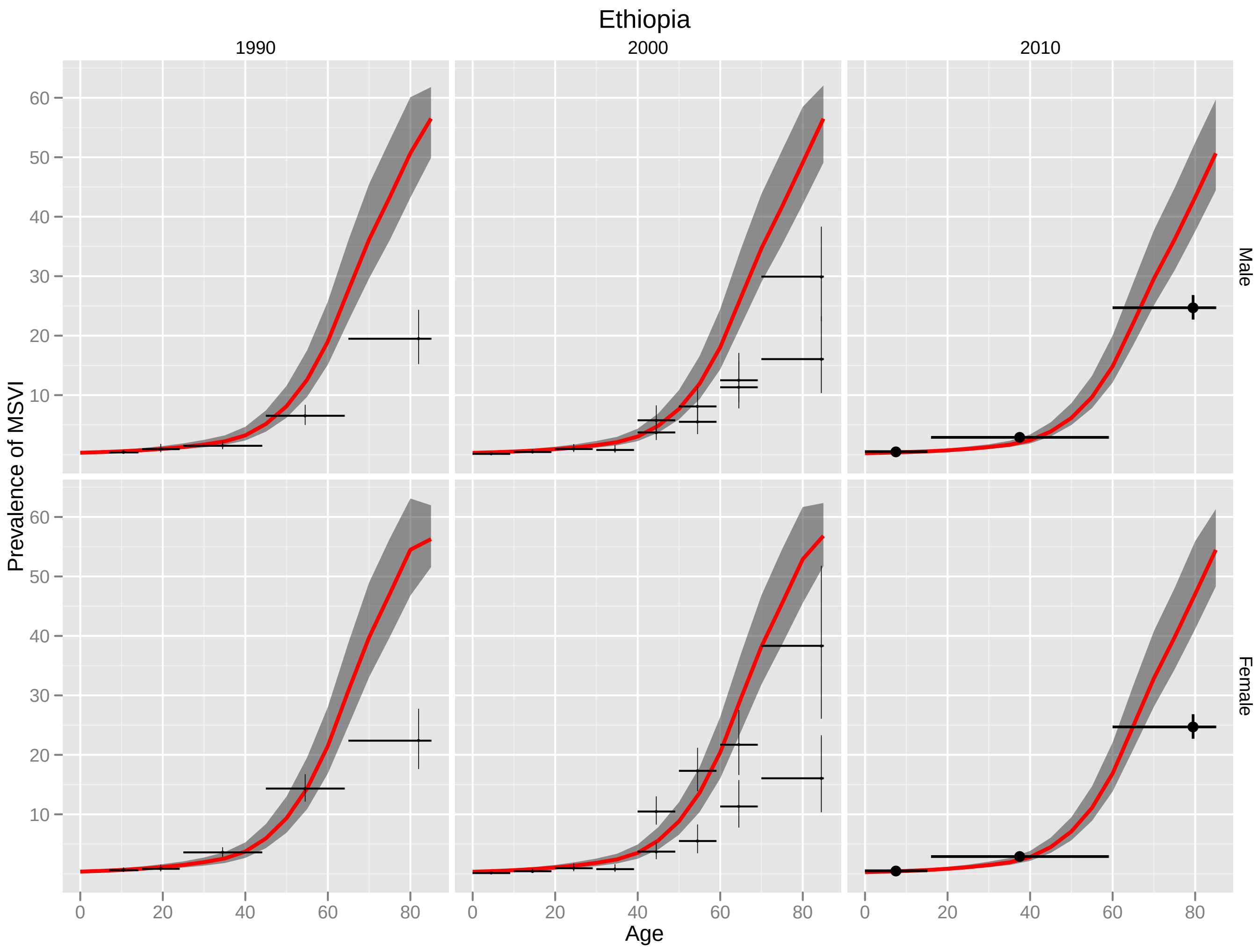
Ethiopia
1990
2000
2010
Male
Female
Prevalence of MSVI
Age
0
20
40
60
80
10
20
30
40
50
60

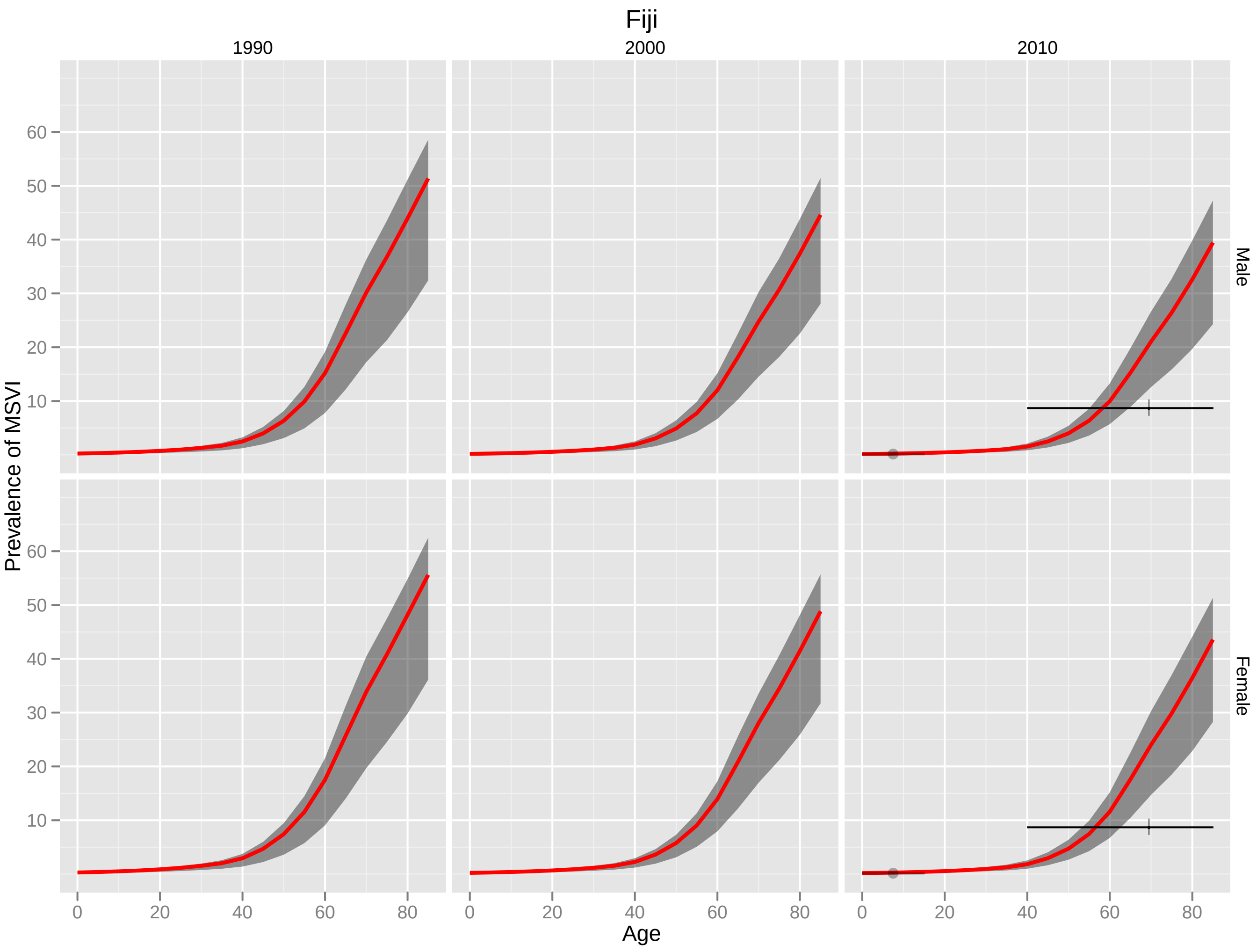

Fiji
1990
2000
2010
Male
Female
Prevalence of MSVI
Age
10
20
30
40
50
60
0
20
40
60
80

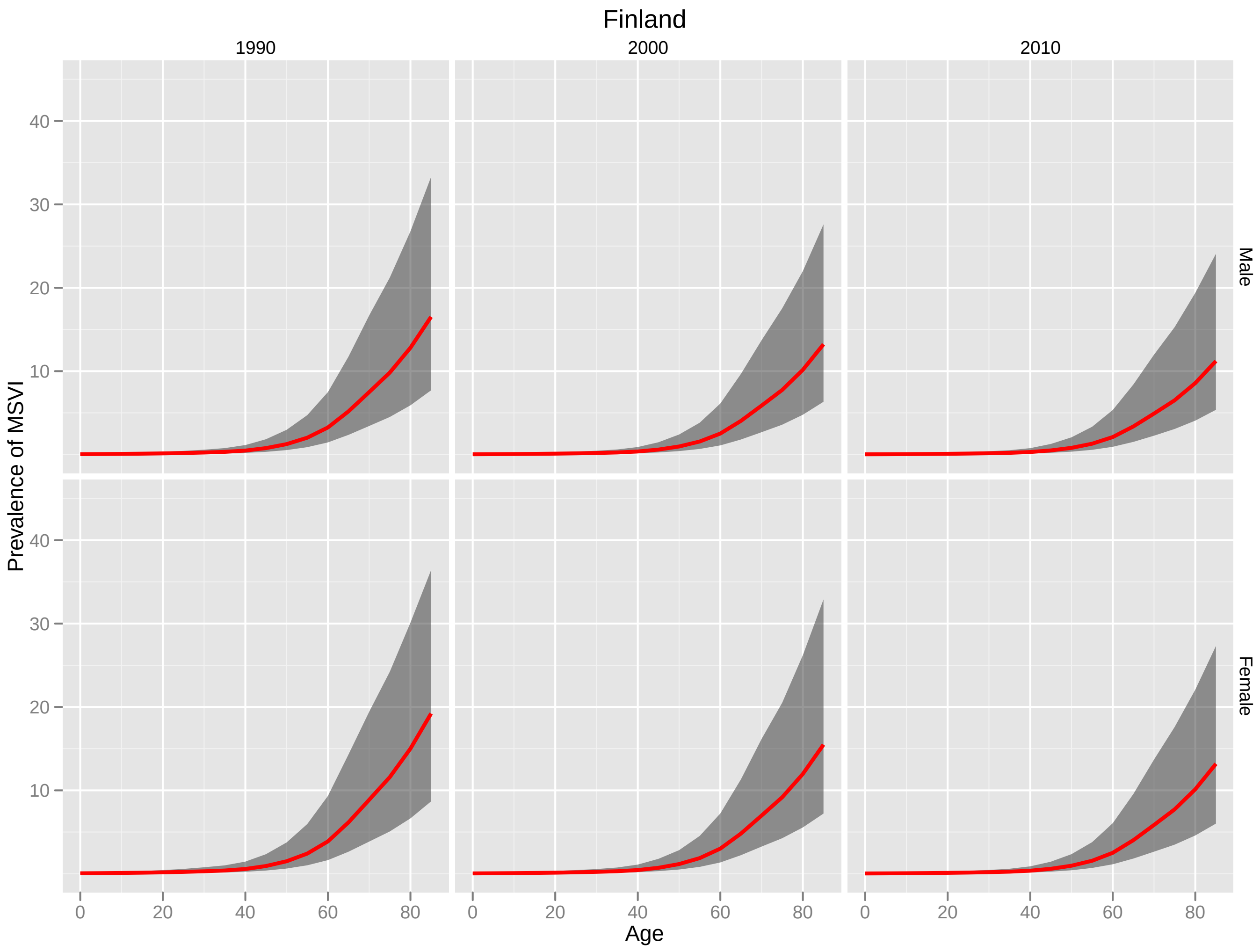

Finland
1990
2000
2010
Male
Female
Prevalence of MSVI
10
20
30
40
Age
0
20
40
60
80

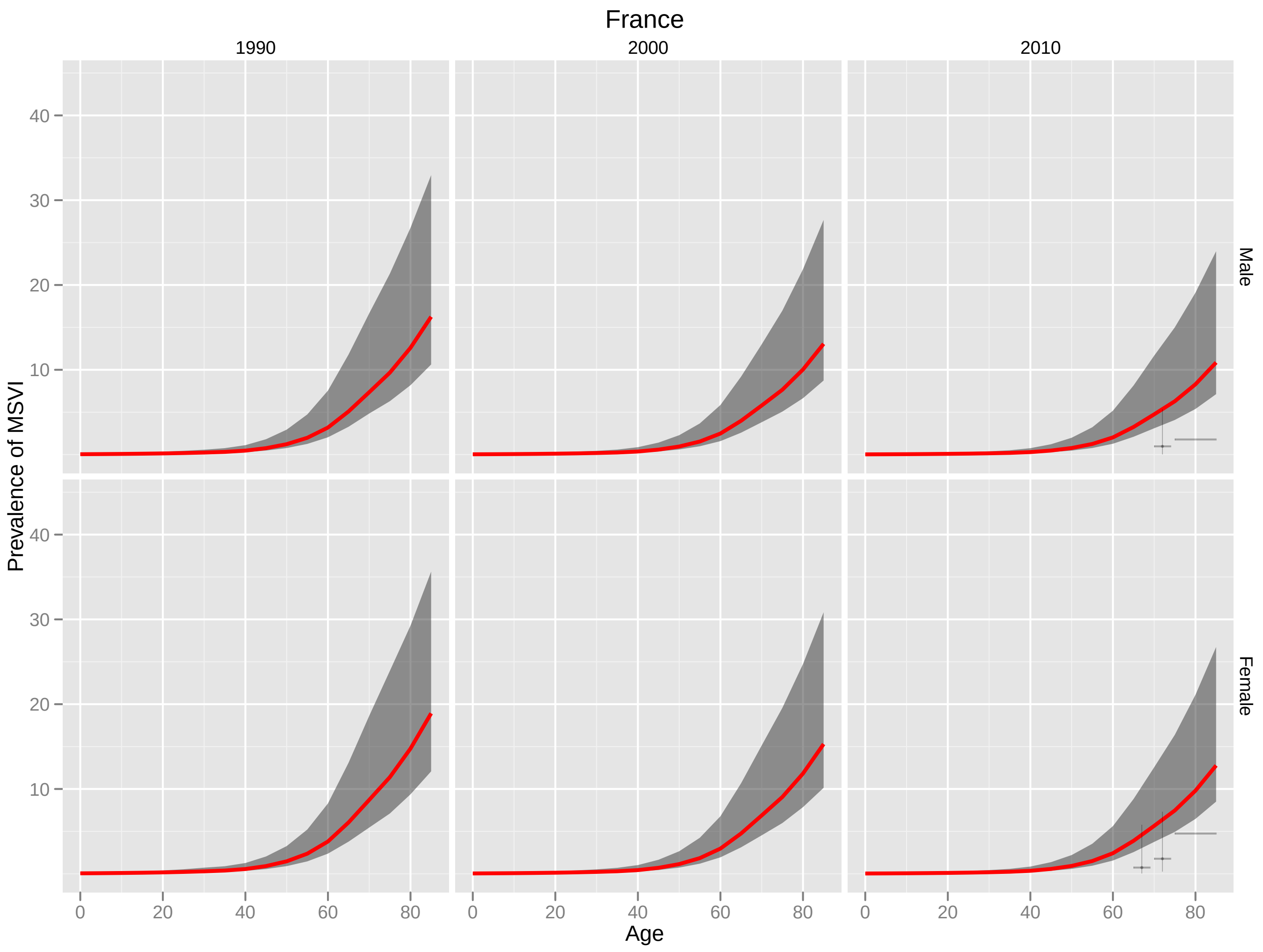
France
1990
2000
2010
Male
Female
Prevalence of MSVI
Age
0
20
40
60
80
10
20
30
40

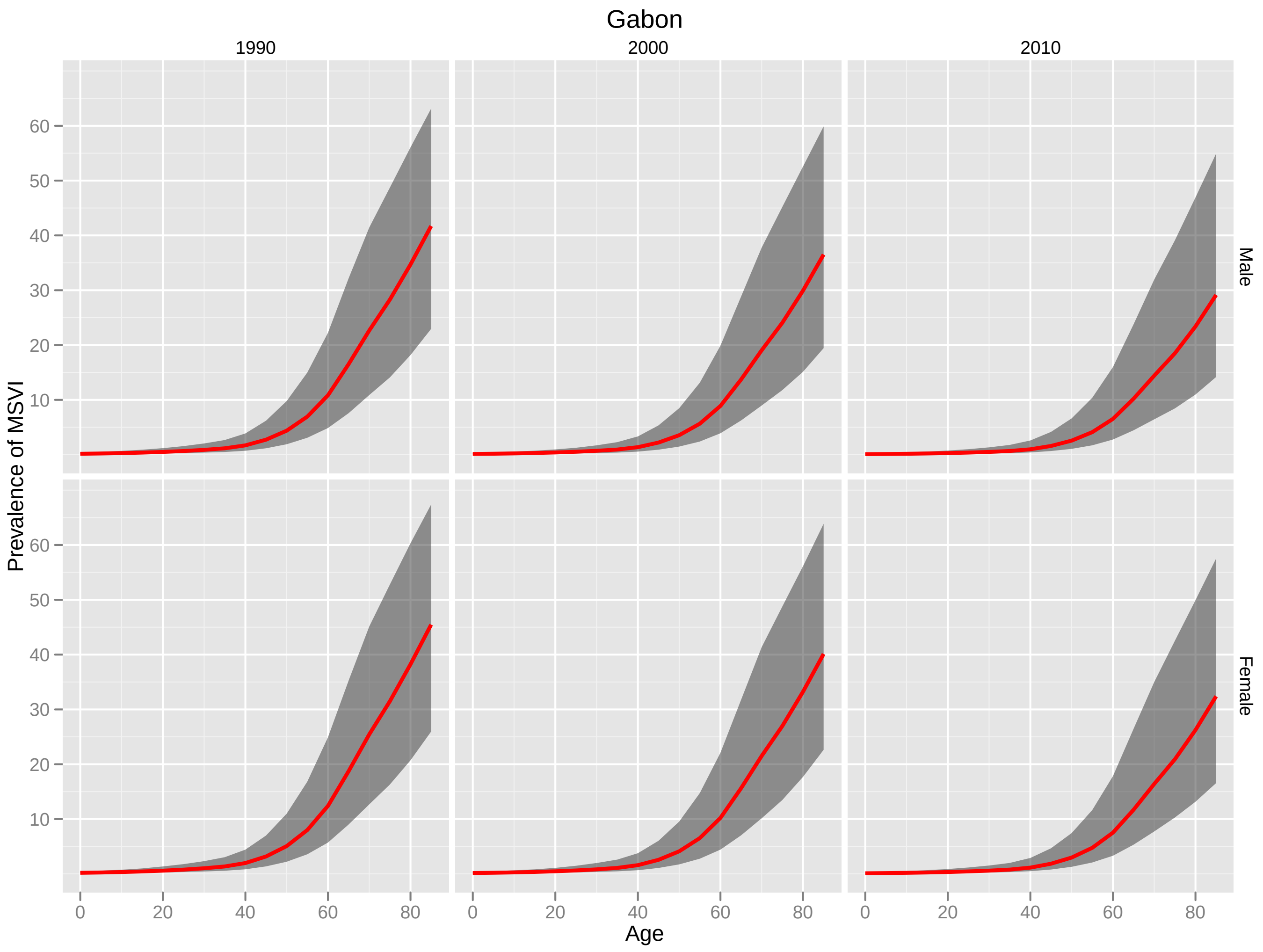
Gabon
1990
2000
2010
Male
Female
Prevalence of MSVI
Age
10
20
30
40
50
60
0
20
40
60
80

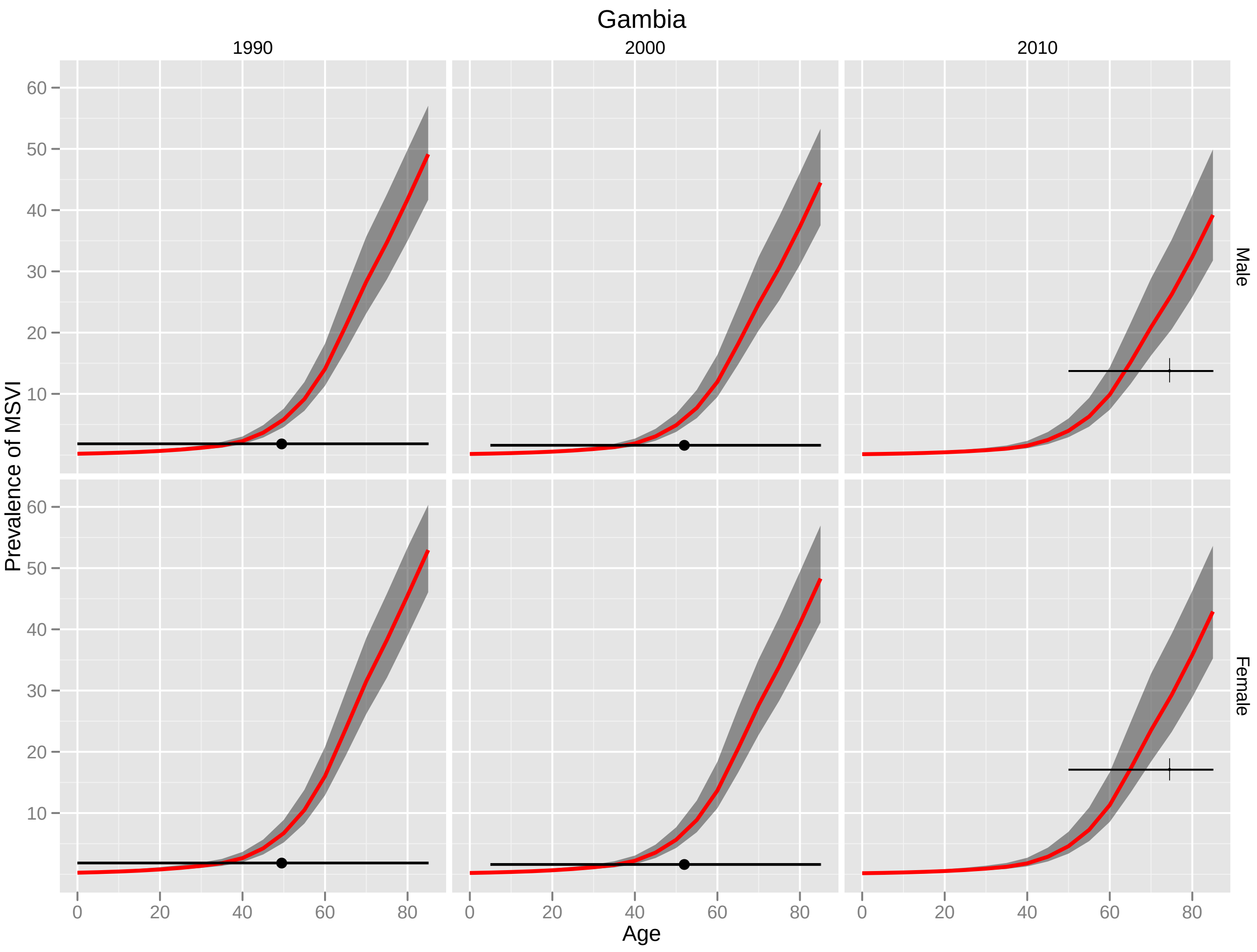

Gambia
1990
2000
2010
Male
Female
Prevalence of MSVI
Age
0
20
40
60
80
10
20
30
40
50
60

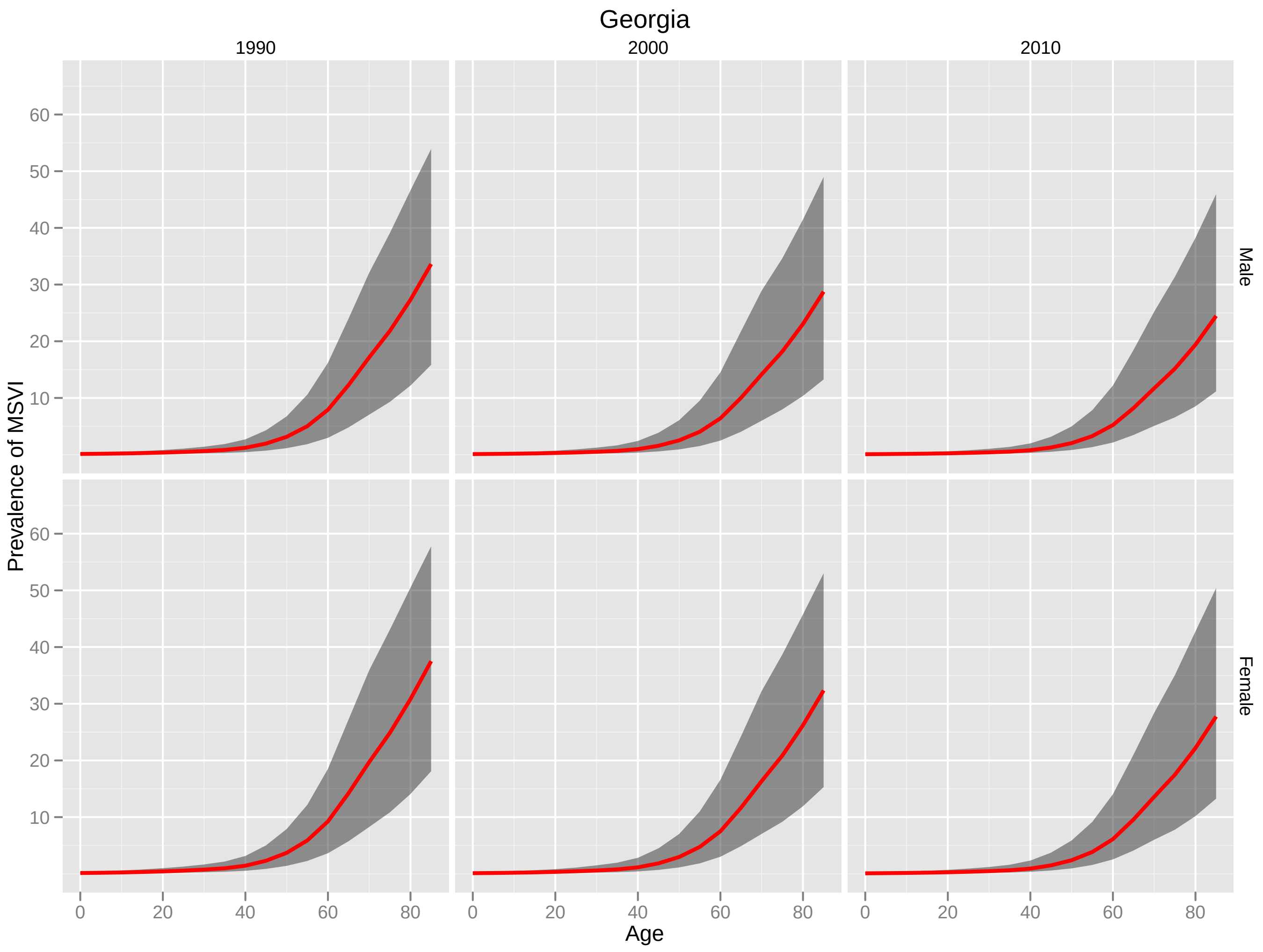
Georgia
1990
2000
2010
Male
Female
Prevalence of MSVI
Age
10
20
30
40
50
60
0
20
40
60
80

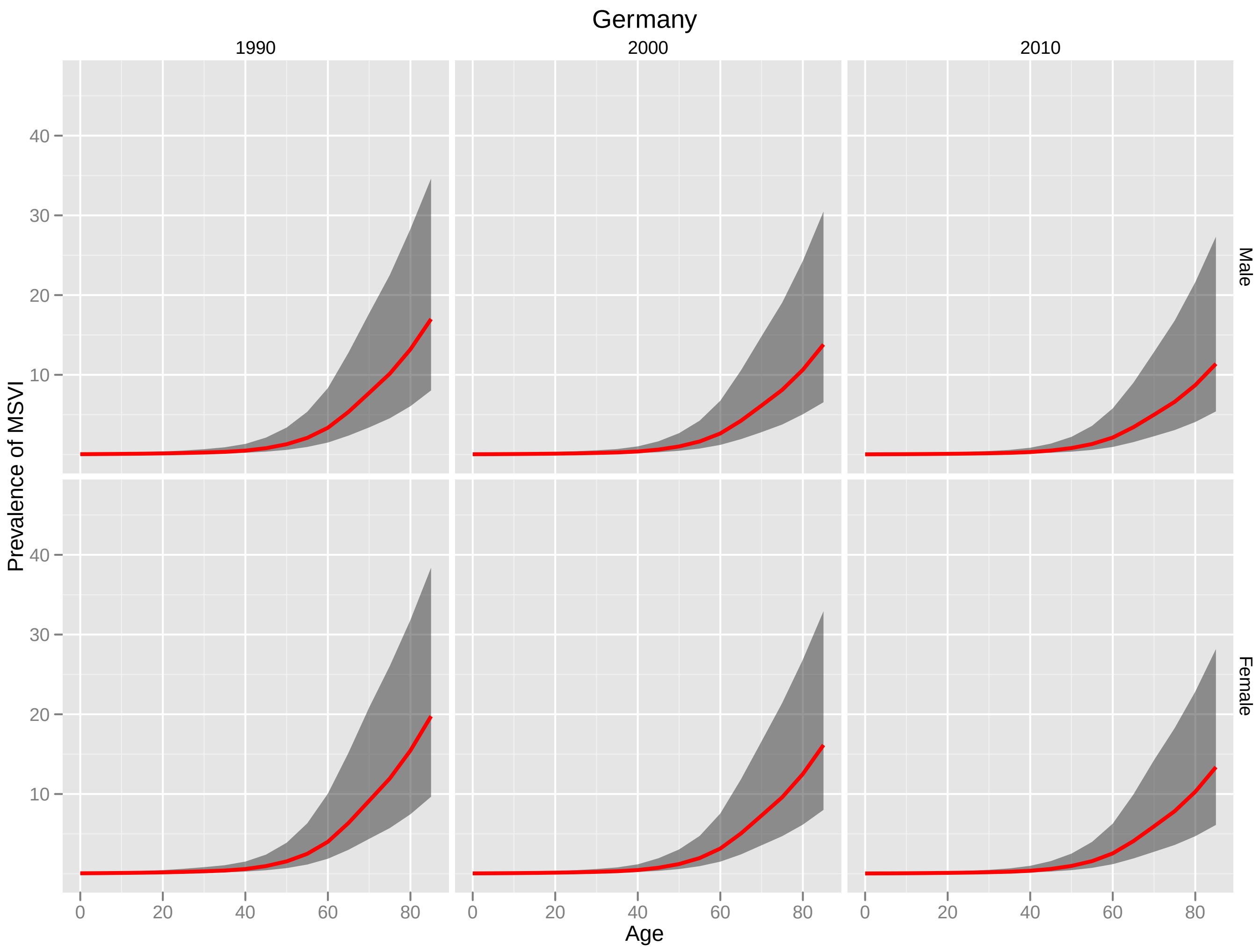
Germany
1990
2000
2010
Male
Female
Prevalence of MSVI
Age
10
20
30
40
0
20
40
60
80

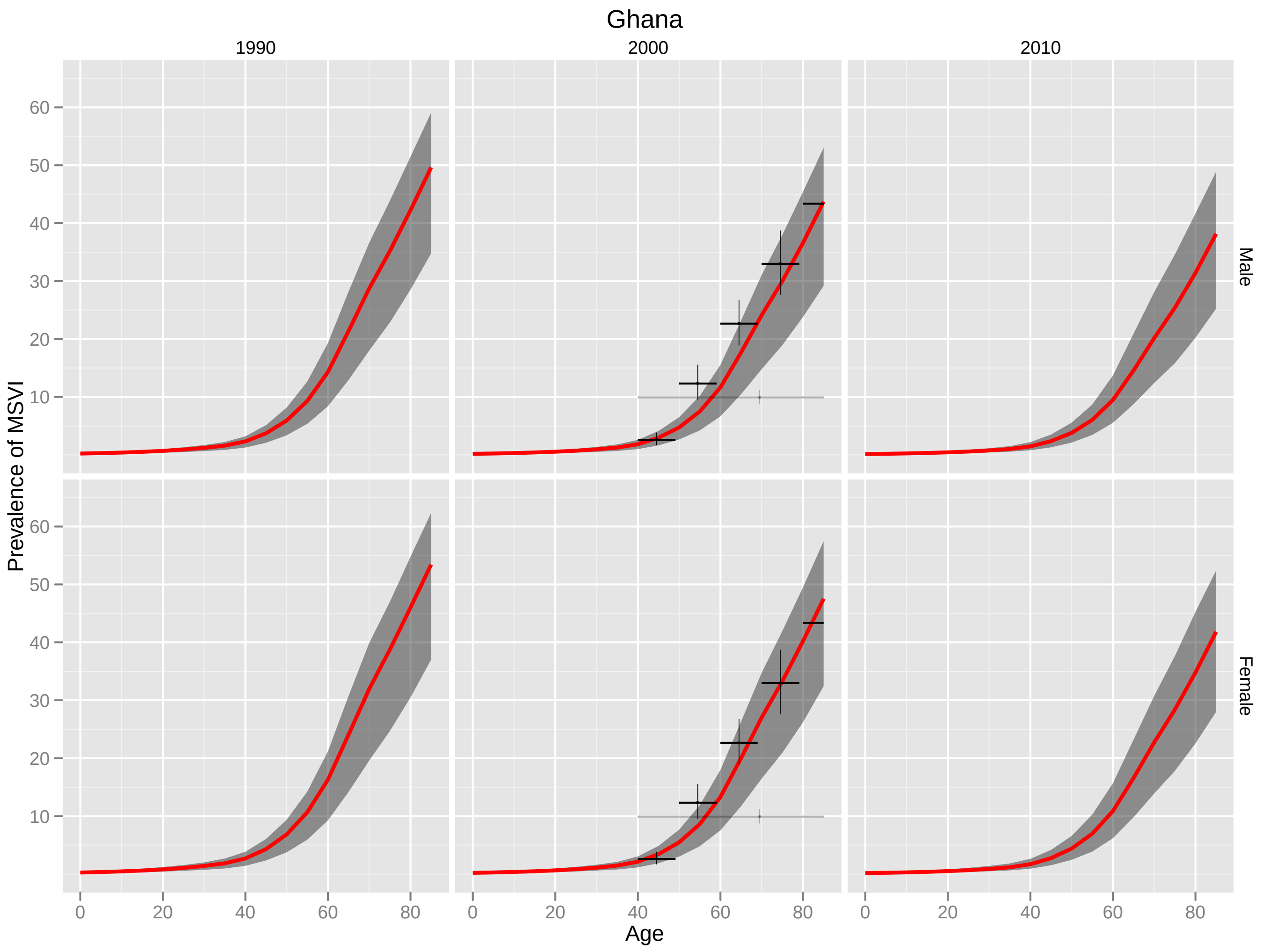

Ghana
1990
2000
2010
Male
Female
Prevalence of MSVI
Age
10
20
30
40
50
60
0
20
40
60
80

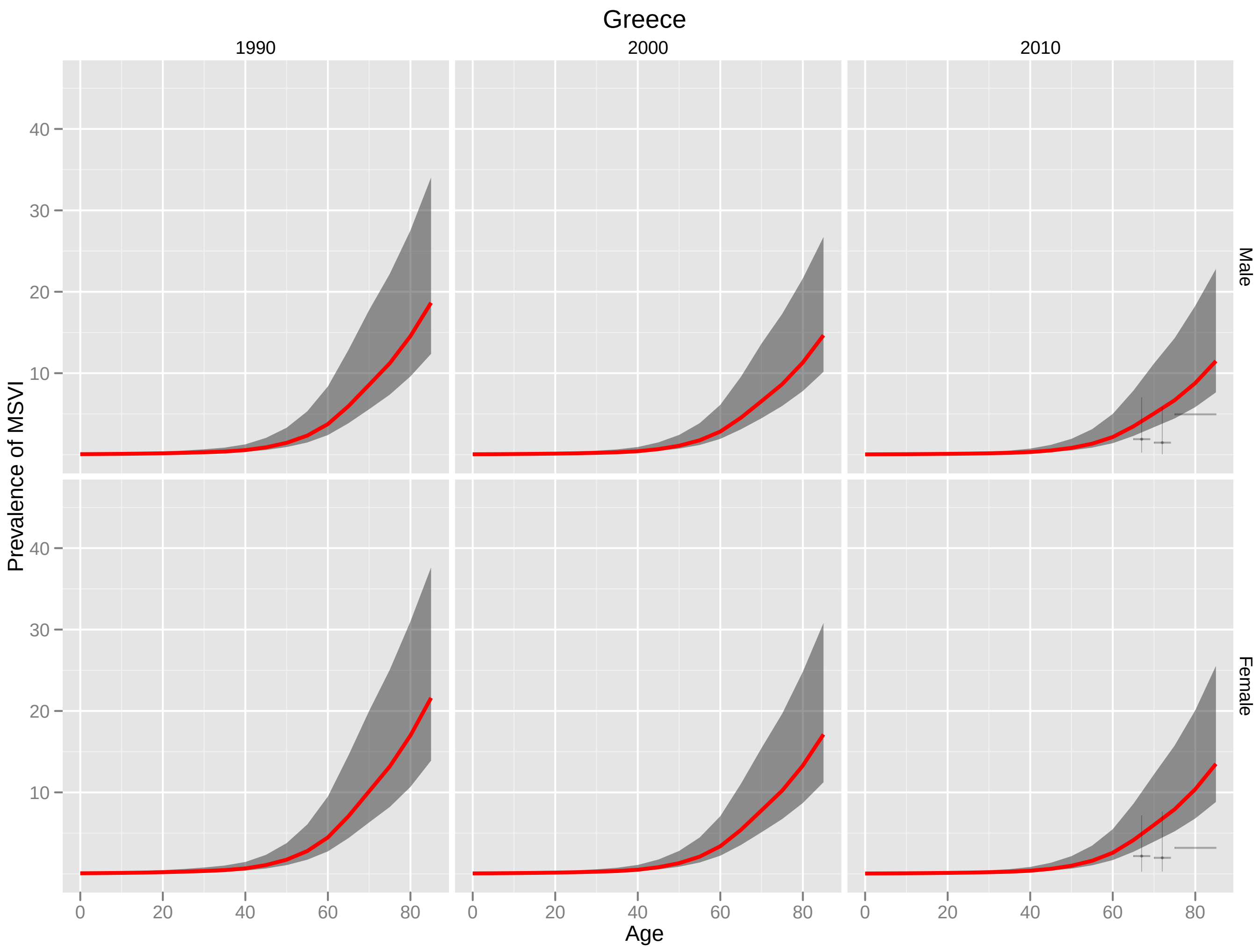
Greece
1990
2000
2010
Male
Female
Prevalence of MSVI
Age
0
20
40
60
80
10
20
30
40

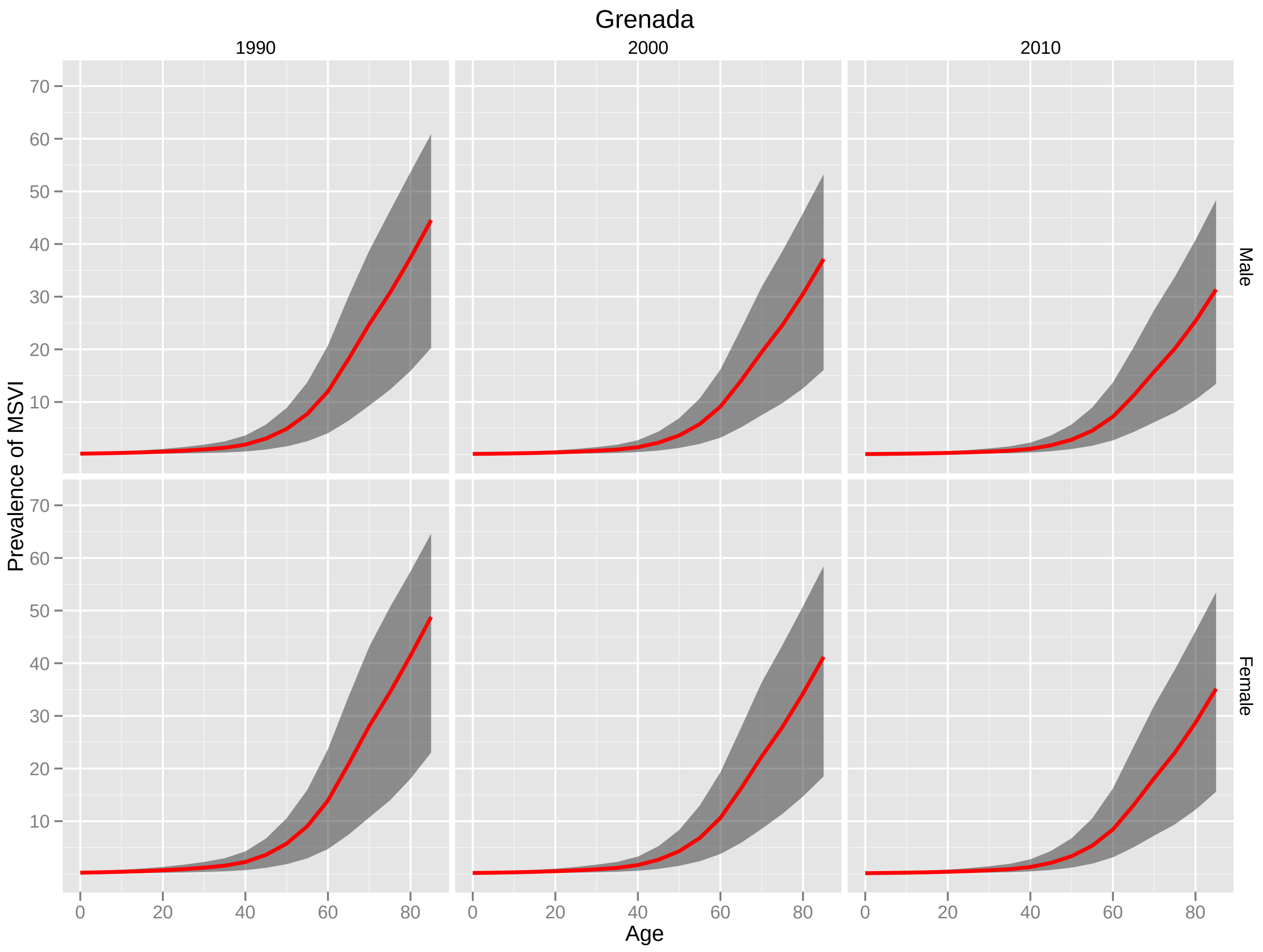
Grenada
1990
2000
2010
Male
Female
Prevalence of MSVI
Age
70
60
50
40
30
20
10
0
20
40
60
80

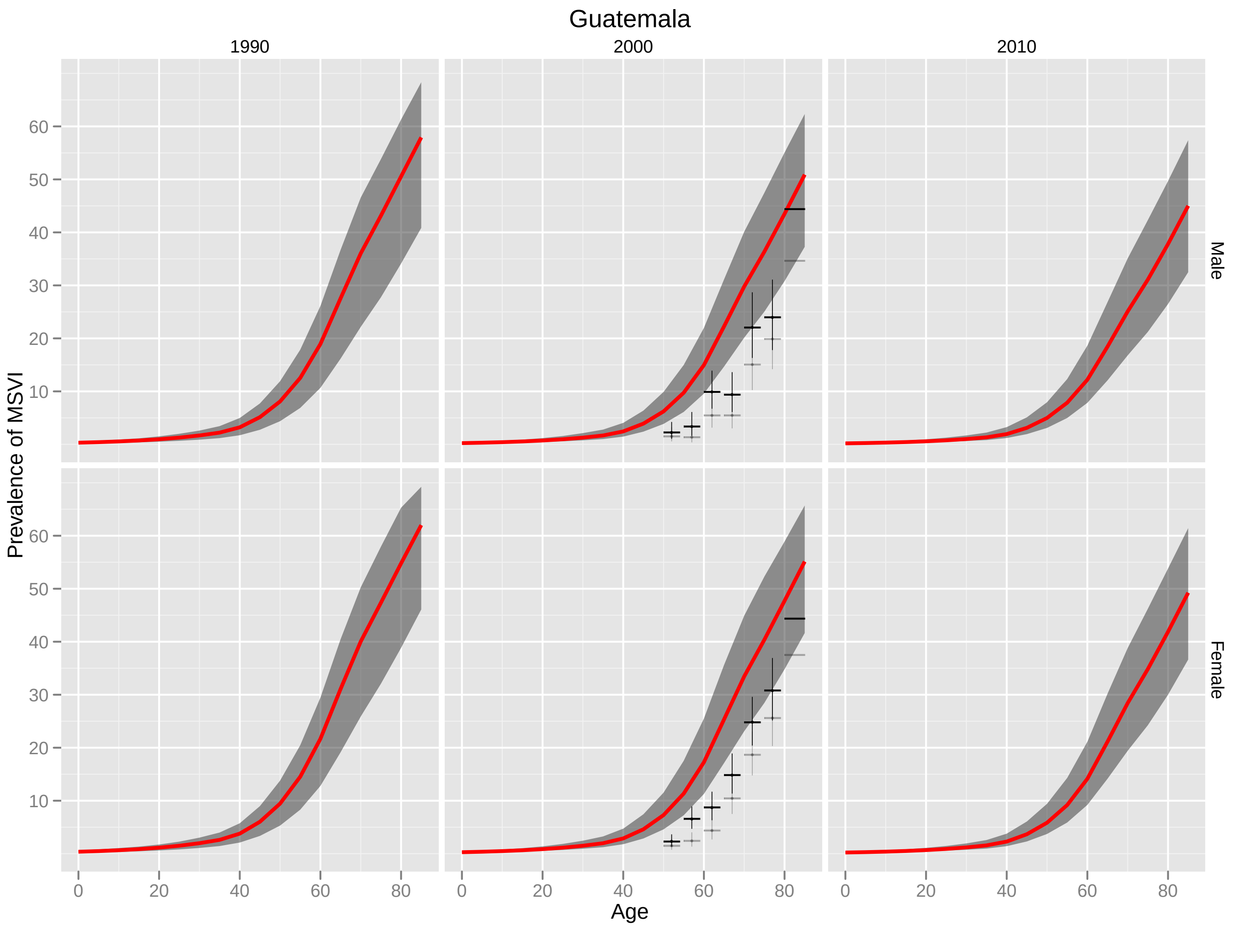

Guatemala
1990
2000
2010
Male
Female
Prevalence of MSVI
Age
10
20
30
40
50
60
0
20
40
60
80

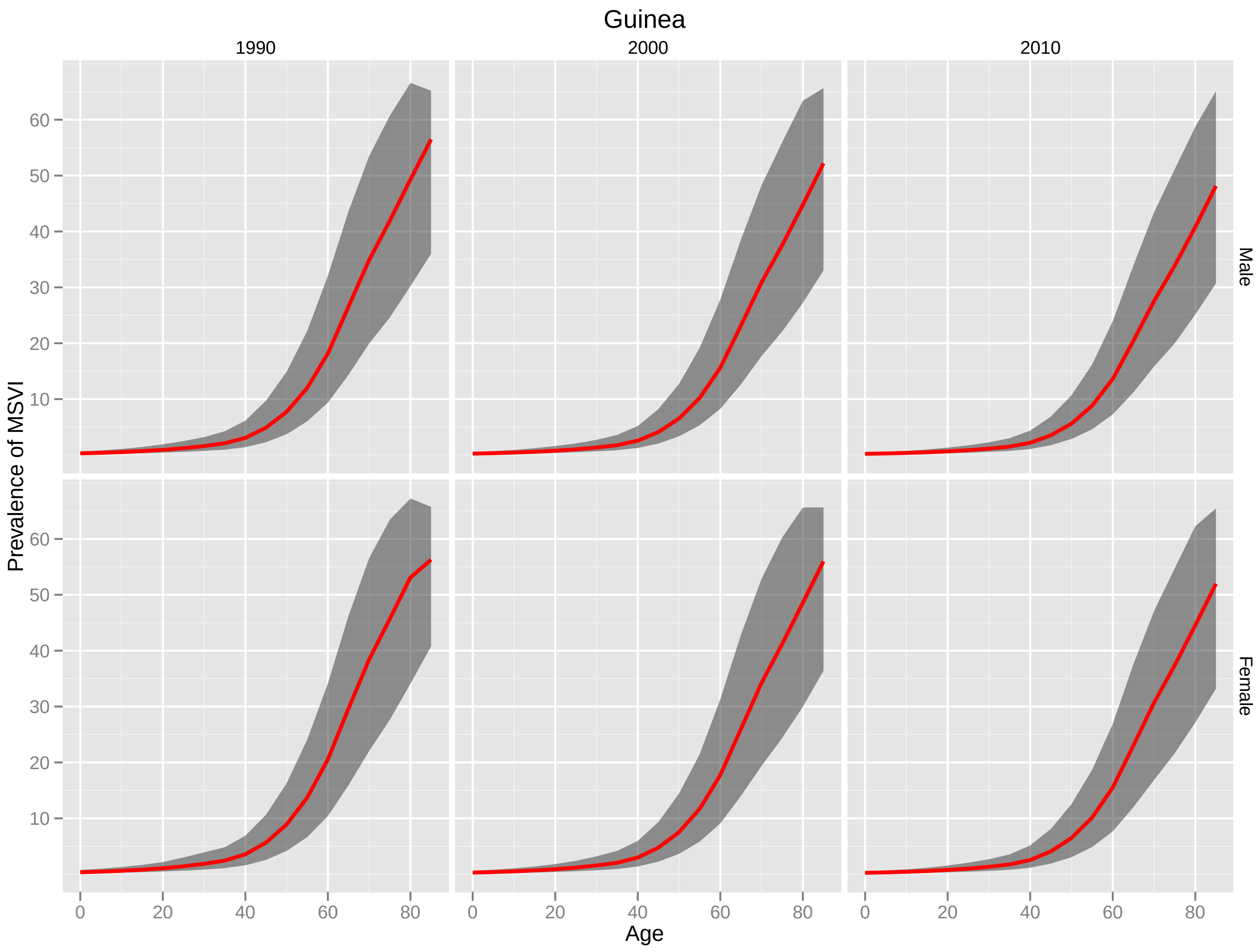
Guinea
1990
2000
2010
Male
Female
Prevalence of MSVI
Age
10
20
30
40
50
60
0
20
40
60
80

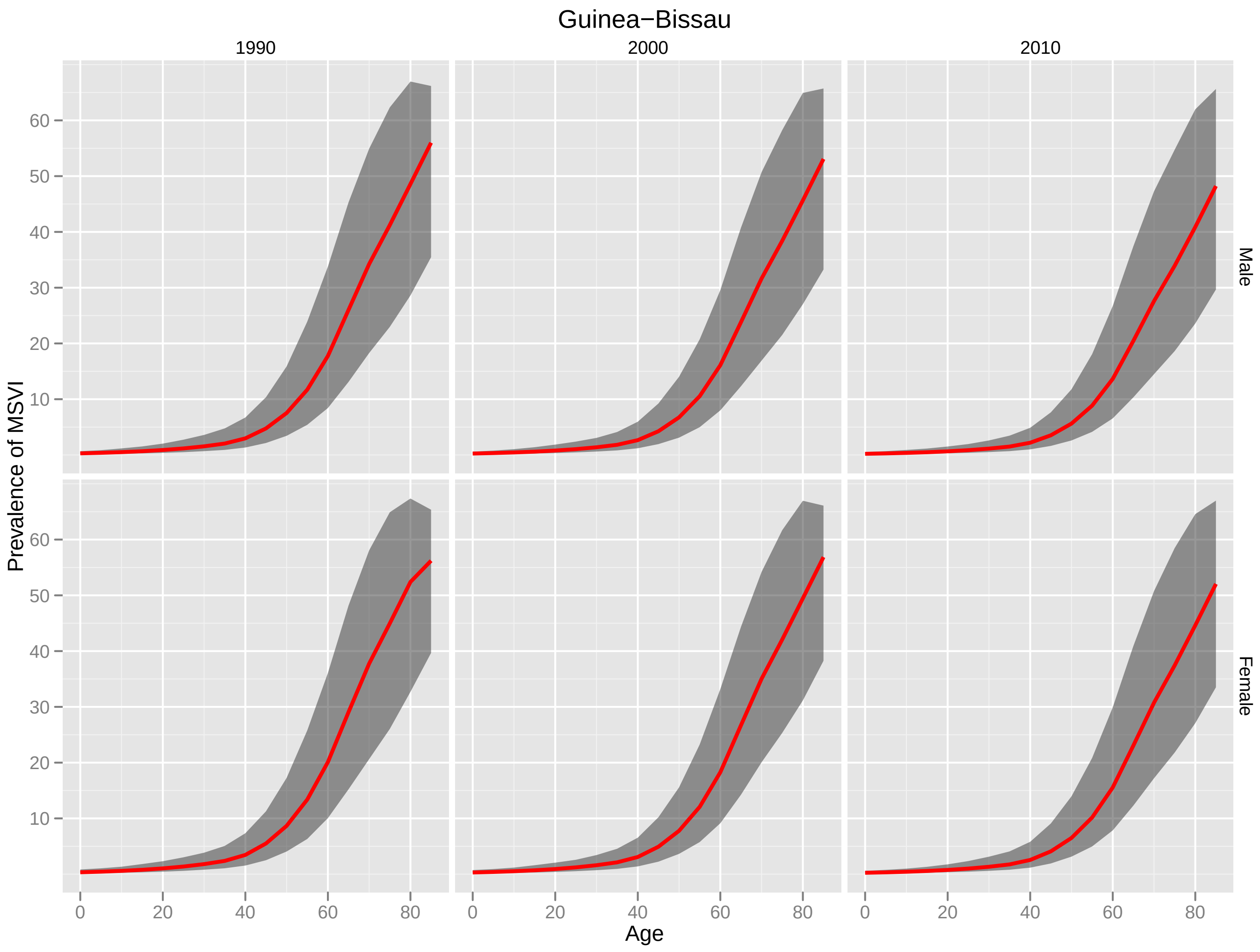
Guinea−Bissau
1990
2000
2010
Male
Female
Prevalence of MSVI
Age
10
20
30
40
50
60
0
20
40
60
80

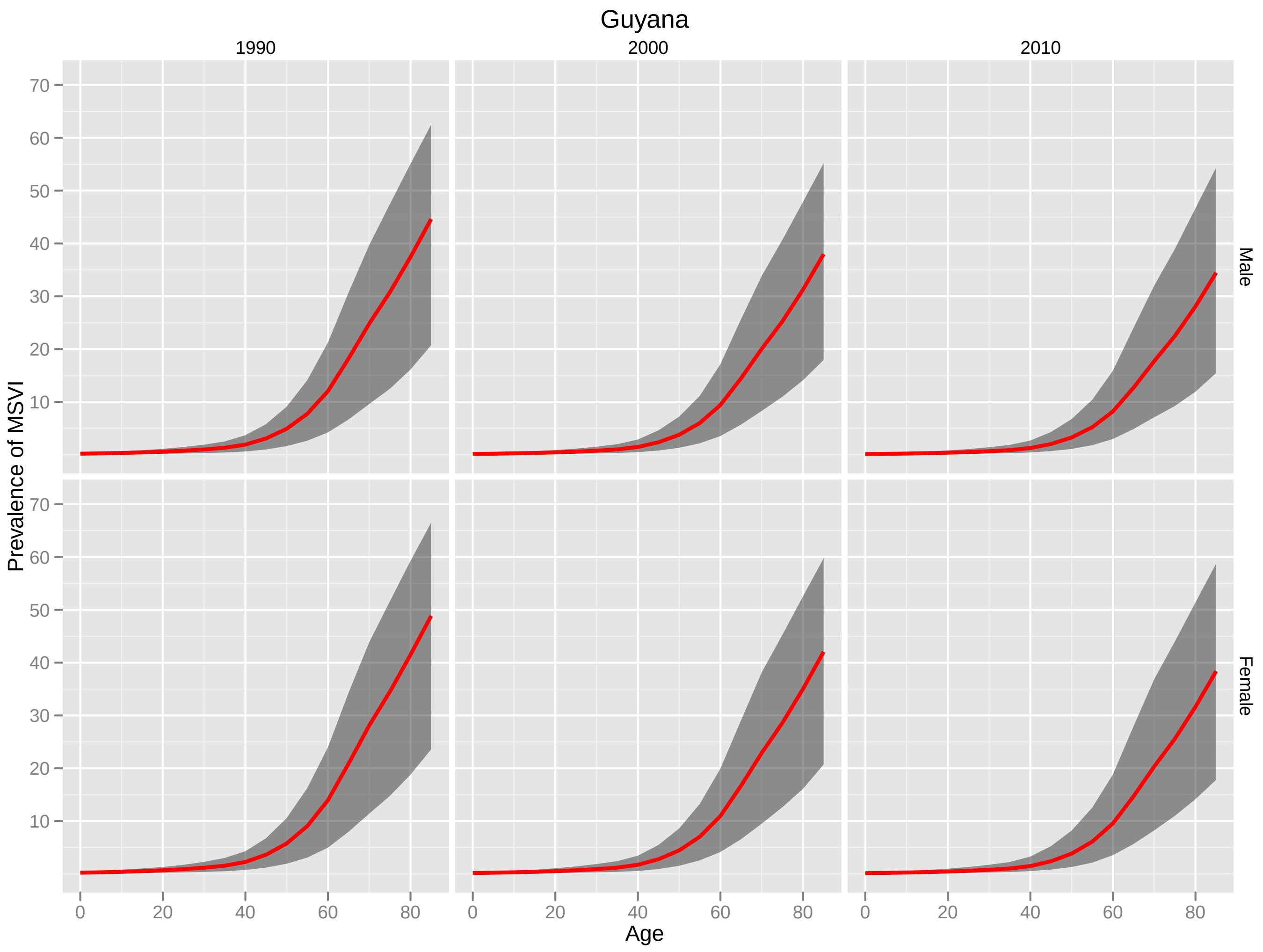

Guyana
1990
2000
2010
Male
Female
Prevalence of MSVI
Age
0
20
40
60
80
10
20
30
40
50
60
70

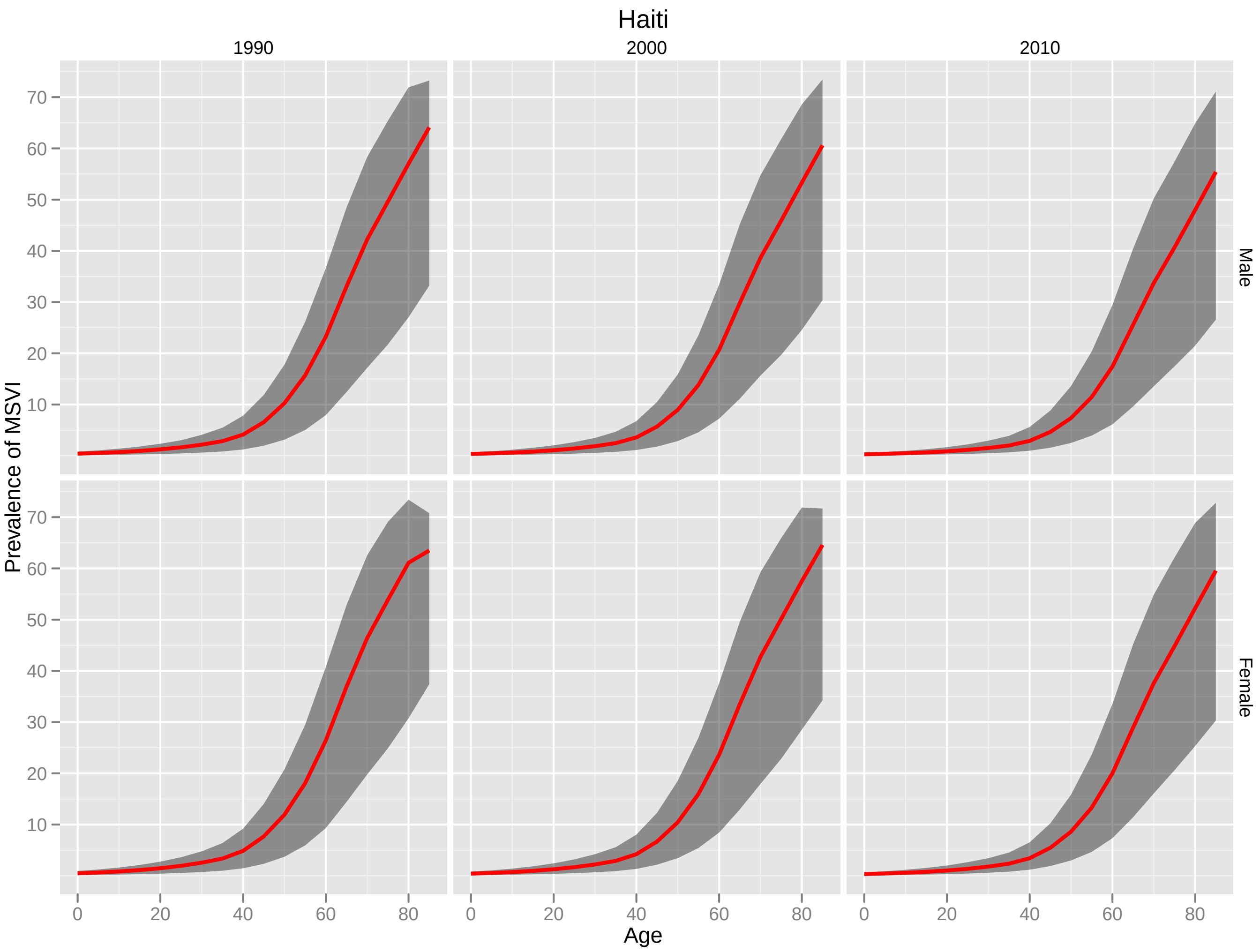

Haiti
1990
2000
2010
Male
Female
Prevalence of MSVI
Age
10
20
30
40
50
60
70
0
20
40
60
80

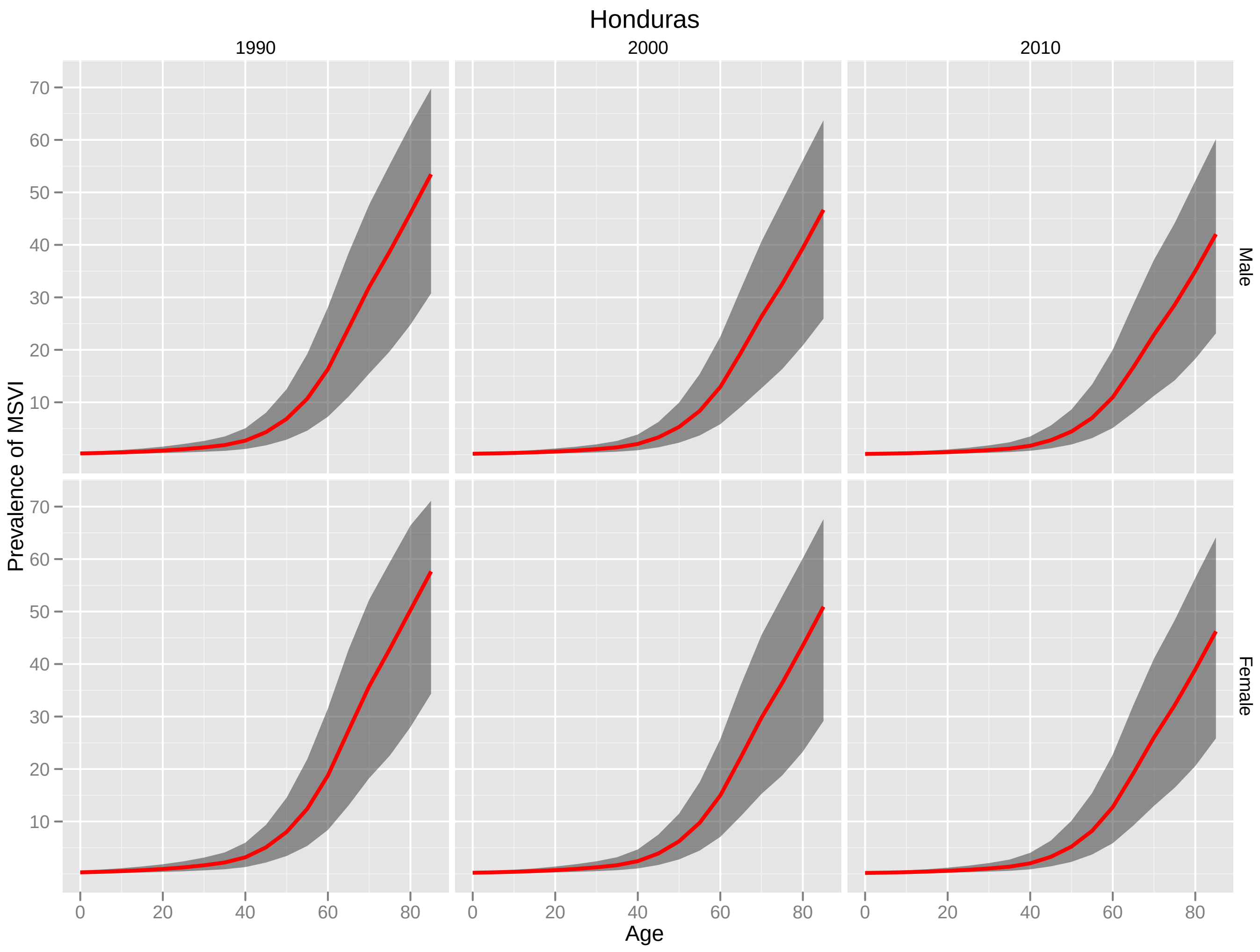
Honduras
1990
2000
2010
Male
Female
Prevalence of MSVI
Age
0
20
40
60
80
10
20
30
40
50
60
70

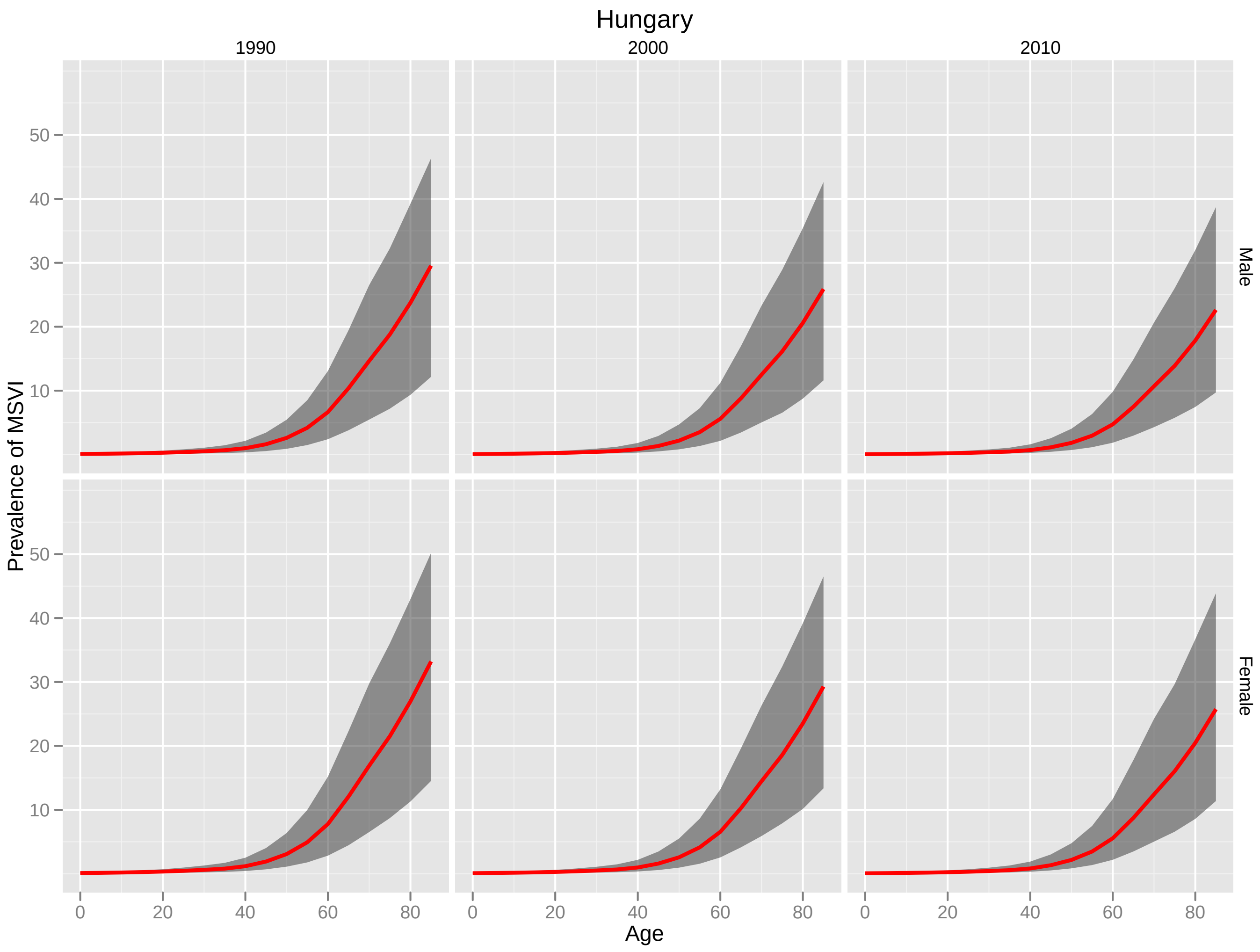
Hungary
1990
2000
2010
Male
Female
Prevalence of MSVI
Age
10
20
30
40
50
0
60
80

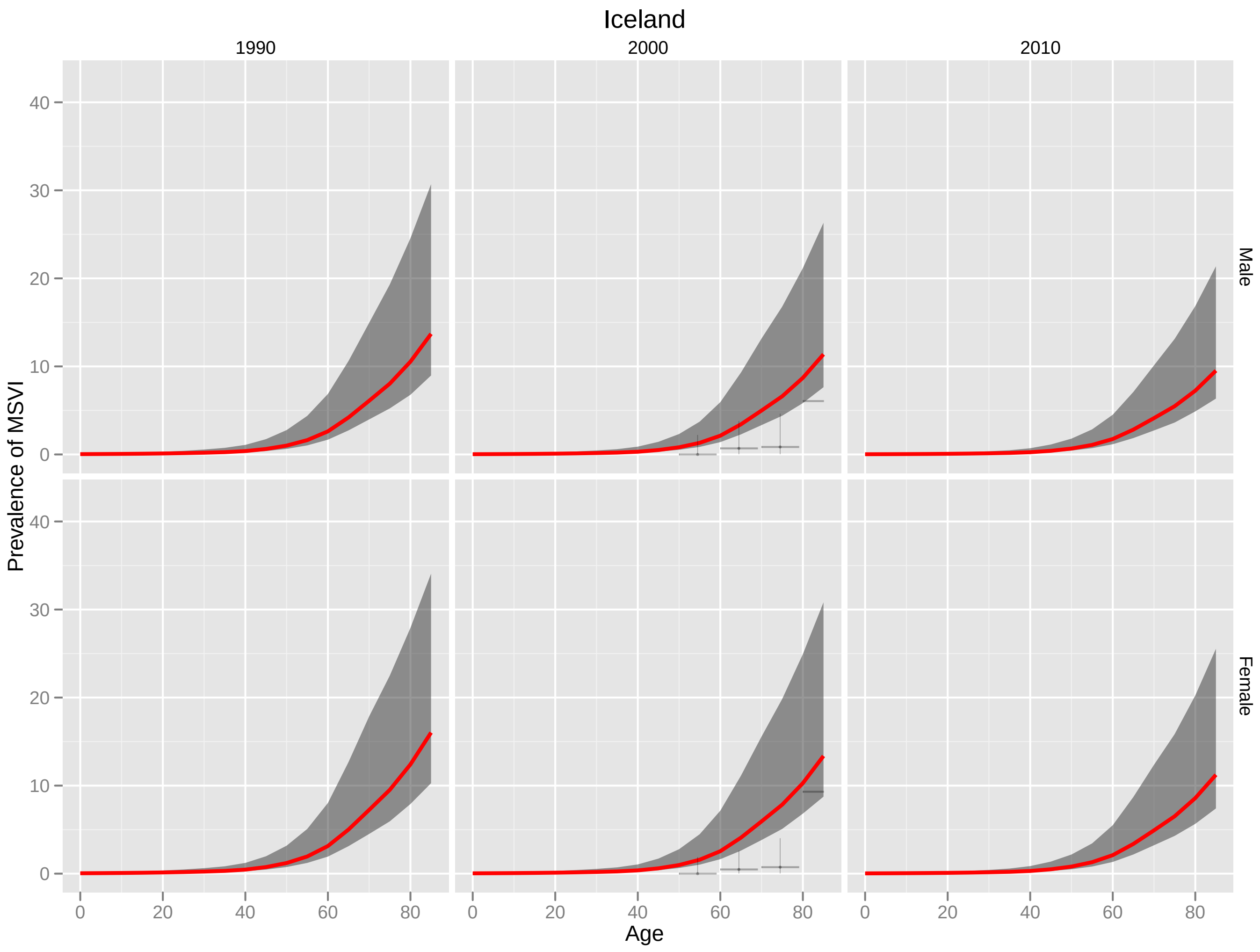

Iceland
1990
2000
2010
Male
Female
Prevalence of MSVI
Age
0
10
20
30
40
0
20
40
60
80

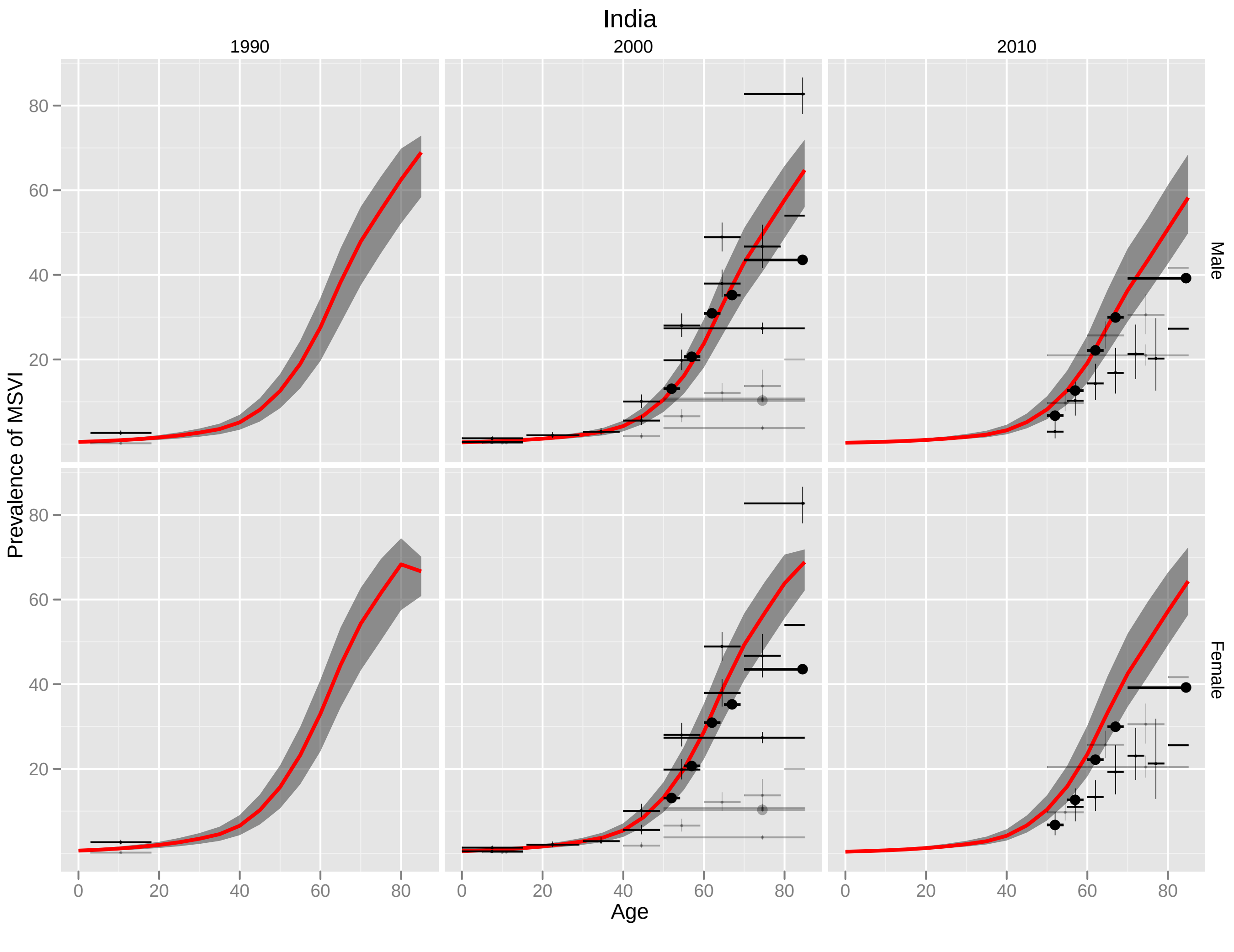
India
1990
2000
2010
Male
Female
Prevalence of MSVI
Age
0
20
40
60
80

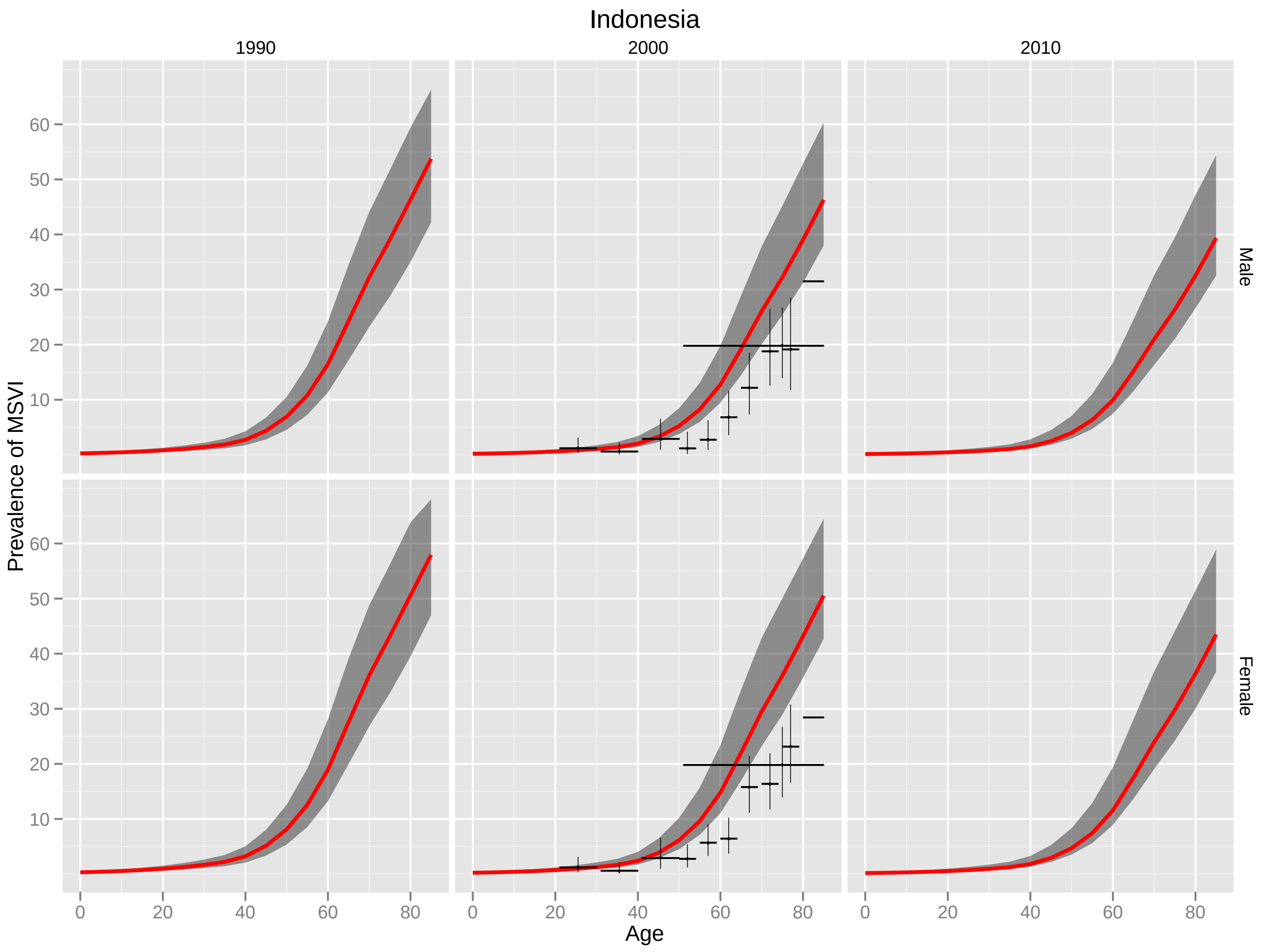
Indonesia
1990
2000
2010
Male
Female
Prevalence of MSVI
10
20
30
40
50
60
Age
0
20
40
60
80

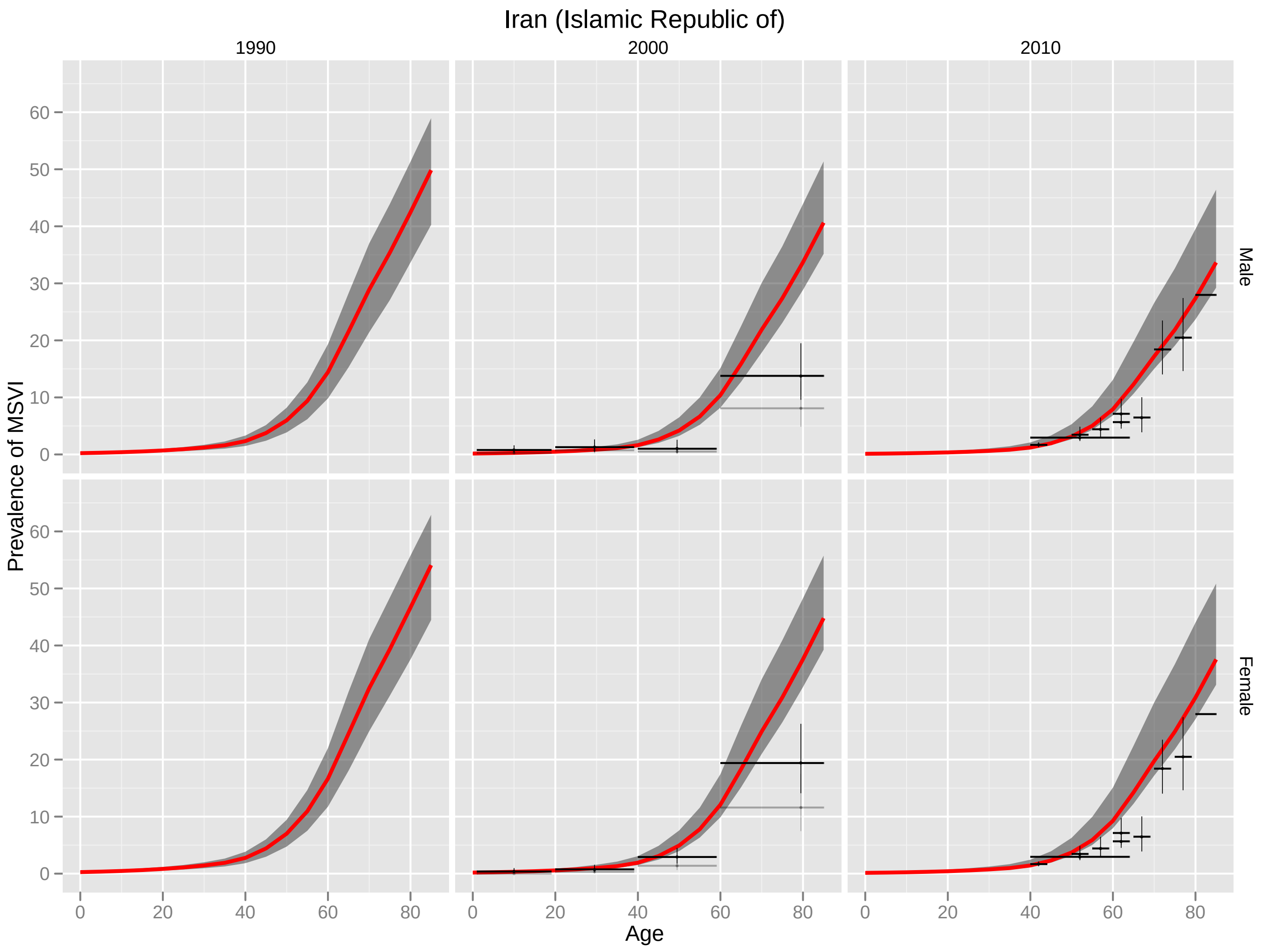

Iran (Islamic Republic of)
1990
2000
2010
Male
Female
Prevalence of MSVI
Age
0
10
20
30
40
50
60
80

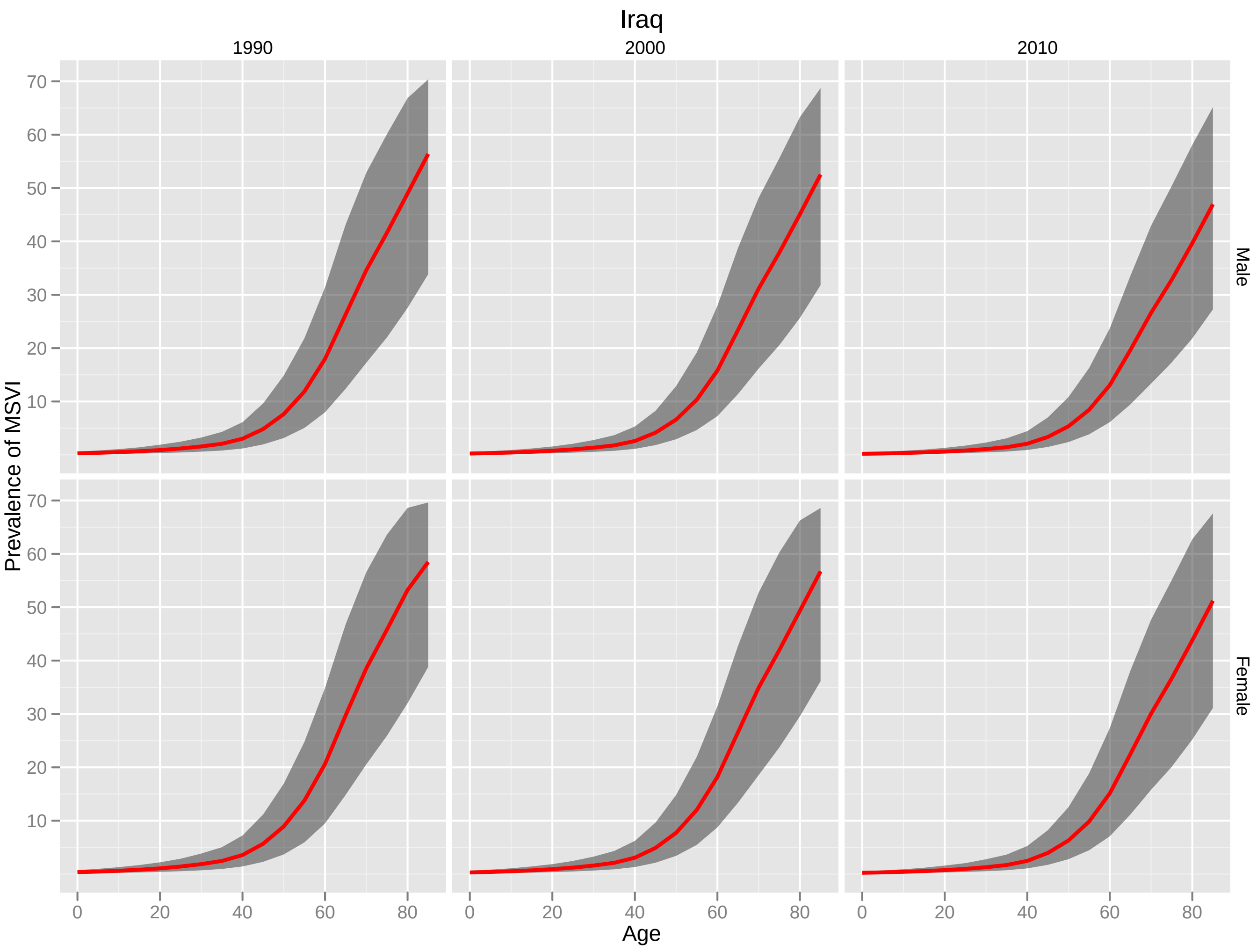

Iraq
1990
2000
2010
Male
Female
Prevalence of MSVI
Age
0
20
40
60
80
10
20
30
40
50
60
70

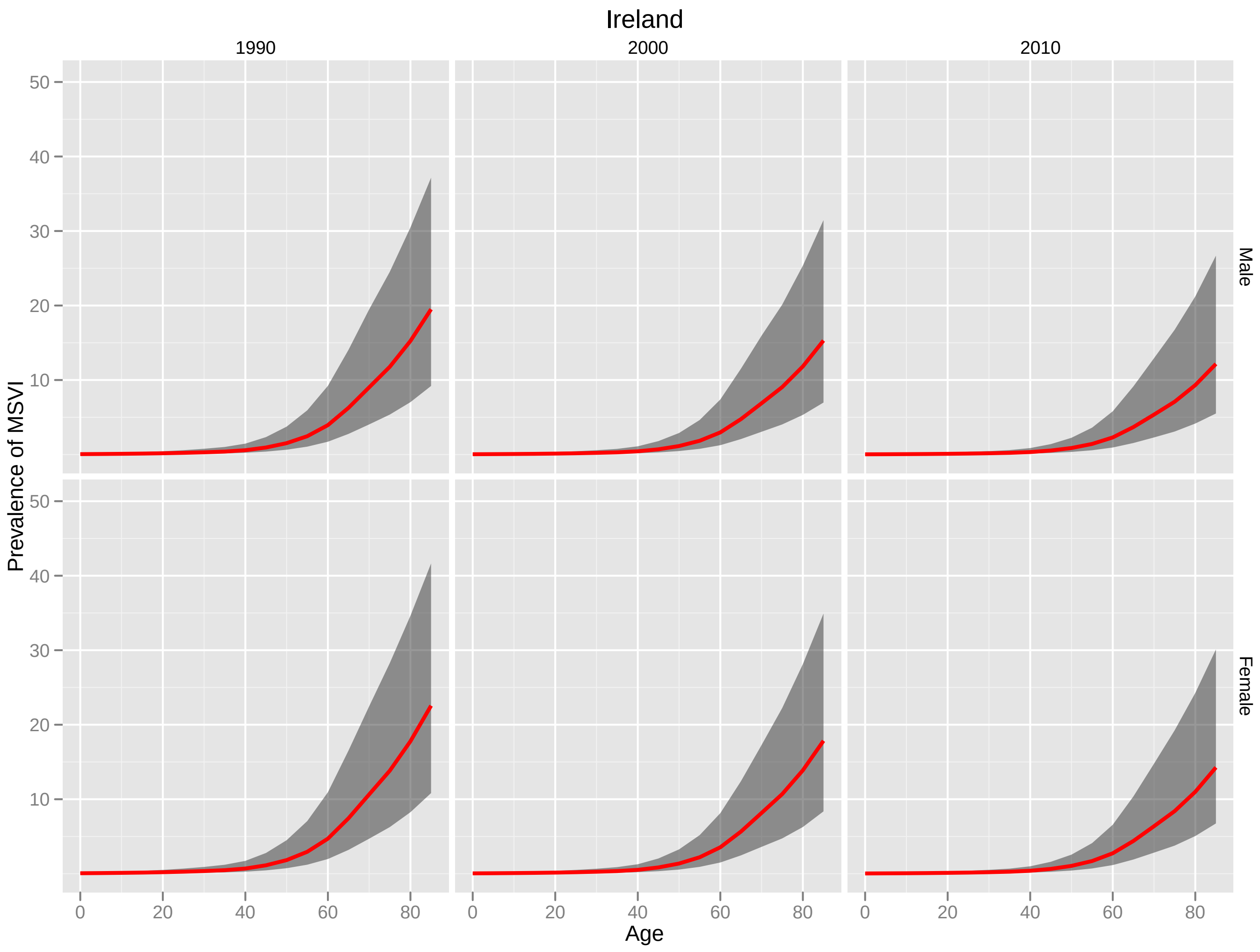
Ireland
1990
2000
2010
Male
Female
Prevalence of MSVI
Age
50
40
30
20
10
0
20
40
60
80

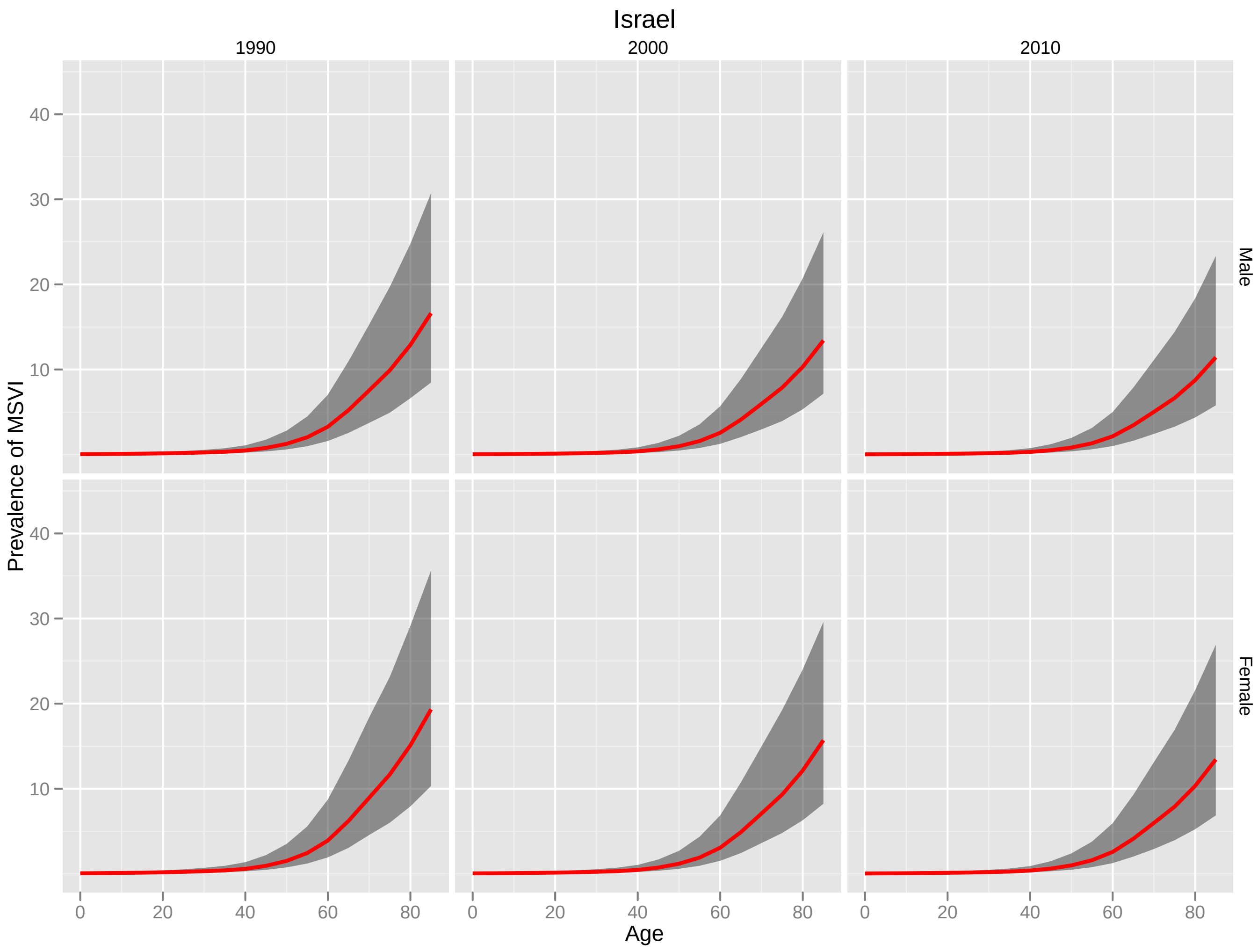
Israel
1990
2000
2010
Male
Female
Prevalence of MSVI
Age
0
20
40
60
80
10
20
30
40

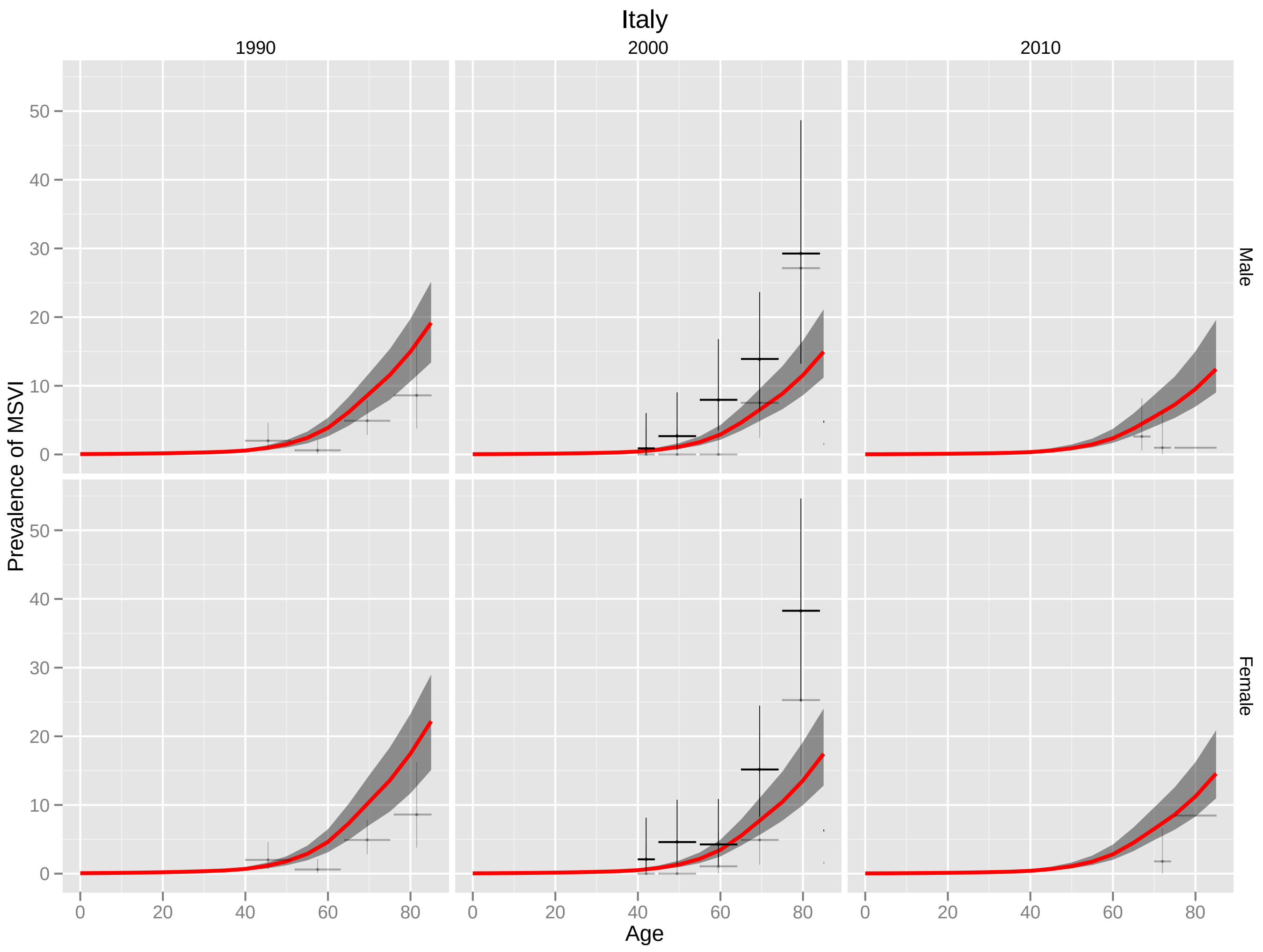
Italy
1990
2000
2010
Male
Female
Prevalence of MSVI
Age
0
10
20
30
40
50
0
20
40
60
80

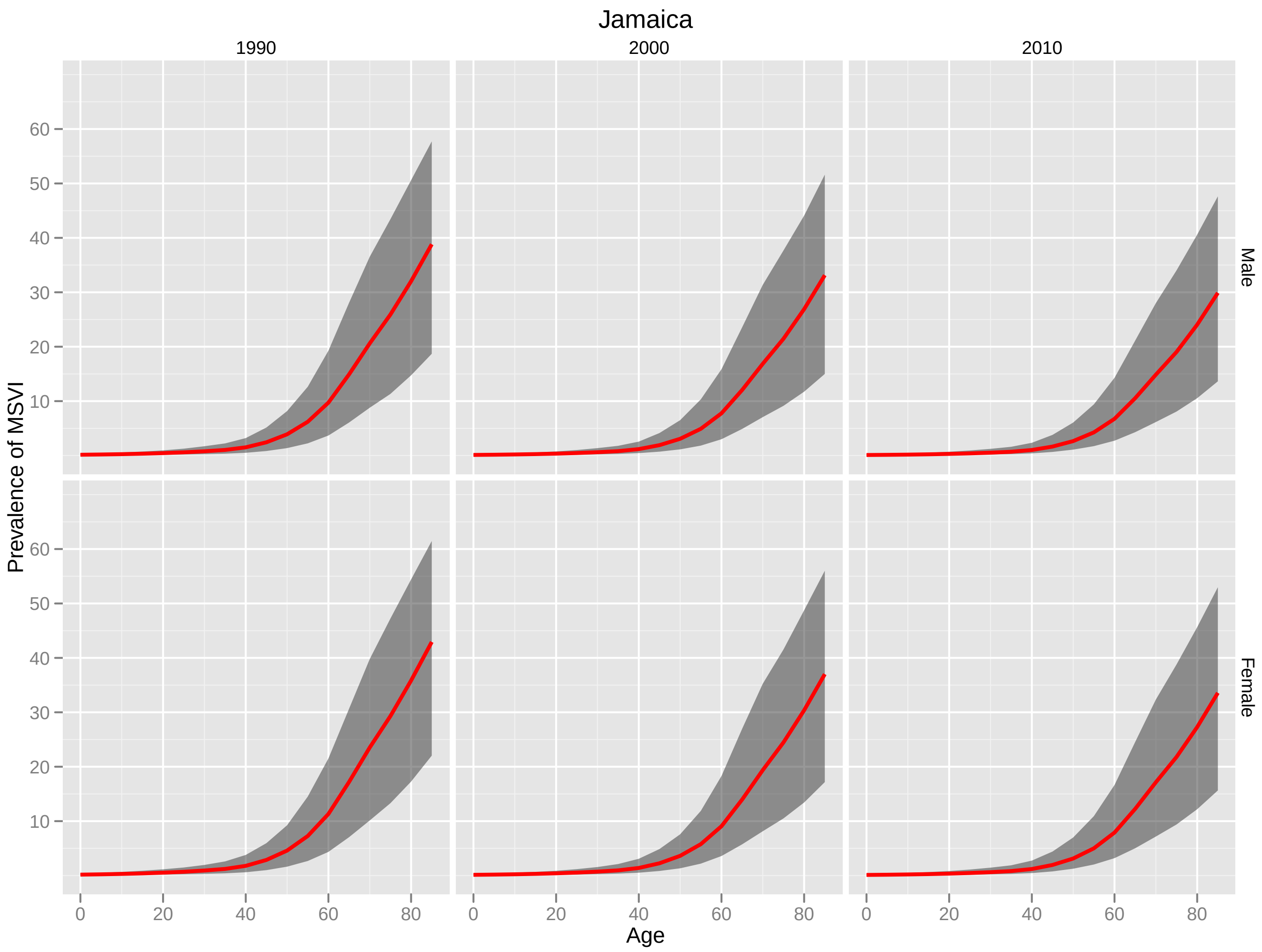
Jamaica
1990
2000
2010
Male
Female
Prevalence of MSVI
Age
10
20
30
40
50
60
0
20
40
60
80

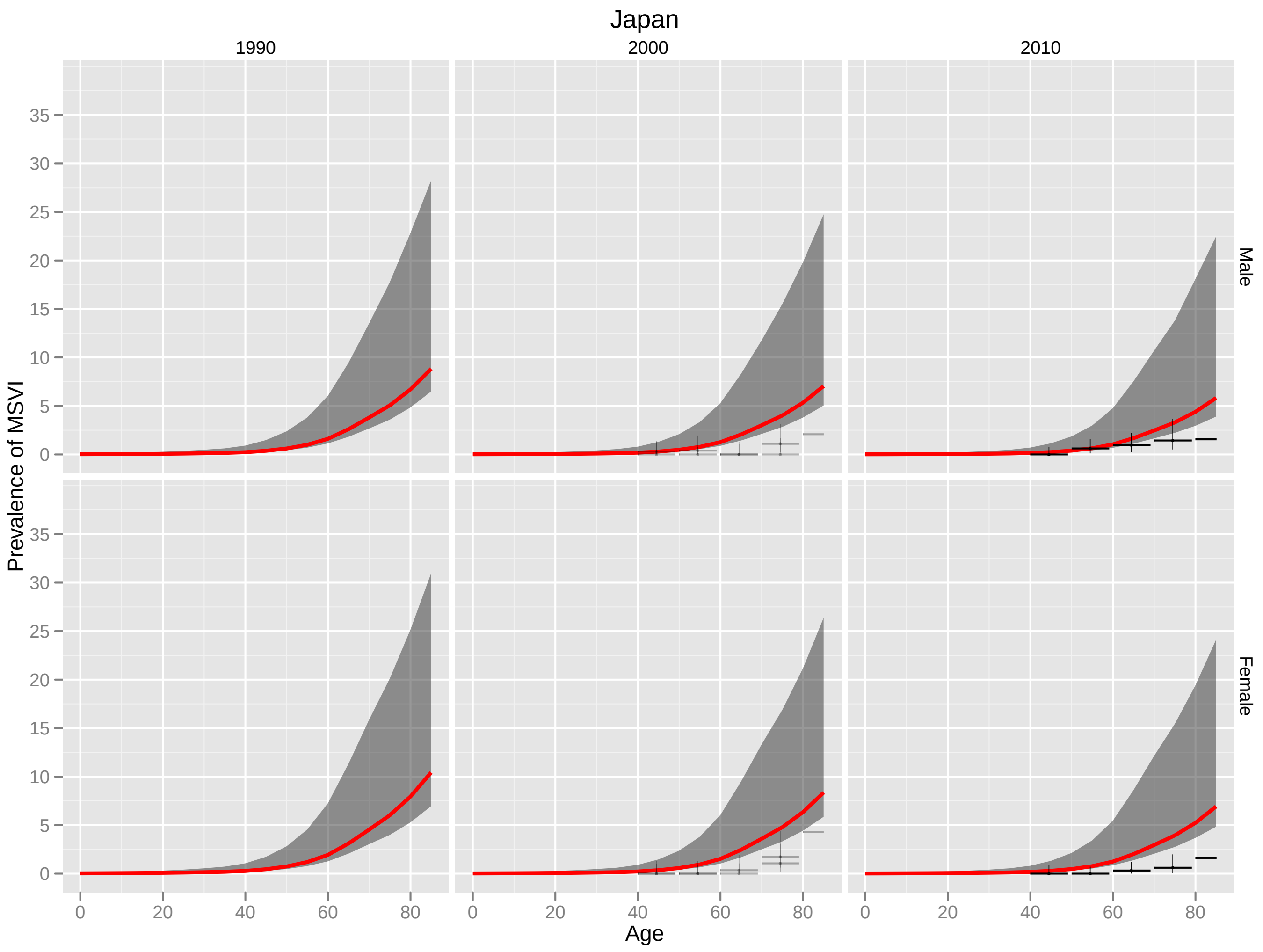
Japan
1990
2000
2010
Male
Female
Prevalence of MSVI
Age
0
5
10
15
20
25
30
35
0
20
40
60
80

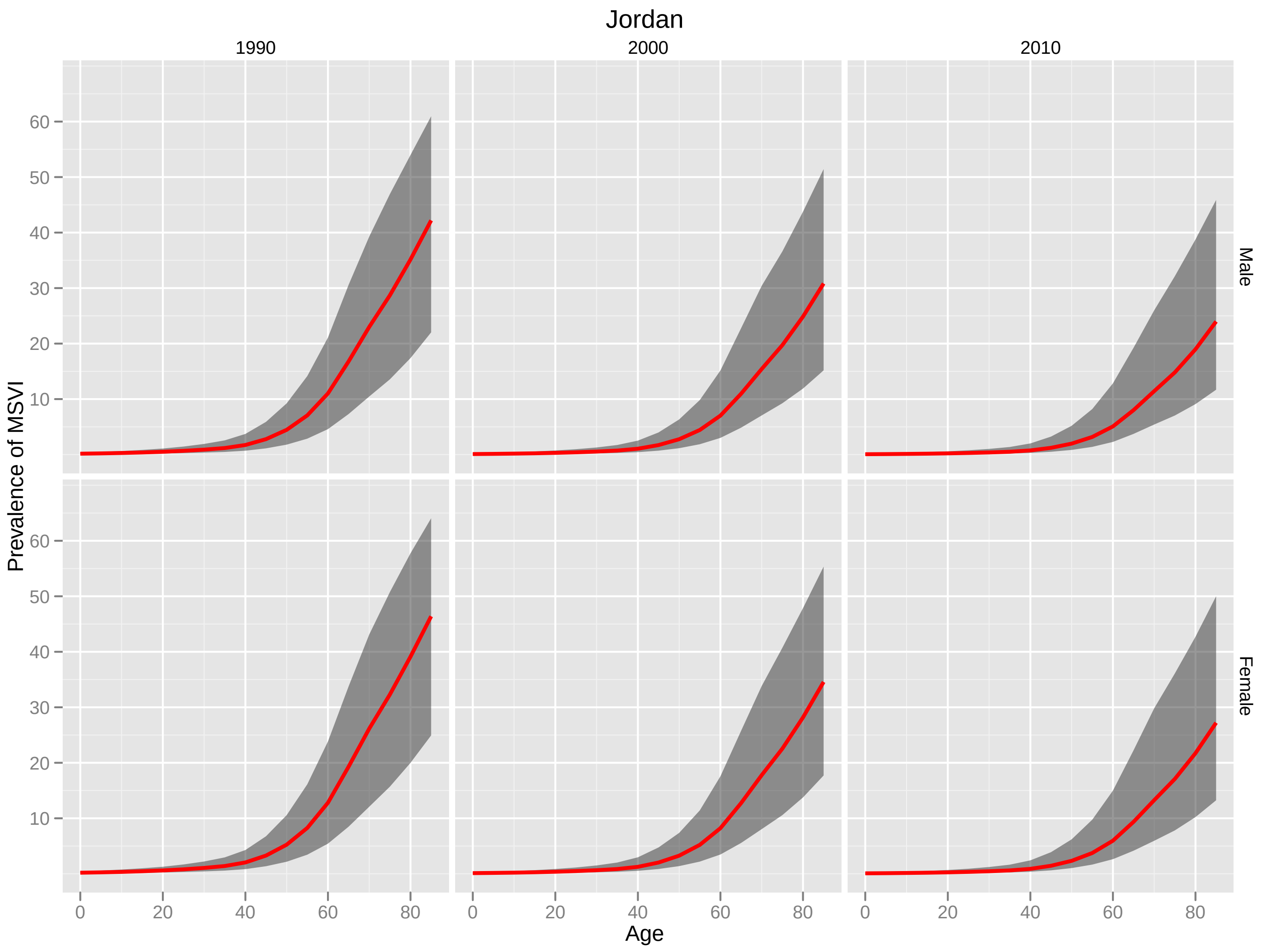

Jordan
1990
2000
2010
Male
Female
Prevalence of MSVI
Age
10
20
30
40
50
60
0
20
40
60
80

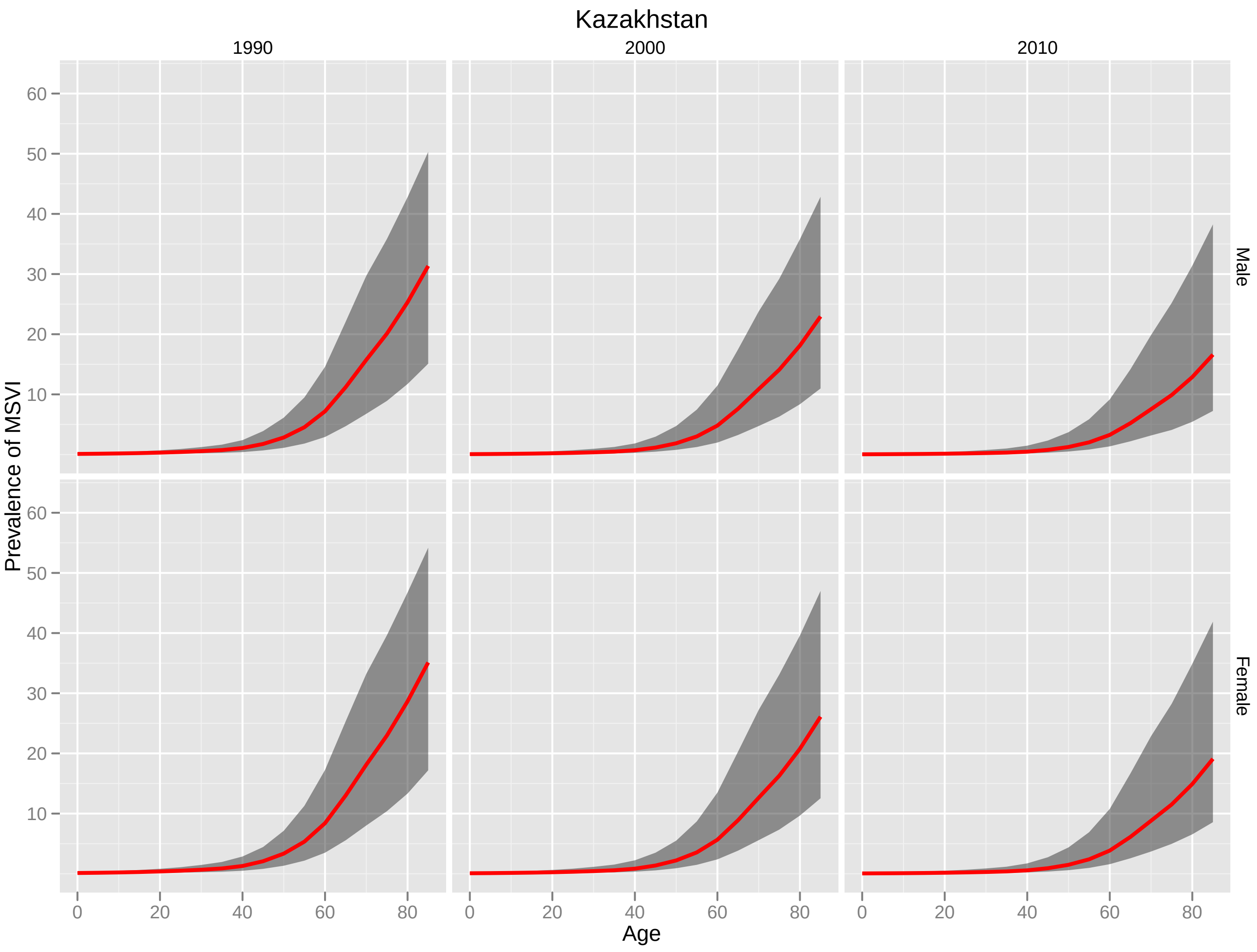
Kazakhstan
1990
2000
2010
Male
Female
Prevalence of MSVI
Age
0
20
40
60
80
10
20
30
40
50
60

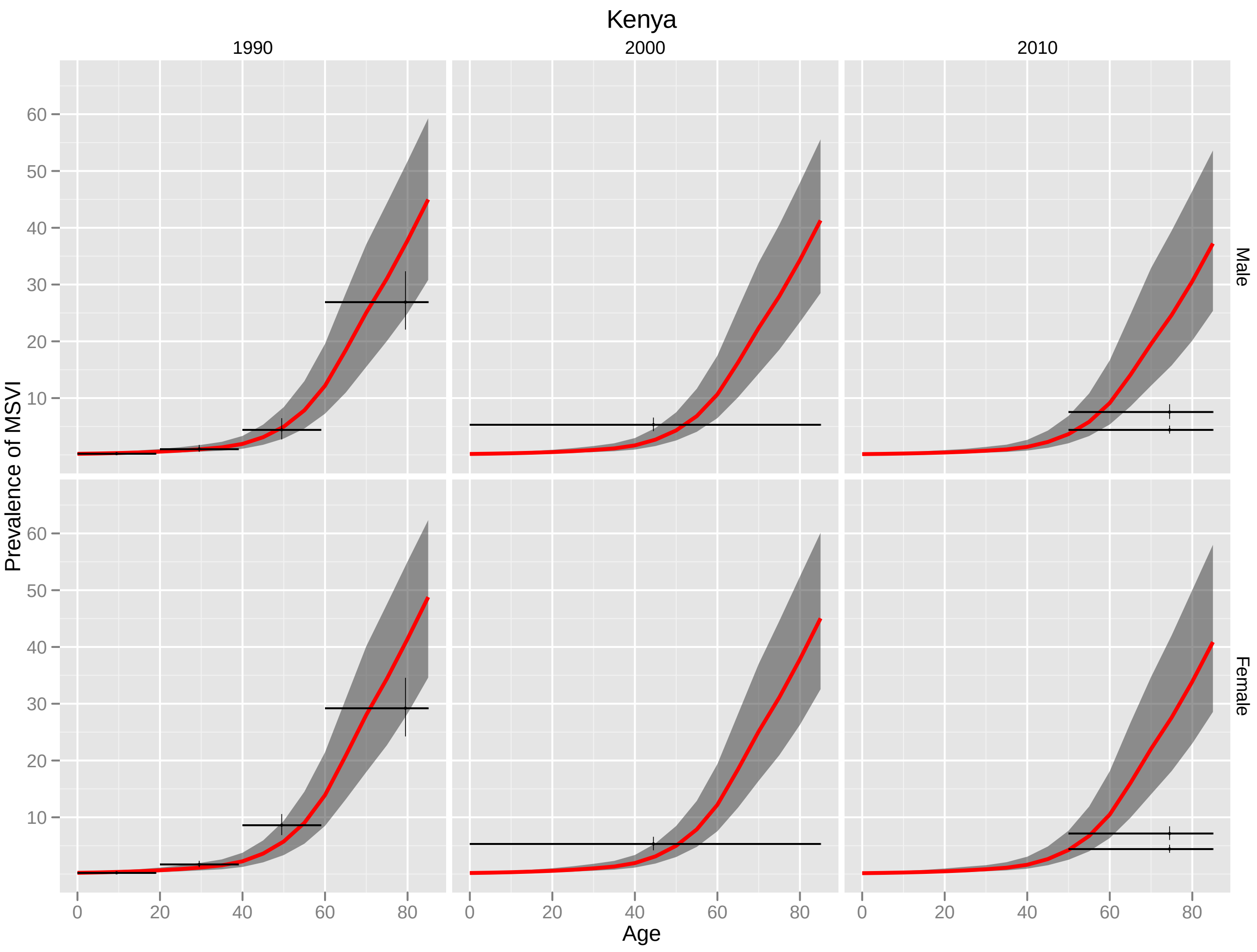
Kenya
1990
2000
2010
Male
Female
Prevalence of MSVI
Age
10
20
30
40
50
60
0
20
40
60
80

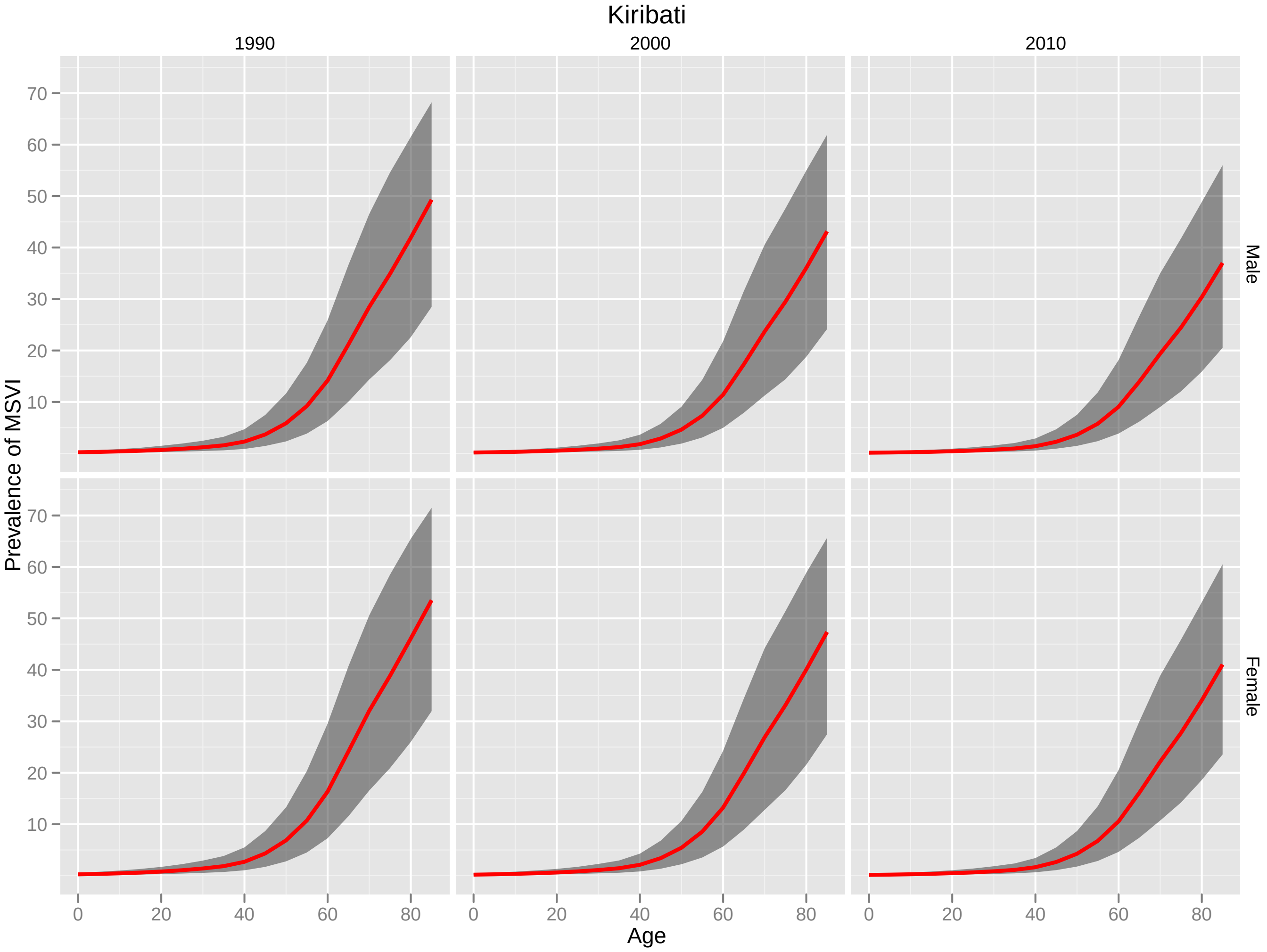

Kiribati
1990
2000
2010
Male
Female
Prevalence of MSVI
Age
0
20
40
60
80
10
20
30
40
50
60
70

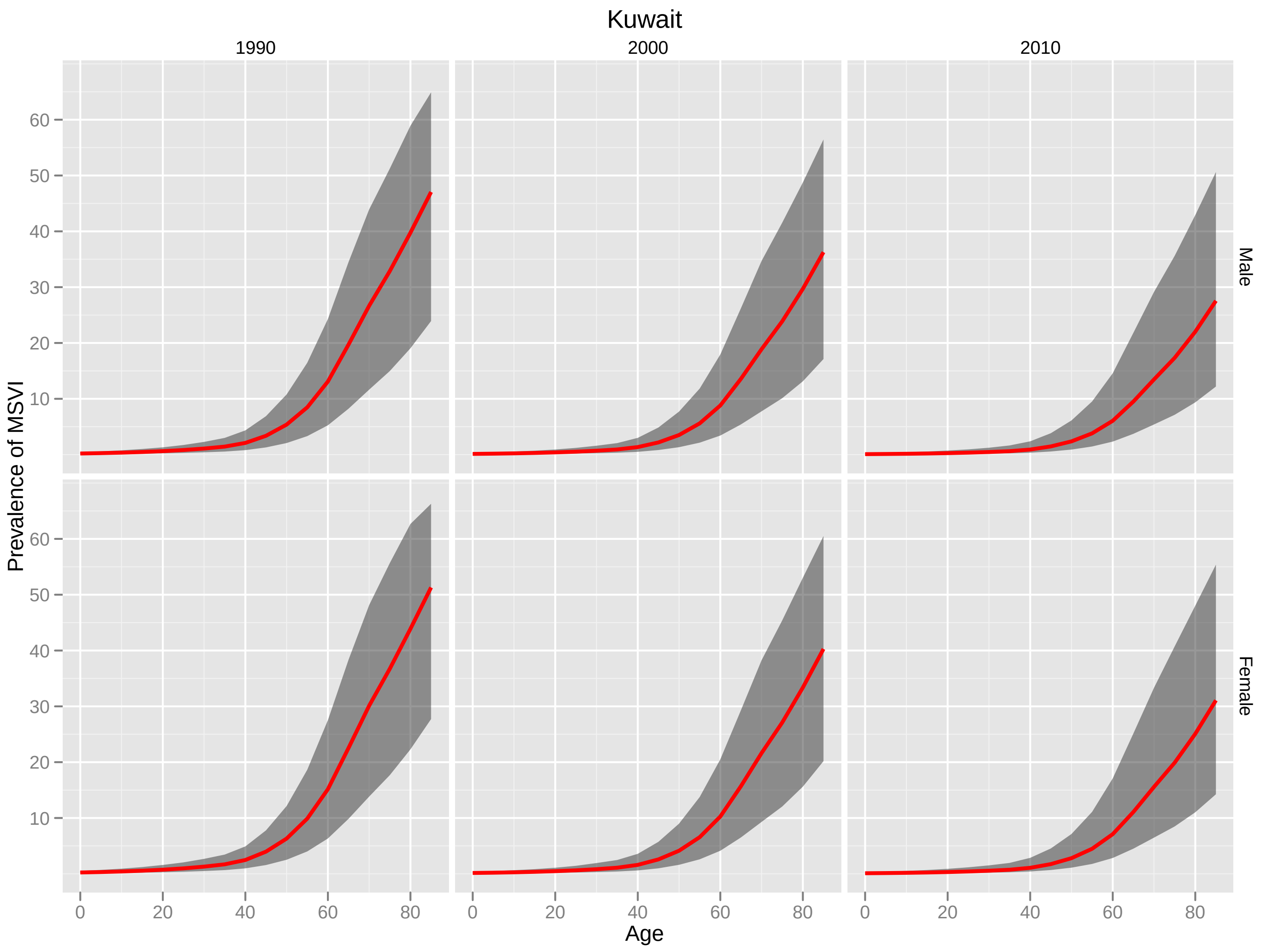

Kuwait
1990
2000
2010
Male
Female
Prevalence of MSVI
Age
10
20
30
40
50
60
0
20
40
60
80

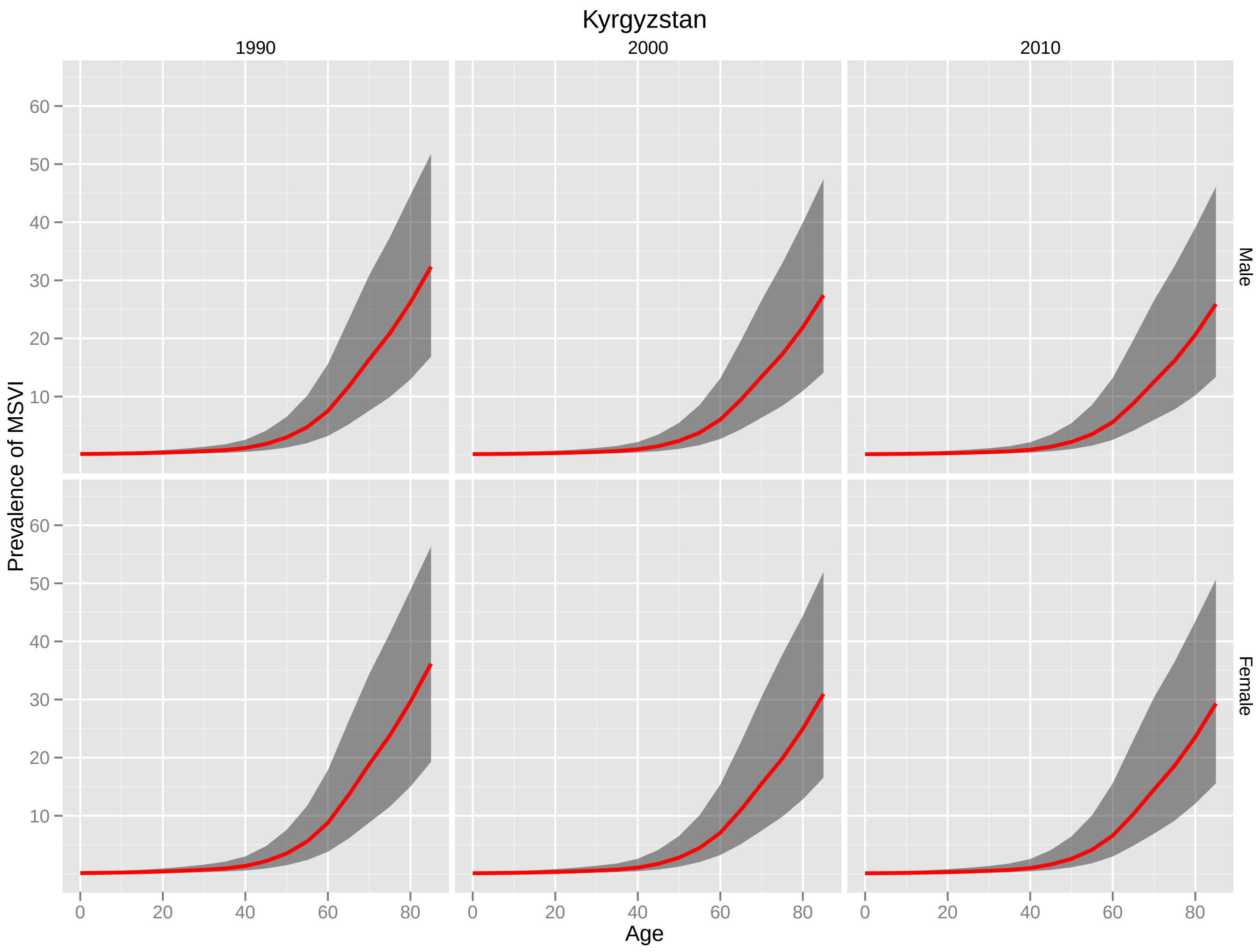

Kyrgyzstan
1990
2000
2010
Male
Female
Prevalence of MSVI
Age
10
20
30
40
50
60
0
20
40
60
80

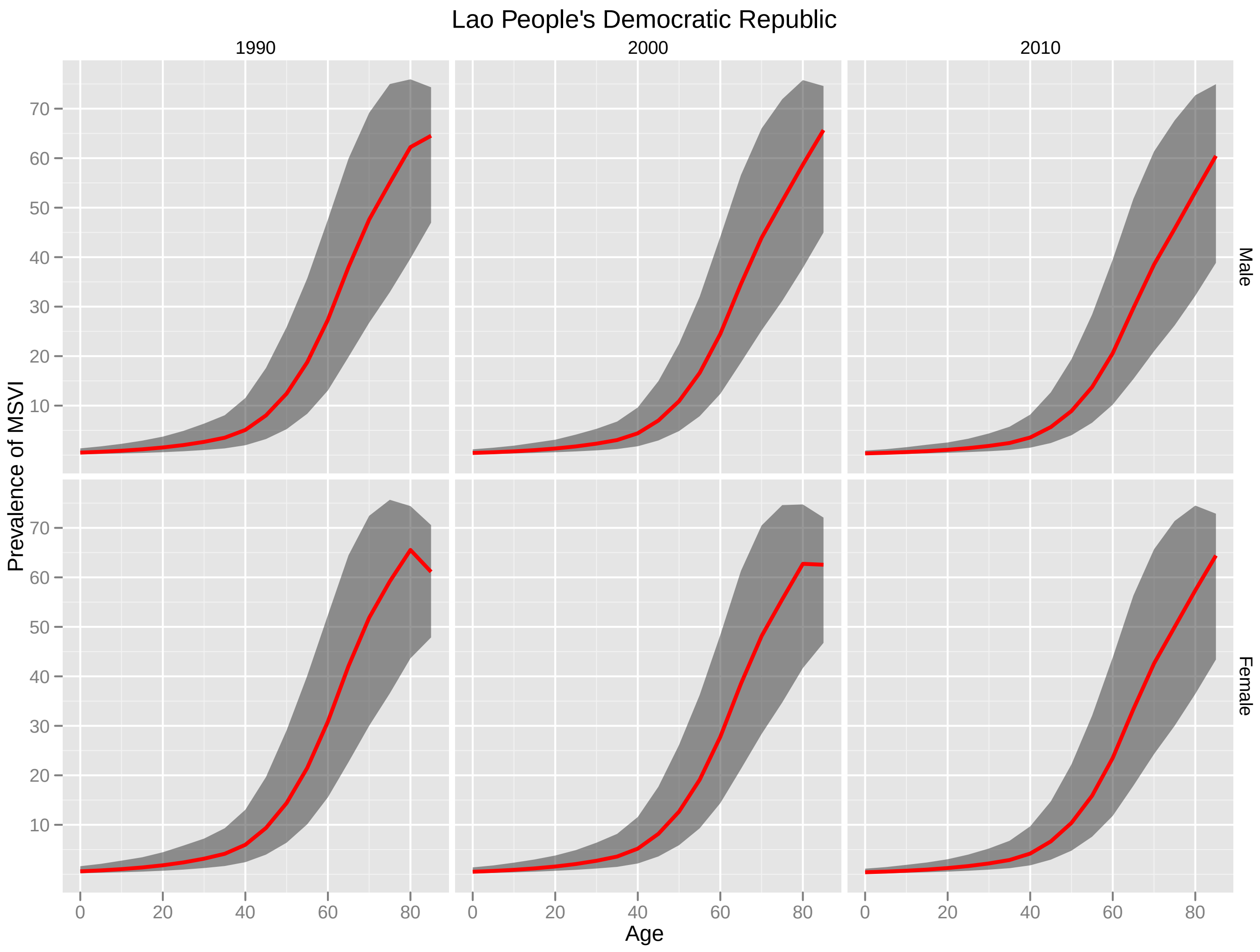
Lao People's Democratic Republic
1990
2000
2010
Male
Female
Prevalence of MSVI
Age
10
20
30
40
50
60
70
0
20
40
60
80

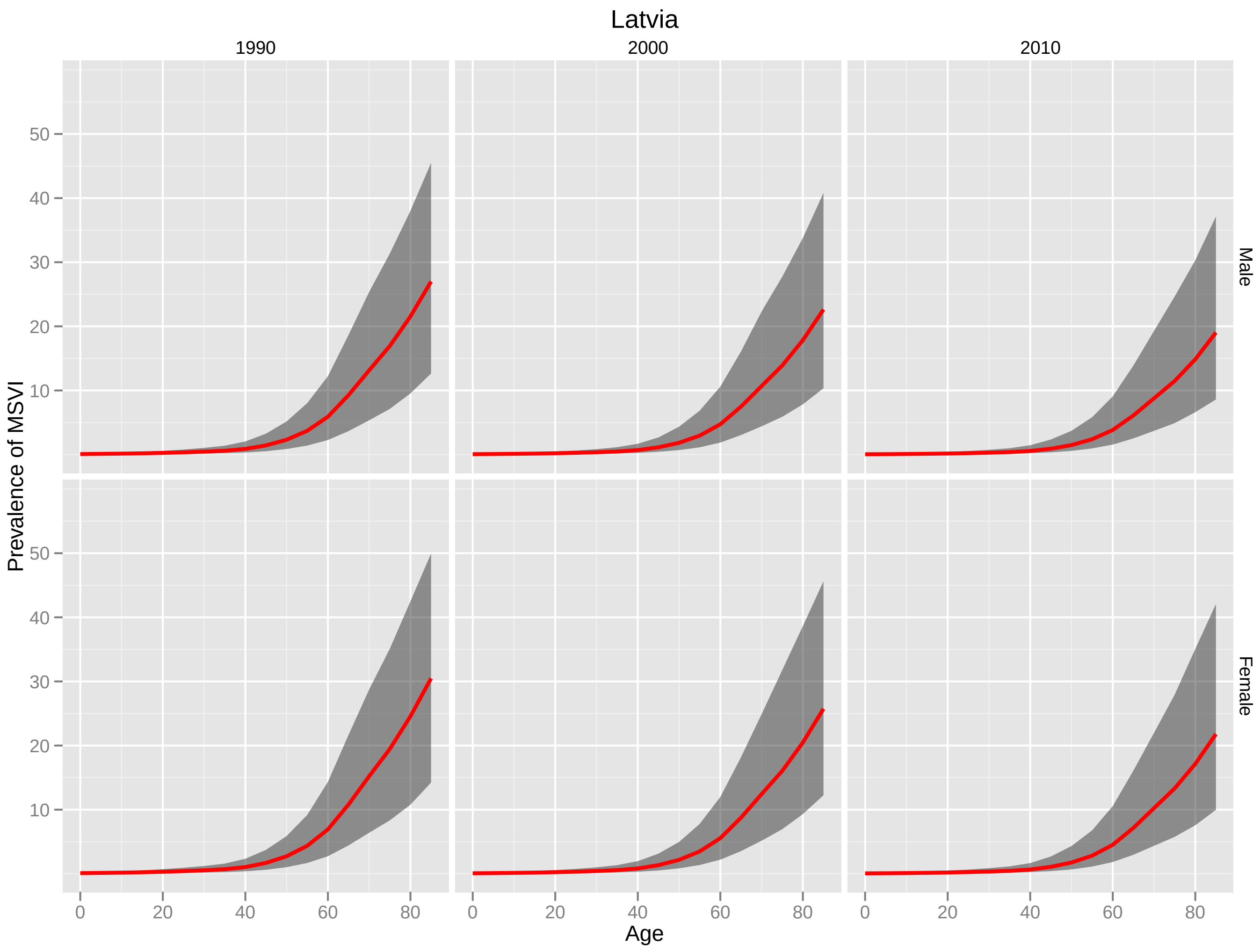
Latvia
1990
2000
2010
Male
Female
Prevalence of MSVI
Age
10
20
30
40
50
0
20
40
60
80

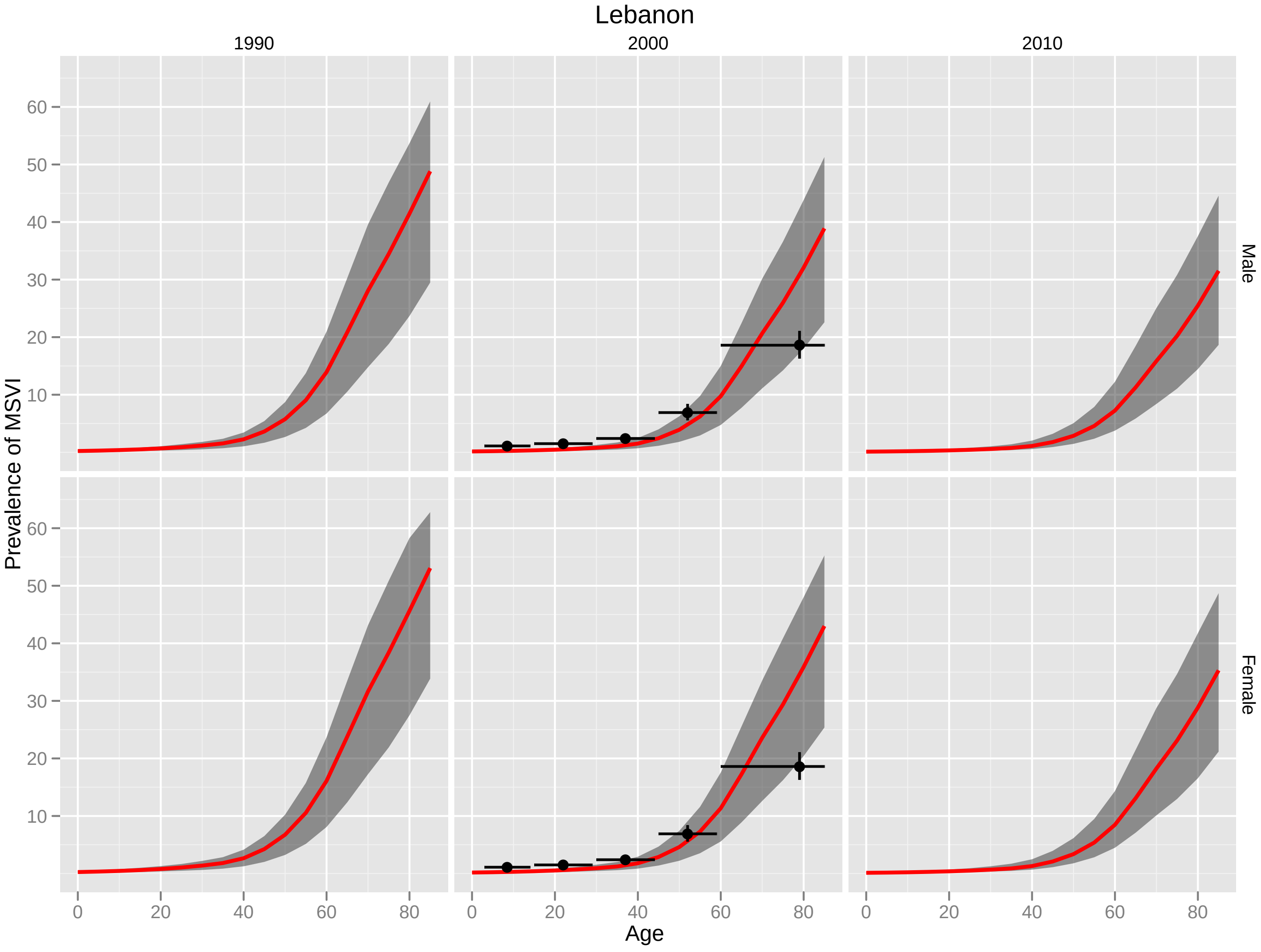
Lebanon
1990
2000
2010
Male
Female
Prevalence of MSVI
Age
10
20
30
40
50
60
0
20
40
60
80

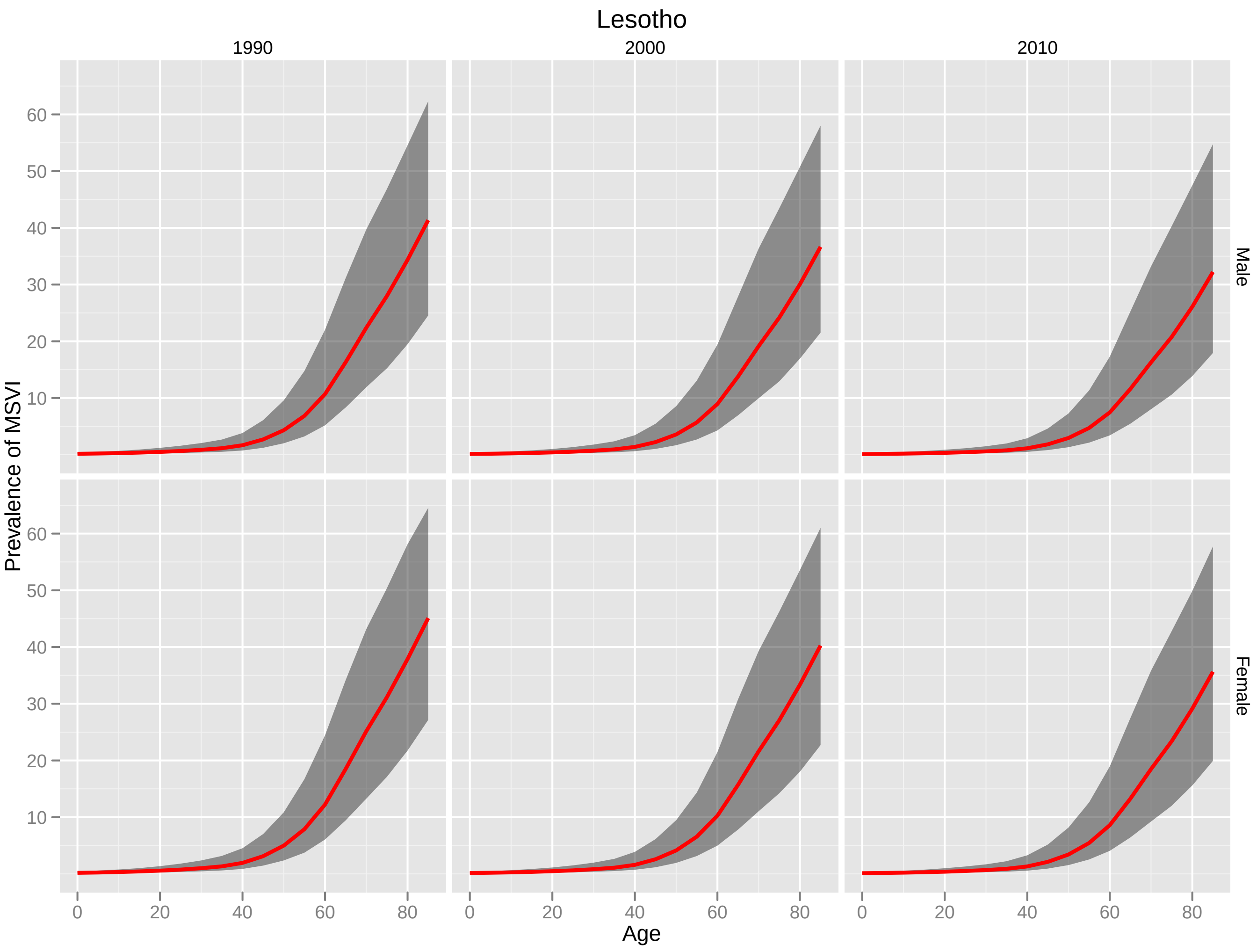

Lesotho
1990
2000
2010
Male
Female
Prevalence of MSVI
Age
10
20
30
40
50
60
0
20
40
60
80

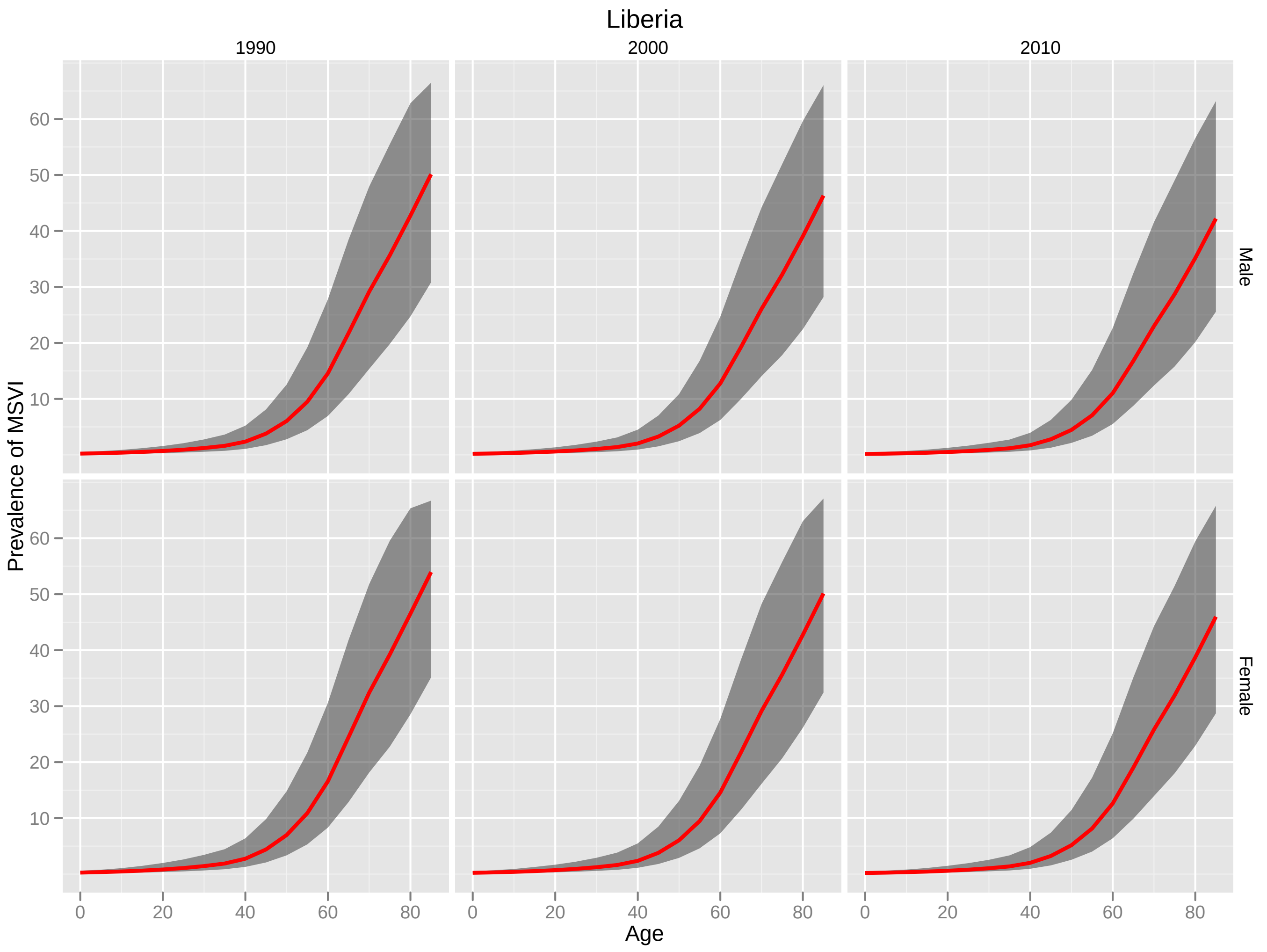
Liberia
1990
2000
2010
Male
Female
Prevalence of MSVI
Age
10
20
30
40
50
60
0
20
40
60
80

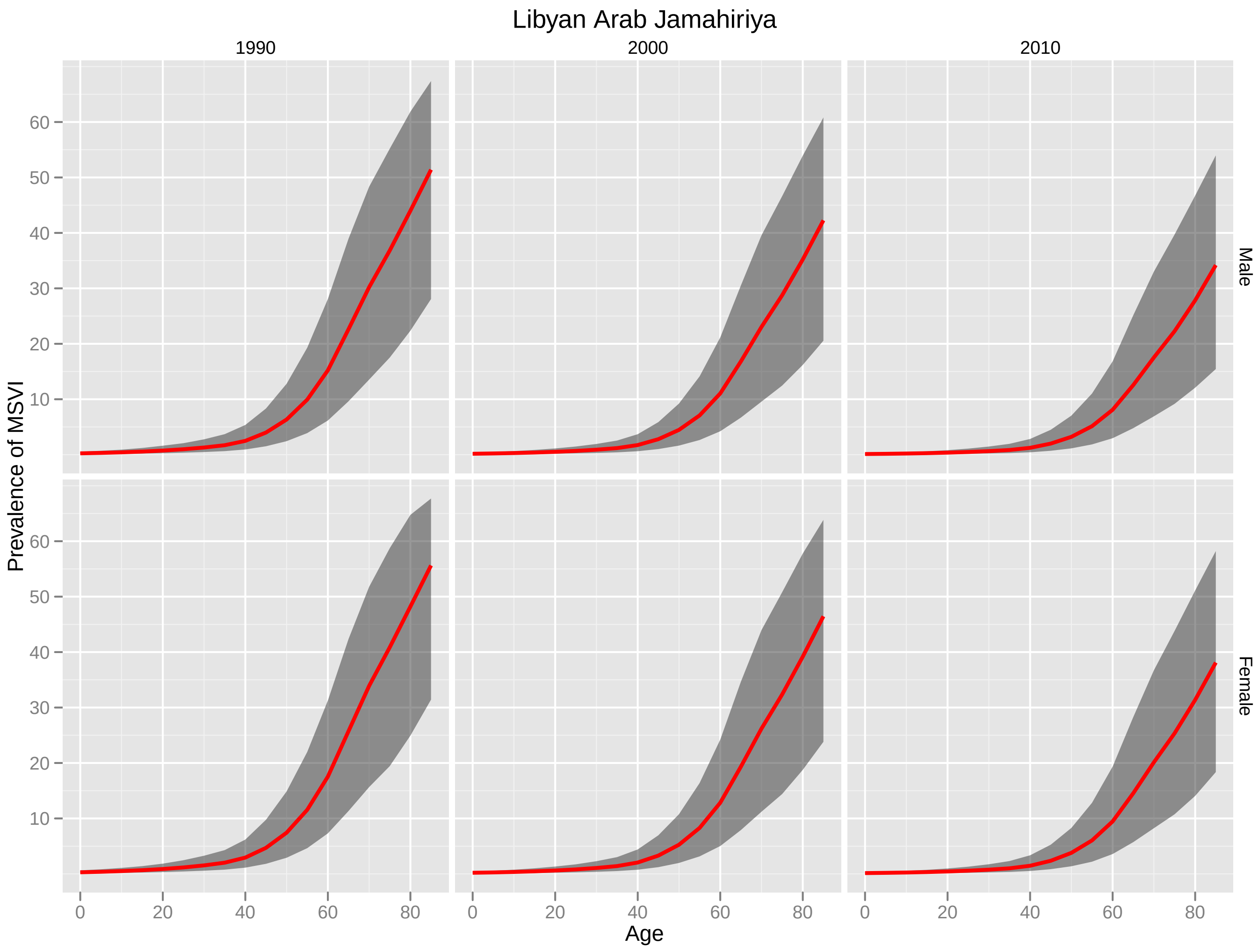
Libyan Arab Jamahiriya
1990
2000
2010
Male
Female
Prevalence of MSVI
Age
10
20
30
40
50
60
0
20
40
60
80

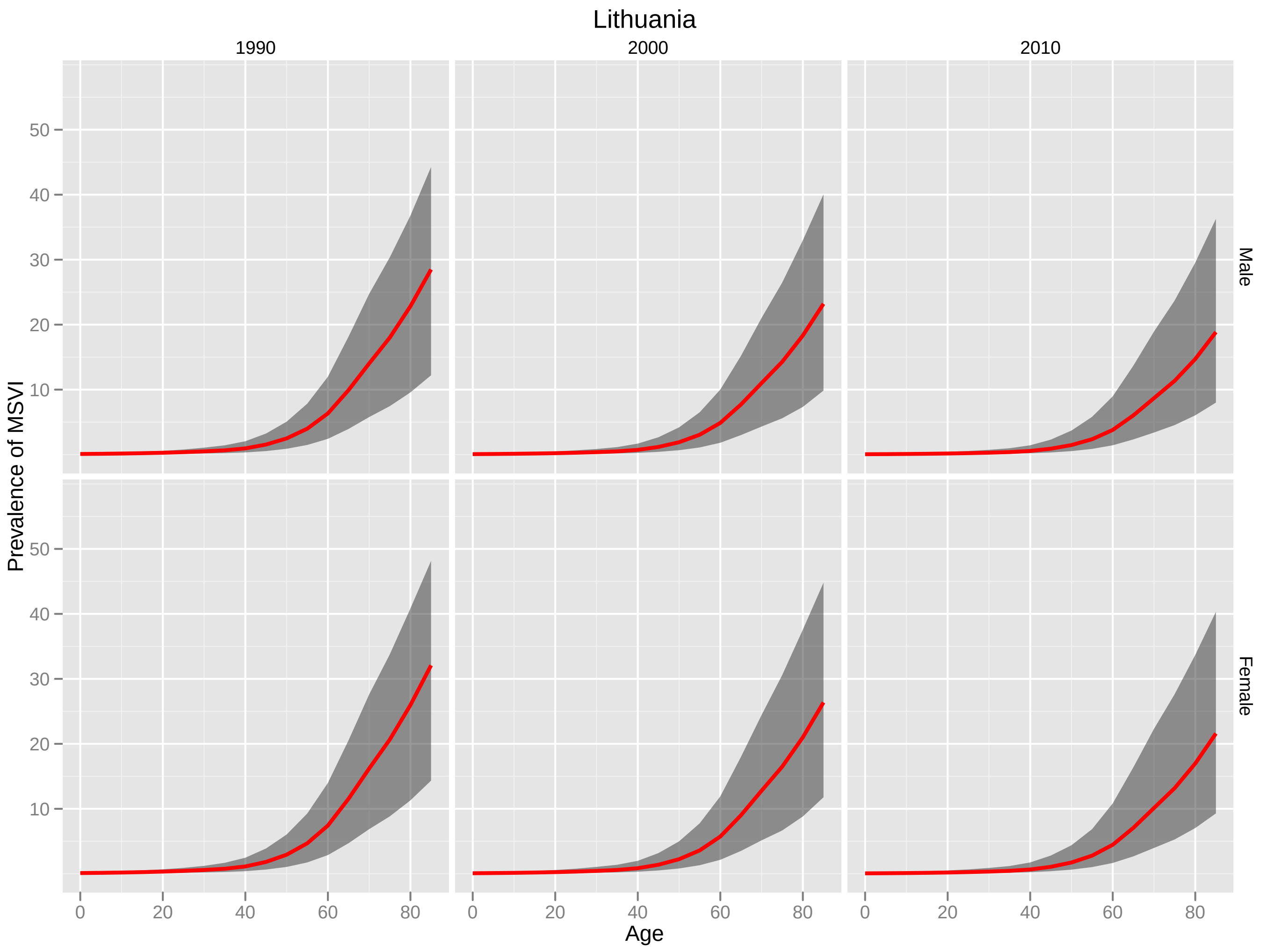
Lithuania
1990
2000
2010
Male
Female
Prevalence of MSVI
Age
10
20
30
40
50
0
20
40
60
80

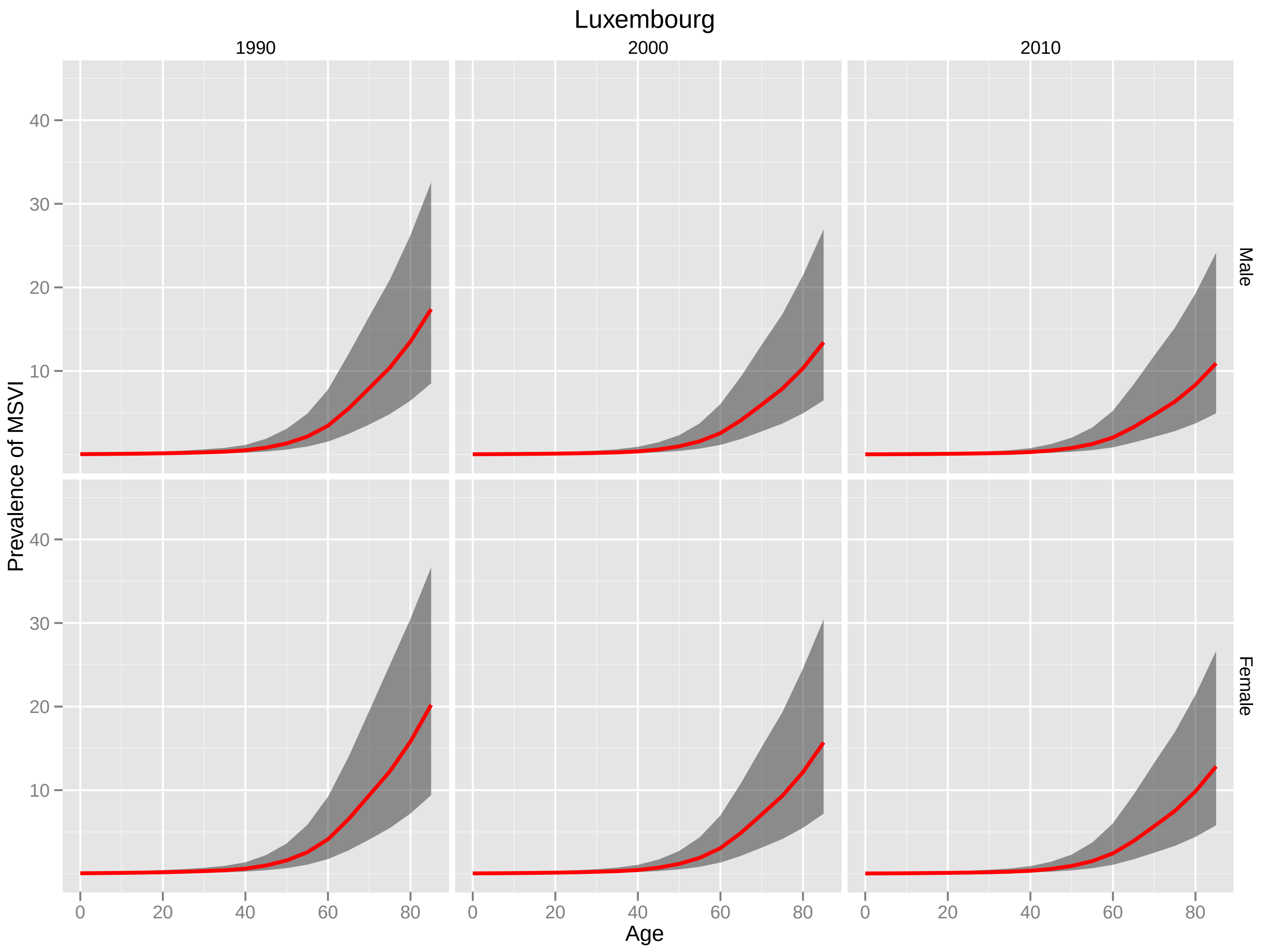

Luxembourg
1990
2000
2010
Male
Female
Prevalence of MSVI
10
20
30
40
0
20
40
60
80
Age

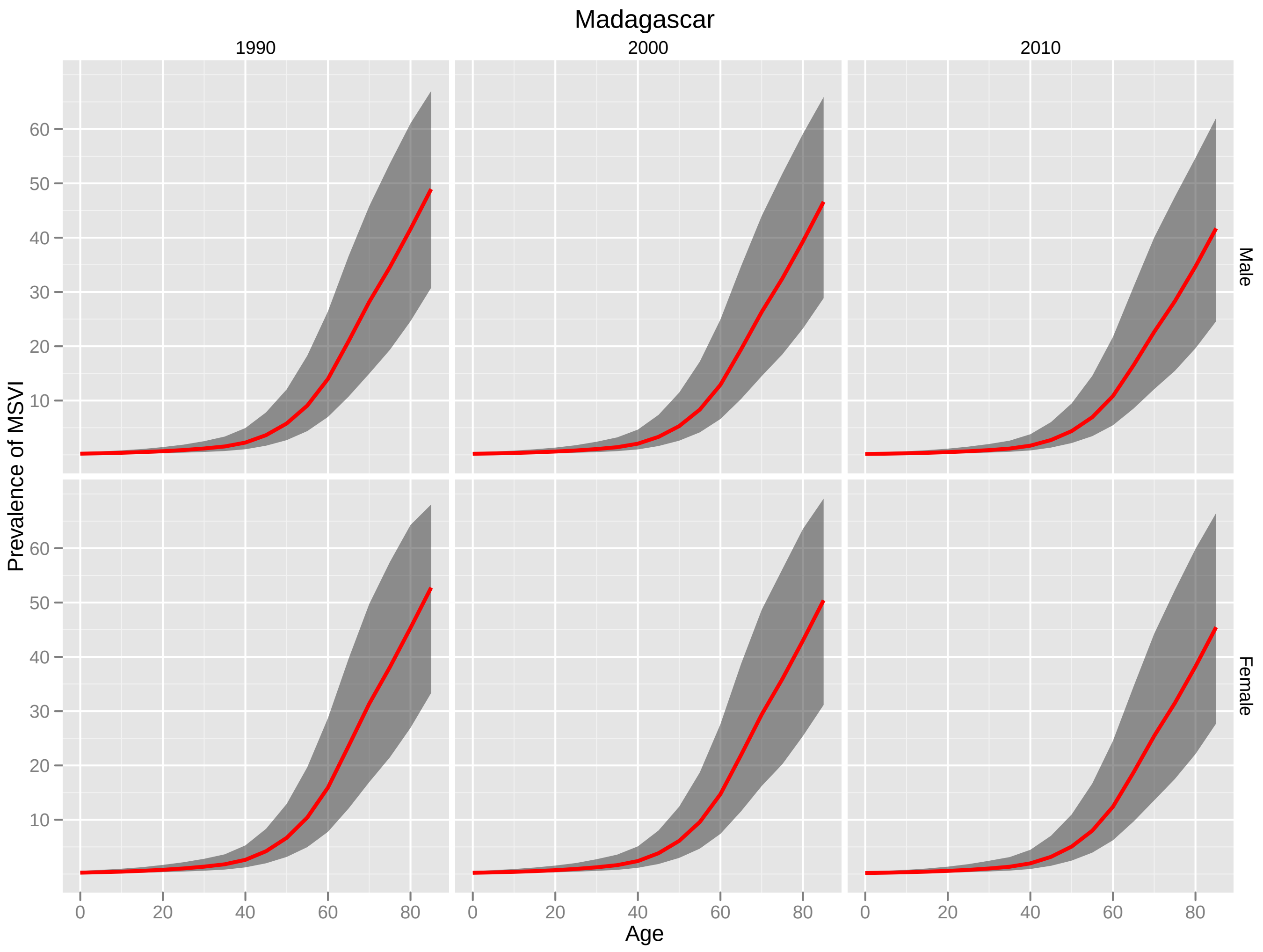
Madagascar
1990
2000
2010
Male
Female
Prevalence of MSVI
10
20
30
40
50
60
0
20
40
60
80
Age

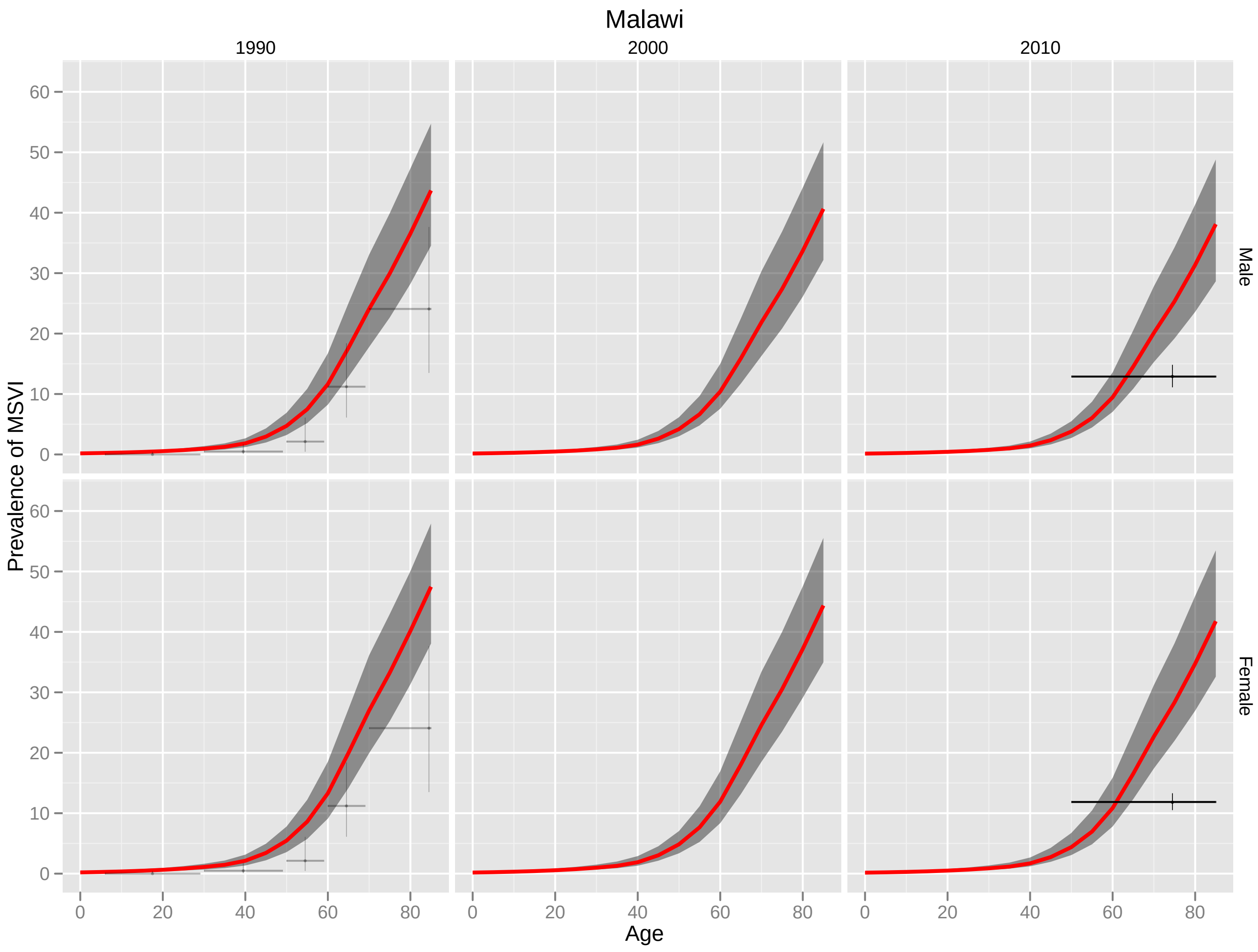
Malawi
1990
2000
2010
Male
Female
Prevalence of MSVI
Age
0
10
20
30
40
50
60
80

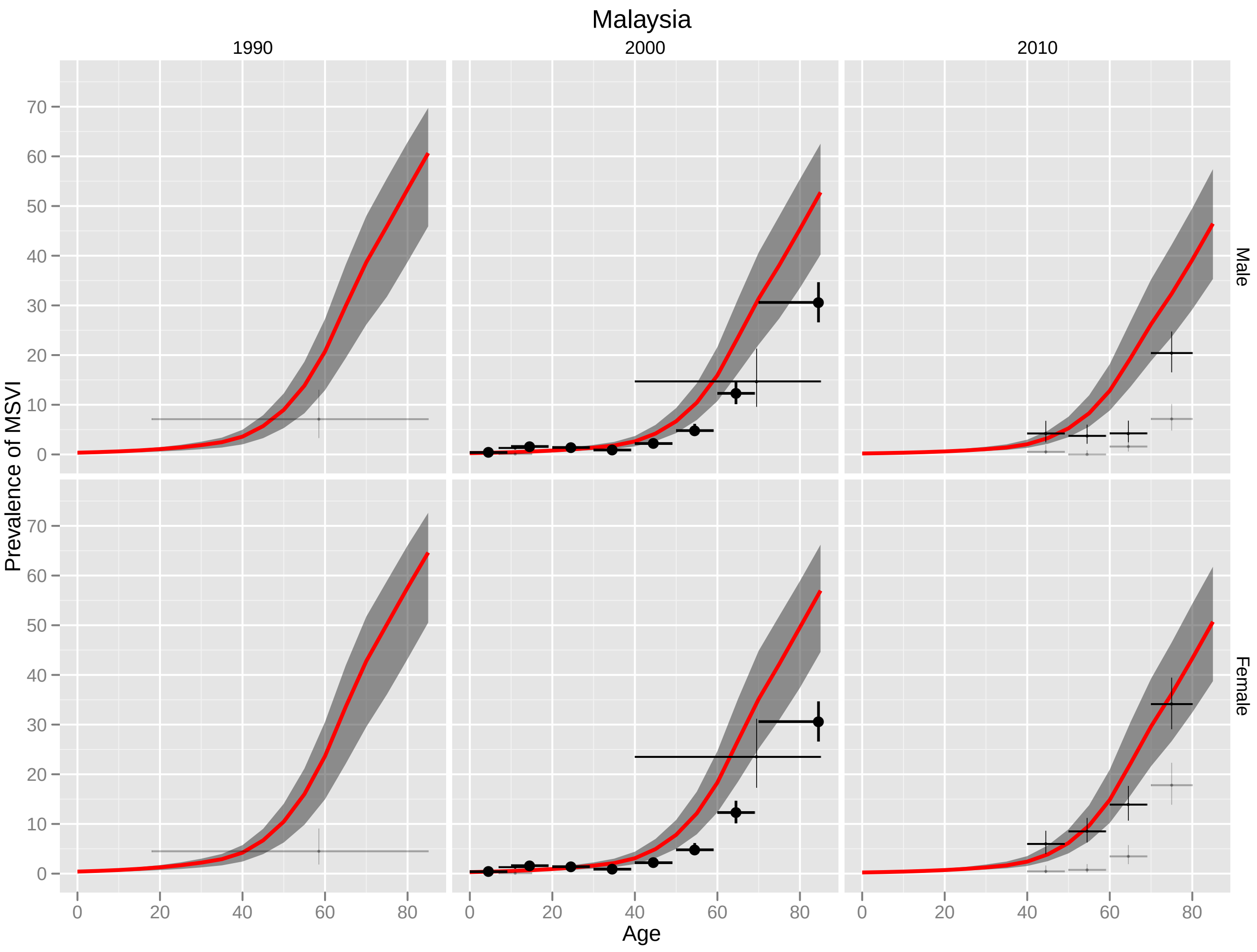
Malaysia
1990
2000
2010
Male
Female
Prevalence of MSVI
Age
0
10
20
30
40
50
60
70
80

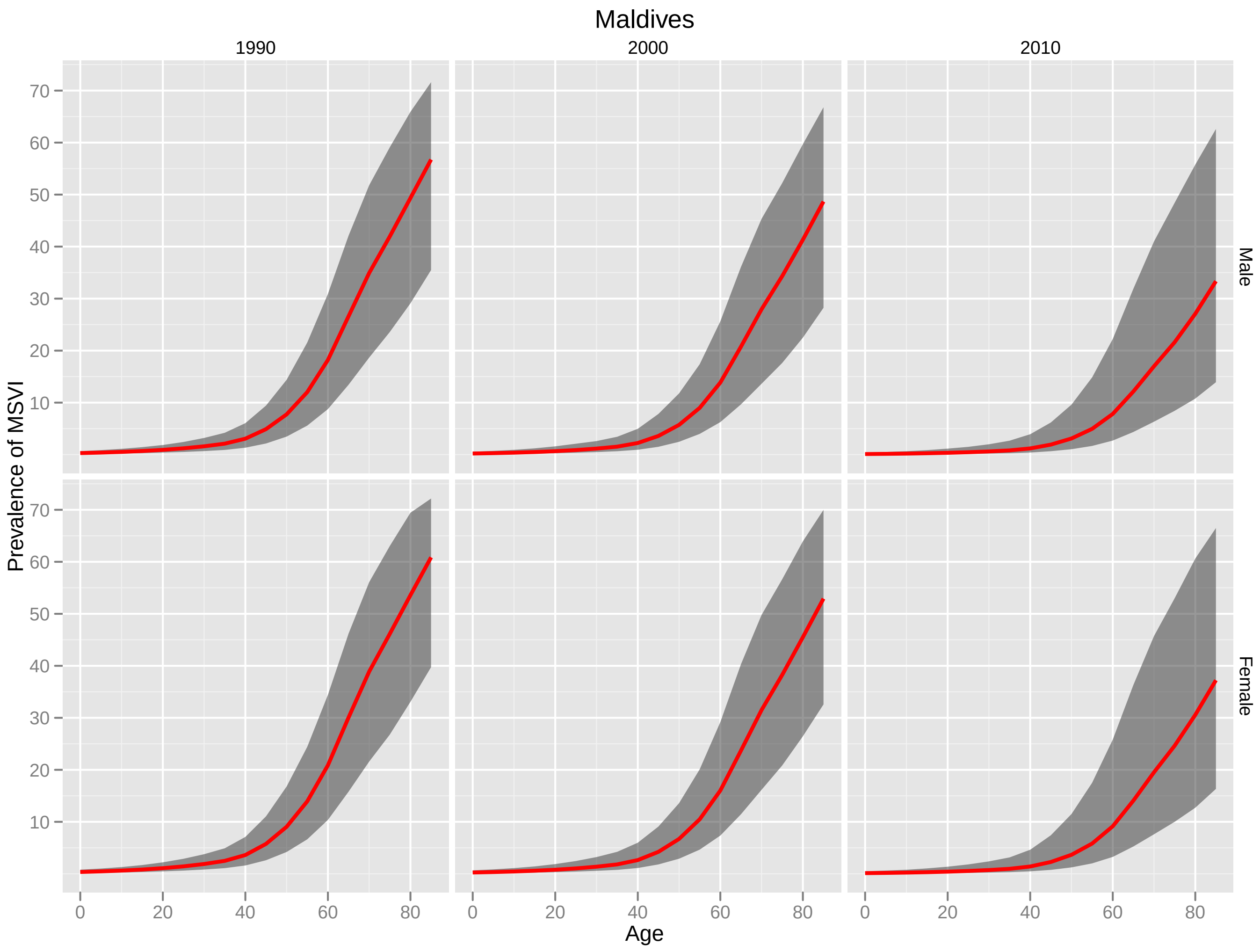

Maldives
1990
2000
2010
Male
Female
Prevalence of MSVI
Age
70
60
50
40
30
20
10
0
20
40
60
80

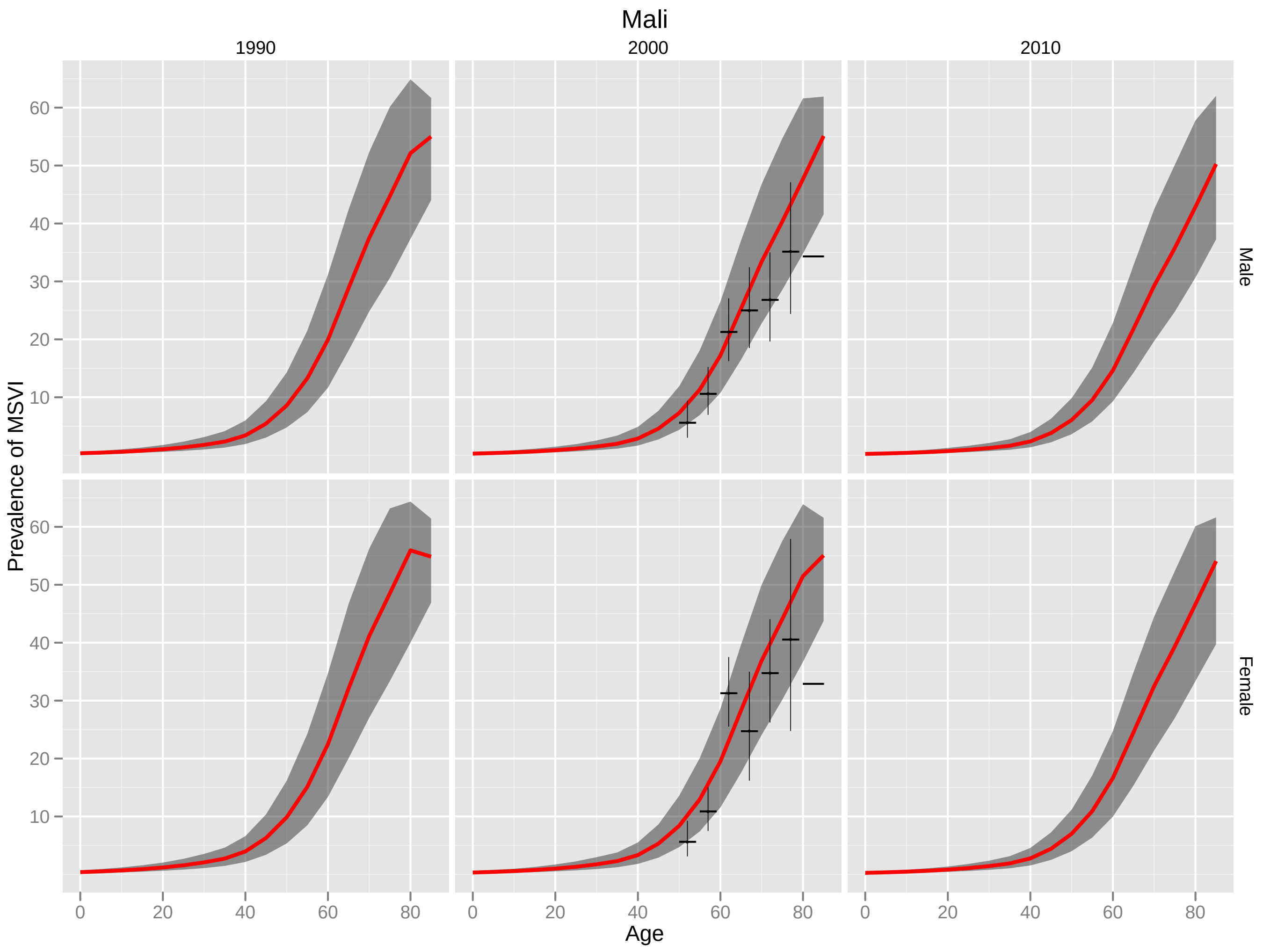

Mali
1990
2000
2010
Male
Female
Prevalence of MSVI
Age
10
20
30
40
50
60
0
20
40
60
80

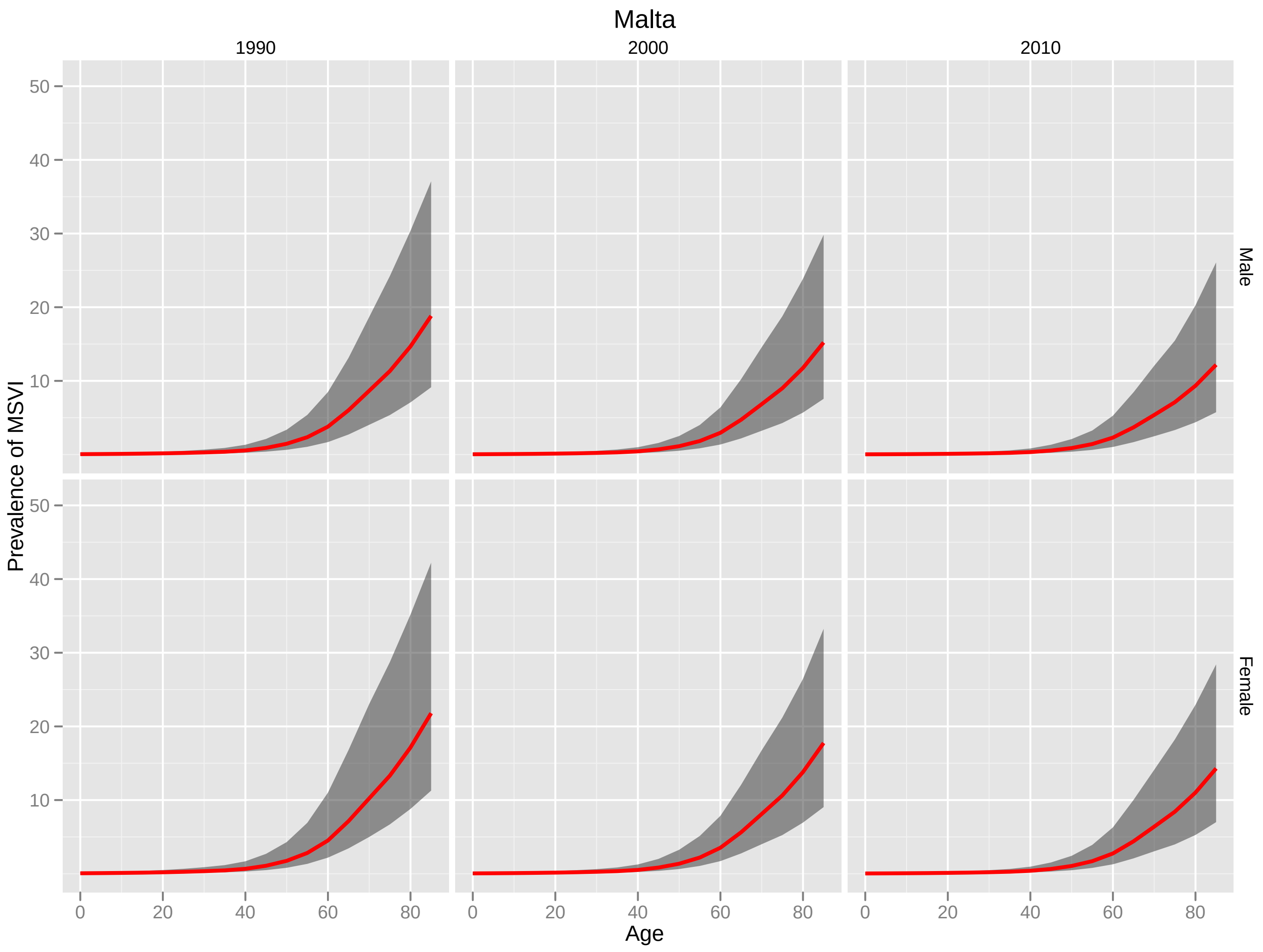

Malta
1990
2000
2010
Male
Female
Prevalence of MSVI
Age
0
10
20
30
40
50
60
80

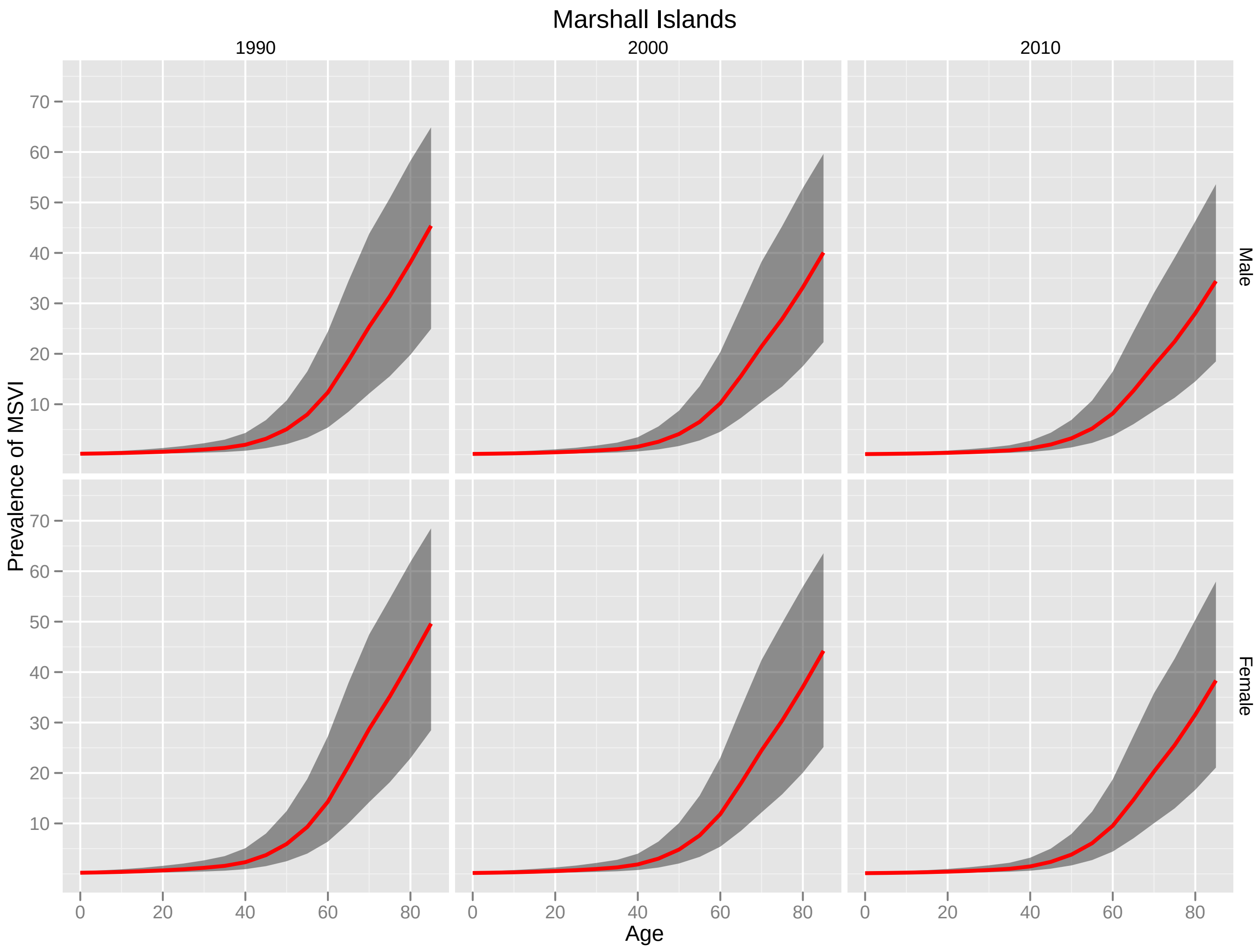

Marshall Islands
1990
2000
2010
Male
Female
Prevalence of MSVI
Age
0
20
40
60
80
10
20
30
40
50
60
70

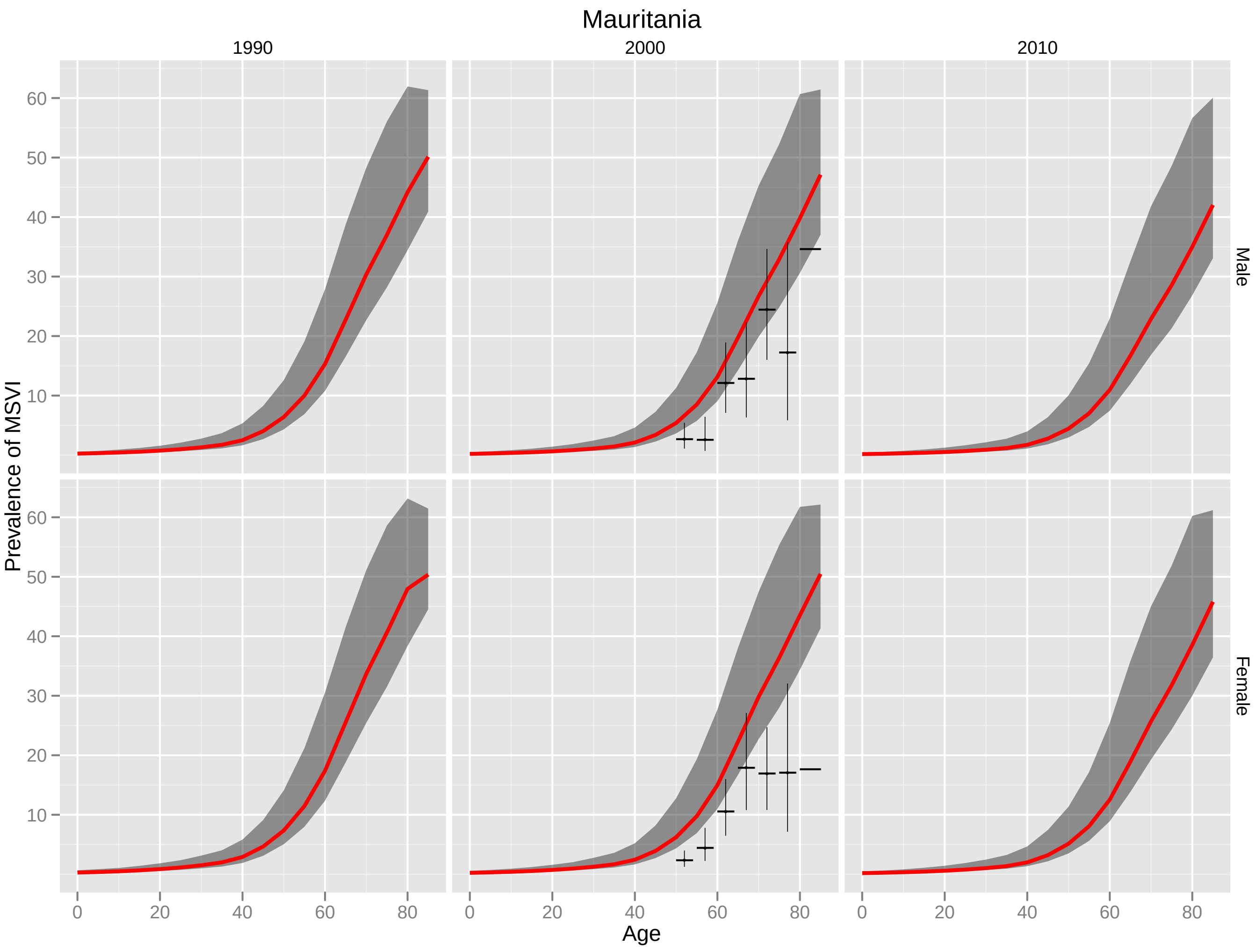
Mauritania
1990
2000
2010
Male
Female
Prevalence of MSVI
10
20
30
40
50
60
Age
0
20
40
60
80

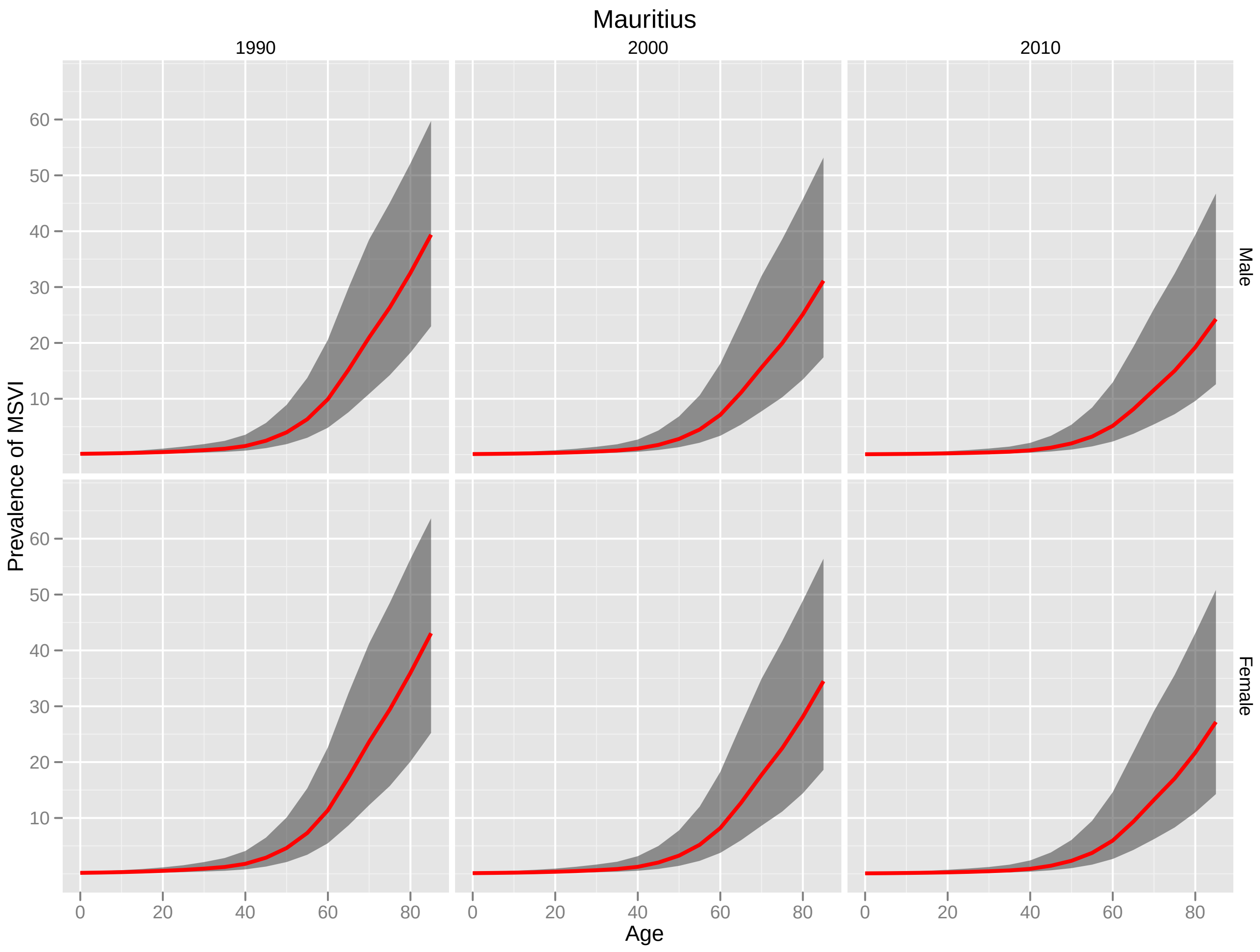

Mauritius
1990
2000
2010
Male
Female
Prevalence of MSVI
Age
0
20
40
60
80
10
20
30
40
50
60

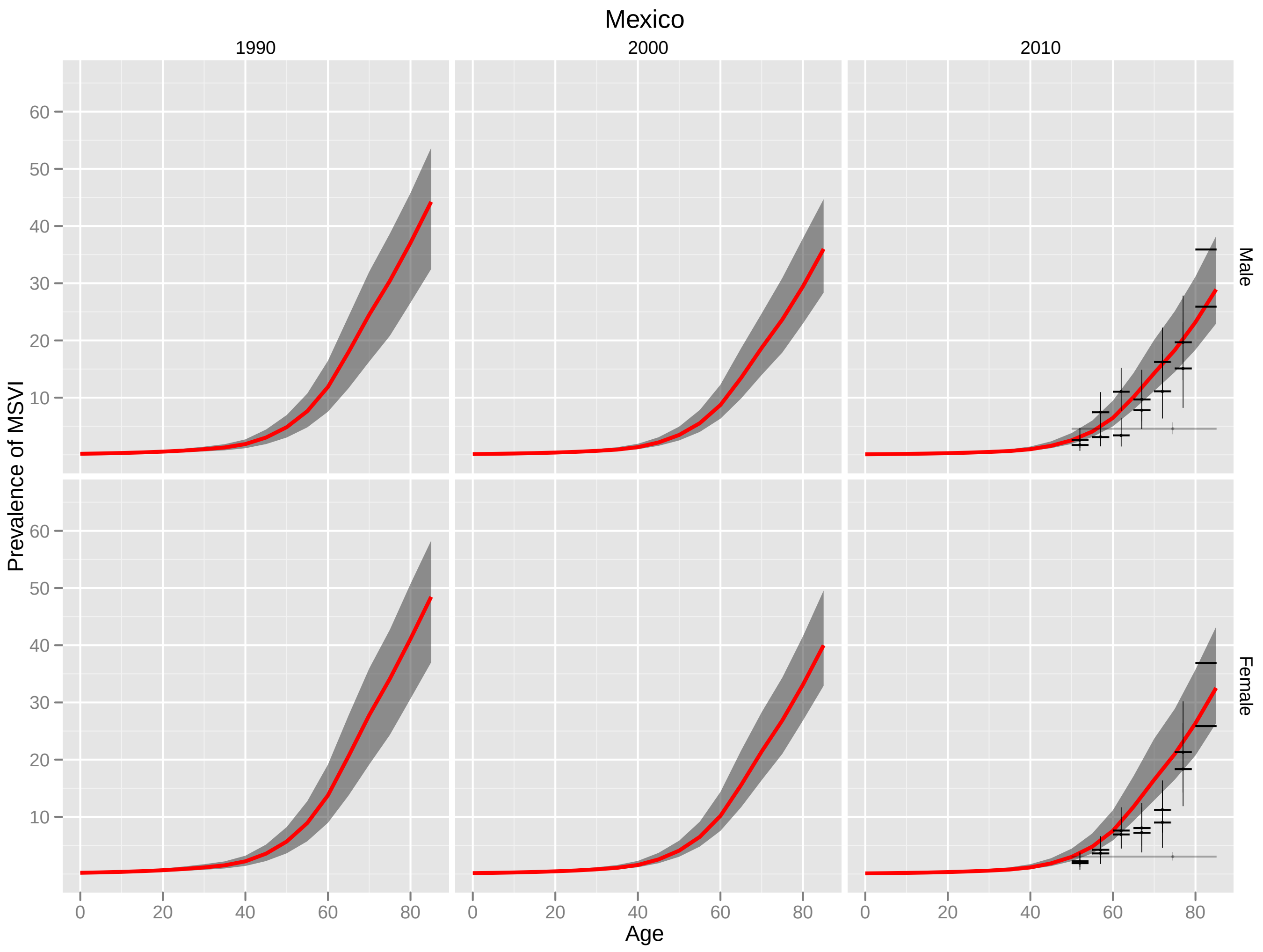
Mexico
1990
2000
2010
Male
Female
Prevalence of MSVI
Age
10
20
30
40
50
60
0
20
40
60
80

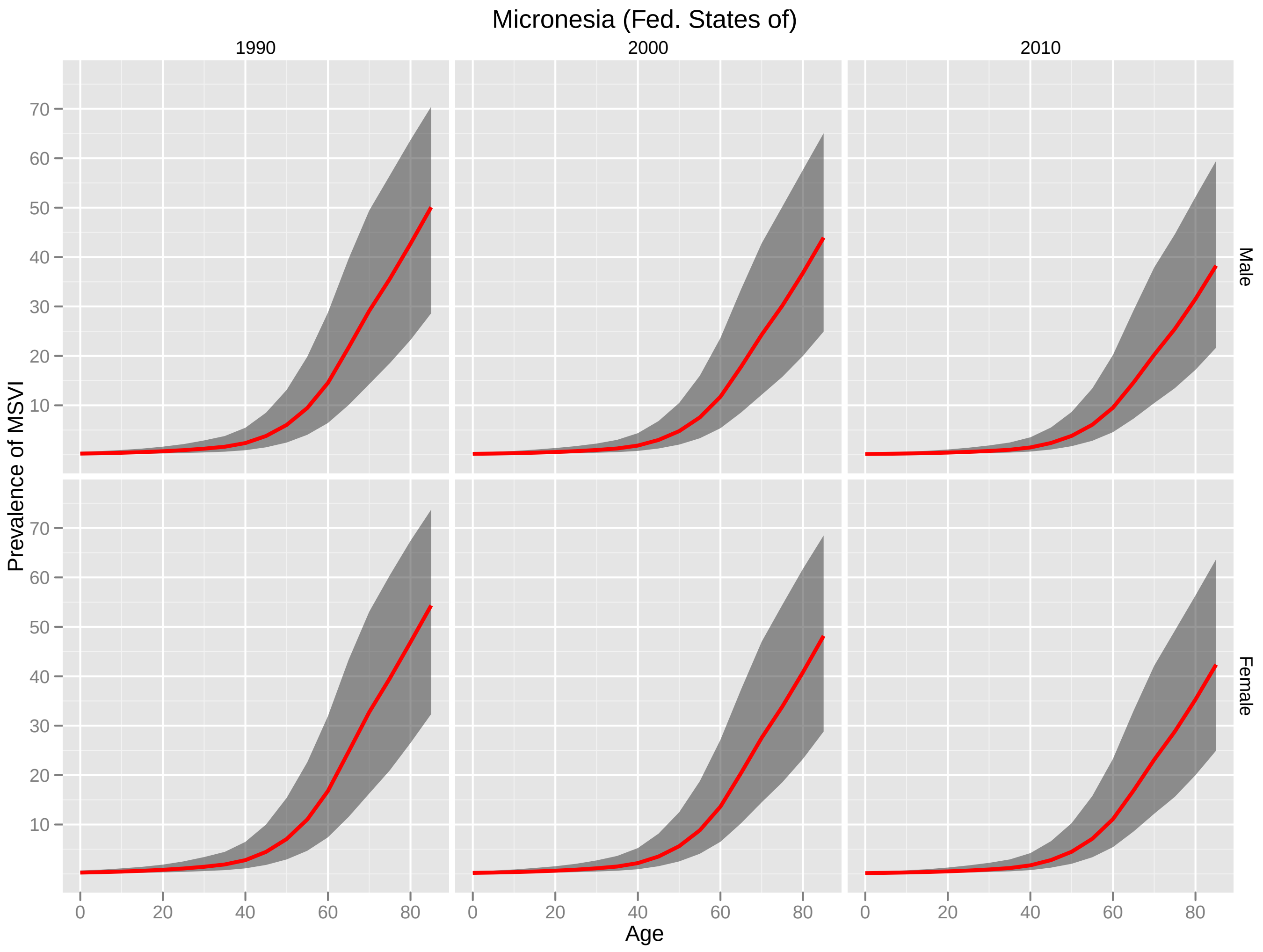
Micronesia (Fed. States of)
1990
2000
2010
Male
Female
Prevalence of MSVI
Age
10
20
30
40
50
60
70
0
20
40
60
80

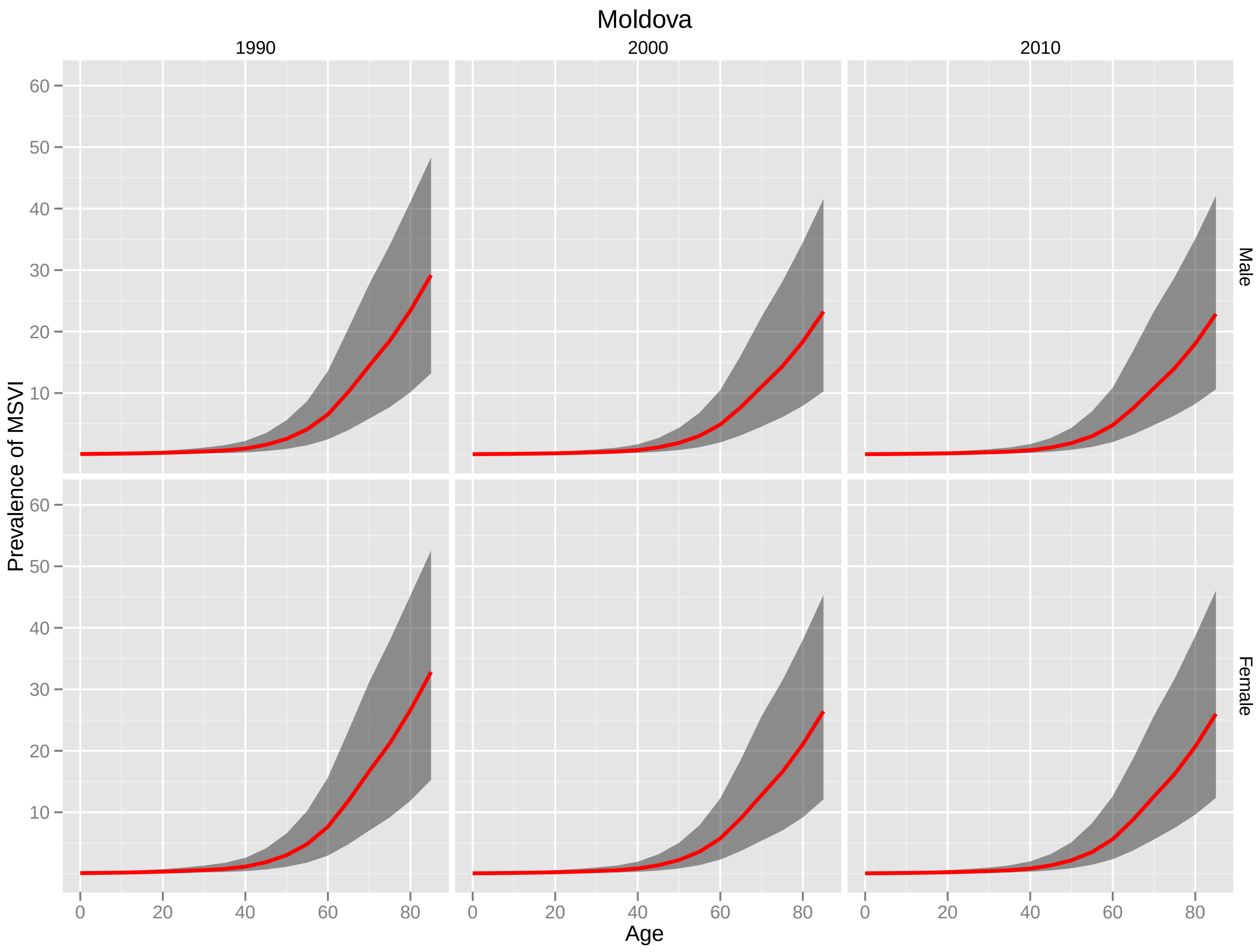
Moldova
1990
2000
2010
Male
Female
Prevalence of MSVI
Age
0
20
40
60
80
10
20
30
40
50
60

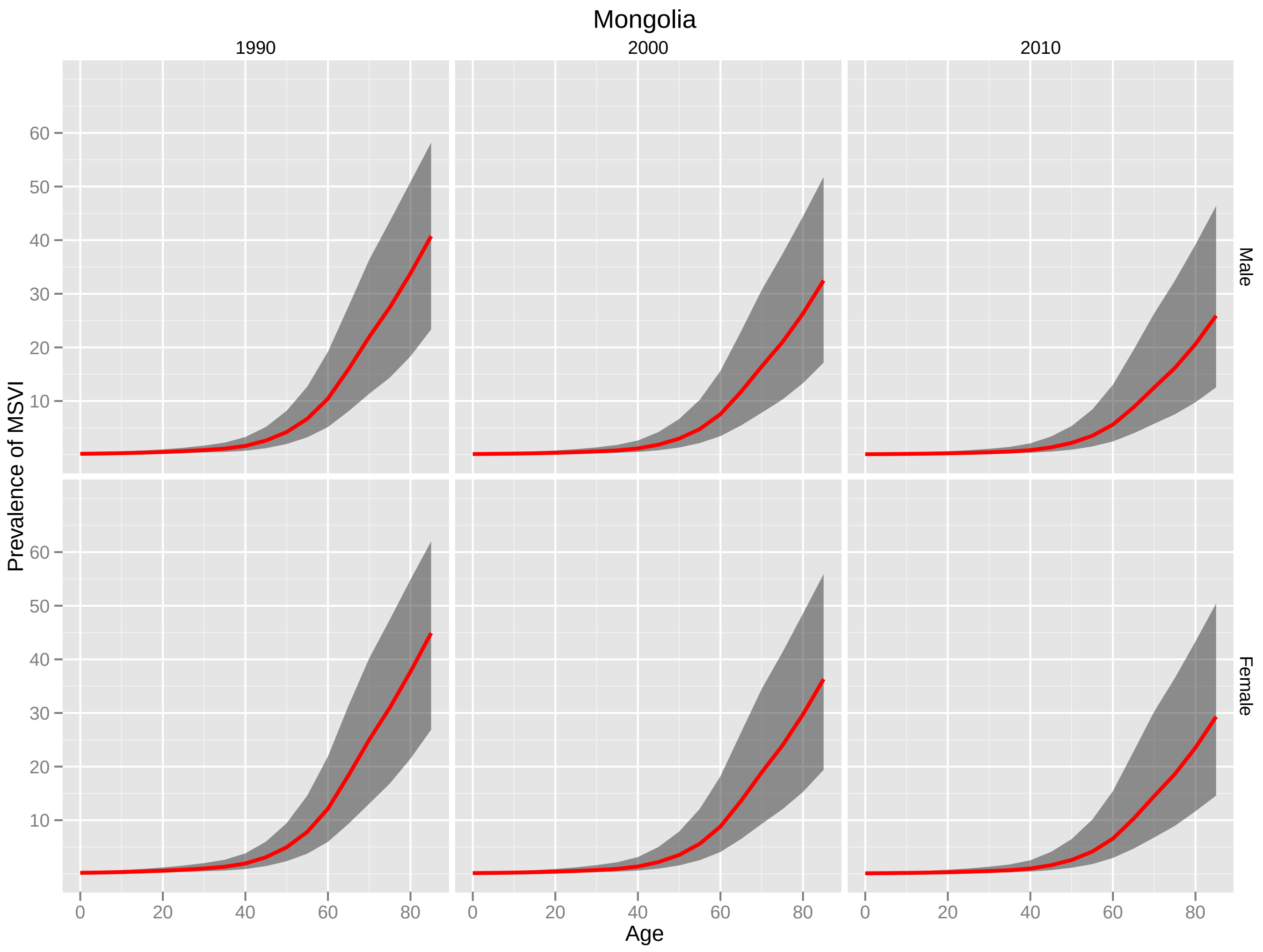
Mongolia
1990
2000
2010
Male
Female
Prevalence of MSVI
Age
10
20
30
40
50
60
0
20
40
60
80

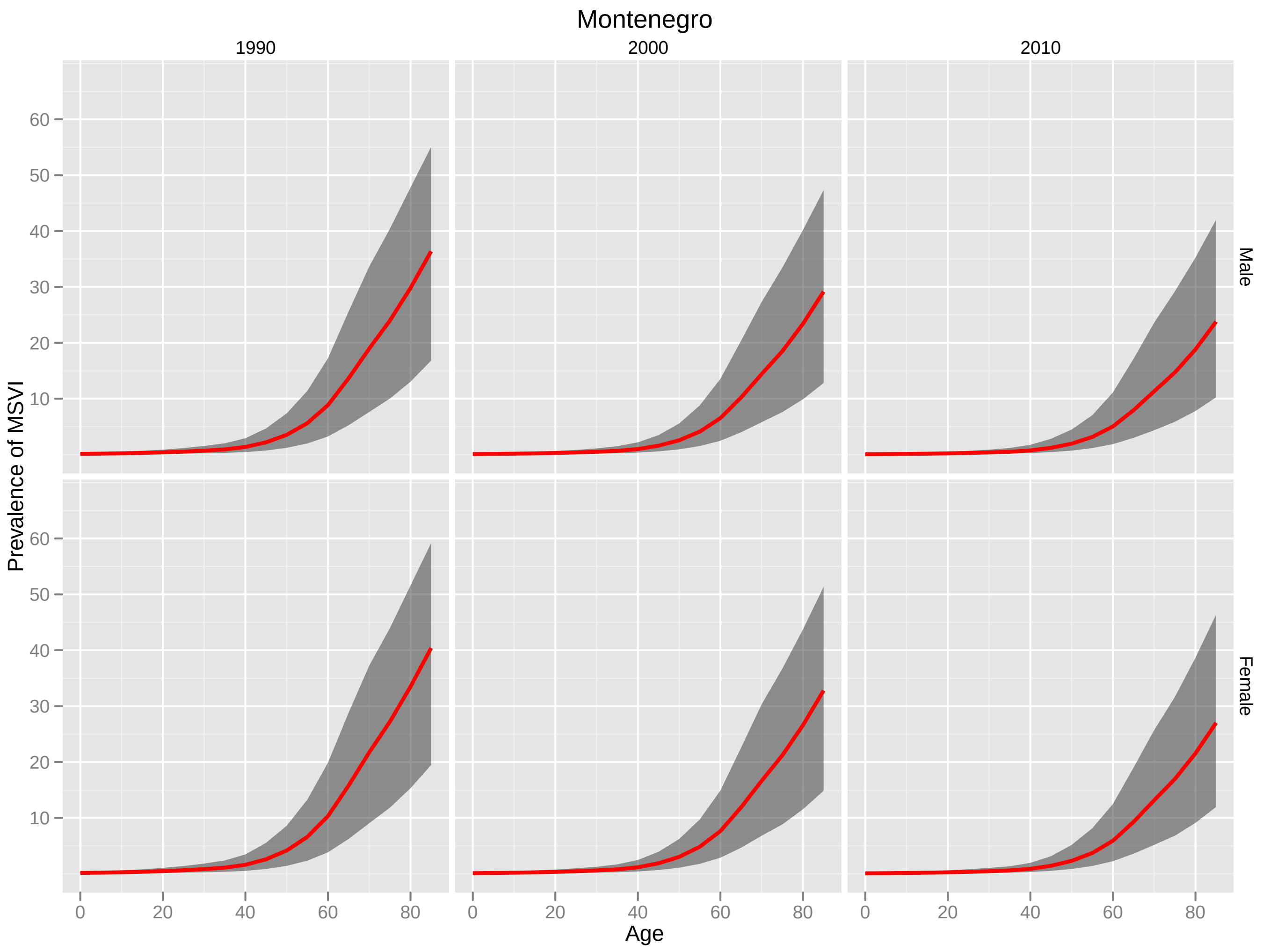
Montenegro
1990
2000
2010
Male
Female
Prevalence of MSVI
Age
10
20
30
40
50
60
0
20
40
60
80

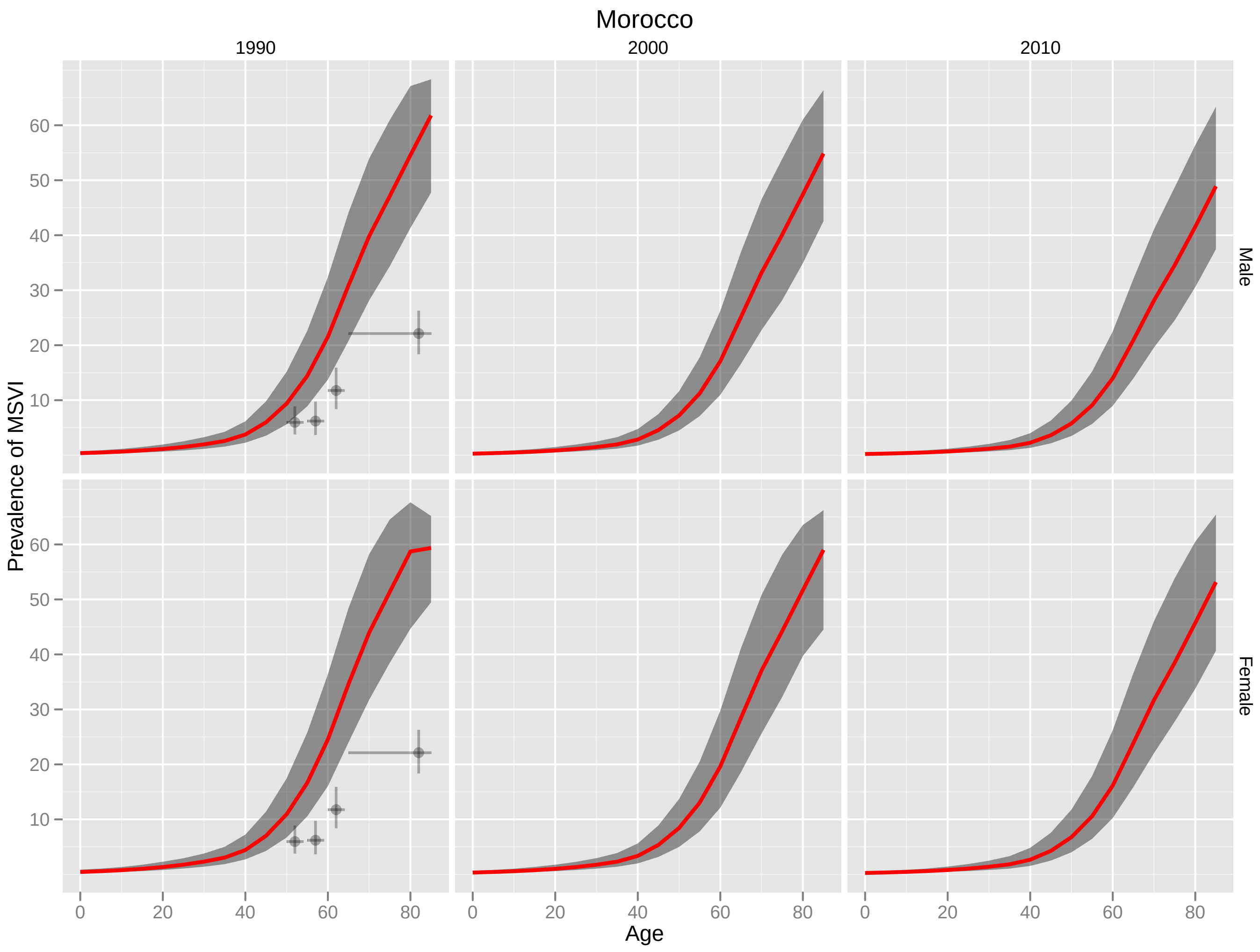

Morocco
1990
2000
2010
Male
Female
Prevalence of MSVI
Age
10
20
30
40
50
60
0
20
40
60
80

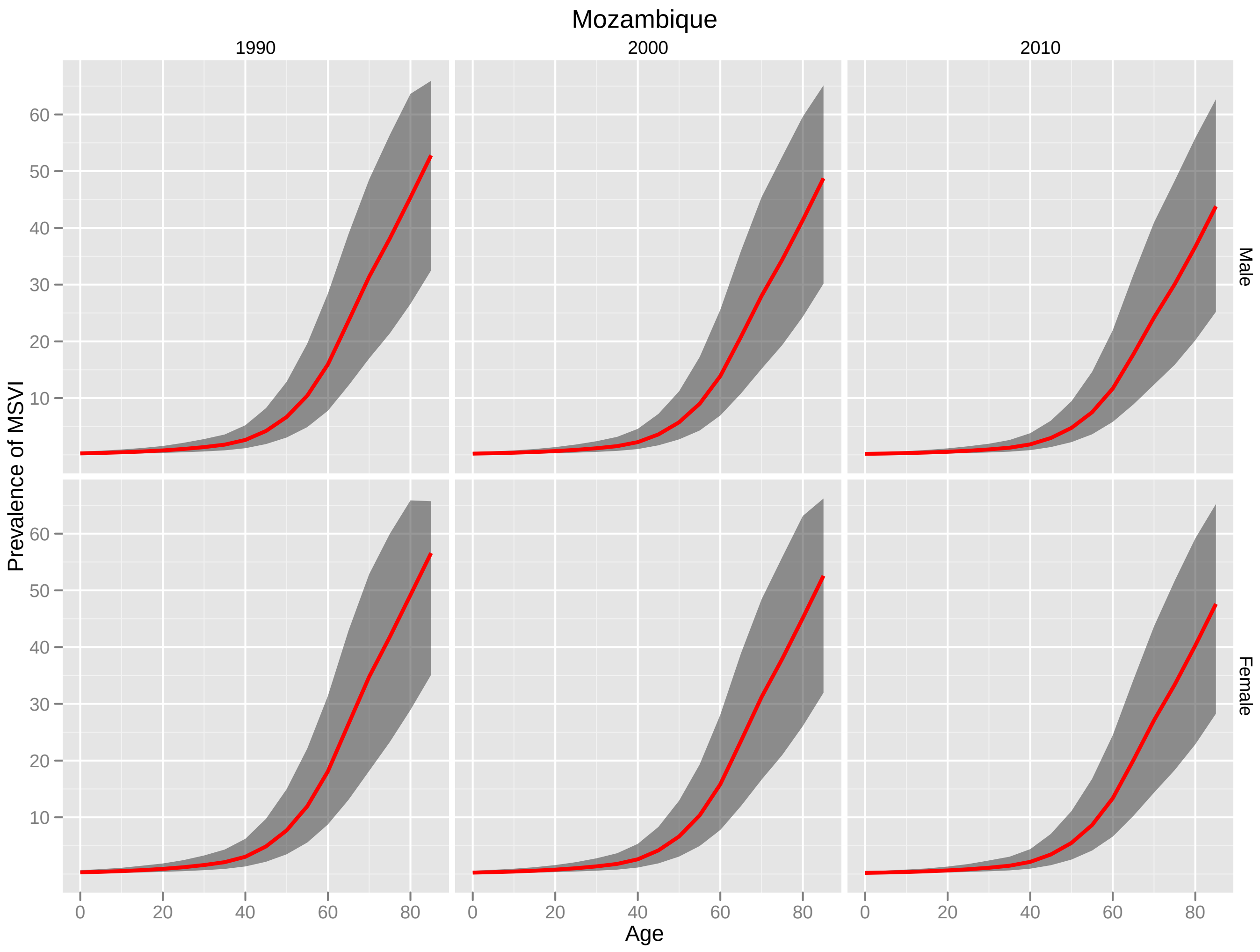
Mozambique
1990
2000
2010
Male
Female
Prevalence of MSVI
Age
10
20
30
40
50
60
0
20
40
60
80

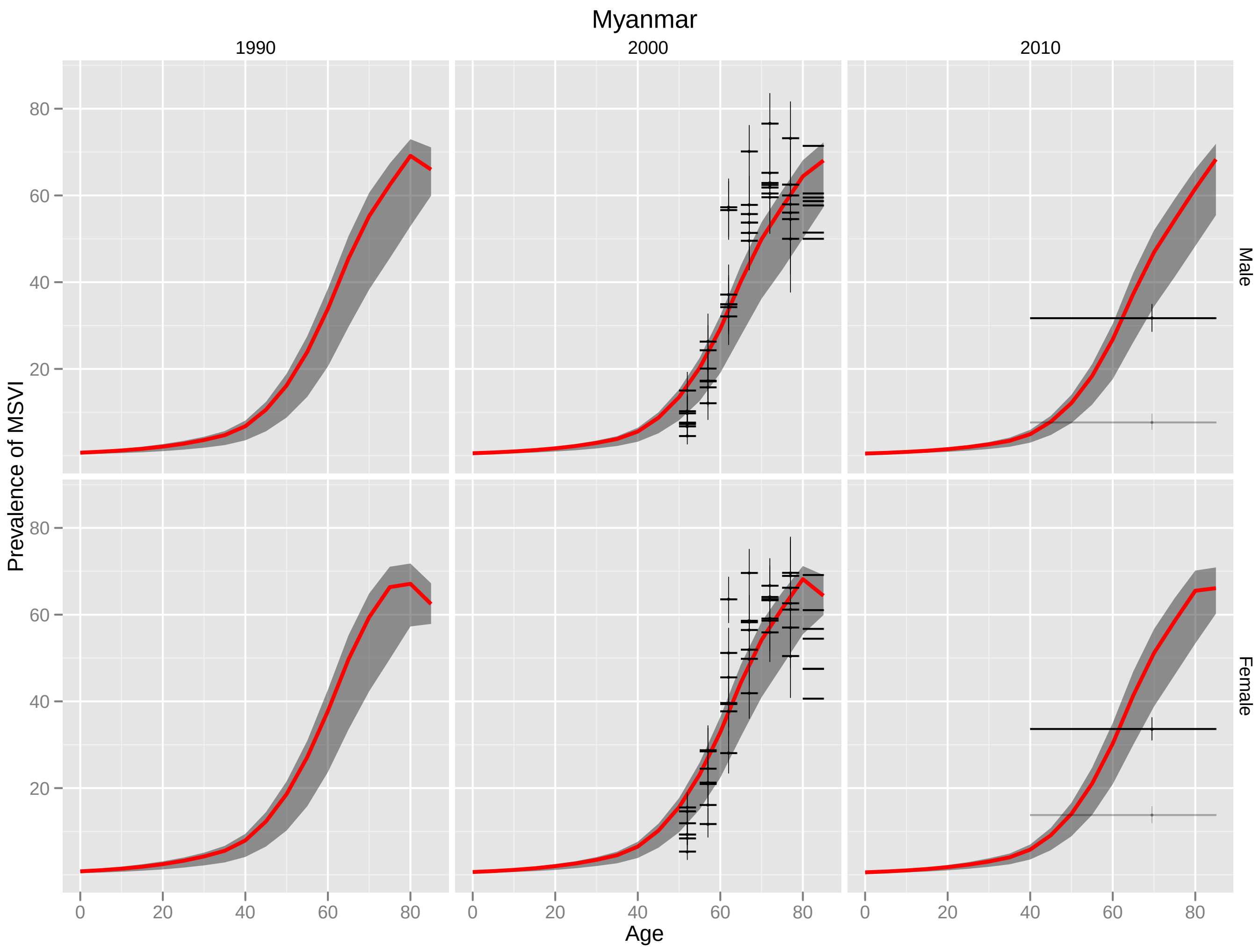
Myanmar
1990
2000
2010
Male
Female
Prevalence of MSVI
Age
0
20
40
60
80

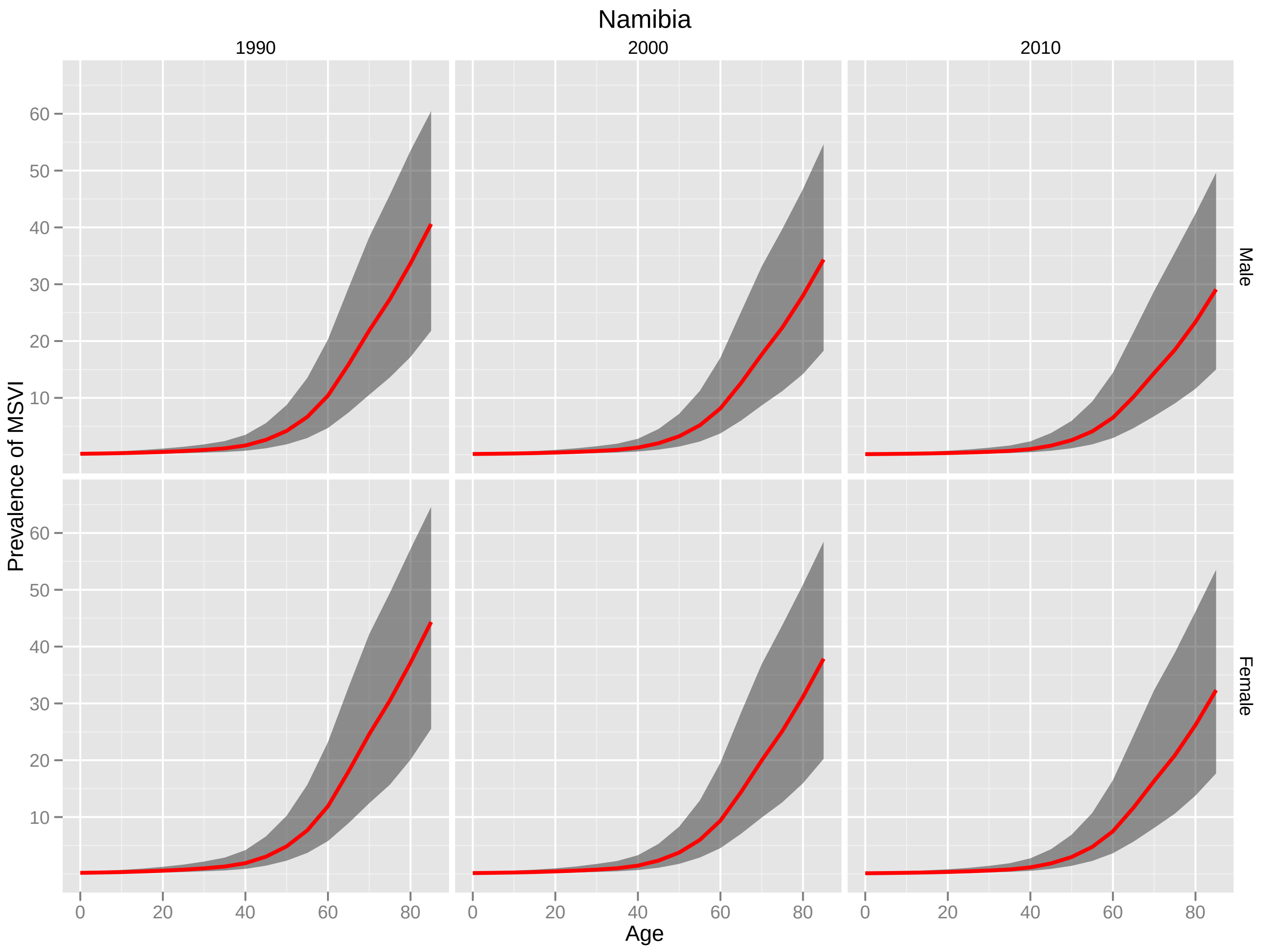
Namibia
1990
2000
2010
Male
Female
Prevalence of MSVI
10
20
30
40
50
60
0
20
40
60
80
Age

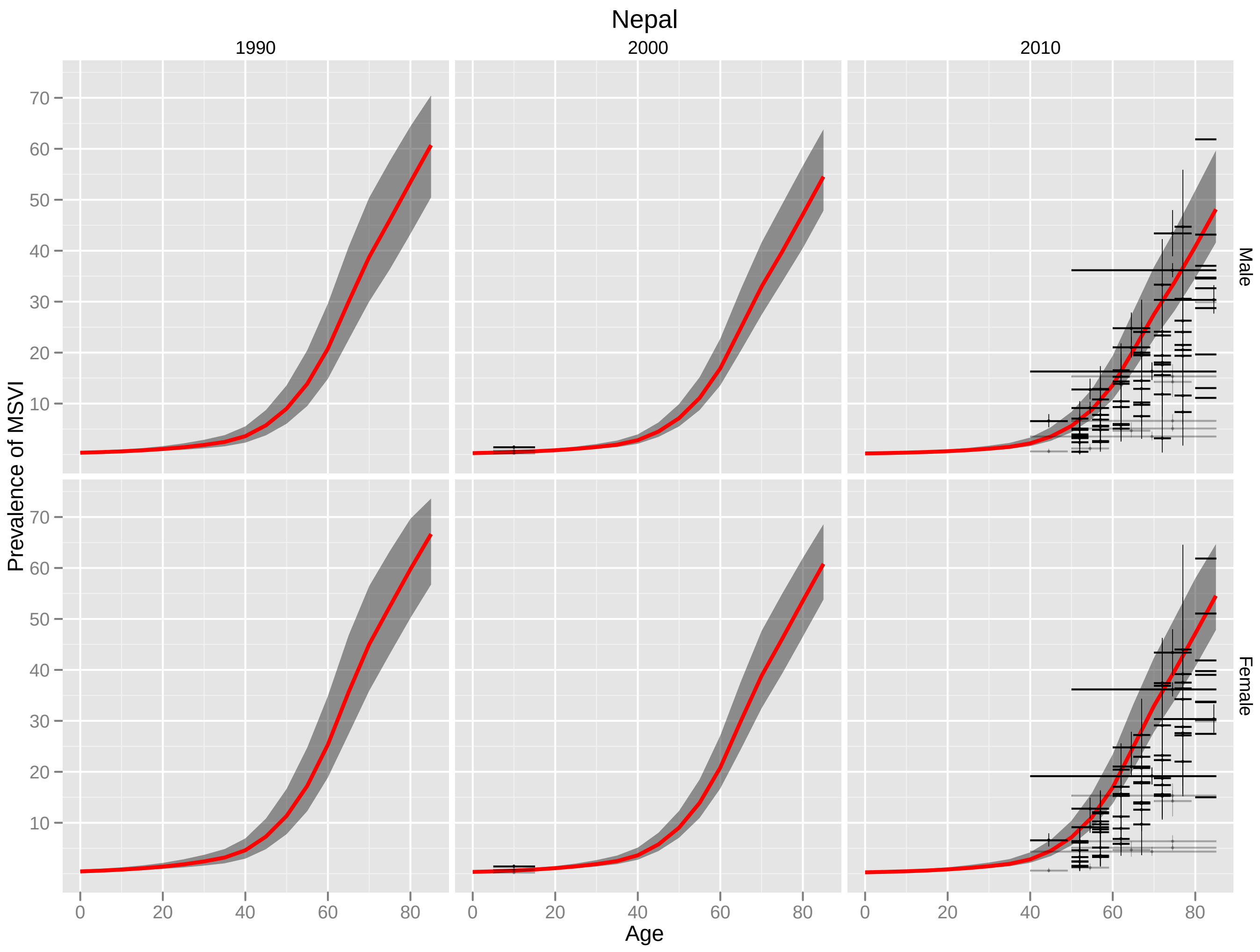
Nepal
1990
2000
2010
Male
Female
Prevalence of MSVI
Age
10
20
30
40
50
60
70
0
20
40
60
80

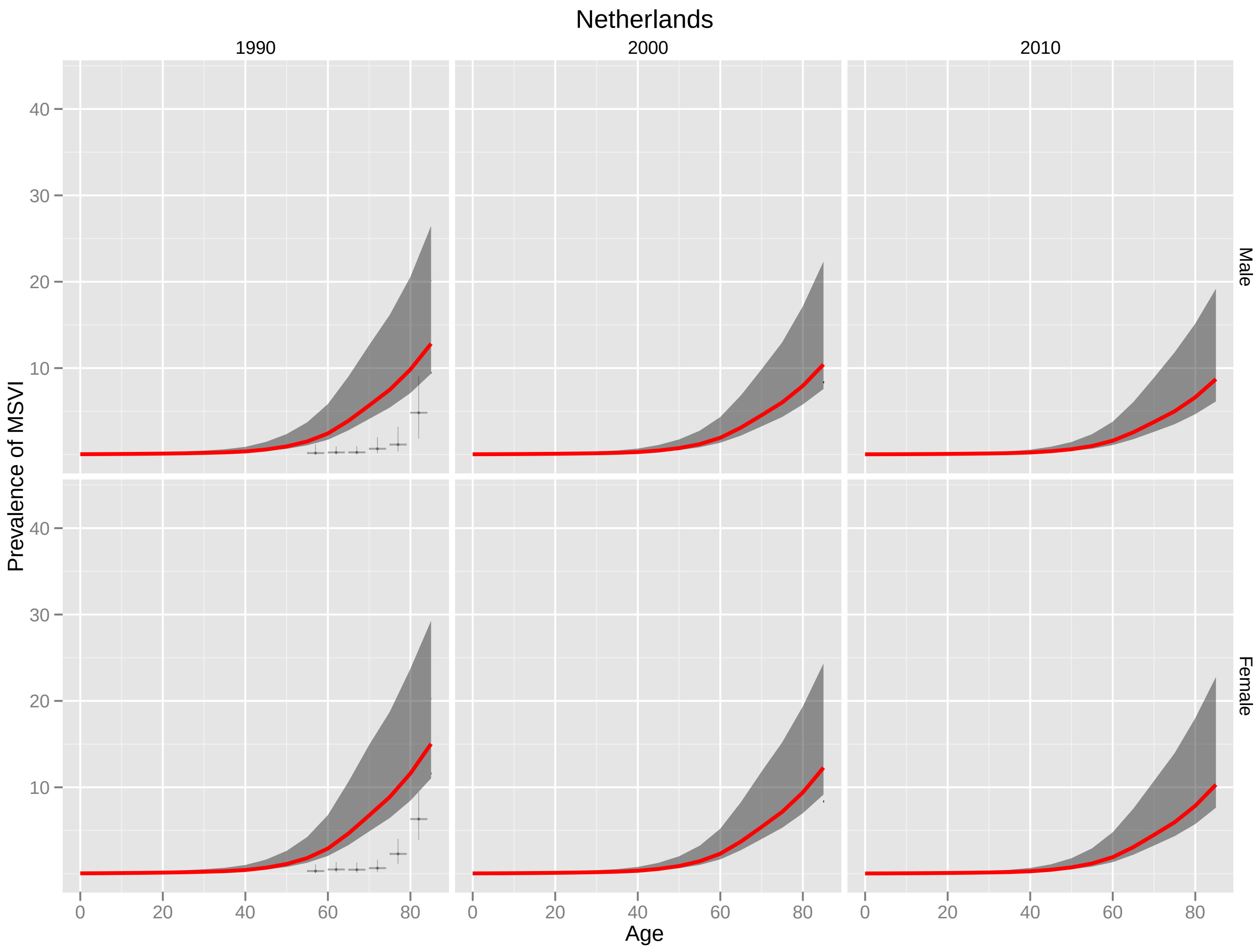
Netherlands
1990
2000
2010
Male
Female
Prevalence of MSVI
Age
0
20
40
60
80
10
20
30
40

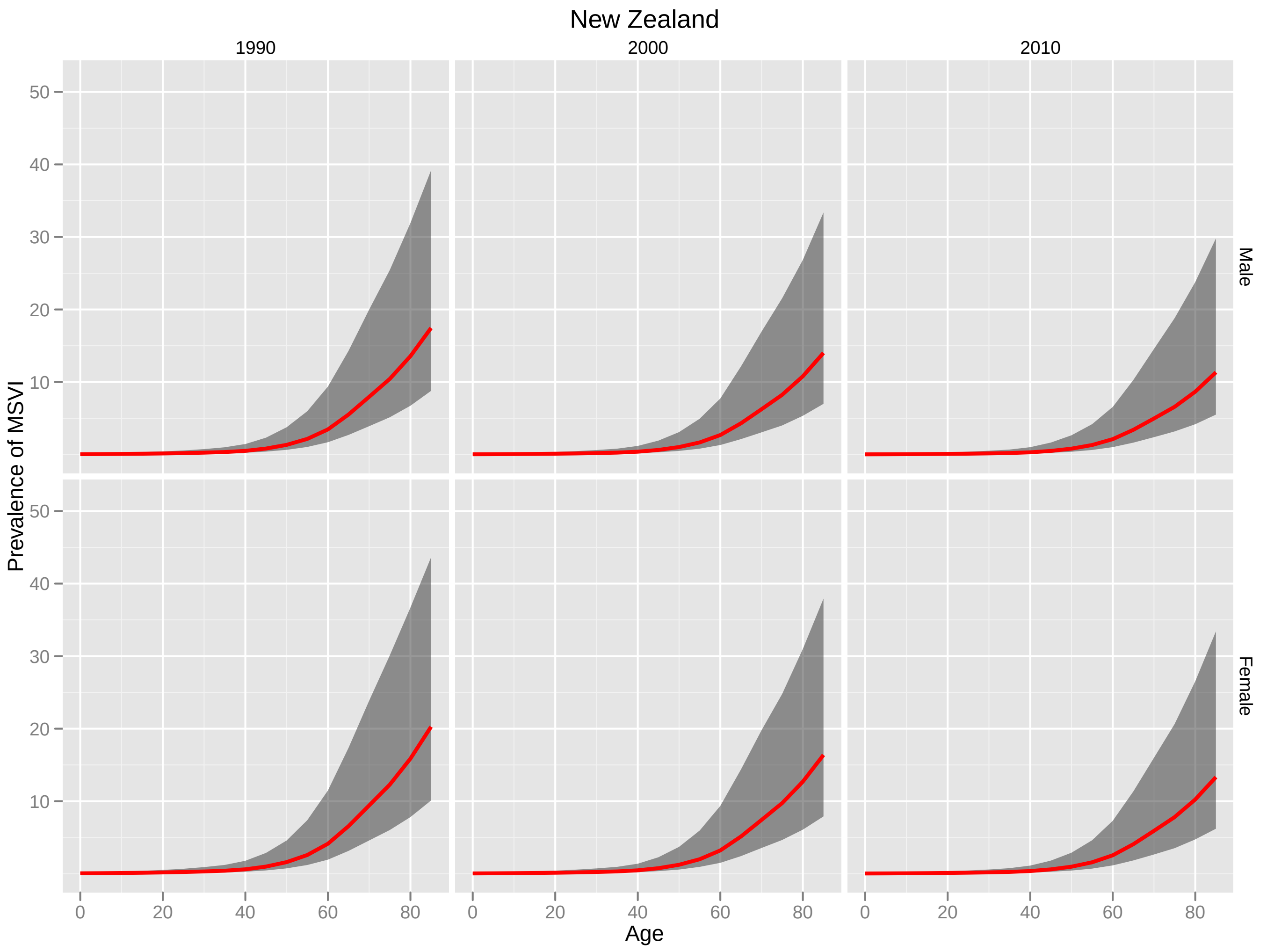
New Zealand
1990
2000
2010
Male
Female
Prevalence of MSVI
Age
0
20
40
60
80
10
20
30
40
50

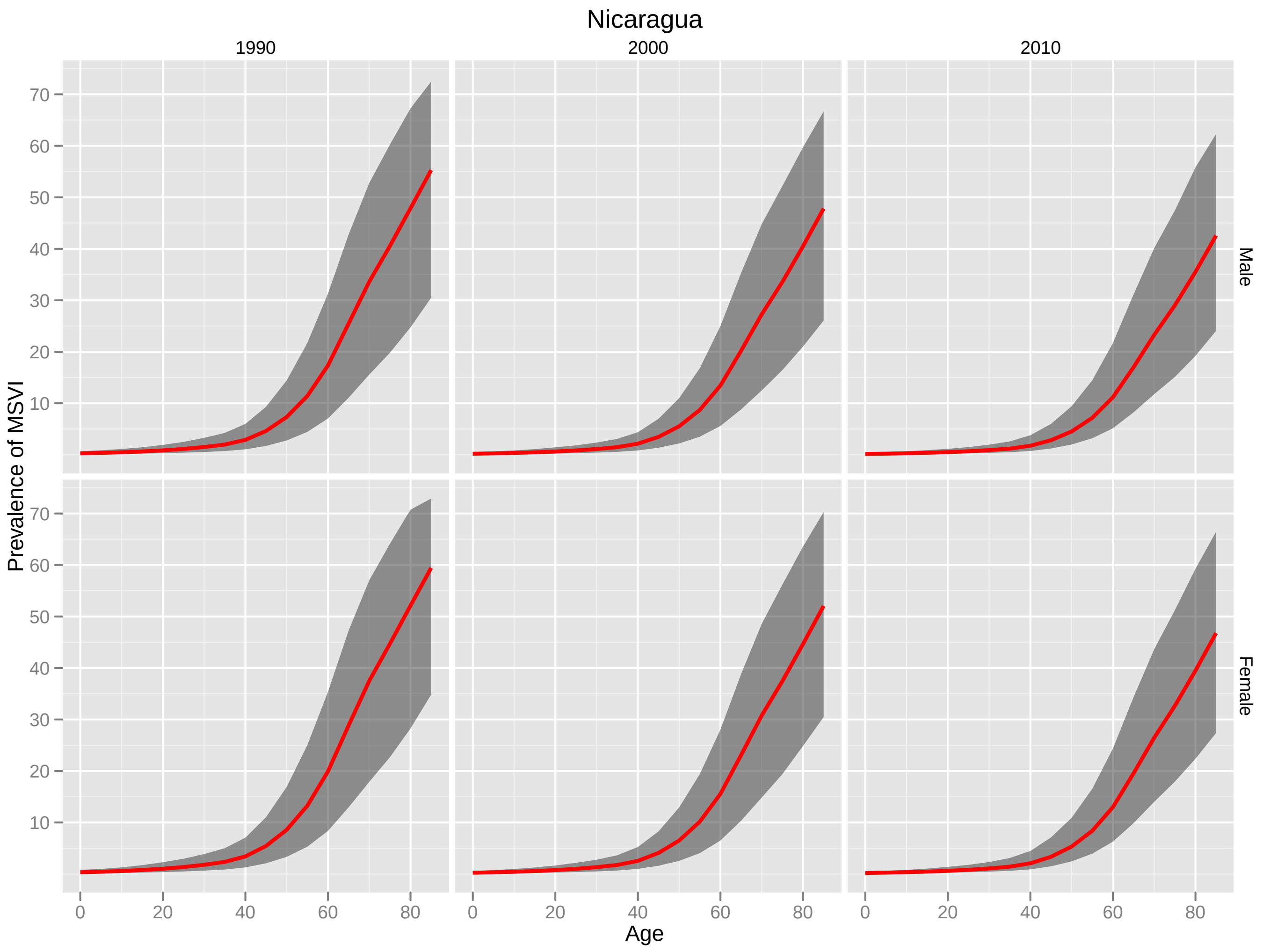
Nicaragua
1990
2000
2010
Male
Female
Prevalence of MSVI
Age
0
20
40
60
80
10
20
30
40
50
60
70

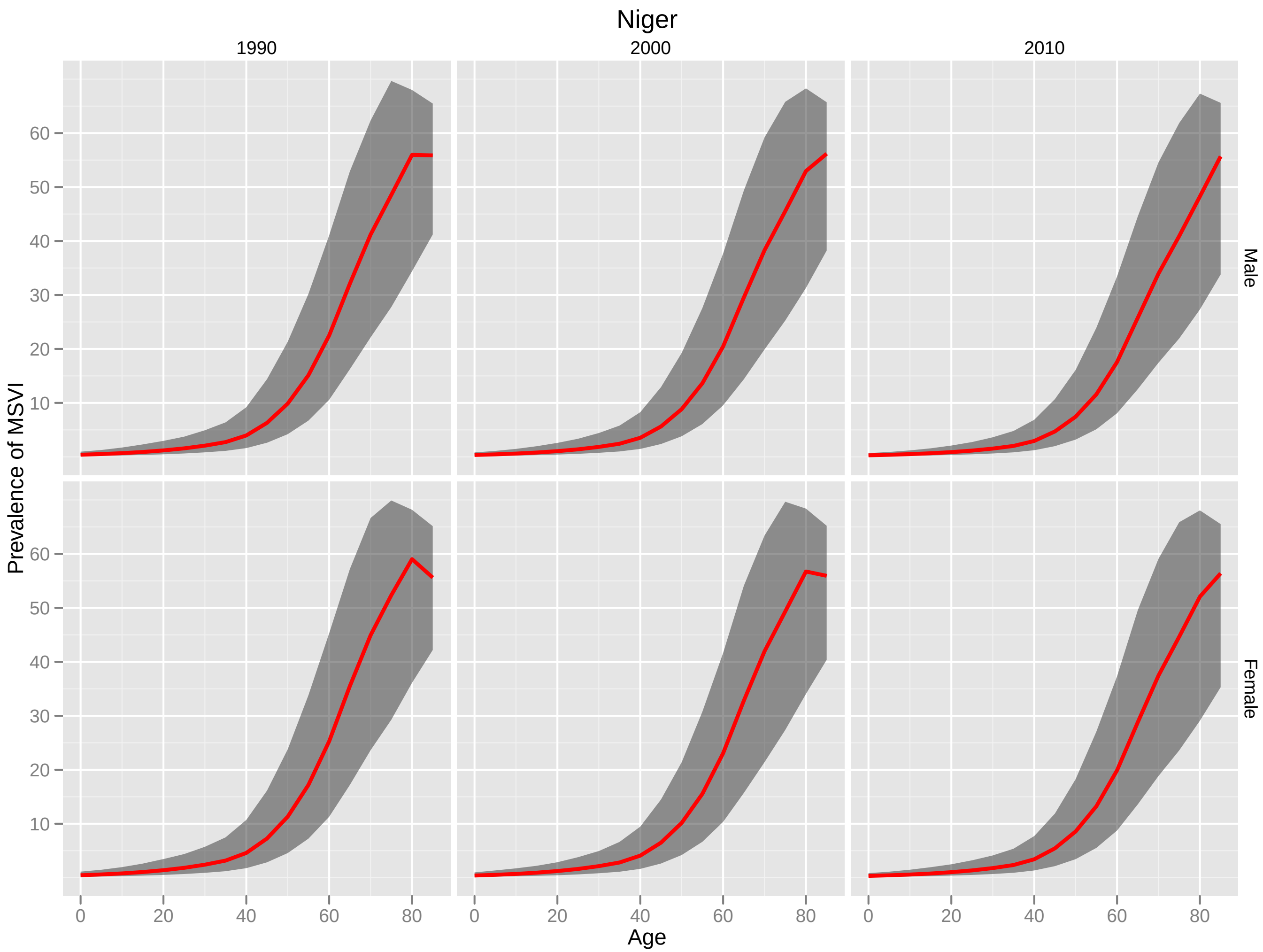

Niger
1990
2000
2010
Male
Female
Prevalence of MSVI
Age
10
20
30
40
50
60
0
20
40
60
80

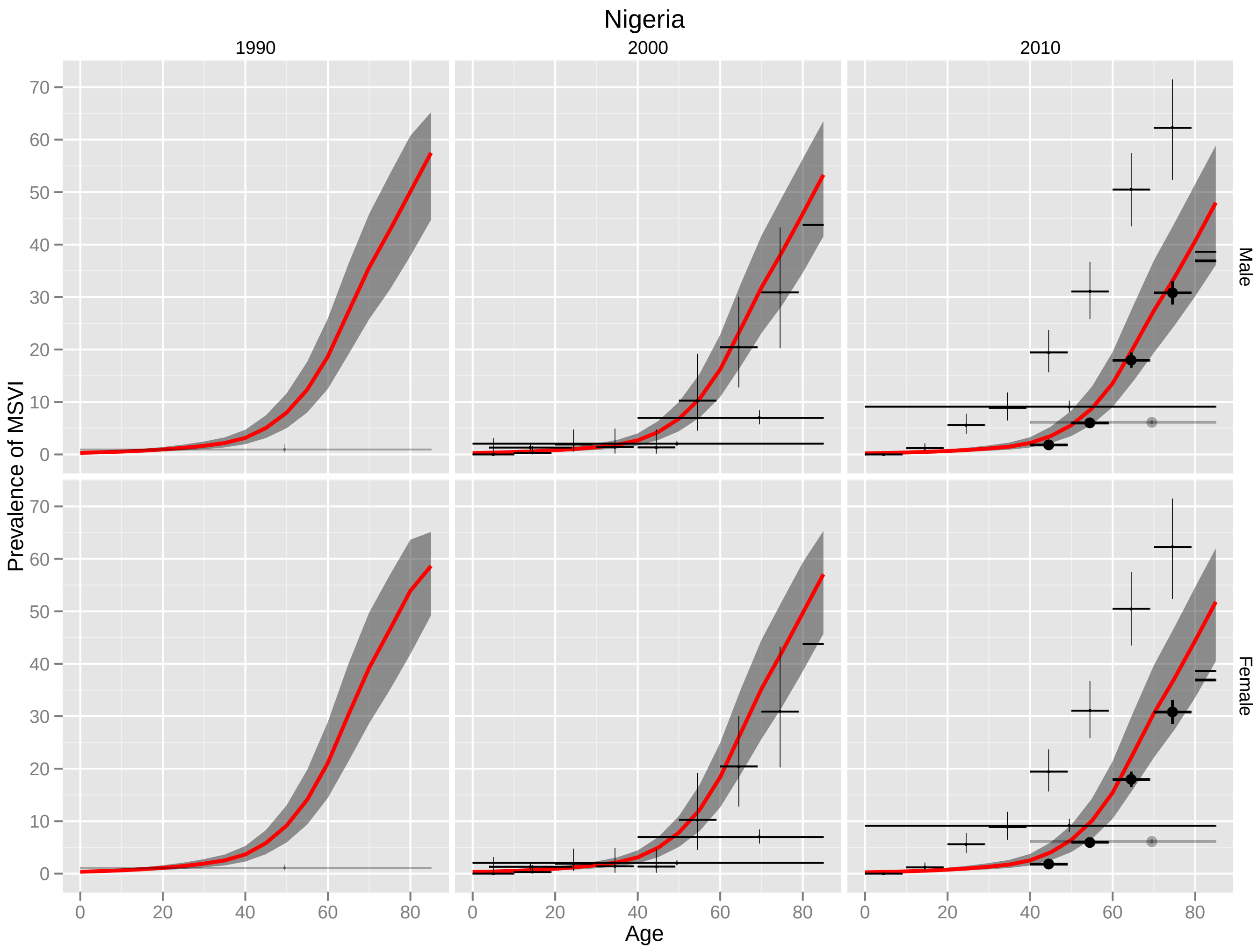
Nigeria
1990
2000
2010
Prevalence of MSVI
Age
Male
Female
0
10
20
30
40
50
60
70
0
20
40
60
80

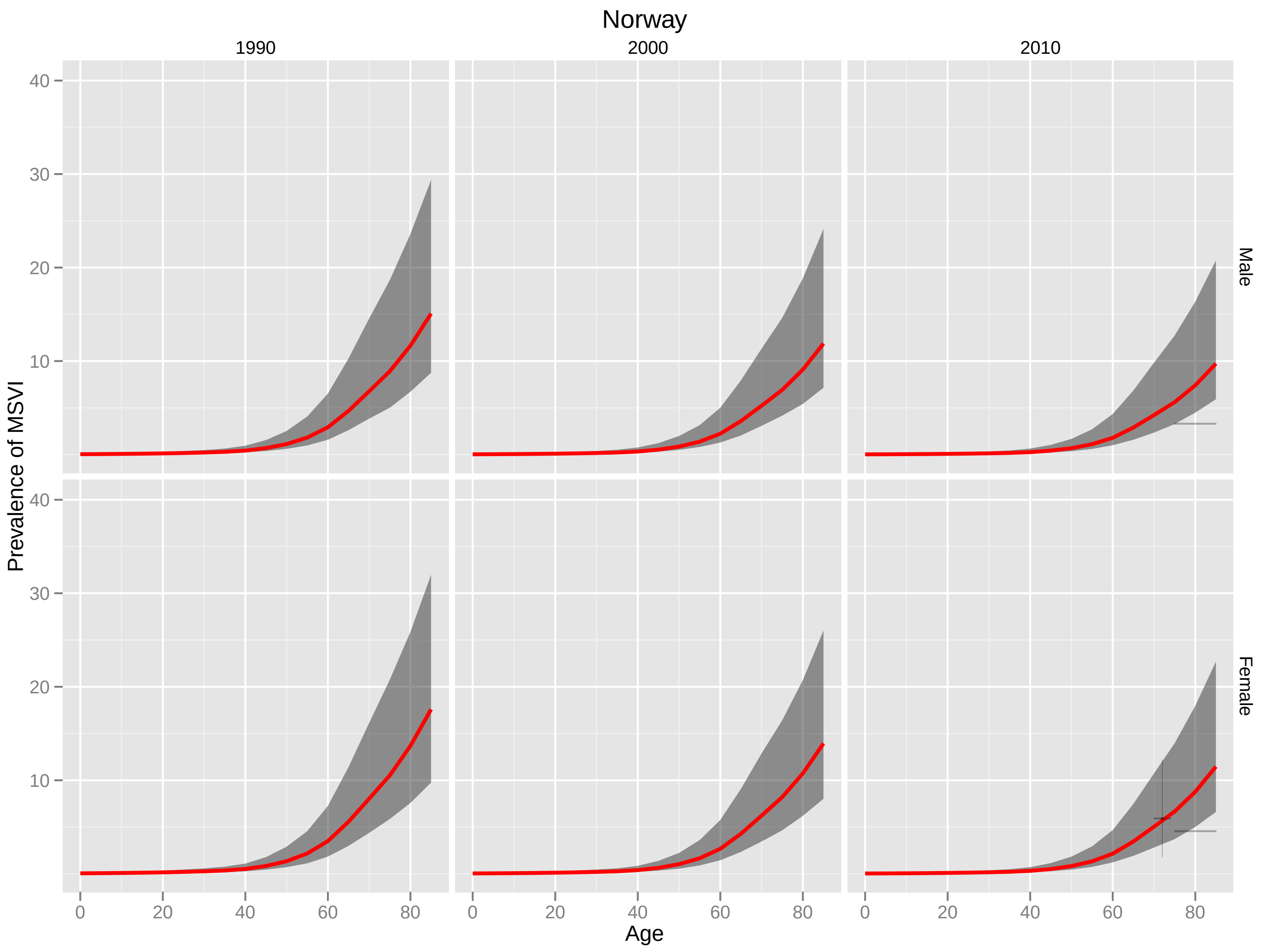
Norway
1990
2000
2010
Male
Female
Prevalence of MSVI
Age
40
30
20
10
0
20
40
60
80

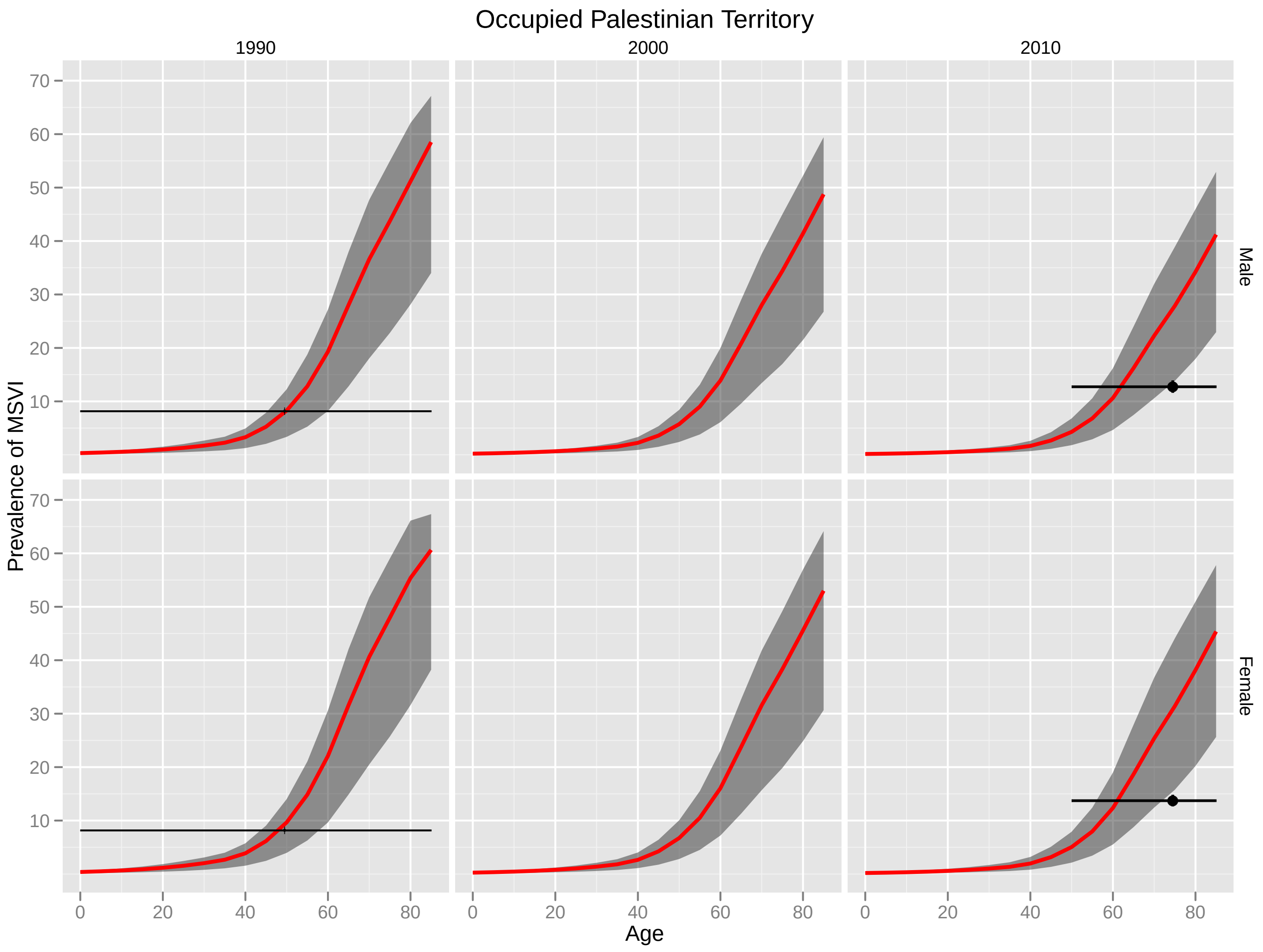
Occupied Palestinian Territory
1990
2000
2010
Male
Female
Prevalence of MSVI
Age
0
20
40
60
80
10
20
30
40
50
60
70

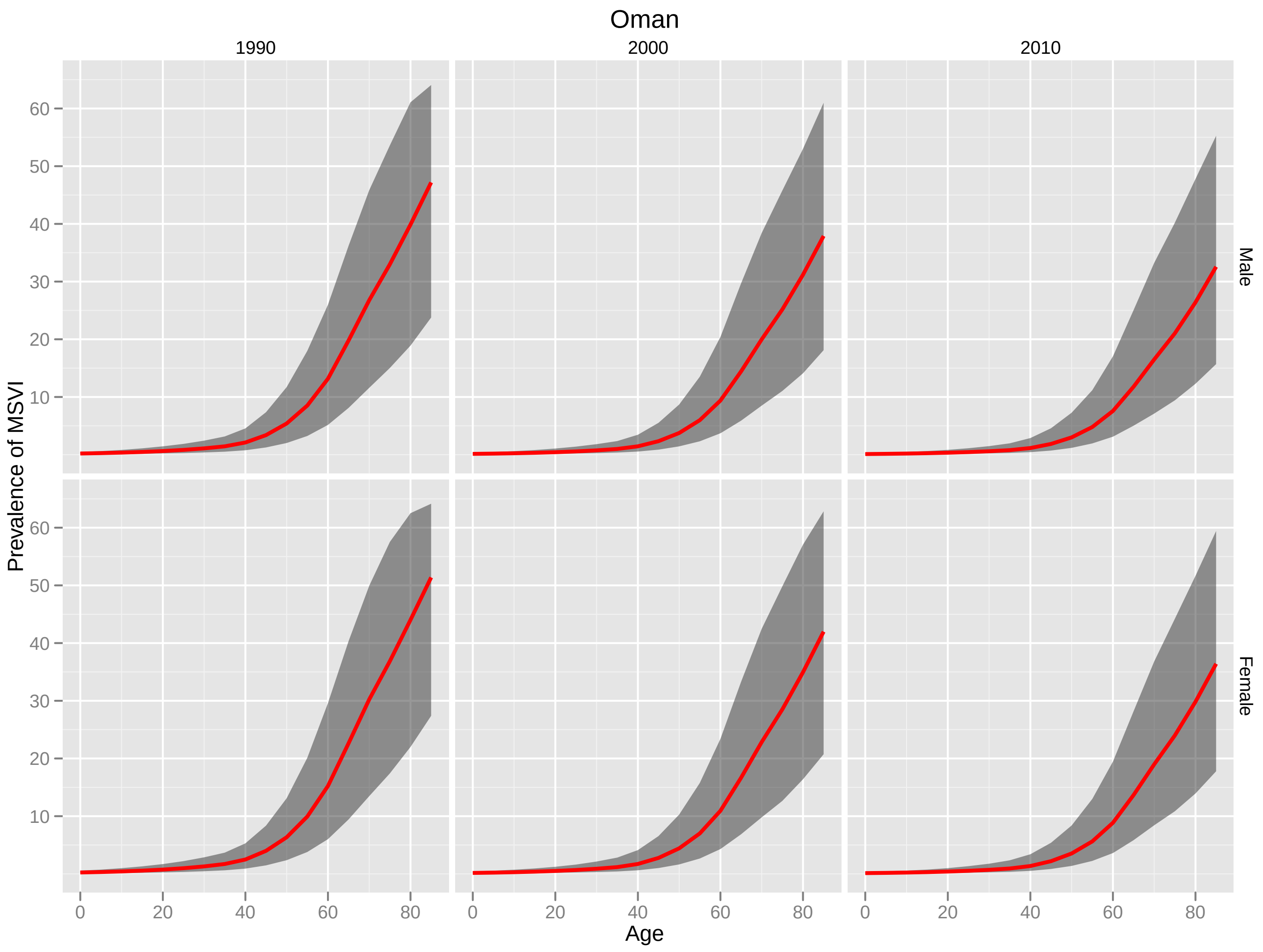
Oman
1990
2000
2010
Male
Female
Prevalence of MSVI
Age
10
20
30
40
50
60
0
20
40
60
80

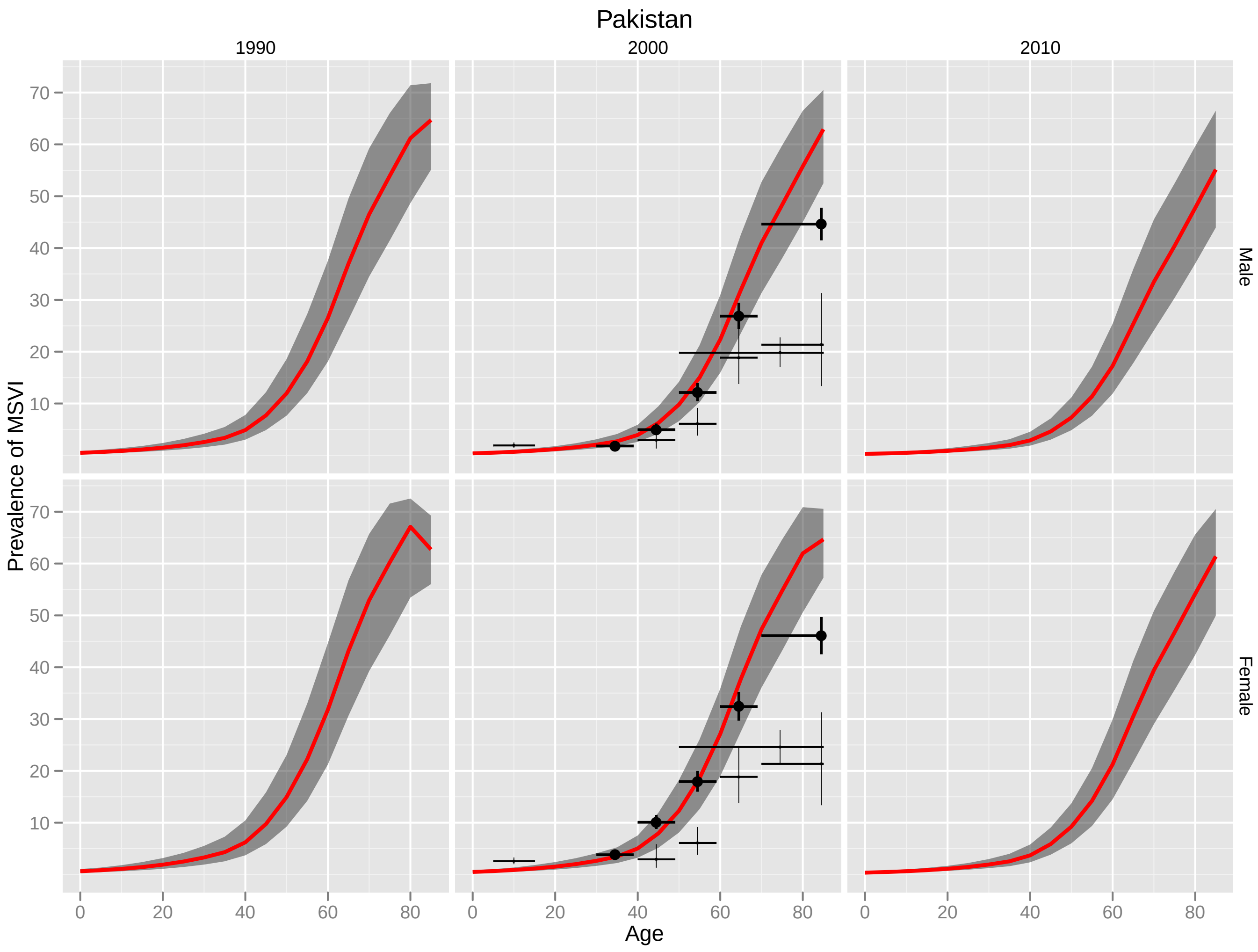
Pakistan
1990
2000
2010
Male
Female
Prevalence of MSVI
Age
10
20
30
40
50
60
70
0
20
40
60
80

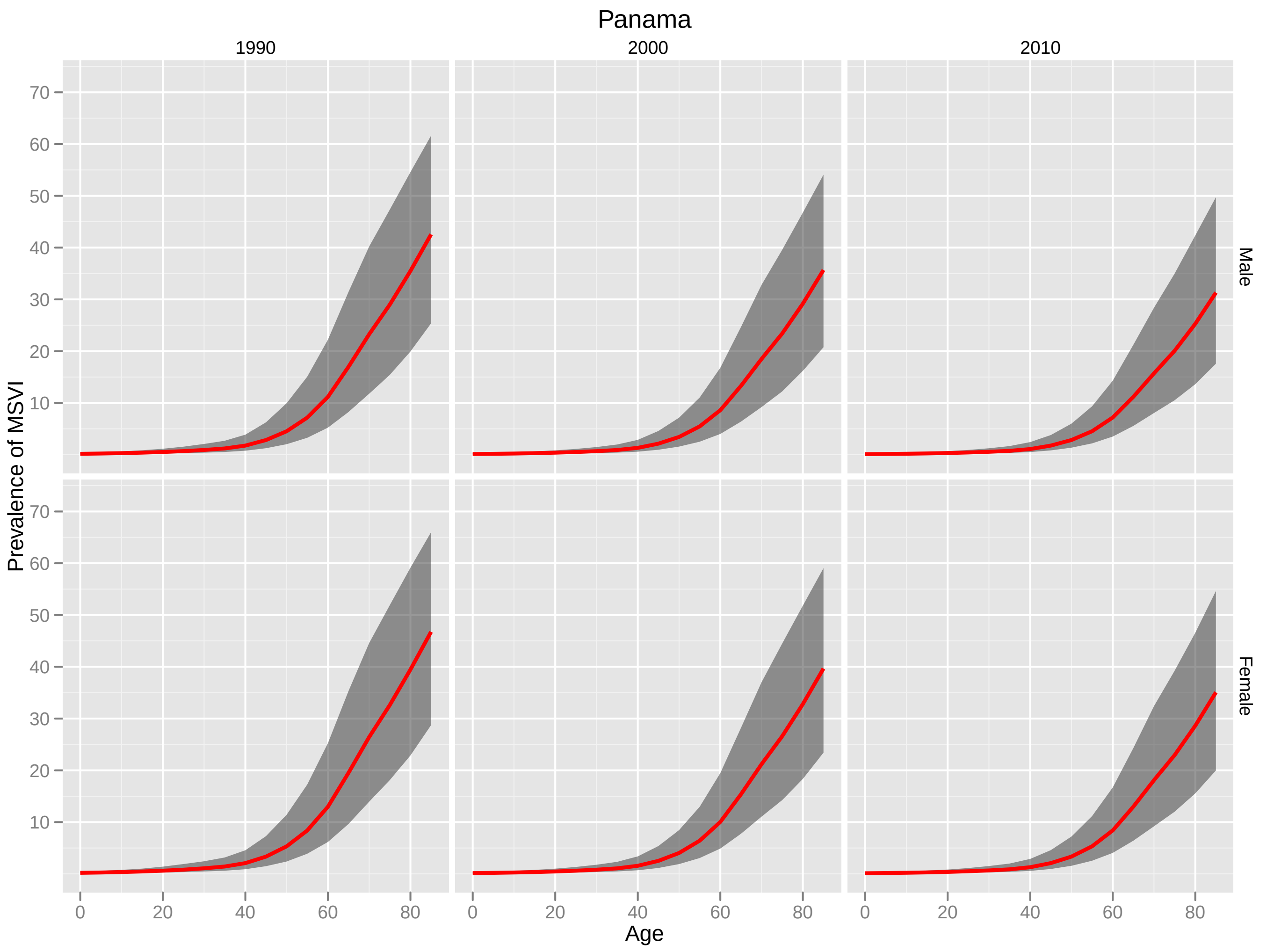
Panama
1990
2000
2010
Male
Female
Prevalence of MSVI
Age
10
20
30
40
50
60
70
0
20
40
60
80

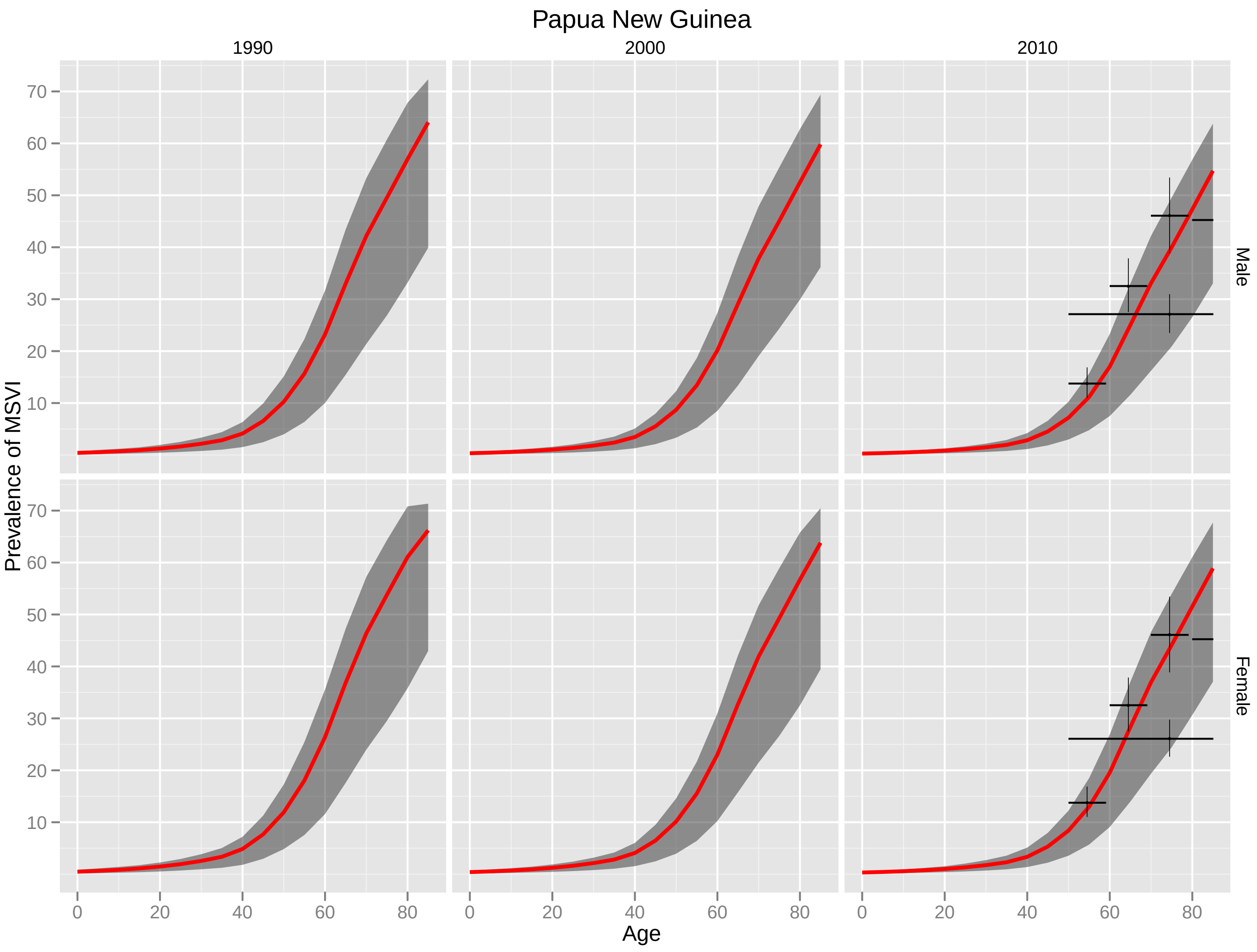
Papua New Guinea
1990
2000
2010
Male
Female
Prevalence of MSVI
Age
0
20
40
60
80
10
20
30
40
50
60
70

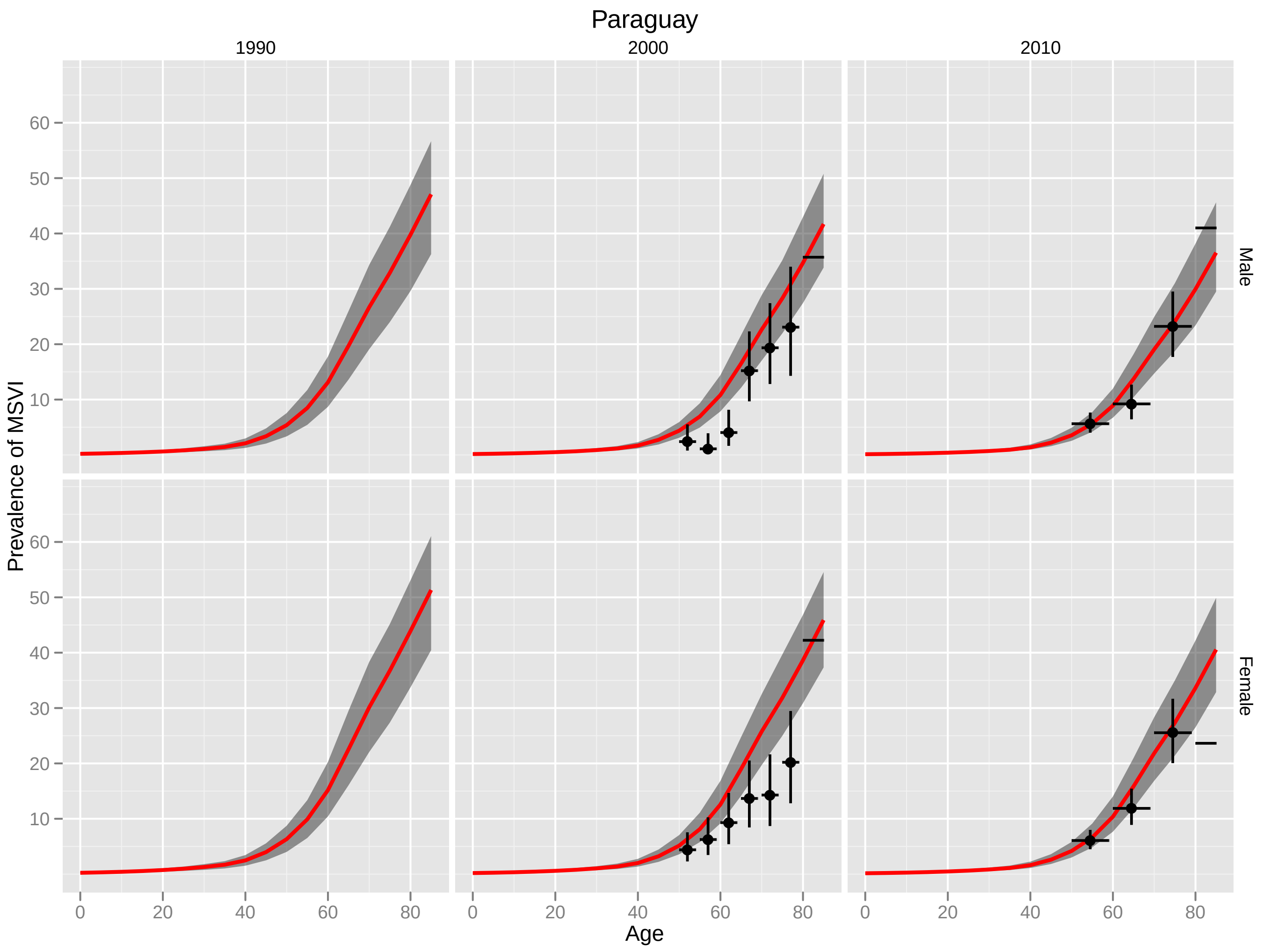

Paraguay
1990
2000
2010
Male
Female
Prevalence of MSVI
Age
10
20
30
40
50
60
0
20
40
60
80

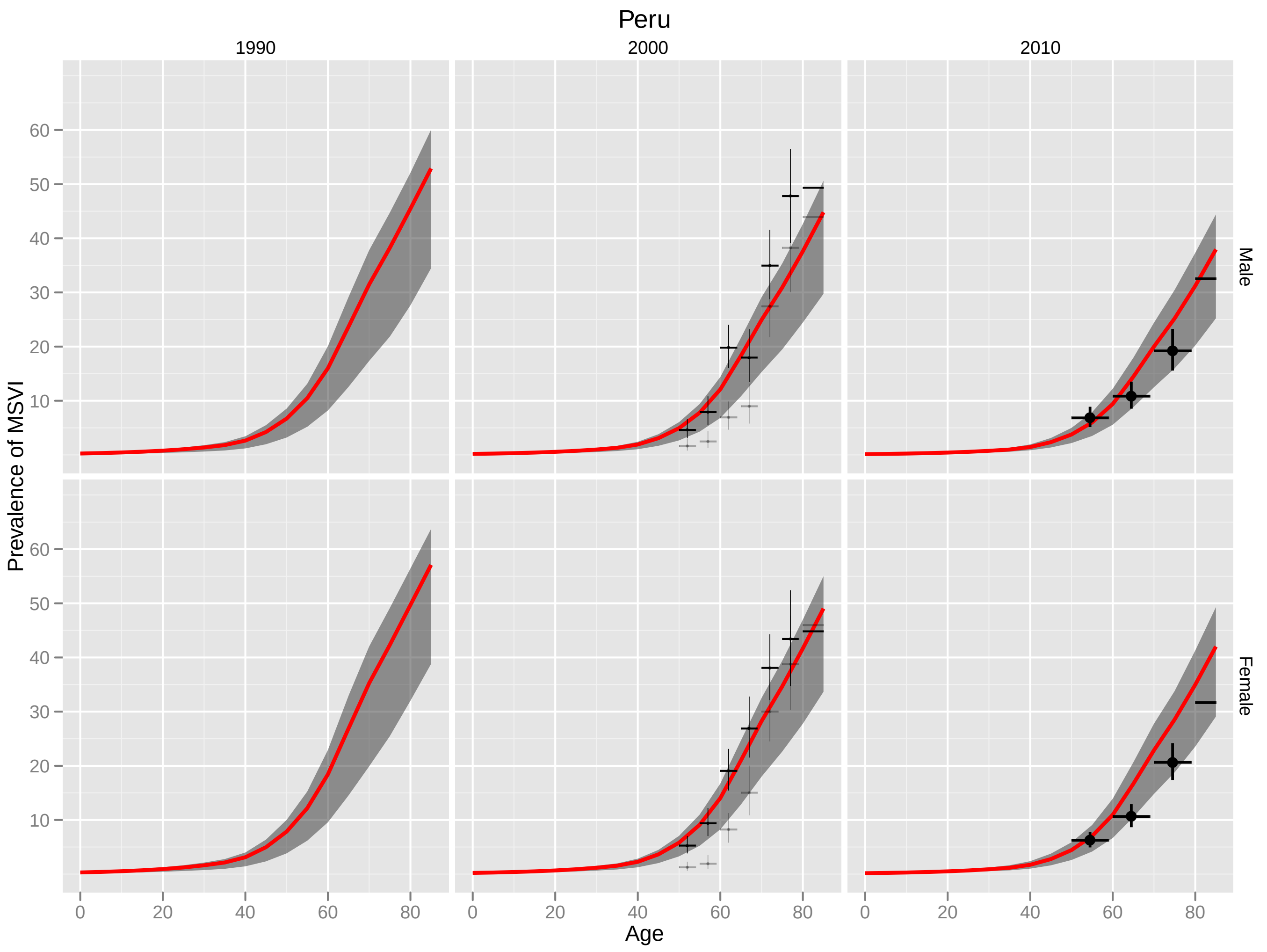
Peru
1990
2000
2010
Male
Female
Prevalence of MSVI
Age
10
20
30
40
50
60
0
20
40
60
80

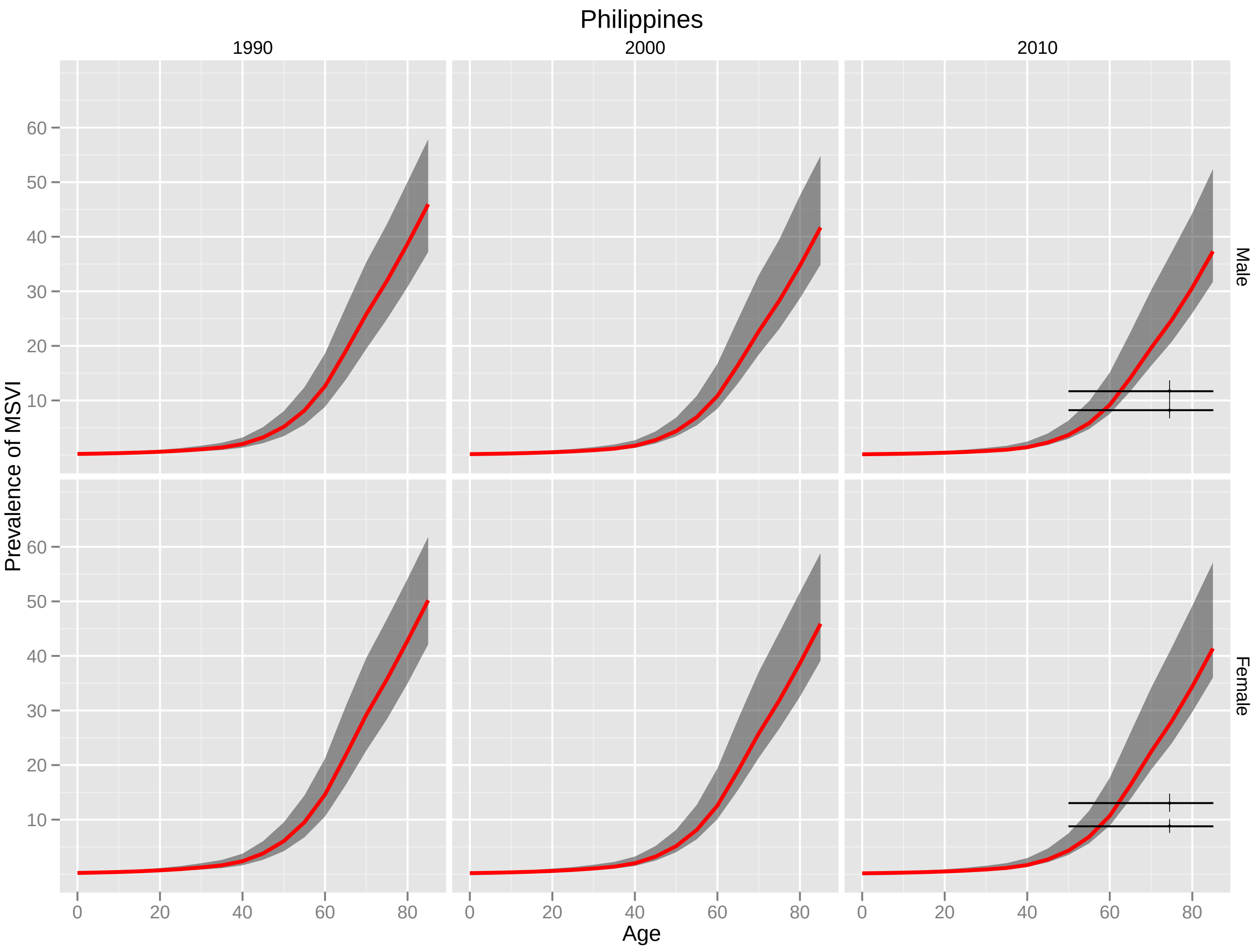
Philippines
1990
2000
2010
Male
Female
Prevalence of MSVI
Age
10
20
30
40
50
60
0
20
40
60
80

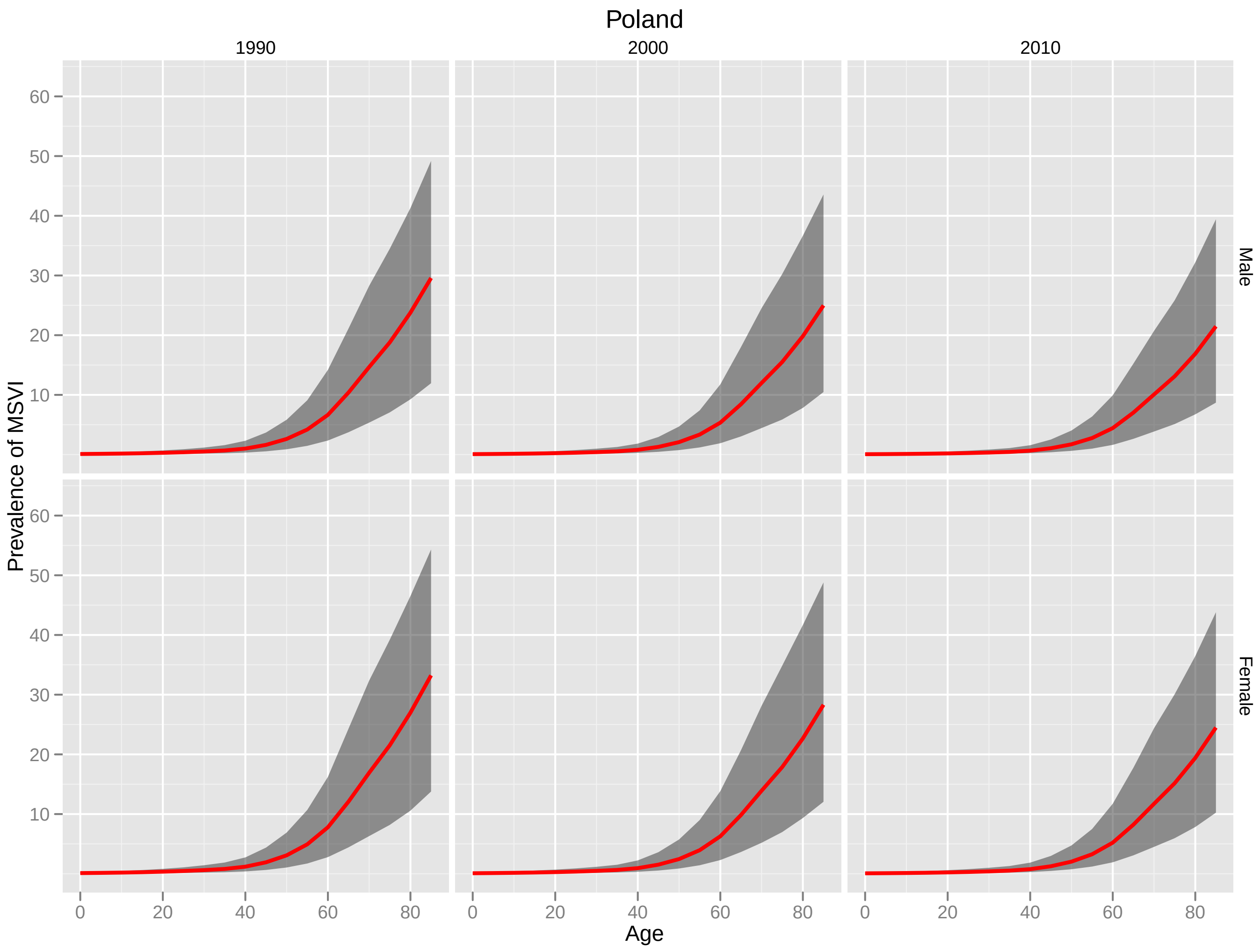
Poland
1990
2000
2010
Male
Female
Prevalence of MSVI
Age
10
20
30
40
50
60
0
20
40
60
80

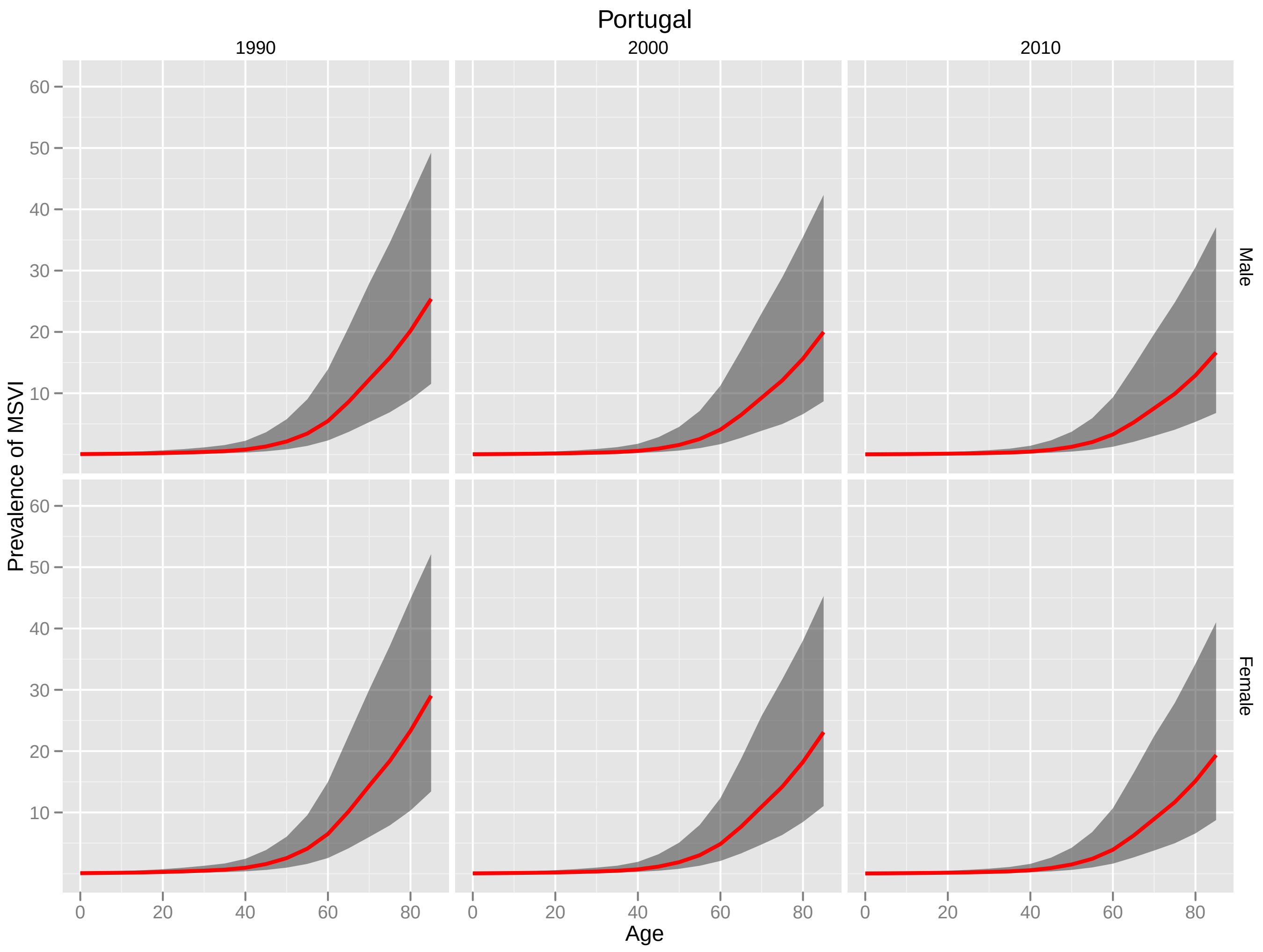

Portugal
1990
2000
2010
Male
Female
Prevalence of MSVI
Age
0
20
40
60
80
10
20
30
40
50
60

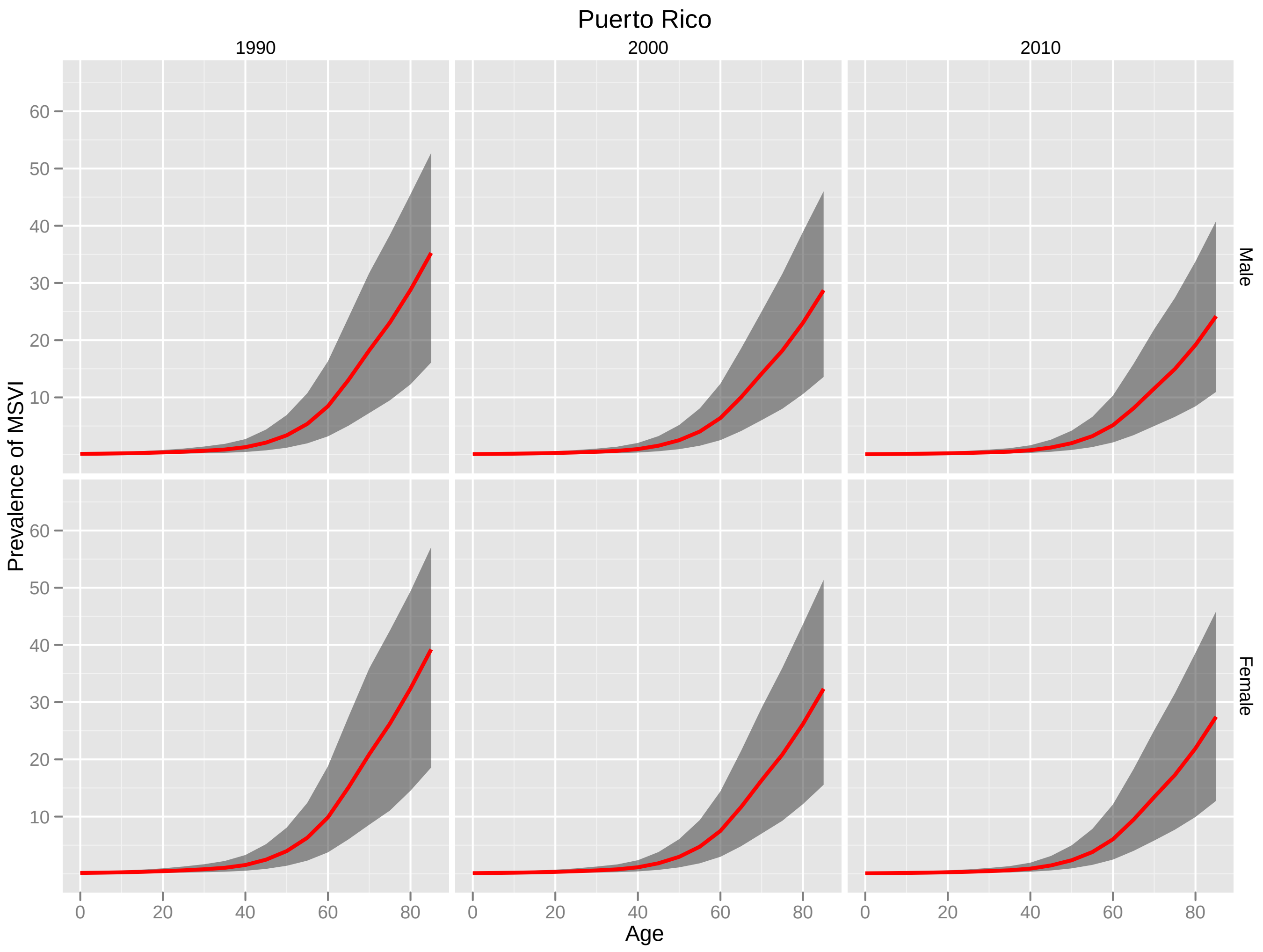
Puerto Rico
1990
2000
2010
Male
Female
Prevalence of MSVI
Age
0
20
40
60
80
10
20
30
40
50
60

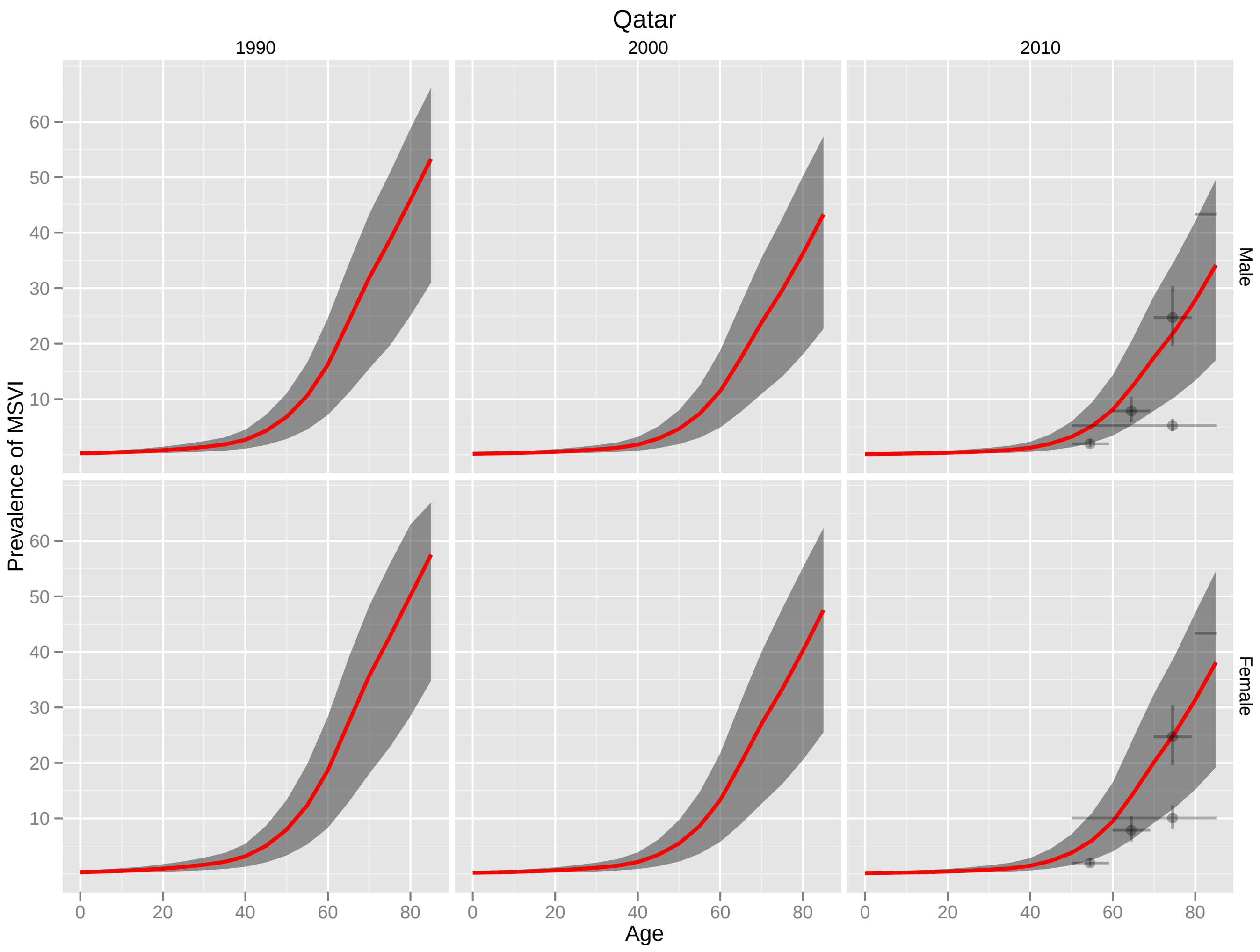

Qatar
1990
2000
2010
Male
Female
Prevalence of MSVI
Age
10
20
30
40
50
60
0
20
40
60
80

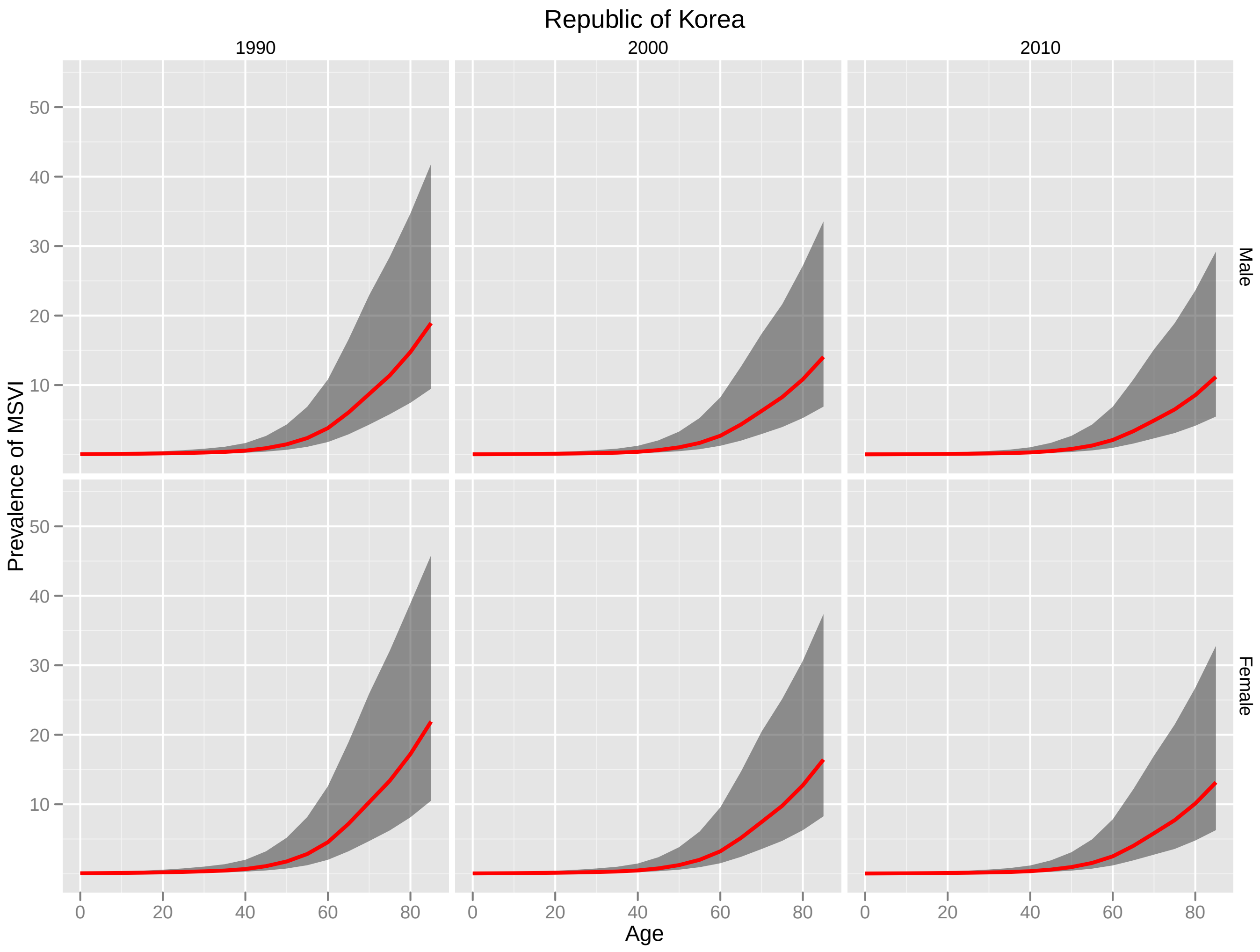
Republic of Korea
1990
2000
2010
Male
Female
Prevalence of MSVI
Age
0
20
40
60
80
10
20
30
40
50

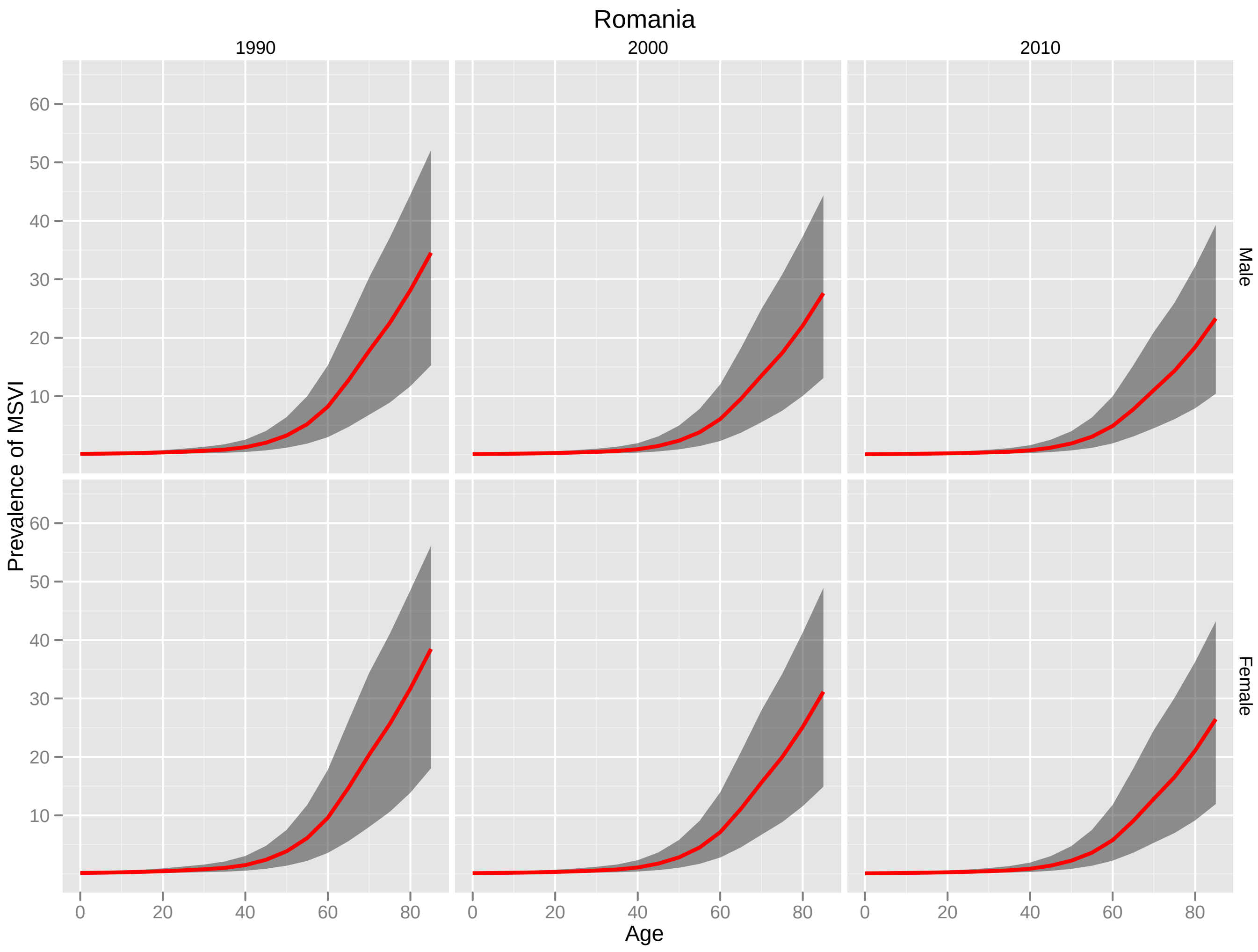
Romania
1990
2000
2010
Male
Female
Prevalence of MSVI
Age
0
20
40
60
80
10
20
30
40
50
60

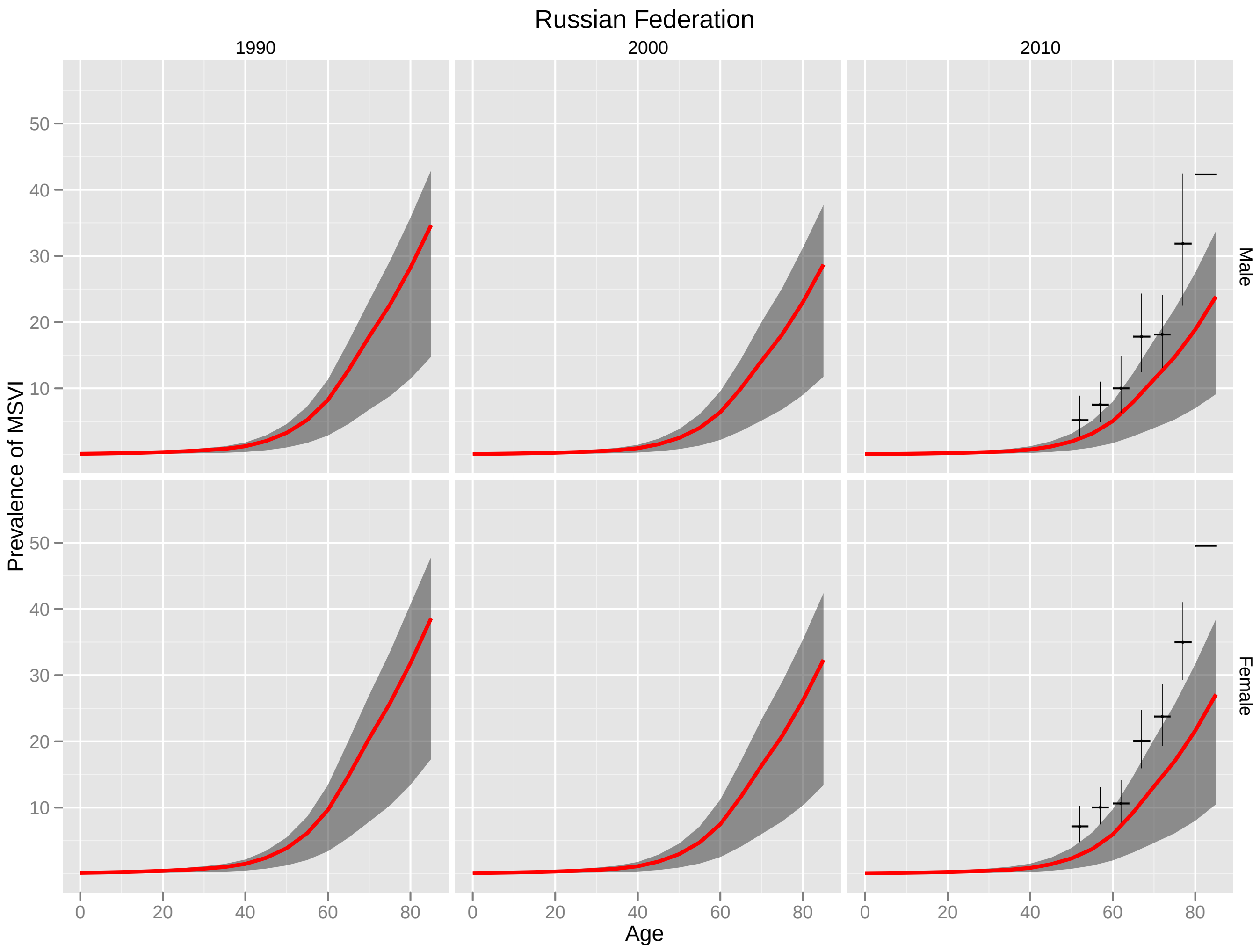
Russian Federation
1990
2000
2010
Male
Female
Prevalence of MSVI
Age
0
20
40
60
80
10
20
30
40
50

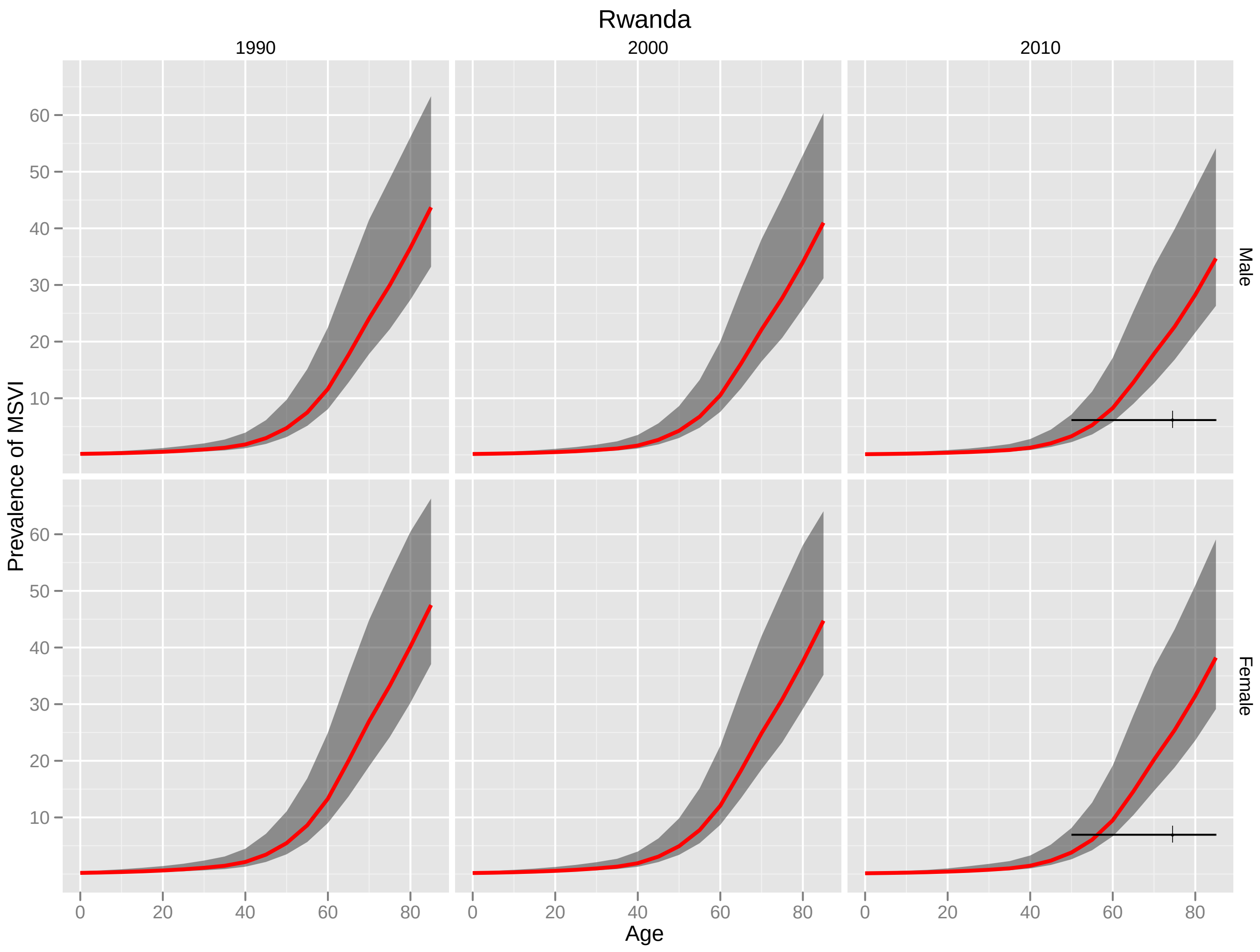
Rwanda
1990
2000
2010
Male
Female
Prevalence of MSVI
Age
0
20
40
60
80
10
20
30
40
50
60

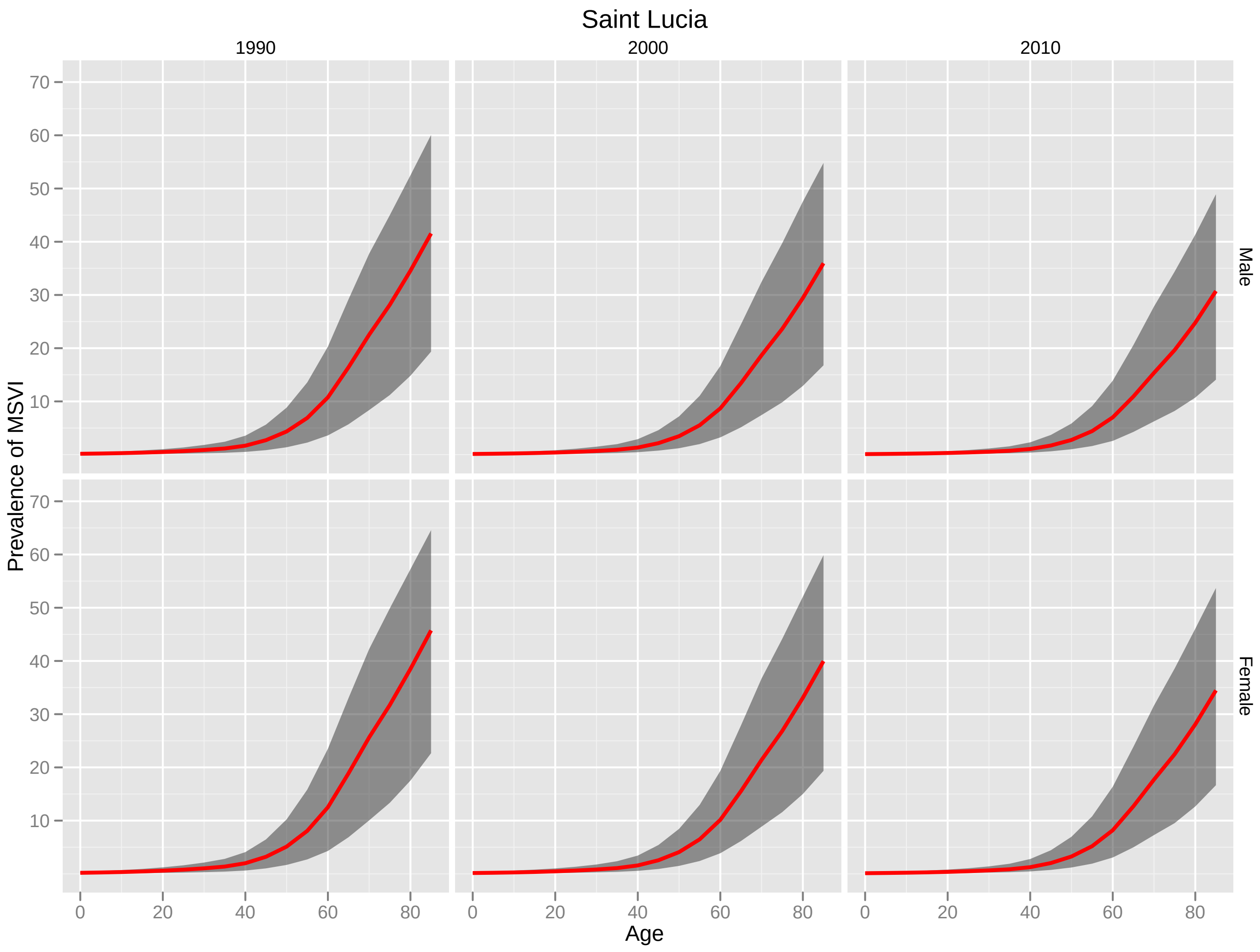

Saint Lucia
1990
2000
2010
Male
Female
Prevalence of MSVI
Age
0
20
40
60
80
10
20
30
40
50
60
70

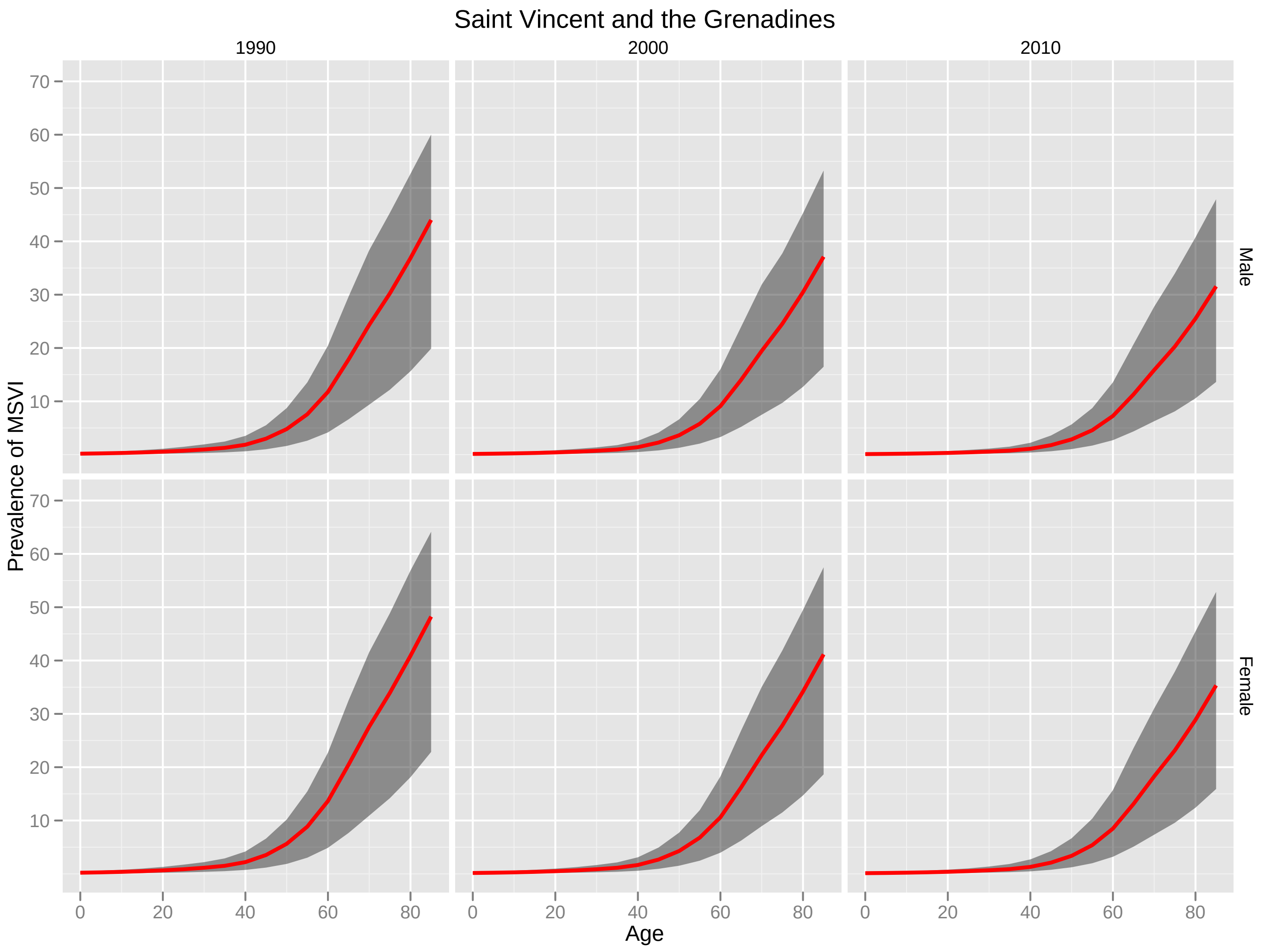

Saint Vincent and the Grenadines
1990
2000
2010
Male
Female
Prevalence of MSVI
Age
0
20
40
60
80
10
20
30
40
50
60
70

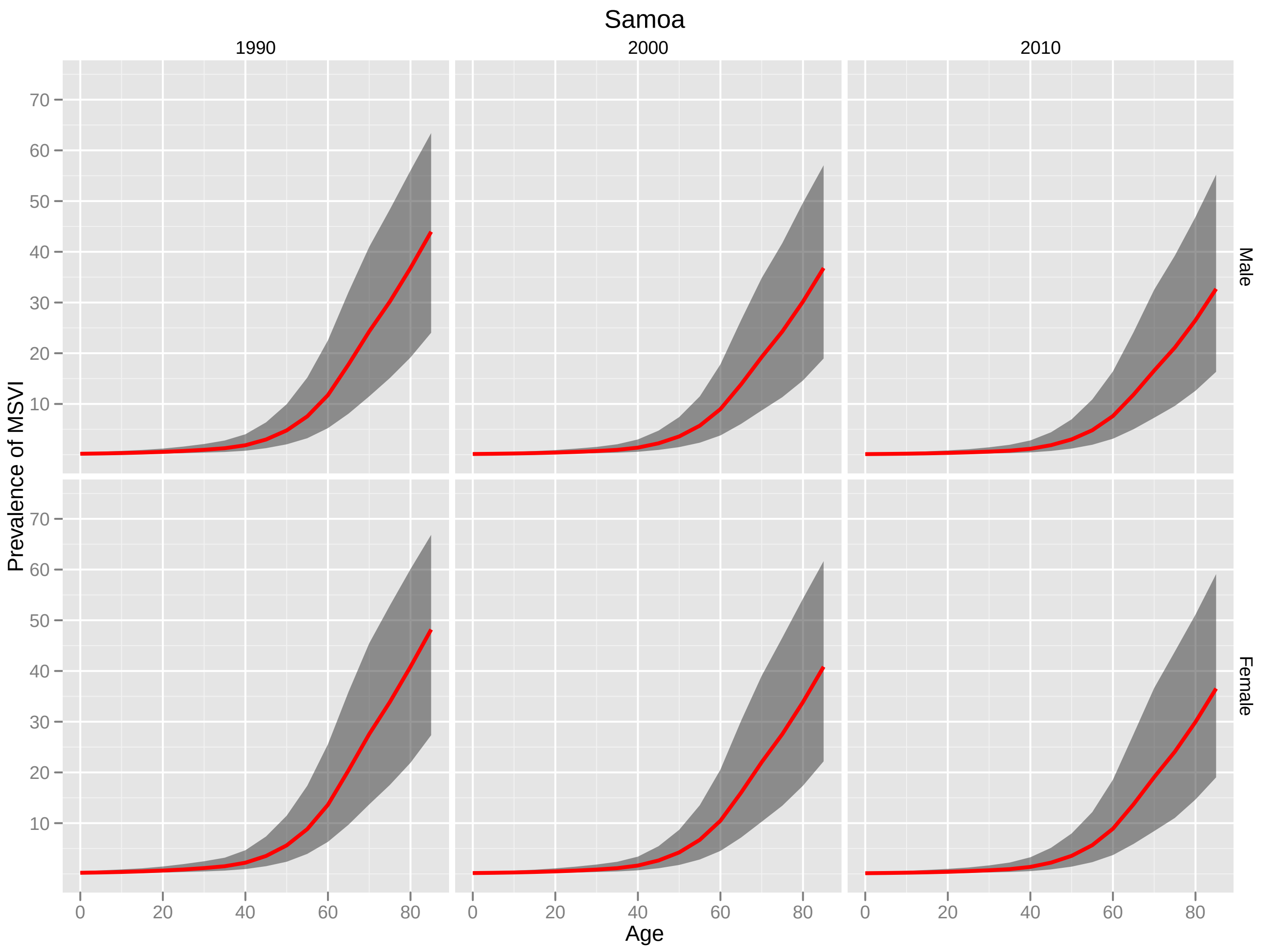
Samoa
1990
2000
2010
Male
Female
Prevalence of MSVI
Age
0
20
40
60
80
10
20
30
40
50
60
70

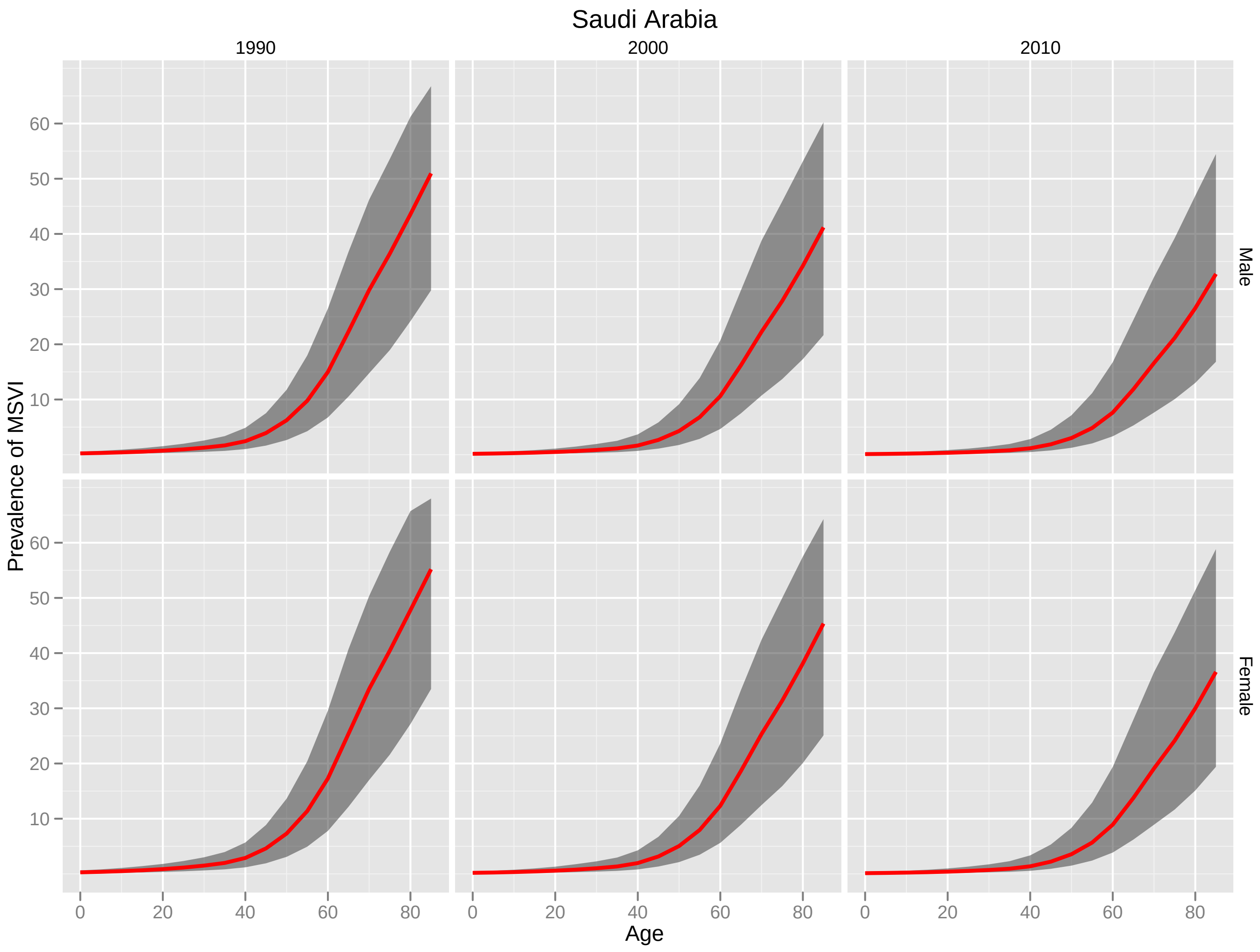
Saudi Arabia
1990
2000
2010
Male
Female
Prevalence of MSVI
Age
0
20
40
60
80
10
20
30
40
50
60

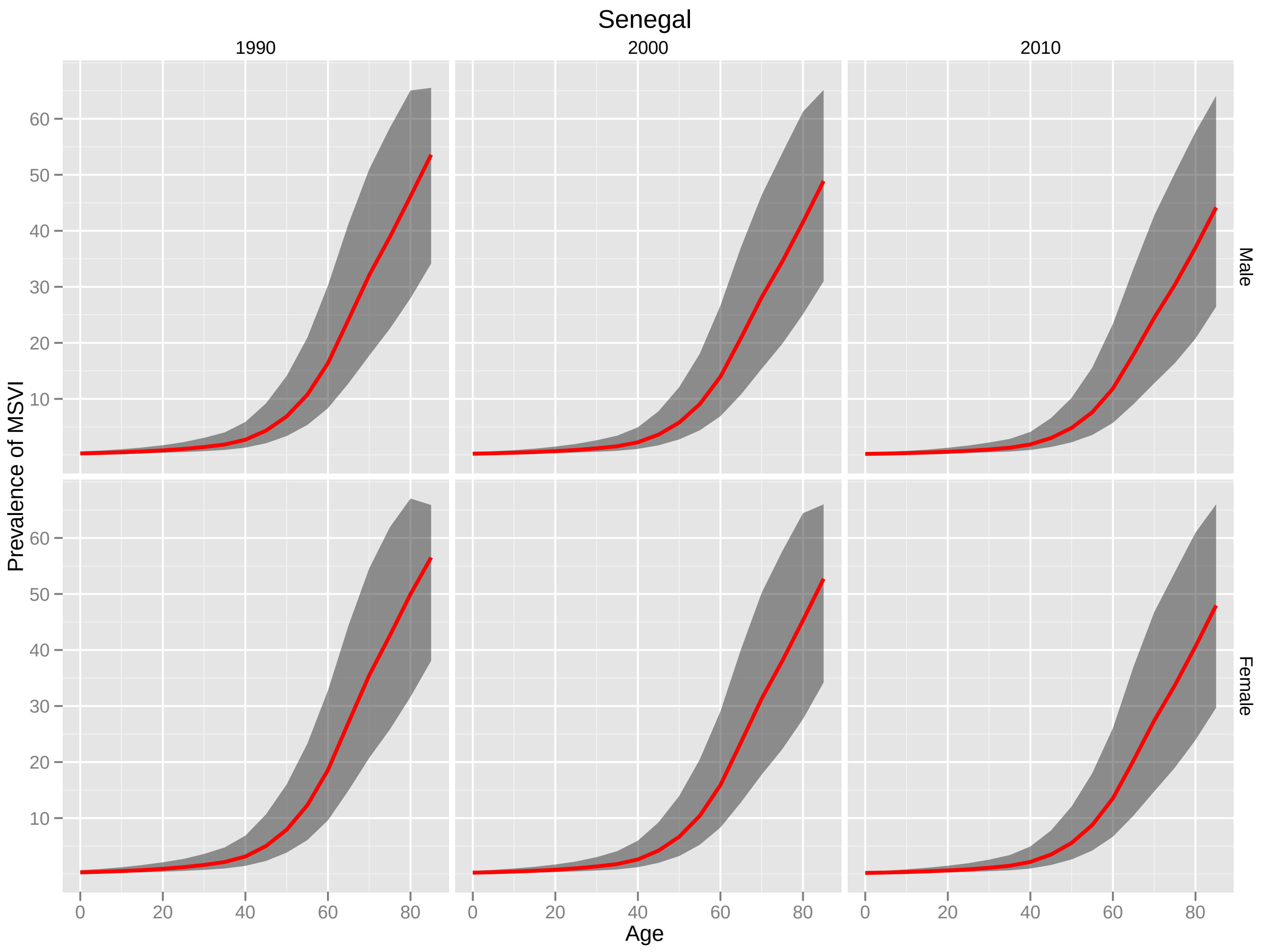
Senegal
1990
2000
2010
Male
Female
Prevalence of MSVI
Age
10
20
30
40
50
60
0
20
40
60
80

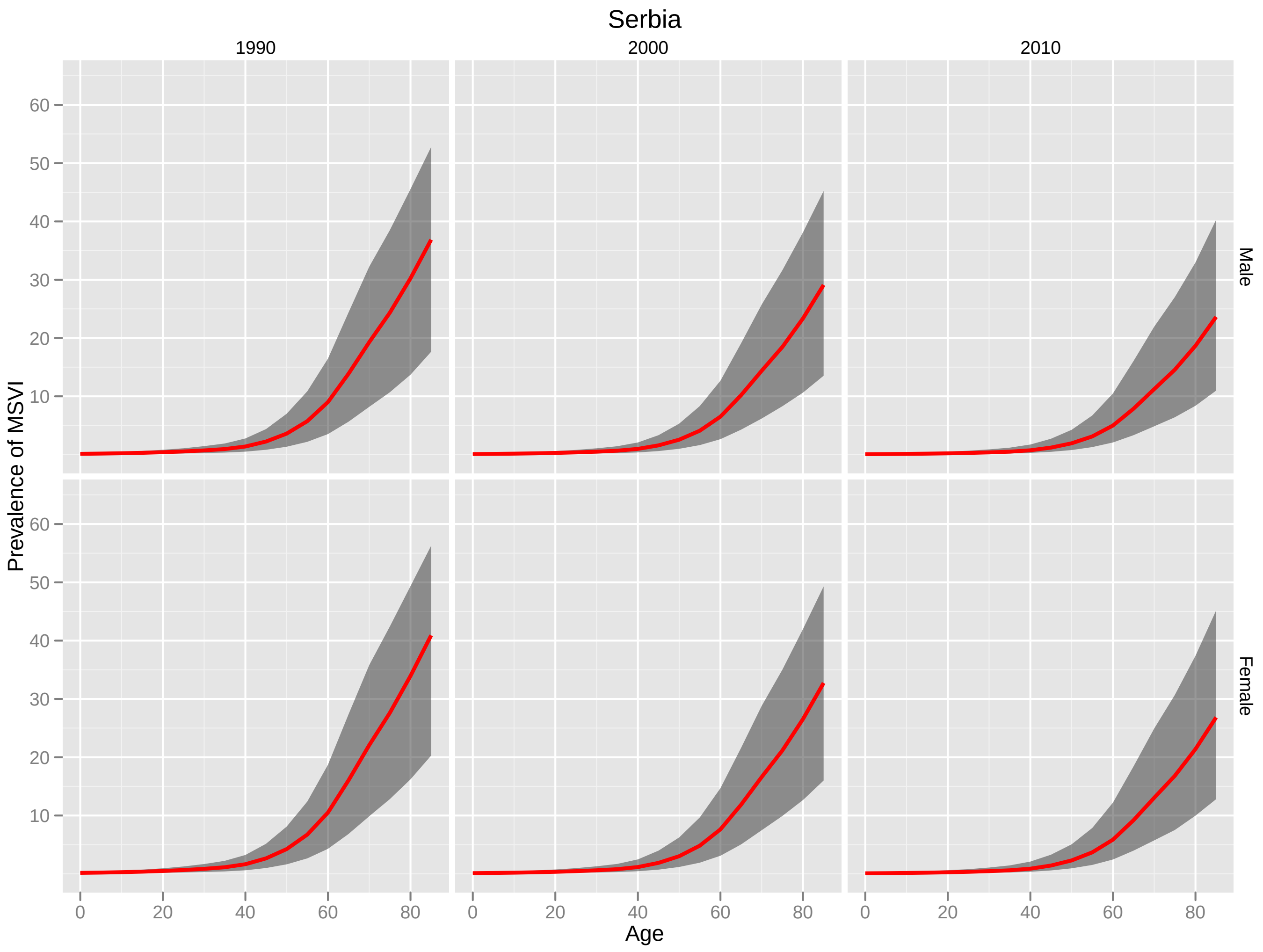

Serbia
1990
2000
2010
Male
Female
Prevalence of MSVI
Age
60
50
40
30
20
10
0
20
40
60
80

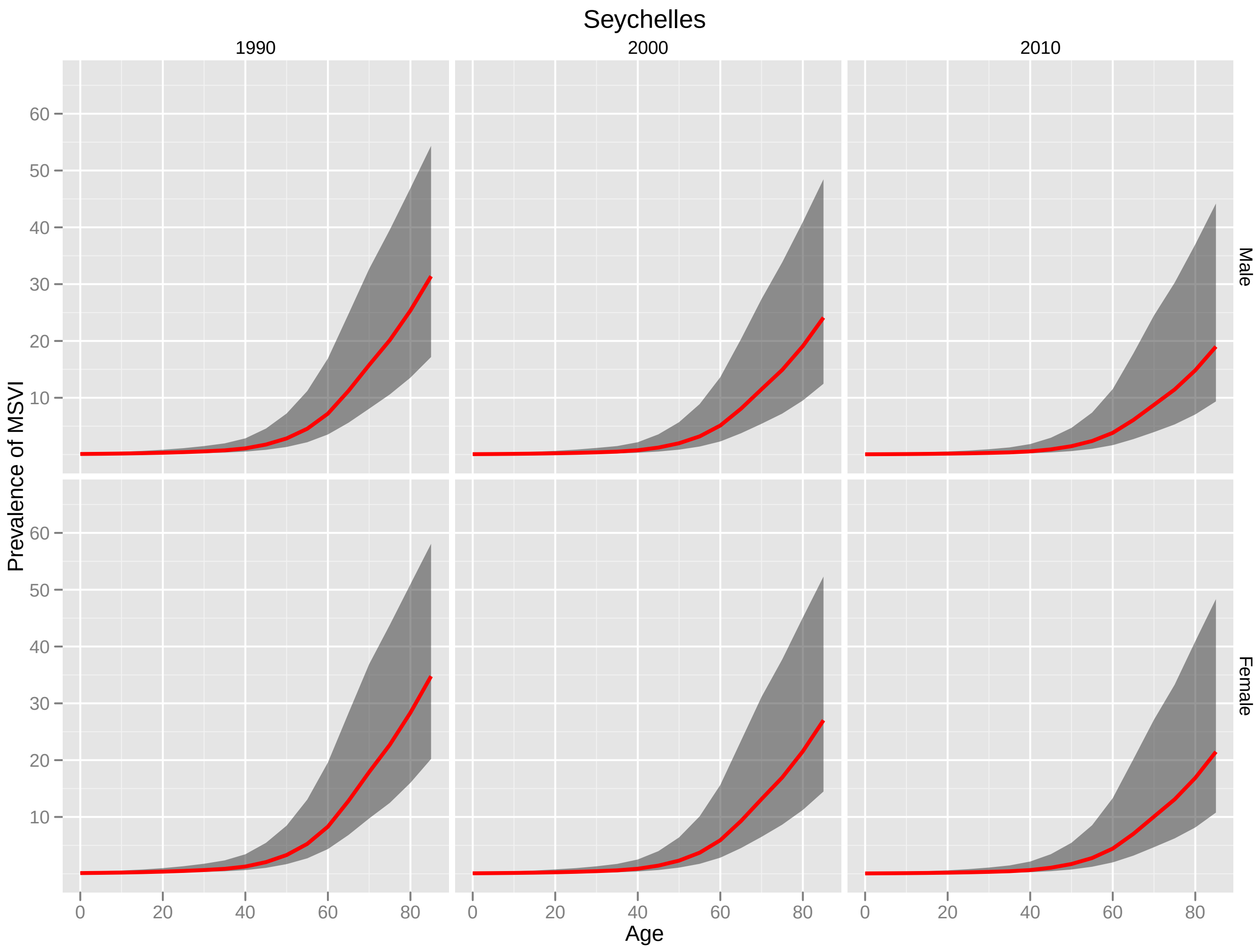
Seychelles
1990
2000
2010
Male
Female
Prevalence of MSVI
Age
10
20
30
40
50
60
0
20
40
60
80

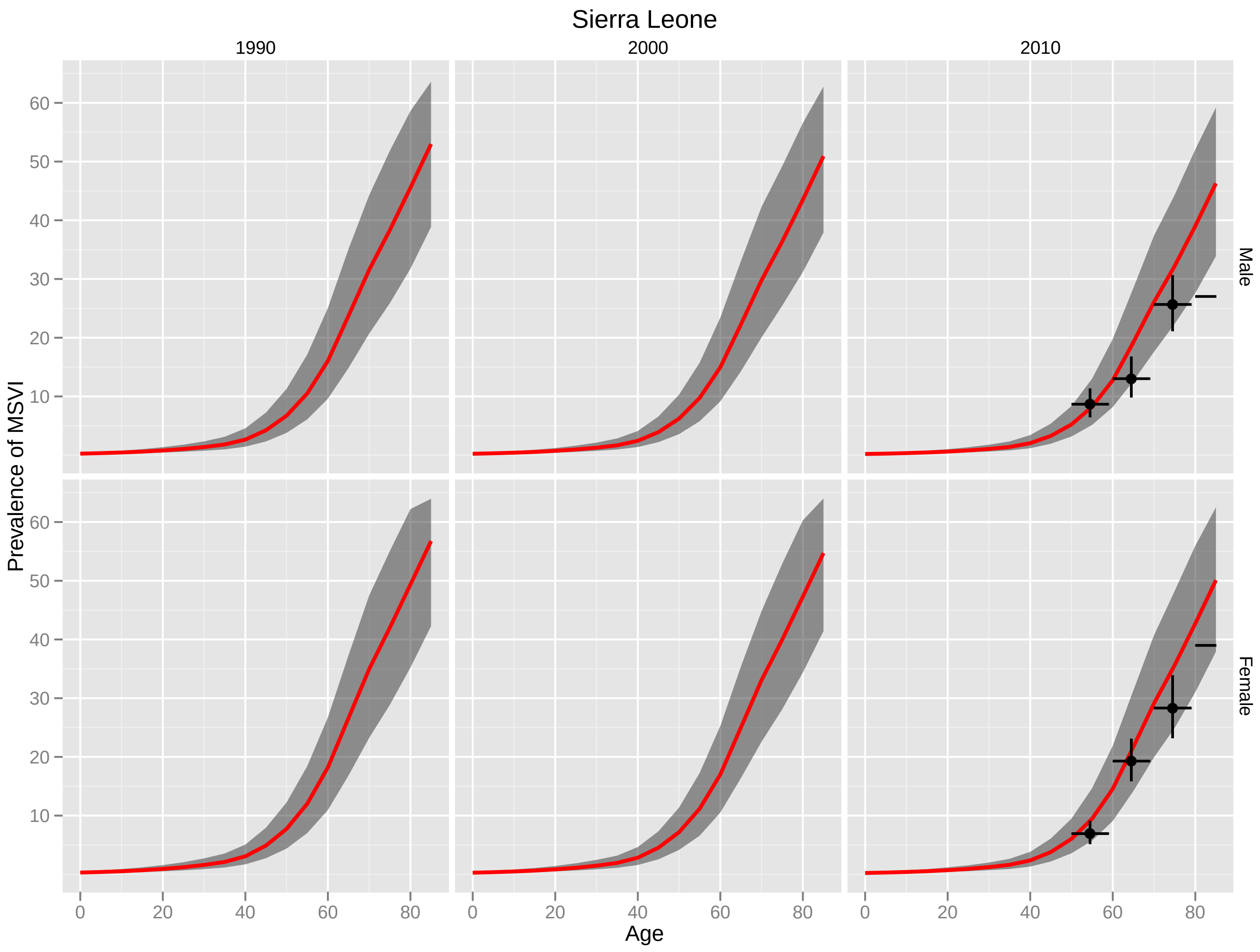
Sierra Leone
1990
2000
2010
Male
Female
Prevalence of MSVI
Age
10
20
30
40
50
60
0
20
40
60
80

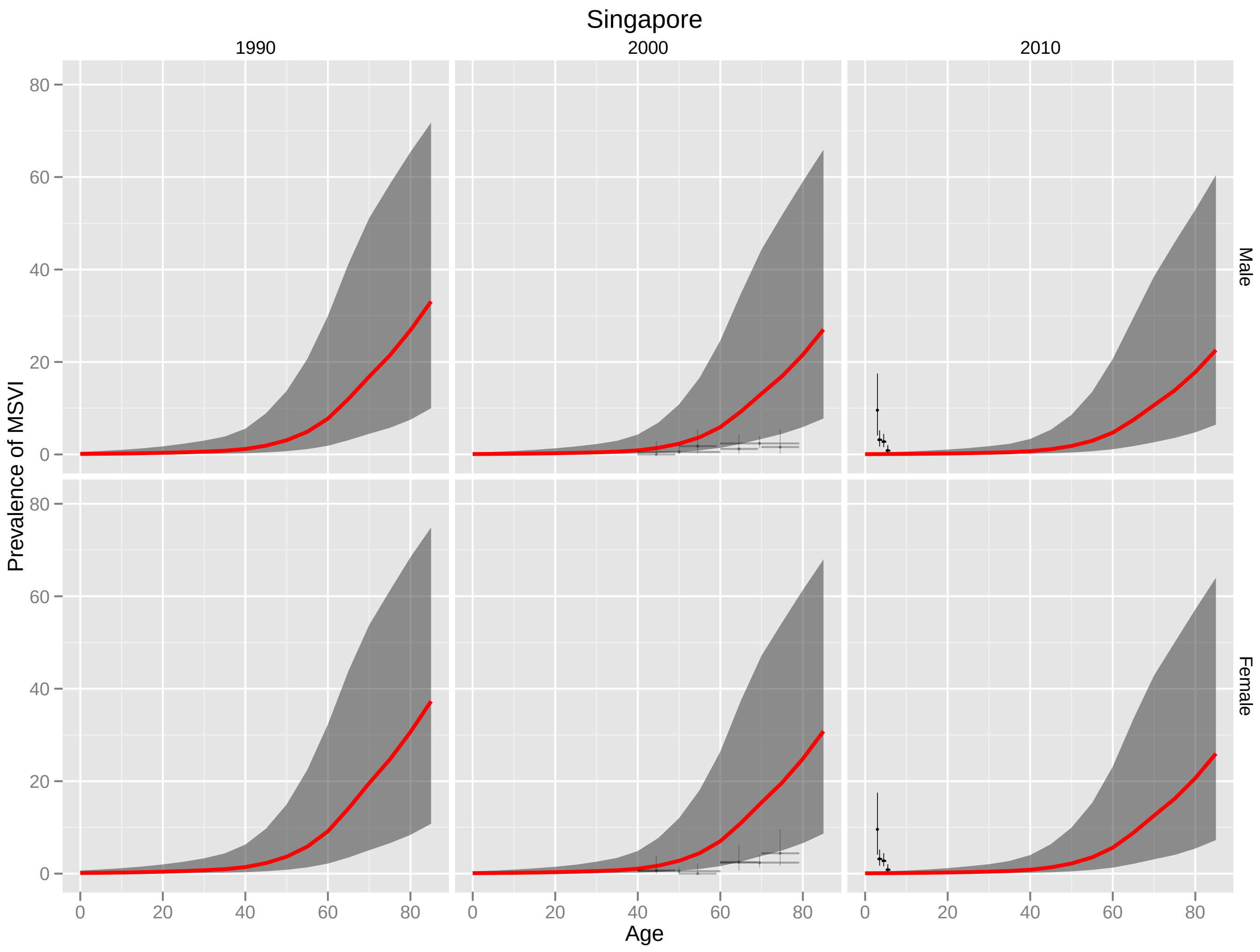
Singapore
1990
2000
2010
Male
Female
Prevalence of MSVI
Age
0
20
40
60
80

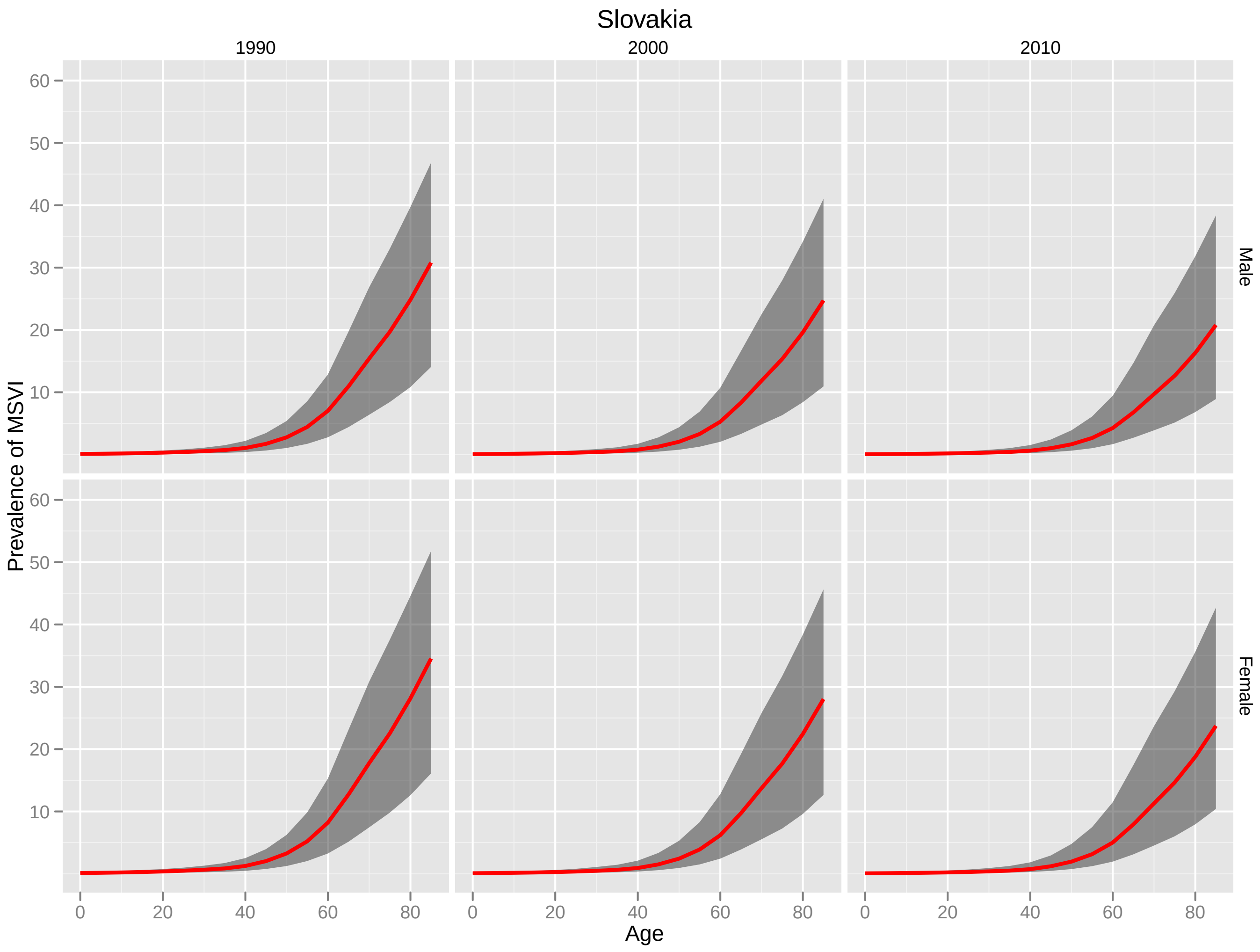
Slovakia
1990
2000
2010
Male
Female
Prevalence of MSVI
Age
10
20
30
40
50
60
0
20
40
60
80

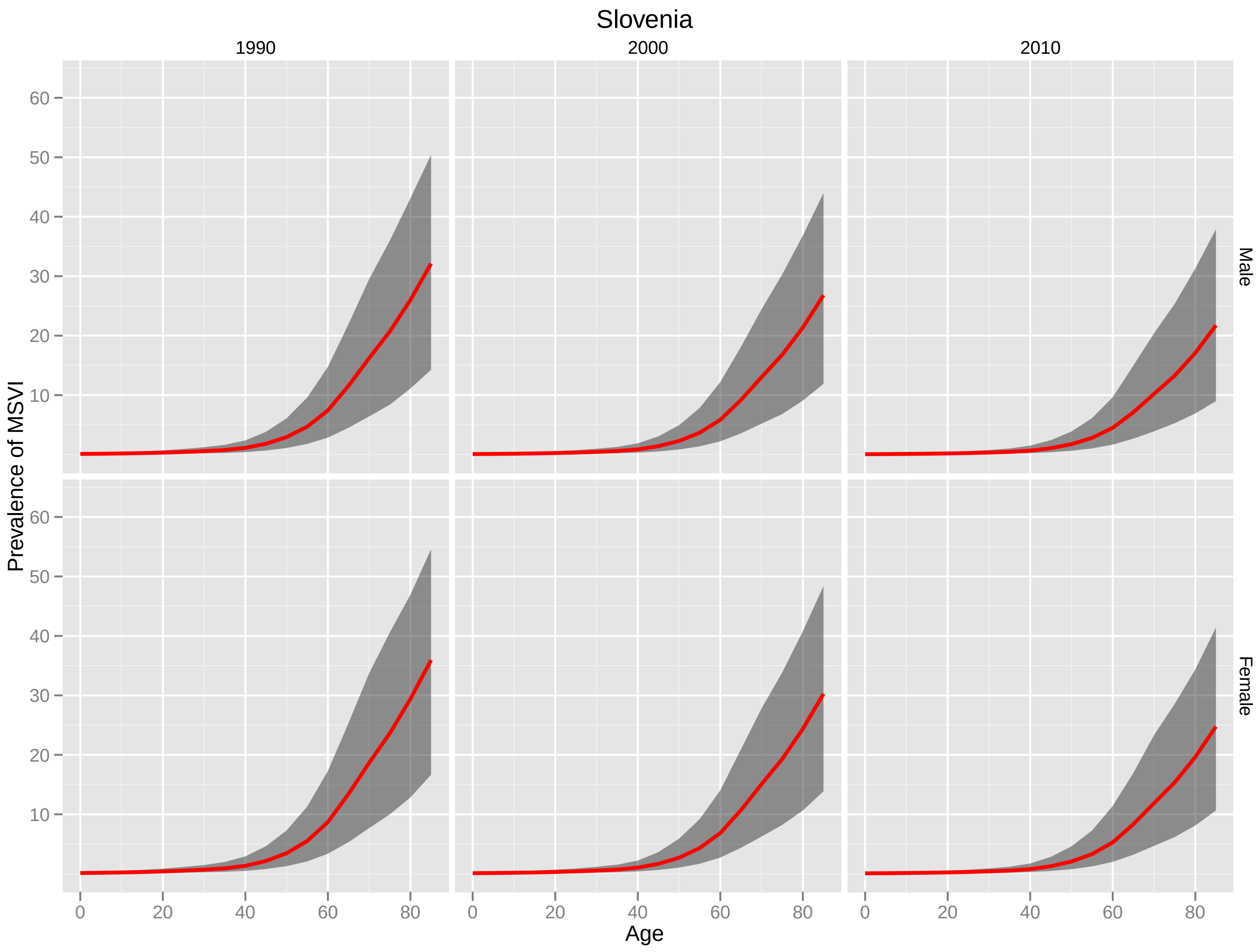
Slovenia
1990
2000
2010
Male
Female
Prevalence of MSVI
Age
10
20
30
40
50
60
0
20
40
60
80

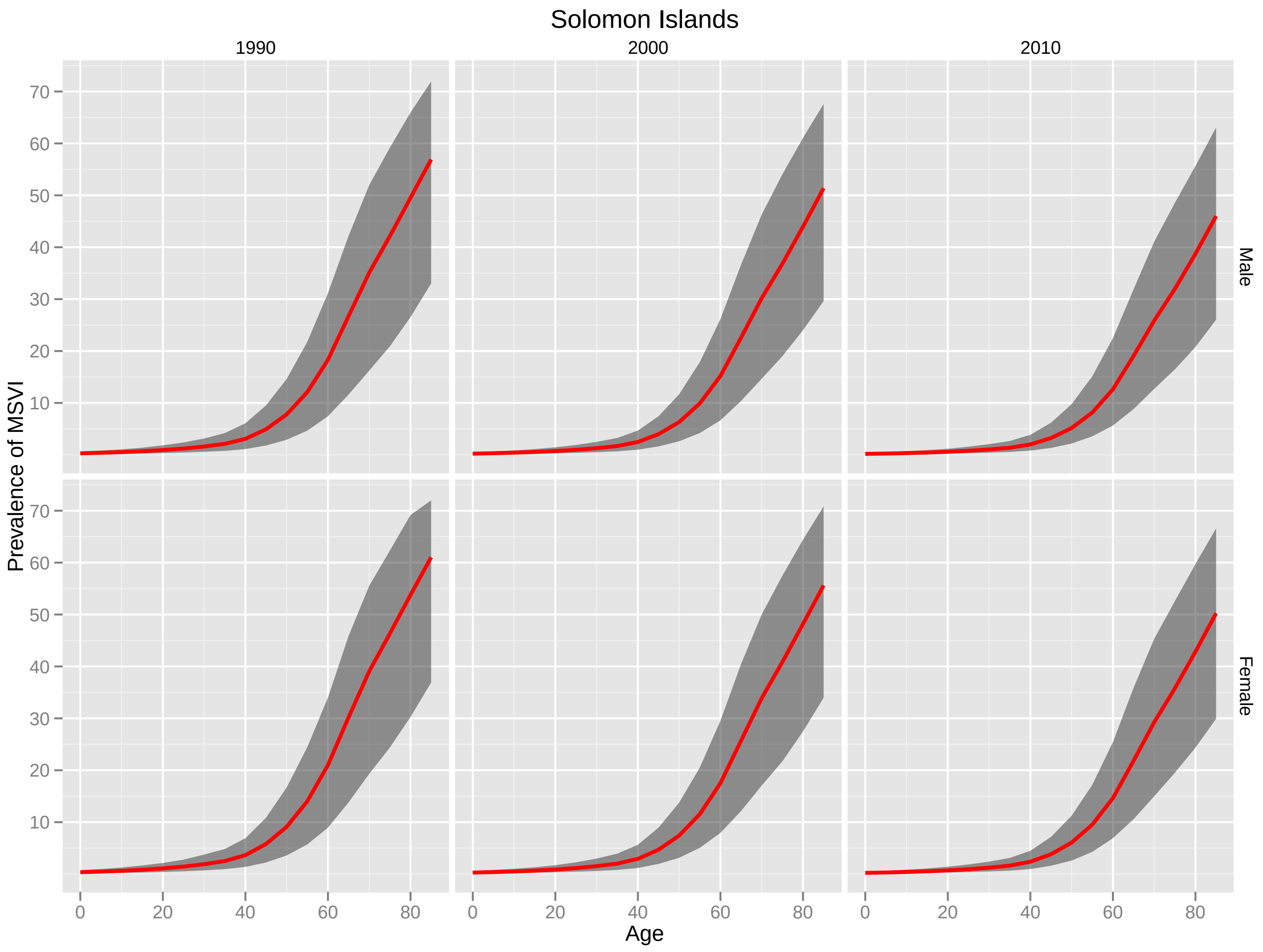
Solomon Islands
1990
2000
2010
Male
Female
Prevalence of MSVI
Age
10
20
30
40
50
60
70
0
20
40
60
80

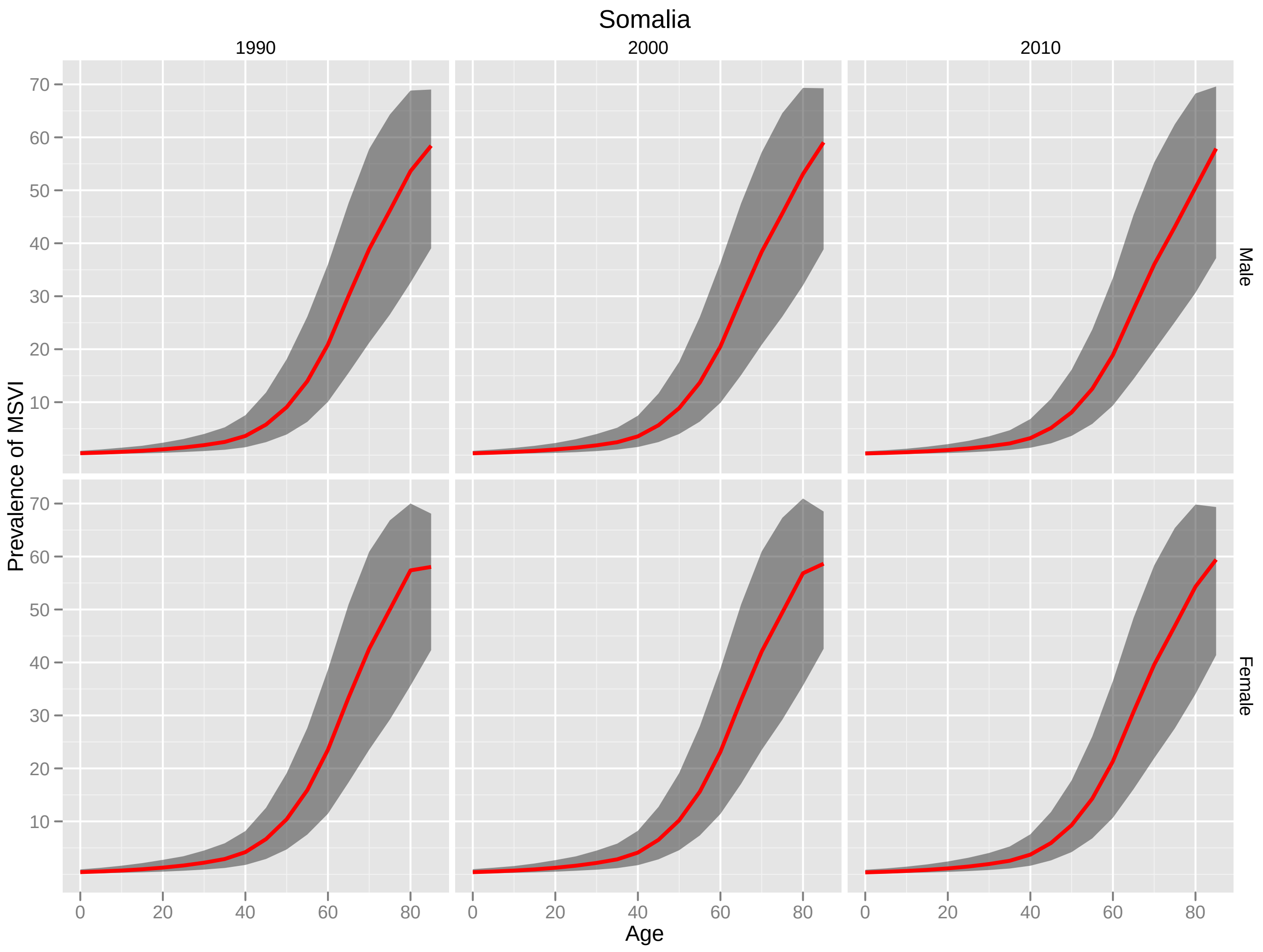
Somalia
1990
2000
2010
Male
Female
Prevalence of MSVI
Age
10
20
30
40
50
60
70
0
20
40
60
80

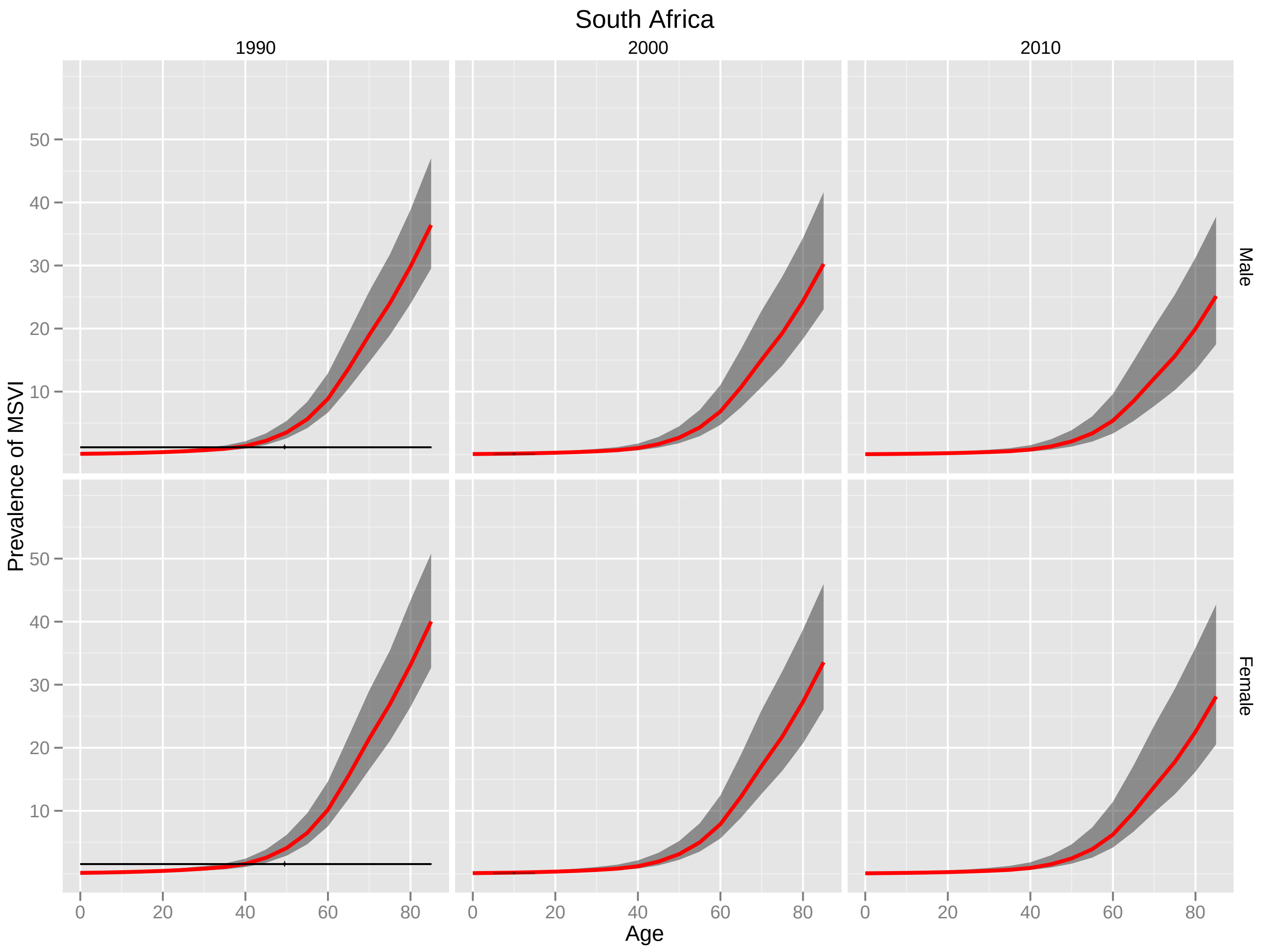
South Africa
1990
2000
2010
Male
Female
Prevalence of MSVI
Age
10
20
30
40
50
0
20
40
60
80

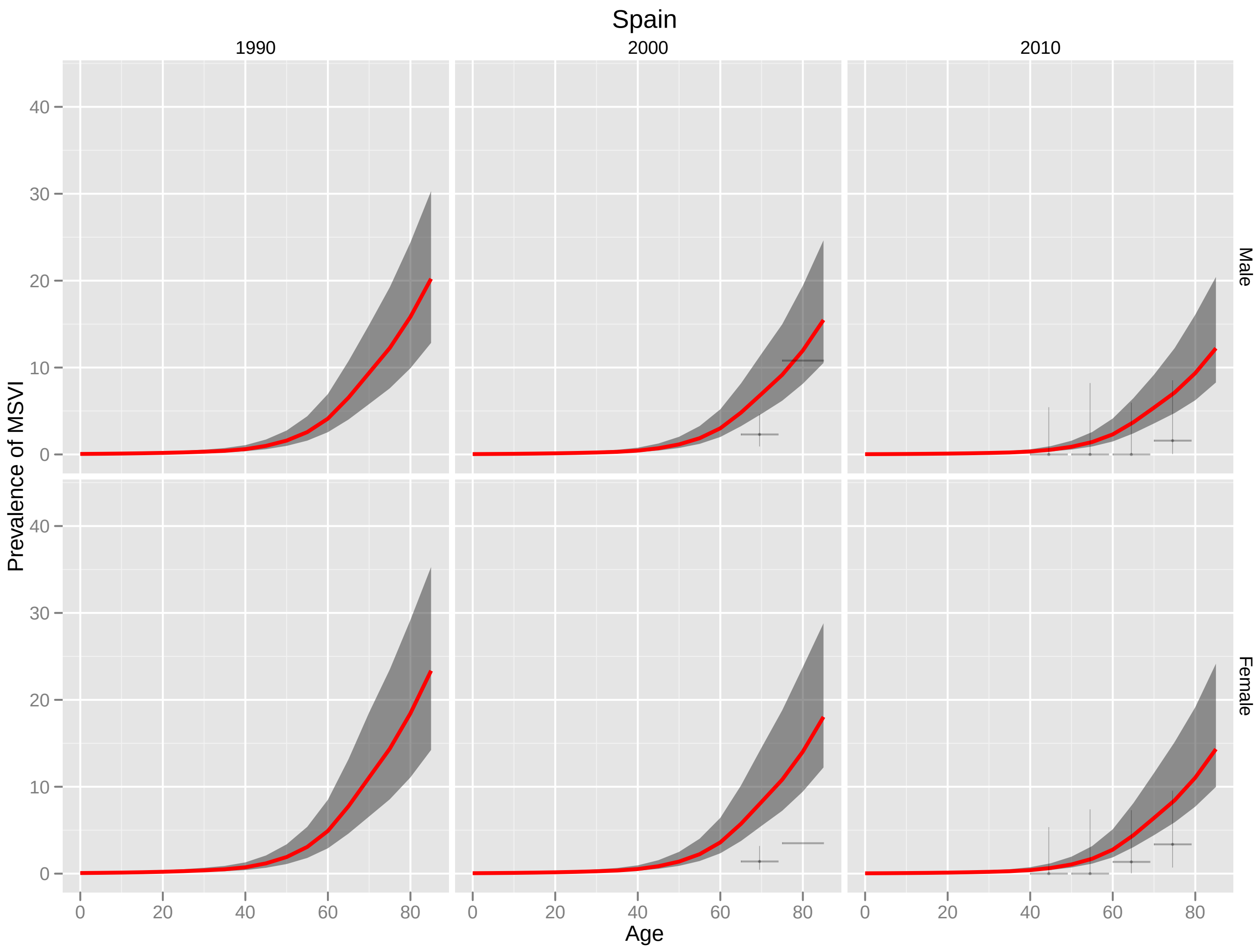
Spain
1990
2000
2010
Male
Female
Prevalence of MSVI
Age
0
10
20
30
40
0
20
40
60
80

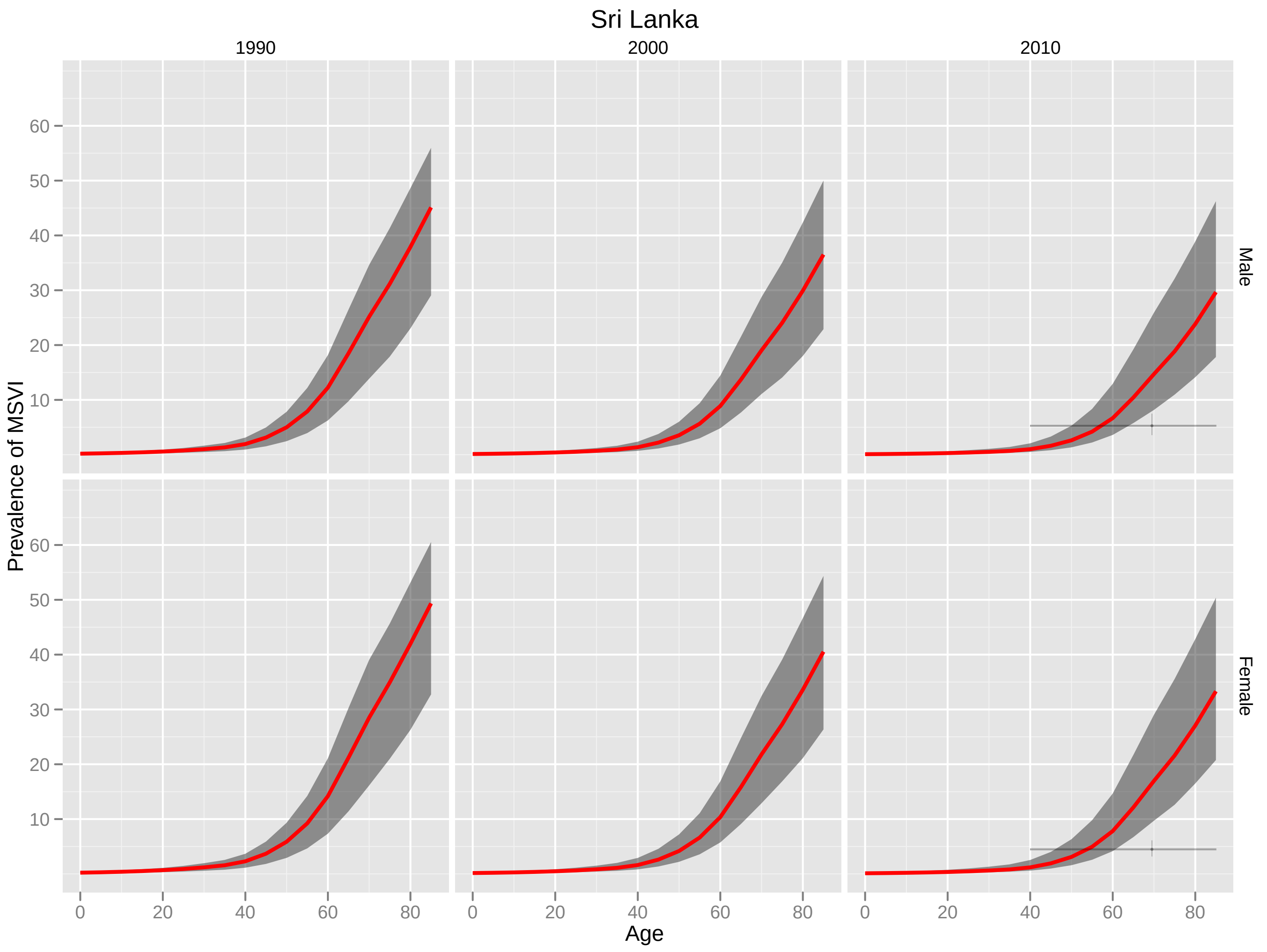

Sri Lanka
1990
2000
2010
Male
Female
Prevalence of MSVI
Age
0
20
40
60
80
10
20
30
40
50
60

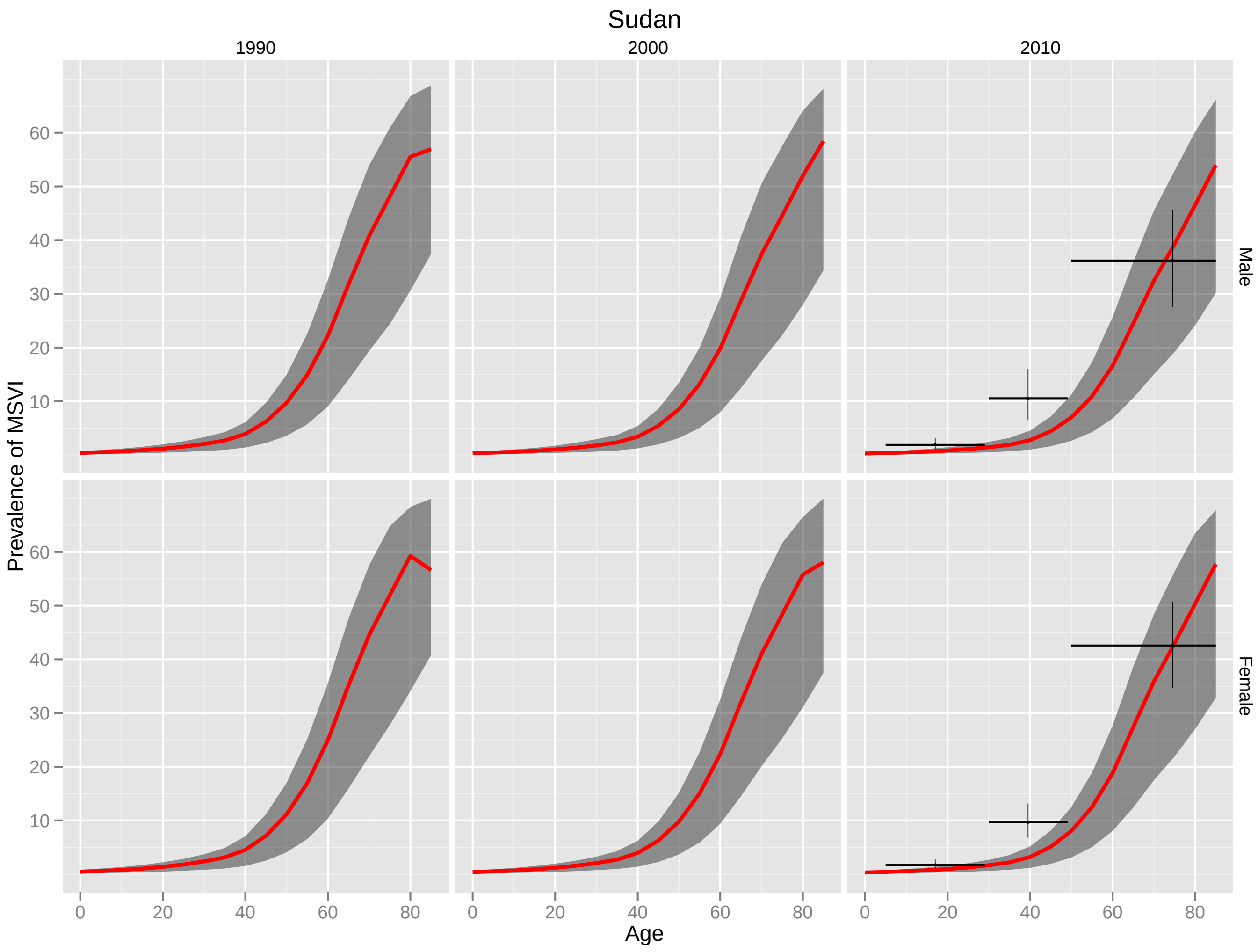
Sudan
1990
2000
2010
Male
Female
Prevalence of MSVI
Age
0
20
40
60
80
10
30
50

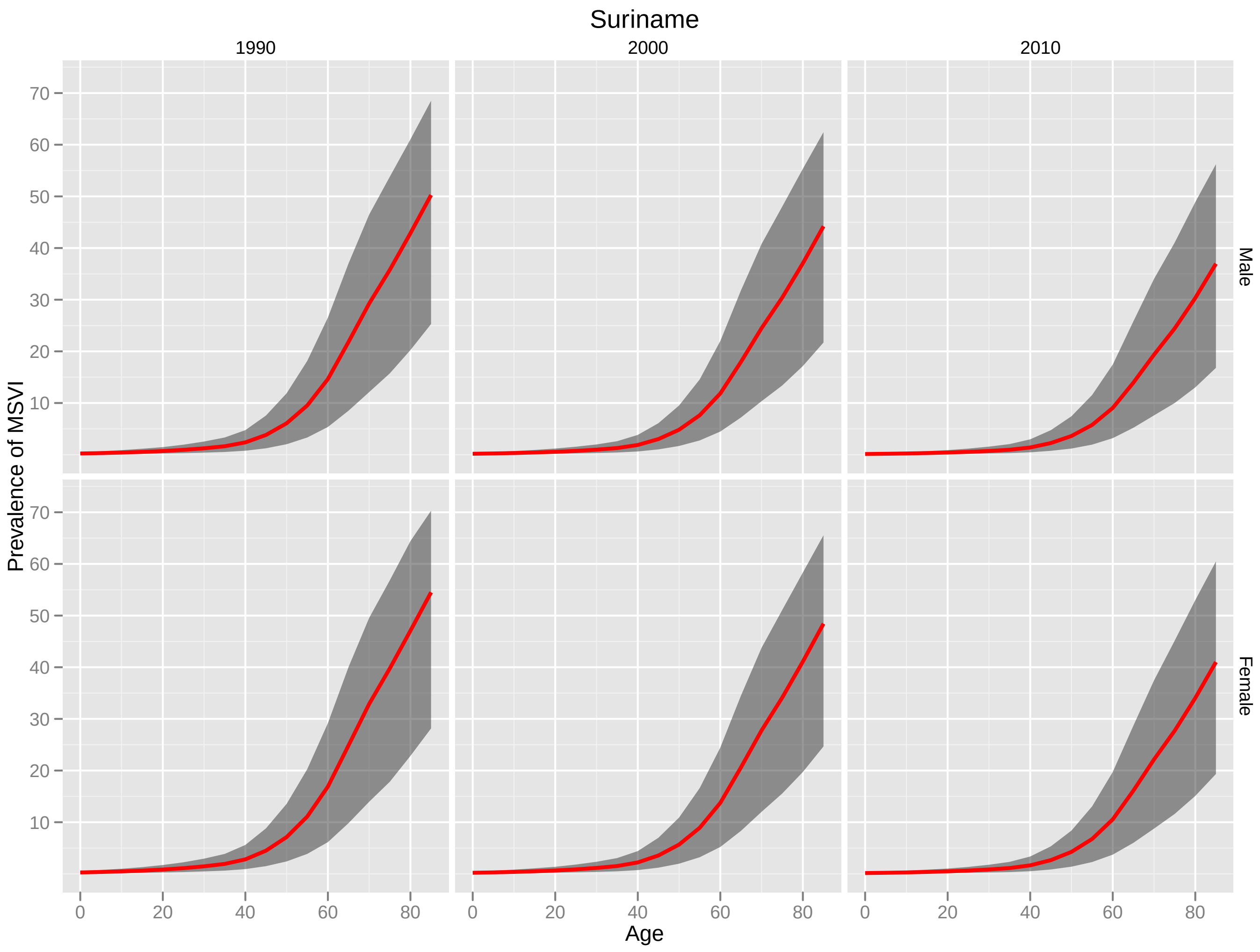

Suriname
1990
2000
2010
Male
Female
Prevalence of MSVI
Age
10
20
30
40
50
60
70
0
20
40
60
80

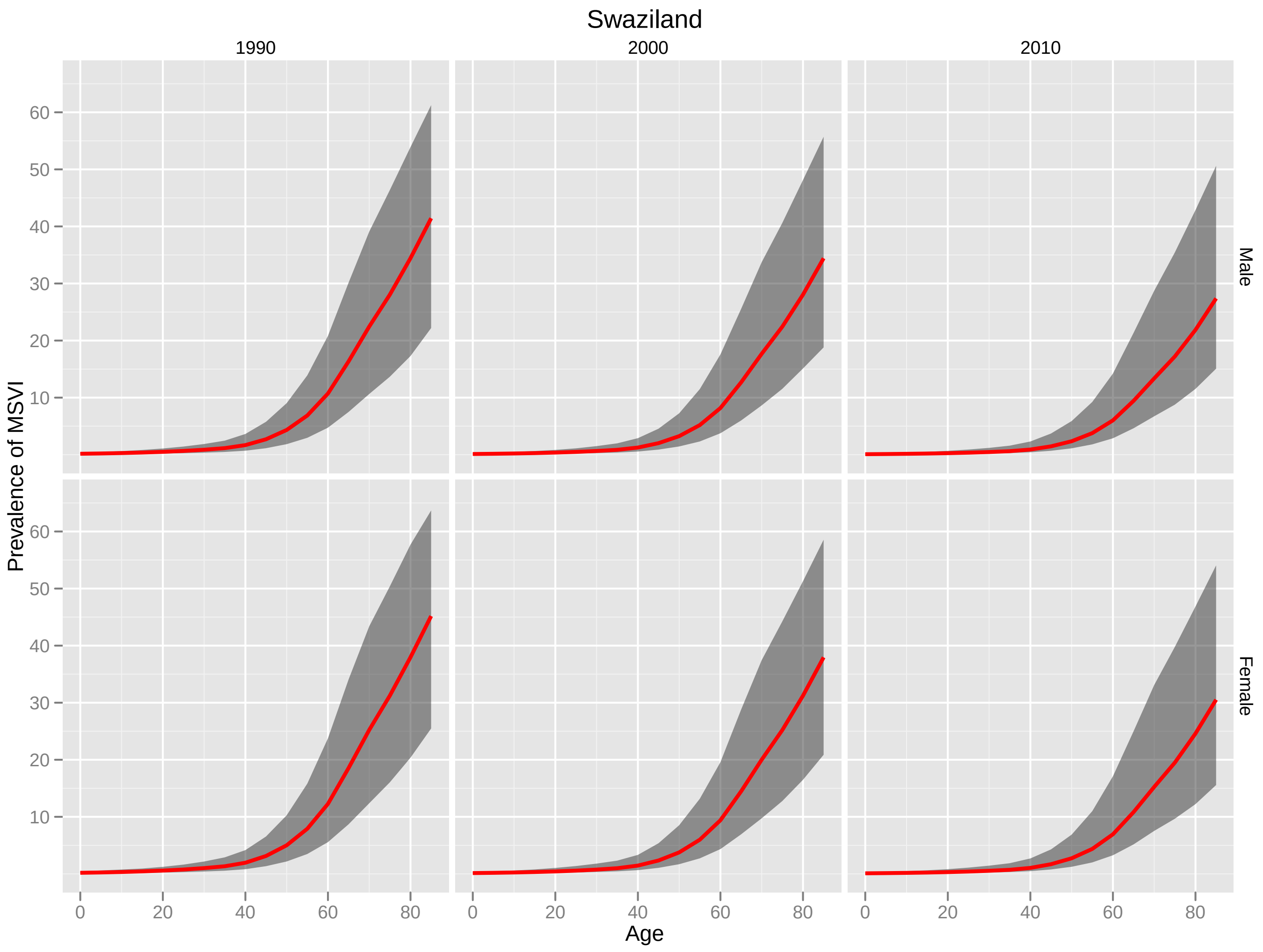
Swaziland
1990
2000
2010
Male
Female
Prevalence of MSVI
Age
0
20
40
60
80
10
20
30
40
50
60

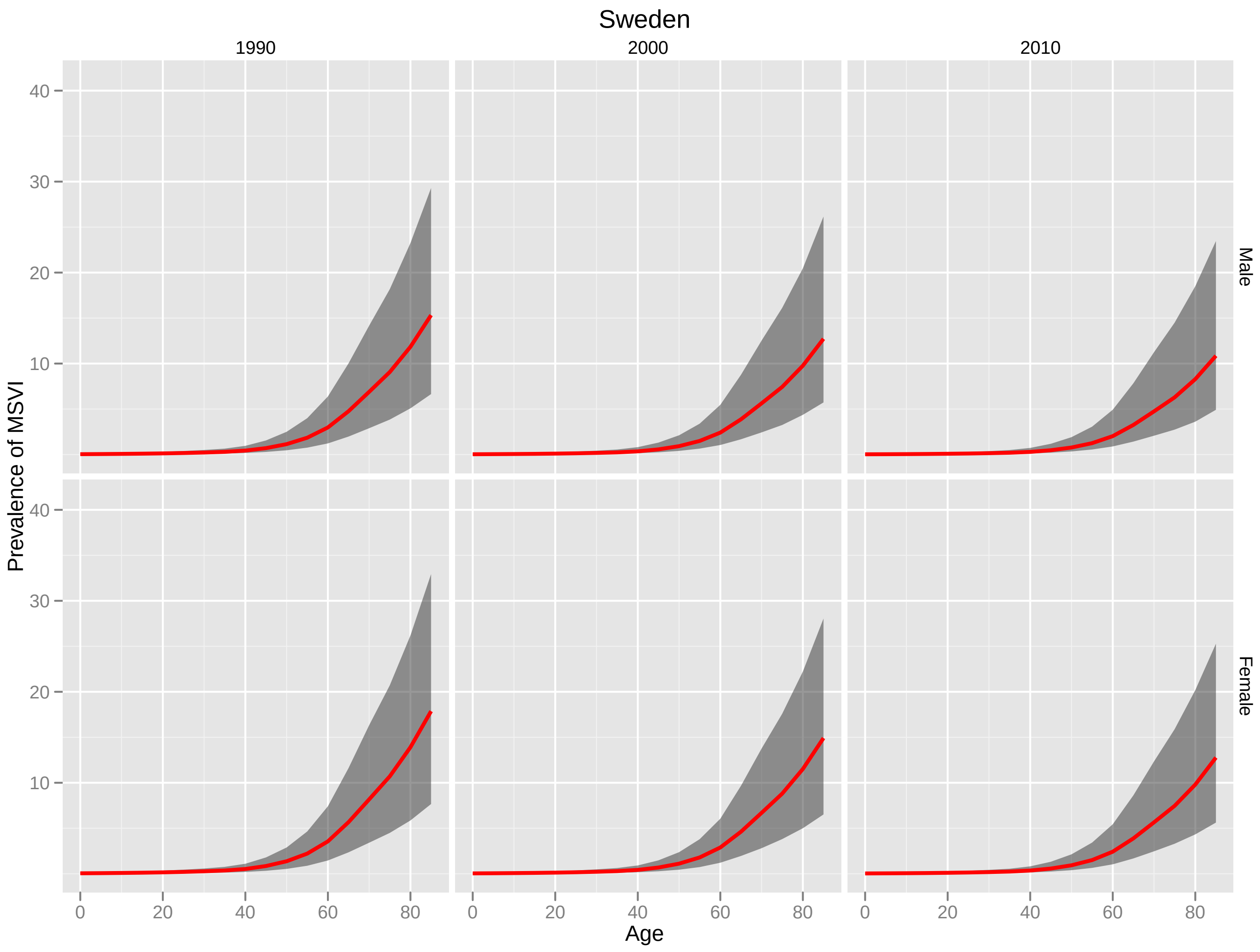
Sweden
1990
2000
2010
Male
Female
Prevalence of MSVI
Age
0
20
40
60
80
10
20
30
40

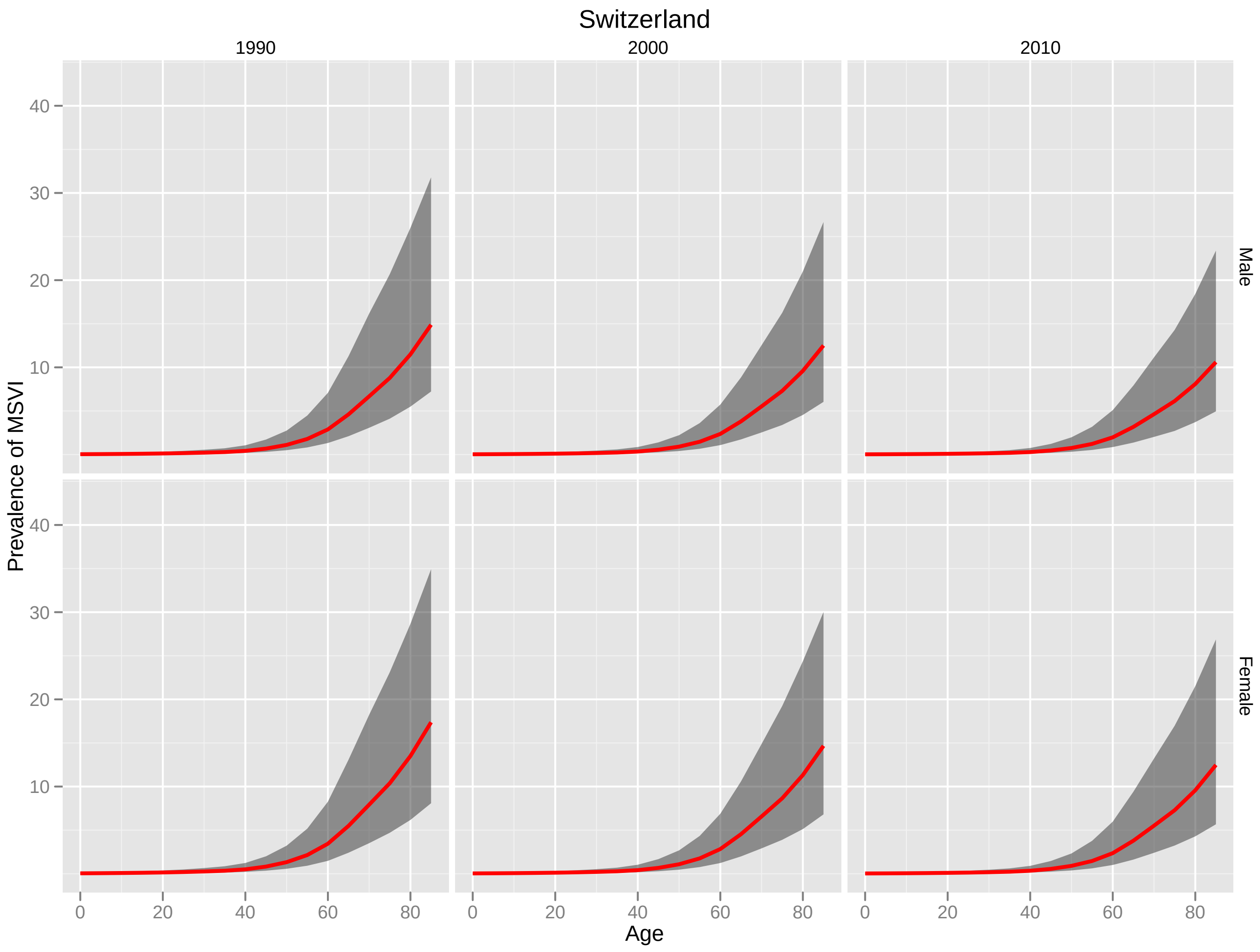
Switzerland
1990
2000
2010
Male
Female
Prevalence of MSVI
10
20
30
40
Age
0
20
40
60
80

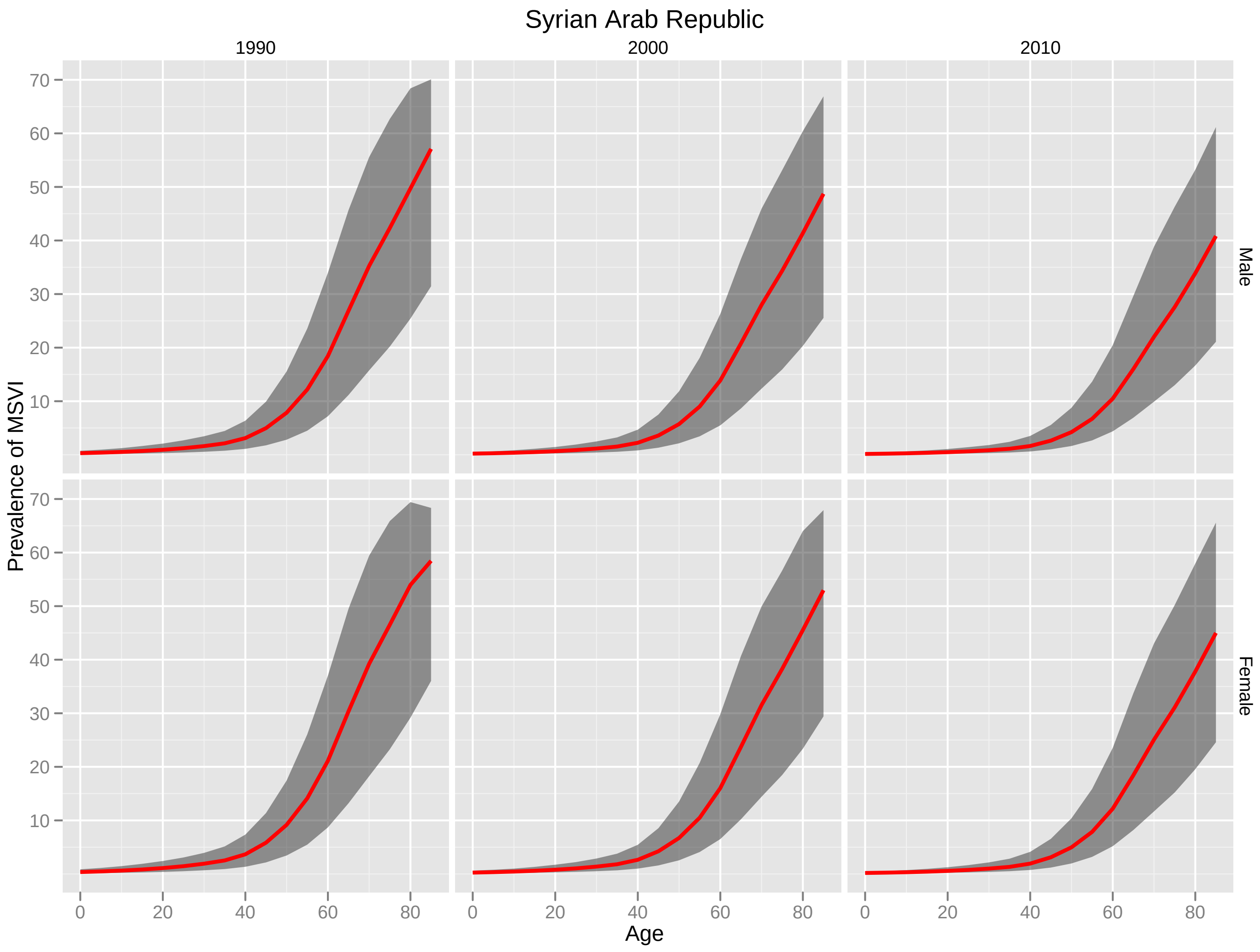
Syrian Arab Republic
1990
2000
2010
Male
Female
Prevalence of MSVI
Age
10
20
30
40
50
60
70
0
20
40
60
80

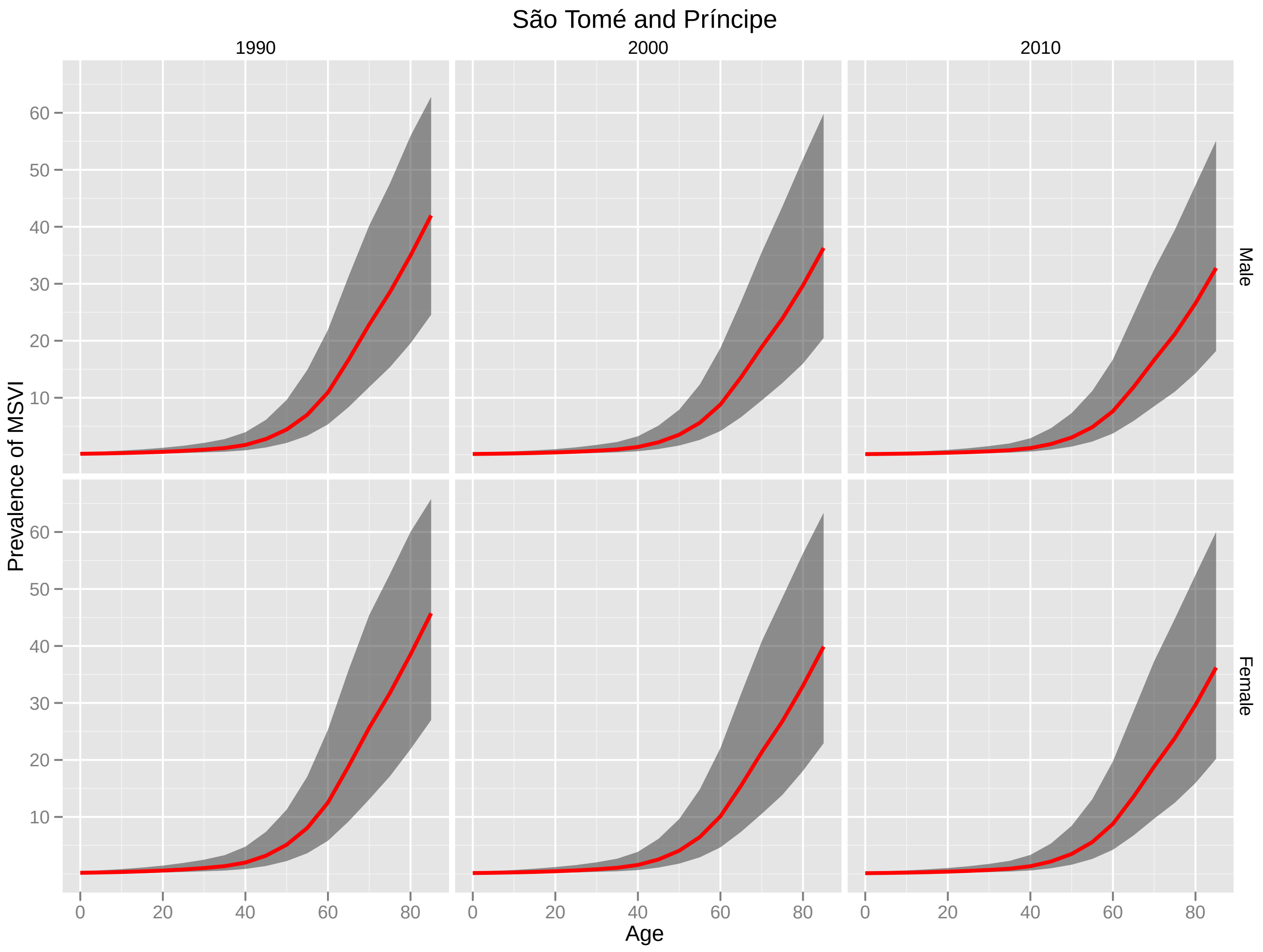
São Tomé and Príncipe
1990
2000
2010
Male
Female
Prevalence of MSVI
Age
10
20
30
40
50
60
0
20
40
60
80

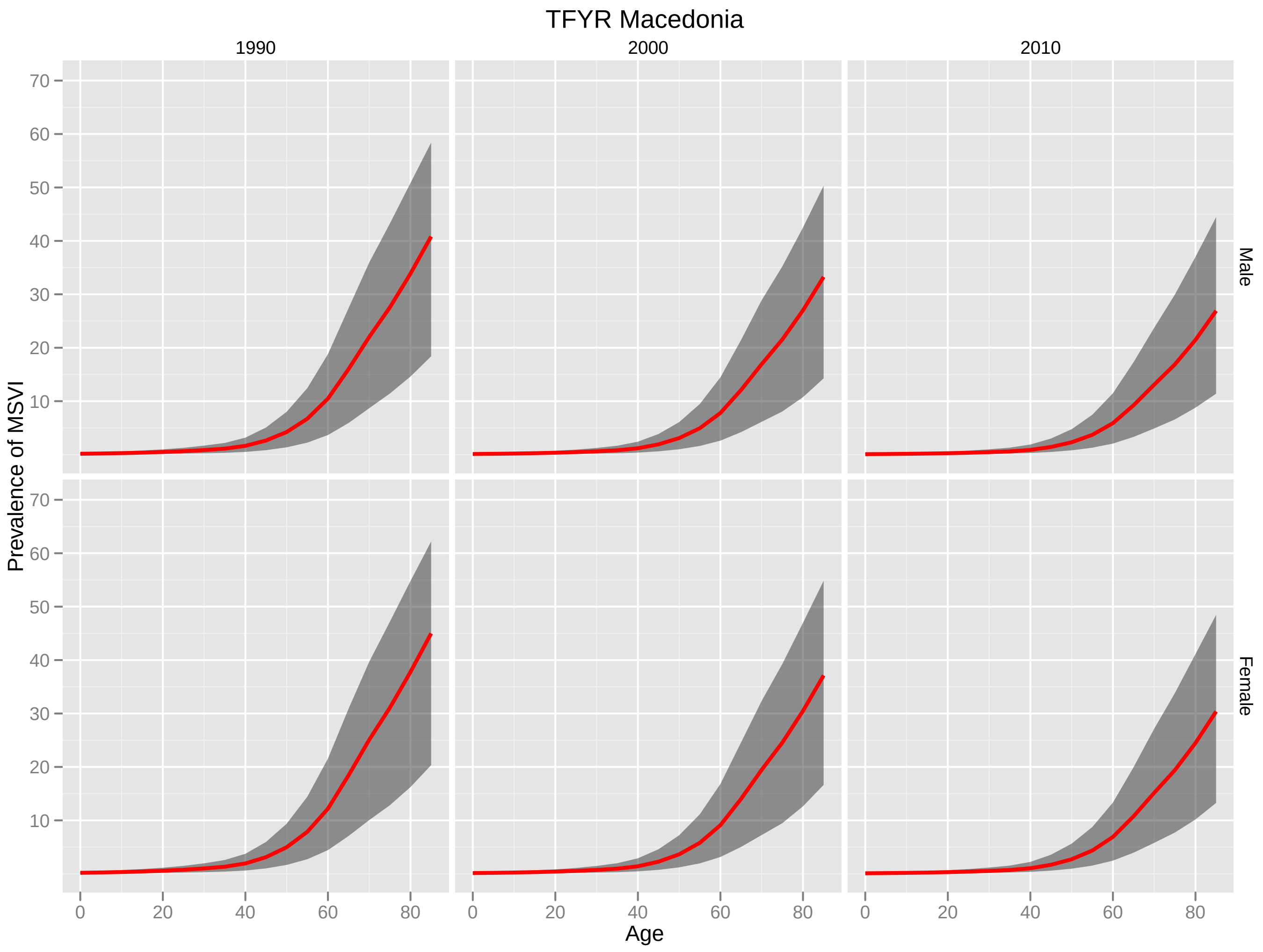

TFYR Macedonia
1990
2000
2010
Male
Female
Prevalence of MSVI
Age
10
20
30
40
50
60
70
0
20
40
60
80

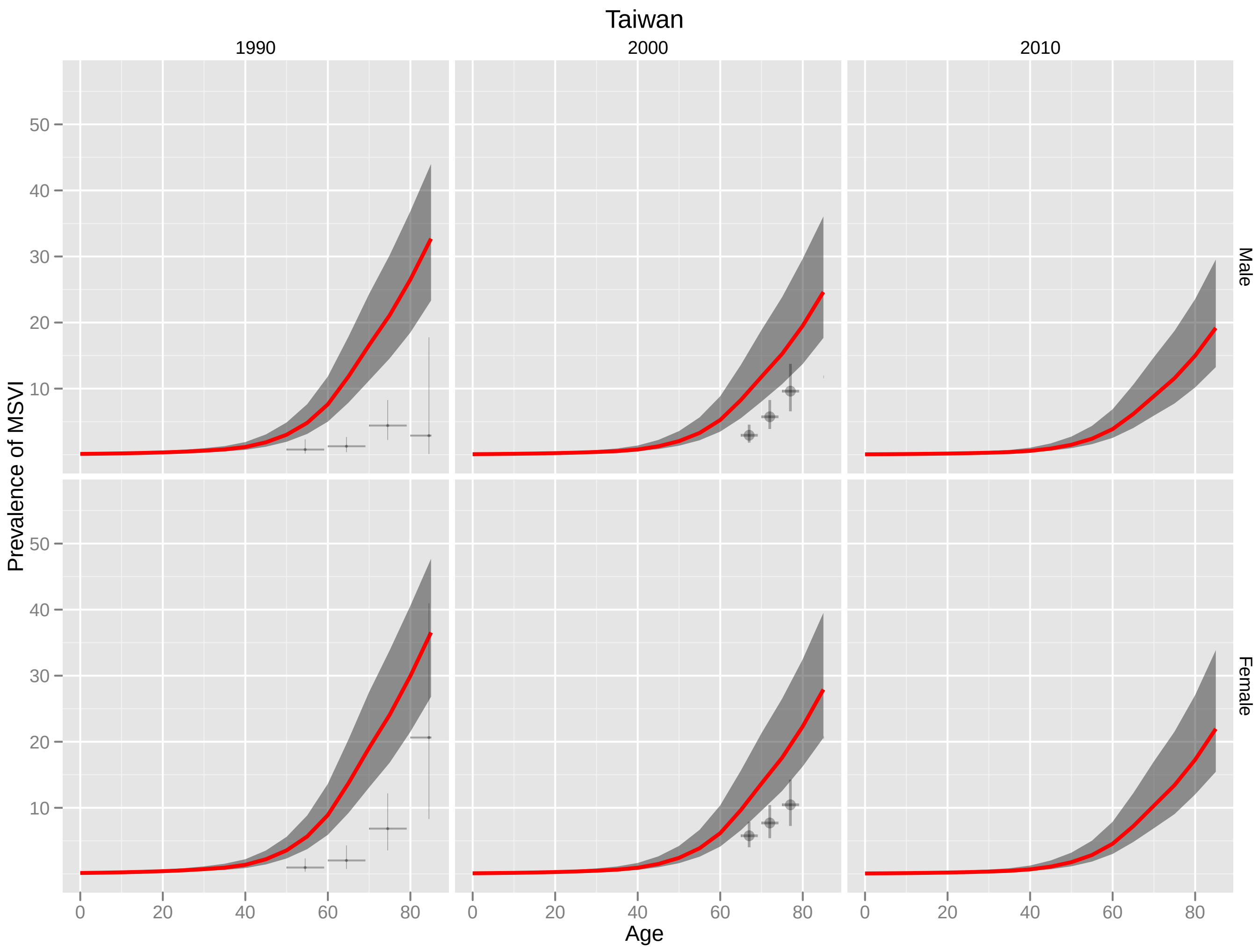

Taiwan
1990
2000
2010
Male
Female
Prevalence of MSVI
Age
0
20
40
60
80
10
20
30
40
50

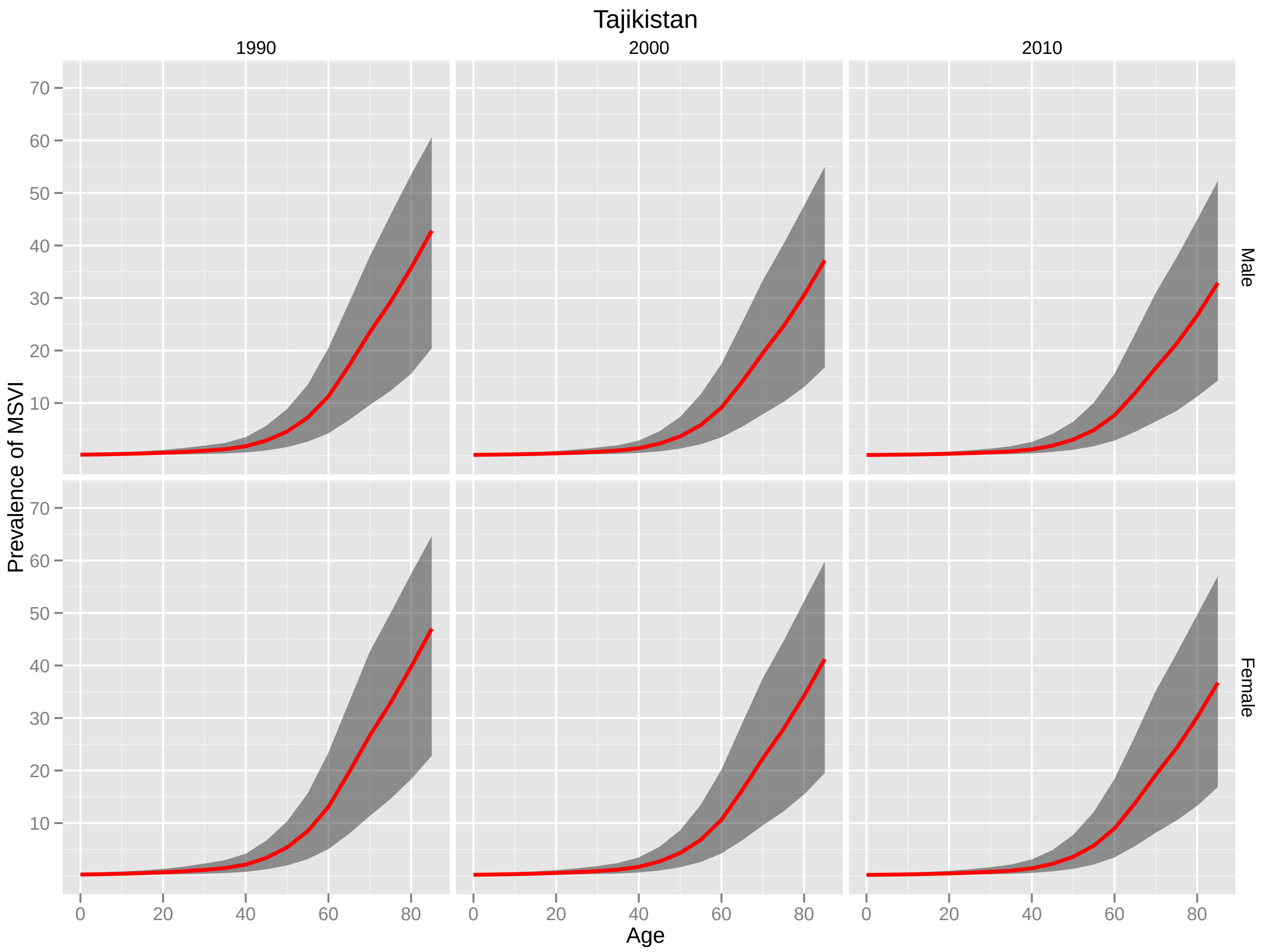
Tajikistan
1990
2000
2010
Male
Female
Prevalence of MSVI
Age
0
20
40
60
80
10
20
30
40
50
60
70

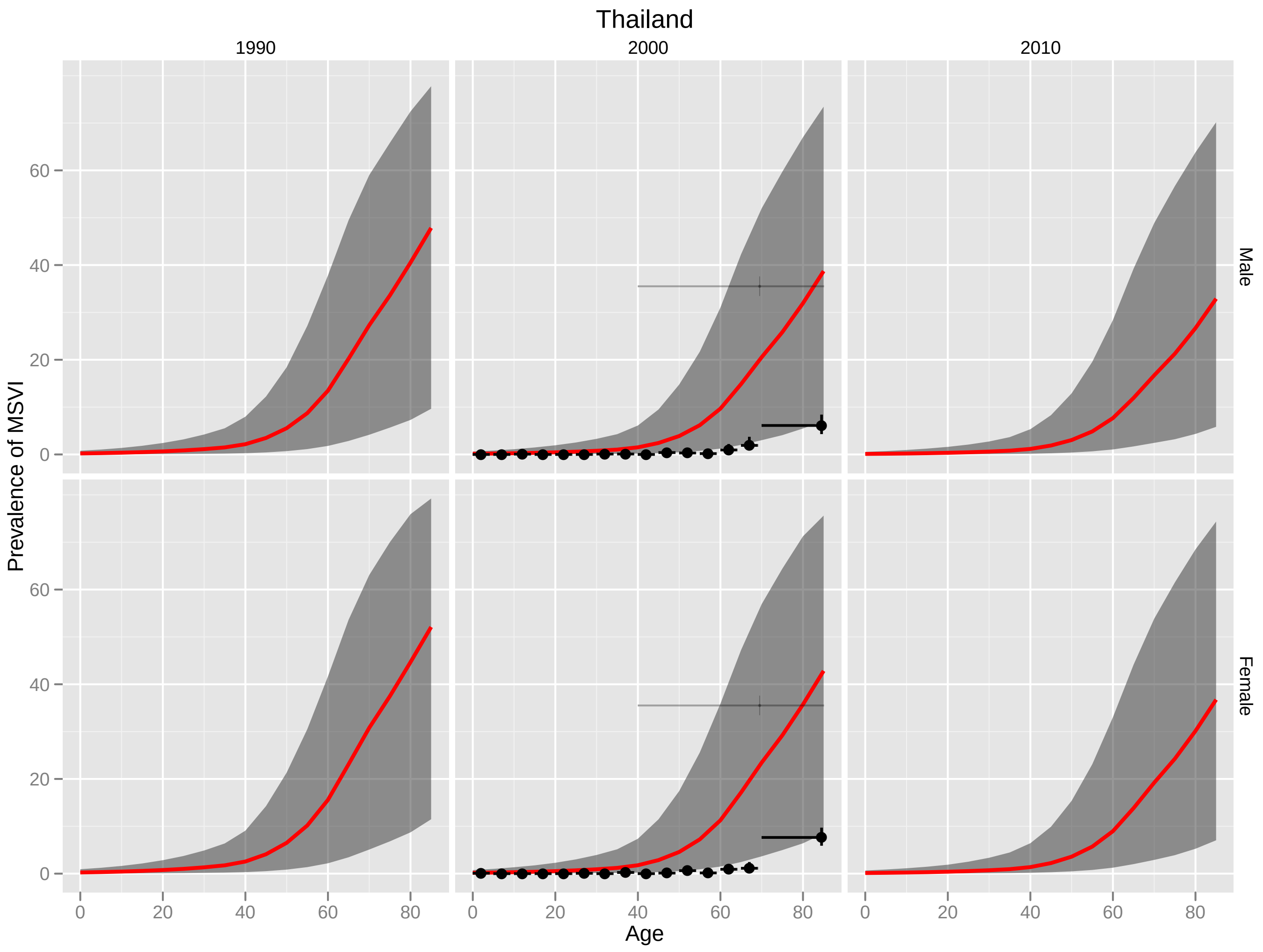

Thailand
1990
2000
2010
Male
Female
Prevalence of MSVI
Age
0
20
40
60
80

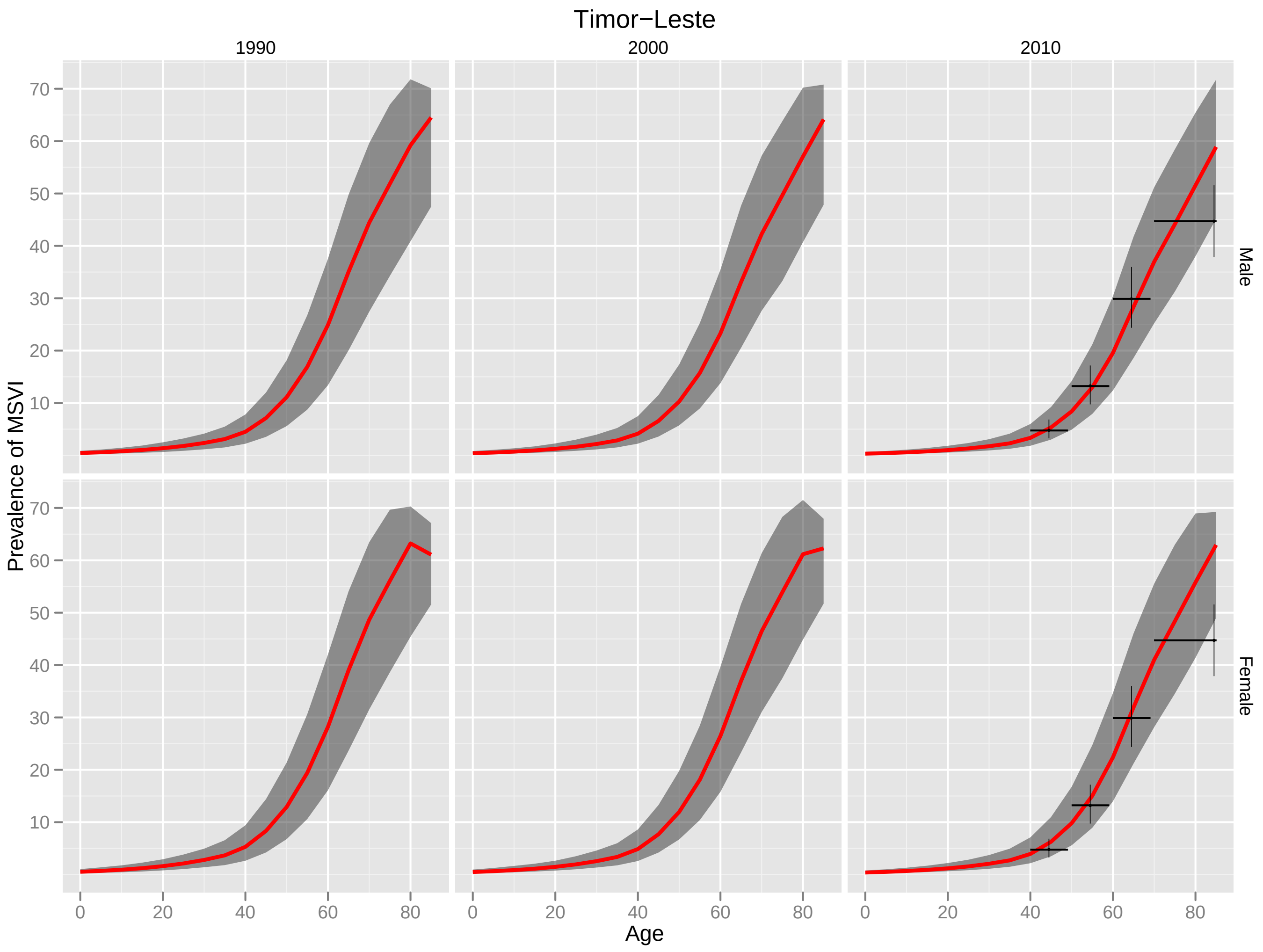
Timor−Leste
1990
2000
2010
Male
Female
Prevalence of MSVI
Age
10
20
30
40
50
60
70
0
20
40
60
80

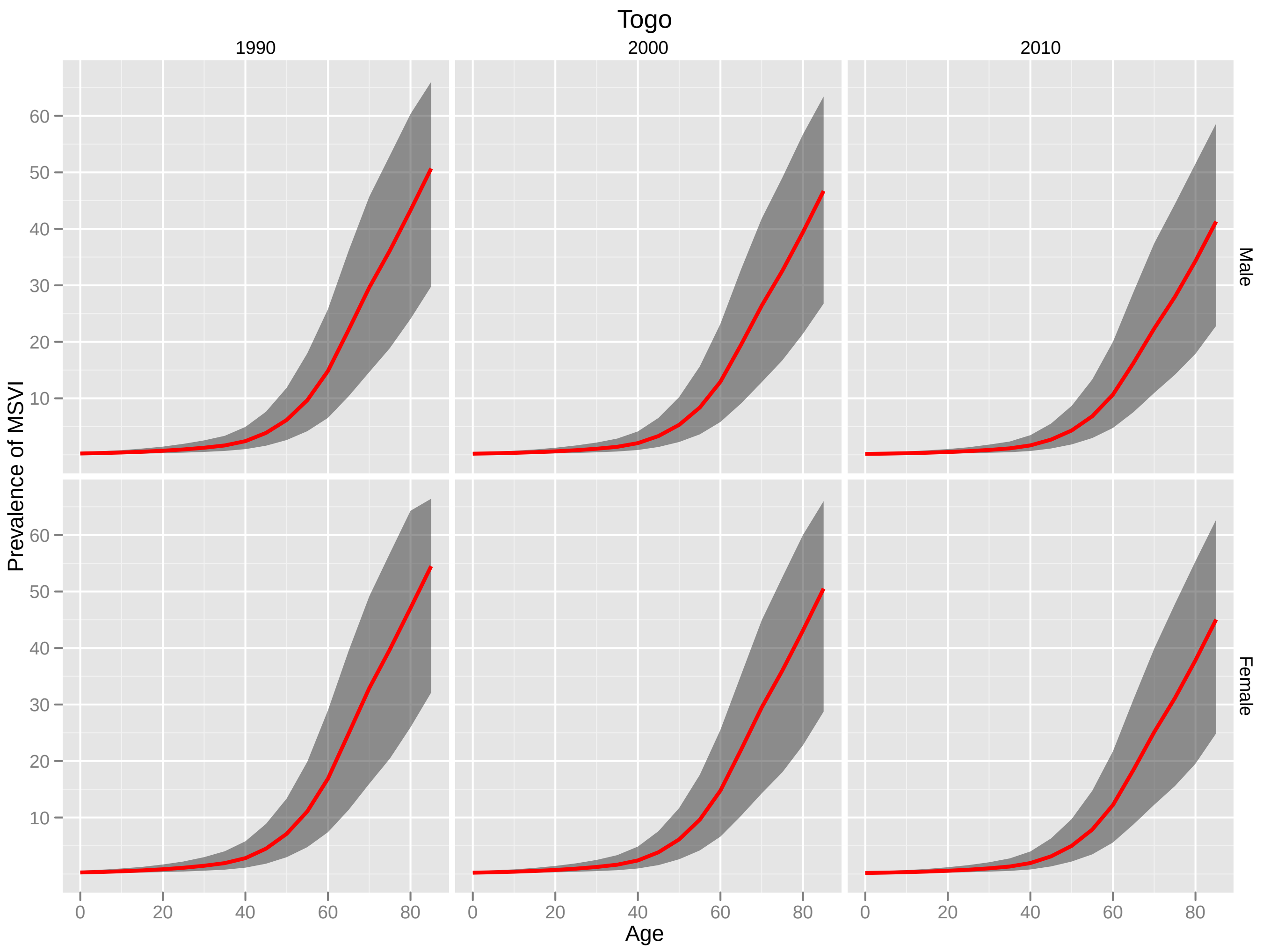
Togo
1990
2000
2010
Male
Female
Prevalence of MSVI
Age
10
20
30
40
50
60
0
20
40
60
80

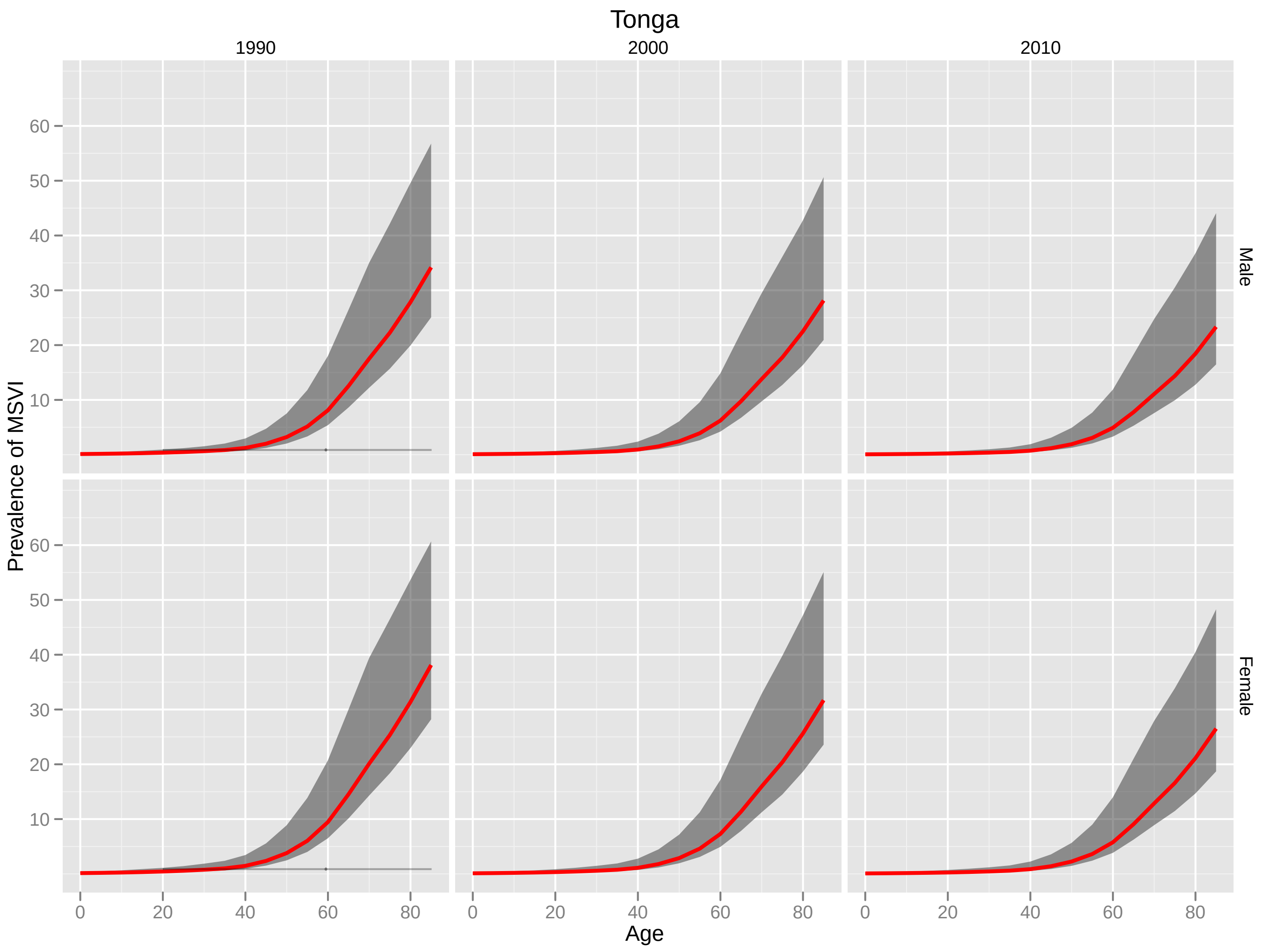

Tonga
1990
2000
2010
Male
Female
Prevalence of MSVI
Age
10
20
30
40
50
60
0
20
40
60
80

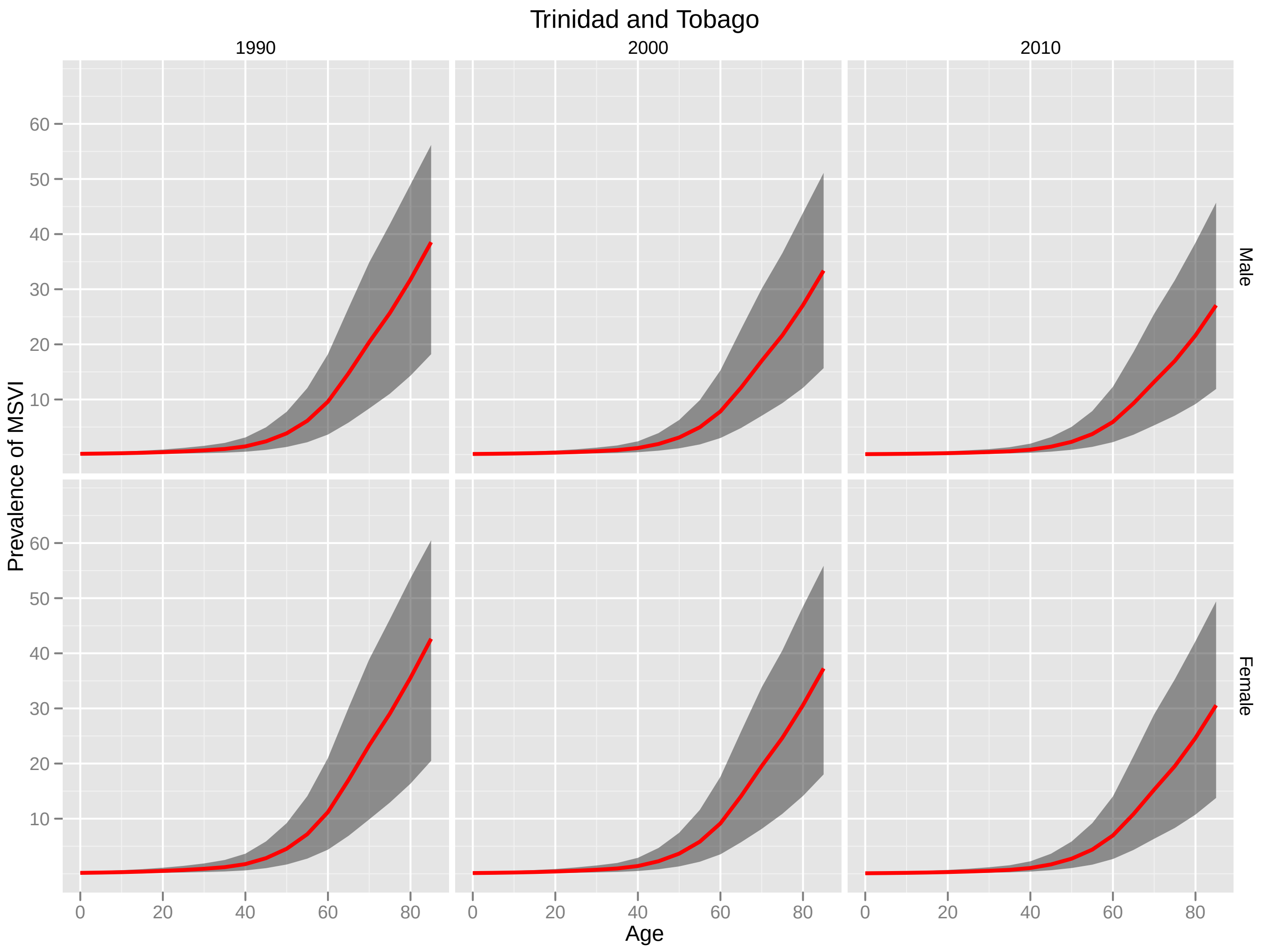
Trinidad and Tobago
1990
2000
2010
Male
Female
Prevalence of MSVI
Age
0
20
40
60
80
10
20
30
40
50
60

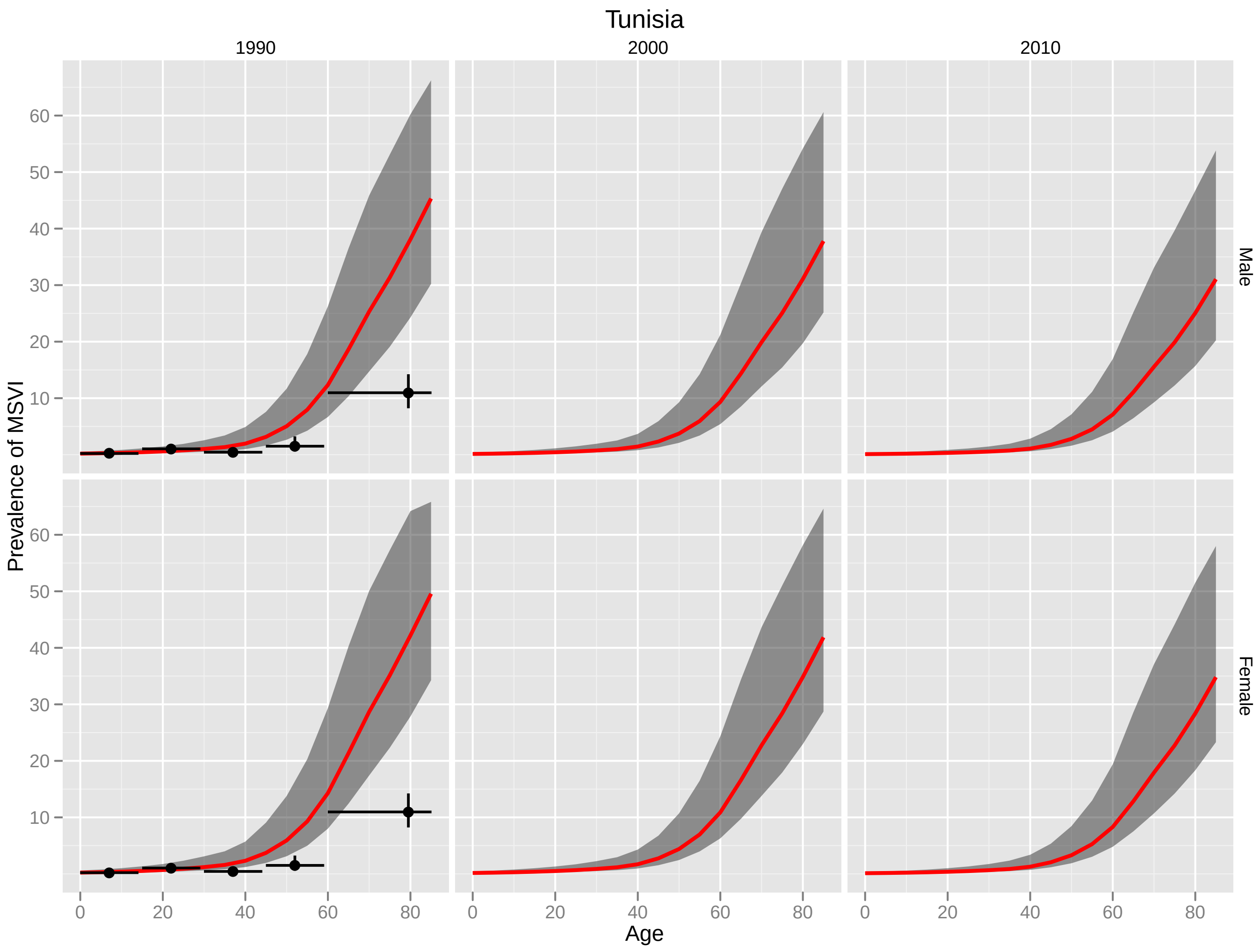
Tunisia
1990
2000
2010
Male
Female
Prevalence of MSVI
Age
10
20
30
40
50
60
0
20
40
60
80

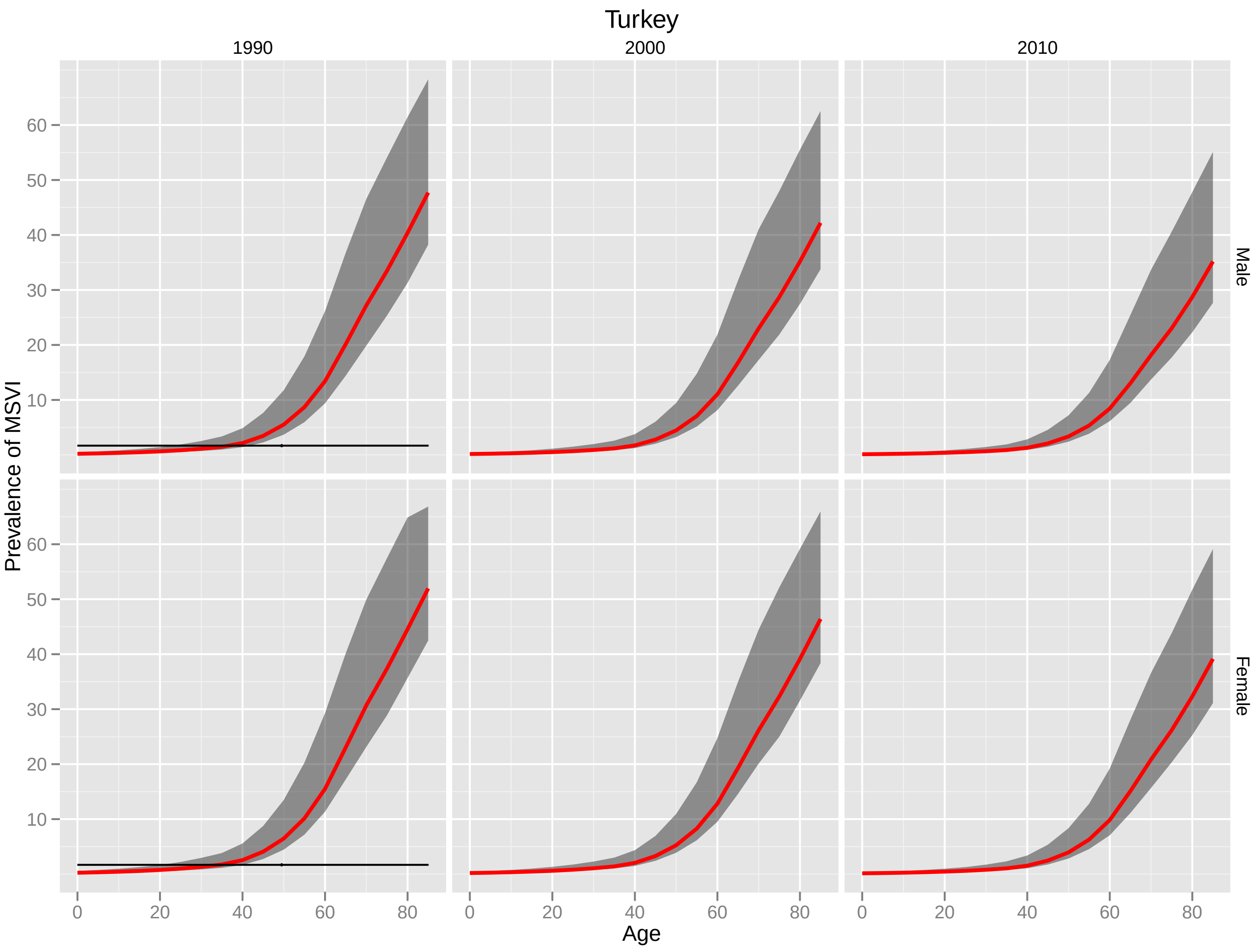
Turkey
1990
2000
2010
Male
Female
Prevalence of MSVI
Age
10
20
30
40
50
60
0
20
40
60
80

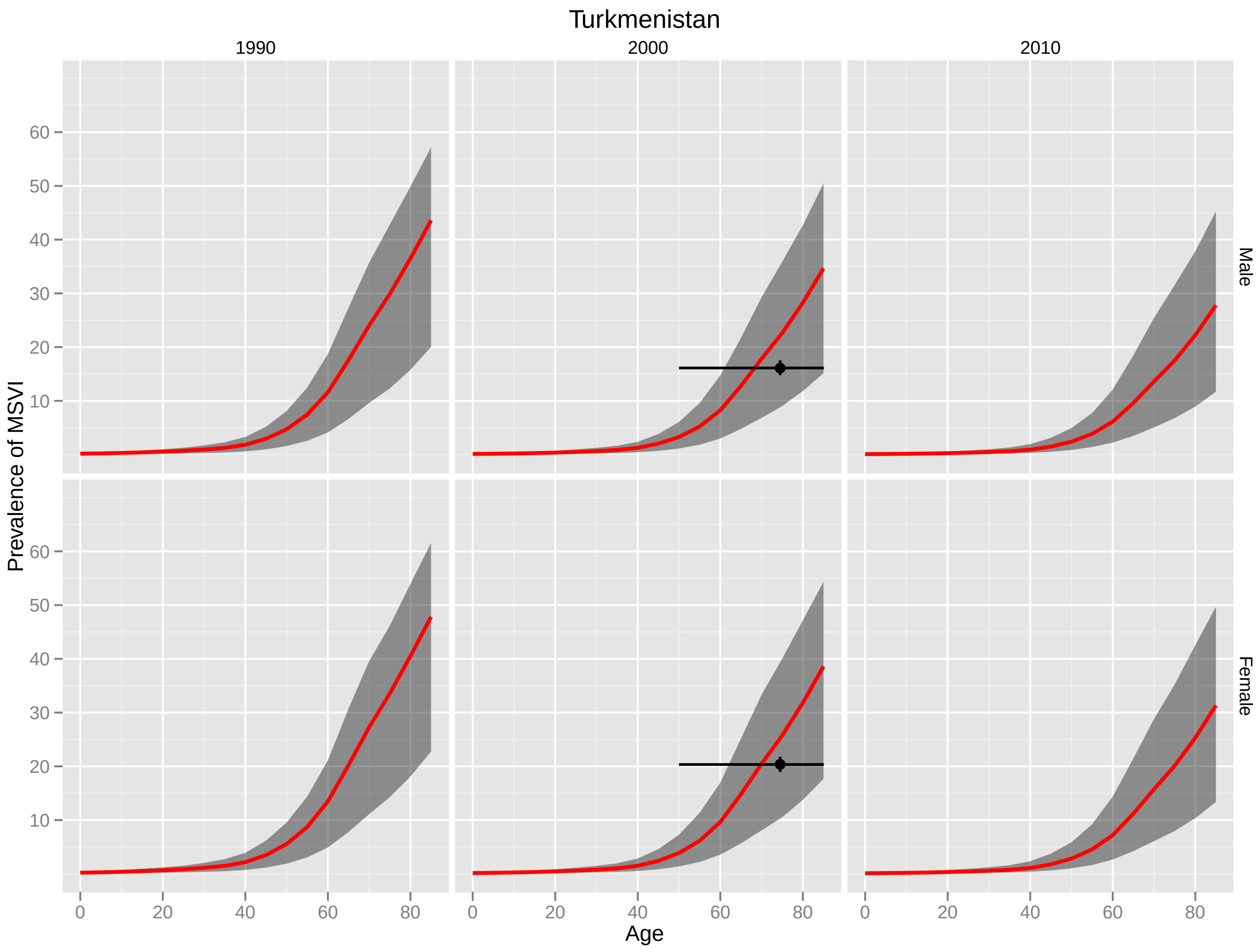
Turkmenistan
1990
2000
2010
Male
Female
Prevalence of MSVI
Age
10
20
30
40
50
60
0
20
40
60
80

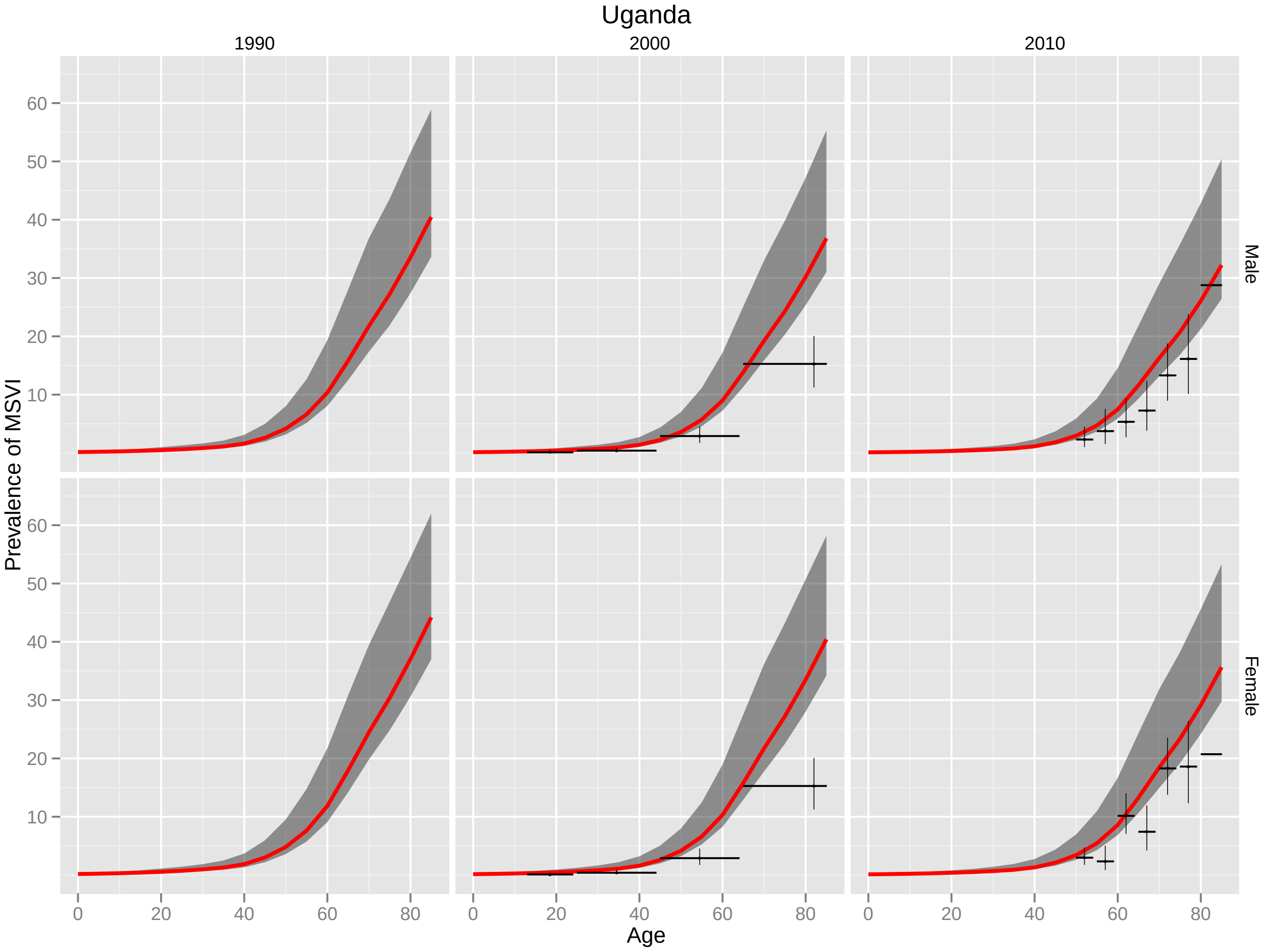

Uganda
1990
2000
2010
Male
Female
Prevalence of MSVI
Age
0
20
40
60
80
10
20
30
40
50
60

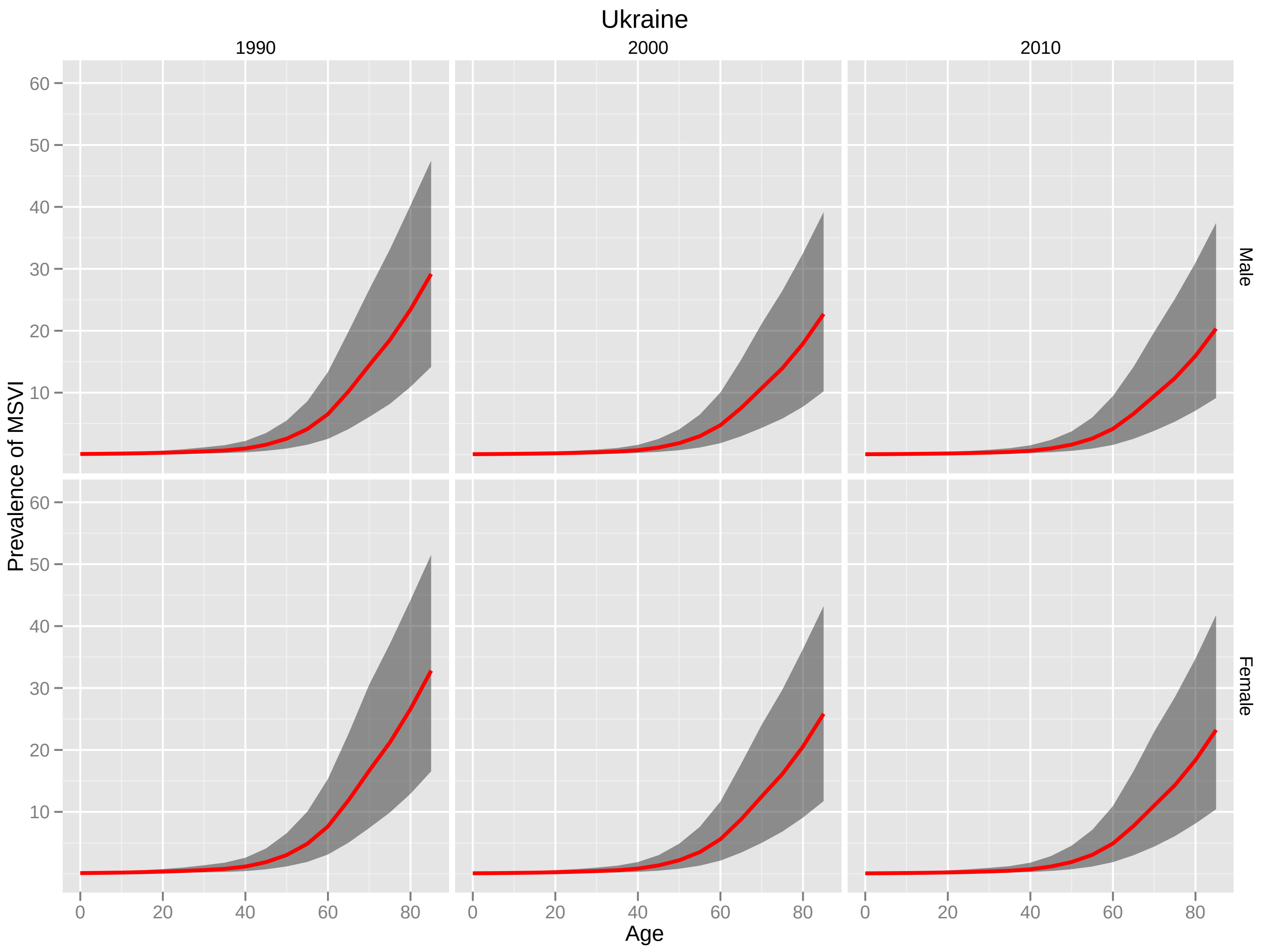
Ukraine
1990
2000
2010
Male
Female
Prevalence of MSVI
Age
0
20
40
60
80
10
20
30
40
50
60

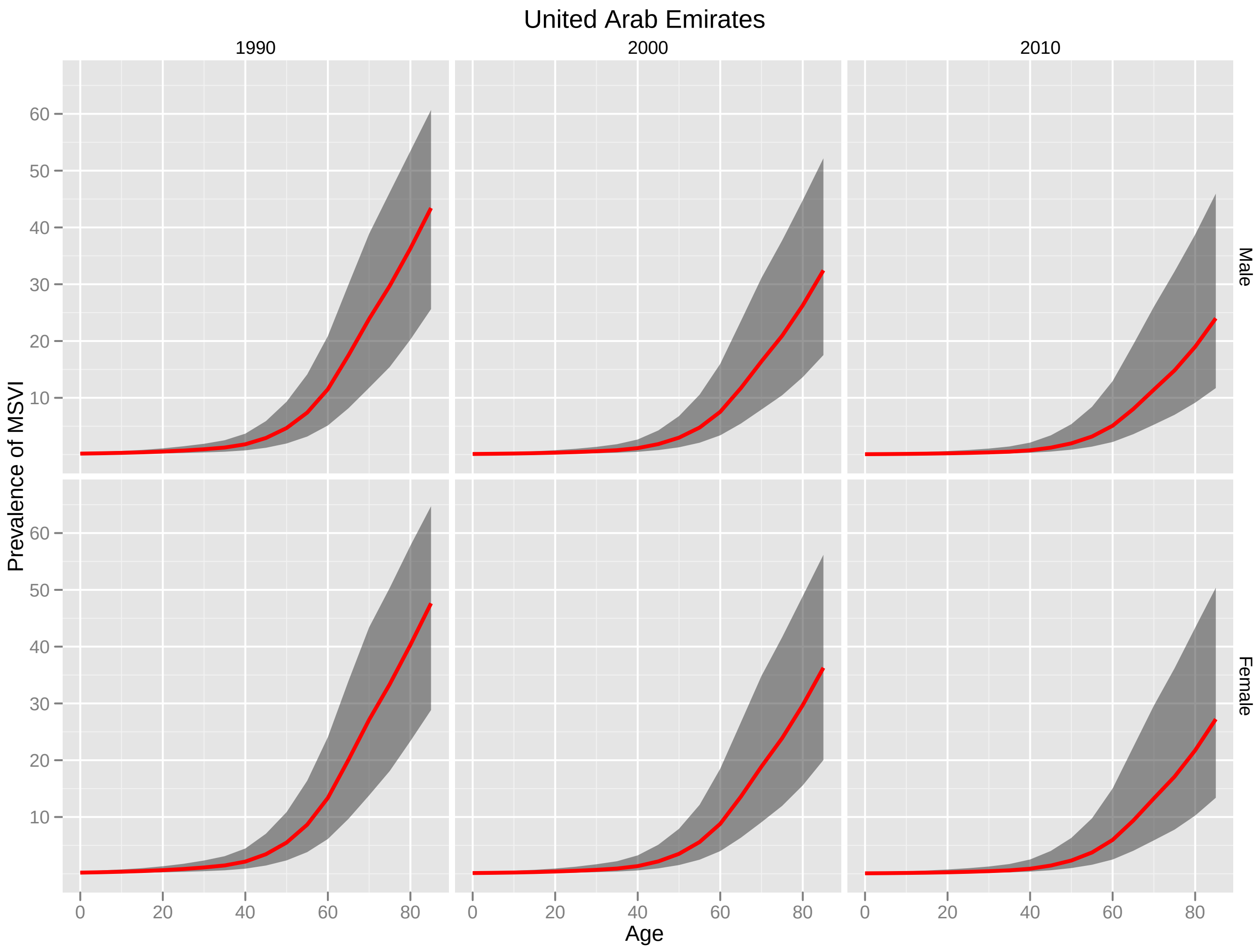

United Arab Emirates
1990
2000
2010
Male
Female
Prevalence of MSVI
Age
10
20
30
40
50
60
0
20
40
60
80

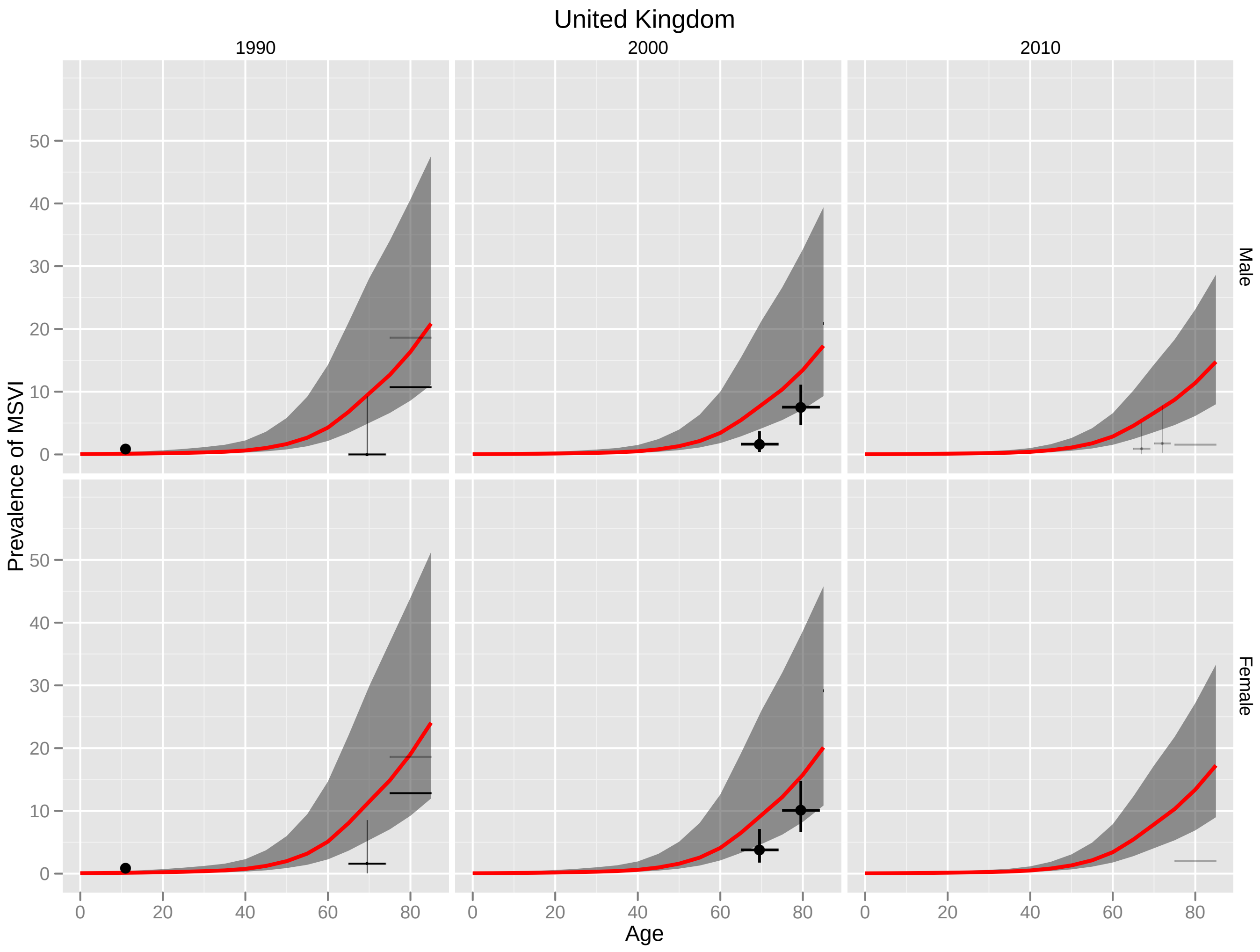
United Kingdom
1990
2000
2010
Male
Female
Prevalence of MSVI
Age
0
10
20
30
40
50
0
20
40
60
80

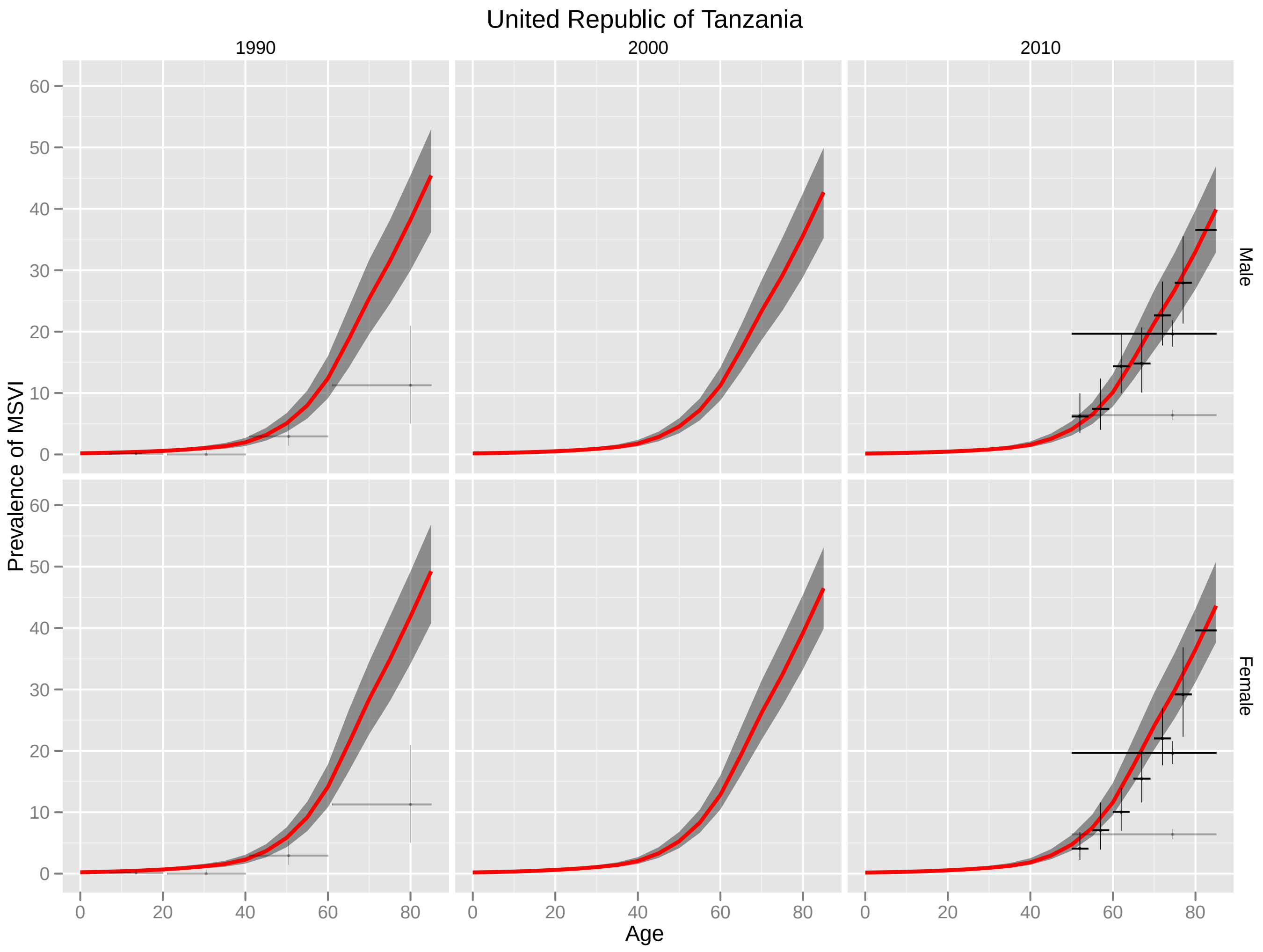
United Republic of Tanzania
1990
2000
2010
Male
Female
Prevalence of MSVI
Age
0
10
20
30
40
50
60
0
20
40
60
80

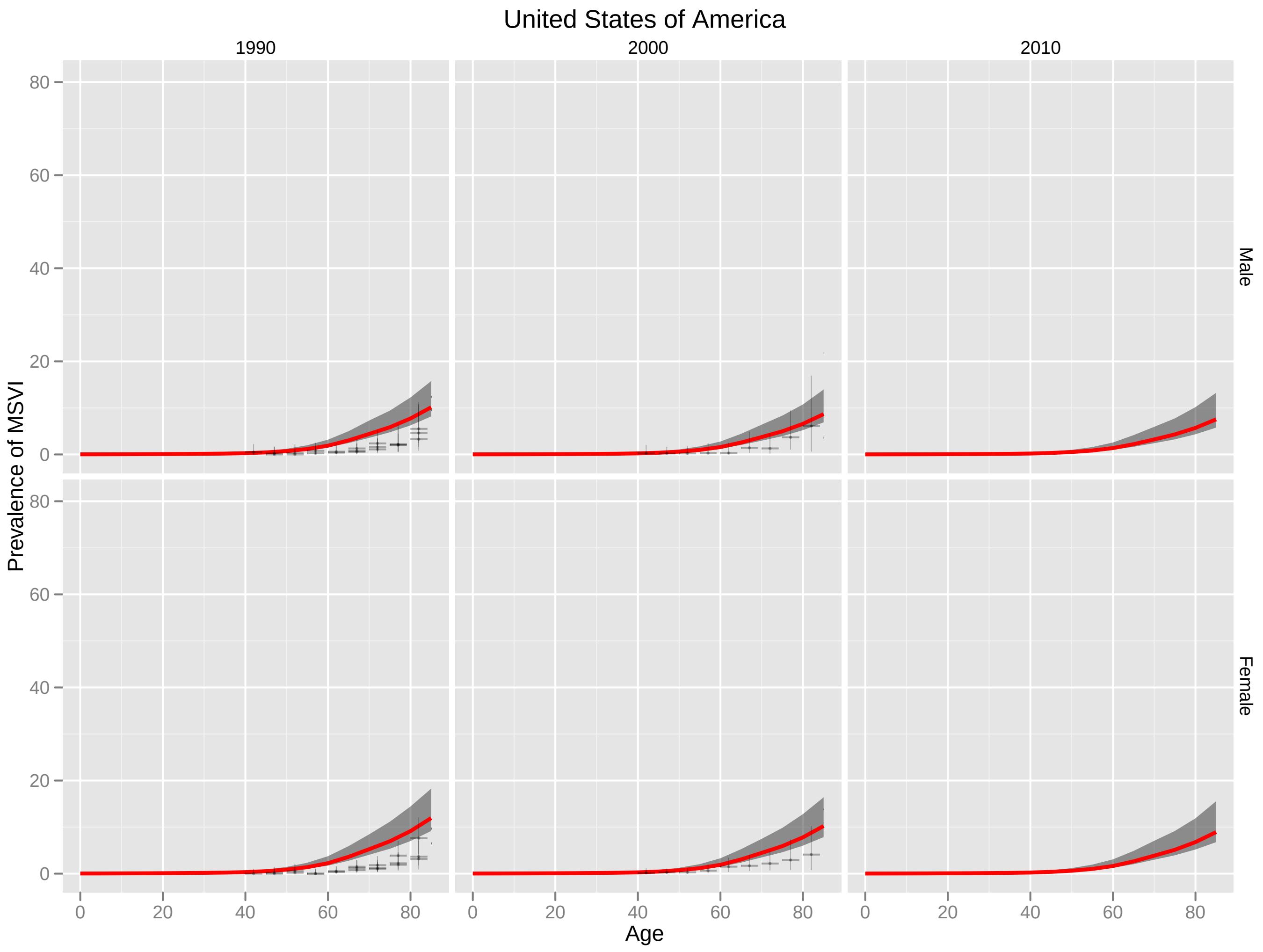
United States of America
1990
2000
2010
Male
Female
Prevalence of MSVI
Age
0
20
40
60
80

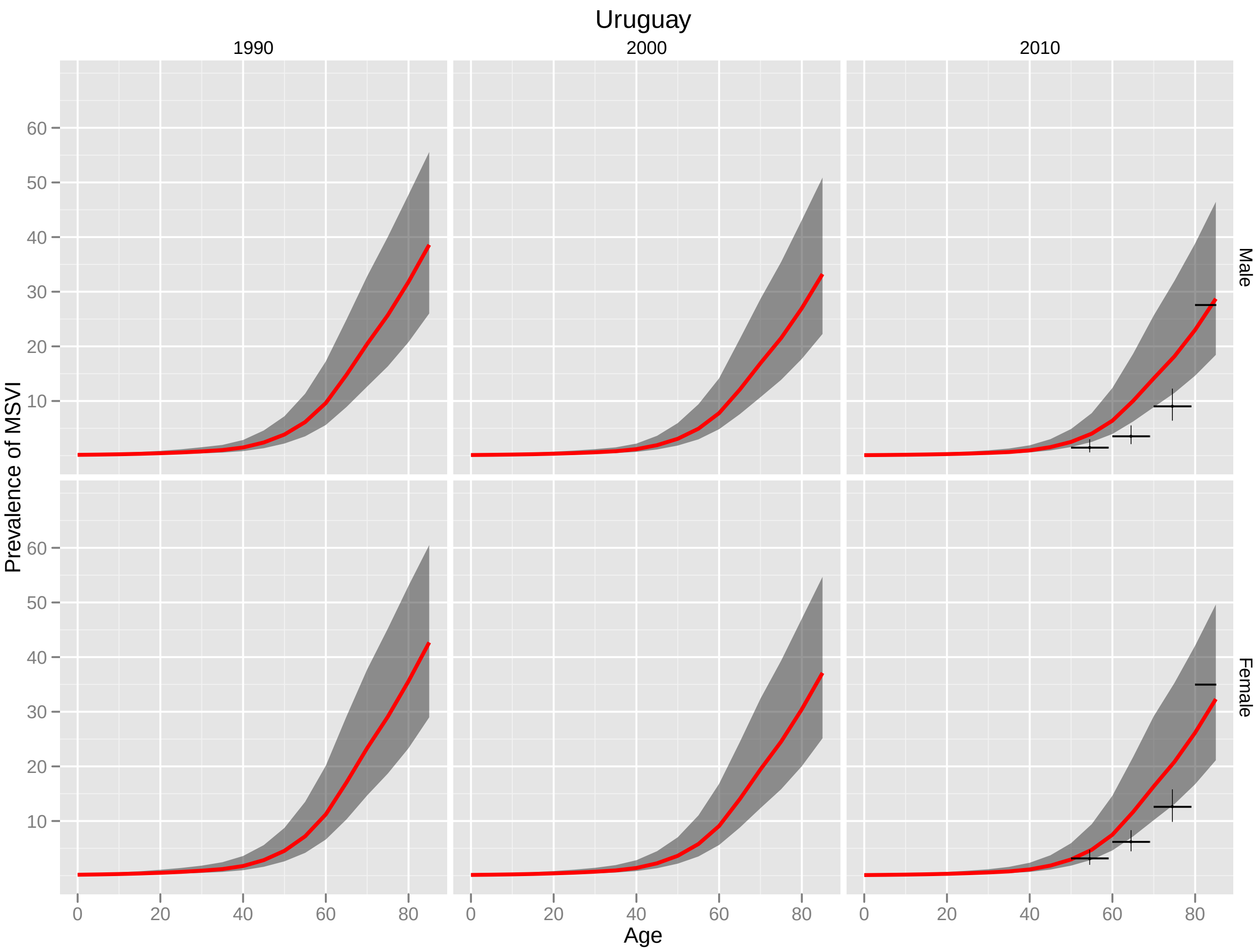
Uruguay
1990
2000
2010
Male
Female
Prevalence of MSVI
Age
10
20
30
40
50
60
0
20
40
60
80

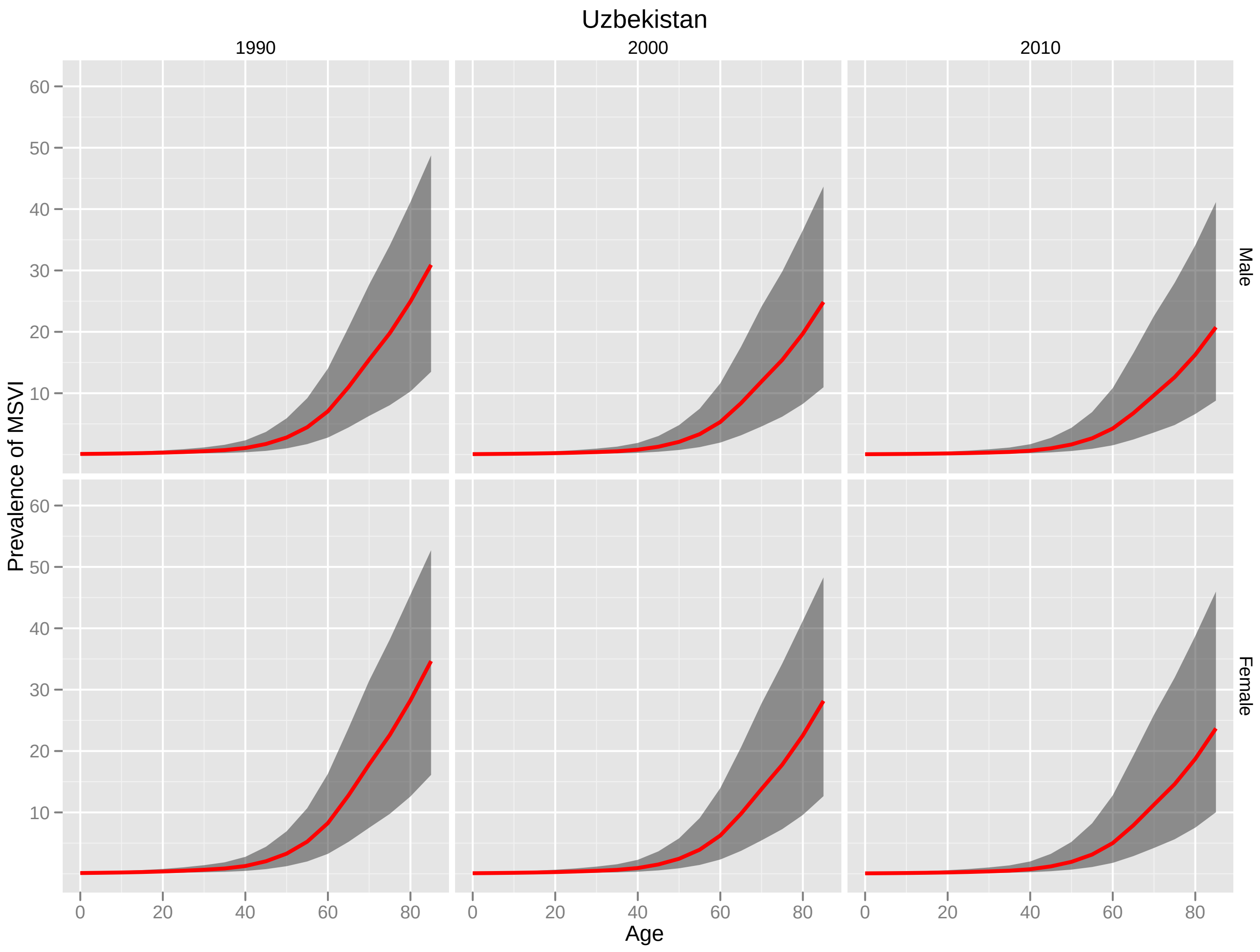
Uzbekistan
1990
2000
2010
Male
Female
Prevalence of MSVI
Age
10
20
30
40
50
60
0
20
40
60
80

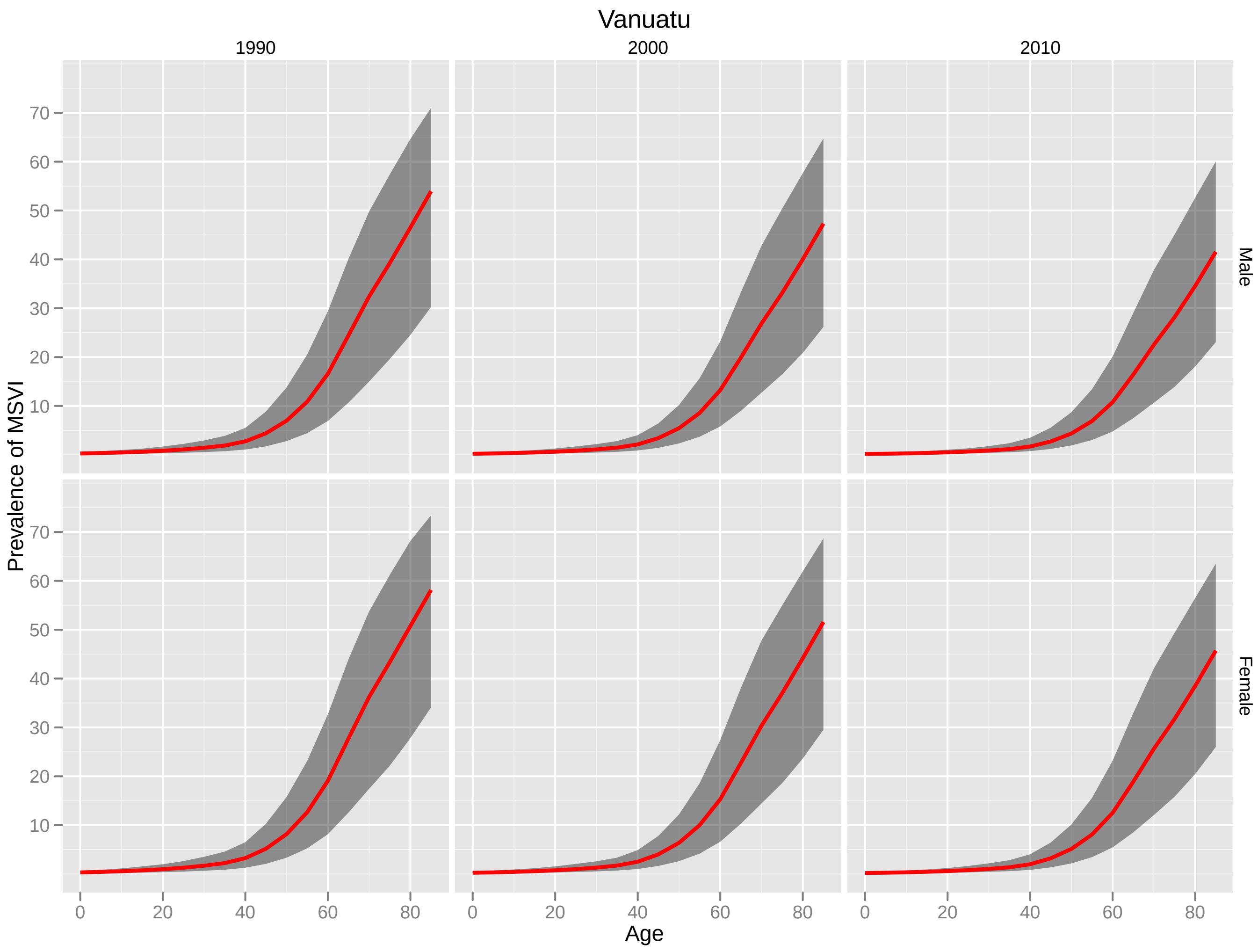
Vanuatu
1990
2000
2010
Male
Female
Prevalence of MSVI
Age
70
60
50
40
30
20
10
0
20
40
60
80

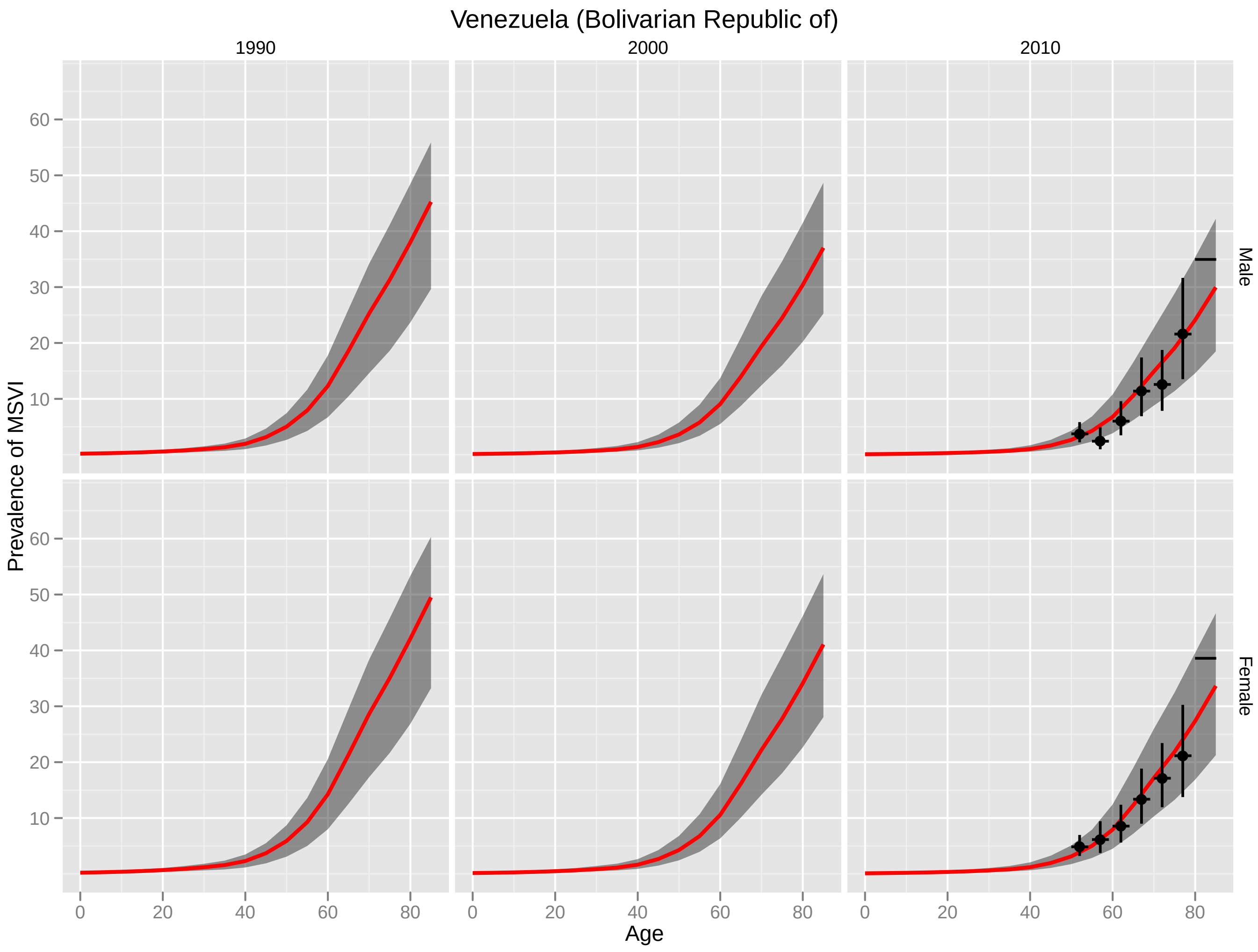
Venezuela (Bolivarian Republic of)
1990
2000
2010
Male
Female
Prevalence of MSVI
Age
10
20
30
40
50
60
0
20
40
60
80

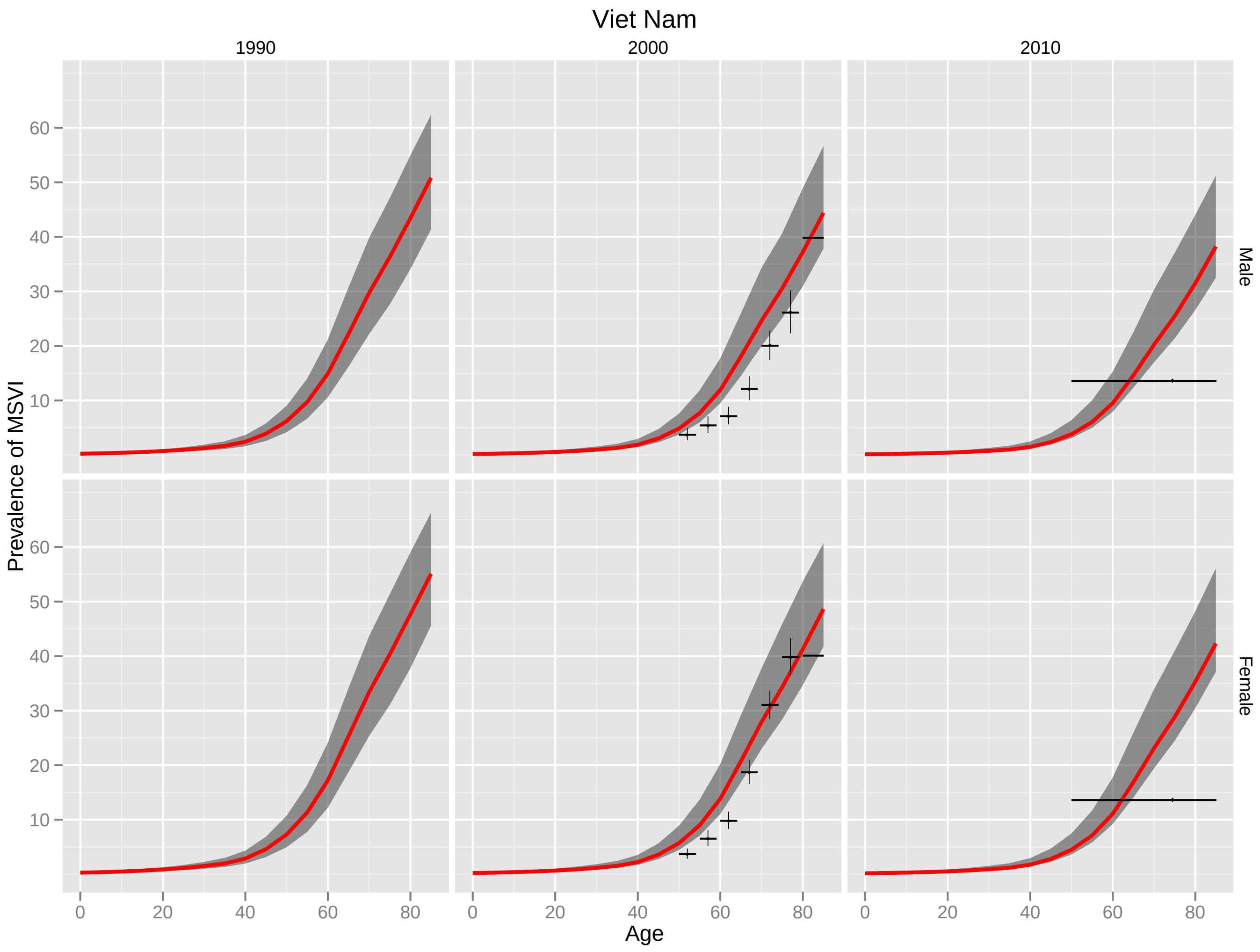
Viet Nam
1990
2000
2010
Male
Female
Prevalence of MSVI
Age
0
20
40
60
80
10
20
30
40
50
60

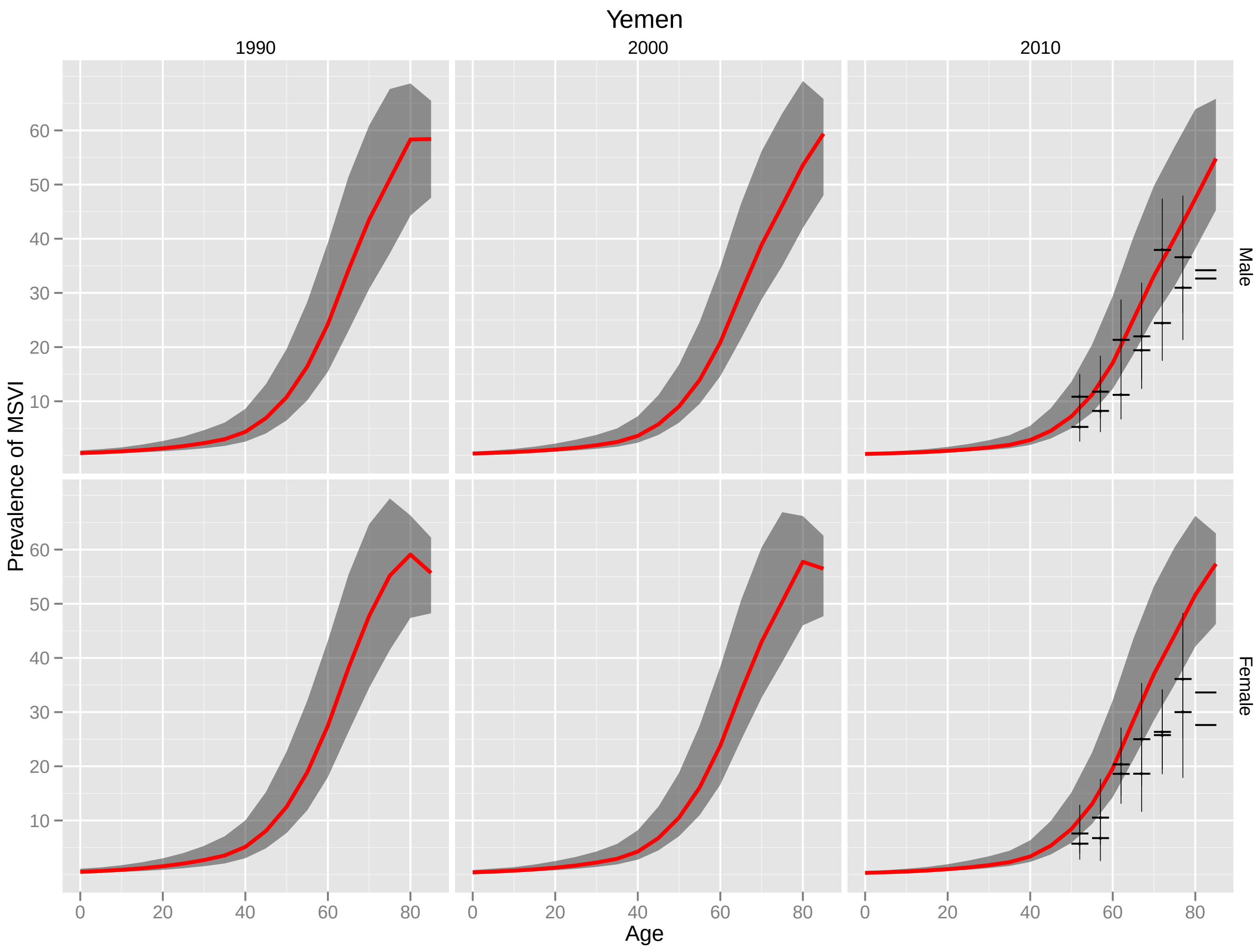
Yemen
1990
2000
2010
Male
Female
Prevalence of MSVI
Age
0
20
40
60
80
10
20
30
40
50
60

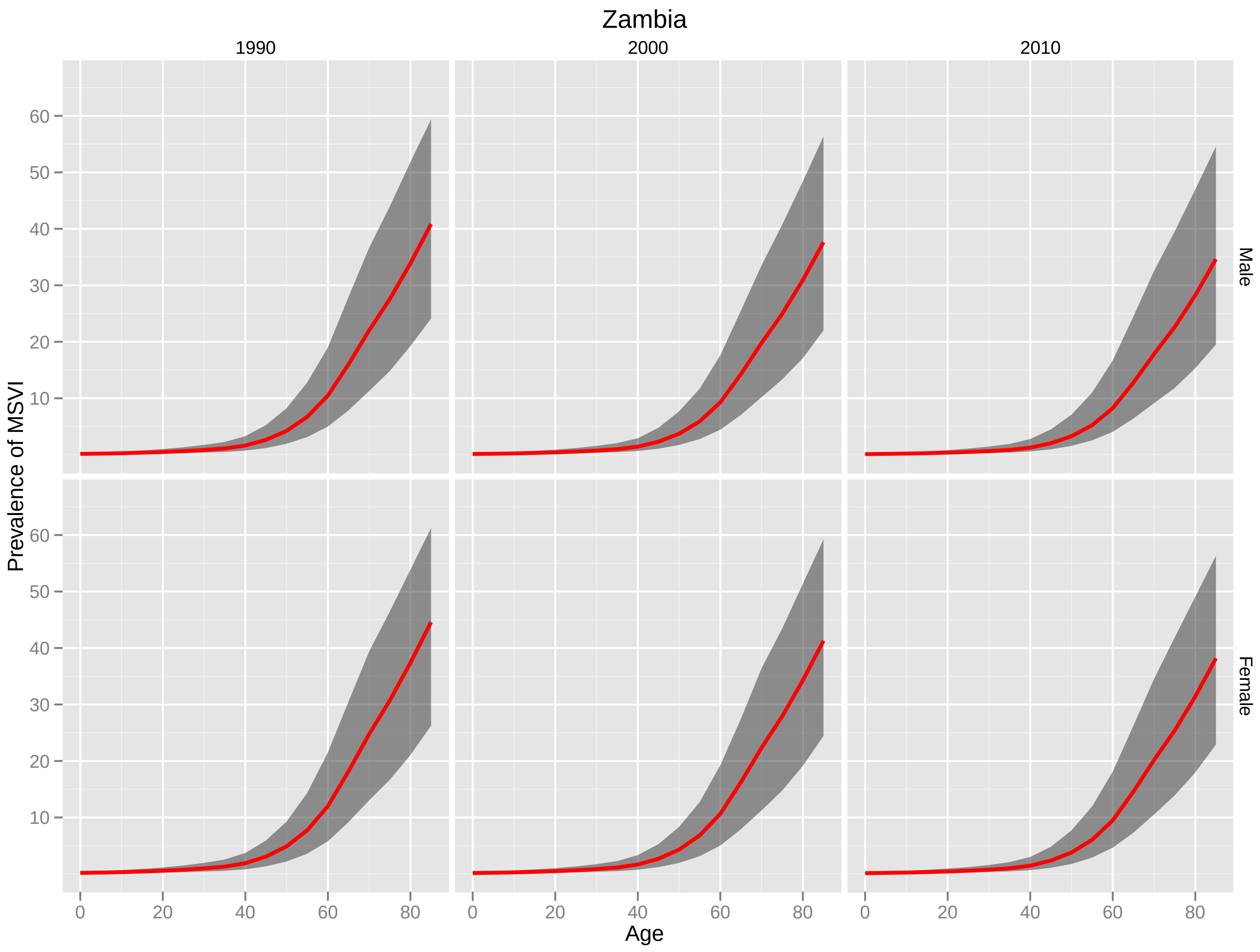
Zambia
1990
2000
2010
Male
Female
Prevalence of MSVI
Age
10
20
30
40
50
60
0
20
40
60
80

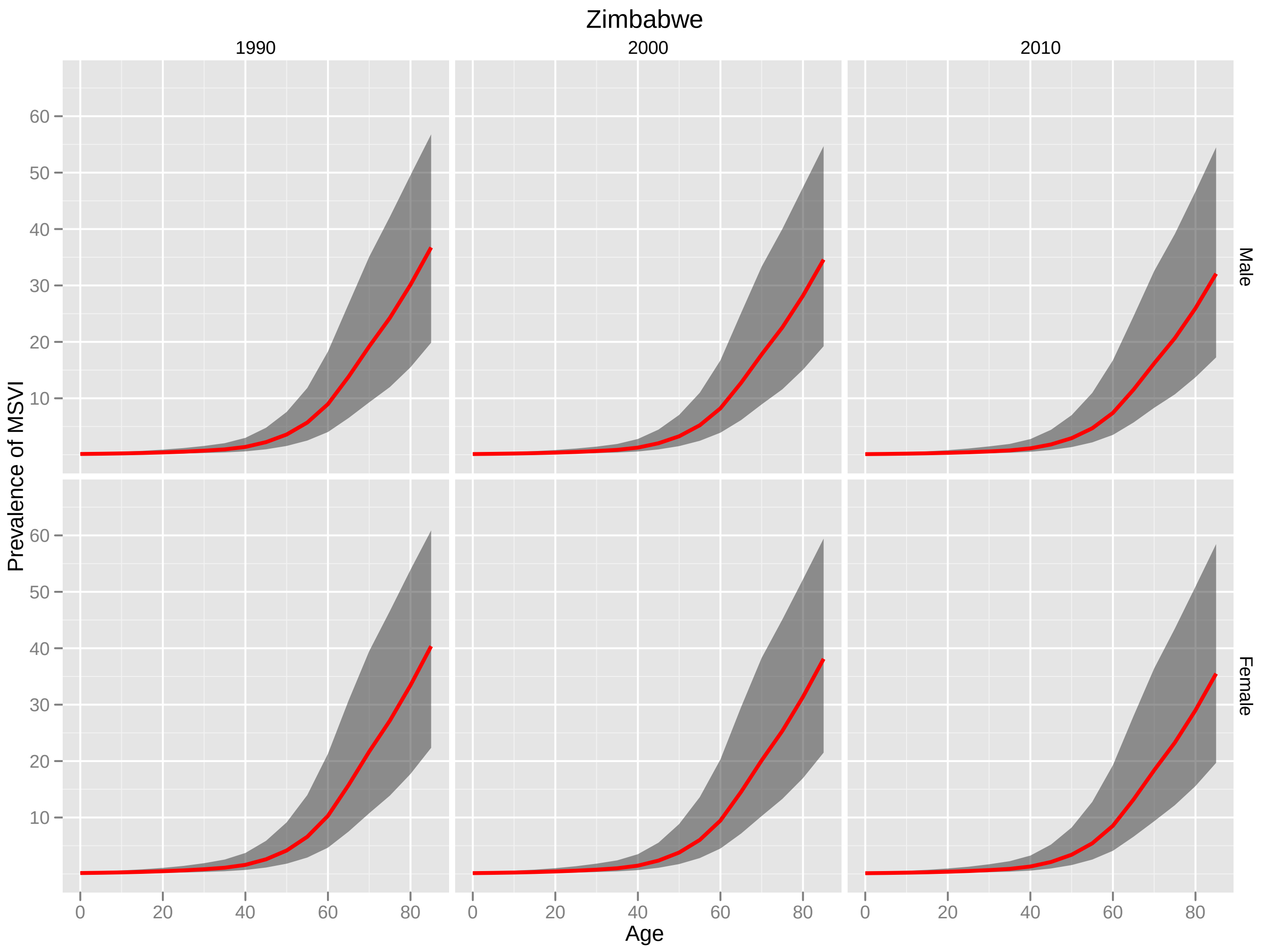
Zimbabwe
1990
2000
2010
Male
Female
Prevalence of MSVI
Age
10
20
30
40
50
60
0
20
40
60
80
